# Derivatives of the Clinically Used HIF Prolyl Hydroxylase Inhibitor Desidustat Are Efficient Inhibitors of Human γ-Butyrobetaine Hydroxylase

**DOI:** 10.1021/acs.jmedchem.5c00586

**Published:** 2025-04-23

**Authors:** Thomas P. Corner, Anthony Tumber, Eidarus Salah, Mohammadparsa Jabbary, Yu Nakashima, Lara I. Schnaubelt, Shyam Basak, Faisal M. Alshref, Lennart Brewitz, Christopher J. Schofield

**Affiliations:** † Chemistry Research Laboratory, Department of Chemistry and the Ineos Oxford Institute for Antimicrobial Research, University of Oxford, 12 Mansfield Road, Oxford OX1 3TA, U.K.; ‡ Institute of Natural Medicine, University of Toyama, 2630-Sugitani, Toyama 930-0194, Japan; § Department of Biochemistry, Faculty of Science, King AbdulAziz University, Jeddah 21589, Saudi Arabia

## Abstract

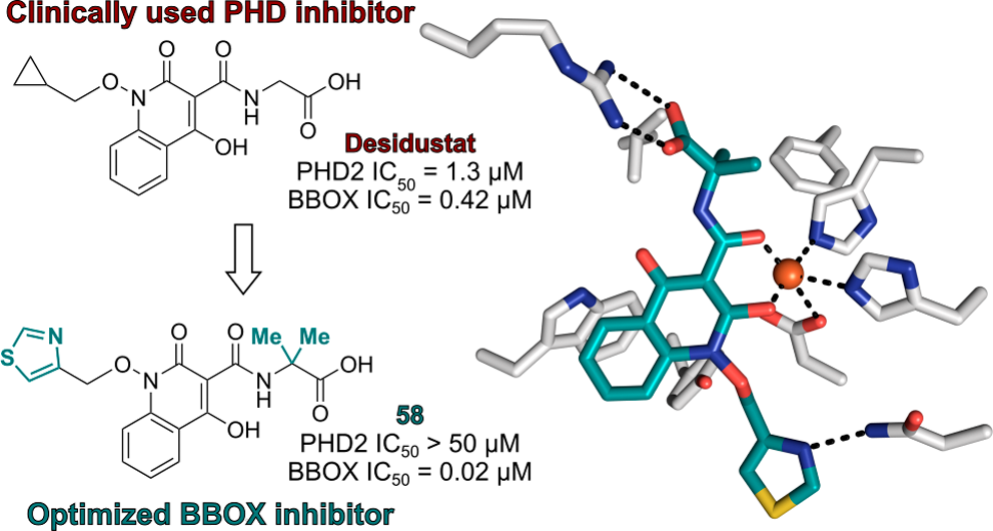

The 2-oxoglutarate (2OG)/Fe(II)-dependent γ-butyrobetaine hydroxylase (BBOX) catalyzes the final step in l-carnitine biosynthesis, *i.e.*, stereoselective C-3 oxidation of γ-butyrobetaine (GBB). BBOX inhibition is a validated clinical strategy to modulate l-carnitine levels and to enhance cardiovascular efficiency. Reported BBOX inhibitors, including the clinically used cardioprotective agent Mildronate, manifest moderate inhibitory activity *in vitro*, limited selectivity, and/or unfavorable physicochemical properties, indicating a need for improved BBOX inhibitors. We report that the clinically used hypoxia-inducible factor-α prolyl residue hydroxylase (PHD) inhibitors Desidustat, Enarodustat, and Vadadustat efficiently inhibit isolated recombinant BBOX, suggesting that BBOX inhibition by clinically used PHD inhibitors should be considered as a possible off-target effect. Structure–activity relationship studies on the Desidustat scaffold enabled development of potent BBOX inhibitors that manifest high levels of selectivity for BBOX inhibition over representative human 2OG oxygenases, including PHD2. The Desidustat derivatives will help to enable investigations into the biological roles of l-carnitine and the therapeutic potential of BBOX inhibition.

## Introduction

Mitochondrial β-oxidation of long-chain fatty acids giving acetyl-coenzyme A (acetyl-CoA) is an important energy source in eukaryotic cells.^1,2^ The transport of fatty acids through the mitochondrial membrane is mediated by l-carnitine via the “carnitine shuttle”.^3−5^l-carnitine thus contributes to regulating the ratio between free- and acyl/acetyl-CoAs within mitochondria, the tight control of which is required for regulation of CoA-dependent processes, including glycolysis and pyruvate oxidation.^3,6^

A reduction in the intracellular concentration of l-carnitine limits fatty acid mitochondrial metabolism, leading to a shift in cellular energy production from fatty acid β-oxidation to peroxisomal metabolism and glucose oxidation, a change which can improve cardiac efficiency, in particular under ischemic conditions.^7−9^ Genome-wide association studies indicate a correlation between l-carnitine and cardiovascular disease in men,^10^ potentially due to formation of its metabolite trimethylamine *N*-oxide (TMAO), which is proposed to promote atherosclerosis.^11,12^ However, the role of l-carnitine in human health is incompletely understood and is likely multifaceted.^13^l-carnitine supplementation is also associated with cardiovascular and other health benefits, e.g., improved insulin sensitivity,^14−17^ and mutations in the *SLC22A5* gene resulting in l-carnitine deficiency can cause cardiomyopathy.^18,19^

**Figure 1:**
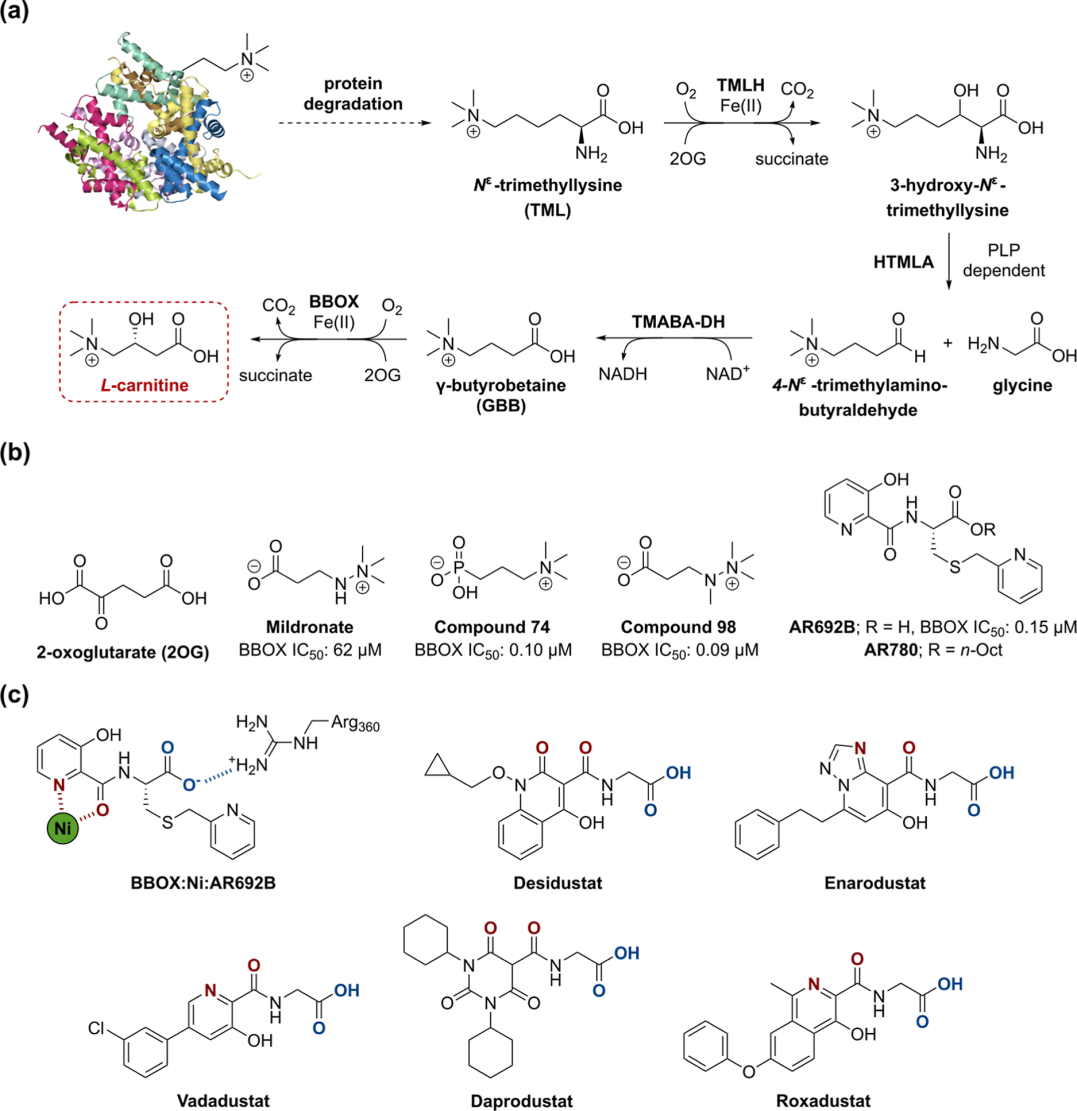
The l-carnitine biosynthesis pathway and selected reported 2OG oxygenase inhibitors. (a) l-Carnitine (red box) is endogenously synthesized from *N*^ε^-trimethyllysine.^21,22^ The 2-oxoglutarate (2OG) oxygenase γ-butyrobetaine hydroxylase (BBOX) catalyzes β-hydroxylation of γ-butyrobetaine (GBB) to give l-carnitine.^23−25^ (b) 2OG and the reported BBOX inhibitors: Mildronate,^31^ compound 74,^7^ compound 98,^7^ AR692B,^36^ and AR780 (*i.e.*, the *n*-octyl prodrug form of AR692B).^36^ (c) AR692B is crystallographically observed to chelate Ni (used as an inert Fe(II) surrogate) at the BBOX active site and to interact with the side chain of Arg360, which binds the 2OG C-5 carboxylate, via its carboxylate group (in blue).^36,39^ The clinically used PHD inhibitors Desidustat,^38,40^ Enarodustat,^41,42^ Vadadustat,^43,44^ Daprodustat,^45,46^ and Roxadustat^47,48^ are observed or predicted to coordinate Fe(II) at the active sites of 2OG oxygenases (Fe(II)-binding motifs are in red).^49−51^ These five PHD inhibitors also contain carboxylate groups (in blue) observed or predicted to interact with PHD residues that bind to the 2OG C-5 carboxylate (*i.e.*, Tyr329 and Arg383).^49−51^ TMLH, *N*^ε^-trimethyllysine hydroxylase; HTMLA, 3-hydroxy-*N*^ε^-trimethyllysine aldolase; PLP, pyridoxal phosphate; TMABA-DH, 4-*N*^ε^-trimethylaminobutyraldehyde dehydrogenase.

The concentration of l-carnitine in healthy cells is regulated through dietary intake and endogenous biosynthesis;^20,21^ ∼25% of l-carnitine in human cells is produced from *N*^ε^-trimethyllysine (TML; Figure 1a), formed via degradation of proteins that contain *N*^ε^-trimethylated lysine residues.^21,22^ The final step of l-carnitine biosynthesis, i.e., the stereoselective C-3 hydroxylation of γ-butyrobetaine (GBB), is catalyzed in the cytosol by the 2-oxoglutarate (2OG)- and Fe(II)-dependent oxygenase γ-butyrobetaine hydroxylase (BBOX).^23−25^ Pharmacological inhibition of BBOX is reported to reduce l-carnitine levels in cells and in vivo, which is of interest for the treatment of cardiovascular diseases, and to investigate the biological functions of l-carnitine.^9,26,27^ In addition to its role in l-carnitine biosynthesis, BBOX is proposed to contribute to triple negative breast cancer (TNBC) cell growth through its interactions with inositol-1,4,5-trisphosphate receptor type 3 (IP3R3); thus, BBOX is a potential target for TNBC treatment.^28,29^

The GBB analogue Mildronate (THP, MET-88, and Meldonium), which is both a substrate and a GBB-competitive BBOX inhibitor,^30^ is used clinically as a cardioprotective agent.^26,31,32^ Mildronate is a relatively weak inhibitor/competitive substrate of isolated BBOX in vitro (reported IC_50_: 62 μM);^30,33^ structure–activity relationship (SAR) studies investigating the effect of modifications to its trimethylammonium moiety, ethylene core, and carboxylate group have identified Mildronate/GBB analogues with improved BBOX inhibition potency (*i.e.*, compound 74 and compound 98; Figure 1b).^7^ Nonetheless, the observations that BBOX can accept Mildronate as a substrate to generate multiple products, including (potentially toxic) formaldehyde,^30^ and that Mildronate/GBB-derived BBOX inhibitors inhibit other GBB- and l-carnitine-interacting proteins,^34,35^ highlights a need for mechanistically distinct BBOX inhibitors.

The 3-hydroxypyridine derivative AR692B (Figure 1b) is an efficient 2OG-competitive inhibitor of isolated BBOX that manifests high levels of selectivity for inhibition of BBOX over other human 2OG oxygenases, including the hypoxia-inducible factor-α (HIF-α) prolyl hydroxylase domain-containing protein 2 (PHD2), factor inhibiting HIF-α (FIH), and JmjC histone *N*^ε^-lysine demethylases (KDMs).^36^ Although AR692B provides a proof-of-concept that isolated BBOX can be efficiently inhibited by 2OG-competing inhibitors with high selectivity, it does not interact with the GBB substrate-binding pocket of BBOX, suggesting that more efficient BBOX inhibitors can be identified. The thioether group of AR692B may be susceptible to metabolic oxidation,^37^ which may impact on its BBOX inhibitory activity in cells and/or whole organisms. AR692B is substantially more potent (>10-fold) in cells as its *n*-octyl ester prodrug form (AR780; Figure 1b),^36^ indicating that the cellular efficacy of AR692B may be limited by incomplete cellular uptake. Note that the use of AR780 is likely limited to cell lines containing suitable esterases, since hydrolysis of its *n*-octyl ester group is necessary for potent BBOX inhibition.^36^ Thus, the combined evidence indicates that improved BBOX inhibitors are desirable in order to support cellular and *in vivo* BBOX functional assignment studies and investigate the therapeutic relevance of endogenous l-carnitine depletion.

A search for novel BBOX inhibitors revealed that several clinically used PHD inhibitors manifest efficient inhibition of isolated recombinant BBOX and thus represent promising scaffolds for BBOX inhibitor development, given their use as therapeutics. Computationally guided structural optimization enabled the discovery of potent 2OG-competing BBOX inhibitors derived from the approved PHD inhibitor Desidustat.^38^ Mass spectrometric studies indicate that the optimized Desidustat derivatives exhibit excellent levels of selectivity over a representative panel of structurally diverse 2OG oxygenases, including PHD2.

## Results

### Clinically Used PHD Inhibitors Efficiently Inhibit BBOX *In Vitro*

AR692B is structurally related to PHD inhibitors that are used clinically for the treatment of chronic kidney disease (CKD)-associated anemia,^36,51,52^ including Desidustat,^38,40^ Enarodustat,^41,42^ Vadadustat,^43,44^ Daprodustat,^45,46^ and Roxadustat^47,48^ (Figure 1c). Like AR692B, these five PHD inhibitors contain both a bidentate metal-binding motif, which is crystallographically observed or computationally predicted to chelate Fe(II) at the active sites of 2OG oxygenases, and a carboxylate group, binding of which mimics that of the C-5 carboxylate group of 2OG.^49−51,53^

Since Desidustat, Enarodustat, Vadadustat, Daprodustat, and Roxadustat manifest pharmacokinetic and toxicity profiles suitable for clinical use,^49,54−59^ and their modular structures are amenable to systematic derivatization, we envisaged that they would be attractive lead scaffolds for the development of improved BBOX inhibitors suitable for cellular and *in vivo* applications. We therefore investigated the effect of these five PHD inhibitors on the activity of isolated recombinant BBOX. BBOX inhibition was evaluated using hydrophilic interaction liquid chromatography (HILIC) solid-phase extraction coupled to mass spectrometry (SPE-MS)-based BBOX inhibition assays, which monitor the change in mass of GBB (*i.e.*, +16 Da) upon its hydroxylation by BBOX. The HILIC SPE-MS assays were preferred over reported fluoride-detection-based fluorescence^36,60^ and ligand-based NMR BBOX assays,^61^ since they, unlike the fluorescence assays, provide a direct measurement of BBOX-catalyzed GBB hydroxylation while enabling a higher sample throughput than the NMR assays.

The SPE-MS results indicated that all of the five tested PHD inhibitors manifested BBOX inhibition, with IC_50_ values ranging from ∼0.1 to ∼13 μM (Table 1). The most potent BBOX inhibitor identified was Enarodustat (IC_50_ ∼ 0.10 μM; Table 1, entry iv), which is approved for the treatment of CKD-associated anemia in Japan.^58,62^ Notably, Enarodustat manifested ∼5-fold more efficient BBOX inhibition in the SPE-MS inhibition assays than AR692B (IC_50_ ∼ 0.52 μM; Table 1, entry i), and >500-fold more efficient BBOX inhibition than clinically used Mildronate (IC_50_ > 50 μM; Table 1, entry ii). Desidustat,^38,40^ which is approved for clinical use in India and China,^38,63,64^ and Vadadustat, which is approved for clinical use in 37 countries,^65,66^ also efficiently inhibited BBOX catalysis (IC_50_ values: ∼0.42 and ∼0.28 μM, respectively; Table 1, entries iii and v), while Daprodustat and Roxadustat were identified as moderately potent BBOX inhibitors (IC_50_ values: ∼13 and ∼8.1 μM, respectively; Table 1, entries vi and vii).

**Table 1:**
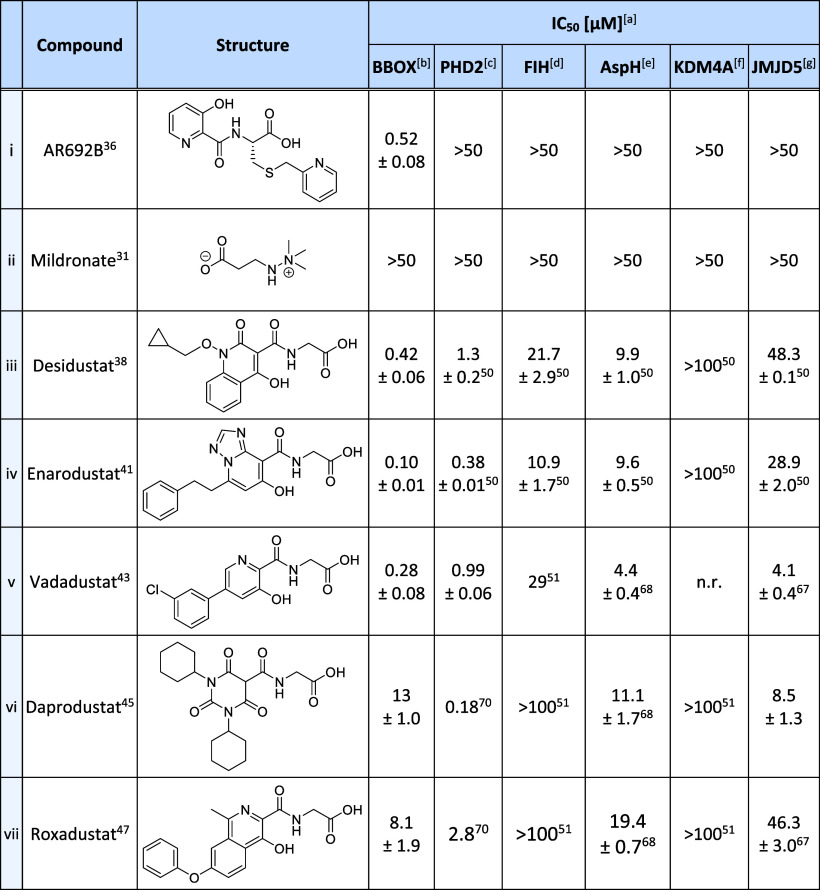
Inhibition of Human 2OG Oxygenases by the Reported BBOX Inhibitors AR692B and Mildronate and by Selected Clinically Used PHD Inhibitors

a IC_50_ values are means ± standard deviation (SD) of independent duplicates (each composed of technical duplicates).

b Using 0.05 μM BBOX, 400 μM 2OG, and 25 μM GBB.

c Using 0.15 μM PHD2_181–426_, 10 μM 2OG, and 5.0 μM HIF-1α CODD_556–574_.^71^

d Using 0.15 μM FIH, 10 μM 2OG, and 5.0 μM HIF-1α C-TAD_788–822_.^71^

e Using 0.05 μM His_6_-AspH_315–758_, 3 μM 2OG, and 1.0 μM of a human Factor X-derived cyclic peptide (hFX-CP_101–119_).^68^

f Using 0.15 μM KDM4A, 10 μM 2OG, and 10 μM of a H3_1–15_K9me3 variant.^72^

g Using 0.15 μM JMJD5, 2 μM 2OG, and 2.0 μM RSP6_128–148_.^67^ Inhibition assays were performed using SPE-MS, as described in the Experimental Section. n.r.: not reported.

We have previously investigated the ability of Enarodustat, Desidustat, and Vadadustat to inhibit isolated human 2OG oxygenases other than PHD2, including representative protein hydroxylases, *i.e.*, FIH, aspartate/asparagine β-hydroxylase (AspH) and Jumonji-C domain-containing protein 5 (JMJD5), and histone *N*^ε^-methyllysine demethylase 4A (KDM4A).^50,53,67,68^ All three PHD inhibitors manifested inhibition of AspH,^50,68^ FIH,^50,51,53^ and JMJD5,^50,67^ in addition to efficient PHD2 inhibition (Table 1, entries iii–v). Notably, Vadadustat efficiently inhibits isolated AspH and JMJD5 (IC_50_ values: ∼4.4 and ∼4.1 μM, respectively; Table 1, entry v). Importantly, the combined results indicate that Enarodustat, Desidustat, and Vadadustat inhibit BBOX ∼3–4-fold more efficiently than PHD2 under SPE-MS assay conditions (Table 1), suggesting that BBOX inhibition should be considered a potential off-target effect of their usage. Note, however, that BBOX is a dimer in solution,^39^ with both monomers likely contributing in a cooperative manner to catalysis,^69^ a property which may complicate direct comparison of IC_50_ values.

For comparison, we investigated the 2OG oxygenase inhibitory activity of the clinically used BBOX inhibitor Mildronate and AR692B (Table 1, entries i and ii). Consistent with previous reports,^36,68^ Mildronate and AR692B manifested negligible inhibition of all tested 2OG oxygenases. The combined inhibition results thus indicated that AR692B is a selective BBOX inhibitor, at least with respect to inhibition of the other isolated human 2OG oxygenases tested, and that selective BBOX inhibitors for safe clinical use can potentially be designed. Desidustat, Enarodustat, and Vadadustat were observed to inhibit a broader subset of isolated 2OG oxygenases *in vitro* than AR692B.

### Protein–Ligand Docking Predicts the BBOX Binding Mode of Desidustat

The combined inhibition results (Table 1) imply that Desidustat, Enarodustat, and Vadadustat could serve, at least in principle, as suitable scaffolds for the development of selective cell-permeable BBOX inhibitors. We chose to prioritize Desidustat for further optimization studies, because its reported synthesis^73^ appeared more amenable to late-stage derivatization than that reported for Enarodustat,^41^ enabling streamlined SAR studies, and because it inhibited AspH and JMJD5 ∼2- and ∼10-fold less efficiently than Vadadustat (Table 1),^67,68^ indicative of a potentially higher selectivity for BBOX inhibition over inhibition of other human 2OG oxygenases.^74^ However, the extent to which biochemical inhibition potency translates to selectivity in cells and *in vivo* is unclear.

Computational studies on the binding mode of Desidustat to BBOX were performed to enable comparison with reported crystal structures of BBOX in complex with AR692B,^36^ GBB,^39^ and the 2OG analogue *N*-oxalylglycine (NOG) and to inform on potential strategies for optimizing its BBOX inhibition and selectivity profile. The potential binding mode of Desidustat to BBOX was predicted by protein–ligand docking, using GOLD 5.1^75^ and an ensemble model of reported BBOX:Ni:AR692B (PDB ID: 4C8R(36)) and BBOX:Zn:NOG:GBB (PDB ID: 3O2G(39)) complex structures. Note that an ensemble model of reported BBOX:inhibitor complex structures was used to best replicate the reported conformational dynamics of the BBOX active site.^36,39,69^

**Figure 2:**
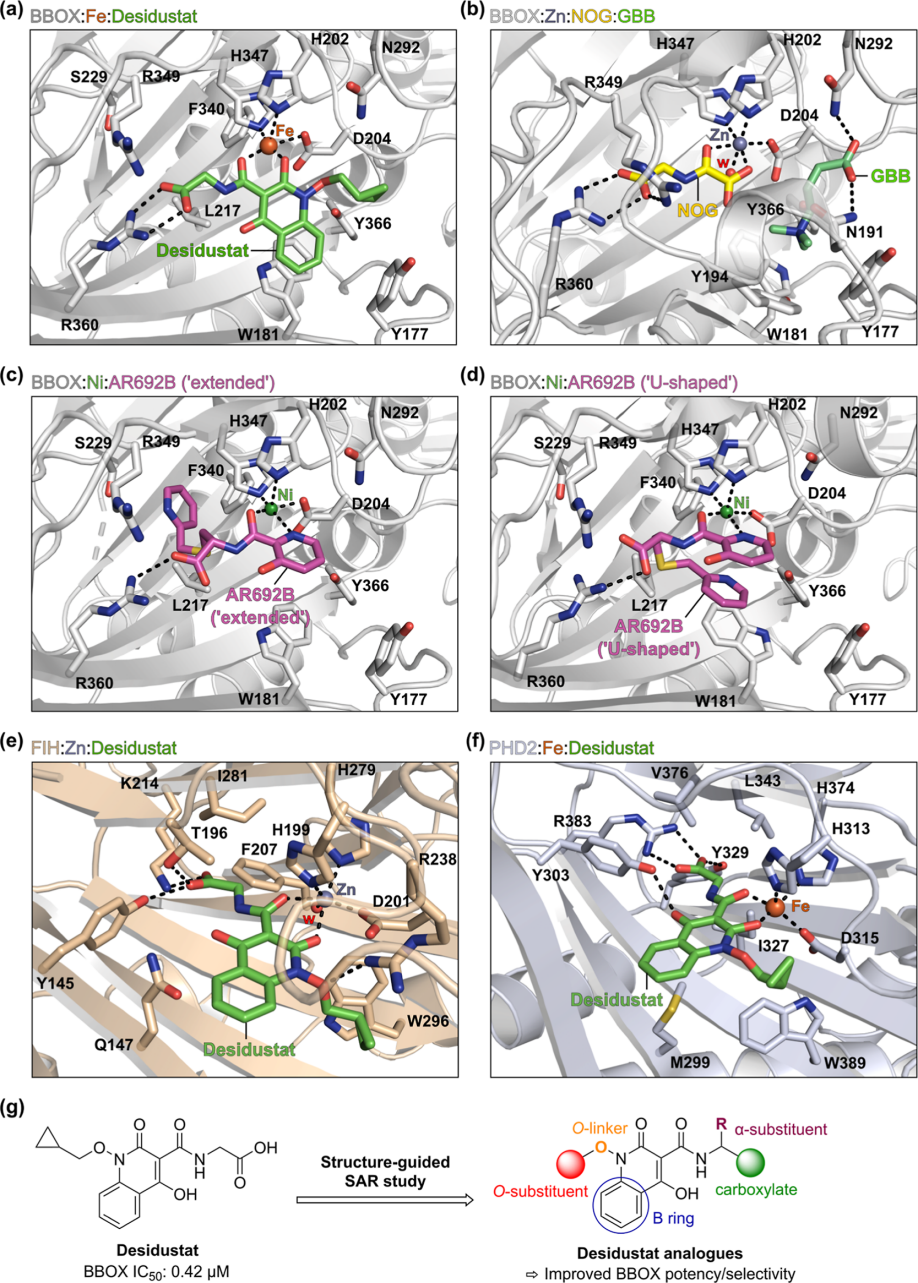
Protein–ligand docking informs on potential strategies for optimizing the Desidustat scaffold for BBOX inhibition. (a–d) Active site views from (a) the BBOX:Fe(II):Desidustat docking prediction, (b) a reported BBOX:Zn:*N*-oxalylglycine (NOG):GBB complex crystal structure (PDB ID: 3O2G;^39^ yellow: carbon-backbone of NOG; lime green: carbon-backbone of GBB), and (c,d) a reported BBOX:Ni:AR692B complex crystal structure (PDB ID: 4C8R;^36^ magenta: carbon-backbone of AR692B). AR692B is crystallographically observed to adopt two distinct binding modes within the BBOX 2OG-binding site: (i) an “extended” conformation in which its pyridine-2-ylmethyl thioether side chain binds within a hydrophobic pocket formed by BBOX active site residues Leu217, Ser229, and Phe340 (panel c); and (ii) a “U-shaped” conformation in which intramolecular π-stacking interactions are observed between its two pyridine rings (panel d). (e,f) Active site views from (e) a reported FIH:Zn:Desidustat complex crystal structure (PDB ID: 9IIF;^50^ ochre: FIH), and (f) the PHD2:Fe(II):Desidustat docking prediction (blue-gray: PHD2). Protein–ligand docking was performed using GOLD 5.1^75^ and BBOX/PHD2 receptor models derived from PDB IDs: 3O2G(39) and 4C8R(36) (for BBOX) and PDB ID: 7UMP(49) (for PHD2), as described in the Experimental Section. (g) Strategies investigated to optimize the Desidustat scaffold for BBOX inhibition. Color code: light gray: BBOX; green: carbon backbone of Desidustat; green: nickel; orange: iron; red: oxygen; blue: nitrogen; gold: sulfur. w: water.

The docking studies predicted that Desidustat will interact with the 2OG binding pocket of BBOX in a manner similar to that crystallographically observed for NOG and the 3-hydroxyhippuric acid core of AR692B (Figure 2a–d),^36,39^ including via bidentate metal coordination through the oxygen atom of its exocyclic amide substituent (positioned *trans* to Asp204) and the C-2 oxygen atom of its isoquinolinone core (positioned *trans* to His347). The glycinamide side chain of Desidustat is predicted to form electrostatic interactions with the side chain of Arg360, in an analogous manner to the glycinamide moieties of NOG and AR692B in complex with BBOX.^36,39^

The predicted binding mode of Desidustat to BBOX is thus similar to that of it in complex with FIH, as observed by crystallography (PDB ID: 9IIF; Figure 2e).^50^ Consistent with the docking prediction, Carr–Purcell–Meiboom–Gill (CPMG)-edited ^1^H NMR studies were performed using isolated recombinant *Pseudomonas* sp. AK1 BBOX (PsBBOX AK1),^61,76^ which shares ∼30% sequence similarity with human BBOX,^77,78^ indicating that Desidustat competes with 2OG for binding to PsBBOX AK1 (Figure S7) and, by implication, human BBOX.

In the predicted BBOX:Fe(II):Desidustat complex, the methylene unit of the Desidustat glycinamide side chain is orientated to face a pocket formed by the side chains of BBOX active site residues Leu217, Ser229, and Phe340. By contrast, analysis of both the reported FIH:Zn:Desidustat complex structure (Figure 2e; PDB ID: 9IIF)^50^ and the predicted binding mode of Desidustat in complex with PHD2 (Figure 2f)^50^ indicates that the Desidustat glycinamide side chain likely binds to FIH and PHD2 in close proximity to the side chains of Ile281 and Phe207 (in FIH) and Ile327 and Leu343 (in PHD2). This observation suggested that introducing substituents α to the Desidustat carboxylate group may enhance the selectivity of Desidustat for BBOX inhibition over that of FIH and PHD2. Accordingly, the thioether-linked pyridine group of AR692B is proposed to prevent efficient binding of AR692B to FIH and PHD2 (Figures S5 and S6).^36^

The introduction of substituents at the NOG C-α position is also reported to hinder binding to PHD2.^79^ By contrast, C-4-substituted 2OG derivatives and C-α-substituted NOG analogues (e.g., *N*-oxalyl-d-phenylalanine; NOFD) efficiently inhibit FIH activity;^79,80^ however, crystallographic studies have indicated that the coordination modes through which Desidustat and NOG bind to the active site metal of FIH are different.^50,79,81^ While Desidustat occupies the metal coordination sites trans to Asp201 and His279,^50^ NOG binds via the coordination sites trans to Asp201 and His199.^79,81^ Thus, the hydrogens of the methylene group of NOG face toward a vacant hydrophobic pocket formed by side chains of FIH Tyr102, Tyr145, Gln147, and Leu186, unlike that observed for Desidustat in complex with FIH.^50,79,81^

The methylenecyclopropane group of Desidustat and the phenyl ring of its quinolinone core are predicted to bind within the BBOX substrate-binding pocket (Figure 2a), with the phenyl ring positioned to form hydrophobic interactions with the side chain of Trp181, a residue that forms part of the “aromatic cage” responsible for binding the GBB trimethylammonium group (Figure 2b).^82^ The methylenecyclopropane group is predicted to bind proximal to the polar GBB-interacting residues Tyr177, Asn292, and Tyr366; thus, the computational studies imply that there is scope to optimize the interactions between Desidustat and the BBOX substrate-binding pocket, *e.g.*, through the formation of electrostatic interactions with the side chains of Tyr177, Asn292, and/or Tyr366. The targeting of specific residues within substrate binding sites has proven a successful design strategy in the development of efficient inhibitors for other 2OG oxygenases, including FIH,^53,83^ PHD2,^70^ and the fat mass and obesity associated protein (FTO).^84^

Based on the combined computational predictions, SAR studies were initiated to systematically investigate the effect of modifications to the main structural features of Desidustat on BBOX inhibition potency and selectivity with respect to inhibition of other human 2OG oxygenases, *i.e.*, (i) the glycinamide side chain, (ii) the quinolinone core, and (iii) the methylenecyclopropane group (Figure 2g).

### Effects of Substituents α to the Desidustat Carboxylate Group on BBOX Inhibition Potency

The BBOX:Fe(II):Desidustat complex docking model predicted a pocket formed by Leu217, Ser229, and Phe340 proximal to the position α to the Desidustat carboxylate (Figure 2). Thus, a set of 12 Desidustat derivatives were synthesized to investigate the effect of substituents α to the Desidustat carboxylate on BBOX inhibition potency (**8**–**19;**Table 2) with the aim of enabling stabilizing interactions with the vacant Leu217/Ser229/Phe340 pocket. The glycinamide substituents investigated included linear and branched aliphatic (i.e., **8**–**11** and **13**), carbocyclic (i.e., **12**, **14**, and **15**), and (hetero)aromatic (i.e., **16**–**19**) groups.

**Table 2:**
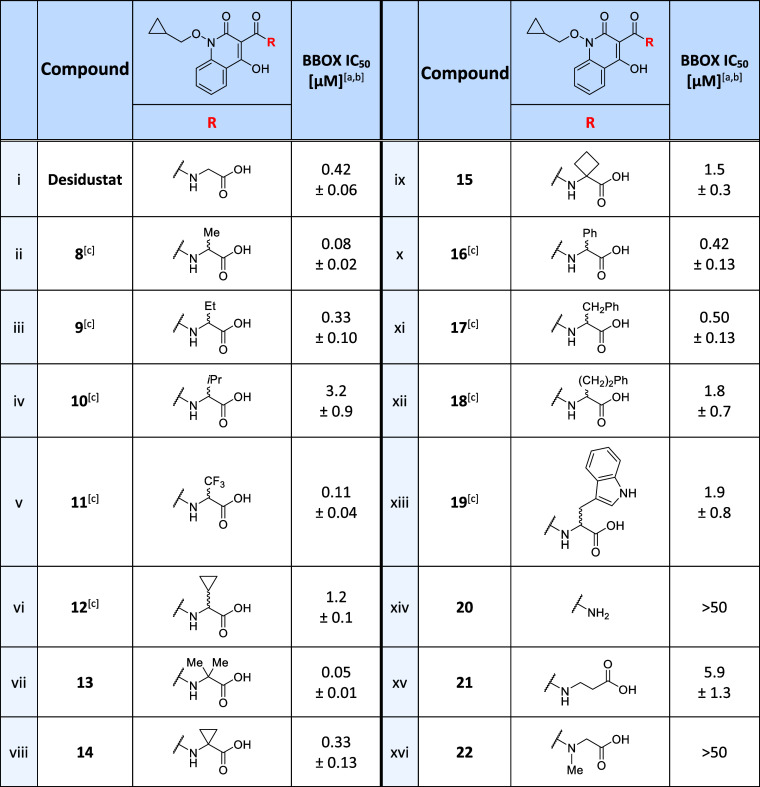
Effects of the Desidustat Glycinamide Group on BBOX Inhibition

a IC_50_ values are means ± SD of independent duplicates (each composed of technical duplicates).

b BBOX SPE-MS assays were performed using BBOX (0.05 μM), 2OG (400 μM), l-ascorbic acid (LAA; 500 μM), (NH_4_)_2_Fe(SO_4_)_2_·6H_2_O (FAS; 50 μM) and GBB (25 μM), as described in the Experimental Section.

c Chiral Desidustat analogues **8**–**12** and **16**–**19** were prepared as racemic mixtures.

**Scheme 1:**
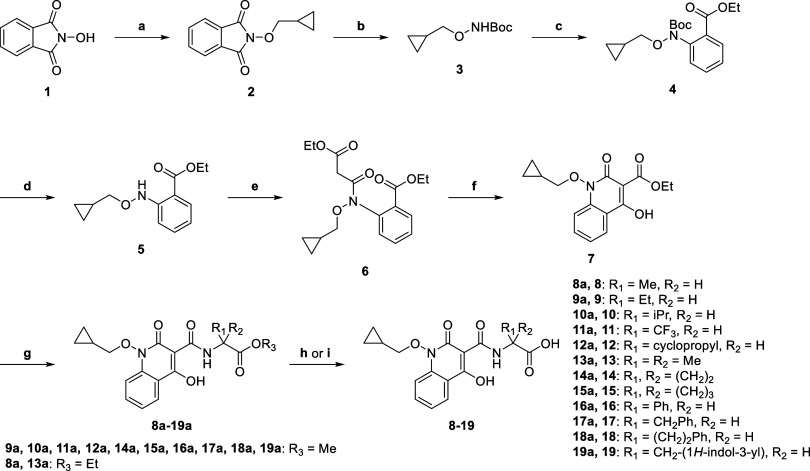
Synthesis of Desidustat Analogues **8**–**19** Reagents and conditions: (a) (bromomethyl)cyclopropane, K_2_CO_3_, DMSO, 50 °C, 99%; (b) N_2_H_4_, CH_2_Cl_2_, 0 °C to rt; then: Boc_2_O, Na_2_CO_3_, 0 °C to rt, 98%; (c) ethyl 2-iodobenzoate, CuI, glycine, K_2_CO_3_, toluene, reflux, 47%; (d) HCl/dioxane, 0 °C to rt, 99%; (e) ethyl malonyl chloride, Et_3_N, EtOAc, 0 °C to rt, 93%; (f) NaOEt, EtOH, 0 °C to rt, 94%; (g) RNH_2_·HCl, Et_3_N, dioxane, 120 °C (sealed tube), 26–96%; (h) **8a**−**10a** and **12a−19a**, LiOH, MeOH/H_2_O, 0 °C to rt, 33–65%; (i) **11a**, HCl/AcOH, reflux, 34%. Note that all chiral compounds were prepared as racemates.

The synthesis of **8**–**19** was achieved from *N*-hydroxyphthalimide **1** in eight steps, following procedures reported for the synthesis of Desidustat (Scheme 1).^40,50,73^ First, *N*-hydroxyphthalimide **1** was alkylated with (bromomethyl)cyclopropane to afford **2**. Hydrazine-mediated *N*-phthalimide deprotection of **2**, followed by *N*-Boc protection, generated *N*-Boc hydroxylamine **3**, which was then coupled with ethyl 2-iodobenzoate via a Cu(I)-catalyzed Ullmann–Goldberg reaction^85^ to give ester **4**. Sequential *N*-Boc deprotection of **4** and coupling with ethyl malonyl chloride gave malonate **6**, which was then cyclized using sodium ethoxide to afford ethyl ester **7** as a common intermediate (overall yield: 39%). Ester **7** was directly reacted with commercially sourced α-amino acids to afford glycine ester derivatives **8a−19a** in 26–96% yield. Note that α-amino acids bearing a stereogenic center were used as racemates. Finally, lithium-hydroxide-mediated ester saponification of **8a−10a** and **12a−19a** generated carboxylic acids **8**–**10** and **12**–**19**. Note that attempted saponification of trifluoromethyl intermediate **11a** using LiOH resulted in degradation; hence, **11** was synthesized from **11a** using acidic conditions.

The effects of **8**–**19** on the catalysis of isolated recombinant BBOX were determined *in vitro* using SPE-MS assays (Table 2). The results reveal that Desidustat derivative **8**, which bears a methyl group at the C-α position, inhibited human BBOX ∼5-fold more efficiently than Desidustat (IC_50_ ∼ 0.08 μM; Table 2, entry ii). This result may reflect enhanced hydrophobic interactions of **8** with the BBOX 2OG-binding site, *e.g.*, with the Leu217/Ser229/Phe340 pocket, as indicated by the predicted BBOX:Fe(II):Desidustat complex (Figure 2a). Incremental increases in the steric bulk of the C-α alkyl substituent to ethyl (**9**; IC_50_ ∼ 0.33 μM), cyclopropyl (**12**; IC_50_ ∼ 1.2 μM), and isopropyl (**10**; IC_50_ ∼ 3.2 μM) groups, correlated with decreased inhibition potency, suggesting that the α-substituent may sterically clash with residues in the BBOX active site. By contrast, the trifluoromethyl derivative **11** inhibited BBOX with similar efficiency as the alanine derivative **8** (IC_50_ ∼ 0.11 μM), i.e., ∼4-fold more efficient than Desidustat and ∼30-fold more efficient than isopropyl-containing **10**, although the trifluoromethyl substituent has an *A*-value (∼2.4 kcal/mol) comparable to that of an isopropyl group (∼2.2 kcal/mol).^86,87^ This observation suggests that factors other than the steric bulk of the C-α substituent may also determine the inhibition potency.

Notably, the most efficient BBOX inhibitor identified in this initial series was the α,α-dimethyl glycine derivative **13** (IC_50_ ∼ 0.05 μM; Table 2, entry vii), which inhibited isolated recombinant BBOX ∼10-fold more efficiently than both Desidustat and AR692B (IC_50_ values: ∼0.42 and ∼0.52 μM, respectively). Thus, **13** was >1000-fold more efficient at inhibiting BBOX than the clinically used BBOX inhibitor Mildronate (IC_50_ > 50 μM; Table 1, entry ii). Substituting the α,α-dimethyl group of **13** with cyclopropane (**14**; IC_50_ ∼ 0.33 μM) or cyclobutane (**15**; IC_50_ ∼ 1.5 μM) carbocycles reduced the potency of BBOX inhibition relative to **13**, by ∼7 and ∼30-fold, respectively, an observation which may reflect the reduced conformational flexibility of carbocyclic substituents compared to the α,α-dimethyl substituent of **13**. Note that **14**, as well as the phenylglycine (**16**; IC_50_ ∼ 0.42 μM) and phenylalanine (**17**; IC_50_ ∼ 0.50 μM) derivatives, manifested similar levels of BBOX inhibition as Desidustat (Table 2, entries viii, x, and xi). By contrast, **18** and **19**, which contain sterically larger homophenylalanine- and tryptophan-derived substituents, respectively, were ∼4-fold less efficient at inhibiting BBOX that Desidustat (IC_50_ values: ∼1.8 and ∼1.9 μM). Note that the Desidustat analogues **8**–**12** and **16**–**19** were prepared as racemic mixtures, which may influence inhibitor potency, as previously observed for AR692B and related 3-hydroxypyridine derivatives, for which the (*S*)-enantiomers typically inhibited isolated BBOX more efficiently than the corresponding (*R*)-enantiomers.^36^

The effect of introducing substituents α to the Desidustat carboxylate group on BBOX inhibition potency contrasts somewhat with that reported for 3-hydroxypyridine-based BBOX inhibitors,^36^ despite the predicted similar BBOX binding modes of AR692B and Desidustat. For instance, the (*S*)-tryptophan-derived analogue of AR692B manifested ∼10-fold more efficient BBOX inhibition than its corresponding glycine analogue, a result that contrasts with the ∼6-fold reduced inhibitory activity of **18** compared to Desidustat.^36^ Note that the (*S*)-alanine derivative of AR692B is reported to exhibit similar BBOX inhibition compared to its glycine congener, while the (*R*)-alanine derivative of AR692B was ∼10-fold less potent.^36^ By contrast, the introduction of a methyl substituent α to the Desidustat carboxylate (*i.e.*, as in **8**) enhanced the BBOX inhibition potency by ∼5-fold.

The observed differences in the SAR of AR692B and Desidustat derivatives could reflect the different assay conditions employed and/or subtle conformational differences of AR692B and Desidustat derivatives in complex with BBOX, in particular, since the pyridine-2-ylmethyl thioether side chain of AR692B is observed to adopt two distinct conformations in the reported BBOX:Ni:AR692B complex structure (Figure 2c,d).^36^ The SAR data presented here on Desidustat derivatives indicate that further modification of the AR692B thioether-linked 2-pyridyl side chain may afford 3-hydroxypyridine-derived BBOX inhibitors with improved inhibition activity.

Derivatives **20**–**22** were then prepared to investigate the effects of: (i) removing the glycinamide side chain (*i.e.*, **20**), (ii) replacing the glycinamide unit with a three carbon β-alanine-derived side chain (*i.e.*, **21**), and (iii) the methylation of the glycinamide *N* atom (*i.e.*, **22**) on BBOX inhibition (Scheme S1). While Desidustat derivatives **8**–**19** were relatively efficient inhibitors of BBOX (IC_50_ < 5 μM; Table 2, entries ii–xiii), the primary amide **20** manifested no effect on BBOX catalysis in the tested concentration range (IC_50_ > 50 μM; Table 2, entry xiv), implying that the Desidustat glycinamide side chain is important for efficient BBOX inhibition, likely because its carboxylate group is positioned to interact with the guanidinium side chain of Arg360 (Figure 2a).

The β-alanine derivative **21** inhibited BBOX ∼14-fold less efficiently than Desidustat (IC_50_ ∼ 5.9 μM; Table 2, entry xv), while the sarcosine derivative **22** did not inhibit within the tested concentration range (IC_50_ > 50 μM; Table 2, entry xvi). In the modeled BBOX:Fe(II):Desidustat complex, the glycinamide NH group of Desidustat is predicted to form an internal hydrogen bond with the C-4 oxygen atom of its quinolinone core (Figure 2a). Analogous internal hydrogen bonding interactions are observed crystallographically for Desidustat in complex with FIH (Figure 2e)^50^ and for quinolinone- and, structurally related, 3-hydroxypyridine-based inhibitors (including AR692B) in complex with BBOX, PHD2, FIH, and FTO.^36,51,84,88,89^ This interaction is proposed to restrict the conformational flexibility of the bidentate metal-binding group of the inhibitor and thus to enhance metal binding and may also aid in cell membrane penetration.

### Effects of the Desidustat Carboxylate Group on BBOX Inhibition Potency

The observation that Desidustat derivative **20**, which lacks the glycine unit of Desidustat, does not efficiently inhibit BBOX (IC_50_ > 50 μM; Table 2, entry xiv) implies that the predicted salt bridge interaction of the glycine carboxylate with the guanidinium group of Arg360 is important for BBOX inhibition (Figure 2a). However, the carboxylate group of AR692B in the reported BBOX:Ni:AR692B complex structure is positioned to form only a single hydrogen bond interaction with Arg360 (Figure 2c,d).^36^ This observation indicates that it may be possible to replace the Desidustat carboxylate with alternative hydrogen bond acceptors, while maintaining efficient BBOX inhibition.

Since the Desidustat carboxylate is predicted/observed crystallographically to form ionic interactions to Arg or Lys residues within the active sites of, *e.g.*, PHD and FIH (Figure 2e,f),^50^ we considered it possible that isosteric replacement of the carboxylate group may enhance selectivity for BBOX inhibition over that of other 2OG oxygenases. Note that potent small-molecule inhibitors of other 2OG oxygenases,^90−94^ including the clinically used PHD inhibitor Molidustat,^95,96^ have been developed which interact with protein residues binding the 2OG C-5 carboxylate via functional groups other than carboxylic acids, *e.g.*, a triazole in the case of Molidustat.^95,96^ Replacement of the Desidustat carboxylate group may improve physicochemical properties for future cell-based and *in vivo* applications, including cell permeability.^97^ Thus, a further set of Desidustat derivatives (**23–32**) was synthesized in which the carboxylate moiety was replaced with different polar functional groups, *e.g.*, ester (**23** and **24**), amide (**25** and **26**), nitrile (**27**), alcohol (**28** and **29**), and heterocyclic (**30**–**32**) groups (Schemes S2–S4).

By contrast with truncated Desidustat derivative **20** (Table 2, entry xiv), all of **23**–**32** inhibited BBOX under the SPE-assay conditions employed, with IC_50_ values ranging from ∼0.55 to ∼27 μM (Table 3). Notably, the ethyl ester **24** efficiently inhibited BBOX, with an IC_50_ value in the range as that for Desidustat (IC_50_ ∼ 0.55 μM; Table 3, entry iii). Hydrolysis of **24** under the assay conditions to form Desidustat could account, at least in part, for the high potency of **24**. However, since methyl ester **23** should be more susceptible toward saponification than **24**, but inhibits BBOX ∼7-fold less efficiently than **24** (IC_50_ ∼ 3.7 μM; Table 3, entry ii), the combined results indicate that replacement of the Desidustat carboxylate with an ethyl ester group is a viable strategy to maintain efficient BBOX inhibition. It appears possible that the Desidustat ester derivatives adopt a conformation in complex with BBOX in which their ester carboxylate faces away from the guanidinium group of Arg360, as crystallographically observed for the carboxylate group of AR692B in the reported BBOX:Ni:AR692B complex structure (Figure 2c,d).^36^ Note that inhibitors of PHD2^98^ and the lysine C-5 hydroxylase JMJD6^99^ have also been reported that contain ester groups.

**Table 3:**
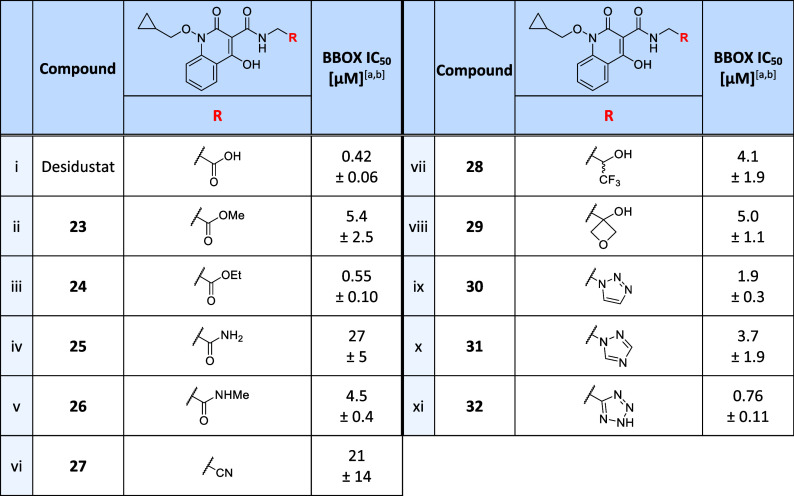
Effects of Desidustat Carboxylate Isosteres on BBOX Inhibition

a IC_50_ values are means ± SD of independent duplicates (each composed of technical duplicates).

b BBOX SPE-MS assays were performed using BBOX (0.05 μM), 2OG (400 μM), LAA (500 μM), FAS (50 μM), and GBB (25 μM), as described in the Experimental Section.

The tetrazole derivative **32** inhibited BBOX with an IC_50_ value ∼2-fold greater than that determined for Desidustat (IC_50_ ∼ 0.76 μM; Table 3, entry xi), while moderate levels of BBOX inhibition were observed for *N*-methyl amide **26** (IC_50_ ∼ 4.5 μM), trifluoroethanol analogue **28** (IC_50_ ∼ 4.1 μM), cyclic ether **29** (IC_50_ ∼ 5.0 μM), and the triazole derivatives **30** (IC_50_ ∼ 1.9 μM) and **31** (IC_50_ ∼ 3.7 μM). Overall, the inhibition results indicate that the Desidustat carboxylate group is useful but is not essential for potent BBOX inhibition, an observation that may be of interest for the design of selective Desidustat-based small-molecule BBOX inhibitors for cellular studies.

### Effects of the Desidustat *N*-Alkoxy Substituent on BBOX Inhibition Potency

**Figure 3:**
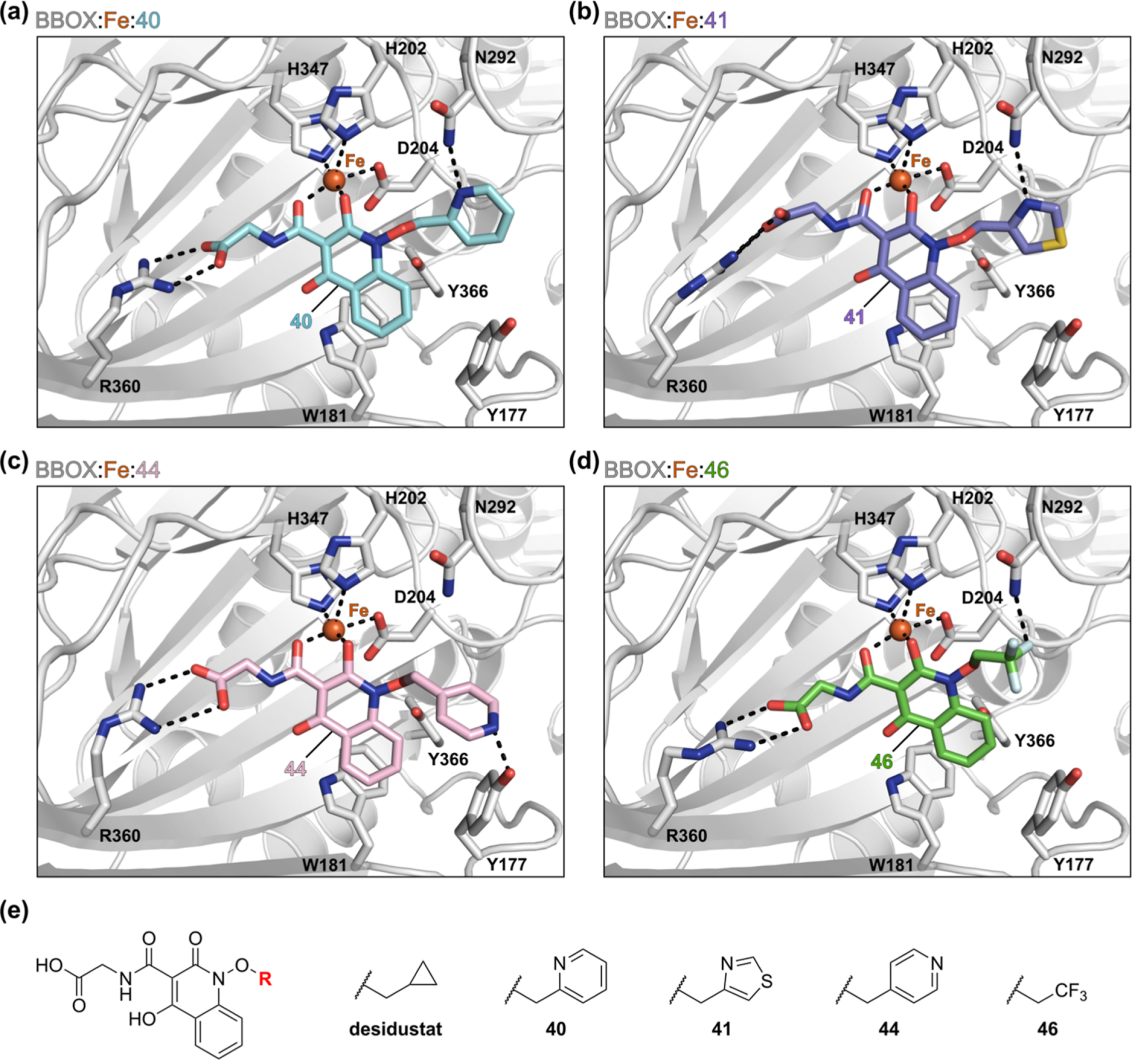
Computational studies predicted that Desidustat derivatives may form hydrogen bonds with the side chains of GBB-binding residues in the BBOX active site. (a–d) Active site views from docking predictions: (a) BBOX:Fe:**40** (cyan: carbon-backbone of **40**), (b) BBOX:Fe:**41** (violet: carbon-backbone of **41**), (c) BBOX:Fe:**44** (light pink: carbon-backbone of **44**), and (d) BBOX:Fe:**46** (green: carbon-backbone of **46**). (e) Structures of Desidustat and selected Desidustat analogues predicted to form hydrogen bonds with the side chains of residues within the BBOX substrate-binding pocket. Color code: light gray: BBOX; orange: iron; red: oxygen; blue: nitrogen; gold: sulfur. w: water. Protein–ligand docking was performed using GOLD 5.1^75^ and a BBOX receptor model derived from reported BBOX:Zn:*N*-oxalylglycine (NOG):GBB and BBOX:Ni:AR692B complex crystal structures (PDB IDs: 3O2G(39) and 4C8R(36)), as described in the Experimental Section.

The BBOX:Fe(II):Desidustat complex docking model predicted that the Desidustat methylenecyclopropane group would project into the BBOX substrate-binding pocket (Figure 2a). Hence, we employed protein–ligand docking (using GOLD 5.1^75^) to identify groups which may interact favorably with the BBOX GBB-binding site, *e.g.*, via formation of hydrogen bonding and/or enhanced hydrophobic interactions, to improve the inhibition potency of Desidustat. The computational studies predicted that three different classes of substituents replacing the Desidustat methylenecyclopropane group may enhance the binding affinity of Desidustat to BBOX, *i.e.*, (i) ortho-substituted heteroaromatic groups, which were predicted to form hydrogen bonds with the Asn292 side chain, mimicking the crystallographically observed interaction between the Asn292 side chain and the GBB carboxylate (*i.e.*, **40**–**43**; Figure 3a,b); (ii) heterocyclic groups, which were predicted to form hydrogen bonds with the Tyr177 phenolic hydroxyl group (*i.e.*, para-substituted pyridine **44** and tetrahydropyran **45**; Figure 3c); and (iii) trifluoromethyl derivative **46** which was predicted to hydrogen bond with the side chain of Asn292 (Figure 3d).

**Scheme 2:**
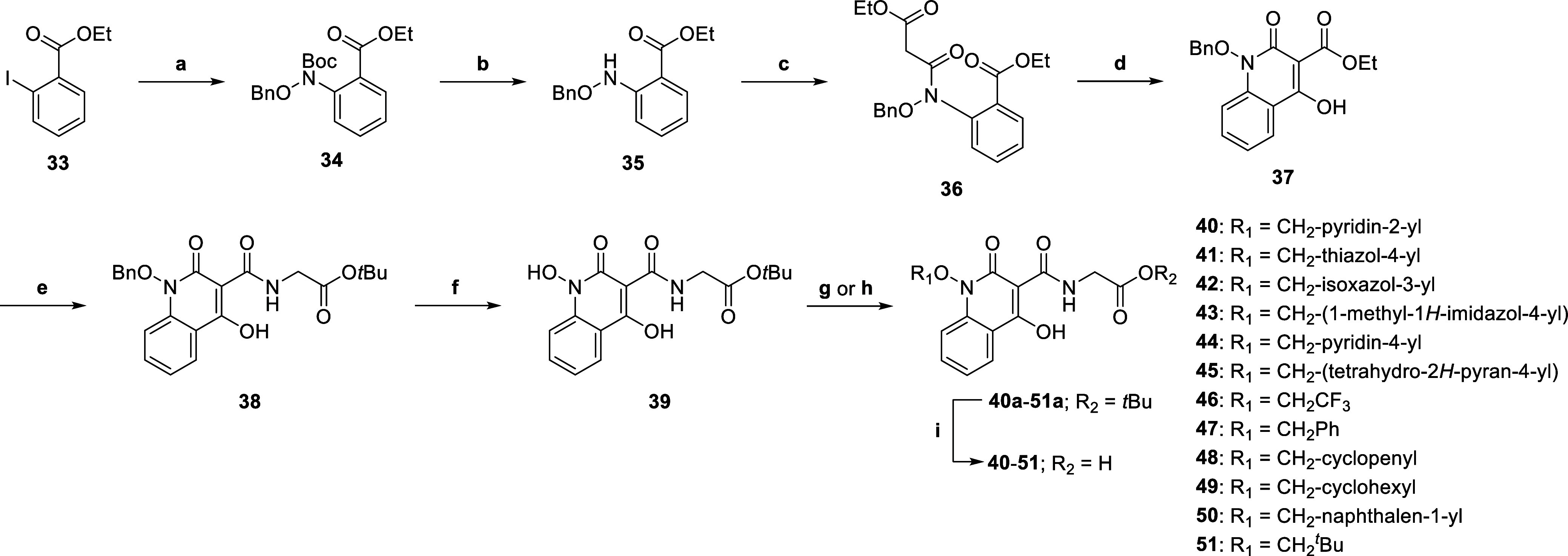
Synthesis of Desidustat Derivatives **40**–**51** with Altered *N*-Alkoxy Substituents Reagents and conditions: (a) *tert*-butyl *N*-(benzyloxy)carbamate, CuI, glycine, K_2_CO_3_, toluene, reflux, 56%; (b) HCl/dioxane, 0 °C to rt, 66%; (c) ethyl malonyl chloride, Et_3_N, EtOAc, 0 °C to rt, 74%; (d) NaOEt, EtOH, 0 °C to rt, 72%; (e) glycine *tert*-butyl ester hydrochloride, Et_3_N, dioxane, 120 °C (sealed tube), 82%; (f) H_2_, Pd/C, MeOH, rt, 34%; (g) R_1_OH, di-2-methoxyethyl azodicarboxylate (DMEAD),^100^ PPh_3_, THF, rt, 33–98%; (h) 2,2,2-trifluoroethan-1-ol, K_2_CO_3_, DMSO, 50 °C, 68%; (i) TFA, CH_2_Cl_2_, rt, 23–98%.

The Desidustat derivatives **40**–**46** were synthesized to probe the three strategies mentioned above to target binding in the BBOX substrate binding pocket. Additionally, phenyl, cyclopentyl, cyclohexyl, naphthalene, and *tert*-butyl derivatives **47**–**51**, which are unable to form hydrogen bonding interactions, were synthesized as (likely) negative controls. A synthetic route to **40**–**51** was developed that enabled the late-stage incorporation of substituents onto the hydroxylamine group of the Desidustat quinolinone core (Scheme 2). *O*-Benzyl protected 1,2-dihydroxyquinolin-4-one **38** was synthesized via an analogous route to esters **8a−19a** (Scheme 1), employing commercially sourced *tert*-butyl (benzyloxy)carbamate and phenyl iodide **33** as coupling partners in a Goldberg–Ullmann reaction (Scheme 2).^85^ Hydroxylamine **39** was obtained in five steps (overall yield: ∼5%) and was used as a common intermediate for subsequent alkylations. The hydroxylamine *O*-atom of **39** was efficiently alkylated under Mitsunobu conditions to generate **40a**−**45a** and **47a−51a**. Trifluoromethyl derivative **46a** was prepared via K_2_CO_3_-mediated alkylation of **39** with 1,1,1-trifluoro-2-iodoethane, because the attempted Mitsunobu reaction between **39** and 2,2,2-trifluoroethan-1-ol was unsuccessful. Subsequent acid-mediated *tert*-butyl ester cleavage of **40a−51a** afforded carboxylic acids **40**–**51**.

The ability of Desidustat derivatives **40**–**51** to inhibit isolated recombinant BBOX was investigated by SPE-MS (Table 4). The results reveal that derivatives **40**–**43** bearing *ortho*-substituted heteroaromatic rings which were predicted to form hydrogen bonds with the side chain of Asn292 (Figure 3a,b), *i.e.*, to mimic the BBOX binding mode of the GBB carboxylate,^39^ inhibited BBOX with high potency (Table 4, entries ii-v). Notably, the pyridine **40** and thiazole **41** derivatives inhibited BBOX ∼7- and ∼20-fold more efficiently than Desidustat, manifesting IC_50_ values close to the lower intrinsic limit of the SPE-MS assay (IC_50_ values: ∼0.06 and ∼0.02 μM, respectively). **40** and **41** were also ∼7- and ∼20-fold more efficient at inhibiting BBOX than AR692B and >800- and >2500-fold more potent than Mildronate. By contrast to **40** and **41**, the corresponding phenyl and cyclopentyl derivatives **47** and **48** inhibited BBOX ∼2-fold less efficiently than Desidustat (IC_50_ values: ∼0.79 and ∼0.92 μM), consistent with the prediction that the increase in inhibition potency observed for **40**–**43** is due to the formation of hydrogen bonds with the BBOX substrate-binding site (Figure 3a,b).

**Table 4:**
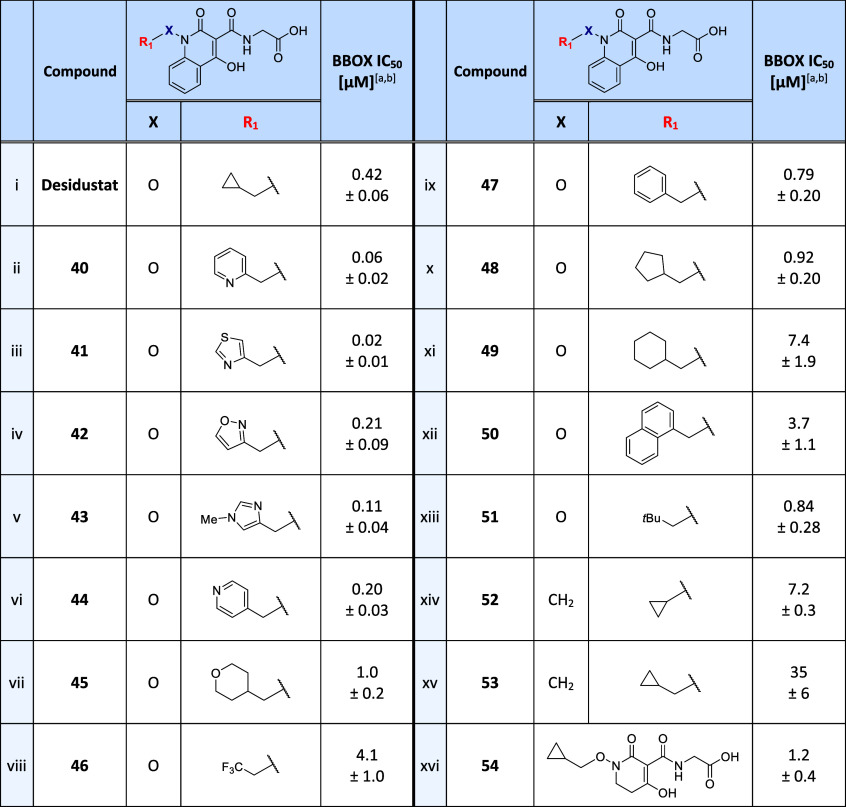
Effects of the Desidustat *N*-Alkoxy Substituent and Quinolinone Core on BBOX Inhibition

a IC_50_ values are means ± SD of independent duplicates (each composed of technical duplicates).

b BBOX SPE-MS assays were performed using BBOX (0.05 μM), 2OG (400 μM), LAA (500 μM), FAS (50 μM), and GBB (25 μM), as described in the Experimental Section.

The pyridine derivative **44** was also more potent in inhibiting isolated BBOX than both Desidustat and the phenyl derivative **47** (IC_50_ ∼ 0.20 μM; Table 4, entry vi), potentially reflecting the formation of the computationally predicted hydrogen bond between the pyridine ring *N* atom of **44** with the phenolic *OH* group of Tyr177 (Figure 3c). By contrast, tetrahydropyran derivative **45**, which was also predicted to interact with Tyr177, inhibited BBOX ∼2-fold less efficiently than Desidustat (IC_50_ ∼ 1.0 μM). Note, however, that **45** inhibited BBOX ∼7-fold more efficiently than cyclohexane derivative **49** (IC_50_ ∼ 7.4 μM), indicating that steric effects may be responsible for the observed reduction in potency relative to Desidustat. Trifluoromethyl derivative **46**, which was predicted to interact with the side chain of Asn204 (Figure 3d), and naphthalene **50** also manifested reduced levels of inhibition of BBOX compared with Desidustat (IC_50_s: ∼4.1 and ∼3.7 μM).

Desidustat derivatives **52**–**54** were synthesized to probe the importance of the *O*-atom that links the Desidustat quinolinone core and its methylenecyclopropane side chain (Scheme S5) and of the quinolinone core phenyl ring (Scheme S6), on BBOX inhibition. SPE-MS assays showed that Desidustat derivatives **52** and **53** inhibited BBOX substantially less efficiently than Desidustat (IC_50_ values: ∼7.2 and ∼35 μM), a result that indicates that the hydroxylamine linker of Desidustat is important for efficient inhibition. The phenyl ring of Desidustat’s quinolinone core is also important for BBOX inhibition, since its removal (as in **54**) led to a ∼3-fold decrease in potency compared with Desidustat, potentially reflecting the loss of favorable hydrophobic interactions with the side chain of Trp181 (Figure 2a).

### Design of Optimized BBOX Inhibitors

**Scheme 3:**
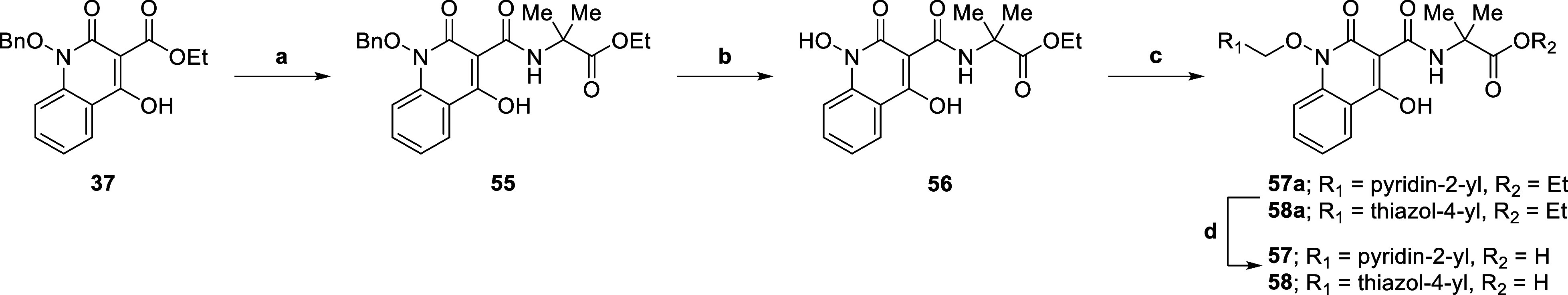
Synthesis of Desidustat Analogues **57** and **58** Reagents and conditions: (a) glycine ethyl ester hydrochloride, Et_3_N, dioxane, 120 °C (sealed tube), 82%; (b) H_2_, Pd/C, MeOH, rt, 34%; (c) ROH, DMEAD,^100^ PPh_3_, THF, rt, 40–42%; (d) LiOH, MeOH/H_2_O, 60 °C, 50–57%.

The combined structure activity relationship studies described above indicate that the potency of Desidustat for BBOX inhibition can be enhanced by structural modification of both its glycinamide and methylenecyclopropane side chains (Tables 2 and 4). For instance, derivative **13**, which contains a α,α-dimethyl-substituted glycinamide side chain, inhibited BBOX ∼9-fold more efficiently than Desidustat, while derivatives **40** and **41**, which contain pyridin-2-yl and thiazol-4-yl groups in place of the Desidustat cyclopropyl group, were ∼7- and ∼20-fold more potent than Desidustat. Consequently, Desidustat derivatives **57** and **58** were synthesized that combine the α,α-dimethyl glycine side chain of **13** with the pyridin-2-yl and thiazol-4-yl groups of **40** and **41**, to obtain highly optimized inhibitors for functional assignment studies. Acids **57** and **58** were prepared in four steps from **37** via common intermediate **56**, according to the synthetic route outlined in Scheme 3. Ethyl ester analogues of **57** and **58**, i.e., **57a** and **58a**, were also prepared, following the observation that ethyl ester **24** manifested BBOX inhibitory activity similar to that of Desidustat.

Derivatives **57** and **58** inhibited isolated BBOX with similar potency as their respective glycine analogues **40** and **41** (IC_50_ values: ∼0.04 and ∼0.02 μM; Table 5 entries xii and xiii) and were substantially more efficient at inhibiting BBOX than Desidustat and AR692B (IC_50_ values: ∼0.42 and ∼0.52 μM, respectively). Thus, they were >1200-fold more efficient at inhibiting BBOX than the clinically used BBOX inhibitor Mildronate (IC_50_ > 50 μM; Table 1, entry ii). It should be noted, however, that the BBOX IC_50_ values determined for **57** and **58** were close to the lower intrinsic limit of the SPE-MS assay (BBOX concentration: 0.05 μM); thus, as with **40** and **41**, it is possible that **57** and **58** are more efficient BBOX inhibitors than those indicated by their IC_50_ values.

**Table 5:**
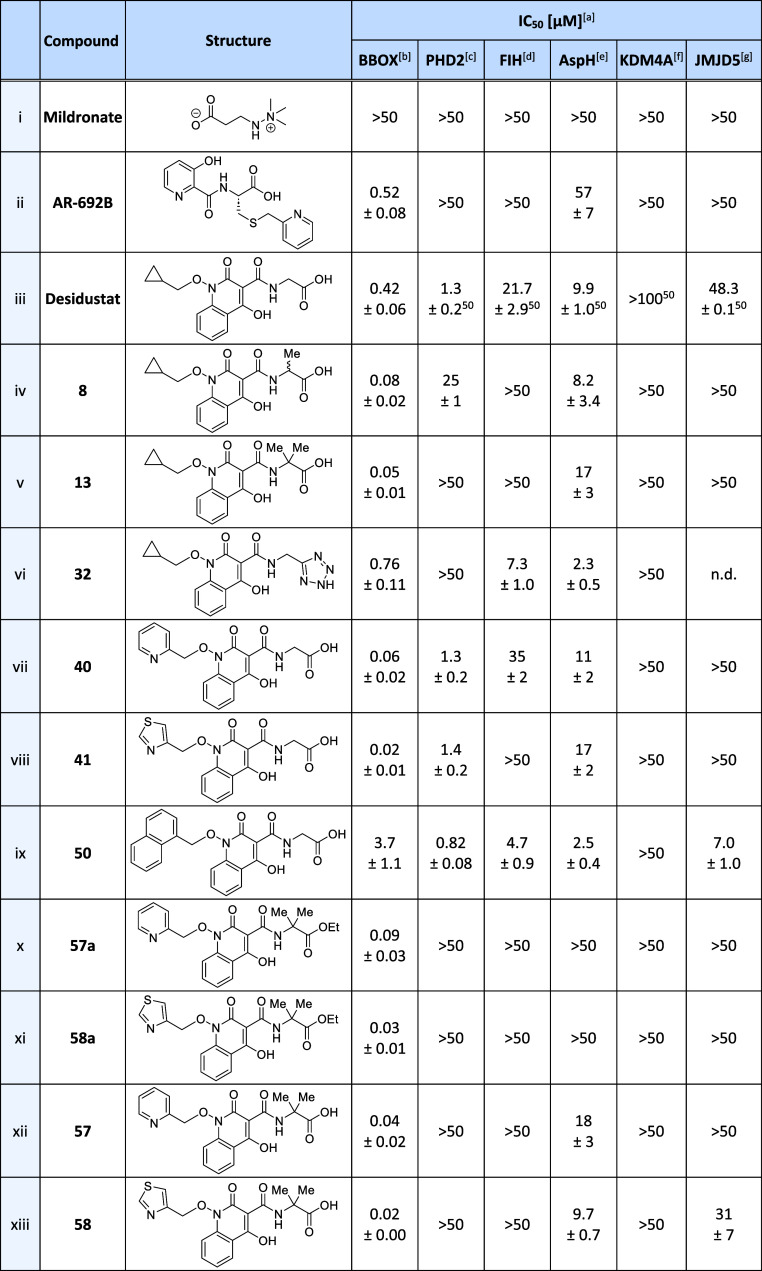
Inhibition of Human 2OG Oxygenases by Selected Desidustat Derivatives

a IC_50_ values are means ± SD of independent duplicates (each composed of technical duplicates).

b Using 0.05 μM BBOX, 400 μM 2OG, and 25 μM GBB.

c Using 0.15 μM PHD2_181–426_, 10 μM 2OG, and 5.0 μM HIF-1α CODD_556–574_.^71^

d Using 0.15 μM FIH, 10 μM 2OG, and 5.0 μM HIF-1α C-TAD_788–822_.^71^

e Using 0.05 μM His_6_-AspH_315–758_, 3 μM 2OG, and 1.0 μM hFX-CP_101–119_.^68^

f Using 0.15 μM KDM4A, 10 μM 2OG, and 10.0 μM of a H3_1–15_K9me3 variant.^72^

g Using 0.15 μM JMJD5, 2 μM 2OG, and 2.0 μM RSP6_128–148_.^67^ Inhibition assays were performed using SPE-MS, as described in the Experimental Section. n.d.: not determined.

The ethyl ester derivatives **57a** and **58a** manifested similar levels of BBOX inhibition (within experimental error) as the carboxylic acids **40** and **41** (IC_50_ values: ∼0.09 and ∼0.03 μM; Table 5, entries x and xi). Thus, **57a** and **58a** manifested ∼5- and ∼15-fold more efficient BBOX inhibition than both Desidustat ethyl ester **24** and Desidustat itself. It should be noted, however, that the ethyl ester groups of **57a** and **58a** are likely susceptible to cleavage by esterases in cells, which may limit their suitability for cell-based studies.

### 2OG Oxygenase Selectivity Studies

Reported SPE-MS assays^67,68,71,72^ were employed to investigate whether the structural modifications made to the Desidustat glycinamide and methylenecyclopropane groups influenced the 2OG oxygenase selectivity profile of the Desidustat scaffold (Tables 5 and S1). The selectivity studies revealed that the introduction of substituents onto the glycinamide side chain of Desidustat, at least for the compounds tested, substantially reduced the level of PHD inhibition relative to Desidustat. For instance, the alanine derivative **8** inhibited PHD2 ∼10-fold less efficiently than Desidustat (IC_50_ ∼ 24.6 μM; Table 5, entry iv), while the α,α-dimethyl substituted analogue **13** did not inhibit PHD2 in the tested concentration range (IC_50_ > 50 μM; Table 5, entry v). This effect is similar to that reported for derivatives of NOG, for which the introduction of groups adjacent to its glycinamide carboxylate largely abrogated PHD2 inhibition, while maintaining or improving FIH inhibition.^79^

In general, Desidustat analogues that contained substituents at the C-α position of the glycinamide side chain manifested reduced FIH inhibition relative to Desidustat, while a negligible change in AspH inhibitory activity was observed (Table 5). For instance, **8** and **13** inhibited FIH > 2-fold less efficiently than Desidustat, while both **8** and **13** inhibited AspH with approximately similar efficiency to that observed for Desidustat (IC_50_ values: ∼8.2 μM and ∼17 μM; Table 5, entries iv and v). The observed reduction in potency for FIH inhibition contrasts with that observed for C-α-substituted NOG analogues; for instance, the phenylalanine derivative NOFD is reported to inhibit isolated FIH with similar efficacy as NOG (IC_50_ values: ∼0.36 μM and ∼0.24 μM, respectively).^53,79^ Nonetheless, this apparent discrepancy likely reflects the different FIH binding modes of Desidustat and NOG.^50,79^ NOG binds FIH with its pro-*R* methylene H atom orientated toward a hydrophobic pocket formed by the side chains of Tyr102, Tyr145, GLn147, and Leu186,^79^ while in the reported FIH:Zn:Desidustat complex structure, the C-α atom of Desidustat’s glycinamide unit is positioned proximal to the side chains of Ile281 and Phe207.^50^ Note that both **8** and **13** did not inhibit KDM4A and JMJD5 (IC_50_ > 50 μM; Table 5).

The tetrazole-containing Desidustat derivative **32** did not inhibit the activities of PHD2 and KDM4A within the tested concentration range (IC_50_ > 50 μM; Table 5, entry vi). By contrast, **32** inhibited AspH and FIH ∼4- and ∼3-fold more efficiently, respectively, than Desidustat (AspH IC_50_ ∼2.3 μM; FIH IC_50_ ∼7.3 μM). Thus, tetrazole **32** may represent a potential starting point for the development of efficient Desidustat-based AspH and/or FIH inhibitors. Note, tetrazole-containing derivatives of 2OG have been developed as KDM4 inhibitors.^94,101^

The replacement of the Desidustat methylenecyclopropane side chain with heteroaromatic groups, as in **40** and **41**, had little effect on PHD2 inhibition potency (Tables 5, entries vii and viii; S1). Desidustat derivatives **40** and **41** were therefore more selective for BBOX versus PHD2 inhibition than Desidustat (∼7- and ∼23-fold more selective), although this effect was largely driven by their increased BBOX inhibitory activity. The selectivity of **40** and **41** for the inhibition of BBOX over FIH and AspH inhibition was also increased relative to Desidustat. In addition, **40** and **41** did not inhibit KDM4A in the tested concentration range (IC_50_ > 50 μM). By contrast with the heterocyclic analogues evaluated, naphthalene derivative **50** was ∼14-fold more selective for inhibiting PHD2 over BBOX than Desidustat (Table 5, entry ix), an observation potentially of interest for the development of Desidustat-based PHD inhibitors with improved PHD selectivity over BBOX. Note, however, that **50** also exhibited increased FIH (∼5-fold more potent), AspH (∼4-fold) and JMJD5 (∼7-fold) inhibition compared with Desidustat.

The optimized BBOX inhibitors **57** and **58** manifested excellent selectivity for inhibiting BBOX over other 2OG oxygenases tested (Table 5, entries xii and xiii). For instance, the thiazole derivative **58** inhibited BBOX highly selectively over inhibition of PHD2, FIH, and KDM4A (>2500-fold selectivity), AspH (∼500-fold selectivity) and JMJD5 (∼1500-fold selectivity). Interestingly, ethyl ester derivatives **57a** and **58a** manifested no inhibition of any of the other human 2OG oxygenases tested (Table 5, entries x and xi).

### Crystallographic Studies

To investigate the 2OG oxygenase binding mode of Desidustat derivative **58**, crystallographic studies were performed. Our attempts to crystallize **58** in complex with isolated recombinant BBOX and PsBBOX AK1 were unsuccessful. **58** was instead cocrystallized with isolated recombinant FIH, using Zn(II) as a catalytically inactive surrogate for Fe(II). FIH has been used as a model human 2OG oxygenase to investigate the oxygenase binding modes of 2OG-competitive inhibitors (*e.g.*, Desidustat),^50,51^ because (i) FIH can be recombinantly expressed in*Escherichia coli*and be isolated in high yield and purity; (ii) isolated FIH is readily crystallized under robust sitting-drop vapor diffusion conditions; and (iii) the crystallographically observed FIH binding modes of 2OG-competing inhibitors are similar to those observed in complex with other human 2OG oxygenases,^51,96^ reflecting the structural similarities between the active sites of human 2OG oxygenases, *e.g.*, in Fe(II)- and 2OG-binding.^102^

**Figure 4:**
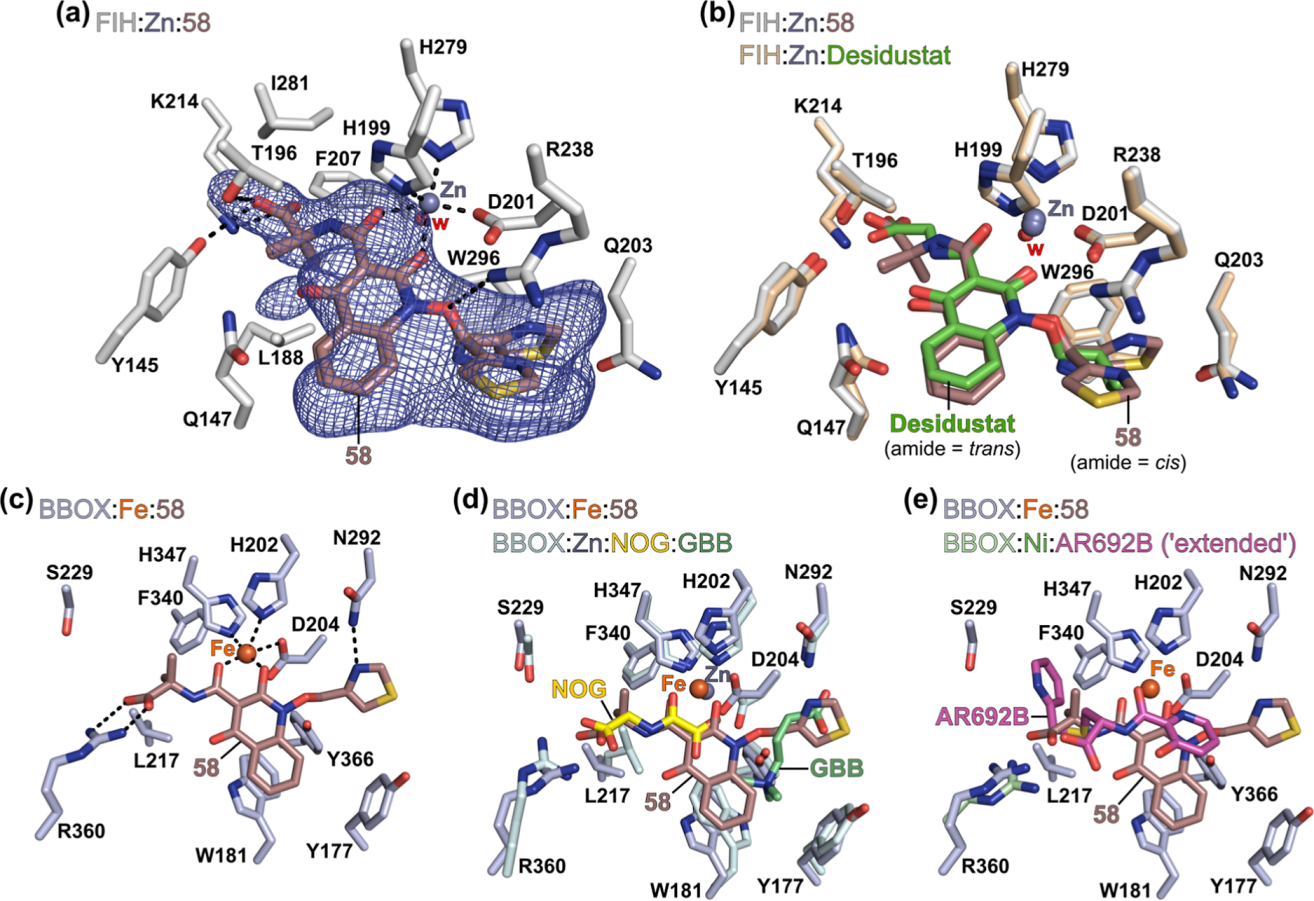
Crystallographic studies reveal Desidustat derivative **58** binds to the FIH active site in a similar manner as Desidustat. (a) Active site view from the FIH:Zn:**58** (PDB ID: 9JTX; light gray: FIH) complex structure showing the OMIT electron density map (mFo-DFc) contoured to 4.0 σ around **58**. (b) Superimposition of active site views from the FIH:Zn:**58** complex structure (light gray: FIH) with a reported FIH:Zn:Desidustat complex structure (PDB ID: 9IIF;^50^ ochre: FIH; green: carbon-backbone of Desidustat). (c) Active site view from the predicted BBOX:Fe(II):**58** complex structure (light blue: BBOX). (d,e) Superimposition of active site views from the predicted BBOX:Fe(II):**58** complex structure (light blue: BBOX) and reported (d) BBOX:Zn:NOG:GBB (PDB ID: 3O2G;^39^ light cyan: BBOX; yellow: carbon-backbone of NOG; lime green: carbon-backbone of GBB), and (e) BBOX:Ni:AR692B (PDB ID: 4C8R;^36^ light green: BBOX; magenta: carbon-backbone of AR692B) complex crystal structures. In (e), AR692B is shown in its “extended” BBOX binding mode. Protein–ligand docking was performed using GOLD 5.1^75^ and a BBOX receptor model derived from reported BBOX:Zn:*N*-oxalylglycine (NOG):GBB and BBOX:Ni:AR692B complex crystal structures (PDB IDs: 3O2G(39) and 4C8R(36)), as described in the Experimental Section. Note, the amide group of **58** was permitted to invert between *cis*- and *trans*-conformations during the docking simulation. Color code: brown: carbon backbone of **58**; gray: zinc; orange: iron; red: oxygen; blue: nitrogen; green: chlorine; gold: sulfur. w: water.

Crystals of FIH in complex with **58** and Zn(II) were obtained by cocrystallization and the structure was solved by molecular replacement (MR) (PDB ID:9JTX; space group: *P*4_1_2_1_2, resolution: 2.08 Å). Analysis of the FIH:Zn:**58** complex structure reveals that **58** binds to the FIH active site in a similar manner to that observed for Desidustat (Figure 4a,b).^50^ Thus, **58** is observed to coordinate the active site Zn(II) (substituting for Fe(II)) of FIH through the C-2 *O*-atom of its quinoline core and its amide *O*-atom. The hydroxylamine *O*-atom of **58** is positioned to interact with the side chain of Arg238, while its carboxylate group is oriented to interact with the Tyr145, Thr196, and Lys214 side chains. The thiazol-4-yl side chain of **58** is observed to extend into the FIH substrate binding pocket and likely has multiple conformations in complex with FIH; two major conformations were identified during structural refinement (Figure 4a,b).

The glycinamide group of **58** occupies an unusual *cis*-conformation in the FIH:Zn:**58** complex structure, potentially to avoid steric interactions between the α,α-dimethyl glycinamide group of **58** and FIH residues Phe207 and Ile281, the side chains of which are proximal to the Desidustat glycinamide side chain in the FIH:Zn:Desidustat complex structure.^50^ As a result, the amide NH of **58** is unable to engage in intramolecular hydrogen bonding with the C-4 *O*-atom of its quinolinone core, as observed for Desidustat in complex with FIH (Figure 4). By contrast, protein–ligand docking studies indicate that **58** may bind BBOX with its amide group positioned in the *trans* geometry (Figure 4c). **58** is predicted to bind at the BBOX active site and compete for binding with both 2OG and GBB (Figure 4d), *i.e.*, via a related binding mode to that of AR692B in complex with BBOX (Figure 4e).^36^ In the predicted BBOX:Fe:**58** complex model, the α,α-dimethyl glycinamide group of **58** is positioned to form hydrophobic interactions with the side chains of Leu217 and Phe340, while the phenyl ring of its quinolinone core is positioned to form a hydrophobic contact with the Trp181 side chain, a residue crystallographically observed to be involved in binding of the GBB trimethylammonium moiety (Figure 4d).^82^ The thiazole *N*-atom of **58** is predicted to form a hydrogen bond with the side chain of Asn292 (Figure 4c), an interaction that is analogous to the hydrogen bond observed between Asn292 and the GBB carboxylate group in complex with BBOX (Figure 4d).^39^ This predicted interaction implies that the observed conformational flexibility of the thiazol-4-yl side chain of **58** in complex with FIH is likely not representative of that in complex with BBOX.

The combined crystallographic and computational studies indicate that the substantial improvement in BBOX selectivity observed for **58**, relative to Desidustat, *i.e.*, **58** inhibits BBOX >2500-fold more selectively over PHD2 than Desidustat (Table 5), is likely a result of: (i) enhanced BBOX inhibition due to improved hydrophobic interactions with residues within the BBOX 2OG-binding site (*i.e.*, with the Leu217/Ser229/Phe340 pocket); (ii) enhanced BBOX inhibition due to the formation of electrostatic interactions with the BBOX substrate-binding site (*i.e.*, with the side chain of Asn292), which mimic binding of the GBB carboxylate to BBOX;^39^ and (iii) steric clashes with FIH (Phe207 and Ile281) and PHD2 (Ile327 and Leu343) active site residues that hinder efficient binding of **58** to FIH and PHD2. It is important to note, however, that the kinetics of BBOX catalysis are complicated by dimerization,^39^ with both monomers likely contributing in a cooperative manner to the binding of substrate and cosubstrate to the other monomer.^69^ Thus, empirical studies are required to validate the proposed computational predictions.

## Discussion

The development of efficient and selective BBOX inhibitors is of interest from therapeutic perspectives, *e.g.*, for treatment of cardiovascular disease^9,26,27^ and TNBC,^28,29^ as well as to enable studies that interrogate the (patho)physiological roles of BBOX and l-carnitine.^36^ Based on the observation that the reported BBOX inhibitor AR692B is structurally related to reported PHD inhibitor scaffolds,^36,52^ we profiled a set of five clinically used PHD inhibitors for BBOX inhibition. The results reveal Desidustat,^38,40^ Enarodustat^41,42^ and Vadadustat^43,44^ were potent BBOX inhibitors (IC_50_ values: ∼0.1–0.5 μM; Table 1). Given that these, and structurally related, PHD inhibitors are typically prescribed for long-term use to treat CKD-associated anemia,^52,103,104^ the safety implications of off-target BBOX inhibition by PHD inhibitors requires further consideration. Pharmacokinetic studies conducted in rats indicate Desidustat is rapidly distributed in the liver and kidneys following oral administration.^54^ Since human BBOX localizes in the cytosol and is highly expressed in both kidney and liver tissue,^105^ it is possible that BBOX is exposed to PHD inhibitors administered *in vivo*.

Investigations are required to establish whether BBOX inhibition may contribute to phenotypes, particularly those relating to cardiovascular function, observed following the use of PHD inhibitors in cells and in vivo.^106^ Notably, preclinical studies indicate Enarodustat may attenuate cardiac hypertrophy, potentially offering cardioprotective benefits during CKD-associated anemia treatment.^107^ By contrast, Vadadustat treatment has been associated with increased risk of myocardial infarction in some CKD patients, compared with those treated with the erythropoiesis-stimulating agent (ESA) darbepoetin alpha.^108−110^ Desidustat demonstrates a comparable cardiovascular safety profile to ESAs in CKD patients.^64^

Given that Desidustat, Enarodustat, and Vadadustat are clinically used and that their modular structures are amenable to systematic modification, all three compounds are attractive starting points for the development of improved BBOX inhibitors suitable for cell-based and *in vivo* studies. In this study, computationally guided SAR studies enabled the synthesis of potent and selective BBOX inhibitors derived from the *N*-alkoxyquinoline scaffold of Desidustat. We anticipate that the workflow described here for design of selective BBOX inhibitors can likely also be applied to Enarodustat, Vadadustat and related PHD inhibitors^52,70^ to generate selective BBOX inhibitors and/or selective inhibitors of other 2OG oxygenases which are current medicinal chemistry targets,^111−113^ including AspH,^114−118^ JmjC KDMs,^119−122^ and JMJD5.^123^

Notably, the thiazole-containing Desidustat derivative **58** manifested highly efficient inhibition of BBOX (IC_50_ ∼ 0.02 μM; Table 5, entry xiii) with an IC_50_ value ∼25- and >2500-fold more potent than the reported BBOX inhibitors AR692B and Mildronate, respectively. Thiazole **58** inhibited BBOX with high selectivity over inhibition of a representative panel of structurally- and functionally diverse human 2OG oxygenases, including PHD2 (>2500-fold selectivity), FIH (>2500-fold), KDM4A (>2500-fold), AspH (∼500-fold), and JMJD5 (∼1500-fold) (Table 5, entry xiii). Considering the reported cellular and in vivo efficacy of Desidustat for inhibiting the PHDs,^38,50,64,124^ it is likely that **58** will be a valuable tool compound for cellular and *in vivo* studies into the functional and (patho)physiological roles of BBOX and l-carnitine.

Protein–ligand docking predictions indicate that the thiazole *N*-atom of **58** will be positioned to form a hydrogen bonding interaction with the side chain of Asn292, in a manner which mimics binding of the GBB carboxylate to BBOX,^39^ and which is likely responsible, at least in part, for the increased inhibitory potency of **58** relative to Desidustat (Figure 4). The methyl groups of the α,α-dimethyl glycinamide group of **58** are predicted to bind favorably within a pocket formed by BBOX residues Leu217, Ser229, and Phe340, which is unoccupied in the predicted BBOX:Fe(II):Desidustat complex, and likely prevent efficient binding of **58** to the active sites of FIH and PHD2, likely resulting in the high levels of selectivity manifest by **58**.

Interestingly, the ethyl ester derivatives of **58** and of its 2-pyridyl-containing analogue **57** (*i.e.*, **58a** and **57a**) and of Desidustat itself (*i.e.*, **24**) were also efficient inhibitors of BBOX (IC_50_ values: ∼0.03, ∼0.09, and ∼0.55 μM, respectively; Tables 3 and 5). Moreover, **24**, **57a,** and **58a** had no effect on the activities of all the other human 2OG oxygenases evaluated under the tested conditions (IC_50_ > 50 μM). It should be noted, however, that the ethyl ester groups of **24, 57a,** and **58a** may be unstable in cells with respect to esterase-mediated cleavage. Nonetheless, the inhibitory activities of **24, 57a,** and **58a** demonstrate that the carboxylate group of the Desidustat scaffold can be replaced without a concomitant loss in BBOX inhibitory activity. It is likely that additional Desidustat derivatives containing alternative groups that replace the terminal carboxylate, including those with improved metabolic stabilities, may be identified that also manifest efficient BBOX inhibition.

The design of highly selective 2OG oxygenase inhibitors, in particular those that compete with 2OG, is challenging, in part likely reflecting the structural similarity observed between the 2OG binding sites of many human 2OG oxygenases. Nonetheless, the results described herein showcase how the use of structural and computational information can provide an efficient workflow for identifying unique features of 2OG oxygenase (co)substrate binding sites that may be utilized to generate highly selective 2OG oxygenase inhibitors. Notably, subtle, empirically guided chemical modifications were responsible for substantial changes in inhibitor selectivity. It is likely that these, at least in part, reflect dynamic conformational changes during 2OG oxygenase catalysis, including those relating to the dimeric nature of BBOX and the associated cooperative nature of cosubstrate/substrate binding to BBOX.^39,69^ We envisage that similar strategies may be employed to identify selective active-site-binding inhibitors of other 2OG oxygenases. In addition, the inhibition studies described will likely inform the design of more efficient and selective PHD inhibitors based on the Desidustat scaffold for anemia treatment.

## Experimental Section

The synthesis and characterization of all novel compounds used in this work are described in the associated Supporting Information. Desidustat^50,73^ and AR692B^36^ were synthesized as reported. All compounds were ≥95% pure, as determined by HPLC, ^1^H NMR, and ^13^C NMR analyses, unless stated otherwise. NMR spectra (for all novel compounds) and HPLC traces (for final compounds) are shown in the Supporting Information.

### Production and Purification of Human Recombinant 2OG Oxygenases

BBOX,^39^ PsBBOX AK1 (UniProt ID: P80193),^78^ PHD2_181–426_,^71^ FIH,^80^ His_6_-AspH_315–758_,^125,126^ His_6_-thioredoxin-tagged JMJD5,^67^ and His_6_-KDM4A_1–359_^127^ were prepared according to established procedures. As described, the purified enzymes were >95% pure as determined by SDS-PAGE and MS analyses and had the anticipated masses;^39,67,71,78,80,125−127^ fresh aliquots were used for all inhibition and crystallization studies.

### BBOX SPE-MS Inhibition Assays

Solutions of the small-molecules (original concentration: 20 mM in DMSO) were dry dispensed across 384-well polypropylene V-bottom assay microplates (Greiner) in an ∼3-fold and 11-point dilution series (100 μM inhibitor top concentration; 0.5%_v/v_ final DMSO assay concentration) using an ECHO 550 acoustic dispenser (Labcyte). DMSO and Roxadustat^47^ (final assay concentration: 100 μM) were used as negative- and positive-inhibition controls, respectively. Each reaction was performed in technical duplicate in adjacent wells of the assay plates.

(Co)-substrate/cofactor stock solutions were freshly prepared from commercial solids (Sigma-Aldrich) on the day the assay was performed. LAA: 50 mM in Milli-Q Ultrapure grade water; 2-oxoglutarate (2OG): 100 mM in Milli-Q Ultrapure grade water; ammonium iron(II) sulfate hexahydrate (FAS, (NH_4_)_2_Fe(SO_4_)_2_·6H_2_O): 400 mM in 20 mM aqueous HCl diluted to 5 mM in Milli-Q Ultrapure grade water; γ-butyrobetaine (GBB, (3-carboxypropyl)trimethylammonium chloride): 10 mM in Milli-Q Ultrapure grade water.

The enzyme mixture, containing 0.1 μM full-length human BBOX (*i.e.*, 2× assay concentration) in 50 mM Tris buffer (pH 7.5) containing 200 mM KCl, was dispensed across the inhibitor-containing 384-well plates (25 μL per well) by using a multidrop dispenser (ThermoFischer Scientific) at room temperature under an ambient atmosphere. The plates were centrifuged (1000 rpm, 5 s) and incubated for 15 min at ambient temperature. The substrate mixture, containing 50 μM GBB (*i.e.*, 2× assay concentration), 800 μM 2OG, 1 mM LAA, and 100 μM FAS, was then dispensed across the plates (25 μL per well) using the multidrop dispenser. The plates were centrifuged (1000 rpm, 5 s) and incubated for 20 min at ambient temperature. The enzyme reaction was then stopped by the addition of 10%_v/v_ aqueous formic acid (5 μL per well) by using the multidrop dispenser. The plates were then centrifuged (1000 rpm, 30 s) and analyzed by MS.

Substrate hydroxylation was analyzed by MS using a RapidFire RF 365 high-throughput sampling robot (Agilent) attached to an iFunnel Agilent 6550 accurate mass quadrupole time-of-flight (Q-TOF) mass spectrometer operated in positive ionization mode. Assay samples were aspirated under a vacuum for 0.6 s and loaded onto a HILIC-Z (type H6) solid phase extraction (SPE) cartridge. After loading, the SPE cartridge was washed with 0.1%_v/v_ aqueous formic acid in 85/15_v/v_ acetonitrile/water (5 s, 1.0 mL/min). The substrate and hydroxylated product were eluted from the SPE cartridge with 0.1%_v/v_ aqueous formic acid in 50/50_v/v_ acetonitrile/water (5 s, 0.5 mL/min) into the mass spectrometer and the SPE cartridge re-equilibrated with 0.1%_v/v_ aqueous formic acid in 85/15_v/v_ acetonitrile/water (1 s, 1.0 mL/min). The mass spectrometer was operated in positive ionization mode with the following MS settings: drying gas temperature (280 °C), drying gas flow rate (13 L/min), nebulizer pressure (40 psig), sheath gas temperature (350 °C), sheath gas flow rate (12 L/min), capillary voltage (4000 V), nozzle voltage (1000 V), and fragmentor voltage (365 V).

Peaks corresponding to the substrate (*i.e.*, GBB) and the hydroxylated product (*i.e.*, carnitine) (*m*/*z* = 1) were extracted from the ion chromatogram and integrated using RapidFire Integrator 4.3.0 (Agilent). Peak area data were exported into Microsoft Excel and were used to calculate the % reaction conversion using the following equation
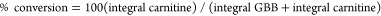


From the raw data, dose–response curves (normalized to the positive and negative inhibition controls) were obtained by nonlinear regression (GraphPad Prism 5), which were used to determine IC_50_ values. Representative dose–response curves are shown in Figure S2.

### PHD2, FIH, AspH, JMJD5, and KDM4A SPE-MS Inhibition Assays

The *in vitro* PHD2,^71^ FIH,^80^ AspH,^68^ JMJD5,^67^ and KDM4A^72^ SPE-MS inhibition assays were performed as reported using purified recombinant human enzymes (PHD2_181–426_, FIH, His_6_-AspH_315–758_, His_6_-thioredoxin-tagged JMJD5, and His_6_-KDM4A_1–359_). A summary of the conditions used for the in vitro SPE-MS inhibition assays is given in Table S2. The synthetic peptide substrates used were: HIF-1α C-terminal oxygen-dependent degradation domain fragment (HIF-1α CODD_556–574_) for PHD2;^71^ HIF-1α C-terminal transactivation domain fragment (HIF-1α C-TAD_788–822_) for FIH;^71^ human Factor X-derived cyclic peptide fragment (hFX-CP_101–119_) for AspH;^68^ 40S ribosomal protein S6 fragment (RPS6_128–148_) for JMJD5;^67^ histone 3 variant fragment (H3_1–15_K9(Me3) with Lys9 bearing three methyl groups at the *N*^ε^ position, Lys4 being substituted by an Ala residue, and Lys14 being substituted by an Ile residue) for KDM4A.^72^ Peptides were prepared as C-terminal amides by GL Biochem (Shanghai) Ltd. Peptide hydroxylations in the cases of FIH, PHD2, AspH, and JMJD5 (+16 Da mass shift) or peptide demethylation in the case of KDM4A (−14 Da mass shift) were monitored by SPE-MS.

### Crystallography

FIH crystallography was carried out as reported.^51^ FIH (final concentration: 0.27 mM) was mixed with Zn(OAc)_2_·2H_2_O (final concentration: 0.5 mM) in Tris buffer (50 mM; pH 7.5) and incubated at 0 °C for 5 min. The Desidustat derivative **58** (100 mM stock solution in DMSO; final concentration: 2 mM) was added, and the mixture was incubated at 0 °C for 15 min. The FIH-inhibitor mixture was then centrifuged using a MicroCL 21R centrifuge (Thermo Fisher Scientific) (18,800*g*, 4 °C, 10 min).

Crystallizations were performed in 96-well, three-subwell, low-profile SWISSCI 3 Lens crystallization plates using a Mosiquito LCP (SPT Labtech) dispensing robot. FIH crystals were grown using the sitting-drop vapor diffusion method at 20 °C in 300 nL sitting drops with 2:1, 1:1, or 1:2 sample/precipitant solution ratios. Crystals were cryo-protected using mother liquor supplemented with 10%_v/v_ glycerol before manual loop cryo-cooling in liquid N_2_. The composition of the precipitant solution used is given in Table S3.

Data were collected at the I03 beamline at the Diamond Light Source (UK). Data were indexed, integrated, and scaled using the Xia2^128^ strategy of the beamline autoprocessing pipeline (Table S3).

The FIH:Zn:**58** complex crystal structure was determined by MR using the AutoMR (PHASER)^129^ subroutine in PHENIX^130^ based on a reported FIH crystal structure (PDB ID: 4B7K(131)). The structural model was refined using COOT^132^ and phenix.refine^130^ (Table S3). Crystal structure data for the FIH:Zn:**58** complex are deposited in the PDB with the PDB accession code: 9JTX. PyMOL (version 4.6.0)^133^ was used for the generation of graphical representations; omit maps were calculated using Polder Maps^134^ in PHENIX (version 1.18.2).^130^

### PsBBOX AK1 Carr–Purcell–Meiboom–Gill (CPMG)-Edited ^1^H NMR Studies

PsBBOX AK1 Carr–Purcell–Meiboom–Gill (CPMG)-edited ^1^H NMR (co)-substrate displacement studies were carried out as reported^61^ at 298 K using a Bruker Avance III 600 MHz spectrometer equipped with a 5 mm BB-F/^1^H Prodigy N_2_ cryoprobe. The PROJECT-CPMG pulse sequence^76^ was used to attenuate broad resonances. The total CPMG time was adjusted to 32 ms. The raw spectra were processed with an exponential window function (line broadening was set to 0.3), and the peaks were referenced to an internal standard (the 1,1,1-trifluoroacetone CH_3_ peak at 1.29 ppm). Bruker 5 mm tubes were used.

The assay mixture contained GBB (20 μM), 2OG (300 μM), and MnCl_2_ (100 μM) in Tris-D_11_ buffer (50 mM in 1:9_v/v_ D_2_O/H_2_O; pH 7.5) containing 80 mM KCl. To this assay mixture was added psBBOX AK1 (15 μM) followed by inhibitor (concentrations as specified Figure S7).^61^

### Protein–Ligand Docking

Reported BBOX:Ni:AR692B (PDB ID: 4C8R(36)), BBOX:Zn:NOG:GBB (PDB ID: 3O2G(39)), and PHD2:Fe:Vadadustat (PDB ID: 7UMP(49)) complex structures were downloaded from the PDB (https://www.rcsb.org/).^135^ Hydrogen atoms were added and Asn/Gln/His residues checked for conformational isomers/flips with REDUCE^136^ using the MolProbity server.^137^ The p*K*_a_ values of ionizable groups were calculated using PropKa,^138^ and ionizable groups were protonated using Pymol (version 4.6.0)^133^ at pH 7.5. Where applicable, the inactive metal ions in the catalytic 2OG oxygenase domain were replaced with Fe(II). Alternative side chain conformations, bound ligands, and all crystallographic waters were removed by using PyMol. Molecular docking studies were performed using the protein–ligand docking software Gold (version 5.1).^75^ For BBOX, an ensemble receptor structure was used (derived from PDB IDs: 4C8R(36) and 3O2G(39)), and the side chain conformation of Arg360 was made flexible; the side chain conformations of all other active site residues were kept rigid. For PHD2, a single receptor structure was used (derived from PDB IDs: 7UMP^49^) and the side chain conformations of all active site residues were kept rigid. For each ligand, 25 genetic algorithm runs were carried out and the GoldScore-CS consensus scoring function of Gold was used to evaluate the predicted ligand binding poses.^139^ The binding site was defined as all atoms within 20 Å of catalytic Fe(II). The “Detect internal H bonds” and “Flip amide bond” ligand flexibility parameters were enabled. The ″allow early termination” option was disabled. The coordination geometry of the Fe(II) ion was set as octahedral. All other settings were used as the defaults.

## Data Availability

The crystal structure data for the FIH:Zn:**58** complex structure has been deposited in the protein data bank^135^ with the PDB accession code: 9JTX. Authors will release the atomic coordinates upon article publication.

## Supporting Information Available

General synthetic procedures; experimental procedures for small-molecule synthesis; mass spectrometry inhibition assays; NMR and HPLC analysis; and crystallographic analysis (PDF)
Molecular formula strings (CSV)
Predicted BBOX:Fe:**40** complex (PDB)
Predicted BBOX:Fe:**41** complex (PDB)
Predicted BBOX:Fe:**44** complex (PDB)
Predicted BBOX:Fe:**46** complex (PDB)
Predicted BBOX:Fe:**48** complex (PDB)
Predicted BBOX:Fe:Desidustat complex (PDB)
Predicted PHD2:Fe:Desidustat complex (PDB)

## Supplementary Material

jm5c00586_si_002.csv

jm5c00586_si_003.pdb

jm5c00586_si_004.pdb

jm5c00586_si_005.pdb

jm5c00586_si_006.pdb

jm5c00586_si_007.pdb

jm5c00586_si_008.pdb

jm5c00586_si_009.pdb

## Abbreviations

2OG: 2-oxoglutarate
AspH: aspartate/asparagine-β-hydroxylase
BBOX: γ-butyrobetaine hydroxylase
CKD: chronic kidney disease
CoA: coenzyme A
CPMG: Carr–Purcell–Meiboom–Gill
FIH: factor inhibiting hypoxia-inducible factor-α
GBB: γ-butyrobetaine
HIF-α: hypoxia-inducible factor-α
HILIC: hydrophilic interaction liquid chromatography
JMJD: Jumonji-C domain-containing protein
KDM: *N*^ε^-methyllysine demethylase
PHD: prolyl hydroxylase domain-containing protein
NOFD: *N*-oxalyl-d-phenylalanine
NOG: *N*-oxalylglycine
SPE-MS: solid-phase extraction coupled to mass spectrometry
TNBC: triple negative breast cancer.
