# Supplementary material for: Derivatives of the Clinically Used HIF Prolyl Hydroxylase Inhibitor Desidustat Are Efficient Inhibitors of Human γ-Butyrobetaine Hydroxylase
Source: J Med Chem. 2025 Apr 23;68(9):9777–98. doi: 10.1021/acs.jmedchem.5c00586 (PMC12067446; doi:10.1021/acs.jmedchem.5c00586)
Supplement: Supplementary file 1 — jm5c00586_si_001.pdf [file jm5c00586_si_001.pdf]

Supporting Information

Derivatives of the Clinically Used HIF Prolyl Hydroxylase Inhibitor Desidustat Are Efficient Inhibitors of Human γ-Butyrobetaine Hydroxylase

Thomas P. Corner[1,#], Anthony Tumber[1], Eidarus Salah[1], Mohammadparsa Jabbary[1], Yu Nakashima[2], Lara I. Schnaubelt[1], Shyam Basak[1], Faisal M. Alshref[1,3], Lennart Brewitz[1,*], and Christopher J. Schofield[1,*]

Author affiliations

[1]Chemistry Research Laboratory, Department of Chemistry and the Ineos Oxford Institute for Antimicrobial Research, University of Oxford, 12 Mansfield Road, Oxford OX1 3TA, U.K.

[2]Institute of Natural Medicine, University of Toyama, 2630-Sugitani, Toyama 930-0194, Japan

[3]Department of Biochemistry, Faculty of Science, King AbdulAziz University, Jeddah 21589, Saudi Arabia

*Email: lennart.brewitz@chem.ox.ac.uk, christopher.schofield@chem.ox.ac.uk

[#]Present Address: Department of Molecular, Cellular, and Developmental Biology, Yale University, New Haven, Connecticut 06511, United States of America

Table of Contents

1. Supporting figures S3

2. Supporting synthetic schemes S15

3. General synthesis information S25

4. General synthetic procedures S26

5. Synthetic procedures and compound characterizations S27

6. ^{1}H and ^{13}C NMR spectra of novel compounds prepared for this study S69

7. HPLC traces of final compounds prepared for this study S139

8. References S154

1. Supporting figures

Supporting Figure S1. Analysis of BBOX-catalyzed γ-butyrobetaine hydroxylation by mass spectrometry. Time-course reactions were performed to investigate the BBOX-catalyzed hydroxylation of γ-butyrobetaine (GBB). Reactions were performed in 96-deep well polypropylene assay plates (Greiner). Full-length recombinant isolated human BBOX (final concentration: 0.05 µM or 0.1 µM) was incubated in the presence of *L*-ascorbate (500 µM), ferrous ammonium sulfate (50 µM), 2-oxoglutarate (2OG; 400 µM) and GBB (25 µM) in Tris buffer (50 mM, pH 7.5, 20 ˚C) containing KCl (200 mM). The extent of GBB hydroxylation was monitored by solid-phase extraction (SPE) coupled to mass spectrometry (MS) using a hydrophilic interaction liquid chromatography (HILIC[1]) SPE cartridge.

(**a**) Extent of BBOX-catalyzed GBB hydroxylation over time. 0.1 µM BBOX: green squares, 0.05 µM BBOX: orange triangles.

(**b**) Deconvoluted MS spectra of the BBOX-catalyzed GBB hydroxylation reaction using 0.1 µM BBOX after 20 min.

(a)

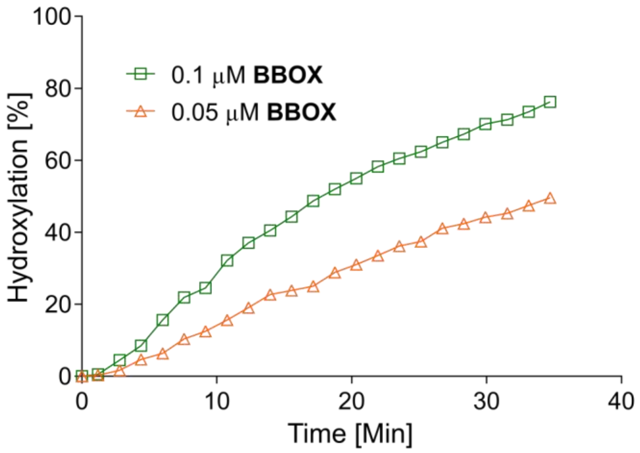

(b)

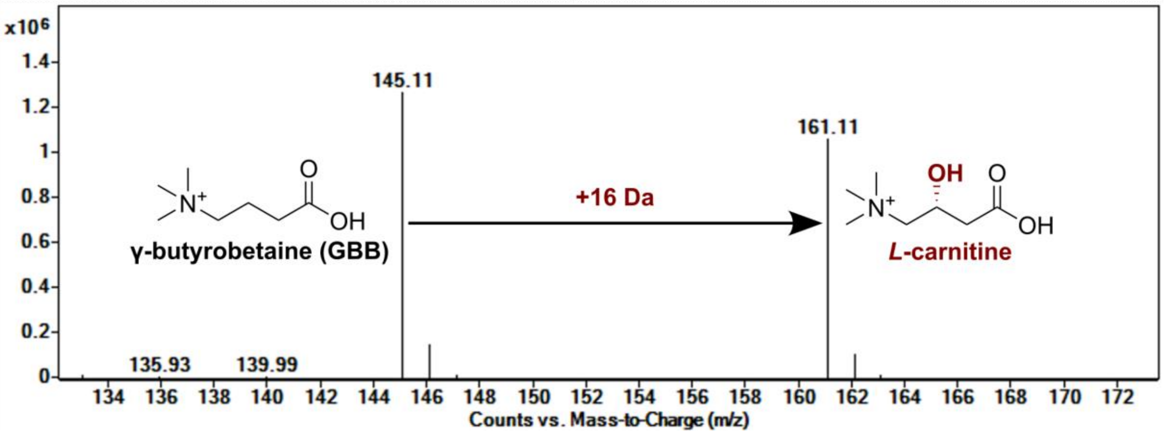

Supporting Figure S2. Representative dose-response curves used to determine IC_{50} values for the inhibition of BBOX by small-molecules investigated in this study (continues on the following two pages). SPE-MS inhibition assays were performed as described in the Experimental Section using recombinant isolated BBOX (0.05 µM), GBB (25 µM), 2OG (400 µM), ammonium iron(III) sulfate (FAS; 50 µM) and *L*-ascorbic acid (LAA; 500 µM) in Tris buffer (50 mM, pH 7.5, 20 ˚C) containing KCl (200 mM). Dose-response curves are means of two technical duplicates (n = 2; mean ± standard deviation, SD). The mean of two independent duplicates each composed of technical duplicates was used to determine IC_{50} values.

(**a**) **Mildronate**:[2] pink circles, **AR692B**:[3] green squares, **Desidustat**:[4] orange diamonds.

(**b**) **Enarodustat**:[5] pink circles, **Vadadustat**:[6] green squares, **Daprodustat**:[7] blue triangles, **Roxadustat**:[8] purple inverse triangles.

(**c**) **8**: pink circles, **9**: green squares, **10**: blue triangles, **11**: purple inverse triangles, **12**: orange hexagons.

(**d**) **13**: pink circles, **14**: green squares, **15**: blue triangles, **16**: purple inverse triangles, **17**: orange hexagons.

(**e**) **18**: pink circles, **19**: green squares, **20**: blue triangles, **21**: purple inverse triangles, **22**: orange hexagons.

(**f**) **23**: pink circles, **24**: green squares, **25**: blue triangles, **26**: purple inverse triangles, **27**: orange hexagons.

(**g**) **28**: pink circles, **29**: green squares, **30**: blue triangles, **31**: purple inverse triangles, **32**: orange hexagons.

(**h**) **40**: pink circles, **41**: green squares, **42**: blue triangles, **43**: purple inverse triangles, **44**: orange hexagons.

(**i**) **45**: pink circles, **46**: green squares, **47**: blue triangles, **48**: purple inverse triangles, **49**: orange hexagons.

(**j**) **50**: pink circles, **51**: green squares, **52**: blue triangles, **53**: purple inverse triangles, **54**: orange hexagons.

(**k**) **57a**: pink circles, **57**: green squares, **58a**: blue triangles, **58**: purple inverse triangles.

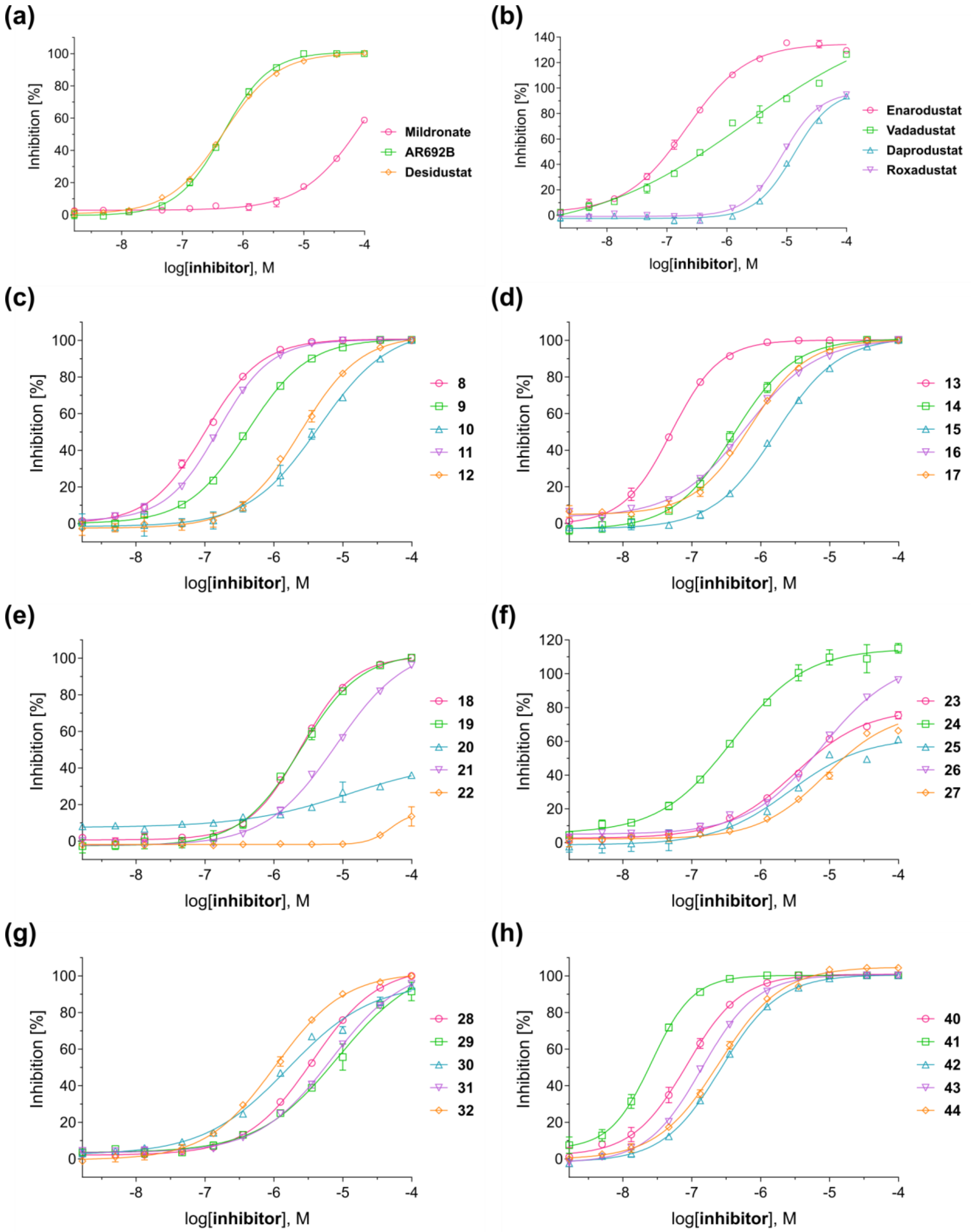
(a)
Inhibition [%]
log[inhibitor], M
Mildronate
AR692B
Desidustat
(b)
Inhibition [%]
log[inhibitor], M
Enarodustat
Vadadustat
Daprodustat
Roxadustat
(c)
Inhibition [%]
log[inhibitor], M
8
9
10
11
12
(d)
Inhibition [%]
log[inhibitor], M
13
14
15
16
17
(e)
Inhibition [%]
log[inhibitor], M
18
19
20
21
22
(f)
Inhibition [%]
log[inhibitor], M
23
24
25
26
27
(g)
Inhibition [%]
log[inhibitor], M
28
29
30
31
32
(h)
Inhibition [%]
log[inhibitor], M
40
41
42
43
44

(i)

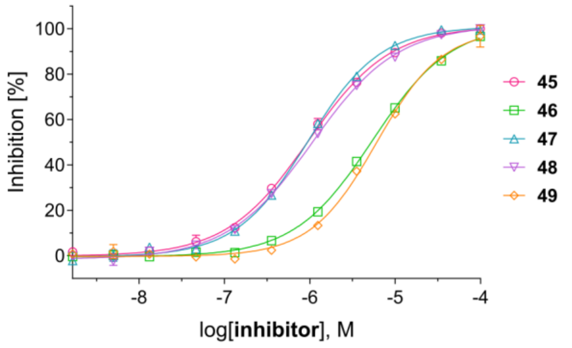
Inhibition [%]
100
80
60
40
20
0
-8
-7
-6
-5
-4
log[**inhibitor**], M
45
46
47
48
49

(j)

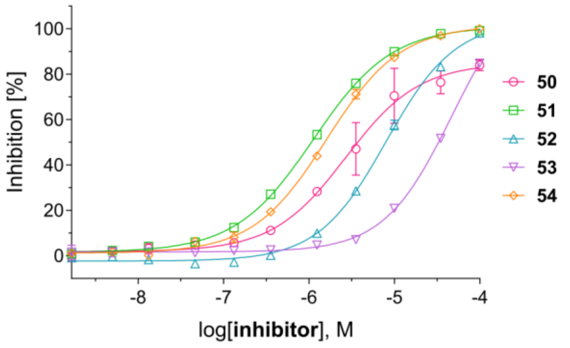
Inhibition [%]
100
80
60
40
20
0
-8
-7
-6
-5
-4
log[**inhibitor**], M
50
51
52
53
54

(k)

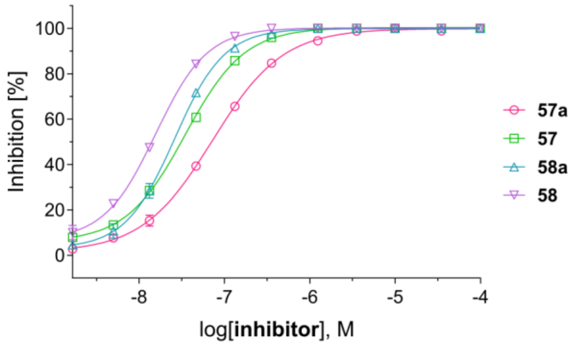
Inhibition [%]
100
80
60
40
20
0
-8
-7
-6
-5
-4
log[**inhibitor**], M
57a
57
58a
58

Supporting Figure S3. Robustness of the BBOX SPE-MS inhibition assays. (**a**) Z'-factors[9] and (**b**) signal-to-noise (S/N) ratios for the BBOX inhibition assay plates analyzed to determine IC_{50} values. The Z'-factors >0.5 (grey line) indicate a stable and robust assay of high quality. Z'-factors and S/N ratios were determined as reported using Microsoft Excel.[9]

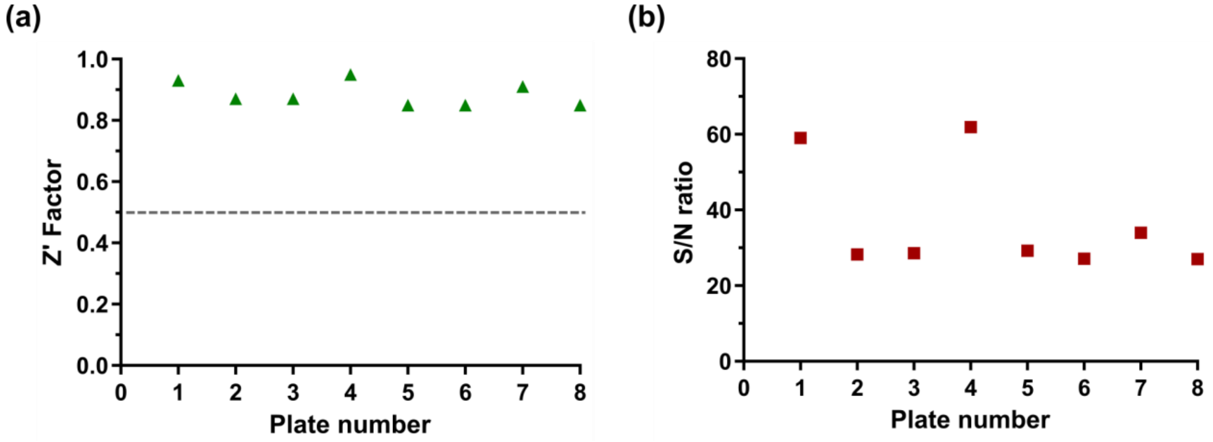

Supporting Figure S4. Reported crystallographic results reveal AR692B binds to the 2OG-binding site of BBOX in two conformations (continues on the following page). A reported BBOX:Ni:AR692B complex structure (PDB ID: 4C8R[3]) contains six BBOX molecules per asymmetric unit corresponding to three homodimers. In three of the monomers in the asymmetric unit, AR692B is observed in an 'extended' conformation in which its pyridine-2-ylmethyl thioether side chain binds within a hydrophobic pocket formed by BBOX active site residues Leu217, Ser229 and Phe340. By contrast, in the other three monomers in the asymmetric unit, AR692B is observed in a 'U-shaped' conformation in which intramolecular π-stacking interactions are observed between its two pyridine rings. Colors: magenta: carbon-backbone of AR692B[3] in its 'extended' BBOX binding mode; cyan: carbon-backbone of AR692B[3] in its 'U-shaped' binding mode; yellow: carbon-backbone of *N*-oxalylglycine (NOG); green: carbon-backbone of γ-butyrobetaine (GBB); grey: Zn; lime green: Ni; red: oxygen; blue: nitrogen.

(**a**) Active site view from a reported BBOX:Ni:AR692B complex structure (PDB ID: 4C8R[3]; ochre: BBOX) showing AR692B[3] adopts an 'extended' BBOX binding mode. AR692B binds to the BBOX active site via bidentate coordination to Ni(II) through its pyridine *N*-atom and exocyclic amide *O*-atom. The carboxylate group of AR692B is positioned to interact with the side chain of Arg360. In its 'extended' BBOX binding mode, the thioether side chain of AR692 binds within a hydrophobic pocket formed by the BBOX residues Leu217, Ser229 and Phe340.

(**b**) Active site view from a reported BBOX:Ni:AR692B complex structure (PDB ID: 4C8R[3]; light pink: BBOX) showing AR692B[3] adopts a 'U-shaped' BBOX binding mode. In its 'U-shaped' BBOX binding mode, a π-stacking interaction is observed between the two pyridine rings of AR692B.

(**c**) Active site view from a reported BBOX:Zn:NOG:GBB complex structure (PDB ID: 3O2G[10]; light grey: BBOX). The 2OG mimetic NOG binds to the BBOX active site metal via its oxalyl carboxylate and keto groups. The glycine carboxylate of NOG is positioned to interact with the side chains of Arg349 and Arg360. GBB binds to BBOX with its C-3 carbon atom proximal to the active site metal. The carboxylate group of GBB is positioned to interact with the side chains of Asn191 and Asn292, whilst its trimethylammonium moiety binds within an 'aromatic cage' in BBOX formed by the side chains of residues Tyr177, Trp181, Tyr194 and Tyr366.

(**d**) Superimposition of views from the reported BBOX:Ni:AR692B (PDB ID: 4C8R[3]; light grey: BBOX) and BBOX:Zn:NOG:GBB (PDB ID: 3O2G[10]; ochre: BBOX) complex structures indicates that AR692B[3] likely completes with 2OG for BBOX binding; however, AR692B is apparently not positioned to interact with BBOX residues that bind GBB.

(**e**) NOG, GBB and the reported BBOX inhibitor AR692B.[3]

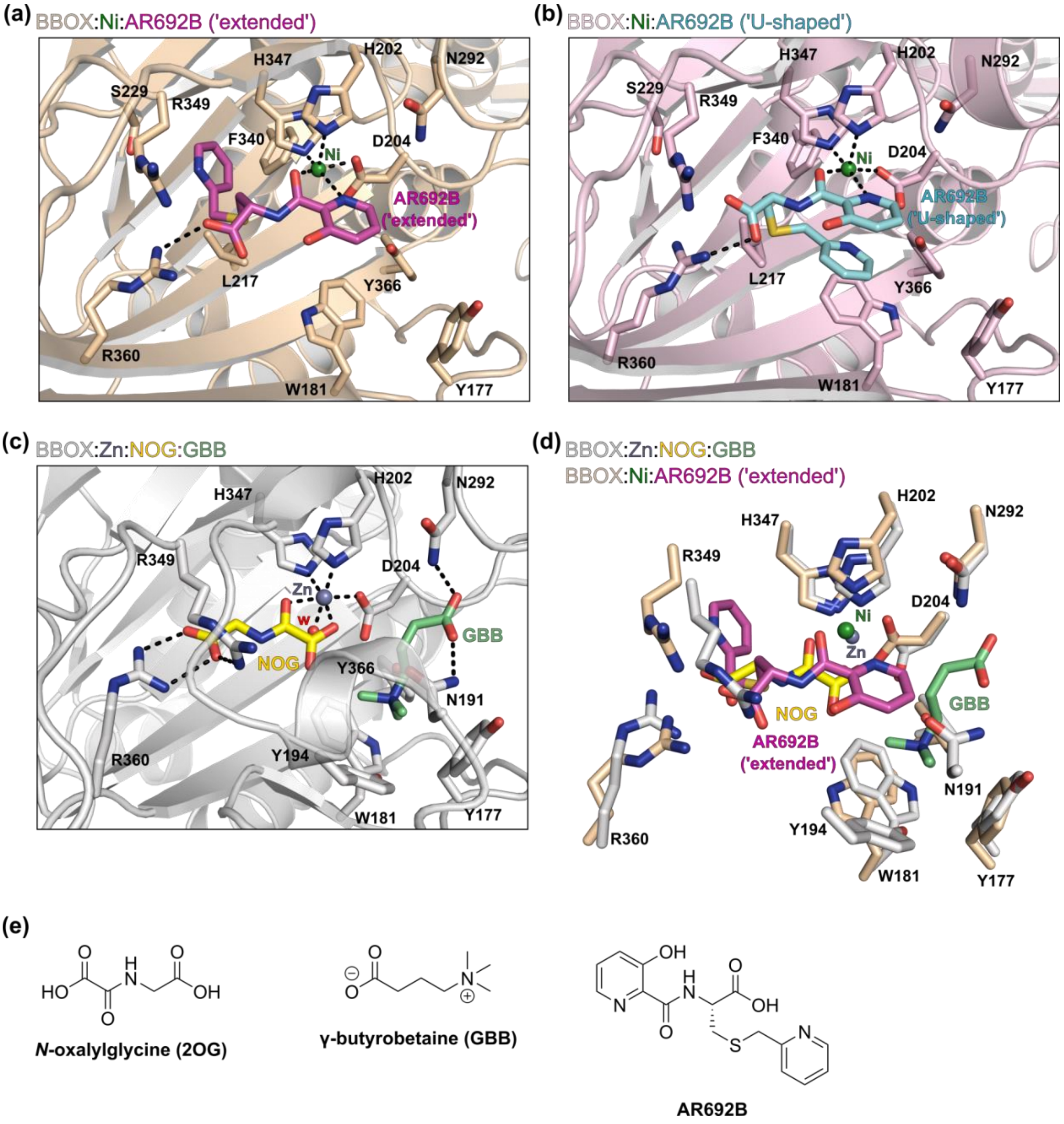
(a) BBOX:Ni:AR692B ('extended')
H347
H202
N292
S229
R349
F340
D204
Ni
AR692B ('extended')
L217
Y366
R360
W181
Y177
(b) BBOX:Ni:AR692B ('U-shaped')
H347
H202
N292
S229
R349
F340
D204
Ni
AR692B ('U-shaped')
L217
Y366
R360
W181
Y177
(c) BBOX:Zn:NOG:GBB
H202
N292
H347
R349
D204
Zn
w
GBB
NOG
Y366
N191
R360
Y194
W181
Y177
(d) BBOX:Zn:NOG:GBB
BBOX:Ni:AR692B ('extended')
H202
H347
N292
R349
D204
Ni
Zn
NOG
GBB
AR692B ('extended')
N191
R360
Y194
W181
Y177
(e)
N-oxalylglycine (2OG)
γ-butyrobetaine (GBB)
AR692B

Supporting Figure S5. Proposed structural basis for the selectivity of AR692B for BBOX inhibition over PHD2 inhibition (continues on the following page). Colors: magenta/cyan, carbon-backbone of AR692B[3] in its 'extended'/'U-shaped' BBOX binding mode; yellow: carbon-backbone of Vadadustat;[6] lime green: Ni; violet: Mn; red: oxygen; blue: nitrogen; green: chlorine. w: water.

(**a**) Active site view from a reported BBOX:Ni:AR692B complex structure (PDB ID: 4C8R[3]; light grey: BBOX) showing AR692B[3] in its 'extended' BBOX binding mode.

(**b**) Active site view from a reported PHD2:Mn:Vadadustat complex structure (PDB ID: 5OX6[11]; light green: PHD2) reveals that Vadadustat[6] competes with 2OG for binding to PHD2. Vadadustat coordinates to the active site Mn(II) via a bidentate mode; its carboxylate group is positioned to interact with the side chains of Tyr329 and Arg383.

(**c**) Superimposition of views from reported BBOX:Ni:AR692B (PDB ID: 4C8R[3]; light grey: BBOX) and PHD2:Mn:Vadadustat (PDB ID: 5OX6[11]; light green: PHD2) complex structures, with AR692B[3] shown in its 'extended' BBOX binding mode, indicates that the binding modes of AR692B to BBOX and Vadadustat[6] to PHD2 are related; however, the thioether side chain of AR692B would likely clash with the side chain of the PHD2 active site residue Leu343, preventing efficient binding of AR692B to PHD2 in its 'extended' conformation.

(**d**) Superimposition of views from the reported BBOX:Ni:AR692B (PDB ID: 4C8R[3]; ochre: BBOX) and PHD2:Mn:Vadadustat (PDB ID: 5OX6[11]; light green: PHD2) complex structures, with AR692B[3] shown in its 'U-shaped' BBOX binding mode, indicates that the binding modes of AR692B to BBOX and Vadadustat[6] to PHD2 are related; however, the thioether side chain of AR692B would likely clash with the side chain of the PHD2 active site residue Ile327, preventing efficient binding of AR692B to PHD2 in its 'U-shaped' conformation. Note, however, that AR692B may adopt different conformations in complex with PHD2 than with BBOX.

(**e**) The reported PHD inhibitor Vadadustat[6] and the reported BBOX inhibitor AR692B.[3]

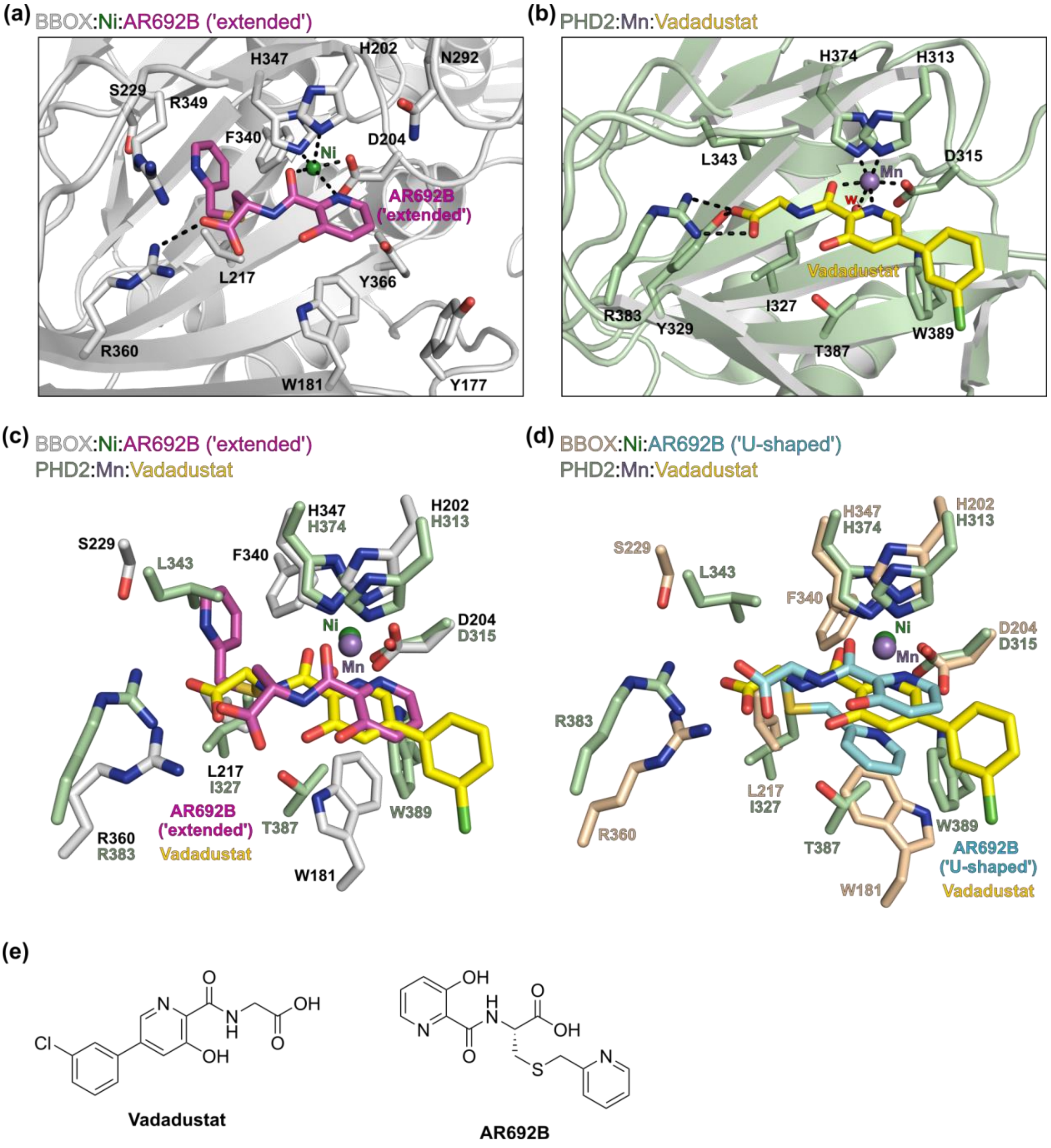
(a) BBOX:Ni:AR692B ('extended')
H347
H202
N292
S229
R349
F340
Ni
D204
AR692B ('extended')
L217
Y366
R360
W181
Y177
(b) PHD2:Mn:Vadadustat
H374
H313
L343
D315
Mn
w
Vadadustat
R383
Y329
I327
T387
W389
(c) BBOX:Ni:AR692B ('extended')
PHD2:Mn:Vadadustat
H347
H374
H202
H313
S229
L343
F340
D204
D315
Ni
Mn
L217
I327
AR692B ('extended')
Vadadustat
T387
W389
R360
R383
W181
(d) BBOX:Ni:AR692B ('U-shaped')
PHD2:Mn:Vadadustat
H347
H374
H202
H313
S229
L343
F340
D204
D315
Ni
Mn
R383
R360
L217
I327
T387
W389
AR692B ('U-shaped')
Vadadustat
W181
(e)
Cl
N
O
N
H
OH
O
OH
Vadadustat
OH
N
O
H
N
O
OH
S
N
AR692B

Supporting Figure S6. Proposed structural basis for the selectivity of AR692B for BBOX inhibition over FIH inhibition (continues of the following page). Colors: magenta/cyan, carbon-backbone of AR692B[3] in its 'extended'/'U-shaped' BBOX binding mode; yellow: carbon-backbone of Vadadustat;[6] lime green: Ni; violet: Mn; red: oxygen; blue: nitrogen; green: chlorine.

(**a**) Active site view from a reported BBOX:Ni:AR692B complex structure (PDB ID: 4C8R[3]; light grey: BBOX) showing AR692B[3] in its 'extended' BBOX binding mode.

(**b**) Active site view from a reported FIH:Mn:Vadadustat complex structure (PDB ID: 5OPC[11]; blue-grey: FIH) reveals that Vadadustat[6] binds to FIH in a 2OG-competing manner. Vadadustat coordinates to the active site Mn(II) via a bidentate mode; its carboxylate group is positioned to interact with the side chain of Lys214.

(**c**) Superimposition of views from reported BBOX:Ni:AR692B (PDB ID: 4C8R[3]; light grey: BBOX) and FIH:Mn:Vadadustat (PDB ID: 5OPC[11]; blue-grey: FIH) complex structures, with AR692B[3] shown in its 'extended' BBOX binding mode, indicates that the binding modes of AR692B to BBOX and Vadadustat[6] to FIH are related; however, the thioether side chain of AR692B would likely clash with the side chain of the FIH active site residues Ile218 and Phe207; thus, preventing efficient binding of AR692B to FIH in its 'extended' conformation.

(**d**) Superimposition of views from reported BBOX:Ni:AR692B (PDB ID: 4C8R[3]; ochre: BBOX) and FIH:Mn:Vadadustat (PDB ID: 5OPC[11]; blue-grey: FIH) complex structures, with AR692B[3] shown in its 'U-shaped' BBOX binding mode, indicates that the binding modes of AR692B to BBOX and Vadadustat[6] to FIH are related; however, the thioether side chain of AR692B would likely clash with the side chain of the FIH active site residue Trp296, thus preventing efficient binding of AR692B to FIH in its 'U-shaped' conformation. Note, however, that AR692B may adopt a different conformation in complex with FIH than with BBOX.

(**e**) The reported PHD inhibitor Vadadustat[6] and the reported BBOX inhibitor AR692B.[3]

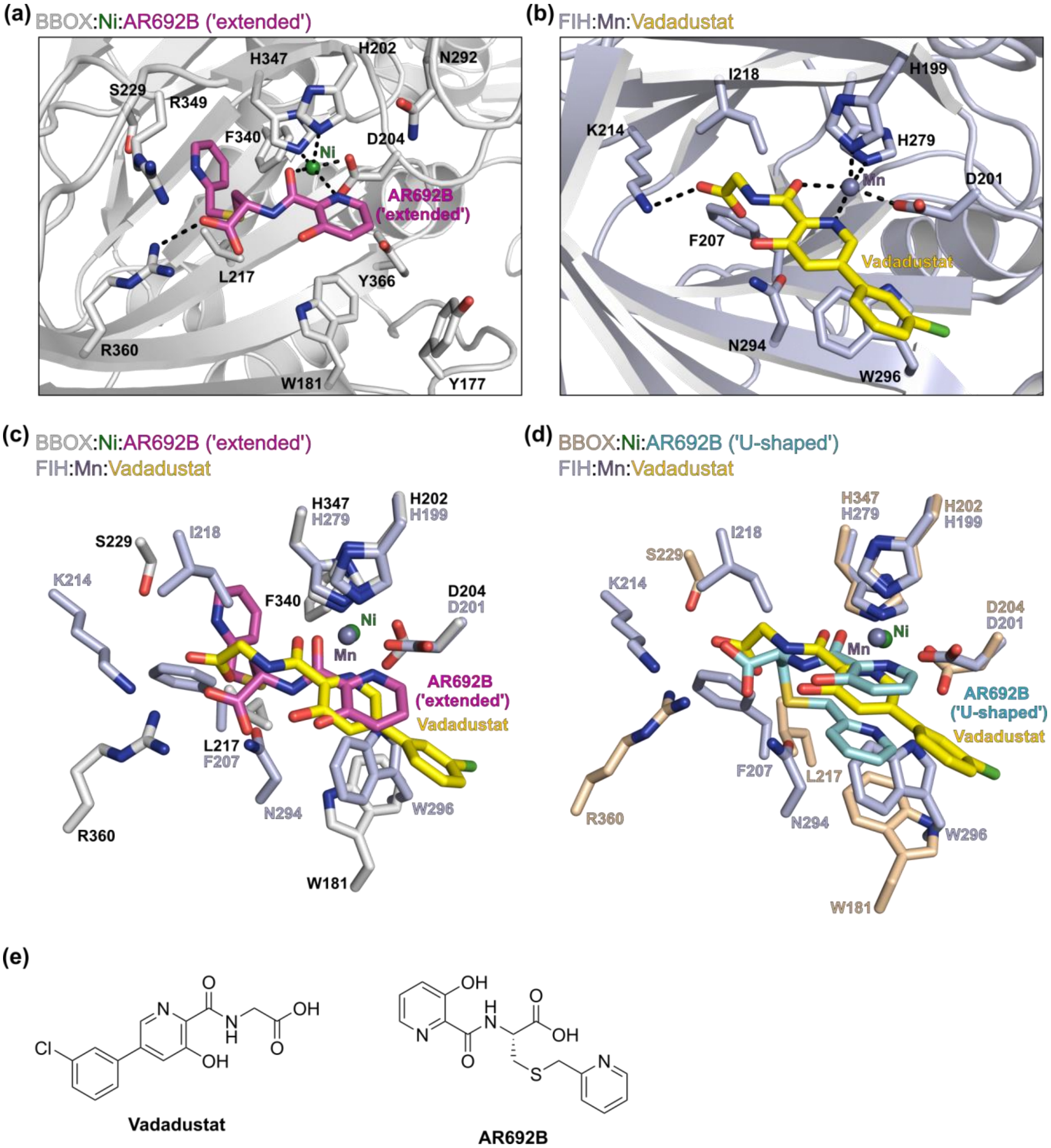
(a) BBOX:Ni:AR692B ('extended')
H347
H202
N292
S229
R349
F340
Ni
D204
AR692B ('extended')
L217
Y366
R360
W181
Y177
(b) FIH:Mn:Vadadustat
I218
H199
K214
H279
Mn
D201
F207
Vadadustat
N294
W296
(c) BBOX:Ni:AR692B ('extended')
FIH:Mn:Vadadustat
H347
H279
H202
H199
S229
I218
K214
F340
D204
D201
Ni
Mn
AR692B ('extended')
Vadadustat
L217
F207
N294
W296
R360
W181
(d) BBOX:Ni:AR692B ('U-shaped')
FIH:Mn:Vadadustat
H347
H279
H202
H199
S229
I218
K214
D204
D201
Mn
Ni
AR692B ('U-shaped')
Vadadustat
F207
L217
R360
N294
W296
W181
(e)
Vadadustat
AR692B

Supporting Figure S7. *Pseudomonas* sp. AK1 BBOX (PsBBOX AK1) Carr-Purcell-Meiboom-Gill (CPMG)-edited ^{1}H NMR (co)-substrate displacement studies. Carr-Purcell-Meiboom-Gill[12] (CPMG)-edited ^{1}H NMR spectra (~2.3-3.1 ppm region) showing the titration of Desidustat[4] into a mixture containing PsBBOX AK1, Mn(II), 2OG and γ-butyrobetaine (GBB). ^{1}H NMR signals corresponding to the hydrogen atoms of the GBB methyl groups (green triangles) as well as to the 2OG C-3 (orange squares) and C-4 (blue circles) atoms are shown. The final assay mixture contained 15 μM PsBBOX AK1, 100 μM Mn(II), 300 μM 2OG, 20 μM GBB and 100 μM Desidustat in Tris-D_{11} buffer (50 mM in 1:$9_{v/v}$ D_2O:H_2O; pH 7.5 containing 80 mM KCl). NMR studies were performed according to a reported procedure,[13] as described in the Experimental Section.

Upon titration of Desidustat into a mixture of PsBBOX AK1, Mn(II), 2OG and GBB, recovery of the CPMG-edited ^{1}H signals of 2OG were observed, indicating that Desidustat displaces 2OG from the PsBBOX AK1 active site. Even at high Desidustat concentrations (*i.e.*, 100 μM), the intensity of the CPMG-edited ^{1}H NMR signal corresponding to the GBB methyl groups was not recovered, implying that Desidustat does not complete with GBB for binding to PsBBOX AK1. It should be noted, however, that the interactions of Desidustat with the PsBBOX AK1 GBB-binding site may not be fully representative of those with the human BBOX GBB-binding site, because of differences in the residues that form the GBB-binding pockets in human BBOX and PsBBOX AK1, *i.e.*, Tyr177, Asn192, Tyr205, Asn292 and Thr295 of human BBOX correspond to Phe184, Ser198, Leu212 and Ala296 and Leu299 in PsBBOX AK1. Additionally, human BBOX and PsBBOX AK1 dimerize in solution,[10] and mechanistic studies have observed cooperative substrate binding likely linked to interactions between the monomers of the dimer,[14] which potentially complicates ligand displacement studies.

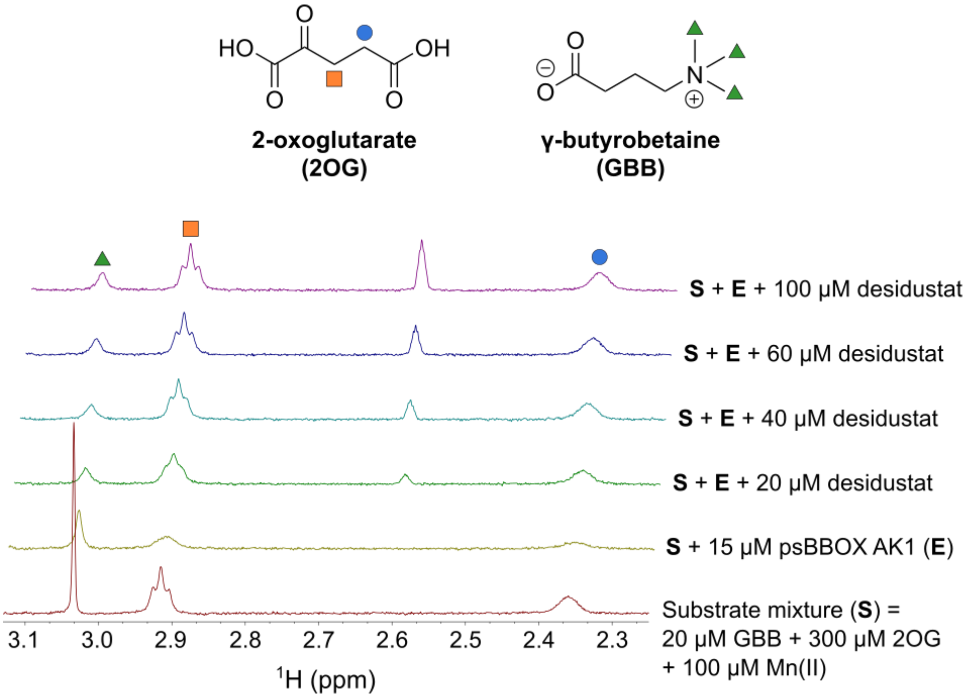

2. Supporting synthetic schemes

Supporting Scheme S1. Synthesis of the Desidustat derivatives 20-22.[a] (**a**) Primary amide **20** was prepared by reacting ethyl ester **7** with methanolic ammonia (yield: 88%). (**b** and **c**) Desidustat derivatives (**b**) **21** and (**c**) **22** were prepared in two steps from ethyl ester **7** (overall yields: 60% and 34%, respectively). First, **7** was coupled with β-alanine ethyl ester or sarcosine ethyl ester to afford ethyl esters **21a** and **22a**, respectively. **21** and **22** were then obtained via lithium hydroxide-mediated saponification.

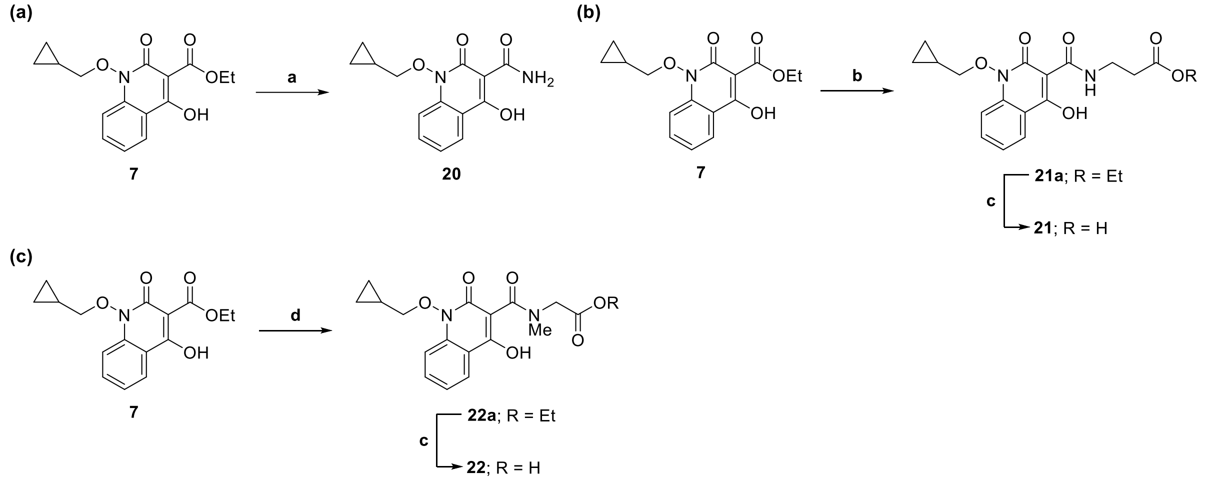

[a]Reagents and conditions: **a**) NH_4OH, MeOH, rt, 88%; **b**) β-alanine ethyl ester, Et_3N, dioxane, 120 °C (sealed tube), 93%; **c**) LiOH, MeOH/H_2O, 0 °C to rt, 60-65%; **d**) sarcosine ethyl ester, Et_3N, dioxane, 120 °C (sealed tube), 57%.

Supporting Scheme S2. Synthesis of the Desidustat derivatives 23-26.[a] (**a**) Esters **23** and **24** were synthesized via coupling of ethyl ester **7** with glycine methyl or ethyl ester hydrochloride. Methyl ester **24** was then reacted with methanolic ammonia to afford primary amide **25**. (**b**) Methyl amide **26** was obtained in two steps from methyl ester **23**. Initially, a lithium hydroxide-mediated saponification of methyl ester **23** afforded Desidustat. Desidustat was then coupled with methylamine hydrochloride using HATU[15] as a coupling reagent to give methyl amide **26**.

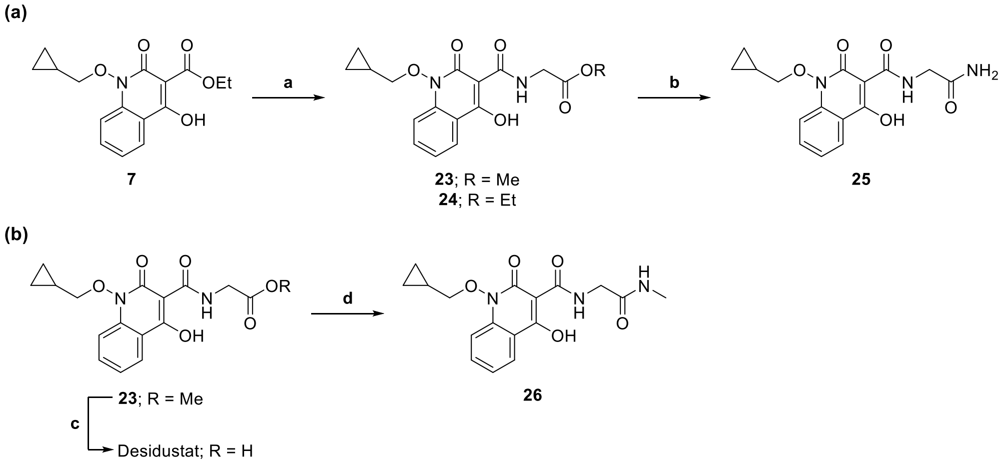

[a]Reagents and conditions: **a**) glycine methyl or ethyl ester hydrochloride, Et_3N, dioxane, 120 °C (sealed tube), 86-94%; **b**) **23**, NH_3/MeOH, rt, 87%; **c**) LiOH, MeO/H_2O, 0 ˚C to rt, 79%; **d**) HCl·NH_2Me, HATU[15], ^{i}Pr_2NEt, DMF, 0 °C to rt, 64%.

Supporting Scheme S3. Synthesis of the Desidustat derivatives 27-29 and 32.[a] The Desidustat derivatives **27**-**29** and **32** were prepared by reacting ethyl ester **7** with primary amines or primary amine hydrochloride salts (overall yields: 40-90%).

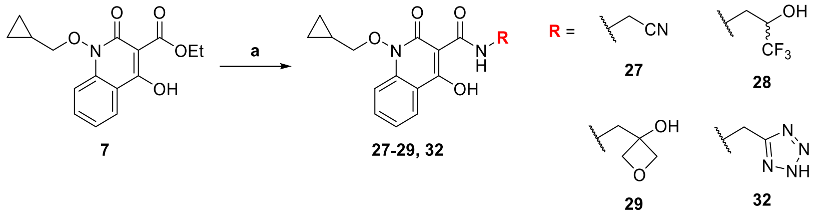

[a]Reagents and conditions: **a**) RNH_2 or $RNH_2{\cdot}HCl$, Et_3N, dioxane, 120 °C (sealed tube), 40-90%.

Supporting Scheme S4. Synthesis of the triazole-containing Desidustat derivatives 30 and 31.[a] The triazoles **30** and **31** were prepared in two steps from primary amide **20** (overall yields: 22 and 36%, respectively). Primary amide **20** was condensed with formaldehyde to give hemiaminal **30a**, which was then chlorinated using thionyl chloride, following by coupling with 1*H*-1,2,3-triazole or 1*H*-1,2,4-triazole to afford triazoles **30** and **31**, respectively.

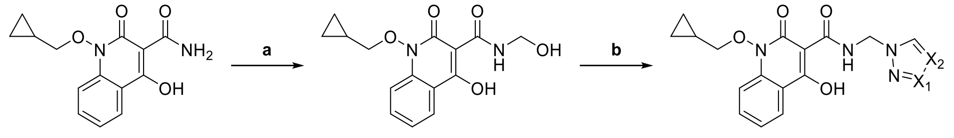

20 **30a** **30**: X_1 = N; X_2 = CH
31: X_1 = CH; X_2 = N

[a]Reagents and conditions: **a**) CH_2O, K_2CO_3, THF, reflux, 95%; **b**) 1*H*-1,2,3-triazole or 1*H*-1,2,4-triazole, CH_2Cl_2, rt, 24% and 40%, respectively.

Supporting Scheme S5. Synthesis of the Desidustat derivatives 52 and 53.[a] The Desidustat derivatives **52** and **53** were prepared in four steps from commercially-sourced isatoic anhydride **52a** according to a modified literature procedure (overall yields: 26 and 34%, respectively).[16] Isatoic anhydride **52a** was alkylated with cyclopropylmethanol or 2-cyclopropylethanol under modified Mitsunobu conditions[17] to afford **52b** and **53a**, respectively. **52b** and **53a** were condensed with ethyl malonate and then reacted with glycine *tert*-butyl ester hydrochloride to give *tert*-butyl esters **52d** and **53c**, respectively. Finally, carboxylic acids **52** and **53** were obtained from **52d** and **53c** following TFA-mediated *tert*-butyl ester cleavage.

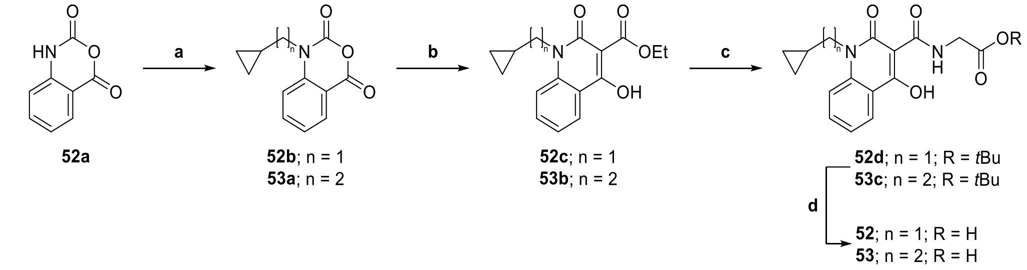

[a]Reagents and conditions: **a**) ROH, DMEAD,[17] PPh_3, CH_2Cl_2, 75-77%; **b**) diethyl malonate, NaH, DMF, 90 °C, 59-87%; **c**) glycine *tert*-butyl ester hydrochloride, Et_3N, dioxane, 120 °C (sealed tube), 81-88%; d) TFA, CH_2Cl_2, rt, 2 h, 60-72%.

Supporting Scheme S6. Synthesis of the monocyclic Desidustat derivative 54.[a] The monocyclic Desidustat derivative **54** was synthesized in six steps from *N*-Boc hydroxylamine **3** (overall yield: 9%). Initially, **3** was reacted with ethyl acrylate in the presence of *tert*-butyl ammonium bromide (TBAB) and potassium hydroxide; the product **54a** was then sequentially deprotected using HCl in dioxane, and coupled with ethyl malonyl chloride to afford **54c**. Sodium ethoxide-mediated cyclisation of **54c** gave ethyl ester **54d**, which was then reacted with glycine methyl ester hydrochloride followed by saponification with lithium hydroxide to afford carboxylic acid **54**.

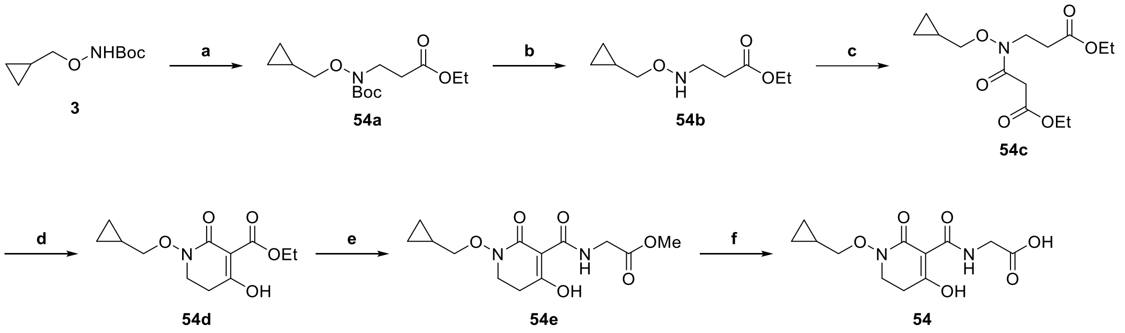

[a]Reagents and conditions: **a**) ethyl acrylate, TBAB, KOH, toluene/H_2O, rt, 72%; **b**) HCl/dioxane, 0 °C to rt, 99%; **c**) ethyl malonyl chloride, Et_3N, EtOAc, 0 °C to rt, 83%; **d**) NaOEt, EtOH, 0 °C to rt, 61%; **e**) glycine methyl ester hydrochloride, Et_3N, dioxane, 120 °C (sealed tube), 60%; **f**) LiOH, MeOH/H_2O, 0 °C to rt, 42%.

3. Supporting tables

Supporting Table S1. Inhibition of human 2OG oxygenases by selected Desidustat derivatives.

	Compound	Structure	IC_{50} [μM][a]					
			**BBOX[b]**	**PHD2[c]**	**FIH[d]**	**AspH[e]**	**KDM4A[f]**	**JMJD5[g]**
i	**9**[h]	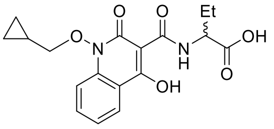	0.33 ± 0.10	>50	>50	19 ± 4	>50	>50
ii	**11**[h]	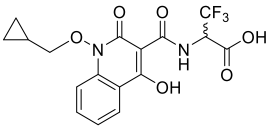	0.11 ± 0.04	>50	>50	8.2 ± 1.2	>50	31 ± 2
iii	**14**	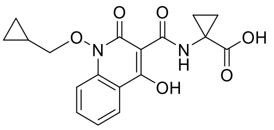	0.33 ± 0.13	30 ± 3	>50	16 ± 3	>50	>50
iv	**16**[h]	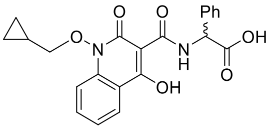	0.42 ± 0.13	>50	>50	9.5 ± 0.2	>50	42 ± 2
v	**17**[h]	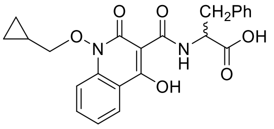	0.50 ± 0.13	>50	24 ± 1	5.5 ± 0.4	>50	10 ± 1
vi	**21**	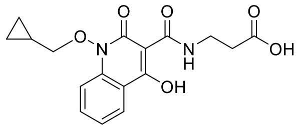	5.9 ± 1.3	5.0 ± 0.4	>50	7.0 ± 1.0	>50	>50
vii	**24**	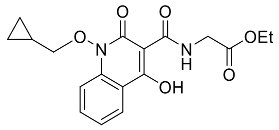	0.55 ± 0.10	>50	>50	>50	>50	>50
viii	**42**	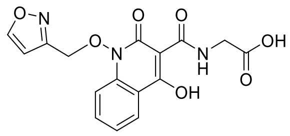	0.21 ± 0.09	1.0 ± 0.1	>50	13 ± 2	>50	>50
ix	**43**	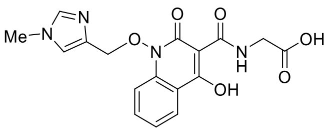	0.11 ± 0.04	2.9 ± 0.4	>50	26 ± 1	>50	>50
x	**44**	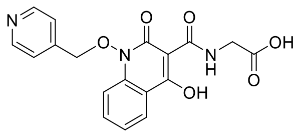	0.20 ± 0.03	1.6 ± 0.1	>50	24 ± 4	>50	>50
xi	**46**	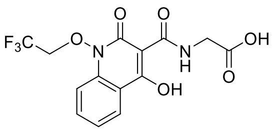	4.1 ± 1.0	1.8 ± 0.4	>50	12 ± 3	>50	>50

xii	**47**	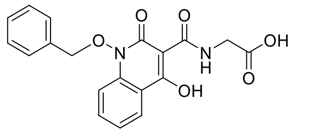	0.79 ± 0.20	1.5 ± 0.2	>50	10 ± 1	>50	19 ± 2
xiii	**52**	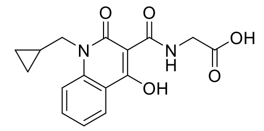	7.2 ± 0.3	5.1 ± 1.2	26 ± 1	11 ± 2	>50	n.d.
xiv	**53**	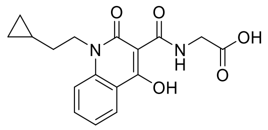	35 ± 6	6.3 ± 0.6	38 ± 6	12 ± 3	>50	n.d.
xv	**54**	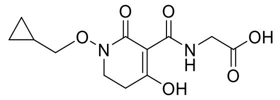	1.2 ± 0.4	4.7 ± 0.7	>50	30 ± 8	>50	>50

[**a**] IC_{50} values are the means ± SD of independent duplicates (each composed of technical duplicates). [**b**] Using 0.05 µM BBOX, 400 µM 2OG and 25 µM GBB. [**c**] Using 0.15 µM $PHD2_{181-426}$, 10 µM 2OG and 5.0 µM HIF-1α $CODD_{556-574}$.[18] [**d**] Using 0.15 µM FIH, 10 µM 2OG and 5.0 µM HIF-1α C-$TAD_{788-822}$.[18] [**e**] Using 0.05 µM His_6-$AspH_{315-758}$, 3 µM 2OG and 1.0 µM hFX-$CP_{101-119}$.[19] [**f**] Using 0.15 µM KDM4A, 10 µM 2OG and 10 µM of a $H3_{1-15}$K9me3 variant.[20] [**g**] Using 0.15 µM JMJD5, 2 µM 2OG and 2.0 µM $RSP6_{128-148}$.[21] [**h**] The chiral Desidustat analogues **9**, **11**, **16** and **17** were prepared as racemic mixtures. Inhibition assays were performed using SPE-MS, as described in the Experimental Section. n.d.: not determined.

Supporting Table S2. Conditions used for the *in vitro* SPE-MS 2OG oxygenase assays.

	Assay	Substrate	Buffer	Incubation time [min][a]	Assay concentration [µM]					Analysed substrate *m/z*	SPE cartridge
					Enzyme	Substrate	2OG	FAS	LAA		
i	BBOX	GBB	50 mM Tris 200 mM KCl pH 7.5	15	0.05	25	400	50	500	1	HILIC
ii	FIH (Ref. 22)	HIF-1α C-TAD$_{788-822}$	50 mM Tris 50 mM NaCl pH 7.5	10	0.15	5	10	10	100	3	C4
iii	PHD2 (Ref. 18)	HIF-1α CODD$_{556-574}$	50 mM Tris 50 mM NaCl pH 7.5	10	0.15	5	10	10	100	2	C4
iv	AspH (Ref. 19)	hFX-CP$_{101-119}$	50 mM HEPES pH 7.5	8	0.05	1	3	2	100	2	C4
v	KDM4A (Ref. 20)	H3$_{1-15}$K9me3 variant	50 mM MOPS pH 7.0	40	0.15	10	10	10	100	3	C4
vi	JMJD5 (Ref. 21)	RSP6$_{128-148}$	50 mM MES pH 7.5	30	0.15	2	2	10	100	3	C4

[**a**] Incubation times may vary between different enzyme batches.

Supporting Table S3. Crystallization conditions, data collection, and refinement statistics for the FIH:Zn:58 complex structure.

	FIH·Zn^{II}·**58** (FIH:**58**)
**PDB ID**	9JTX
**Crystallization**	
Precipitation conditions[a]	0.27 mM FIH,<br>0.5 mM zinc(II) acetate,<br>2 mM **58**,<br>0.1 M HEPES, pH 7.5,<br>6%$_{w/v}$ PEG400,<br>1.6 M ammonium sulfate
**Data collection**	
Space group	$P4_12_12$
Cell dimensions:	
a, *b*, *c* (Å)	86.58, 86.58, 145.18
α, β, γ (°)	90.00, 90.00, 90.00
X-Ray source[b]	Synchrotron (DLS I03)
Resolution (Å)[c]	56.41 - 2.08 (2.12 - 2.08)
R_{merge}	0.080 (4.681)
I / σI	19.3 (0.3)
CC (1/2)	1.000 (0.470)
Total number of reflections	902260 (45341)
Total number unique reflections	33949 (1658)
Completeness (%)	100.0 (100.0)
Multiplicity	26.6 (27.3)
**Refinement**	
R_{work} / R_{free}	0.214 / 0.247
No. atoms:	2920
B-factors:	92.6
R.m.s. deviations:	
Bond lengths (Å)	0.003
Bond angles (°)	1.037

[**a**] Experimental details are described in the Experimental Section. [**b**] DLS: Diamond Light Source. [**c**] Values in parentheses are for the highest-resolution shell.

3. General synthesis information

All reagents were from commercial sources (Sigma-Aldrich, Inc.; Fluorochem Ltd; Tokyo Chemical Industries; Ambeed, Inc.) and used as received. Desidustat,[23, 24] ethyl 1-(cyclopropylmethoxy)-4-hydroxy-2-oxo-1,2-dihydroquinoline-3-carboxylate (**7**),[23, 24] methyl (1-(cyclopropylmethoxy)-4-hydroxy-2-oxo-1,2-dihydroquinoline-3-carbonyl)glycinate (**23**),[24, 25] and AR692B[3] were synthesized as reported. Daprodustat,[7, 26] Vadadustat,[6, 27] Roxadustat,[8, 28] and Enarodustat[5, 29] were from MedChem Express and were used as received. Anhydrous solvents (Sigma-Aldrich, Inc.) were kept under an atmosphere of nitrogen. Purifications were performed using Biotage Isolera One or Biotage Selekt purification machines (wavelengths monitored: 254 and 280 nm) equipped with pre-packed Biotage® Sfär Duo flash chromatography cartridges. The cartridge type and size, as well as solvent gradients (in column volumes, CV) used, are specified in the individual experimental procedures. HPLC grade solvents (Sigma-Aldrich, Inc.) were used for purifications, reaction work-ups, and extractions.

Thin layer chromatography (TLC) was carried out using Merck silica gel 60 F254 TLC plates and visualized using UV light. Melting points (m.p.) were determined using a Stuart SMP-40 automated melting point apparatus. Infrared (IR) spectroscopy was performed using a Bruker Tensor-27 Fourier transform infrared (FT-IR) spectrometer. High-resolution mass spectrometry (HRMS) was performed using electrospray ionization (ESI) mass spectrometry (MS) in the positive or negative ionization mode employing a Thermo Scientific Exactive mass spectrometer (ThermoFisher Scientific); data are presented as a mass-to-charge ratio (*m/z*).

Nuclear magnetic resonance (NMR) spectroscopy was performed using a Bruker AVANCE AVIII HD 600 MHz machine equipped with a 14.1T magnet and with a 5 mm BB-F/^{1}H Prodigy N_2 cryoprobe or a Bruker Avance III HD 500 MHz machine equipped with a 11.75T magnet. Chemical shifts for ^{1}H NMR are reported in parts per million (ppm) downfield from tetramethylsilane and are referenced to the residual protium in the NMR solvent ($CDCl_3$: δ = 7.26 ppm; DMSO-d_6: δ = 2.50 ppm). For ^{13}C NMR, chemical shifts are reported in the scale relative to the NMR solvent ($CDCl_3$: δ = 77.2 ppm; DMSO-d_6: δ = 39.5 ppm). For ^{19}F NMR, chemical shifts are reported in the scale relative to $CFCl_3$. NMR data are reported as follows: chemical shift, multiplicity (s: singlet, d: doublet, dd: doublet of doublets, t: triplet, q: quartet, m: multiplet, br: broad signal), coupling constant (*J*, Hz; accurate to 0.5 Hz), and integration.

4. General synthetic procedures

General Procedure A. A mixture of an ethyl ester (1.0 equiv.), an amine or amine hydrochloride salt (1.15 equiv.) and anhydrous triethylamine (2.0 equiv.) in anhydrous dioxane (0.2 M) in a sealed 10 mL microwave reaction vial was heated in a 120 °C sand bath under a N_2 atmosphere overnight (12-16 h). The reaction mixture was then cooled to ambient temperature and the solvent was removed under reduced pressure. The crude residue was redissolved in ethyl acetate and was washed with H_2O, then brine. The organic layer was dried over anhydrous Na_2SO_4, filtered, and evaporated. The residue was purified using column chromatography to afford the desired amide.

General Procedure B. To a solution of a methyl or ethyl ester (1.0 equiv.) in methanol (0.2 M, HPLC grade) was added an aqueous solution of lithium hydroxide (0.4 M, 2.5 equiv.) under an ambient atmosphere at 0 °C. The reaction mixture was allowed to slowly warm to ambient temperature overnight (12-16 h); the methanol was then removed under reduced pressure. The solution was extracted three time with CH_2Cl_2 (the organic extracts were discarded); the pH of the aqueous phase was acidified (pH~5) by the dropwise addition of 1 N aqueous HCl solution. The water was removed under reduced pressure and the crude residue was purified using reverse-phase column chromatography or by trituration to afford the desired carboxylic acid.

General Procedure C. To a solution of a 1-hydroxyquinolinone derivative (1.0 equiv.) in anhydrous tetrahydrofuran (0.2 M) under a N_2 atmosphere at ambient temperature were added triphenylphosphine (1.5 equiv.), alcohol (1.5 equiv.) and di-2-methoxyethyl azodicarboxylate (DMEAD;[17] 1.5 equiv.). The reaction mixture was stirred overnight (12-16 h) at ambient temperature, before the solvent was removed under reduced pressure; the crude residue was purified using column chromatography to afford the desired 1-alkoxyquinolinone derivative.

General Procedure D. To a solution of a *tert*-butyl ester (1.0 equiv.) in anhydrous dichloromethane (0.2 M) under a N_2 atmosphere at 0 °C was added trifluoroacetic acid (10 equiv.). The reaction mixture was allowed to warm to room temperature and stirred for 2 h. The solvent was removed under reduced pressure and the crude residue was purified by trituration or reverse-phase column chromatography to afford the desired carboxylic acid.

5. Synthetic procedures and compound characterizations

(±)-Ethyl (1-(cyclopropylmethoxy)-4-hydroxy-2-oxo-1,2-dihydroquinoline-3-carbonyl)alaninate (8a)

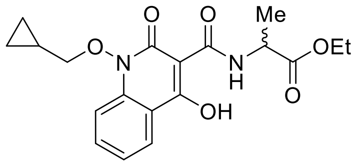

According to General Procedure A, racemic ethyl ester **8a** (78 mg, 93%) was obtained from reported ethyl 1-(cyclopropylmethoxy)-4-hydroxy-2-oxo-1,2-dihydroquinoline-3-carboxylate (**7**)[23, 24] (70 mg, 0.23 mmol) and *DL*-alanine ethyl ester hydrochloride (41 mg, 0.27 mmol), following column chromatography (10 g Sfär Silica D; 35 mL/min; 100% cyclohexane (2 CV), followed by a linear gradient (14 CV): 0%→20% ethyl acetate in cyclohexane).

Yellow oil; ^{1}H NMR (600 MHz, 300 K, $CDCl_3$): δ = 10.44 (d, *J* = 7.0 Hz, 1H), 8.17 (d, *J* = 8.0 Hz, 1H), 7.70 (t, *J* = 8.5 Hz, 1H), 7.65 (d, *J* = 8.5 Hz, 1H), 7.30 (t, *J* = 8.0 Hz, 1H), 4.69 (p, *J* = 7.0 Hz, 1H), 4.24 (q, *J* = 7.0 Hz, 2H), 4.14 – 4.06 (m, 2H), 1.55 (d, *J* = 7.0 Hz, 3H), 1.34 – 1.27 (m, 4H), 0.70 – 0.61 (m, 2H), 0.47 – 0.36 ppm (m, 2H); ^{13}C NMR (151 MHz, 300 K, $CDCl_3$): δ = 172.2, 171.3, 170.5, 159.2, 138.7, 134.2, 125.4, 123.0, 115.1, 112.5, 97.2, 80.8, 61.6, 48.4, 18.1, 14.3, 9.2, 3.6 ppm; IR (film): $\tilde{\nu}$ = 2981, 1743, 1651, 1550, 1406, 1209, 1022 cm^{-1}; HRMS (ESI): *m/z* calcd for $C_{19}H_{23}O_6N_2$ [*M*+H]$^+$: 375.1551, found: 375.1551.

(±)-(1-(Cyclopropylmethoxy)-4-hydroxy-2-oxo-1,2-dihydroquinoline-3-carbonyl)alanine (8)

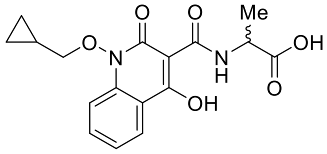

According to General Procedure B, racemic carboxylic acid **8** (27 mg, 40%) was obtained from racemic ethyl ester **8a** (75 mg, 0.20 mmol), following reverse-phase column chromatography (12 g Sfär C18 Duo; 12 mL/min; water (+ 0.1%$_{v/v}$ formic acid) (4 CV), followed by a linear gradient (25 CV): 0%→100% acetonitrile (+ 0.1%$_{v/v}$ formic acid) in water (+ 0.1%$_{v/v}$ formic acid)).

White solid, m.p. 181-183 °C; ^{1}H NMR (600 MHz, 300 K, DMSO-*d*$_6$): δ = 10.39 (d, *J* = 7.0 Hz, 1H), 8.10 (d, *J* = 8.0 Hz, 1H), 7.87 (t, *J* = 8.5 Hz, 1H), 7.71 (d, *J* = 8.5 Hz, 1H), 7.41 (t, *J* = 8.0 Hz, 1H), 4.52 (p, *J* = 7.0 Hz, 1H), 4.05 (d, *J* = 7.5 Hz, 2H), 1.46 (d, *J* = 7.0 Hz, 3H), 1.32 – 1.25 (m, 1H), 0.65 – 0.52 (m, 2H), 0.44 – 0.33 ppm (m, 2H); ^{13}C NMR (151 MHz, 300 K, DMSO-*d*$_6$): δ = 173.0, 170.6, 169.7, 158.0, 138.2, 134.9, 124.6, 123.2, 114.0, 112.5, 96.3, 80.0, 47.9, 17.7, 8.8, 3.2 ppm; IR (film): $\tilde{\nu}$ = 2981, 1714, 1625, 1544, 1405, 1243, 1025 cm^{-1}; HRMS (ESI): *m/z* calcd for $C_{17}H_{19}O_6N_2$ [*M*+H]$^+$: 347.1238, found: 347.1241.

(±)-Methyl 2-(1-(cyclopropylmethoxy)-4-hydroxy-2-oxo-1,2-dihydroquinoline-3-carboxamido)-butanoate (9a)

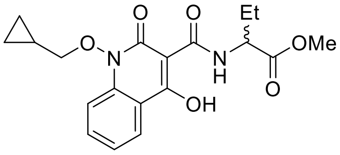

According to General Procedure A, racemic methyl ester **9a** (79 mg, 92%) was obtained from reported ethyl 1-(cyclopropylmethoxy)-4-hydroxy-2-oxo-1,2-dihydroquinoline-3-carboxylate (**7**)[23, 24] (70 mg, 0.23 mmol) and (±)-methyl 2-aminobutanoate hydrochloride (41 mg, 0.27 mmol), following column chromatography (10 g Sfär Silica D; 35 mL/min; 100% cyclohexane (2 CV), followed by a linear gradient (14 CV): 0%→20% ethyl acetate in cyclohexane).

Yellow solid, m.p.: 119-121 °C; ^{1}H NMR (500 MHz, 300 K, $CDCl_3$): δ = 10.47 (d, *J* = 7.5 Hz, 1H), 8.18 (dd, *J* = 8.0, 1.5 Hz, 1H), 7.71 (td, *J* = 8.5, 1.5 Hz, 1H), 7.66 (dd, *J* = 8.5, 1.5 Hz, 1H), 7.31 (td, *J* = 8.0, 1.5 Hz, 1H), 4.66 (td, *J* = 7.5, 5.5 Hz, 1H), 4.16 – 4.06 (m, 2H), 3.78 (s, 3H), 2.06 – 1.96 (m, 1H), 1.96 – 1.86 (m, 1H), 1.36 – 1.28 (m, 1H), 1.04 (t, *J* = 7.5 Hz, 3H), 0.70 – 0.63 (m, 2H), 0.46 – 0.36 ppm (m, 2H); ^{13}C NMR (126 MHz, 300 K, $CDCl_3$): δ = 172.1, 171.3, 170.7, 159.3, 138.7, 134.2, 125.4, 123.0, 115.1, 112.5, 97.2, 80.9, 53.6, 52.5, 25.5, 10.1, 9.2, 3.6 ppm; IR (film): $\tilde{\nu}$ = 3244, 2971, 1747, 1654, 1559, 1407, 1292, 1210, 1144 cm^{-1}; HRMS (ESI): *m/z* calcd for $C_{19}H_{23}N_2O_6$ [*M*+H]$^+$: 375.1551, found: 375.1542.

(±)-2-(1-(Cyclopropylmethoxy)-4-hydroxy-2-oxo-1,2-dihydroquinoline-3-carboxamido)butanoic acid (9)

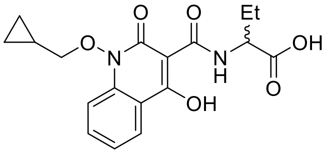

According to General Procedure B, racemic carboxylic acid **9** (27 mg, 58%) was prepared from racemic methyl ester **9a** (50 mg, 0.13 mmol), following reverse-phase column chromatography (12 g Sfär C18 Duo; 12 mL/min; water (+ 0.1%$_{v/v}$ formic acid) (4 CV), followed by a linear gradient (25 CV): 0%→100% acetonitrile (+ 0.1%$_{v/v}$ formic acid) in water (+ 0.1%$_{v/v}$ formic acid)).

White solid, m.p.: 142-146 °C; ^{1}H NMR (500 MHz, 300 K, DMSO-*d*$_6$): δ = 10.42 (d, *J* = 7.0 Hz, 1H), 8.10 (d, *J* = 7.0 Hz, 1H), 7.92 – 7.82 (m, 1H), 7.71 (d, *J* = 8.5 Hz, 1H), 7.41 (t, *J* = 7.5 Hz, 1H), 4.50 (q, *J* = 6.5 Hz, 1H), 4.05 (d, *J* = 7.5 Hz, 2H), 1.97 – 1.78 (m, 2H), 1.33 – 1.26 (m, 1H), 0.93 (t, *J* = 7.5 Hz, 3H), 0.64 – 0.54 (m, 2H), 0.41 – 0.37 ppm (m, 2H); ^{13}C NMR (126 MHz, 300 K, DMSO-*d*$_6$): δ = 172.2, 170.6, 169.9, 158.1, 138.2, 134.9, 124.6, 123.2, 114.0, 112.5, 96.3, 80.0, 53.1, 24.6, 9.5, 8.8, 3.2 ppm; IR (film): $\tilde{\nu}$ = 3249, 3013, 2941, 1745, 1630, 1555, 1414, 1342, 1202, 1025 cm^{-1}; HRMS (ESI): *m/z* calcd for $C_{18}H_{21}O_6N_2$ [*M*+H]$^+$: 361.1394, found: 361.1387.

(±)-Methyl (1-(cyclopropylmethoxy)-4-hydroxy-2-oxo-1,2-dihydroquinoline-3-carbonyl)valinate (10a)

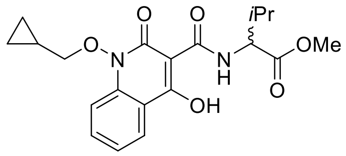

According to General Procedure A, racemic methyl ester **10a** (76 mg, 85%) was obtained from reported ethyl 1-(cyclopropylmethoxy)-4-hydroxy-2-oxo-1,2-dihydroquinoline-3-carboxylate (**7**)[23, 24] (70 mg, 0.23 mmol) and *DL*-valine methyl ester hydrochloride (45 mg, 0.27 mmol), following column chromatography (10 g Sfär Silica D; 35 mL/min; 100% cyclohexane (2 CV), followed by a linear gradient (14 CV): 0%→20% ethyl acetate in cyclohexane).

White solid, m.p.: 112-114 °C; ^{1}H NMR (500 MHz, 300 K, $CDCl_3$): δ = 10.53 (d, *J* = 8.0 Hz, 1H), 8.18 (dd, *J* = 8.0, 1.5 Hz, 1H), 7.71 (td, *J* = 8.5, 1.5 Hz, 1H), 7.67 (dd, *J* = 8.5, 1.5 Hz, 1H), 7.30 (td, *J* = 8.0, 1.5 Hz, 1H), 4.62 (dd, *J* = 8.0, 5.0 Hz, 1H), 4.15 (dd, *J* = 9.5, 7.5 Hz, 1H), 4.07 (dd, *J* = 9.5, 7.5 Hz, 1H), 3.77 (s, 3H), 2.33 (pd, *J* = 7.0, 5.0 Hz, 1H), 1.36 – 1.28 (m, 1H), 1.05 (dd, *J* = 7.0, 4.0 Hz, 6H), 0.70 – 0.62 (m, 2H), 0.45 – 0.36 ppm (m, 2H); ^{13}C NMR (126 MHz, 300 K, $CDCl_3$): δ = 171.8, 171.3, 170.9, 159.3, 138.7, 134.2, 125.4, 123.0, 115.1, 112.5, 97.3, 80.9, 57.7, 52.4, 31.0, 19.4, 18.2, 9.2, 3.6 ppm; IR (film): $\tilde{\nu}$ = 3233, 2966, 1746, 1651, 1557, 1406, 1292, 1210, 1013 cm^{-1}; HRMS (ESI): *m/z* calcd for $C_{20}H_{25}N_2O_6$ [*M*+H]$^+$: 389.1707, found: 389.1697.

(±)-(1-(Cyclopropylmethoxy)-4-hydroxy-2-oxo-1,2-dihydroquinoline-3-carbonyl)valine (10)

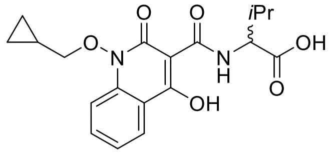

According to General Procedure B, racemic carboxylic acid **10** (30 mg, 62%) was prepared from racemic methyl ester **10a** (50 mg, 0.13 mmol), following reverse-phase column chromatography (12 g Sfär C18 Duo; 12 mL/min; water (+ 0.1%$_{v/v}$ formic acid) (4 CV), followed by a linear gradient (25 CV): 0%→100% acetonitrile (+ 0.1%$_{v/v}$ formic acid) in water (+ 0.1%$_{v/v}$ formic acid)).

White solid, m.p.: 166-168 °C; ^{1}H NMR (600 MHz, 300 K, DMSO-*d*$_6$): δ = 13.16 (br s, 1H), 10.45 (d, *J* = 8.0 Hz, 1H), 8.10 (dd, *J* = 8.0, 1.0 Hz, 1H), 7.87 (td, *J* = 8.5, 1.5 Hz, 1H), 7.72 (d, *J* = 8.5 Hz, 1H), 7.46 – 7.38 (m, 1H), 4.47 (dd, *J* = 8.5, 4.5 Hz, 1H), 4.09 – 4.03 (m, 2H), 2.29 – 2.22 (m, 1H), 1.33 – 1.26 (m, 1H), 0.97 (dd, *J* = 7.0, 1.5 Hz, 6H), 0.63 – 0.56 (m, 2H), 0.44 – 0.36 ppm (m, 2H); ^{13}C NMR (151 MHz, 300 K, DMSO-*d*$_6$): δ = 171.8, 170.5, 170.2, 158.3, 138.2, 134.9, 124.6, 123.3, 114.0, 112.6, 96.3, 80.1, 57.0, 30.3, 19.1, 17.6, 8.8, 3.2 ppm; IR (film): $\tilde{\nu}$ = 3251, 3081, 2970, 1743, 1629, 1555, 1409, 1341, 1181, 1148, 1014 cm^{-1}; HRMS (ESI): *m/z* calcd for $C_{19}H_{23}O_6N_2$ [*M*+H]$^+$: 375.1551, found: 375.1542.

(±)-Methyl 2-(1-(cyclopropylmethoxy)-4-hydroxy-2-oxo-1,2-dihydroquinoline-3-carboxamido)-3,3,3-trifluoropropanoate (11a)

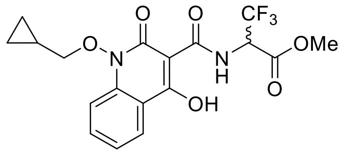

According to General Procedure A, racemic methyl ester **11a** (25 mg, 26%) was obtained from reported ethyl 1-(cyclopropylmethoxy)-4-hydroxy-2-oxo-1,2-dihydroquinoline-3-carboxylate (**7**)[23, 24] (70 mg, 0.23 mmol) and (±)-methyl 2-amino-3,3,3-trifluoropropanoate hydrochloride (52 mg, 0.27 mmol), following column chromatography (10 g Sfär Silica D; 35 mL/min; 100% cyclohexane (2 CV), followed by a linear gradient (14 CV): 0%→20% ethyl acetate in cyclohexane).

White solid, m.p.: 162-163 °C; ^{1}H NMR (500 MHz, 300 K, $CDCl_3$): δ = 11.06 (d, *J* = 9.0 Hz, 1H), 8.19 (dd, *J* = 8.0, 1.5 Hz, 1H), 7.75 (td, *J* = 8.5, 1.5 Hz, 1H), 7.69 (dd, *J* = 8.5, 1.0 Hz, 1H), 7.33 (td, *J* = 8.0, 1.0 Hz, 1H), 5.52 (dq, *J* = 9.0, 7.5 Hz, 1H), 4.12 (d, *J* = 7.5 Hz, 2H), 3.89 (s, 3H), 1.35 – 1.28 (m, 1H), 0.72 – 0.63 (m, 2H), 0.47 – 0.37 ppm (m, 2H); ^{13}C NMR (126 MHz, 300 K, $CDCl_3$): δ = 171.3, 171.1, 164.6, 159.1, 139.0, 134.8, 125.6, 123.3, 122.6 (q, *J* = 283.0 Hz), 114.6, 112.7, 97.1, 81.0, 53.9, 53.6 (q, *J* = 33.0 Hz), 9.2, 3.6 ppm; ^{19}F NMR (470 MHz, 300 K, $CDCl_3$): δ = –71.8 ppm (d, *J* = 7.5 Hz); IR (film): $\tilde{\nu}$ = 3089, 2966, 1765, 1661, 1629, 1543, 1402, 1358, 1292, 1219, 1133 cm^{-1}; HRMS (ESI): *m/z* calcd for $C_{18}H_{18}O_6N_2F_3$ [*M*+H]$^+$: 415.1112, found: 415.1107.

(±)-2-(1-(Cyclopropylmethoxy)-4-hydroxy-2-oxo-1,2-dihydroquinoline-3-carboxamido)-3,3,3-trifluoropropanoic acid (11)

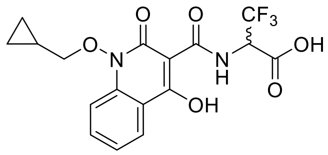

A mixture of racemic methyl ester **11a** (40 mg, 0.10 mmol) in aqueous HCl (12M; 0.7 mL) and acetic acid (4 mL) was heated under reflux for 4 h under an ambient atmosphere, before being cooled to ambient temperature and concentrated under reduced pressure. The crude residue was purified using reverse-phase column chromatography (12 g Sfär C18 Duo; 12 mL/min; water (+ 0.1%$_{v/v}$ formic acid) (4 CV), followed by a linear gradient (25 CV): 0%→100% acetonitrile (+ 0.1%$_{v/v}$ formic acid) in water (+ 0.1%$_{v/v}$ formic acid)) to afford racemic carboxylic acid **11** (13 mg, 34%).

White solid, m.p.: 143-145 °C; ^{1}H NMR (600 MHz, 300 K, DMSO-d_6): δ = 10.89 (d, *J* = 8.5 Hz, 1H), 8.12 (dd, *J* = 8.0, 1.5 Hz, 1H), 7.90 (td, *J* = 8.5, 1.5 Hz, 1H), 7.74 (d, *J* = 8.5 Hz, 1H), 7.44 (t, *J* = 8.0 Hz, 1H), 5.54 (p, *J* = 8.0 Hz, 1H), 4.06 (d, *J* = 7.5 Hz, 2H), 1.33 – 1.27 (m, 1H), 0.67 – 0.54 (m, 2H), 0.48 – 0.34 ppm (m, 2H); ^{13}C NMR (151 MHz, 300 K, DMSO-d_6): δ = 170.5, 170.4(5), 164.7, 158.1, 138.4, 135.3, 124.7, 123.5, 121.2 (q, *J* = 282.5 Hz), 113.7, 112.7, 96.4, 80.2, 53.4 (q, *J* = 30.5 Hz), 8.8, 3.2 ppm; ^{19}F NMR (471 MHz, 300 K, DMSO-d_6): δ = –70.5 ppm (d, *J* = 8.0 Hz); IR (film): $\tilde{\nu}$ = 3225, 3005, 2945, 1755,

1627, 1542, 1407, 1351, 1290. 1209, 1135 cm^{-1}; HRMS (ESI): *m/z* calcd for $C_{17}H_{16}O_6N_2F_3$ [*M*+H]$^+$: 401.0955, found: 401.0952.

(±)-Methyl 2-cyclopropyl-2-(1-(cyclopropylmethoxy)-4-hydroxy-2-oxo-1,2-dihydroquinoline-3-carboxamido)acetate (12a)

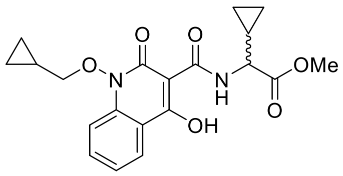

According to General Procedure A, racemic methyl ester **12a** (76 mg, 86%) was obtained from reported ethyl 1-(cyclopropylmethoxy)-4-hydroxy-2-oxo-1,2-dihydroquinoline-3-carboxylate (**7**)[23, 24] (70 mg, 0.23 mmol) and (±)-methyl 2-amino-2-cyclopropylacetate hydrochloride (45 mg, 0.27 mmol), following column chromatography (10 g Sfär Silica D; 35 mL/min; 100% cyclohexane (2 CV), followed by a linear gradient (14 CV): 0%→20% ethyl acetate in cyclohexane).

Yellow solid, m.p.: 90-92 °C; ^{1}H NMR (500 MHz, 300 K, $CDCl_3$): δ = 10.49 (d, *J* = 7.0 Hz, 1H), 8.17 (dd, *J* = 8.0, 1.5 Hz, 1H), 7.71 (td, *J* = 8.5, 1.5 Hz, 1H), 7.69 – 7.64 (m, 1H), 7.31 (td, *J* = 8.0, 1.5 Hz, 1H), 4.16 – 4.05 (m, 3H), 3.80 (s, 3H), 1.35 – 1.23 (m, 2H), 0.71 – 0.56 (m, 5H), 0.52 – 0.46 (m, 1H), 0.46 – 0.37 ppm (m, 2H); ^{13}C NMR (126 MHz, 300 K, $CDCl_3$): δ = 171.8, 171.3, 170.6, 159.2, 138.7, 134.2, 125.4, 123.1, 115.0, 112.5, 97.2, 80.9, 56.4, 52.6, 13.5, 9.2, 3.7, 3.5 ppm; IR (film): $\tilde{\nu}$ = 3234, 3086, 2980, 1749, 1653, 1559, 1469, 1406, 1342, 1292, 1210, 1026 cm^{-1}; HRMS (ESI): *m/z* calcd for $C_{20}H_{23}O_6N_2$ [*M*+H]$^+$: 387.1551, found: 387.1546.

(±)-2-Cyclopropyl-2-(1-(cyclopropylmethoxy)-4-hydroxy-2-oxo-1,2-dihydroquinoline-3-carboxamido)acetic acid (12)

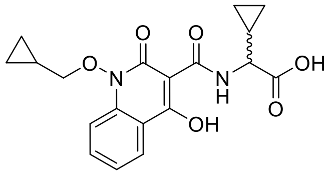

According to General Procedure B, racemic carboxylic acid **12** (21 mg, 43%) was prepared from racemic methyl ester **12a** (50 mg, 0.13 mmol), following trituration with H_2O (3 × 5 mL; Milli-Q® Ultrapure grade) and acetonitrile (3 × 5 mL; HPLC grade).

White solid, m.p.: 186-191 °C; ^{1}H NMR (600 MHz, 300 K, DMSO-*d*$_6$): δ = 13.08 (br s, 1H), 10.39 (d, *J* = 7.0 Hz, 1H), 8.09 (dd, *J* = 8.0, 1.0 Hz, 1H), 7.87 (td, *J* = 8.5, 1.5 Hz, 1H), 7.71 (d, *J* = 8.5 Hz, 1H), 7.46 – 7.35 (m, 1H), 4.05 (d, *J* = 7.5 Hz, 2H), 3.98 (dd, *J* = 8.5, 7.5 Hz, 1H), 1.35 – 1.21 (m, 2H), 0.67 – 0.53 (m, 4H), 0.52 – 0.45 (m, 1H), 0.46 – 0.34 ppm (m, 3H); ^{13}C NMR (151 MHz, 300 K, DMSO-*d*$_6$): δ = 171.9, 170.5, 169.9, 158.1, 138.2, 134.9, 124.6, 123.3, 114.0, 112.5, 96.3, 80.1, 55.5, 13.2, 8.8, 3.2, 3.0 ppm; IR (film): $\tilde{\nu}$ = 3267, 3086, 3019, 1745, 1630, 1544, 1411, 1255, 1175, 1029 cm^{-1}; HRMS (ESI): *m/z* calcd for $C_{19}H_{21}O_6N_2$ [*M*+H]$^+$: 373.1394, found: 373.1391.

Ethyl 2-(1-(cyclopropylmethoxy)-4-hydroxy-2-oxo-1,2-dihydroquinoline-3-carboxamido)-2-methylpropanoate (13a)

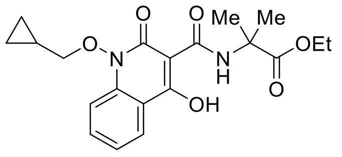

According to General Procedure A, ethyl ester **13a** (78 mg, 84%) was obtained from reported ethyl 1-(cyclopropylmethoxy)-4-hydroxy-2-oxo-1,2-dihydroquinoline-3-carboxylate (**7**)[23, 24] (70 mg, 0.23 mmol) and ethyl 2-amino-2-methyl-propanoate hydrochloride (45 mg, 0.27 mmol), following column chromatography (10 g Sfär Silica D; 35 mL/min; 100% cyclohexane (2 CV), followed by a linear gradient (14 CV): 0%→20% ethyl acetate in cyclohexane).

Yellow oil; ^{1}H NMR (600 MHz, 300 K, $CDCl_3$): δ = 10.40 (s, 1H), 8.17 (d, *J* = 8.0 Hz, 1H), 7.70 (t, *J* = 8.5 Hz, 1H), 7.65 (d, *J* = 8.5 Hz, 1H), 7.30 (t, *J* = 8.0 Hz, 1H), 4.22 (q, *J* = 7.0 Hz, 2H), 4.10 (d, *J* = 7.5 Hz, 2H), 1.64 (s, 6H), 1.36 – 1.28 (m, 1H), 1.25 (t, *J* = 7.0 Hz, 3H), 0.71 – 0.63 (m, 2H), 0.42 – 0.39 ppm (m, 2H); ^{13}C NMR (151 MHz, 300 K, $CDCl_3$): δ = 174.0, 171.4, 170.2, 159.3, 138.6, 134.1, 125.4, 123.0, 115.2, 112.4, 97.4, 80.8, 61.5, 56.4, 25.3, 14.3, 9.2, 3.6 ppm; IR (film): $\tilde{\nu}$ = 2981, 1740, 1650, 1595, 1405, 1289, 1151 cm^{-1}; HRMS (ESI): *m/z* calcd for $C_{20}H_{25}O_6N_2$ $[M+H]^+$: 389.1707, found: 389.1709.

2-(1-(Cyclopropylmethoxy)-4-hydroxy-2-oxo-1,2-dihydroquinoline-3-carboxamido)-2-methylpropanoic acid (13)

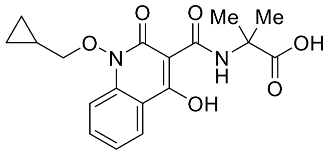

According to General Procedure B, carboxylic acid **13** (22 mg, 33%) was obtained from ethyl ester **13a** (70 mg, 0.18 mmol), following reverse-phase column chromatography (12 g Sfär C18 Duo; 12 mL/min; water (+ 0.1%$_{v/v}$ formic acid) (4 CV), followed by a linear gradient (25 CV): 0%→100% acetonitrile (+ 0.1%$_{v/v}$ formic acid) in water (+ 0.1%$_{v/v}$ formic acid)).

White solid, m.p. 197-200 °C; ^{1}H NMR (600 MHz, 300 K, DMSO-d_6): δ = 10.50 (s, 1H), 8.09 (d, *J* = 8.0 Hz, 1H), 7.87 (t, *J* = 8.0 Hz, 1H), 7.71 (d, *J* = 8.0 Hz, 1H), 7.41 (t, *J* = 8.0 Hz, 1H), 4.04 (d, *J* = 7.5 Hz, 2H), 1.57 (s, 6H), 1.34 – 1.23 (m, 1H), 0.65 – 0.54 (m, 2H), 0.43 – 0.35 ppm (m, 2H); ^{13}C NMR (151 MHz, 300 K, DMSO-d_6): δ = 174.7, 170.6, 169.6, 158.1, 138.1, 134.8, 124.6, 123.2, 114.0, 112.5, 96.6, 80.0, 56.0, 24.5, 8.8, 3.2 ppm; IR (film): $\tilde{\nu}$ = 3212, 2981, 1739, 1628, 1536, 1465, 1403, 1153, 1037 cm^{-1}; HRMS (ESI): *m/z* calcd for $C_{18}H_{21}O_6N_2$ $[M+H]^+$: 361.1394, found: 361.1394.

Methyl 1-(1-(cyclopropylmethoxy)-4-hydroxy-2-oxo-1,2-dihydroquinoline-3-carboxamido)-cyclopropane-1-carboxylate (14a)

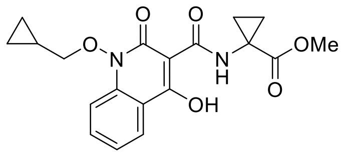

According to General Procedure A, methyl ester **14a** (80 mg, 94%) was obtained from reported ethyl 1-(cyclopropylmethoxy)-4-hydroxy-2-oxo-1,2-dihydroquinoline-3-carboxylate (**7**)[23, 24] (70 mg, 0.23 mmol) and methyl 1-aminocyclopropanecarboxylate hydrochloride (41 mg, 0.27 mmol), following column chromatography (10 g Sfär Silica D; 35 mL/min; 100% cyclohexane (2 CV), followed by a linear gradient (14 CV): 0%→20% ethyl acetate in cyclohexane).

Yellow solid, m.p.: 119-121 °C; ^{1}H NMR (500 MHz, 300 K, $CDCl_3$): δ = 10.43 (s, 1H), 8.19 (dd, *J* = 8.0, 1.5 Hz, 1H), 7.72 (td, *J* = 8.5, 1.5 Hz, 1H), 7.69 – 7.63 (m, 1H), 7.31 (t, *J* = 8.0 Hz, 1H), 4.08 (d, *J* = 7.5 Hz, 2H), 3.72 (s, 3H), 1.67 (q, *J* = 5.0 Hz, 2H), 1.36 – 1.26 (m, 3H), 0.72 – 0.64 (m, 2H), 0.43 – 0.39 ppm (m, 2H); ^{13}C NMR (126 MHz, 300 K, $CDCl_3$): δ = 172.7, 172.4, 171.5, 159.3, 138.7, 134.3, 125.5, 123.1, 115.1, 112.5, 97.4, 80.8, 52.8, 33.2, 17.8, 9.2, 3.6 ppm; IR (film): $\tilde{\nu}$ = 3235, 3016, 1736 , 1631, 1549, 1406, 1340, 1200, 1162 cm^{-1}; HRMS (ESI): *m/z* calcd for $C_{19}H_{21}O_6N_2$ [*M*+H]$^+$: 373.1394, found: 373.1390.

1-(1-(Cyclopropylmethoxy)-4-hydroxy-2-oxo-1,2-dihydroquinoline-3-carboxamido)cyclopropane-1-carboxylic acid (14)

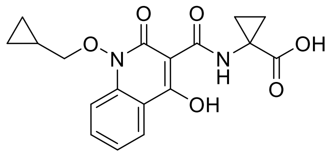

According to General Procedure B, carboxylic acid **14** (29 mg, 62%) was prepared from methyl ester **14a** (50 mg, 0.11 mmol), following reverse-phase column chromatography (12 g Sfär C18 Duo; 12 mL/min; water (+ 0.1%$_{v/v}$ formic acid) (4 CV), followed by a linear gradient (25 CV): 0%→100% acetonitrile (+ 0.1%$_{v/v}$ formic acid) in water (+ 0.1%$_{v/v}$ formic acid)).

White solid, m.p.: 203-206 °C; ^{1}H NMR (500 MHz, 300 K, DMSO-*d*$_6$): δ = 10.32 (s, 1H), 8.13 – 8.07 (m, 1H), 7.90 – 7.83 (m, 1H), 7.70 (d, *J* = 8.5 Hz, 1H), 7.41 (t, *J* = 7.5 Hz, 1H), 4.03 (d, *J* = 7.5 Hz, 2H), 1.49 – 1.45 (m, 2H), 1.32 – 1.23 (m, 3H), 0.66 – 0.54 (m, 2H), 0.44 – 0.32 ppm (m, 2H); ^{13}C NMR (126 MHz, 300 K, DMSO-*d*$_6$): δ = 172.8, 171.9, 170.7, 158.1, 138.2, 134.9, 124.7, 123.2, 114.1, 112.5, 96.6, 80.0, 32.9, 16.5, 8.8, 3.2 ppm; IR (film): $\tilde{\nu}$ = 3272, 3084, 3013, 1692, 1624, 1554, 1464, 1399, 1341, 1292, 1232, 1021 cm^{-1}; HRMS (ESI): *m/z* calcd for $C_{18}H_{19}O_6N_2$ [*M*+H]$^+$: 359.1238, found: 359.1234.

Methyl 1-(1-(cyclopropylmethoxy)-4-hydroxy-2-oxo-1,2-dihydroquinoline-3-carboxamido)-cyclobutane-1-carboxylate (15a)

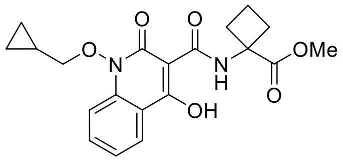

According to General Procedure A, methyl ester **15a** (84 mg, 95%) was obtained from reported ethyl 1-(cyclopropylmethoxy)-4-hydroxy-2-oxo-1,2-dihydroquinoline-3-carboxylate (**7**)[23, 24] (70 mg, 0.23 mmol) and 1-amino-cyclobutanecarboxylic acid methyl ester hydrochloride (45 mg, 0.27 mmol), following column chromatography (10 g Sfär Silica D; 35 mL/min; 100% cyclohexane (2 CV), followed by a linear gradient (14 CV): 0%→20% ethyl acetate in cyclohexane).

Yellow solid, m.p.: 152-154 °C; ^{1}H NMR (500 MHz, 300 K, $CDCl_3$): δ = 10.50 (s, 1H), 8.17 (dd, *J* = 8.0, 1.5 Hz, 1H), 7.71 (td, *J* = 8.5, 1.5 Hz, 1H), 7.66 (dd, *J* = 8.5, 1.5 Hz, 1H), 7.31 (t, *J* = 8.0 Hz, 1H), 4.10 (d, *J* = 7.5 Hz, 2H), 3.77 (s, 3H), 2.85 – 2.73 (m, 2H), 2.45 – 2.38 (m, 2H), 2.18 – 2.03 (m, 2H), 1.36 – 1.29 (m, 1H), 0.71 – 0.63 (m, 2H), 0.43 – 0.39 ppm (m, 2H); ^{13}C NMR (126 MHz, 300 K, $CDCl_3$): δ = 173.5, 171.4, 170.4, 159.3, 138.6, 134.2, 125.4, 123.1, 115.1, 112.5, 97.4, 80.9, 58.3, 52.8, 32.1, 15.7, 9.2, 3.6 ppm; IR (film): $\tilde{\nu}$ = 3257, 3006, 2953, 1742, 1649, 1548, 1406, 1340, 1217, 1124 cm^{-1}; HRMS (ESI): *m/z* calcd for $C_{20}H_{23}N_2O_6$ [*M*+H]$^+$: 387.1551, found: 387.1542.

1-(1-(Cyclopropylmethoxy)-4-hydroxy-2-oxo-1,2-dihydroquinoline-3-carboxamido)cyclobutane-1-carboxylic acid (15)

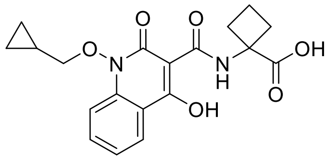

According to General Procedure B, carboxylic acid **15** (27 mg, 52%) was prepared from methyl ester **15a** (50 mg, 0.11 mmol), following reverse-phase column chromatography (12 g Sfär C18 Duo; 12 mL/min; water (+ 0.1%$_{v/v}$ formic acid) (4 CV), followed by a linear gradient (25 CV): 0%→100% acetonitrile (+ 0.1%$_{v/v}$ formic acid) in water (+ 0.1%$_{v/v}$ formic acid)).

White solid, m.p.: 186-188 °C; ^{1}H NMR (500 MHz, 300 K, DMSO-d_6): δ = 10.49 (s, 1H), 8.09 (d, *J* = 8.0 Hz, 1H), 7.91 – 7.83 (m, 1H), 7.71 (d, *J* = 8.5 Hz, 1H), 7.41 (t, *J* = 7.5 Hz, 1H), 4.05 (d, *J* = 7.5 Hz, 2H), 2.65 – 2.58 (m, 2H), 2.46 – 2.39 (m, 2H), 2.01 (p, *J* = 8.0 Hz, 2H), 1.33 – 1.25 (m, 1H), 0.63 – 0.55 (m, 2H), 0.42 – 0.36 ppm (m, 2H); ^{13}C NMR (126 MHz, 300 K, DMSO-d_6): δ = 173.7, 170.6, 169.8, 158.1, 138.2, 134.8, 124.6, 123.2, 114.1, 112.5, 96.6, 80.0, 57.5, 31.1, 15.0, 8.8, 3.2 ppm; IR (film): $\tilde{\nu}$ = 3259, 3012, 2972, 1741, 1628, 1548, 1410, 1342, 1199, 1150 cm^{-1}; HRMS (ESI): *m/z* calcd for $C_{19}H_{21}O_6N_2$ [*M*+H]$^+$: 373.1394, found: 373.1389.

(±)-Methyl 2-(1-(cyclopropylmethoxy)-4-hydroxy-2-oxo-1,2-dihydroquinoline-3-carboxamido)-2-phenylacetate (16a)

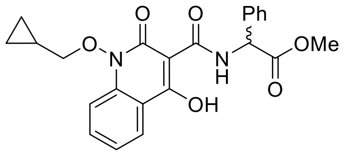

According to General Procedure A, racemic methyl ester **16a** (87 mg, 90%) was obtained from reported ethyl 1-(cyclopropylmethoxy)-4-hydroxy-2-oxo-1,2-dihydroquinoline-3-carboxylate (**7**)[23, 24] (70 mg, 0.23 mmol) and *DL*-phenylglycine methyl ester hydrochloride (55 mg, 0.27 mmol), following column chromatography (10 g Sfär Silica D; 35 mL/min; 100% cyclohexane (2 CV), followed by a linear gradient (14 CV): 0%→20% ethyl acetate in cyclohexane).

White solid, m.p.: 154-155 °C; ^{1}H NMR (500 MHz, 300 K, $CDCl_3$): δ = 11.00 (d, *J* = 7.0 Hz, 1H), 8.17 (d, *J* = 8.0 Hz, 1H), 7.71 (t, *J* = 8.5 Hz, 1H), 7.67 (d, *J* = 8.5 Hz, 1H), 7.52 – 7.47 (m, 2H), 7.42 – 7.33 (m, 3H), 7.30 (t, *J* = 8.0 Hz, 1H), 5.74 – 5.68 (m, 1H), 4.16 – 4.05 (m, 2H), 3.77 (s, 3H), 1.35 – 1.28 (m, 1H), 0.69 – 0.63 (m, 2H), 0.44 – 0.36 ppm (m, 2H); ^{13}C NMR (126 MHz, 300 K, $CDCl_3$): δ = 171.2, 170.7, 170.3, 159.2, 138.8, 136.0, 134.3, 129.2, 128.8, 127.6, 125.4, 123.1, 115.0, 112.5, 97.3, 80.9, 56.9, 53.0, 9.2, 3.6 ppm; IR (film): $\tilde{\nu}$ = 3228, 2955, 1748, 1651, 1539, 1494, 1405, 1340, 1292, 1214, 1170 cm^{-1}; HRMS (ESI): *m/z* calcd for $C_{23}H_{23}N_2O_6$ [*M*+H]$^+$: 423.1551, found: 423.1542.

(±)-2-(1-(Cyclopropylmethoxy)-4-hydroxy-2-oxo-1,2-dihydroquinoline-3-carboxamido)-2-phenylacetic acid (16)

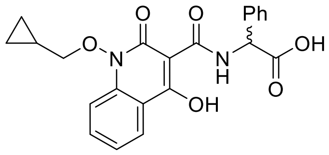

According to General Procedure B, racemic carboxylic acid **16** (32 mg, 65%) was prepared from racemic methyl ester **16a** (50 mg, 0.12 mmol), following trituration with H_2O (3 × 5 mL; Milli-Q® Ultrapure grade) and MeOH (3 × 5 mL; HPLC grade).

White solid, m.p.: >250 °C (decomposition); ^{1}H NMR (600 MHz, 300 K, DMSO-*d*$_6$): δ = 13.63 – 13.24 (br s, 1H), 10.91 (d, *J* = 6.5 Hz, 1H), 8.09 (dd, *J* = 8.0, 1.0 Hz, 1H), 7.87 (td, *J* = 8.5, 1.5 Hz, 1H), 7.72 (d, *J* = 8.0 Hz, 1H), 7.47 – 7.39 (m, 5H), 7.39 – 7.35 (m, 1H), 5.56 (d, *J* = 6.5 Hz, 1H), 4.06 (d, *J* = 7.5 Hz, 2H), 1.33 – 1.26 (m, 1H), 0.62 – 0.56 (m, 2H), 0.42 – 0.36 ppm (m, 2H); ^{13}C NMR (151 MHz, 300 K, DMSO-*d*$_6$): δ = 170.9, 170.4, 169.5, 158.1, 138.3, 136.8, 135.0, 128.9, 128.3, 127.1, 124.6, 123.3, 113.9, 112.6, 96.5, 80.1, 56.4, 8.8, 3.2 ppm; IR (film): $\tilde{\nu}$ = 3267, 3020, 2942, 1739, 1627, 1583, 1542, 1400, 1291, 1223, 1173, 1027 cm^{-1}; HRMS (ESI): *m/z* calcd for $C_{22}H_{21}O_6N_2$ [*M*+H]$^+$: 409.1394, found: 409.1387.

(±)-Methyl (1-(cyclopropylmethoxy)-4-hydroxy-2-oxo-1,2-dihydroquinoline-3-carbonyl)-phenylalaninate (17a)

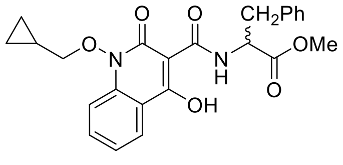

According to General Procedure A, racemic methyl ester **17a** (98 mg, 96%) was obtained from reported ethyl 1-(cyclopropylmethoxy)-4-hydroxy-2-oxo-1,2-dihydroquinoline-3-carboxylate (**7**)[23, 24] (70 mg, 0.23 mmol) and *DL*-phenylalanine methyl ester hydrochloride (57 mg, 0.27 mmol), following column chromatography (10 g Sfär Silica D; 35 mL/min; 100% cyclohexane (2 CV), followed by a linear gradient (14 CV): 0%→20% ethyl acetate in cyclohexane).

Yellow oil; ^{1}H NMR (600 MHz, 300 K, $CDCl_3$): δ = 10.51 (d, *J* = 7.5 Hz, 1H), 8.15 (d, *J* = 8.0 Hz, 1H), 7.70 (t, *J* = 8.5 Hz, 1H), 7.65 (d, *J* = 8.5 Hz, 1H), 7.31 – 7.27 (m, 3H), 7.26 – 7.20 (m, 3H), 4.94 (td, *J* = 8.0, 5.5 Hz, 1H), 4.11 (dd, *J* = 9.5, 7.5 Hz, 1H), 4.05 (dd, *J* = 9.5, 7.5 Hz, 1H), 3.74 (s, 3H), 3.28 (dd, *J* = 14.0, 5.5 Hz, 1H), 3.15 (dd, *J* = 14.0, 8.0 Hz, 1H), 1.34 – 1.23 (m, 1H), 0.70 – 0.62 (m, 2H), 0.45 – 0.35 ppm (m, 2H); ^{13}C NMR (151 MHz, 300 K, $CDCl_3$): δ = 171.5, 171.2, 170.6, 159.1, 138.8, 136.2, 134.2, 129.4, 128.8, 127.2, 125.4, 123.0, 115.0, 112.5, 97.2, 80.8, 54.0, 52.6, 38.3, 9.2, 3.6 ppm; IR (film): $\tilde{\nu}$ = 2981, 1747, 1650, 1550, 1405, 1219 cm^{-1}; HRMS (ESI): *m/z* calcd for $C_{24}H_{25}O_6N_2$ [*M*+H]$^+$: 437.1707, found: 437.1700.

(±)-(1-(Cyclopropylmethoxy)-4-hydroxy-2-oxo-1,2-dihydroquinoline-3-carbonyl)phenylalanine (17)

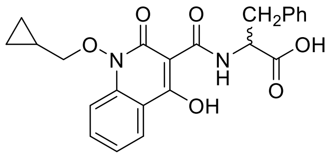

According to General Procedure B, racemic carboxylic acid **17** (25 mg, 33%) was obtained from racemic methyl ester **17a** (80 mg, 0.18 mmol), following reverse-phase column chromatography (12 g Sfär C18 Duo; 12 mL/min; water (+ 0.1%$_{v/v}$ formic acid) (4 CV), followed by a linear gradient (25 CV): 0%→100% acetonitrile (+ 0.1%$_{v/v}$ formic acid) in water (+ 0.1%$_{v/v}$ formic acid)).

Yellow solid, m.p. 186-188 °C; ^{1}H NMR (600 MHz, 300 K, DMSO-*d*$_6$): δ = 13.28 (br s, 1H), 10.34 (d, *J* = 7.5 Hz, 1H), 8.09 (d, *J* = 8.0 Hz, 1H), 7.86 (t, *J* = 8.5 Hz, 1H), 7.70 (d, *J* = 8.5 Hz, 1H), 7.41 (t, *J* = 8.0 Hz, 1H), 7.32 – 7.25 (m, 2H), 7.24 – 7.21 (m, 3H), 4.80 (q, *J* = 7.0 Hz, 1H), 4.06 – 3.98 (m, 2H), 3.24 (dd, *J* = 14.0, 5.5 Hz, 1H), 3.13 (dd, *J* = 14.0, 7.0 Hz, 1H), 1.33 – 1.22 (m, 1H), 0.59 – 0.56 (m, 2H), 0.38 – 0.35 ppm (m, 2H); ^{13}C NMR (151 MHz, 300 K, DMSO-*d*$_6$): δ = 171.6, 170.5, 169.8, 157.9, 138.2, 136.5, 134.9, 129.3, 128.3, 126.8, 124.6, 123.2, 113.9, 112.5, 96.3, 80.0, 53.4, 36.8, 8.8, 3.2 ppm; IR (film): $\tilde{\nu}$ = 2981, 1741, 1627, 1545, 1407, 1216, 1025 cm^{-1}; HRMS (ESI): *m/z* calcd for $C_{23}H_{23}O_6N_2$ [*M*+H]$^+$: 423.1551, found: 423.1551.

(±)-Methyl 2-(1-(cyclopropylmethoxy)-4-hydroxy-2-oxo-1,2-dihydroquinoline-3-carboxamido)-4-phenylbutanoate (18a)

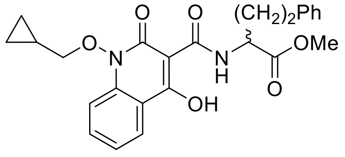

According to General Procedure A, racemic methyl ester **18a** (94 mg, 91%) was obtained from reported ethyl 1-(cyclopropylmethoxy)-4-hydroxy-2-oxo-1,2-dihydroquinoline-3-carboxylate (**7**)[23, 24] (70 mg, 0.23 mmol) and *DL*-homophenylalanine methyl ester hydrochloride (62 mg, 0.27 mmol), following column chromatography (10 g Sfär Silica D; 35 mL/min; 100% cyclohexane (2 CV), followed by a linear gradient (14 CV): 0%→30% ethyl acetate in cyclohexane).

Yellow solid, m.p.: 150-152 °C; ^{1}H NMR (600 MHz, 300 K, $CDCl_3$): δ = 10.56 (d, *J* = 7.5 Hz, 1H), 8.23 – 8.15 (m, 1H), 7.72 (td, *J* = 8.5, 1.5 Hz, 1H), 7.68 (d, *J* = 8.5 Hz, 1H), 7.34 – 7.30 (m, 1H), 7.30 – 7.27 (m, 2H), 7.23 – 7.16 (m, 3H), 4.74 (td, *J* = 8.0, 5.0 Hz, 1H), 4.15 (t, *J* = 8.5 Hz, 1H), 4.09 (dd, *J* = 9.5, 7.5 Hz, 1H), 3.75 (s, 3H), 2.77 (t, *J* = 8.0 Hz, 2H), 2.35 – 2.27 (m, 1H), 2.23 – 2.14 (m, 1H), 1.37 – 1.30 (m, 1H), 0.72 – 0.63 (m, 2H), 0.47 – 0.38 ppm (m, 2H); ^{13}C NMR (151 MHz, 300 K, $CDCl_3$): δ = 172.0, 171.3, 170.8, 159.3, 159.2(6), 140.5, 138.7, 134.3, 128.6, 126.3, 125.5, 123.1, 115.1, 112.5, 97.2, 80.9, 52.6, 52.0, 33.7, 31.9, 9.2, 3.6 ppm; IR (film): $\tilde{\nu}$ = 3222, 3026, 2924, 1747, 1651, 1558, 1406, 1292, 1013 cm^{-1}; HRMS (ESI): *m/z* calcd for $C_{25}H_{27}N_2O_6$ [*M*+H]$^+$: 451.1864, found: 451.1854.

(±)-2-(1-(Cyclopropylmethoxy)-4-hydroxy-2-oxo-1,2-dihydroquinoline-3-carboxamido)-4-phenylbutanoic acid (18)

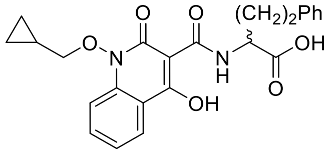

According to General Procedure B, racemic carboxylic acid **18** (27 mg, 56%) was prepared from racemic methyl ester **18a** (50 mg, 0.11 mmol), following reverse-phase column chromatography (12 g Sfär C18 Duo; 12 mL/min; water (+ 0.1%$_{v/v}$ formic acid) (4 CV), followed by a linear gradient (25 CV): 0%→100% acetonitrile (+ 0.1%$_{v/v}$ formic acid) in water (+ 0.1%$_{v/v}$ formic acid)).

White solid, m.p.: 208-212 °C; ^{1}H NMR (500 MHz, 300 K, DMSO-*d*$_6$): δ = 10.46 (d, *J* = 7.5 Hz, 1H), 8.10 (d, *J* = 7.0 Hz, 1H), 7.93 – 7.83 (m, 1H), 7.72 (d, *J* = 8.5 Hz, 1H), 7.42 (t, *J* = 7.5 Hz, 1H), 7.27 (t, *J* = 7.5 Hz, 2H), 7.21 (d, *J* = 7.0 Hz, 2H), 7.16 (t, *J* = 7.0 Hz, 1H), 4.58 – 4.47 (m, 1H), 4.06 (d, *J* = 7.5 Hz, 2H), 2.67 (t, *J* = 8.0 Hz, 2H), 2.26 – 2.18 (m, 1H), 2.14 – 2.06 (m, 1H), 1.34 – 1.26 (m, 1H), 0.67 – 0.53 (m, 2H), 0.42 – 0.38 ppm (m, 2H); ^{13}C NMR (126 MHz, 300 K, DMSO-*d*$_6$): δ = 172.3, 170.5, 170.0, 158.1, 140.7, 138.2, 134.9, 128.4, 128.3, 126.0, 124.6, 123.2, 114.0, 112.5, 96.4, 80.0, 51.8, 33.0, 31.1, 8.8, 3.2 ppm; IR (film): $\tilde{\nu}$ = 3240, 3088, 2968, 1734, 1622, 1576, 1541, 1405, 1296, 1235, 1203, 1020 cm^{-1}; HRMS (ESI): *m/z* calcd for $C_{24}H_{25}O_6N_2$ [*M*+H]$^+$: 437.1707, found: 437.1701.

(±)-Methyl (1-(cyclopropylmethoxy)-4-hydroxy-2-oxo-1,2-dihydroquinoline-3-carbonyl)-tryptophanate (19a)

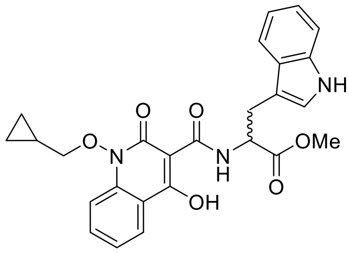

According to General Procedure A, racemic methyl ester **19a** (97 mg, 89%) was obtained from reported ethyl 1-(cyclopropylmethoxy)-4-hydroxy-2-oxo-1,2-dihydroquinoline-3-carboxylate (**7**)[23, 24] (70 mg, 0.23 mmol) and *DL*-tryptophan methyl ester hydrochloride (69 mg, 0.27 mmol), following column chromatography (10 g Sfär Silica D; 35 mL/min; 100% cyclohexane (2 CV), followed by a linear gradient (14 CV): 0%→30% ethyl acetate in cyclohexane).

Yellow solid, m.p.: 82-87 °C; ^{1}H NMR (500 MHz, 300 K, $CDCl_3$): δ = 10.52 (d, *J* = 7.5 Hz, 1H), 8.19 – 8.14 (m, 2H), 7.70 (td, *J* = 8.5, 1.5 Hz, 1H), 7.66 – 7.59 (m, 2H), 7.36 – 7.32 (m, 1H), 7.29 (dd, *J* = 8.0, 7.0 Hz, 1H), 7.20 (s, 1H), 7.16 (dd, *J* = 8.0, 7.0 Hz, 1H), 7.09 (dd, *J* = 8.0, 7.0 Hz, 1H), 5.02 (td, *J* = 7.0, 5.5 Hz, 1H), 4.08 (dd, *J* = 9.5, 7.5 Hz, 1H), 4.01 (dd, *J* = 9.5, 7.5 Hz, 1H), 3.70 (s, 3H), 3.48 – 3.35 (m, 2H), 1.33 – 1.21 (m, 1H), 0.69 – 0.60 (m, 2H), 0.44 – 0.32 ppm (m, 2H); ^{13}C NMR (126 MHz, 300 K, $CDCl_3$): δ = 171.9, 171.3, 170.7, 159.1, 138.7, 136.3, 134.2, 127.5, 125.4, 123.3, 123.0, 122.2, 119.6, 118.8, 115.0, 112.4, 111.3, 110.2, 97.3, 80.8, 53.2, 52.6, 28.0, 9.2, 3.6 ppm; IR (film): $\tilde{\nu}$ = 3407, 3011, 2953, 1746, 1648, 1559, 1409, 1293, 1220, 1012 cm^{-1}; HRMS (ESI): *m/z* calcd for $C_{26}H_{26}N_3O_6$ [*M*+H]$^+$: 476.1816, found: 476.1808.

(±)-(1-(Cyclopropylmethoxy)-4-hydroxy-2-oxo-1,2-dihydroquinoline-3-carbonyl)tryptophan (19)

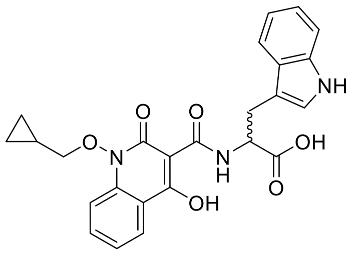

According to General Procedure B, racemic carboxylic acid **19** (19 mg, 37%) was prepared from racemic methyl ester **19a** (50 mg, 0.11 mmol), following reverse-phase column chromatography (12 g Sfär C18 Duo; 12 mL/min; water (+ 0.1%$_{v/v}$ formic acid) (4 CV), followed by a linear gradient (25 CV): 0%→100% acetonitrile (+ 0.1%$_{v/v}$ formic acid) in water (+ 0.1%$_{v/v}$ formic acid)).

White solid, m.p.: 223-227 °C; ^{1}H NMR (500 MHz, 300 K, DMSO-d_6): δ = 10.93 (s, 1H), 10.38 (d, *J* = 7.0 Hz, 1H), 8.09 (d, *J* = 8.0 Hz, 1H), 7.85 (t, *J* = 8.0 Hz, 1H), 7.69 (d, *J* = 8.5 Hz, 1H), 7.50 (d, *J* = 8.0 Hz, 1H), 7.40 (t, *J* = 7.5 Hz, 1H), 7.32 (d, *J* = 8.0 Hz, 1H), 7.18 (d, *J* = 2.0 Hz, 1H), 7.03 (t, *J* = 7.5 Hz, 1H), 6.91 (t, *J* = 7.5 Hz, 1H), 4.81 (q, *J* = 6.0 Hz, 1H), 4.02 – 3.94 (m, 2H), 3.39 – 3.27 (m, 2H; note, that the signal partially overlaps with that of H_2O, perturbing accurate integration), 1.27 – 1.21 (m, 1H), 0.62 – 0.54 (m, 2H), 0.41 – 0.30 ppm (m, 2H); ^{13}C NMR (126 MHz, 300 K, DMSO-d_6): δ = 172.1, 170.5, 169.8, 157.9, 138.2, 136.1, 134.8, 127.3, 124.6, 123.9, 123.2, 120.9, 118.4, 118.2, 114.0, 112.5, 111.4, 108.7, 96.4,

79.9, 53.1, 27.1, 8.8, 3.2 ppm; IR (film): ṽ = 3442, 3213, 3053, 2942, 1728, 1627, 1544, 1411, 1343, 1229, 1012 cm^{-1}; HRMS (ESI): *m/z* calcd for $C_{25}H_{24}O_6N_3$ [*M*+H]$^+$: 462.1660, found: 462.1656.

1-(Cyclopropylmethoxy)-4-hydroxy-2-oxo-1,2-dihydroquinoline-3-carboxamide (20)

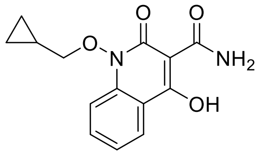

To a solution of reported ethyl 1-(cyclopropylmethoxy)-4-hydroxy-2-oxo-1,2-dihydroquinoline-3-carboxylate (**7**)[23, 24] (303 mg, 1.0 mmol, 1.0 equiv.) in methanol (0.5 mL; HPLC grade) at room temperature under an ambient atmosphere was added aqueous 35%$_{w/w}$ NH_3 solution (5.5 mL, 100 mmol, 100 equiv.). The reaction mixture was stirred for 16 h at room temperature before removal of the solvent under reduced pressure. The crude residue was triturated with methanol (3 × 5 mL) to afford the primary amide **20** (245 mg, 88%).

White solid, m.p. 198-200 °C; ^{1}H NMR (600 MHz, 300 K, DMSO-*d*$_6$): δ = 9.37 (s, 1H), 8.64 (s, 1H), 8.07 (d, *J* = 8.0 Hz, 1H), 7.83 (t, *J* = 8.5 Hz, 1H), 7.67 (d, *J* = 8.5 Hz, 1H), 7.38 (t, *J* = 8.0 Hz, 1H), 4.02 (d, *J* = 7.5 Hz, 2H), 1.30 – 1.22 (m, 1H), 0.61 – 0.55 (m, 2H), 0.46 – 0.28 ppm (m, 2H); ^{13}C NMR (151 MHz, 300 K, DMSO-*d*$_6$): δ = 172.9, 171.9, 158.0, 138.3, 134.7, 124.7, 122.9, 114.4, 112.4, 96.0, 79.8, 8.8, 3.2 ppm; IR (film): ṽ = 3334, 1665, 1633, 1575, 1425, 1289, 1110 cm^{-1}; HRMS (ESI): *m/z* calcd for $C_{14}H_{15}N_2O_4$ [*M*+H]$^+$: 275.1026, found: 275.1023.

Ethyl 3-(1-(cyclopropylmethoxy)-4-hydroxy-2-oxo-1,2-dihydroquinoline-3-carboxamido)-propanoate (21a)

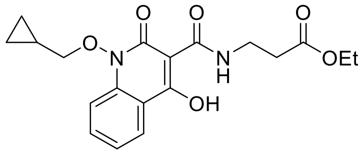

According to General Procedure A, ethyl ester **21a** (80 mg, 93%) was obtained from reported ethyl 1-(cyclopropylmethoxy)-4-hydroxy-2-oxo-1,2-dihydroquinoline-3-carboxylate (**7**)[23, 24] (70 mg, 0.23 mmol) and ethyl 3-aminopropanoate hydrochloride (41 mg, 0.27 mmol), following column chromatography (10 g Sfär Silica D; 35 mL/min; 100% cyclohexane (2 CV), followed by a linear gradient (14 CV): 0%→30% ethyl acetate in cyclohexane).

White solid, m.p. 108-110 °C; ^{1}H NMR (600 MHz, 300 K, $CDCl_3$): δ = 10.26 (t, *J* = 6.0 Hz, 1H), 8.18 (d, *J* = 8.0 Hz, 1H), 7.70 (t, *J* = 8.5 Hz, 1H), 7.65 (d, *J* = 8.5 Hz, 1H), 7.30 (t, *J* = 8.0 Hz, 1H), 4.19 (q, *J* = 7.0 Hz, 2H), 4.07 (d, *J* = 7.5 Hz, 2H), 3.74 (q, *J* = 6.5 Hz, 2H), 2.66 (t, *J* = 6.5 Hz, 2H), 1.31 – 1.26 (m, 4H), 0.75 – 0.59 (m, 2H), 0.48 – 0.34 ppm (m, 2H); ^{13}C NMR (151 MHz, 300 K, $CDCl_3$): δ = 171.6, 171.4, 171.0, 159.2, 138.7, 134.1, 125.4, 123.0, 115.2, 112.4, 97.2, 80.8, 61.0, 34.9, 34.4, 14.3, 9.2, 3.6 ppm; IR (film): ṽ = 2981, 1733, 1631, 1562, 1406, 1188 cm^{-1}; HRMS (ESI): *m/z* calcd for $C_{19}H_{23}O_6N_2$ [*M*+H]$^+$: 375.1551, found: 375.1549.

3-(1-(Cyclopropylmethoxy)-4-hydroxy-2-oxo-1,2-dihydroquinoline-3-carboxamido)propanoic acid (21)[25]

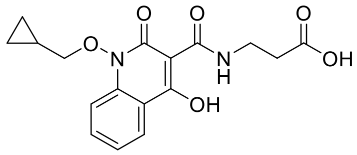

According to General Procedure B, carboxylic acid **21** (76 mg, 65%) was obtained from ethyl ester **21a** (126 mg, 0.34 mmol), following reverse-phase column chromatography (12 g Sfär C18 Duo; 12 mL/min; water (+ 0.1%$_{v/v}$ formic acid) (4 CV), followed by a linear gradient (25 CV): 0%→100% acetonitrile (+ 0.1%$_{v/v}$ formic acid) in water (+ 0.1%$_{v/v}$ formic acid)). The analytical data of carboxylic acid **21** are consistent with those reported.[25]

White solid, m.p.: 175-177 °C; ^{1}H NMR (500 MHz, 300 K, DMSO-d_6): δ = 10.16 (t, *J* = 6.5 Hz, 1H), 8.09 (d, *J* = 8.0 Hz, 1H), 7.85 (t, *J* = 8.5 Hz, 1H), 7.69 (d, *J* = 8.5 Hz, 1H), 7.41 – 7.38 (m, 1H), 4.02 (d, *J* = 7.5 Hz, 2H), 3.58 (q, *J* = 6.5 Hz, 2H), 2.57 (t, *J* = 6.5 Hz, 2H), 1.32 – 1.22 (m, 1H), 0.66 – 0.52 (m, 2H), 0.43 – 0.31 ppm (m, 2H); ^{13}C NMR (126 MHz, 300 K, DMSO-d_6): δ = 172.9, 170.7, 170.2, 158.0, 138.2, 134.7, 124.6, 123.1, 114.2, 112.4, 96.3, 79.9, 34.6, 33.5, 8.8, 3.2 ppm; IR (film): $\tilde{\nu}$ = 3242, 2980, 1737, 1623, 1580, 1545, 1410, 1232 cm^{-1}; HRMS (ESI): *m/z* calcd for $C_{17}H_{19}O_6N_2$ [*M*+H]$^+$: 347.1238, found: 347.1232.

Ethyl *N*-(1-(cyclopropylmethoxy)-4-hydroxy-2-oxo-1,2-dihydroquinoline-3-carbonyl)-*N*-methylglycinate (22a)

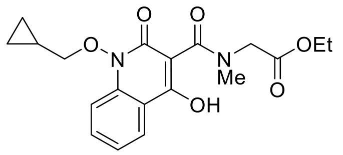

According to General Procedure A, ethyl ester **22a** (50 mg, 57%) was obtained from reported ethyl 1-(cyclopropylmethoxy)-4-hydroxy-2-oxo-1,2-dihydroquinoline-3-carboxylate (**7**)[23, 24] (70 mg, 0.23 mmol) and sarcosine ethyl ester hydrochloride (41 mg, 0.27 mmol), following column chromatography (10 g Sfär Silica D; 35 mL/min; 100% dichloromethane (2 CV), followed by a linear gradient (14 CV): 0%→5% methanol in dichloromethane).

Yellow oil; ^{1}H NMR (600 MHz, 300 K, $CDCl_3$): δ = 8.11 (d, *J* = 8.0 Hz, 1H), 7.71 – 7.62 (m, 2H), 7.31 – 7.26 (m, 1H), 4.33 – 4.17 (m, 4H), 4.05 (d, *J* = 7.5 Hz, 2H), 3.17 (s, 3H), 1.39 – 1.18 (m, 4H), 0.71 – 0.60 (m, 2H), 0.42 – 0.34 ppm (m, 2H); ^{13}C NMR (151 MHz, 300 K, $CDCl_3$): δ = 170.3, 169.1, 166.9, 156.1, 139.2, 133.6, 125.1, 122.7, 114.4, 112.3, 102.8, 80.7, 61.6, 50.5, 38.9, 14.3, 9.2, 3.6 ppm; IR (film): $\tilde{\nu}$ = 2982, 1747, 1654, 1620, 1559, 1497, 1203, 1087, 1030 cm^{-1}; HRMS (ESI): *m/z* calcd for $C_{19}H_{23}O_6N_2$ [*M*+H]$^+$: 375.1551, found: 375.1546.

_N_-(1-(Cyclopropylmethoxy)-4-hydroxy-2-oxo-1,2-dihydroquinoline-3-carbonyl)-_N_-methylglycine (22)

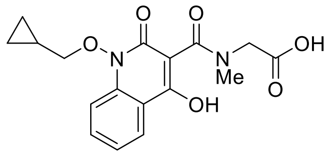

According to General Procedure B, carboxylic acid **22** (25 mg, 60%) was obtained from ethyl ester **22a** (44 mg, 0.12 mmol), following reverse-phase column chromatography (12 g Sfär C18 Duo; 12 mL/min; water (+ 0.1%$_{v/v}$ formic acid) (4 CV), followed by a linear gradient (25 CV): 0%→100% acetonitrile (+ 0.1%$_{v/v}$ formic acid) in water (+ 0.1%$_{v/v}$ formic acid)).

White solid, m.p. 95-97 °C; ^{1}H NMR (600 MHz, 300 K, DMSO-d_6): δ = 8.03 (d, *J* = 8.0 Hz, 1H), 7.73 (t, *J* = 8.5 Hz, 1H), 7.65 (d, *J* = 8.5 Hz, 1H), 7.32 (m, 1H), 4.16 (s, 2H), 3.99 (d, *J* = 7.5 Hz, 2H), 2.96 (s, 3H), 1.27 – 1.21 (m, 1H), 0.66 – 0.51 (m, 2H), 0.45 – 0.26 ppm (m, 2H); ^{13}C NMR (151 MHz, 300 K, DMSO-d_6): δ = 170.6, 165.2, 157.5, 155.6, 137.8, 132.3, 123.9, 122.2, 114.3, 111.9, 108.3, 79.5, 48.4, 36.4, 8.8, 3.1 ppm; IR (film): $\tilde{\nu}$ = 2981, 1730, 1618, 1500, 1404, 1250, 1088, 1026 cm^{-1}; HRMS (ESI): *m/z* calcd for $C_{17}H_{19}O_6N_2$ [*M*+H]$^+$: 347.1238, found: 347.1241.

Ethyl (1-(cyclopropylmethoxy)-4-hydroxy-2-oxo-1,2-dihydroquinoline-3-carbonyl)glycinate (24)[25]

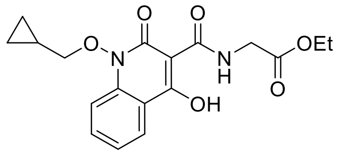

According to General Procedure A, ethyl ester **24** (214 mg, 86%) was obtained from reported ethyl 1-(cyclopropylmethoxy)-4-hydroxy-2-oxo-1,2-dihydroquinoline-3-carboxylate (**7**)[23, 24] (210 mg, 0.69 mmol) and glycine ethyl ester hydrochloride (113 mg, 0.81 mmol), following column chromatography (25 g Sfär Silica D; 60 mL/min; 100% cyclohexane (2 CV), followed by a linear gradient (14 CV): 0%→20% acetone in cyclohexane). The analytical data of ethyl ester **24** are consistent with those reported.[25]

White solid, m.p.: 112-114 °C; ^{1}H NMR (600 MHz, 300 K, $CDCl_3$): δ = 10.45 (t, *J* = 5.5 Hz, 1H), 8.18 (dd, *J* = 8.0, 1.5 Hz, 1H), 7.71 (td, *J* = 8.0, 1.5 Hz, 1H), 7.66 (dd, *J* = 8.5, 1.0 Hz, 1H), 7.31 (td, *J* = 8.0, 1.0 Hz, 1H), 4.26 (q, *J* = 7.0 Hz, 2H), 4.21 (d, *J* = 5.5 Hz, 2H), 4.09 (d, *J* = 7.5 Hz, 2H), 1.33 – 1.29 (m, 4H), 0.70 – 0.62 (m, 2H), 0.44 – 0.36 ppm (m, 2H); ^{13}C NMR (151 MHz, 300 K, $CDCl_3$): δ = 171.2, 171.1(6), 169.2, 159.2, 138.8, 134.3, 125.4, 123.1, 115.0, 112.5, 97.3, 80.8, 61.7, 41.3, 14.3, 9.2, 3.6 ppm; IR (film): $\tilde{\nu}$ = 2981, 1745, 1654, 1627, 1547, 1403, 1292, 1219, 1026 cm^{-1}; HRMS (ESI): *m/z* calcd for $C_{18}H_{21}O_6N_2$ [*M*+H]$^+$: 361.1394, found: 361.1388.

N-(2-Amino-2-oxoethyl)-1-(cyclopropylmethoxy)-4-hydroxy-2-oxo-1,2-dihydroquinoline-3-carboxamide (25)

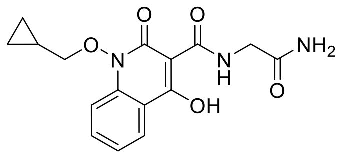

To a solution of methyl ester **23** (30 mg, 0.09 mmol, 1.0 equiv.) in methanol (0.5 mL; HPLC grade) was added a solution of NH_3 (7N in methanol; 1.3 mL, 9.0 mmol, 100 equiv.) at room temperature under an ambient atmosphere. The reaction mixture was stirred for 16 h, before removal of the solvent under reduced pressure. The crude residue was triturated with methanol (3 × 5 mL) to afford the primary amide **25** (26 mg, 87%).

White solid, m.p. >250 °C (decomposition); ^{1}H NMR (600 MHz, 300 K, DMSO-d_6): δ = 10.26 (t, *J* = 5.0 Hz, 1H), 8.09 (d, *J* = 8.0 Hz, 1H), 7.86 (t, *J* = 8.5 Hz, 1H), 7.70 (d, *J* = 8.5 Hz, 1H), 7.57 (s, 1H), 7.40 (t, *J* = 8.0 Hz, 1H), 7.22 (s, 1H), 4.04 (d, *J* = 7.5 Hz, 2H), 4.01 (d, *J* = 5.0 Hz, 2H), 1.31 – 1.25 (m, 1H), 0.65 – 0.54 (m, 2H), 0.43 – 0.33 ppm (m, 2H); ^{13}C NMR (151 MHz, 300 K, DMSO-d_6): δ = 170.5, 170.1, 169.3, 157.8, 138.2, 134.7, 124.6, 123.1, 114.0, 112.4, 96.6, 79.9, 41.9, 8.8, 3.2 ppm; IR (film): $\tilde{\nu}$ = 3413, 3205, 2981, 1674, 1637, 1565, 1406, 1302, 1152, 1032 cm^{-1}; HRMS (ESI): *m/z* calcd for $C_{16}H_{18}O_5N_3$ [*M*+H]$^+$: 322.1241, found: 322.1242.

1-(Cyclopropylmethoxy)-4-hydroxy-N-(2-(methylamino)-2-oxoethyl)-2-oxo-1,2-dihydroquinoline-3-carboxamide (26)

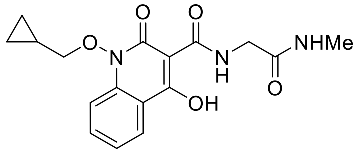

To a solution of Desidustat[23, 24] (30 mg, 0.09 mmol, 1.0 equiv.) and methylamine hydrochloride (7 mg, 0.11 mmol, 1.2 equiv.) in anhydrous *N*,*N*-dimethylformamide (0.5 mL) under a N_2 atmosphere at 0 °C were added redistilled anhydrous *N*,*N*-diisopropylethylamine (63 µL, 0.36 mmol, 4.0 equiv.) and HATU[15] (44 mg, 0.12 mmol, 1.3 equiv.). The reaction mixture was allowed to warm to ambient temperature and stirred for 14 h, before removal of the solvent under reduced pressure (water bath temperature: 50 °C). The crude residue was redissolved in ethyl acetate and was sequentially washed with aqueous HCl solution (1M), saturated aqueous $NaHCO_3$ solution and brine. The organic layer was dried over anhydrous Na_2SO_4, filtered, and evaporated. The crude residue was purified using column chromatography (10 g Sfär Silica D; 35 mL/min; 100% dichloromethane (2 CV), followed by a linear gradient (14 CV): 0%→10% methanol in dichloromethane) to afford the amide **26** (20 mg, 64%).

White solid, m.p. 210-214 °C; ^{1}H NMR (600 MHz, 300 K, DMSO-d_6): δ = 10.28 (t, *J* = 5.5 Hz, 1H), 8.10 (d, *J* = 8.0 Hz, 1H), 8.02 (br s, 1H), 7.86 (t, *J* = 8.0 Hz, 1H), 7.71 (d, *J* = 8.5 Hz, 1H), 7.40 (t, *J* = 8.0 Hz, 1H), 4.05 - 4.00 (m, 4H), 2.64 (d, *J* = 4.5 Hz, 3H), 1.32 – 1.25 (m, 1H), 0.63 – 0.54 (m, 2H), 0.42 – 0.35 ppm

(m, 2H); ^{13}C NMR (151 MHz, 300 K, DMSO-d_6): δ = 170.4, 170.2, 167.8, 157.9, 138.2, 134.7, 124.6, 123.1, 114.0, 112.4, 96.7, 79.9, 42.1, 25.5, 8.8, 3.2 ppm; IR (film): $\tilde{\nu}$ = 3319, 2981, 1653, 1560, 1413, 1238, 1025 cm^{-1}; HRMS (ESI): *m/z* calcd for $C_{17}H_{20}O_5N_3$ [*M*+H]$^+$: 346.1397, found: 346.1399;

N-(Cyanomethyl)-1-(cyclopropylmethoxy)-4-hydroxy-2-oxo-1,2-dihydroquinoline-3-carboxamide (27)[25]

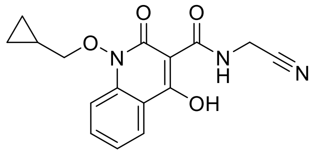

According to General Procedure A, nitrile **27** (56 mg, 78%) was obtained from reported ethyl 1-(cyclopropylmethoxy)-4-hydroxy-2-oxo-1,2-dihydroquinoline-3-carboxylate (**7**)[23, 24] (70 mg, 0.23 mmol) and aminoacetonitrile hydrochloride (25 mg, 0.27 mmol), following column chromatography (10 g Sfär Silica D; 35 mL/min; 100% cyclohexane (2 CV), followed by a linear gradient (14 CV): 0%→20% acetone in cyclohexane). The analytical data of nitrile **27** are consistent with those reported.[25]

White solid, m.p. 164-166 °C; ^1H NMR (600 MHz, 300 K, $CDCl_3$): δ = 10.53 (t, *J* = 6.0 Hz, 1H), 8.20 (d, *J* = 8.0 Hz, 1H), 7.75 (t, *J* = 8.0 Hz, 1H), 7.68 (d, *J* = 8.0 Hz, 1H), 7.35 (t, *J* = 8.0 Hz, 1H), 4.35 (d, *J* = 6.0 Hz, 2H), 4.09 (d, *J* = 7.5 Hz, 2H), 1.33 – 1.27 (m, 1H), 0.74 – 0.61 (m, 2H), 0.50 – 0.29 ppm (m, 2H); ^{13}C NMR (151 MHz, 300 K, $CDCl_3$): δ = 171.3, 171.2, 159.1, 138.9, 134.8, 125.6, 123.4, 115.6, 114.7, 112.7, 97.1, 81.0, 27.1, 9.2, 3.6 ppm; IR (film): $\tilde{\nu}$ = 3232, 1650, 1627, 1540, 1402, 1340, 1029 cm^{-1}; HRMS (ESI): *m/z* calcd for $C_{16}H_{16}N_3O_4$ [*M*+H]$^+$: 314.1135, found: 314.1142.

(±)-1-(Cyclopropylmethoxy)-4-hydroxy-2-oxo-N-(3,3,3-trifluoro-2-hydroxypropyl)-1,2-dihydroquinoline-3-carboxamide (28)

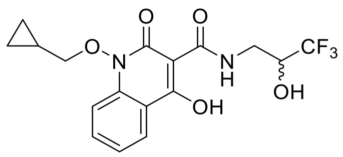

According to General Procedure A, racemic alcohol **28** (20 mg, 40%) was obtained from reported ethyl 1-(cyclopropylmethoxy)-4-hydroxy-2-oxo-1,2-dihydroquinoline-3-carboxylate (**7**)[23, 24] (40 mg, 0.13 mmol) and (±)-3-amino-1,1,1-trifluoropropan-2-ol (20 mg, 0.15 mmol), following column chromatography (10 g Sfär Silica D; 35 mL/min; 100% cyclohexane (2 CV), followed by a linear gradient (14 CV): 0%→30% acetone in cyclohexane).

White solid, m.p. 125-127 °C; ^1H NMR (600 MHz, 300 K, $CDCl_3$): δ = 10.42 (br s, 1H), 8.08 (d, *J* = 8.0 Hz, 1H), 7.76 – 7.73 (m, 1H), 7.66 (d, *J* = 8.5 Hz, 1H), 7.33 – 7.29 (m, 1H), 4.41 – 4.26 (m, 2H), 4.12 – 4.06 (m, 1H), 4.04 – 3.98 (t, *J* = 8.5 Hz, 1H), 3.91 – 3.86 (m, 1H), 3.69 – 3.64 (m, 1H), 1.34 – 1.27 (m, 1H), 0.69 – 0.63 (m, 2H), 0.41 – 0.35 ppm (m, 2H); ^{13}C NMR (151 MHz, 300 K, $CDCl_3$): δ = 172.4, 171.1, 159.2, 138.6, 134.6, 125.5, 124.4 (q, *J* = 282.0 Hz), 123.4, 114.8, 112.6, 96.9, 81.0, 70.2 (q, *J* = 30.5 Hz), 40.2,

9.1, 3.7 ppm; ^{19}F NMR (565 MHz, 300 K, $CDCl_3$): δ = –78.5 ppm; IR (film): $\tilde{\nu}$ = 2981, 1630, 1560, 1405, 1171, 1139 cm^{-1}; HRMS (ESI): *m/z* calcd for $C_{17}H_{18}F_3N_2O_5$ [*M*+H]$^+$: 387.1162, found: 387.1171.

1-(Cyclopropylmethoxy)-4-hydroxy-*N*-((3-hydroxyoxetan-3-yl)methyl)-2-oxo-1,2-dihydroquinoline-3-carboxamide (29)

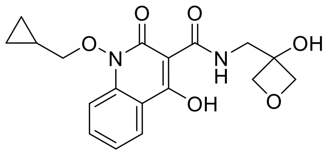

According to General Procedure A, alcohol **29** (42 mg, 90%) was obtained from reported ethyl 1-(cyclopropylmethoxy)-4-hydroxy-2-oxo-1,2-dihydroquinoline-3-carboxylate (**7**)[23, 24] (40 mg, 0.13 mmol) and 3-(aminomethyl)oxetan-3-ol (20 mg, 0.15 mmol), following column chromatography (10 g Sfär Silica D; 35 mL/min; 100% cyclohexane (2 CV), followed by a linear gradient (14 CV): 0%→30% acetone in cyclohexane).

White solid, m.p. 143-145 °C; ^{1}H NMR (700 MHz, 300 K, $CDCl_3$): δ = 10.58 (br s, 1H), 8.21 – 8.16 (m, 1H), 7.76 – 7.72 (m, 1H), 7.67 (d, *J* = 8.5 Hz, 1H), 7.37 – 7.29 (m, 1H), 4.65 (d, *J* = 7.0 Hz, 2H), 4.52 (d, *J* = 7.5 Hz, 2H), 4.43 (s, 1H), 4.08 (d, *J* = 7.5 Hz, 2H), 3.93 (d, *J* = 6.0 Hz, 2H), 1.34 – 1.28 (m, 1H), 0.72 – 0.63 (m, 2H), 0.43 – 0.38 ppm (m, 2H); ^{13}C NMR (176 MHz, 300 K, $CDCl_3$): δ = 172.9, 171.1, 159.2, 138.7, 134.6, 125.5, 123.3, 114.9, 112.6, 97.1, 82.0, 81.0, 74.8, 46.5, 9.2, 3.6 ppm; IR (film): $\tilde{\nu}$ = 2981, 1627, 1558, 1408, 1188 cm^{-1}; HRMS (ESI): *m/z* calcd for $C_{18}H_{21}N_2O_6$ [*M*+H]$^+$: 361.1394, found: 361.1387.

1-(Cyclopropylmethoxy)-4-hydroxy-*N*-(hydroxymethyl)-2-oxo-1,2-dihydroquinoline-3-carboxamide (30a)

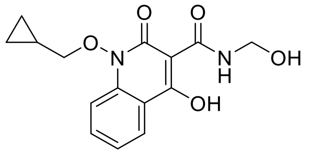

To a mixture of amide **25** (230 mg, 0.93 mmol, 1.0 equiv.) and K_2CO_3 (129 mg, 0.93 mmol, 1.0 equiv.) in tetrahydrofuran (4.5 mL; HPLC grade) and water (1.0 mL; Milli-Q® Ultrapure grade) was added aqueous 37%$_{w/w}$ formaldehyde solution (0.10 mL, 1.4 mmol, 1.5 equiv.) under an ambient atmosphere at room temperature. The reaction mixture was heated under reflux for 24 h before it was cooled to ambient temperature and concentrated under reduced pressure. The crude residue was then redissolved in ethyl acetate and washed with H_2O, then brine. The organic solution was dried over anhydrous Na_2SO_4, filtered and evaporated to afford hemiaminal **30a** (244 mg, 95%), which was used in the subsequent reaction without further purification.

White solid, m.p. 217-220 °C; ^{1}H NMR (400 MHz, 300 K, DMSO-d_6): δ = 10.24 (s, 1H), 7.91 (d, *J* = 8.0 Hz, 1H), 7.68 (t, *J* = 8.0 Hz, 1H), 7.52 (d, *J* = 8.5 Hz, 1H), 7.22 (t, *J* = 7.5 Hz, 1H), 5.86 – 5.77 (m, 1H), 4.69 – 4.58 (m, 2H), 3.85 (d, *J* = 7.5 Hz, 2H), 1.13 – 1.05 (m, 1H), 0.47 – 0.35 (m, 2H), 0.24 – 0.16 ppm (m, 2H); ^{13}C NMR (101 MHz, 300 K, DMSO-d_6): δ = 171.1, 170.5, 158.0, 138.3, 134.8, 124.6, 123.1, 114.0,

112.4, 96.3, 79.9, 62.5, 8.8, 3.2 ppm; IR (film): $\tilde{\nu}$ = 3231, 2923, 1652, 1623, 1546, 1402, 1031 cm^{-1}; HRMS (ESI): *m/z* calcd for $C_{15}H_{17}N_2O_5$ [*M*+H]$^+$: 305.1137, found: 305.1132.

N-((1H-1,2,3-Triazol-1-yl)methyl)-1-(cyclopropylmethoxy)-4-hydroxy-2-oxo-1,2-dihydroquinoline-3-carboxamide (30)

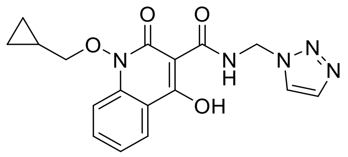

To a solution of hemiaminal **30a** (107 mg, 0.35 mmol, 1.0 equiv.) in anhydrous dichloromethane (2 mL) was added dropwise thionyl chloride (75 µL, 1.1 mmol, 3.0 equiv.) under a N_2 atmosphere at 0 °C. The reaction mixture was stirred at room temperature for 2 h before removal of the solvent under reduced pressure. The crude residue was redissolved in dichloromethane (2 mL; HPLC grade) and 1*H*-1,2,3-triazole (60 mg, 0.88 mmol, 2.5 equiv.) was added under an ambient atmosphere at room temperature. The reaction mixture was stirred at room temperature for 1 h before removal of the solvent under reduced pressure. The crude residue was purified by reverse-phase column chromatography (12 g Sfär C18 Duo; 12 mL/min; water (+ 0.1%$_{v/v}$ formic acid) (4 CV), followed by a linear gradient (25 CV): 0%→100% acetonitrile (+ 0.1%$_{v/v}$ formic acid) in water (+ 0.1%$_{v/v}$ formic acid)) to afford triazole **30** (30 mg, 24%).

White solid, m.p.: 169-171 °C; ^{1}H NMR (600 MHz, 300 K, DMSO-d_6): δ = 10.88 (t, *J* = 7.0 Hz, 1H), 8.23 (s, 1H), 8.10 (d, *J* = 8.0 Hz, 1H), 7.87 (t, *J* = 8.5 Hz, 1H), 7.75 (s, 1H), 7.70 (d, *J* = 8.5 Hz, 1H), 7.41 (t, *J* = 7.5 Hz, 1H), 6.00 (d, *J* = 6.5 Hz, 2H), 4.03 (d, *J* = 7.5 Hz, 2H), 1.30 – 1.24 (m, 1H), 0.63 – 0.51 (m, 2H), 0.43 – 0.32 ppm (m, 2H); ^{13}C NMR (151 MHz, 300 K, DMSO-d_6): δ = 171.2, 170.7, 157.8, 138.4, 135.2, 133.4, 125.1, 124.7, 123.3, 113.7, 112.5, 96.5, 80.0, 52.1, 8.7, 3.2 ppm; IR (film): $\tilde{\nu}$ = 3203, 2981, 1636, 1545, 1381, 1028 cm^{-1}; HRMS (ESI): *m/z* calcd for $C_{17}H_{18}N_5O_4$ [*M*+H]$^+$: 356.1353, found: 356.1347.

N-((1H-1,2,4-Triazol-1-yl)methyl)-1-(cyclopropylmethoxy)-4-hydroxy-2-oxo-1,2-dihydroquinoline-3-carboxamide (31)

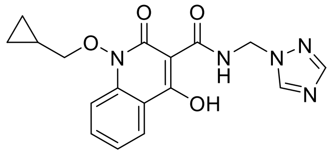

To a solution of hemiaminal **30a** (107 mg, 0.35 mmol, 1.0 equiv.) in anhydrous dichloromethane (2 mL) was added dropwise thionyl chloride (75 µL, 1.1 mmol, 3.0 equiv.) under a N_2 atmosphere at 0 °C. The reaction mixture was stirred at room temperature for 2 h before removal of the solvent under reduced pressure. The crude residue was redissolved in dichloromethane (2 mL; HPLC grade) and 1*H*-1,2,4-triazole (60 mg, 0.88 mmol, 2.5 equiv.) was added under an ambient atmosphere at room temperature. The reaction mixture was stirred at room temperature for 1 h before removal of the solvent under reduced pressure. The crude residue was purified by reverse-phase column chromatography (12 g Sfär C18 Duo; 12 mL/min; water (+ 0.1%$_{v/v}$ formic acid) (4 CV),

followed by a linear gradient (25 CV): 0%→100% acetonitrile (+ 0.1%$_{v/v}$ formic acid) in water (+ 0.1%$_{v/v}$ formic acid)) to afford triazole **31** (50 mg, 40%).

White solid, m.p.: 125-127 °C; ^{1}H NMR (600 MHz, 300 K, DMSO-d_6): δ = 10.79 (br s, 1H), 8.65 (s, 1H), 8.09 (d, *J* = 8.0 Hz, 1H), 8.01 (s, 1H), 7.86 (t, *J* = 8.0 Hz, 1H), 7.69 (d, *J* = 8.5 Hz, 1H), 7.40 (t, *J* = 7.5 Hz, 1H), 5.80 (d, *J* = 6.5 Hz, 2H), 4.02 (d, *J* = 7.5 Hz, 2H), 1.30 – 1.24 (m, 1H), 0.68 – 0.51 (m, 2H), 0.44 – 0.28 ppm (m, 2H); ^{13}C NMR (151 MHz, 300 K, DMSO-d_6): δ = 171.1, 170.7, 157.8, 151.7, 144.8, 138.4, 135.1, 124.7, 123.2, 113.8, 112.5, 96.5, 80.0, 51.6, 8.8, 3.2 ppm; IR (film): $\tilde{\nu}$ = 3223, 3126, 2981, 1654, 1626, 1518, 1398, 1338, 1171, 1037 cm^{-1}; HRMS (ESI): *m/z* calcd for $C_{17}H_{18}N_5O_4$ [*M*+H]$^+$: 356.1353, found: 356.1352.

_N_-((2_H_-Tetrazol-5-yl)methyl)-1-(cyclopropylmethoxy)-4-hydroxy-2-oxo-1,2-dihydroquinoline-3-carboxamide (32)

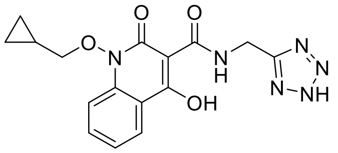

A mixture of reported ethyl 1-(cyclopropylmethoxy)-4-hydroxy-2-oxo-1,2-dihydroquinoline-3-carboxylate (**7**)[23, 24] (70 mg, 0.23 mmol, 1.0 equiv.), (2*H*-tetrazol-5-yl)methanamine (26 mg, 0.26 mmol, 1.15 equiv.) and anhydrous triethylamine (64 µL, 0.46 mmol, 2.0 equiv.) in anhydrous dioxane (1.3 mL) in a sealed 10 mL microwave reaction vial was heated in a 120 °C sand bath under a N_2 atmosphere for 14 h. The reaction mixture was then cooled to ambient temperature and the solvent was removed under reduced pressure. The crude residue was purified by reverse-phase column chromatography (12 g Sfär C18 Duo; 12 mL/min; water (+ 0.1%$_{v/v}$ formic acid) (4 CV), followed by a linear gradient (25 CV): 0%→100% acetonitrile (+ 0.1%$_{v/v}$ formic acid) in water (+ 0.1%$_{v/v}$ formic acid)) to afford tetrazole **32** (45 mg, 0.13 mmol, 55%).

White solid, m.p. 166-170 °C, ^{1}H NMR (600 MHz, 300 K, DMSO-d_6): δ = 10.54 (t, *J* = 6.0 Hz, 1H), 8.11 (dd, *J* = 8.0, 1.0 Hz, 1H), 7.88 (td, *J* = 8.5, 1.5 Hz, 1H), 7.73 (d, *J* = 8.0 Hz, 1H), 7.42 (dd, *J* = 8.0, 1.0 Hz, 1H), 4.92 (d, *J* = 6.0 Hz, 2H), 4.06 (d, *J* = 7.5 Hz, 2H), 1.35 – 1.22 (m, 1H), 0.65 – 0.56 (m, 2H), 0.45 – 0.34 ppm (m, 2H); ^{13}C NMR (151 MHz, 300 K, DMSO-d_6): δ = 170.9, 170.3, 157.9, 154.3 (br), 138.3, 135.0, 124.6, 123.3, 113.8, 112.5, 96.8, 80.0, 33.1, 8.8, 3.2 ppm; IR (film): $\tilde{\nu}$ = 3256, 3014, 2898, 1627, 1556, 1414, 1345, 1233, 1030 cm^{-1}; HRMS (ESI): *m/z* calcd for $C_{16}H_{17}O_4N_6$ [*M*+H]$^+$: 357.1306, found: 357.1302.

Ethyl 2-((benzyloxy)(_tert_-butoxycarbonyl)amino)benzoate (34)

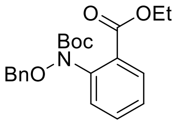

A mixture of commercially-sourced ethyl iodobenzene **33** (2.21 g, 8.0 mmol, 1.0 equiv.), *tert*-butyl *N*-(benzyloxy)carbamate (1.79 g, 8.0 mmol, 1.0 equiv.), copper iodide (152 mg, 0.80 mmol, 0.1 equiv.), glycine (391 mg, 5.2 mmol, 0.65 equiv.) and

K_2CO_3 (8.86 g, 64 mmol, 8.0 equiv.) in anhydrous toluene (20 mL) and anhydrous ethanol (6 mL) was heated under reflux for 16 h under a N_2 atmosphere. The reaction mixture was then cooled to room temperature and the solvent was removed under reduced pressure. The crude residue was redissolved in ethyl acetate and washed with H_2O, then brine. The organic layer was dried over anhydrous Na_2SO_4, filtered, evaporated and purified by column chromatography (100 g Sfär Silica D; 120 mL/min; 100% cyclohexane (2 CV), followed by a linear gradient (14 CV): 0%→10% ethyl acetate in cyclohexane) to afford *N*-Boc protected aniline **34** (1.47 g, 56%).

Colorless oil; ^1H NMR (600 MHz, 300 K, $CDCl_3$): δ = 7.80 (dd, *J* = 8.0, 1.5 Hz, 1H), 7.46 (td, *J* = 8.0, 1.5 Hz, 1H), 7.41 – 7.26 (m, 7H), 4.95 (s, 2H), 4.33 (q, *J* = 7.0 Hz, 2H), 1.47 (s, 9H), 1.36 ppm (t, *J* = 7.0 Hz, 3H); ^{13}C NMR (151 MHz, 300 K, $CDCl_3$): δ = 166.8, 153.9, 139.7, 135.5, 131.9, 130.3, 129.5, 128.6, 128.4, 128.1, 127.0, 126.6, 82.2, 77.4, 61.4, 28.3, 14.3 ppm; IR (film): $\tilde{\nu}$ = 2981, 1723, 1454, 1368, 1256, 1166, 1105 cm^{-1}; HRMS (ESI): *m/z* calcd for $C_{21}H_{25}NO_5Na$ $[M+Na]^+$: 394.1625, found: 394.1631.

Ethyl 2-((benzyloxy)amino)benzoate (35)[25]

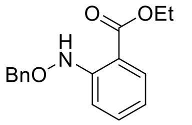

To *N*-Boc protected aniline **34** (1.68 g, 4.5 mmol) under an atmosphere of Ar gas at 0 °C was added HCl in dioxane (4M; 23 mL). The reaction mixture was stirred at ambient temperature for 1 h before removal of the solvent under reduced pressure. The crude residue was purified using column chromatography (25 g Sfär Silica D; 60 mL/min; 100% cyclohexane (2 CV), followed by a linear gradient (14 CV): 0%→10% ethyl acetate in cyclohexane) to afford aniline **35** (815 mg, 66%). The analytical data of aniline **35** are consistent with those reported.[25]

Colorless oil; ^1H NMR (600 MHz, 300 K, $CDCl_3$): δ = 7.93 (d, *J* = 8.0 Hz, 1H), 7.47 – 7.44 (m, 3H), 7.43 – 7.38 (m, 2H), 7.39 – 7.35 (m, 1H), 7.35 – 7.31 (m, 1H), 6.86 (t, *J* = 8.0 Hz, 1H), 4.96 (s, 2H), 4.31 (q, *J* = 7.0 Hz, 2H), 1.36 ppm (t, *J* = 7.0 Hz, 3H); ^{13}C NMR (151 MHz, 300 K, $CDCl_3$): δ = 167.5, 151.4, 136.5, 134.4, 131.1, 128.9, 128.7, 128.5, 119.3, 114.4, 112.3, 77.7, 60.9, 14.4 ppm; IR (film): $\tilde{\nu}$ = 2981, 1688, 1485, 1255, 1090 cm^{-1}; HRMS (ESI): *m/z* calcd for $C_{16}H_{17}NO_3Na$ $[M+Na]^+$: 294.1101, found: 294.1100.

Ethyl 2-(*N*-(benzyloxy)-3-ethoxy-3-oxopropanamido)benzoate (36)[25]

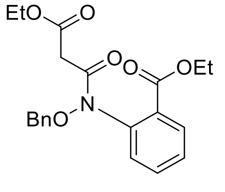

To a solution of aniline **35** (815 mg, 3.0 mmol, 1.0 equiv.) in anhydrous ethyl acetate (12 mL) were sequentially added pyridine (0.49 mL, 6.0 mmol, 2.0 equiv.) and ethyl malonyl chloride (0.46 mL, 3.6 mmol, 1.5 equiv.) under an a N_2 atmosphere at 0 °C. The reaction mixture was stirred at ambient temperature for 14 h before being diluted with ethyl acetate (30 mL) and washed with H_2O, then brine. The organic layer was dried over anhydrous Na_2SO_4, filtered, evaporated, and purified by column chromatography (25 g Sfär Silica D; 60 mL/min; 100% cyclohexane (2 CV), followed by a linear gradient (20 CV):

0%→20% acetone in cyclohexane) to afford malonate **36** (858 mg, 74%). The analytical data of malonate **36** are consistent with those reported.[25]

Colorless oil; ^{1}H NMR (600 MHz, 300 K, $CDCl_3$): δ = 7.87 (d, *J* = 8.0 Hz, 1H), 7.60 – 7.46 (m, 2H), 7.44 – 7.27 (m, 6H), 4.90 (s, 2H), 4.33 (q, *J* = 7.0 Hz, 2H), 4.19 (q, *J* = 7.0 Hz, 2H), 3.64 (s, 2H), 1.35 (t, *J* = 7.0 Hz, 3H), 1.25 ppm (t, *J* = 7.0 Hz, 3H); ^{13}C NMR (151 MHz, 300 K, $CDCl_3$): δ = 167.2, 166.5, 166.2, 137.0, 134.1, 132.3, 130.5, 129.4 (2C), 129.1, 128.7, 128.1, 126.6, 77.1, 61.5, 61.4(7), 41.4, 14.3, 14.2 ppm; IR (film): $\tilde{\nu}$ = 2981, 1727, 1687, 1454, 1369, 1293, 1257, 1151, 1097, 1032 cm^{-1}; HRMS (ESI): *m/z* calcd for $C_{21}H_{24}NO_6$ [*M*+H]$^+$: 386.1598, found: 386.1607.

Ethyl 1-(benzyloxy)-4-hydroxy-2-oxo-1,2-dihydroquinoline-3-carboxylate (37)[25]

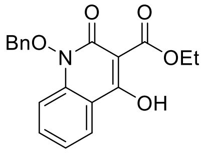

To malonate **36** (433 mg, 1.1 mmol, 1.0 equiv.) was added sodium ethoxide (21%$_{w/w}$ in ethanol; 0.92 mL, 2.5 mmol, 2.2 equiv.) under a N_2 atmosphere at 0 °C. The reaction mixture was stirred at ambient temperature for 1 h, before the pH was neutralized (pH 6-7) with the dropwise addition of aqueous HCl solution (4M). The solvent was removed under reduced pressure and the crude residue was redissolved in ethyl acetate and washed with H_2O, then brine. The organic layer was dried over anhydrous Na_2SO_4, filtered, evaporated, and purified by column chromatography (25 g Sfär Silica D; 60 mL/min; 100% cyclohexane (2 CV), followed by a linear gradient (20 CV): 0%→30% acetone in cyclohexane) to afford ethyl ester **37** (275 mg, 72%). The analytical data of ethyl ester **37** are consistent with those reported.[25]

White solid, m.p. 160-165 °C; ^{1}H NMR (600 MHz, 300 K, $CDCl_3$): δ = 14.28 (s, 1H), 8.15 (d, *J* = 8.0 Hz, 1H), 7.65 (t, *J* = 8.0 Hz, 1H), 7.63 – 7.58 (m, 2H), 7.51 (d, *J* = 8.5 Hz, 1H), 7.43 – 7.34 (m, 3H), 7.27 – 7.22 (m, 1H), 5.23 (s, 2H), 4.55 (q, *J* = 7.0 Hz, 2H), 1.50 ppm (t, *J* = 7.0 Hz, 3H); ^{13}C NMR (151 MHz, 300 K, $CDCl_3$): δ = 172.6, 171.4, 156.0, 139.9, 134.8, 134.1, 130.0, 129.3, 128.8, 125.6, 122.7, 113.7, 112.3, 98.6, 77.4, 62.7, 14.4 ppm; IR (film): $\tilde{\nu}$ = 2981, 1672, 1621, 1559, 1415, 1328, 1268, 1097, 1021 cm^{-1}; HRMS (ESI): *m/z* calcd for $C_{19}H_{18}NO_5$ [*M*+H]$^+$: 340.1180, found: 340.1180.

***tert*-Butyl (1-(benzyloxy)-4-hydroxy-2-oxo-1,2-dihydroquinoline-3-carbonyl)glycinate (38)**

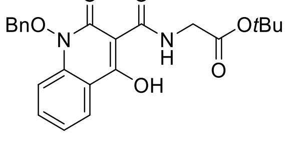

According to General Procedure A, ester **38** (270 mg, 82%) was obtained from ethyl ester **37** (265 mg, 0.78 mmol) and glycine *tert*-butyl ester hydrochloride (151 mg, 0.90 mmol), following column chromatography (10 g Sfär Silica D; 35 mL/min; 100% cyclohexane (2 CV), followed by a linear gradient (14 CV): 0%→20% acetone in cyclohexane).

White solid; m.p.: 175-177 °C; ^{1}H NMR (600 MHz, 300 K, $CDCl_3$): δ = 10.47 (t, *J* = 5.5 Hz, 1H), 8.18 (d, *J* = 8.0 Hz, 1H), 7.66 (t, *J* = 8.5 Hz, 1H), 7.62 – 7.57 (m, 2H), 7.55 (d, *J* = 8.5 Hz, 1H), 7.46 – 7.37 (m, 3H),

7.29 (t, J = 8.0 Hz, 1H), 5.24 (s, 2H), 4.15 (d, J = 5.5 Hz, 2H), 1.52 ppm (s, 9H); ^{13}C NMR (151 MHz, 300 K, $CDCl_3$): δ = 171.3, 171.0, 168.3, 159.2, 138.5, 134.2, 133.9, 129.9, 129.4, 128.9, 125.5, 123.1, 115.0, 112.4, 97.4, 82.5, 77.8, 42.0, 28.2 ppm; IR (film): $\tilde{\nu}$ = 2981, 1742, 1654, 1560, 1411, 1235, 1155 cm^{-1}; HRMS (ESI): *m/z* calcd for $C_{23}H_{25}N_2O_6$ [*M*+H]$^+$: 425.1707, found: 425.1718.

***tert*-Butyl (1,4-dihydroxy-2-oxo-1,2-dihydroquinoline-3-carbonyl)glycinate (39)**

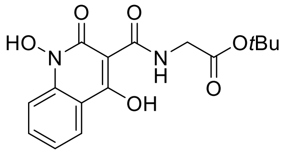

N_2 gas was bubbled through a solution of ester **38** (253 mg, 0.59 mmol) in anhydrous methanol (2 mL) for 15 min. Palladium on charcoal (10%$_{w/w}$ palladium) was added to the solution and H_2 gas was bubbled through the black suspension for 15 min. The reaction mixture was then stirred for 1 h at ambient temperature under an atmosphere of H_2 (1 atm) before being filtered through Celite®, washed with methanol, and evaporated. The crude residue was purified by column chromatography (10 g Sfär Silica D; 35 mL/min; 100% cyclohexane (2 CV), followed by a linear gradient (20 CV): 0%→30% acetone in cyclohexane) to afford *tert*-butyl ester **39** (68 mg, 34%).

Pink solid, m.p.: 156-158 °C; ^{1}H NMR (600 MHz, 300 K, $CDCl_3$): δ = 10.08 (t, J = 5.0 Hz, 1H), 8.23 – 8.16 (m, 1H), 7.78 – 7.71 (m, 2H), 7.36 – 7.31 (m, 1H), 4.13 (d, J = 5.0 Hz, 2H), 1.63 – 1.42 ppm (s, 9H); ^{13}C NMR (151 MHz, 300 K, $CDCl_3$): δ = 170.1, 169.9, 168.3, 157.1, 135.5, 134.4, 125.1, 123.1, 114.2, 112.7, 96.0, 82.7, 42.1, 28.2 ppm; IR (film): $\tilde{\nu}$ = 2981, 1742, 1627, 1560, 1238, 1156 cm^{-1}; HRMS (ESI): *m/z* calcd for $C_{16}H_{18}N_2O_6Na$ [*M*+Na]$^+$: 357.1057, found: 357.1059.

***tert*-Butyl (4-hydroxy-2-oxo-1-(pyridin-2-ylmethoxy)-1,2-dihydroquinoline-3-carbonyl)glycinate (40a)**

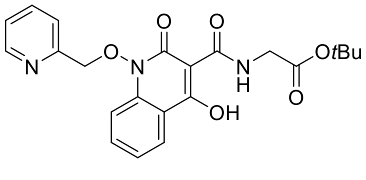

According to General Procedure C, ester **40a** (60 mg, 78%) was obtained from *tert*-butyl ester **39** (60 mg, 0.18 mmol) and pyridin-2-ylmethanol (30 mg, 0.27 mmol), following column chromatography (10 g Sfär Silica D; 35 mL/min; 100% cyclohexane (2 CV), followed by a linear gradient (14 CV): 0%→20% acetone in cyclohexane).

White solid, m.p.: 175-177 °C; ^{1}H NMR (500 MHz, 300 K, $CDCl_3$): δ = 10.41 (t, J = 5.5 Hz, 1H), 8.70 – 8.62 (m, 1H), 8.18 (dd, J = 8.0, 1.5 Hz, 1H), 7.83 – 7.75 (m, 2H), 7.74 – 7.63 (m, 2H), 7.40 – 7.27 (m, 2H), 5.39 (s, 2H), 4.14 (d, J = 5.5 Hz, 2H), 1.51 ppm (s, 9H); ^{13}C NMR (126 MHz, 300 K, $CDCl_3$): δ = 171.5, 170.9, 168.3, 159.2, 153.6, 148.9, 138.4, 138.0, 134.5, 125.4, 124.8, 124.2, 123.3, 115.0, 112.6, 97.2, 82.5, 77.8, 42.1, 28.2 ppm; IR (film): $\tilde{\nu}$ = 3247, 2980, 1743, 1656, 1562, 1411, 1371, 1238, 1157 cm^{-1}; HRMS (ESI): *m/z* calcd for $C_{22}H_{24}O_6N_3$ [*M*+H]$^+$: 426.1660, found: 426.1653.

(4-Hydroxy-2-oxo-1-(pyridin-2-ylmethoxy)-1,2-dihydroquinoline-3-carbonyl)glycine (40)[25]

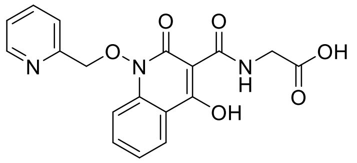

According to General Procedure D, carboxylic acid **40** (18 mg, 61%) was prepared from ester **40a** (33 mg, 0.08 mmol), following trituration with acetonitrile (3 × 5 mL; HPLC grade). The analytical data of carboxylic acid **40** are consistent with those reported.[25]

White solid, m.p.: >250 °C (decomposition); ^{1}H NMR (600 MHz, 300 K, DMSO-d_6): δ = 10.27 (t, *J* = 5.5 Hz, 1H), 8.63 (dd, *J* = 5.0, 2.0 Hz, 1H), 8.10 (dd, *J* = 8.0, 1.5 Hz, 1H), 7.91 – 7.87 (m, 1H), 7.82 (td, *J* = 8.5, 1.5 Hz, 1H), 7.75 – 7.71 (m, 2H), 7.43 (ddd, *J* = 7.5, 5.0, 1.0 Hz, 1H), 7.40 (td, *J* = 8.0, 1.0 Hz, 1H), 5.31 (s, 2H), 4.16 ppm (d, *J* = 5.5 Hz, 2H); ^{13}C NMR (151 MHz, 300 K, DMSO-d_6): δ = 170.7, 170.4, 170.3, 158.0, 153.5, 149.6, 138.1, 137.2, 134.8, 124.6, 124.4, 124.1, 123.3, 114.0, 112.4, 96.5, 77.9, 41.1 ppm; IR (film): $\tilde{\nu}$ = 3552, 3248, 2928, 1727, 1656, 1545, 1438, 1412, 1294, 1242, 1017 cm^{-1}; HRMS (ESI): *m/z* calcd for $C_{18}H_{16}O_6N_3$ [*M*+H]$^+$: 370.1034, found: 370.1029.

***tert*-Butyl (4-hydroxy-2-oxo-1-(thiazol-4-ylmethoxy)-1,2-dihydroquinoline-3-carbonyl)glycinate (41a)**

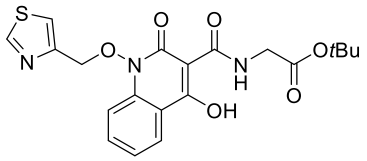

According to General Procedure C, ester **41a** (65 mg, 84%) was obtained from *tert*-butyl ester **39** (60 mg, 0.18 mmol) and (1,3-thiazol-4-yl)methanol (31 mg, 0.27 mmol), following column chromatography (10 g Sfär Silica D; 35 mL/min; 100% cyclohexane (2 CV), followed by a linear gradient (14 CV): 0%→20% acetone in cyclohexane).

White solid, m.p.: 170-174°C; ^{1}H NMR (600 MHz, 300 K, $CDCl_3$): δ = 10.43 (t, *J* = 5.5 Hz, 1H), 8.84 (d, *J* = 2.0 Hz, 1H), 8.15 (dd, *J* = 8.0, 1.5 Hz, 1H), 7.70 (d, *J* = 9.0 Hz, 1H), 7.66 (td, *J* = 8.5, 1.5 Hz, 1H), 7.57 (d, *J* = 2.0 Hz, 1H), 7.30 – 7.26 (m, 1H), 5.46 (s, 2H), 4.14 (d, *J* = 5.5 Hz, 2H), 1.51 ppm (s, 9H); ^{13}C NMR (151 MHz, 300 K, $CDCl_3$): δ = 171.5, 170.9, 168.3, 159.3, 153.6, 150.4, 138.6, 134.3, 125.3, 123.1, 120.7, 114.9, 112.7, 97.2, 82.5, 72.1, 42.0, 28.2 ppm; IR (film): $\tilde{\nu}$ = 3230, 3091, 2975, 1732, 1651, 1628, 1545, 1433, 1371, 1295, 1241, 1155, 1020 cm^{-1}; HRMS (ESI): *m/z* calcd for $C_{20}H_{22}O_6N_3S$ [*M*+H]$^+$: 432.1224, found: 432.1216.

(4-Hydroxy-2-oxo-1-(thiazol-4-ylmethoxy)-1,2-dihydroquinoline-3-carbonyl)glycine (41)

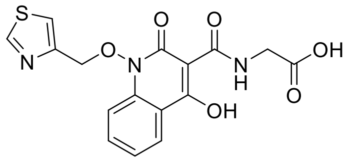

According to General Procedure D, carboxylic acid **41** (21 mg, 56%) was prepared from ester **41a** (45 mg, 0.10 mmol), following trituration with acetonitrile (3 × 5 mL; HPLC grade).

White solid, m.p.: 235-239 °C; ^{1}H NMR (600 MHz, 300 K, DMSO-d_6): δ = 12.99 (br s, 1H), 10.29 (t, *J* = 5.0 Hz, 1H), 9.16 (d, *J* = 2.0 Hz, 1H), 8.08 (dd, *J* = 8.0, 1.0 Hz, 1H), 8.02 (d, *J* = 2.0 Hz, 1H), 7.82 – 7.75 (m, 1H), 7.66 (d, *J* = 8.5 Hz, 1H), 7.37 (t, *J* = 7.5 Hz, 1H), 5.39 (s, 2H), 4.16 ppm (d, *J* = 5.5 Hz, 2H); ^{13}C NMR (151 MHz, 300 K, DMSO-d_6): δ = 171.2, 170.8(4), 170.8, 158.5, 155.6, 150.2, 138.7, 135.1, 125.0, 123.6, 122.7, 114.4, 112.9, 96.9, 72.0, 41.6 ppm; IR (film): $\tilde{\nu}$ = 3600, 3249, 3113, 1723, 1625, 1559, 1419, 1350, 1237, 1025 cm^{-1}; HRMS (ESI): *m/z* calcd for $C_{16}H_{14}O_6N_3S$ [*M*+H]$^+$: 376.0598, found: 376.0591.

***tert*-Butyl (4-hydroxy-1-(isoxazol-3-ylmethoxy)-2-oxo-1,2-dihydroquinoline-3-carbonyl)glycinate (42a)**

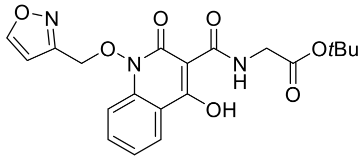

According to General Procedure C, ester **42a** (58 mg, 78%) was obtained from *tert*-butyl ester **39** (60 mg, 0.18 mmol) and isoxazol-3-ylmethanol (27 mg, 0.27 mmol), following column chromatography (10 g Sfär Silica D; 35 mL/min; 100% cyclohexane (2 CV), followed by a linear gradient (14 CV): 0%→15% acetone in cyclohexane).

White solid, m.p.: 141-143 °C; ^{1}H NMR (500 MHz, 300 K, $CDCl_3$): δ = 10.35 (t, *J* = 5.5 Hz, 1H), 8.46 (d, *J* = 1.5 Hz, 1H), 8.18 (dd, *J* = 8.0, 1.5 Hz, 1H), 7.69 (td, *J* = 8.5, 1.5 Hz, 1H), 7.55 (d, *J* = 8.5 Hz, 1H), 7.31 (t, *J* = 8.0 Hz, 1H), 6.70 (d, *J* = 1.5 Hz, 1H), 5.41 (s, 2H), 4.19 – 4.10 (m, 2H), 1.51 ppm (s, 9H); ^{13}C NMR (126 MHz, 300 K, $CDCl_3$): δ = 171.6, 170.8, 168.3, 159.4, 159.1, 157.1, 138.2, 134.5, 125.6, 123.4, 115.0, 112.2, 105.1, 97.2, 82.6, 68.2, 42.1, 28.2 ppm; IR (film): $\tilde{\nu}$ = 3255, 2980, 1742, 1656, 1563, 1412, 1370, 1238, 1158 cm^{-1}; HRMS (ESI): *m/z* calcd for $C_{20}H_{22}O_7N_3$ [*M*+H]$^+$: 416.1452, found: 416.1450.

(4-Hydroxy-1-(isoxazol-3-ylmethoxy)-2-oxo-1,2-dihydroquinoline-3-carbonyl)glycine (42)

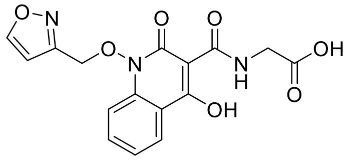

According to General Procedure D, carboxylic acid **42** (16 mg, 56%) was prepared from ester **42a** (43 mg, 0.10 mmol), following trituration with acetonitrile (3 × 5 mL; HPLC grade).

White solid, m.p.: 215-217 °C; ^{1}H NMR (500 MHz, 300 K, DMSO-d_6): δ = 12.98 (br s, 1H), 10.21 (t, *J* = 5.5 Hz, 1H), 9.00 (d, *J* = 1.5 Hz, 1H), 8.10 (dd, *J* = 8.0, 1.5 Hz, 1H), 7.84 (td, *J* = 8.5, 1.5 Hz, 1H), 7.62 (d, *J* = 8.5 Hz, 1H), 7.41 (td, *J* = 8.0, 1.0 Hz, 1H), 6.95 (d, *J* = 1.5 Hz, 1H), 5.42 (s, 2H), 4.16 ppm (d, *J* = 5.5 Hz, 2H); ^{13}C NMR (126 MHz, 300 K, DMSO-d_6): δ = 170.9, 170.3(3), 170.3, 160.9, 157.9, 157.0, 138.0, 134.9, 124.7, 123.4, 114.0, 112.3, 105.4, 96.4, 67.6, 41.1 ppm; IR (film): $\tilde{\nu}$ = 3254, 3132, 2931, 1738, 1720, 1631, 1565, 1430, 1407, 1273, 1148, 1035 cm^{-1}; HRMS (ESI): *m/z* calcd for $C_{16}H_{14}O_7N_3$ [*M*+H]$^+$: 360.0826, found: 360.0824.

tert-Butyl (4-hydroxy-1-((1-methyl-1_H_-imidazol-4-yl)methoxy)-2-oxo-1,2-dihydroquinoline-3-carbonyl)glycinate (43a)

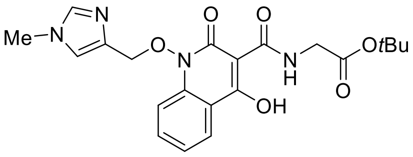

According to General Procedure C, ester **43a** (30 mg, 39%) was obtained from _tert_-butyl ester **39** (60 mg, 0.18 mmol) and (1-methyl-1_H_-imidazol-4-yl)methanol (30 mg, 0.27 mmol), following column chromatography (10 g Sfär Silica D; 35 mL/min; 100% cyclohexane (2 CV), followed by a linear gradient (14 CV): 0%→50% acetone in cyclohexane).

White solid, m.p.: 169-171 °C; ^{1}H NMR (500 MHz, 300 K, $CDCl_3$): δ = 10.39 (t, _J_ = 5.5 Hz, 1H), 8.17 – 8.11 (m, 1H), 7.94 (s, 1H), 7.81 (d, _J_ = 8.5 Hz, 1H), 7.74 – 7.69 (m, 1H), 7.31 – 7.27 (m, 1H), 7.20 (s, 1H), 5.27 (s, 2H), 4.14 (d, _J_ = 5.0 Hz, 2H), 3.76 (s, 3H), 1.51 ppm (s, 9H); ^{13}C NMR (126 MHz, 300 K, $CDCl_3$): δ = 171.5, 170.9, 168.4, 159.3, 138.5, 137.9, 134.6, 133.7, 125.2, 123.2, 122.4, 114.8, 113.2, 97.1, 82.5, 69.7, 42.0, 34.5, 28.2 ppm; IR (film): $\tilde{\nu}$ = 3243, 2980, 2929, 1741, 1655, 1562, 1411, 1271, 1240, 1158 cm^{-1}; HRMS (ESI): _m/z_ calcd for $C_{21}H_{25}O_6N_4$ [_M_+H]$^+$: 429.1769, found: 429.1765.

(4-Hydroxy-1-((1-methyl-1_H_-imidazol-4-yl)methoxy)-2-oxo-1,2-dihydroquinoline-3-carbonyl)glycine (43)

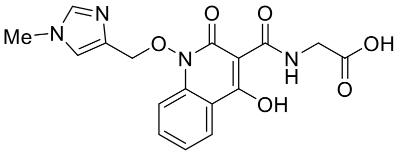

According to General Procedure D, carboxylic acid **43** (13 mg, 70%) was prepared from ester **43a** (22 mg, 0.05 mmol), following trituration with acetonitrile (3 × 5 mL; HPLC grade).

White solid, m.p.: 208-212 °C; ^{1}H NMR (600 MHz, 300 K, DMSO-d_6): δ = 10.28 (t, _J_ = 5.5 Hz, 1H), 8.09 (dd, _J_ = 8.0, 1.0 Hz, 1H), 7.97 (s, 1H), 7.82 (td, _J_ = 8.5, 1.5 Hz, 1H), 7.73 (d, _J_ = 8.5 Hz, 1H), 7.51 (s, 1H), 7.42 – 7.36 (m, 1H), 5.14 (s, 2H), 4.16 (d, _J_ = 5.5 Hz, 2H), 3.68 ppm (s, 3H); ^{13}C NMR (151 MHz, 300 K, DMSO-d_6): δ = 170.6, 170.4, 170.3, 158.1, 138.3 (2C), 134.6, 132.6, 124.5, 123.2, 122.7, 114.0, 112.7, 96.5, 69.8, 41.0, 33.6 ppm; IR (film): $\tilde{\nu}$ = 3259, 3117, 2980, 1723, 1624, 1594, 1559, 1415, 1295, 1236, 1132 cm^{-1}; HRMS (ESI): _m/z_ calcd for $C_{17}H_{17}O_6N_4$ [_M_+H]$^+$: 373.1143, found: 373.1141.

tert-Butyl (4-hydroxy-2-oxo-1-(pyridin-4-ylmethoxy)-1,2-dihydroquinoline-3-carbonyl)glycinate (44a)

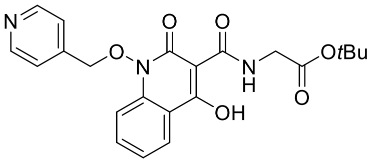

According to General Procedure C, ester **44a** (45 mg, 59%) was obtained from _tert_-butyl ester **39** (60 mg, 0.18 mmol) and pyridin-4-ylmethanol (30 mg, 0.27 mmol), following column chromatography (10 g Sfär Silica D; 35 mL/min; 100% cyclohexane (2 CV), followed by a linear gradient (14 CV): 0%→30% acetone in cyclohexane).

White solid, m.p., 168-170 °C; ^{1}H NMR (500 MHz, 300 K, $CDCl_3$): δ = 10.37 (t, *J* = 5.5 Hz, 1H), 8.70 (m, 2H), 8.21 (dd, *J* = 8.0, 1.5 Hz, 1H), 7.70 (td, *J* = 8.5, 1.5 Hz, 1H), 7.53 – 7.49 (m, 3H), 7.33 (t, *J* = 8.0 Hz, 1H), 5.26 (s, 2H), 4.15 (d, *J* = 5.5 Hz, 2H), 1.51 ppm (s, 9H); ^{13}C NMR (126 MHz, 300 K, $CDCl_3$): δ = 171.5, 170.6, 168.1, 158.9, 148.2, 145.3, 138.0, 134.4, 125.7, 123.5, 123.4, 115.0, 111.6, 97.1, 82.5, 75.2, 41.9, 28.1 ppm; IR (film): $\tilde{\nu}$ = 3260, 2931, 1738, 1651, 1561, 1415, 1292, 1242, 1155, 1016 cm^{-1}; HRMS (ESI): *m/z* calcd for $C_{22}H_{24}N_3O_6$ $[M+H]^+$: 426.1660, found: 426.1652.

(4-Hydroxy-2-oxo-1-(pyridin-4-ylmethoxy)-1,2-dihydroquinoline-3-carbonyl)glycine (44)

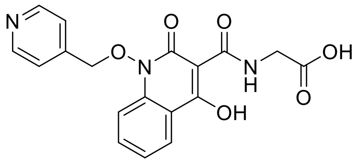

According to General Procedure D, carboxylic acid **44** (29 mg, 95%) was obtained from ester **44a** (35 mg, 0.08 mmol), following trituration with acetonitrile (3 × 5 mL; HPLC grade).

White solid, m.p.: >250 °C (decomposition); ^{1}H NMR (500 MHz, 300 K, DMSO-d_6): δ = 12.96 (br s, 1H), 10.23 (t, *J* = 5.5 Hz, 1H), 8.67 (d, *J* = 5.0 Hz, 2H), 8.12 (dd, *J* = 8.0, 1.5 Hz, 1H), 7.86 (td, *J* = 8.5, 1.5 Hz, 1H), 7.71 – 7.61 (m, 3H), 7.43 (td, *J* = 8.0, 1.0 Hz, 1H), 5.29 (s, 2H), 4.16 ppm (d, *J* = 5.5 Hz, 2H); ^{13}C NMR (126 MHz, 300 K, DMSO-d_6): δ = 170.8, 170.4, 170.3, 157.9, 149.9, 142.8, 137.9, 135.0, 124.8, 123.4(3), 123.4, 114.1, 112.3, 96.5, 75.3, 41.0 ppm; IR (film): $\tilde{\nu}$ = 3233, 2981, 1737, 1652, 1626, 1545, 1406, 1234, 1159, 1071 cm^{-1}; HRMS (ESI): *m/z* calcd for $C_{18}H_{16}N_3O_6$ $[M+H]^+$: 370.1034, found: 370.1030.

***tert*-Butyl (4-hydroxy-2-oxo-1-((tetrahydro-2*H*-pyran-4-yl)methoxy)-1,2-dihydroquinoline-3-carbonyl)glycinate (45a)**

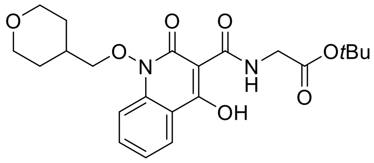

According to General Procedure C, ester **45a** (69 mg, 92%) was obtained from *tert*-butyl ester **39** (60 mg, 0.18 mmol) and (tetrahydro-2*H*-pyran-4-yl)methanol (31 mg, 0.27 mmol), following column chromatography (10 g Sfär Silica D; 35 mL/min; 100% cyclohexane (2 CV), followed by a linear gradient (14 CV): 0%→30% acetone in cyclohexane).

White solid, m.p.: 145-146 °C; ^{1}H NMR (600 MHz, 300 K, $CDCl_3$): δ = 10.39 (t, *J* = 5.5 Hz, 1H), 8.19 (d, *J* = 8.0, Hz, 1H), 7.72 (t, *J* = 8.5 Hz, 1H), 7.56 – 7.50 (m, 1H), 7.32 (t, *J* = 8.0 Hz, 1H), 4.15 – 4.01 (m, 6H), 3.49 (td, *J* = 12.0, 2.0 Hz, 2H), 2.30 – 2.23 (m, 1H), 1.89 – 1.85 (m, 2H), 1.60 – 1.53 (m, 2H), 1.50 ppm (s, 9H); ^{13}C NMR (151 MHz, 300 K, $CDCl_3$): δ = 171.3, 170.9, 168.3, 159.0, 138.3, 134.4, 125.7, 123.1, 115.2, 111.8, 97.3, 82.5, 79.5, 67.6, 42.0, 34.6, 29.7, 28.2 ppm; IR (film): $\tilde{\nu}$ = 3232, 2980, 1732, 1656, 1543, 1371, 1240, 1154, 1018 cm^{-1}; HRMS (ESI): *m/z* calcd for $C_{22}H_{29}N_2O_7$ $[M+H]^+$: 433.1969, found: 433.1960.

(4-Hydroxy-2-oxo-1-((tetrahydro-2*H*-pyran-4-yl)methoxy)-1,2-dihydroquinoline-3-carbonyl)glycine (45)

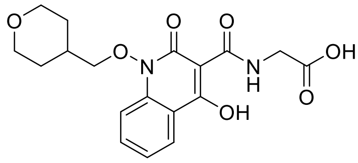

According to General Procedure D, carboxylic acid **45** (30 mg, 98%) was obtained from ester **45a** (35 mg, 0.10 mmol), following reverse-phase column chromatography (12 g Sfär C18 Duo; 12 mL/min; water (+ 0.1%$_{v/v}$ formic acid) (4 CV), followed by a linear gradient (25 CV): 0%→100% acetonitrile (+ 0.1%$_{v/v}$ formic acid) in water (+ 0.1%$_{v/v}$ formic acid)).

White solid, m.p.: 220-224 °C; ^{1}H NMR (600 MHz, 300 K, DMSO-*d*$_6$): δ = 12.93 (br s, 1H), 10.24 (t, *J* = 5.5 Hz, 1H), 8.11 (d, *J* = 8.0 Hz, 1H), 7.93 – 7.85 (m, 1H), 7.61 (d, *J* = 8.5 Hz, 1H), 7.45 – 7.39 (m, 1H), 4.14 (d, *J* = 5.5 Hz, 2H), 4.05 (d, *J* = 6.5 Hz, 2H), 3.95 – 3.87 (m, 2H), 3.41 – 3.35 (m, 2H), 2.23 – 2.16 (m, 1H), 1.84 – 1.70 (m, 2H), 1.49 – 1.41 ppm (m, 2H); ^{13}C NMR (151 MHz, 300 K, DMSO-*d*$_6$): δ = 170.6, 170.4, 170.3, 157.7, 137.9, 135.0, 124.8, 123.2, 114.1, 112.0, 96.5, 79.1, 66.5, 41.0, 33.8, 29.1 ppm; IR (film): $\tilde{\nu}$ = 3216, 2980, 1728, 1625, 1542, 1416, 1239, 1154, 1089 cm^{-1}; HRMS (ESI): *m/z* calcd for $C_{18}H_{21}N_2O_7$ [*M*+H]$^+$: 377.1343, found: 377.1337.

***tert*-Butyl (4-hydroxy-2-oxo-1-(2,2,2-trifluoroethoxy)-1,2-dihydroquinoline-3-carbonyl)glycinate (46a)**

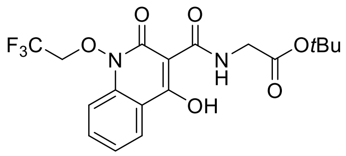

To a mixture of *tert*-butyl ester **39** (60 mg, 0.18 mmol, 1.0 equiv.) and K_2CO_3 (40 mg, 0.29 mmol, 1.6 equiv.) in dimethyl sulfoxide (1 mL; HPLC grade) was added 1,1,1-trifluoro-2-iodoethane (45 mg, 0.22 mmol, 1.2 equiv.) under an ambient atmosphere at room temperature. The reaction mixture was heated at 50 °C for 14 h before being cooled to room temperature and being purified by reverse-phase column chromatography (30 g Sfär C18 Duo; 25 mL/min; water (+ 0.1%$_{v/v}$ formic acid) (4 CV), followed by a linear gradient (25 CV): 0%→100% acetonitrile (+ 0.1%$_{v/v}$ formic acid) in water (+ 0.1%$_{v/v}$ formic acid)) to afford ester **46a** (44 mg, 68%).

White solid, m.p.: 153-155 °C; ^{1}H NMR (600 MHz, 300 K, $CDCl_3$): δ = 10.20 (t, *J* = 5.5 Hz, 1H), 8.20 (dd, *J* = 8.0, 1.5 Hz, 1H), 7.75 (td, *J* = 8.5, 1.5 Hz, 1H), 7.64 (d, *J* = 8.5 Hz, 1H), 7.36 (t, *J* = 8.0 Hz, 1H), 4.74 (q, *J* = 8.5 Hz, 2H), 4.13 (d, *J* = 5.5 Hz, 2H), 1.51 ppm (s, 9H); ^{13}C NMR (151 MHz, 300 K, $CDCl_3$): δ = 171.9, 170.6, 168.2, 159.1, 138.0, 134.7, 125.6, 123.7, 122.7 (q, *J* = 278.5 Hz), 115.1, 111.9, 96.9, 82.7, 71.9 (q, *J* = 35.0 Hz), 42.0, 28.2 ppm; ^{19}F NMR (565 MHz, 300 K, $CDCl_3$): δ = –72.6 ppm (t, *J* = 8.0 Hz); IR (film): $\tilde{\nu}$ = 3007, 2921, 1740, 1661, 1569, 1411, 1275, 1243, 1175, 1056 cm^{-1}; HRMS (ESI): *m/z* calcd for $C_{18}H_{20}O_6N_2F_3$ [*M*+H]$^+$: 417.1273, found: 417.1263.

(4-Hydroxy-2-oxo-1-(2,2,2-trifluoroethoxy)-1,2-dihydroquinoline-3-carbonyl)glycine (46)[25]

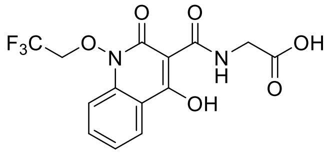

According to General Procedure D, carboxylic acid **46** (18 mg, 63%) was prepared from ester **46a** (30 mg, 0.08 mmol), following reverse-phase column chromatography (12 g Sfär C18 Duo; 12 mL/min; water (+ 0.1%$_{v/v}$ formic acid) (4 CV), followed by a linear gradient (25 CV): 0%→100% acetonitrile (+ 0.1%$_{v/v}$ formic acid) in water (+ 0.1%$_{v/v}$ formic acid)). The analytical data of carboxylic acid **46** are consistent with those reported.[25]

White solid, m.p.: 220-223 °C; ^{1}H NMR (600 MHz, 300 K, DMSO-d_6): δ = 10.10 (t, J = 5.5 Hz, 1H), 8.13 (dd, J = 8.0, 1.5 Hz, 1H), 7.91 (td, J = 8.5, 1.5 Hz, 1H), 7.60 (d, J = 8.5 Hz, 1H), 7.50 – 7.41 (m, 1H), 5.04 (q, J = 9.0 Hz, 2H), 4.13 ppm (d, J = 5.5 Hz, 2H); ^{13}C NMR (151 MHz, 300 K, DMSO-d_6): δ = 171.1, 170.2, 170.1, 157.8, 137.7, 135.0, 124.9, 123.6, 123.0 (q, J = 279.0 Hz), 114.2, 111.7, 96.2, 71.1 (q, J = 33.5 Hz), 41.2 ppm; ^{19}F NMR (565 MHz, DMSO-d_6): δ = –71.3 ppm (t, J = 8.5 Hz); IR (film): $\tilde{\nu}$ = 3216, 2967, 1726, 1654, 1626, 1596, 1424, 1163, 1057 cm^{-1}; HRMS (ESI): m/z calcd for $C_{14}H_{12}O_6N_2F_3$ [M+H]$^+$: 361.0642, found: 361.0638.

(1-(Benzyloxy)-4-hydroxy-2-oxo-1,2-dihydroquinoline-3-carbonyl)glycine (47)[25]

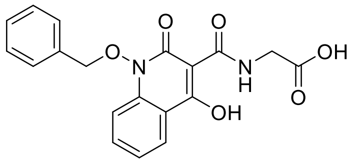

According to General Procedure D, carboxylic acid **47** (31 mg, 89%) was obtained from ester **38** (40 mg, 0.09 mmol), following reverse-phase column chromatography (12 g Sfär C18 Duo; 12 mL/min; water (+ 0.1%$_{v/v}$ formic acid) (4 CV), followed by a linear gradient (25 CV): 0%→100% acetonitrile (+ 0.1%$_{v/v}$ formic acid) in water (+ 0.1%$_{v/v}$ formic acid)). The analytical data of carboxylic acid **47** are consistent with those reported.[25]

White solid, m.p.: 231-233 °C; ^{1}H NMR (600 MHz, 300 K, DMSO-d_6): δ = 12.98 (s, 1H), 10.29 (t, J = 5.5 Hz, 1H), 8.11 (dd, J = 8.0, 1.5 Hz, 1H), 7.85 (td, J = 8.5, 1.5 Hz, 1H), 7.67 – 7.63 (m, 3H), 7.48 – 7.39 (m, 4H), 5.22 (s, 2H), 4.16 ppm (d, J = 5.5 Hz, 2H); ^{13}C NMR (151 MHz, 300 K, DMSO-d_6): δ = 170.7, 170.4, 170.3, 158.0, 138.1, 134.9, 133.9, 129.9, 129.2, 128.6, 124.7, 123.2, 114.0, 112.4, 96.5, 77.0, 41.1 ppm; IR (film): $\tilde{\nu}$ = 3209, 2981, 1733, 1622, 1538, 1429, 1402, 1237, 1014 cm^{-1}; HRMS (ESI): m/z calcd for $C_{19}H_{17}N_2O_6$ [M+H]$^+$: 369.1081, found: 369.1077.

***tert*-Butyl (1-(cyclopentylmethoxy)-4-hydroxy-2-oxo-1,2-dihydroquinoline-3-carbonyl)glycinate (48a)**

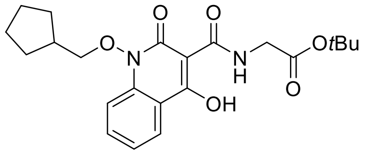

According to General Procedure C, ester **48a** (66 mg, 88%) was obtained from *tert*-butyl ester **39** (60 mg, 0.18 mmol) and cyclopentylmethanol (27 mg, 0.27 mmol), following column chromatography (10 g Sfär Silica D; 35 mL/min; 100% cyclohexane (2 CV), followed by a linear gradient (14 CV): 0%→10% ethyl acetate in cyclohexane).

White solid, m.p.: 97-99 °C; ^{1}H NMR (500 MHz, 300 K, $CDCl_3$): δ = 10.43 (t, *J* = 5.5 Hz, 1H), 8.18 (dd, *J* = 8.0, 1.5 Hz, 1H), 7.71 (td, *J* = 8.5, 1.5 Hz, 1H), 7.59 (d, *J* = 8.5 Hz, 1H), 7.31 (t, *J* = 8.0 Hz, 1H), 4.15 – 4.10 (m, 4H), 2.50 – 2.43 (m, 1H), 2.01 – 1.88 (m, 2H), 1.77 – 1.61 (m, 4H), 1.51 – 1.42 ppm (m, 11H); ^{13}C NMR (126 MHz, 300 K, $CDCl_3$): δ = 171.2, 171.0, 168.3, 159.1, 138.4, 134.3, 125.5, 123.0, 115.1, 112.2, 97.4, 82.4, 79.9, 42.0, 38.3, 29.8, 28.2, 25.6 ppm; IR (film): $\tilde{\nu}$ = 3251, 2952, 2870, 1745, 1656, 1563, 1409, 1370, 1236, 1159 cm^{-1}; HRMS (ESI): *m/z* calcd for $C_{22}H_{29}O_6N_2$ [*M*+H]$^+$: 417.2020, found: 417.2014.

(1-(Cyclopentylmethoxy)-4-hydroxy-2-oxo-1,2-dihydroquinoline-3-carbonyl)glycine (48)[25]

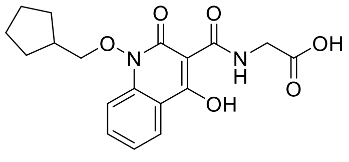

According to General Procedure D, carboxylic acid **48** (22 mg, 76%) was prepared from ester **48a** (35 mg, 0.08 mmol), following trituration with acetonitrile (3 × 5 mL; HPLC grade). The analytical data of carboxylic acid **48** are consistent with those reported.[25]

White solid, m.p.: 229-231 °C; ^{1}H NMR (600 MHz, 300 K, DMSO-*d*$_6$): δ = 12.94 (br s, 1H), 10.25 (t, *J* = 5.5 Hz, 1H), 8.11 (d, *J* = 8.0 Hz, 1H), 7.92 – 7.85 (m, 1H), 7.63 (d, *J* = 8.5 Hz, 1H), 7.42 (t, *J* = 7.5 Hz, 1H), 4.13 (d, *J* = 5.5 Hz, 2H), 4.07 (d, *J* = 7.0 Hz, 2H), 2.45 – 2.38 (m, 1H), 1.87 – 1.81 (m, 2H), 1.69 – 1.52 (m, 4H), 1.50 – 1.43 ppm (m, 2H); ^{13}C NMR (151 MHz, 300 K, DMSO-*d*$_6$): δ = 170.5, 170.4, 170.3, 157.7, 137.9, 135.0, 124.8, 123.2, 114.1, 112.1, 96.5, 79.0, 41.0, 37.4, 29.0, 25.1 ppm; IR (film): $\tilde{\nu}$ = 3263, 2952, 2869, 1736, 1620, 1577, 1493, 1407, 1347, 1296, 1227, 1020 cm^{-1}; HRMS (ESI): *m/z* calcd for $C_{18}H_{21}O_6N_2$ [*M*+H]$^+$: 361.1394, found: 361.1391.

***tert*-Butyl (1-(cyclohexylmethoxy)-4-hydroxy-2-oxo-1,2-dihydroquinoline-3-carbonyl)glycinate (49a)**

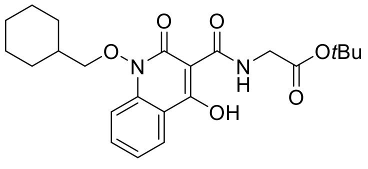

According to General Procedure C, ester **49a** (56 mg, 72%) was obtained from *tert*-butyl ester **39** (60 mg, 0.18 mmol) and cyclohexylmethanol (33 µL, 0.27 mmol), following column

chromatography (10 g Sfär Silica D; 35 mL/min; 100% cyclohexane (2 CV), followed by a linear gradient (14 CV): 0%→20% ethyl acetate in cyclohexane).

White solid, m.p.: 108-110 °C; ^{1}H NMR (600 MHz, 300 K, $CDCl_3$): δ = 10.43 (t, *J* = 5.0 Hz, 1H), 8.18 (d, *J* = 8.0 Hz, 1H), 7.74 – 7.68 (m, 1H), 7.57 (d, *J* = 8.5 Hz, 1H), 7.34 – 7.28 (m, 1H), 4.11 (d, *J* = 5.5 Hz, 2H), 4.02 (d, *J* = 5.5 Hz, 2H), 2.04 – 1.93 (m, 3H), 1.83 – 1.77 (m, 2H), 1.75 – 1.69 (m, 1H), 1.50 (s, 9H), 1.40 – 1.30 (m, 2H), 1.29 – 1.14 ppm (m, 3H); ^{13}C NMR (151 MHz, 300 K, $CDCl_3$): δ = 171.2, 171.0, 168.3, 159.0, 138.4, 134.3, 125.5, 123.0, 115.2, 112.1, 97.4, 82.4, 80.5, 42.0, 37.2, 29.9, 28.2, 26.5, 25.8 ppm; IR (film): $\tilde{\nu}$ = 3245, 2930, 1745, 1657, 1562, 1410, 1370, 1236, 1157 cm^{-1}; HRMS (ESI): *m/z* calcd for $C_{23}H_{31}N_2O_6$ [*M*+H]$^{+}$: 431.2177, found: 431.2166.

(1-(Cyclohexylmethoxy)-4-hydroxy-2-oxo-1,2-dihydroquinoline-3-carbonyl)glycine (49)[25]

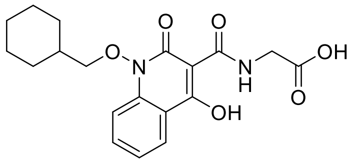

According to General Procedure D, carboxylic acid **49** (26 mg, 77%) was obtained from ester **49a** (40 mg, 0.09 mmol), following reverse-phase column chromatography (12 g Sfär C18 Duo; 12 mL/min; water (+ 0.1%$_{v/v}$ formic acid) (4 CV), followed by a linear gradient (25 CV): 0%→100% acetonitrile (+ 0.1%$_{v/v}$ formic acid) in water (+ 0.1%$_{v/v}$ formic acid)). The analytical data of carboxylic acid **49** are consistent with those reported.[25]

White solid, m.p. 228-230 °C; ^{1}H NMR (600 MHz, 300 K, DMSO-*d*$_6$): δ = 12.90 (br s, apparent 2H), 10.25 (t, *J* = 5.5 Hz, 1H), 8.11 (d, *J* = 8.0 Hz, 1H), 7.91 – 7.86 (m, 1H), 7.60 (d, *J* = 8.5 Hz, 1H), 7.45 – 7.39 (m, 1H), 4.13 (d, *J* = 5.5 Hz, 2H), 4.00 (d, *J* = 5.5 Hz, 2H), 1.94 – 1.86 (m, 3H), 1.76 – 1.72 (m, 2H), 1.71 – 1.64 (m, 1H), 1.37 – 1.10 ppm (m, 5H); ^{13}C NMR (151 MHz, 300 K, DMSO-*d*$_6$): δ = 170.5, 170.4, 170.3, 157.7, 137.9, 135.0, 124.8, 123.2, 114.1, 112.0, 96.5, 79.8, 41.0, 36.4, 29.2, 25.9, 25.2 ppm; IR (film): $\tilde{\nu}$ = 3253, 2981, 1738, 1620, 1542, 1395, 1227, 1157 cm^{-1}; HRMS (ESI): *m/z* calcd for $C_{19}H_{23}N_2O_6$ [*M*+H]$^{+}$: 375.1551, found: 375.1545.

***tert*-Butyl (4-hydroxy-1-(naphthalen-1-ylmethoxy)-2-oxo-1,2-dihydroquinoline-3-carbonyl)-glycinate (50a)**

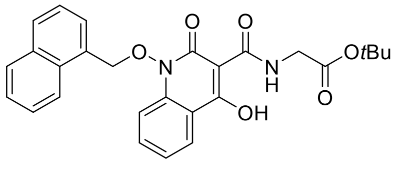

According to General Procedure C, ester **50a** (47 mg, 55%) was obtained from *tert*-butyl ester **39** (60 mg, 0.18 mmol) and naphthalen-1-ylmethanol (43 mg, 0.27 mmol), following column chromatography (10 g Sfär Silica D; 35 mL/min; 100% cyclohexane (2 CV), followed by a linear gradient (14 CV): 0%→10% ethyl acetate in cyclohexane).

White solid, m.p.: 162-163 °C; ^{1}H NMR (600 MHz, 300 K, $CDCl_3$): δ = 10.55 (t, *J* = 5.5 Hz, 1H), 8.78 (d, *J* = 8.5 Hz, 1H), 8.18 (d, *J* = 8.0 Hz, 1H), 7.96 – 7.88 (m, 2H), 7.70 (t, *J* = 8.5 Hz, 1H), 7.63 (d, *J* = 7.0 Hz,

1H), 7.62 – 7.56 (m, 2H), 7.53 – 7.47 (m, 2H), 7.28 (t, *J* = 8.0 Hz, 1H), 5.69 (s, 2H), 4.20 (d, *J* = 5.5 Hz, 2H), 1.55 ppm (s, 9H); ^{13}C NMR (151 MHz, 300 K, $CDCl_3$): δ = 171.4, 171.1, 168.3, 159.4, 138.4, 134.3, 133.9, 132.7, 130.5, 130.2, 129.1, 128.6, 127.3, 126.4, 125.5, 125.3, 124.9, 123.1, 115.1, 112.4, 97.4, 82.5, 75.8, 42.1, 28.3 ppm; IR (film): $\tilde{\nu}$ = 3233, 2981, 1747, 1654, 1561, 1405, 1232, 1159 cm^{-1}; HRMS (ESI): *m/z* calcd for $C_{27}H_{27}N_2O_6$ [*M*+H]$^+$: 475.1864, found: 475.1857.

(4-Hydroxy-1-(naphthalen-1-ylmethoxy)-2-oxo-1,2-dihydroquinoline-3-carbonyl)glycine (50)

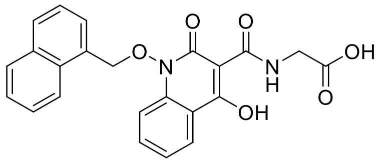

According to General Procedure D, carboxylic acid **50** (14 mg, 67%) was obtained from ester **50a** (25 mg, 0.05 mmol), following reverse-phase column chromatography (12 g Sfär C18 Duo; 12 mL/min; water (+ 0.1%$_{v/v}$ formic acid) (4 CV), followed by a linear gradient (25 CV): 0%→100% acetonitrile (+ 0.1%$_{v/v}$ formic acid) in water (+ 0.1%$_{v/v}$ formic acid)).

White solid, m.p. 185-187 °C; ^{1}H NMR (600 MHz, 300 K, DMSO-d_6): δ = 12.95 (br s, 1H), 10.33 (t, *J* = 5.5 Hz, 1H), 8.81 (d, *J* = 8.5 Hz, 1H), 8.14 (d, *J* = 8.0 Hz, 1H), 8.06 – 8.01 (m, 2H), 7.86 – 7.78 (m, 2H), 7.72 – 7.67 (m, 1H), 7.65 – 7.56 (m, 2H), 7.54 (d, *J* = 8.5 Hz, 1H), 7.45 – 7.39 (m, 1H), 5.68 (s, 2H), 4.21 ppm (d, *J* = 5.5 Hz, 2H); ^{13}C NMR (151 MHz, 300 K, DMSO-d_6): δ = 170.8, 170.5, 170.4, 158.2, 137.9, 135.0, 133.3, 132.1, 130.1, 129.9, 129.5, 128.4, 126.8, 126.3, 125.5, 124.8 (2C), 123.3, 114.2, 112.3, 96.5, 75.0, 41.1 ppm; IR (film): $\tilde{\nu}$ = 3254, 2981, 1747, 1655, 1627, 1561, 1398, 1237, 1157 cm^{-1}; HRMS (ESI): *m/z* calcd for $C_{23}H_{19}N_2O_6$ [*M*+H]$^+$: 419.1238, found: 419.1230.

***tert*-Butyl (4-hydroxy-1-(neopentyloxy)-2-oxo-1,2-dihydroquinoline-3-carbonyl)glycinate (51a)**

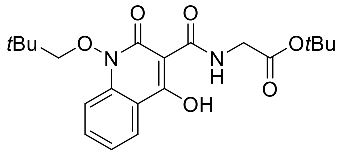

According to General Procedure C, ester **51a** (46 mg, 63%) was obtained from *tert*-butyl ester **39** (60 mg, 0.18 mmol) and 2,2-dimethylpropan-1-ol (24 mg, 0.27 mmol), following column chromatography (10 g Sfär Silica D; 35 mL/min; 100% cyclohexane (2 CV), followed by a linear gradient (14 CV): 0%→10% ethyl acetate in cyclohexane).

White solid, m.p.: 137-140 °C; ^{1}H NMR (600 MHz, 300 K, $CDCl_3$): δ = 10.42 (t, *J* = 5.5 Hz, 1H), 8.18 (dd, *J* = 8.0, 1.5 Hz, 1H), 7.70 (td, *J* = 8.5, 1.5 Hz, 1H), 7.62 – 7.53 (m, 1H), 7.31 (t, *J* = 8.0 Hz, 1H), 4.12 (d, *J* = 5.5 Hz, 2H), 3.91 (s, 2H), 1.50 (s, 9H), 1.19 ppm (s, 9H); ^{13}C NMR (151 MHz, 300 K $CDCl_3$): δ = 171.2, 171.1, 168.3, 159.0, 138.5, 134.3, 125.6, 123.0, 115.2, 112.0, 97.4, 84.1, 82.4, 41.9, 32.3, 28.2, 26.8 ppm; IR (film): $\tilde{\nu}$ = 3236, 2959, 1745, 1658, 1563, 1408, 1369, 1236, 1158 cm^{-1}; HRMS (ESI): *m/z* calcd for $C_{21}H_{29}N_2O_6$ [*M*+H]$^+$: 405.2020, found: 405.2012.

(4-Hydroxy-1-(neopentyloxy)-2-oxo-1,2-dihydroquinoline-3-carbonyl)glycine (51)

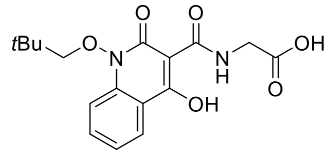

According to General Procedure D, carboxylic acid **51** (15 mg, 43%) was obtained from ester **51a** (40 mg, 0.10 mmol), following reverse-phase column chromatography (12 g Sfär C18 Duo; 12 mL/min; water (+ 0.1%$_{v/v}$ formic acid) (4 CV), followed by a linear gradient (25 CV): 0%→100% acetonitrile (+ 0.1%$_{v/v}$ formic acid) in water (+ 0.1%$_{v/v}$ formic acid)).

White solid, m.p. 225-228 °C; ^{1}H NMR (600 MHz, 300 K, DMSO-*d*$_6$): δ = 12.94 (br s, 1H) 10.24 (t, *J* = 5.0 Hz, 1H), 8.11 (d, *J* = 8.0 Hz, 1H), 7.93 – 7.86 (m, 1H), 7.57 (d, *J* = 8.5 Hz, 1H), 7.45 – 7.49 (m, 1H), 4.13 (d, *J* = 5.5 Hz, 2H), 3.88 (s, 2H), 1.13 ppm (s, 9H); ^{13}C NMR (151 MHz, 300 K, DMSO-*d*$_6$): δ = 170.5, 170.4, 170.3, 157.7, 137.9, 135.0, 124.8, 123.2, 114.2, 111.7, 96.5, 83.3, 41.0, 31.7, 26.4 ppm; IR (film): $\tilde{\nu}$ = 3258, 2981, 1736, 1619, 1574, 1540, 1405, 1225, 1018 cm^{-1}; HRMS (ESI): *m/z* calcd for $C_{17}H_{21}N_2O_6$ [*M*+H]$^+$: 349.1394, found: 349.1389.

1-(Cyclopropylmethyl)-2*H*-benzo[*d*][1,3]oxazine-2,4(1*H*)-dione (52b)[30]

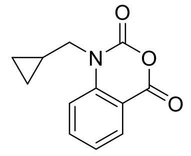

To isatoic anhydride **52a** (489 mg, 3.0 mmol, 1.0 equiv.) in anhydrous dichloromethane were sequentially added cyclopropylmethanol (0.24 mL, 3.0 mmol, 1.0 equiv.), triphenylphosphine (787 mg, 3.0 mmol, 1.0 equiv.) and DMEAD[17] (703 mg, 3.0 equiv., 1.0 equiv.) at ambient temperature under a N_2 atmosphere. The reaction mixture was stirred for 16 h before removal of the solvent under reduced pressure. The crude residue was purified by column chromatography (25 g Sfär Silica D; 60 mL/min; 100% cyclohexane (2 CV), followed by a linear gradient (14 CV): 0%→30% ethyl acetate in cyclohexane) to afford oxazine **52b** (501 mg, 77%). The analytical data of oxazine **52b** are consistent with those reported.[30]

White solid, m.p. 115-117 °C; ^{1}H NMR (600 MHz, 300 K, $CDCl_3$): δ = 8.18 (d, *J* = 7.5 Hz, 1H), 7.76 (t, *J* = 8.0 Hz, 1H), 7.32 – 7.28 (m, 2H), 4.01 (d, *J* = 7.0 Hz, 2H), 1.33 – 1.16 (m, 1H), 0.66 – 0.51 ppm (m, 4H); ^{13}C NMR (151 MHz, 300 K, $CDCl_3$): δ = 158.7, 148.4, 141.8, 137.3, 131.1, 124.0, 114.3, 112.0, 49.2, 9.3, 4.1 ppm; IR (film): $\tilde{\nu}$ = 2981, 1775, 1726, 1608, 1494, 1478, 1376, 1345, 1315, 1272, 1030 cm^{-1}; HRMS (ESI): *m/z* calcd for $C_{12}H_{12}O_3N$ [*M*+H]$^+$: 218.0812, found: 218.0812.

Ethyl 1-(cyclopropylmethyl)-4-hydroxy-2-oxo-1,2-dihydroquinoline-3-carboxylate (52c)[31]

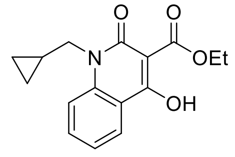

To a mixture of sodium hydride (35 mg, 1.18 mmol, 1.0 equiv.) in anhydrous *N*,*N*-dimethylformamide (6 mL) was added diethyl malonate (0.18 mL, 1.24 mmol, 1.05 equiv.) at ambient temperature under a N_2 atmosphere. After stirring for 30 min, oxazine **52b** (270 mg, 1.24 mmol, 1.05 equiv.) was added to the reaction mixture, which was then heated at 90 °C for 16 h under a N_2 atmosphere. The reaction mixture was then cooled

to ambient temperature, and poured onto ice-cold H_2O. The pH of the aqueous mixture was acidified (pH 4-5) with the dropwise addition of aqueous HCl (4M) and extracted three times with ethyl acetate. The combined organic extracts were washed with brine, dried over anhydrous Na_2SO_4, filtered, evaporated, and purified by column chromatography (10 g Sfär Silica D; 35 mL/min; 100% cyclohexane (2 CV), followed by a linear gradient (14 CV): 0%→30% ethyl acetate in cyclohexane) to afford ethyl ester **52c** (200 mg, 59%). The analytical data of ethyl ester **52c** are consistent with those reported.[31]

White solid, m.p.: >250 °C (decomposition); ^{1}H NMR (600 MHz, 300 K, $CDCl_3$): δ = 14.23 (s, 1H), 8.20 (d, *J* = 8.0 Hz, 1H), 7.67 (t, *J* = 8.0 Hz, 1H), 7.43 (d, *J* = 8.5 Hz, 1H), 7.24 (t, *J* = 8.5 Hz, 1H), 4.51 (q, *J* = 7.0 Hz, 2H), 4.19 (d, *J* = 7.0 Hz, 2H), 1.47 (t, *J* = 7.0 Hz, 3H), 1.30 – 1.16 (m, 1H), 0.63 – 0.45 ppm (m, 4H); ^{13}C NMR (151 MHz, 300 K, $CDCl_3$): δ = 173.0, 171.8, 159.8, 141.1, 134.3, 126.0, 121.8, 115.2, 114.7, 98.2, 62.5, 45.8, 14.4, 9.9, 4.3 ppm; IR (film): $\tilde{\nu}$ = 3213, 2981, 1742, 1633, 1563, 1408, 1369, 1233, 1156, 1023 cm^{-1}; HRMS (ESI): *m/z* calcd for $C_{16}H_{18}O_4N$ [*M*+H]$^+$: 288.1230, found: 288.1228.

tert-Butyl (1-(cyclopropylmethyl)-4-hydroxy-2-oxo-1,2-dihydroquinoline-3-carbonyl)glycinate (52d)

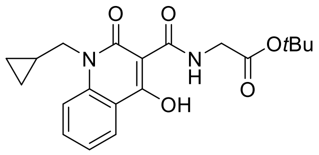

According to General Procedure A, *tert*-butyl ester **52d** (191 mg, 81%) was obtained from ethyl ester **52c** (180 mg, 0.63 mmol) and glycine *tert*-butyl ester hydrochloride (121 mg, 0.72 mmol), following column chromatography (25 g Sfär Silica D; 60 mL/min; 100% cyclohexane (2 CV), followed by a linear gradient (14 CV): 0%→10% ethyl acetate in cyclohexane).

Yellow oil; ^{1}H NMR (600 MHz, 300 K, $CDCl_3$): δ = 10.78 (t, *J* = 5.5 Hz, 1H), 8.23 (t, *J* = 8.5 Hz, 1H), 7.68 (t, *J* = 8.5 Hz, 1H), 7.49 (d, *J* = 8.0 Hz, 1H), 7.29 (t, *J* = 8.0 Hz, 1H), 4.23 (d, *J* = 7.0 Hz, 2H), 4.13 (d, *J* = 5.5 Hz, 2H), 1.51 (s, 9H), 1.27 – 1.22 (m, 1H), 0.65 – 0.47 ppm (m, 4H); ^{13}C NMR (151 MHz, 300 K, $CDCl_3$): δ = 171.8, 171.4, 168.5, 162.8, 139.8, 133.8, 125.8, 122.3, 116.4, 114.8, 97.1, 82.4, 45.8, 42.0, 28.2, 10.0, 4.2 ppm; IR (film): $\tilde{\nu}$ = 2981, 1742, 1633, 1563, 1407, 1234, 1155 cm^{-1}; HRMS (ESI): *m/z* calcd for $C_{20}H_{24}O_5N_2Na$ [*M*+Na]$^+$: 395.1577, found: 395.1574.

(1-(Cyclopropylmethyl)-4-hydroxy-2-oxo-1,2-dihydroquinoline-3-carbonyl)glycine (52)[23]

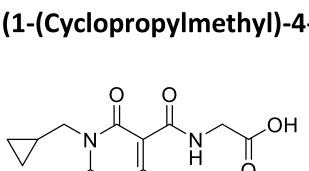

According to General Procedure D, carboxylic acid **52** (112 mg, 72%) was obtained from *tert*-butyl ester **52d** (174 mg, 0.49 mmol), following reverse-phase column chromatography (12 g Sfär C18 Duo; 12 mL/min; water (+ 0.1%$_{v/v}$ formic acid) (4 CV), followed by a linear gradient (25 CV): 0%→100% acetonitrile (+ 0.1%$_{v/v}$ formic acid) in water (+ 0.1%$_{v/v}$ formic acid)). The analytical data of carboxylic acid **52** are consistent with those reported.[23]

White solid, m.p.: 239-243 °C; ^{1}H NMR (600 MHz, 300 K, DMSO-d_6): δ = 12.93 (br s, 1H), 10.55 (t, *J* = 5.5 Hz, 1H), 8.12 (d, *J* = 8.0 Hz, 1H), 7.82 (t, *J* = 8.0 Hz, 1H), 7.76 (d, *J* = 8.0 Hz, 1H), 7.38 (t, *J* = 8.0 Hz, 1H), 4.24 (d, *J* = 7.0 Hz, 2H), 4.13 (d, *J* = 5.5 Hz, 2H), 1.30 – 1.18 (m, 1H), 0.52 – 0.42 ppm (m, 4H); ^{13}C NMR (151 MHz, 300 K, DMSO-d_6): δ = 171.1, 170.7, 170.4, 161.6, 139.3, 134.5, 124.7, 122.5, 115.6, 115.1, 95.9, 45.0, 41.0, 9.8, 3.7 ppm; IR (film): $\tilde{\nu}$ = 3294, 2981, 1758, 1624, 1575, 1545, 1408, 1191, 1176 cm^{-1}; HRMS (ESI): *m/z* calcd for $C_{16}H_{17}O_5N_2$ [*M*+H]$^+$: 317.1132, found: 317.1130.

1-(2-Cyclopropylethyl)-2*H*-benzo[*d*][1,3]oxazine-2,4(1*H*)-dione (53a)

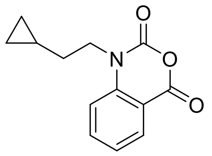

To isatoic anhydride **52a** (489 mg, 3.0 mmol, 1.0 equiv.) in anhydrous dichloromethane were sequentially added 2-cyclopropylethan-1-ol (0.27 mL, 3.0 mmol, 1.0 equiv.), triphenylphosphine (787 mg, 3.0 mmol, 1.0 equiv.) and DMEAD[17] (703 mg, 3.0 equiv., 1.0 equiv.) at ambient temperature under a N_2 atmosphere. The reaction mixture was stirred for 16 h before removal of the solvent under reduced pressure. The crude residue was purified by column chromatography (25 g Sfär Silica D; 60 mL/min; 100% cyclohexane (2 CV), followed by a linear gradient (14 CV): 0%→30% ethyl acetate in cyclohexane) to afford oxazine **53a** (523 mg, 75%).

White solid, m.p. 92-93 °C; ^{1}H NMR (600 MHz, 300 K, $CDCl_3$): δ = 8.15 (d, *J* = 8.0 Hz, 1H), 7.75 (t, *J* = 8.5 Hz, 1H), 7.28 (t, *J* = 8.0 Hz, 1H), 7.20 (d, *J* = 8.5 Hz, 1H), 4.17 (t, *J* = 7.0 Hz, 2H), 1.66 (q, *J* = 7.0 Hz, 2H), 0.87 – 0.66 (m, 1H), 0.58 – 0.42 (m, 2H), 0.11 – 0.08 ppm (m, 2H); ^{13}C NMR (151 MHz, 300 K, $CDCl_3$): δ = 158.7, 147.8, 141.6, 137.3, 131.1, 124.0, 114.0, 111.9, 45.0, 31.9, 8.3, 4.4 ppm; IR (film): $\tilde{\nu}$ = 2972, 1776, 1724, 1606, 1494, 1478, 1380, 1325, 1252, 1031 cm^{-1}; HRMS (ESI): *m/z* calcd for $C_{13}H_{14}O_3N$ [*M*+H]$^+$: 232.0968, found: 232.0969.

Ethyl 1-(2-cyclopropylethyl)-4-hydroxy-2-oxo-1,2-dihydroquinoline-3-carboxylate (53b)

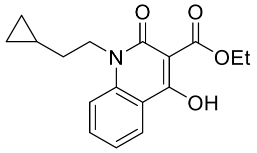

To sodium hydride (39 mg, 1.6 mmol, 1.05 equiv.) in anhydrous *N*,*N*-dimethylformamide (8 mL) was added diethyl malonate (0.24 mL, 1.5 mmol, 1.0 equiv.) at ambient temperature under a N_2 atmosphere. After stirring for 30 min, oxazine **53a** (375 mg, 1.6 mmol, 1.05 equiv.) was added to the reaction mixture which was then heated at 90 °C for 16 h under a N_2 atmosphere. The reaction mixture was then cooled to ambient temperature, and poured onto ice-cold H_2O. The pH of the aqueous mixture was acidified (pH 4-5) with the dropwise addition of aqueous HCl (4M) and extracted three times with ethyl acetate. The combined organic extracts were washed with brine, dried over anhydrous Na_2SO_4, filtered, evaporated, and purified by column chromatography (10 g Sfär Silica D; 35 mL/min; 100% cyclohexane

(2 CV), followed by a linear gradient (14 CV): 0%→30% ethyl acetate in cyclohexane) to afford ethyl ester **53b** (402 mg, 87%).

White solid, m.p.: 70-71 °C; ^{1}H NMR (600 MHz, 300 K, $CDCl_3$): δ = 8.18 (d, *J* = 8.0 Hz, 1H), 7.65 (t, *J* = 8.0 Hz, 1H), 7.31 (d, *J* = 8.0 Hz, 1H), 7.22 (t, *J* = 8.0 Hz, 1H), 4.51 (q, *J* = 7.0 Hz, 2H), 4.31 (t, *J* = 7.5 Hz, 2H), 1.62 (q, *J* = 7.5 Hz, 2H), 1.48 (t, *J* = 7.0 Hz, 3H), 0.84 – 0.69 (m, 1H), 0.53 – 0.41 (m, 2H), 0.18 – 0.08 ppm (m, 2H); ^{13}C NMR (151 MHz, 300 K, $CDCl_3$): δ = 172.9, 171.7, 159.5, 140.9, 134.4, 126.0, 121.8, 115.2, 114.2, 98.0, 62.5, 42.4, 32.4, 14.4, 8.7, 4.4 ppm; IR (film): $\tilde{\nu}$ = 2981, 1661, 1624, 1562, 1415, 1327, 1248, 1178, 1092, 1018 cm^{-1}; HRMS (ESI): *m/z* calcd for $C_{17}H_{20}O_4N$ [*M*+H]$^+$: 302.1387, found: 302.1385.

tert-Butyl (1-(2-cyclopropylethyl)-4-hydroxy-2-oxo-1,2-dihydroquinoline-3-carbonyl)glycinate (53c)

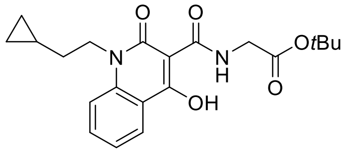

According to General Procedure A, *tert*-butyl ester **53c** (275 mg, 88%) was obtained from ethyl ester **53b** (245 mg, 0.81 mmol) and glycine *tert*-butyl ester hydrochloride (157 mg, 0.94 mmol), following column chromatography (10 g Sfär Silica D; 35 mL/min; 100% cyclohexane (2 CV), followed by a linear gradient (14 CV): 0%→10% ethyl acetate in cyclohexane).

White solid, m.p. 151-153 °C; ^{1}H NMR (600 MHz, 300 K, $CDCl_3$): δ = 10.78 (t, *J* = 5.5 Hz, 1H), 8.21 (d, *J* = 8.0 Hz, 1H), 7.66 (t, *J* = 8.0 Hz, 1H), 7.37 (d, *J* = 8.0 Hz, 1H), 7.27 (t, *J* = 8.0 Hz, 1H), 4.37 (t, *J* = 7.5 Hz, 2H), 4.12 (d, *J* = 5.5 Hz, 2H), 1.64 (q, *J* = 7.5 Hz, 2H), 1.50 (s, 9H), 0.81 – 0.76 (m, 1H), 0.54 – 0.43 (m, 2H), 0.15 – 0.08 ppm (m, 2H); ^{13}C NMR (151 MHz, 300 K, $CDCl_3$): δ = 171.7, 171.3, 168.5, 162.5, 139.6, 133.8, 125.8, 122.2, 116.4, 114.4, 97.0, 82.4, 42.3, 42.0, 32.7, 28.2, 8.7, 4.4 ppm; IR (film): $\tilde{\nu}$ = 2981, 1738, 1633, 1563, 1407, 1368, 1227, 1155 cm^{-1}; HRMS (ESI): *m/z* calcd for $C_{21}H_{27}O_5N_2$ [*M*+H]$^+$: 387.1914, found: 387.1914.

(1-(2-Cyclopropylethyl)-4-hydroxy-2-oxo-1,2-dihydroquinoline-3-carbonyl)glycine (53)[32]

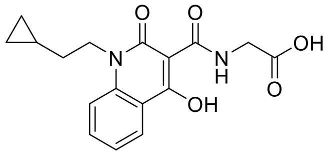

According to General Procedure D, carboxylic acid **53** (81 mg, 60%) was obtained from *tert*-butyl ester **53d** (137 mg, 0.41 mmol), following reverse-phase column chromatography (12 g Sfär C18 Duo; 12 mL/min; water (+ 0.1%$_{v/v}$ formic acid) (4 CV), followed by a linear gradient (25 CV): 0%→100% acetonitrile (+ 0.1%$_{v/v}$ formic acid) in water (+ 0.1%$_{v/v}$ formic acid)). The analytical data of carboxylic acid **53** are consistent with those reported.[32]

White solid, m.p. 206-208 °C; ^{1}H NMR (600 MHz, 300 K, DMSO-d_6): δ = 12.92 (s, 1H), 10.57 (t, *J* = 5.5 Hz, 1H), 8.10 (d, *J* = 8.0 Hz, 1H), 7.81 (t, *J* = 8.5 Hz, 1H), 7.67 (d, *J* = 8.5 Hz, 1H), 7.36 (t, *J* = 8.0 Hz, 1H), 4.35 (t, *J* = 7.5 Hz, 2H), 4.12 (d, *J* = 5.5 Hz, 2H), 1.54 (q, *J* = 7.5 Hz, 2H), 0.84 – 0.77 (m, 1H), 0.48 – 0.32 (m,

2H), 0.13 – 0.01 ppm (m, 2H); ^{13}C NMR (151 MHz, 300 K, DMSO-d_6): δ = 171.0, 170.7, 170.4, 161.3, 139.1, 134.4, 124.7, 122.4, 115.3, 115.1, 95.9, 41.5, 41.0, 32.0, 8.2, 4.1 ppm; IR (film): $\tilde{\nu}$ = 3212, 2981, 1738, 1628, 1536, 1403, 1152 cm^{-1}; HRMS (ESI): *m/z* calcd for $C_{17}H_{19}O_5N_2$ [*M*+H]$^+$: 331.1289, found: 331.1283.

Ethyl 3-((*tert*-butoxycarbonyl)(cyclopropylmethoxy)amino)propanoate (54a)

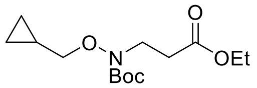

To a solution of *tert*-butyl (cyclopropylmethoxy)carbamate **3**[23, 24] (937 mg, 5.0 mmol, 2.0 equiv.) and tetrabutylammonium bromide (TBAB; 81 mg, 0.25 mmol, 0.1 equiv.) in toluene (10 mL; HPLC grade) and water (1.5 mL; Milli-Q® Ultrapure grade) were sequentially added KOH (168 mg, 3.0 mmol, 1.2 equiv.) and ethyl acrylate (0.27 mL, 2.5 mmol, 1.0 equiv.) under an ambient atmosphere at room temperature. The reaction mixture was stirred at room temperature for 14 h, before being diluted with ethyl acetate and washed with brine. The organic layer was dried over anhydrous Na_2SO_4, filtered, and evaporated. The crude residue was purified using column chromatography (10 g Sfär Silica D; 35 mL/min; 100% cyclohexane (2 CV), followed by a linear gradient (14 CV): 0%→20% acetone in cyclohexane) to afford ethyl ester **54a** (517 mg, 72%).

Colorless oil; ^1H NMR (500 MHz, 300 K, $CDCl_3$): δ = 4.14 (q, *J* = 7.0 Hz, 2H), 3.76 (t, *J* = 7.0 Hz, 2H), 3.64 (d, *J* = 7.5 Hz, 2H), 2.61 (t, *J* = 7.0 Hz, 2H), 1.48 (s, 9H), 1.25 (t, *J* = 7.0 Hz, 3H), 1.14 – 0.99 (m, 1H), 0.63 – 0.51 (m, 2H), 0.30 – 0.20 ppm (m, 2H); ^{13}C NMR (126 MHz, 300 K, $CDCl_3$): δ = 171.9, 156.5, 81.6, 79.8, 60.7, 45.6, 32.4, 28.4, 14.3, 9.3, 3.3 ppm; IR (film): $\tilde{\nu}$ = 2980, 1734, 1704, 1368, 1252, 1161, 1252, 1161, 1096, 1024 cm^{-1}; HRMS (ESI): *m/z* calcd for $C_{14}H_{25}O_5NNa$ [*M*+Na]$^+$: 310.1625, found: 310.1623.

Ethyl 3-((cyclopropylmethoxy)amino)propanoate hydrochloride (54b)

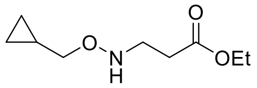

To *N*-Boc protected hydroxylamine **54a** (450 mg, 1.6 mmol, 1.0 equiv.) under an atmosphere of argon gas at 0 °C was added a solution of HCl in dioxane (4M; 4 mL, 16 mmol, 10.0 equiv.). The reaction mixture was allowed to warm to ambient temperature and stirred for 1 h before removal of the solvent under reduced pressure to afford hydroxylamine **54b** (300 mg, 99%), which was used in the subsequent reaction without further purification.

Colorless oil; ^1H NMR (500 MHz, 300 K, $CDCl_3$): δ = 12.11 (s, 1H), 4.16 – 4.06 (m, 4H), 3.63 – 3.53 (m, 2H), 2.96 – 2.91 (m, 2H), 1.22 (t, *J* = 7.0 Hz, 3H), 1.17 – 1.01 (m, 1H), 0.64 – 0.58 (m, 2H), 0.38 – 0.32 ppm (m, 2H); ^{13}C NMR (126 MHz, 300 K, $CDCl_3$): δ = 169.8, 79.6, 61.4, 44.9, 28.7, 14.1, 8.8, 3.6 ppm; IR (film): $\tilde{\nu}$ = 3417, 2983, 2938, 1734, 1468, 1379, 1258, 1201, 1028 cm^{-1}; HRMS (ESI): *m/z* calcd for $C_9H_{18}O_3N$ [*M*+H]$^+$: 188.1281, found: 188.1279.

Ethyl 3-((cyclopropylmethoxy)(3-ethoxy-3-oxopropyl)amino)-3-oxopropanoate (54c)

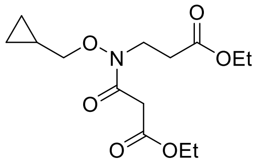

To a solution of hydroxylamine **54b** (268 mg, 1.2 mmol, 1.0 equiv.) in anhydrous ethyl acetate (5 mL) were sequentially added triethylamine (0.40 mL, 2.9 mmol, 2.0 equiv.) and ethyl malonyl chloride (0.28 mL, 2.2 mmol, 1.5 equiv.) at 0 °C under an a N_2 atmosphere. The reaction mixture was stirred at ambient temperature overnight (14 h) before being diluted with ethyl acetate (30 mL) and being washed with H_2O, then brine. The organic solution was dried over anhydrous Na_2SO_4, filtered, evaporated, and purified by column chromatography (10 g Sfär Silica D; 35 mL/min; 100% cyclohexane (2 CV), followed by a linear gradient (20 CV): 0%→50% ethyl acetate in cyclohexane) to afford malonate **54c** (305 mg, 83%).

Yellow oil; ^{1}H NMR (600 MHz, 300 K, $CDCl_3$): δ = 4.26 – 4.07 (m, 4H), 3.93 (t, *J* = 7.0 Hz, 2H), 3.65 (d, *J* = 7.5 Hz, 2H), 3.48 (s, 2H), 2.61 (t, *J* = 7.0 Hz, 2H), 1.29 – 1.21 (m, 6H), 1.07 – 1.02 (m, 1H), 0.69 – 0.55 (m, 2H), 0.29 – 0.26 ppm (m, 2H); ^{13}C NMR (151 MHz, 300 K, $CDCl_3$): δ = 171.7, 168.4, 167.3, 79.2, 61.4, 60.8, 41.4, 40.9, 31.8, 14.3, 14.2, 9.0, 3.5 ppm; IR (film): $\tilde{\nu}$ = 2981, 1736, 1673, 1184, 1029 cm^{-1}; HRMS (ESI): *m/z* calcd for $C_{14}H_{24}O_6N$ [*M*+H]$^+$: 302.1598, found: 302.1596.

Ethyl 1-(cyclopropylmethoxy)-4-hydroxy-2-oxo-1,2,5,6-tetrahydropyridine-3-carboxylate (54d)

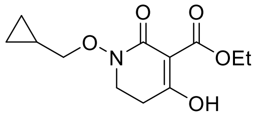

To malonate **54c** (155 mg, 0.51 mmol, 1.0 equiv.) was added sodium ethoxide (21 $\%_{w/w}$ in ethanol; 0.42 mL, 1.1 mmol, 2.2 equiv.) under a N_2 atmosphere at 0 °C. The reaction mixture was stirred at ambient temperature for 1 h before the pH was neutralized (pH 6-7) with the dropwise addition of aqueous HCl solution (4M). The solvent was removed under reduced pressure and the crude residue was redissolved in ethyl acetate and washed with H_2O, then brine. The organic solution was dried over anhydrous Na_2SO_4, filtered and evaporated to afford ethyl ester **54d** (80 mg, 61%), which was used in the subsequent reaction without further purification.

Colorless oil; ^{1}H NMR (400 MHz, 300 K, $CDCl_3$): δ = 4.37 (q, *J* = 7.0 Hz, 2H), 3.80 (d, *J* = 7.0 Hz, 2H), 3.65 (t, *J* = 7.0 Hz, 2H), 2.83 (t, *J* = 7.0 Hz, 2H), 1.39 (t, *J* = 7.0 Hz, 3H), 1.21 – 1.08 (m, 1H), 0.63 – 0.51 (m, 2H), 0.38 – 0.26 ppm (m, 2H); ^{13}C NMR (151 MHz, 300 K, $CDCl_3$): δ = 183.5, 172.1, 163.9, 97.5, 80.0, 62.1, 46.0, 30.5, 14.3, 9.6, 3.2 ppm; IR (film): $\tilde{\nu}$ = 2928, 1681, 1594, 1476, 1424, 1327, 1262, 1226 cm^{-1}; HRMS (ESI): *m/z* calcd for $C_{12}H_{18}O_5N$ [*M*+H]$^+$: 256.1179, found: 256.1178.

Methyl (1-(cyclopropylmethoxy)-4-hydroxy-2-oxo-1,2,5,6-tetrahydropyridine-3-carbonyl)glycinate (54e)

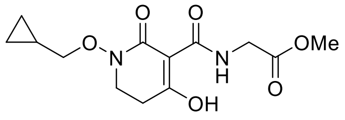

According to General Procedure A, methyl ester **54e** (45 mg, 60%) was obtained from ethyl ester **54d** (62 mg, 0.25 mmol) and glycine methyl ester hydrochloride (35 mg, 0.28 mmol), following column chromatography (10 g Sfär Silica D; 35 mL/min; 100% cyclohexane (2 CV), followed by a linear gradient (14 CV): 0%→30% acetone in cyclohexane).

Colorless oil; ^{1}H NMR (600 MHz, 300 K, $CDCl_3$): δ = 9.95 (t, *J* = 6.0 Hz, 1H), 4.11 (d, *J* = 6.0 Hz, 2H), 3.84 – 3.74 (m, 5H), 3.65 (t, *J* = 7.0 Hz, 2H), 2.81 (t, *J* = 7.0 Hz, 2H), 1.20 – 1.13 (m, 1H), 0.69 – 0.53 (m, 2H), 0.34 – 0.31 ppm (m, 2H); ^{13}C NMR (151 MHz, 300 K, $CDCl_3$): δ = 184.9, 171.2, 169.5, 168.5, 94.7, 80.1, 52.6, 46.2, 40.9, 30.9, 9.6, 3.2 ppm; IR (film): $\tilde{\nu}$ = 3247, 2981, 1752, 1652, 1556, 1413, 1216 cm^{-1}; HRMS (ESI): *m/z* calcd for $C_{13}H_{19}O_6N_2$ [*M*+H]$^+$: 299.1238, found: 299.1240.

(1-(Cyclopropylmethoxy)-4-hydroxy-2-oxo-1,2,5,6-tetrahydropyridine-3-carbonyl)glycine (54)

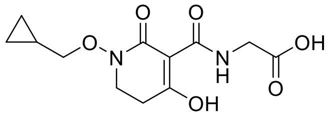

According to General Procedure B, carboxylic acid **54** (18 mg, 42%) was obtained from methyl ester **54e** (45 mg, 0.15 mmol), following reverse-phase column chromatography (12 g Sfär C18 Duo; 12 mL/min; water (+ 0.1%$_{v/v}$ formic acid) (4 CV), followed by a linear gradient (25 CV): 0%→100% acetonitrile (+ 0.1%$_{v/v}$ formic acid) in water (+ 0.1%$_{v/v}$ formic acid)).

White solid, m.p.: 138-140 °C; ^{1}H NMR (600 MHz, 300 K, DMSO-*d*$_6$): δ = 12.87 (s, 1H), 9.80 (t, *J* = 5.5 Hz, 1H), 4.01 (d, *J* = 5.5 Hz, 2H), 3.71 (d, *J* = 7.0 Hz, 2H), 3.61 (d, *J* = 7.0 Hz, 2H), 2.84 (t, *J* = 7.0 Hz, 2H), 1.06 – 1.02 (m, 1H), 0.60 – 0.46 (m, 2H), 0.37 – 0.18 ppm (m, 2H); ^{13}C NMR (151 MHz, 300 K, DMSO-*d*$_6$): δ = 185.7, 170.6, 170.3, 166.7, 93.4, 78.5, 45.3, 40.7, 30.3, 9.3, 3.0 ppm; IR (film): $\tilde{\nu}$ = 2981, 1715, 1656, 1629, 1549, 1397, 1263, 1153 cm^{-1}; HRMS (ESI): *m/z* calcd for $C_{12}H_{16}O_6N_2Na$ [*M*+Na]$^+$: 307.0901, found: 307.0900.

Ethyl 2-(1-(benzyloxy)-4-hydroxy-2-oxo-1,2-dihydroquinoline-3-carboxamido)-2-methylpropanoate (55)

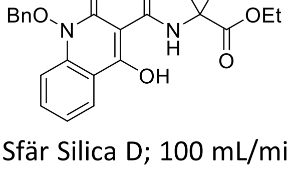

According to General Procedure A, ethyl ester **55** (2.12 g, 83%) was prepared from ester **37** (2.04 g, 6.0 mmol) and ethyl 2-amino-2-methylpropanoate hydrochloride (1.16 g, 6.9 mmol), following column chromatography (50 g Sfär Silica D; 100 mL/min; 100% cyclohexane (2 CV), followed by a linear gradient (14 CV): 0%→15% ethyl acetate in cyclohexane).

Colorless oil; ^{1}H NMR (600 MHz, 300 K, $CDCl_3$): δ = 10.46 (s, 1H), 8.17 (dd, *J* = 8.0, 1.5 Hz, 1H), 7.65 (td, *J* = 8.5, 1.5 Hz, 1H), 7.61 – 7.57 (m, 2H), 7.56 – 7.52 (m, 1H), 7.45 – 7.38 (m, 3H), 7.29 (t, *J* = 8.0 Hz, 1H), 5.24 (s, 2H), 4.24 (q, *J* = 7.0 Hz, 2H), 1.68 (s, 6H), 1.27 ppm (t, *J* = 7.0 Hz, 3H); ^{13}C NMR (151 MHz, 300 K, $CDCl_3$): δ = 174.0, 171.5, 170.2, 159.3, 138.3, 134.2, 133.9, 129.8, 129.4, 128.9, 125.4, 123.1, 115.2, 112.4, 97.4, 77.8, 61.6, 56.5, 25.3, 14.3 ppm; IR (film): $\tilde{\nu}$ = 3233, 2986, 1740, 1651, 1629, 1558, 1408, 1291, 1219, 1150, 1015 cm^{-1}; HRMS (ESI): *m/z* calcd for $C_{23}H_{25}O_6N_2$ [*M*+H]$^+$: 425.1707, found: 425.1696.

Ethyl 2-(1,4-dihydroxy-2-oxo-1,2-dihydroquinoline-3-carboxamido)-2-methylpropanoate (56)

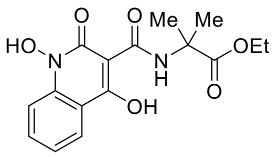

N_2 gas was bubbled through a solution of ester **55** (2.01 g, 4.7 mmol) in anhydrous methanol (40 mL) for 15 min. Palladium on charcoal (10%$_{w/w}$ palladium) was added to the solution and H_2 gas was bubbled through the black suspension for 15 min. The reaction mixture was stirred for 30 min at ambient temperature under an atmosphere of H_2 (1 atm) before being filtered through Celite®, washed with methanol, and evaporated. The crude residue was purified by column chromatography (50 g Sfär Silica D; 100 mL/min; 100% cyclohexane (2 CV), followed by a linear gradient (20 CV): 0%→30% acetone in cyclohexane) to afford ester **56** (630 mg, 40%).

White solid, m.p.: 114-117 °C; ^{1}H NMR (500 MHz, 300 K, DMSO-*d*$_6$): δ = 11.41 (s, 1H), 10.64 (s, 1H), 8.07 (dd, *J* = 8.0, 1.5 Hz, 1H), 7.84 (td, *J* = 8.0, 1.5 Hz, 1H), 7.74 (d, *J* = 8.5 Hz, 1H), 7.44 – 7.32 (m, 1H), 4.14 (q, *J* = 7.0 Hz, 2H), 1.57 (s, 6H), 1.17 ppm (t, *J* = 7.0 Hz, 3H); ^{13}C NMR (126 MHz, 300 K, DMSO-*d*$_6$): δ = 172.9, 169.7, 169.4, 158.3, 138.7, 134.7, 124.2, 122.8, 113.5, 112.9, 96.3, 60.9, 55.8, 24.6, 13.9 ppm; IR (film): $\tilde{\nu}$ = 3219, 2988, 2925, 1741, 1626, 1562, 1469, 1409, 1342, 1284, 1180, 1154 cm^{-1}; HRMS (ESI): *m/z* calcd for $C_{16}H_{19}O_6N_2$ [*M*+H]$^+$: 335.1238, found: 335.1232.

Ethyl 2-(4-hydroxy-2-oxo-1-(pyridin-2-ylmethoxy)-1,2-dihydroquinoline-3-carboxamido)-2-methylpropanoate (57a)

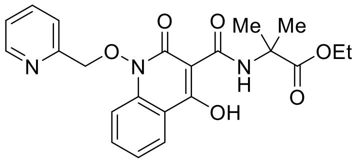

According to General Procedure C, pyridine **57a** (41 mg, 40%) was obtained from ester **56** (80 mg, 0.24 mmol) and pyridin-2-ylmethanol (39 mg, 0.36 mmol), following column chromatography (10 g Sfär Silica D; 35 mL/min; 100% cyclohexane (2 CV), followed by a linear gradient (14 CV): 0%→25% acetone in cyclohexane).

White solid, mp: 119-121 °C; ^{1}H NMR (600 MHz, 300 K, $CDCl_3$): δ = 10.40 (s, 1H), 8.70 – 8.60 (m, 1H), 8.19 – 8.11 (m, 1H), 7.80 (td, *J* = 7.5, 1.5 Hz, 1H), 7.75 (d, *J* = 7.5 Hz, 1H), 7.70 – 7.63 (m, 2H), 7.36 – 7.30 (m, 1H), 7.28 (td, *J* = 8.0, 1.5 Hz, 1H), 5.38 (s, 2H), 4.23 (q, *J* = 7.0 Hz, 2H), 1.65 (s, 6H), 1.26 ppm

(t, J = 7.0 Hz, 3H); ^{13}C NMR (151 MHz, 300 K, $CDCl_3$): δ = 173.9, 171.6, 170.1, 159.2, 153.7, 149.4, 138.2, 137.4, 134.4, 125.3, 124.3, 124.0, 123.2, 115.1, 112.4, 97.3, 78.0, 61.5, 56.5, 25.2, 14.2 ppm; IR (film): $\tilde{\nu}$ = 3235, 2985, 2919, 1739, 1651, 1594, 1547, 1408, 1292, 1151, 1019 cm^{-1}; HRMS (ESI): *m/z* calcd for $C_{22}H_{24}O_6N_3$ [M+H]$^+$: 426.1660, found: 426.1652.

2-(4-Hydroxy-2-oxo-1-(pyridin-2-ylmethoxy)-1,2-dihydroquinoline-3-carboxamido)-2-methylpropanoic acid (57)

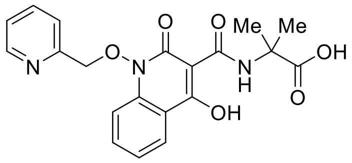

To a solution of pyridine **57a** (30 mg, 0.07 mmol, 1.0 equiv.) in methanol (1 mL, HPLC grade) was added an aqueous solution of lithium hydroxide (0.4 M; 0.44 mL, 0.17 mmol, 2.5 equiv.) under an ambient atmosphere at room temperature. The reaction mixture was heated at 60 °C for 72 h under an ambient atmosphere, before the methanol was removed under reduced pressure. The aqueous solution was washed three times with CH_2Cl_2 (the organic extracts were discarded); the pH of the aqueous phase was neutralized (pH~7) by the dropwise addition of an aqueous HCl solution (1 N) and the water was removed under reduced pressure. The crude residue was purified using reverse-phase column chromatography (12 g Sfär C18 Duo; 12 mL/min; water (+ 0.1%$_{v/v}$ formic acid) (4 CV), followed by a linear gradient (25 CV): 0%→100% acetonitrile (+ 0.1%$_{v/v}$ formic acid) in water (+ 0.1%$_{v/v}$ formic acid)) to afford carboxylic acid **57** (14 mg, 50%).

White solid, m.p.: 209-213 °C; ^{1}H NMR (600 MHz, 300 K, DMSO-d_6): δ = 12.96 (br s, 1H), 10.51 (s, 1H), 8.64 (dd, J = 5.0, 2.0 Hz, 1H), 8.09 (dd, J = 8.0, 1.5 Hz, 1H), 7.89 (td, J = 7.5, 2.0 Hz, 1H), 7.82 (td, J = 8.0, 1.5 Hz, 1H), 7.75 – 7.72 (m, 2H), 7.43 (ddd, J = 7.5, 5.0, 1.0 Hz, 1H), 7.39 (t, J = 7.5 Hz, 1H), 5.30 (s, 2H), 1.60 ppm (s, 6H); ^{13}C NMR (151 MHz, 300 K, DMSO-d_6): δ = 174.7, 170.8, 169.6, 158.2, 153.5, 149.5, 137.9, 137.1, 134.7, 124.6, 124.3, 124.0, 123.3, 114.1, 112.4, 96.6, 77.9, 56.1, 24.5 ppm; IR (film): $\tilde{\nu}$ = 3272, 3073, 2982, 1733, 1622, 1547, 1419, 1222, 1154, 1021 cm^{-1}; HRMS (ESI): *m/z* calcd for $C_{20}H_{20}O_6N_3$ [M+H]$^+$: 398.1347, found: 398.1338.

Ethyl 2-(4-hydroxy-2-oxo-1-(thiazol-4-ylmethoxy)-1,2-dihydroquinoline-3-carboxamido)-2-methylpropanoate (58a)

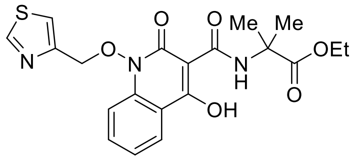

According to General Procedure C, thiazole **58a** (43 mg, 42%) was obtained from ethyl ester **56** (80 mg, 0.24 mmol) and thiazol-4-ylmethanol (41 mg, 0.36 mmol), following column chromatography (10 g Sfär Silica D; 35 mL/min; 100% cyclohexane (2 CV), followed by a linear gradient (14 CV): 0%→20% acetone in cyclohexane).

White solid, m.p.: 110-112 °C; ^{1}H NMR (600 MHz, 300 K, DMSO-d_6): δ = 10.46 (s, 1H), 9.17 (d, J = 2.0 Hz, 1H), 8.08 (dd, J = 8.0, 1.5 Hz, 1H), 8.04 (d, J = 2.0 Hz, 1H), 7.80 (td, J = 8.0, 1.5 Hz, 1H), 7.69 (d, J = 8.5 Hz, 1H), 7.43 – 7.35 (m, 1H), 5.39 (s, 2H), 4.16 (q, J = 7.0 Hz, 2H), 1.59 (s, 6H), 1.19 ppm (t, J = 7.0 Hz, 3H); ^{13}C NMR (151 MHz, 300 K, DMSO-d_6): δ = 172.8, 170.6, 169.6, 158.3, 155.1, 149.7, 138.1, 134.7, 124.5, 123.3, 122.2, 113.9, 112.5, 96.5, 71.6, 60.9, 55.9, 24.6, 14.0 ppm; IR (film): $\tilde{\nu}$ = 3082, 2987, 1741, 1652, 1562, 1415, 1342, 1293, 1179, 1153 cm^{-1}; HRMS (ESI): m/z calcd for $C_{20}H_{22}O_6N_3S$ [M+H]$^+$: 432.1224, found: 432.1218.

2-(4-Hydroxy-2-oxo-1-(thiazol-4-ylmethoxy)-1,2-dihydroquinoline-3-carboxamido)-2-methylpropanoic acid (58)

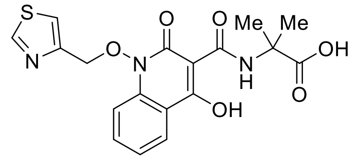

To a solution of thiazole **58a** (30 mg, 0.07 mmol, 1.0 equiv.) in methanol (1 mL, HPLC grade) was added an aqueous solution of lithium hydroxide (0.4 M; 0.44 mL, 0.17 mmol, 2.5 equiv.) under an ambient atmosphere at room temperature. The reaction mixture was heated at 60 °C for 72 h under an ambient atmosphere before the methanol was removed under reduced pressure. The aqueous solution was washed three times with CH_2Cl_2 (the organic extracts were discarded); the pH of the aqueous phase was neutralized (pH~7) by the dropwise addition of an aqueous HCl solution (1 N), and the water was removed under reduced pressure. The crude residue was purified using reverse-phase column chromatography (12 g Sfär C18 Duo; 12 mL/min; water (+ 0.1%$_{v/v}$ formic acid) (4 CV), followed by a linear gradient (25 CV): 0%→100% acetonitrile (+ 0.1%$_{v/v}$ formic acid) in water (+ 0.1%$_{v/v}$ formic acid)) to afford carboxylic acid **58** (16 mg, 57%).

White solid, m.p.: 201-204 °C; ^{1}H NMR (600 MHz, 300 K, DMSO-d_6): δ = 12.95 (br s, 1H), 10.53 (s, 1H), 9.17 (d, J = 2.0 Hz, 1H), 8.08 (dd, J = 8.0, 1.5 Hz, 1H), 8.05 (d, J = 2.0 Hz, 1H), 7.79 (td, J = 8.0, 1.5 Hz, 1H), 7.71 – 7.65 (m, 1H), 7.38 (td, J = 8.0, 1.0 Hz, 1H), 5.38 (s, 2H), 1.60 ppm (s, 6H); ^{13}C NMR (151 MHz, 300 K, DMSO-d_6): δ = 174.7, 170.7, 169.6, 158.3, 155.1, 149.7, 138.1, 134.6, 124.4, 123.2, 122.2, 114.0, 112.5, 96.6, 71.6, 56.1, 24.5 ppm; IR (film): $\tilde{\nu}$ = 3273, 3072, 2995, 1733, 1621, 1583, 1547, 1419, 1222, 1153, 1022 cm^{-1}; HRMS (ESI): m/z calcd for $C_{18}H_{18}O_6N_3S$ [M+H]$^+$: 404.0911, found: 404.0904.

6. ^{1}H and ^{13}C NMR spectra of novel compounds prepared for this study

(±)-Ethyl (1-(cyclopropylmethoxy)-4-hydroxy-2-oxo-1,2-dihydroquinoline-3-carbonyl)alaninate (8a)

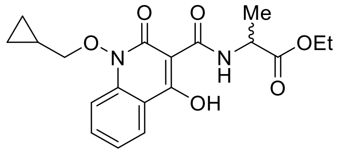

^{1}H NMR (600 MHz, 300 K, $CDCl_3$):

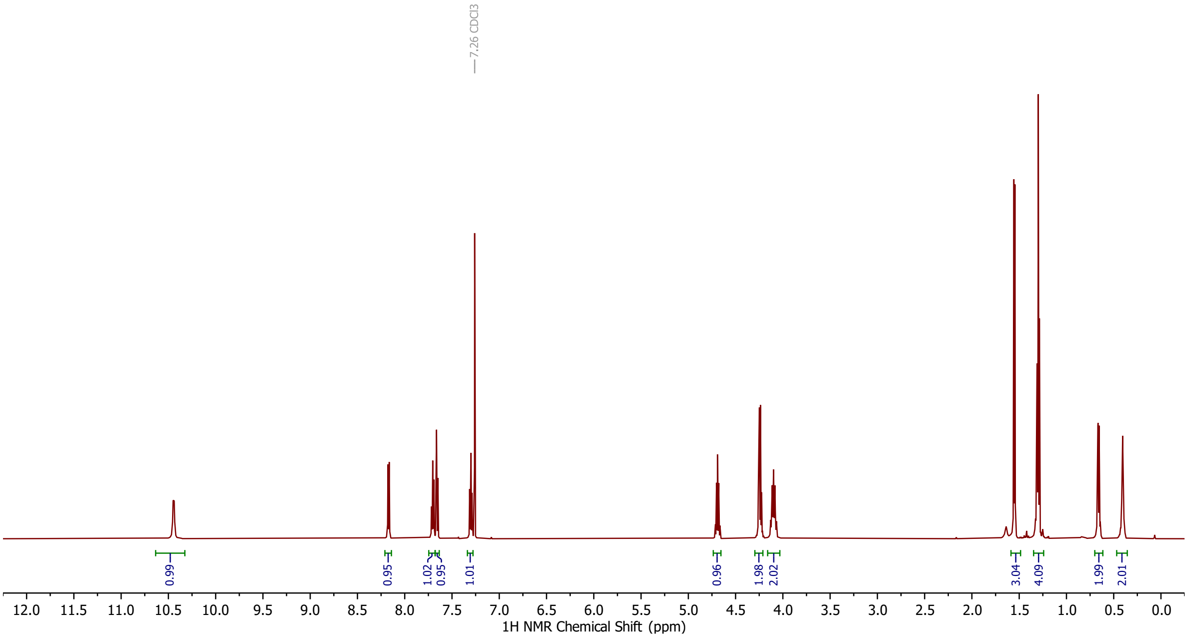

^{13}C NMR (151 MHz, 300 K, $CDCl_3$):

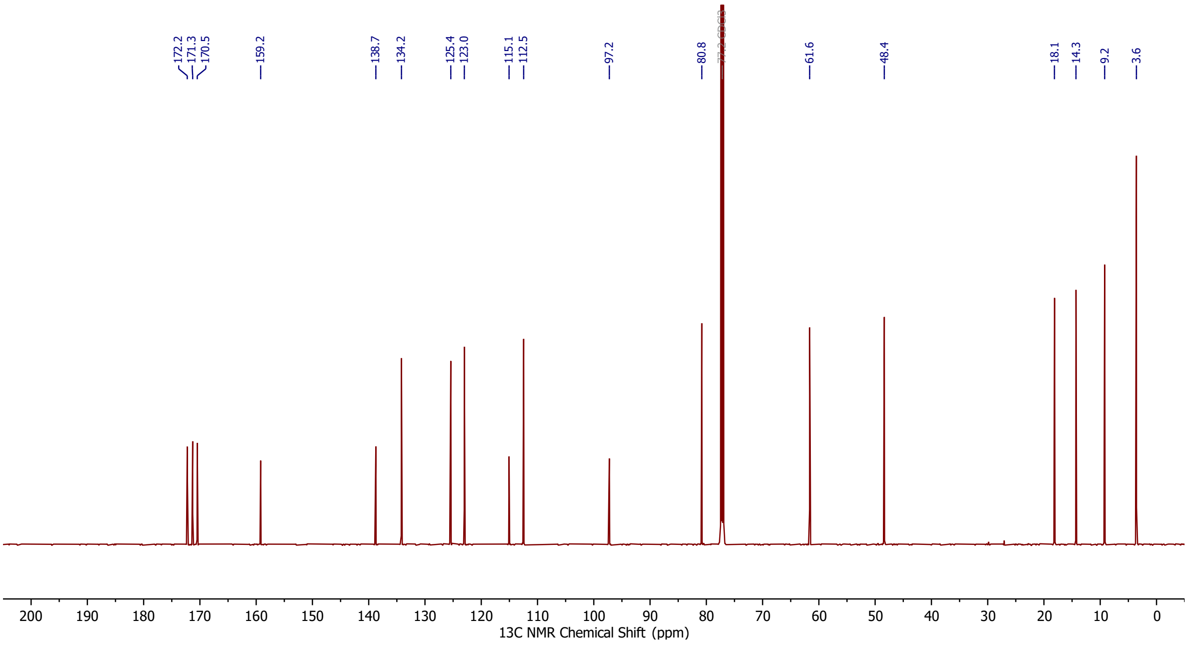

(±)-(1-(Cyclopropylmethoxy)-4-hydroxy-2-oxo-1,2-dihydroquinoline-3-carbonyl)alanine (8)

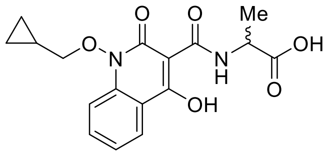

^{1}H NMR (600 MHz, 300 K, DMSO-d_6):

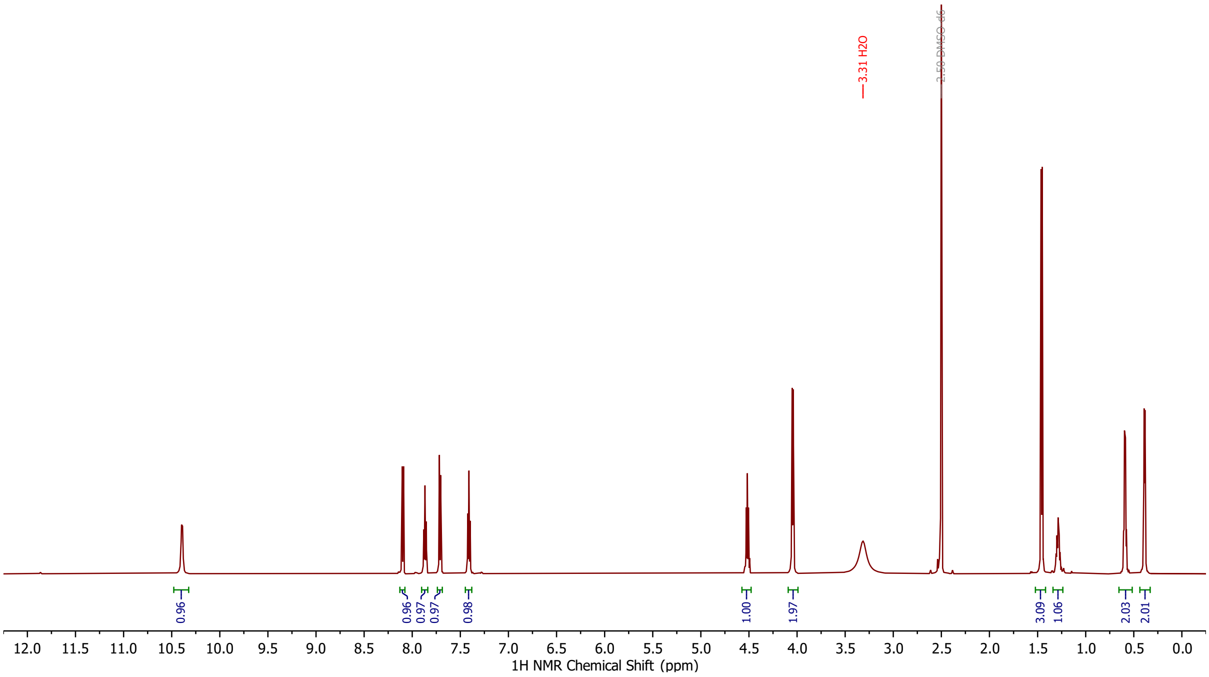

^{13}C NMR (151 MHz, 300 K, DMSO-d_6):

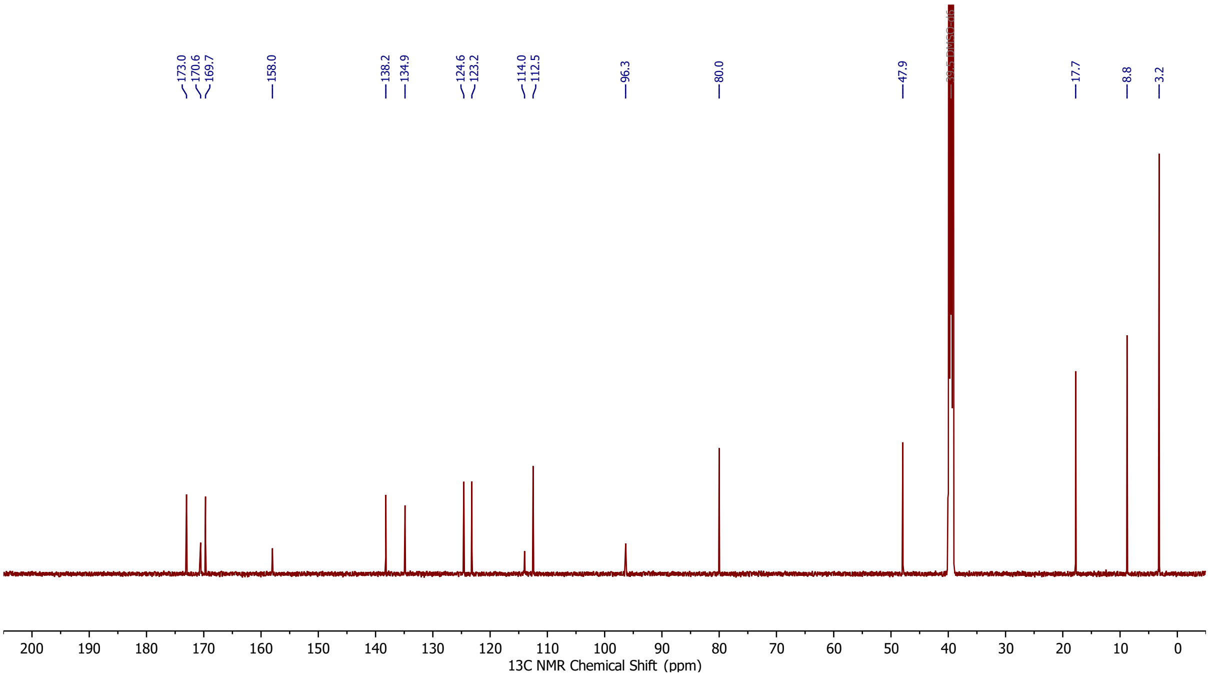

(±)-Methyl 2-(1-(cyclopropylmethoxy)-4-hydroxy-2-oxo-1,2-dihydroquinoline-3-carboxamido)-butanoate (9a)

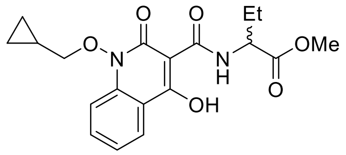

^{1}H NMR (500 MHz, 300 K, $CDCl_3$):

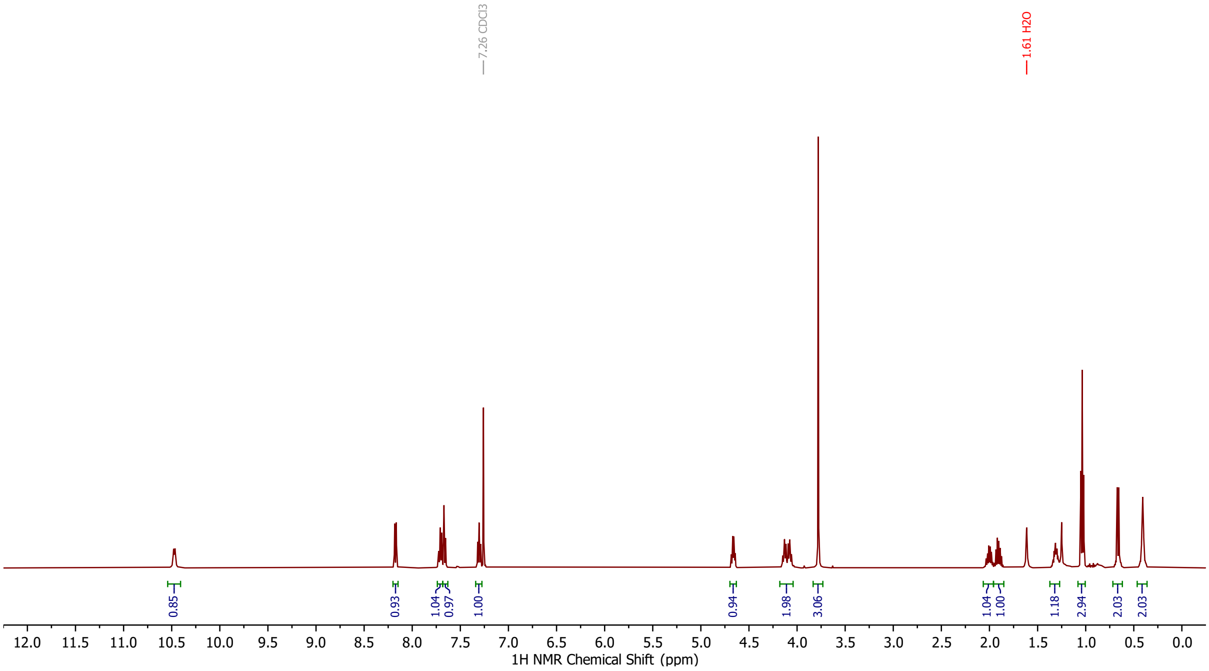

^{13}C NMR (126 MHz, 300 K, $CDCl_3$):

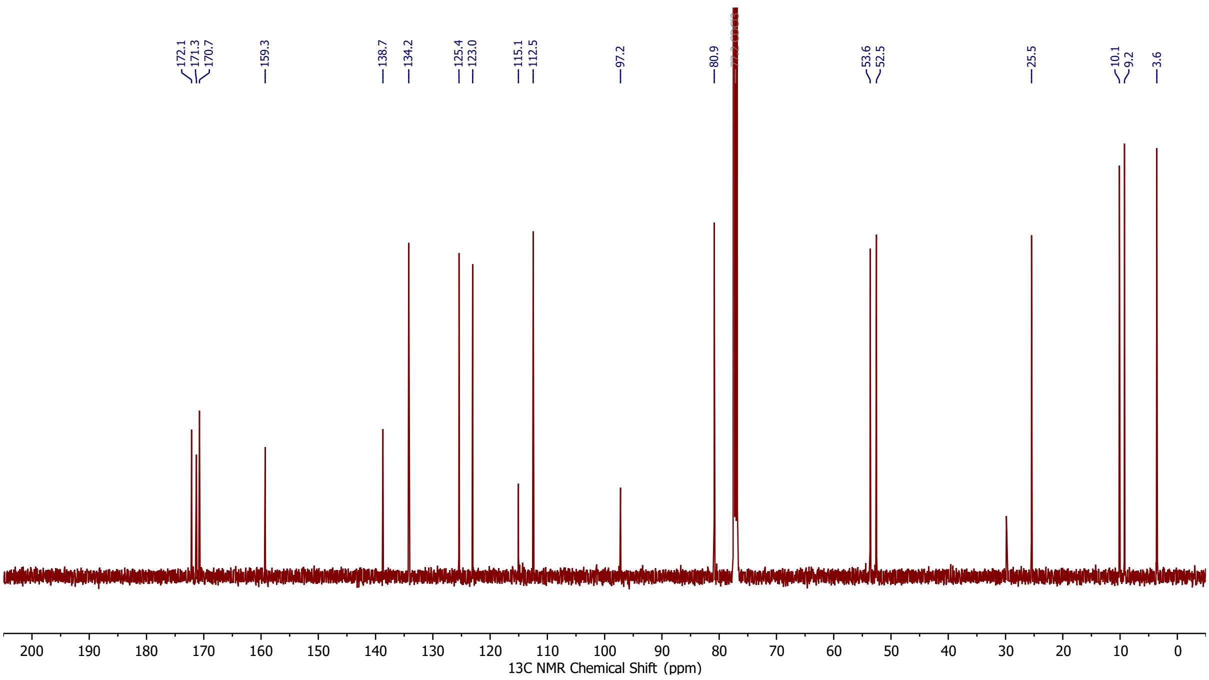

(±)-2-(1-(Cyclopropylmethoxy)-4-hydroxy-2-oxo-1,2-dihydroquinoline-3-carboxamido)butanoic acid (9)

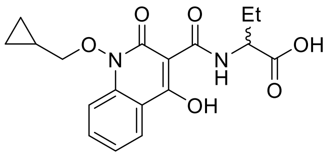

^{1}H NMR (500 MHz, 300 K, DMSO-d_6):

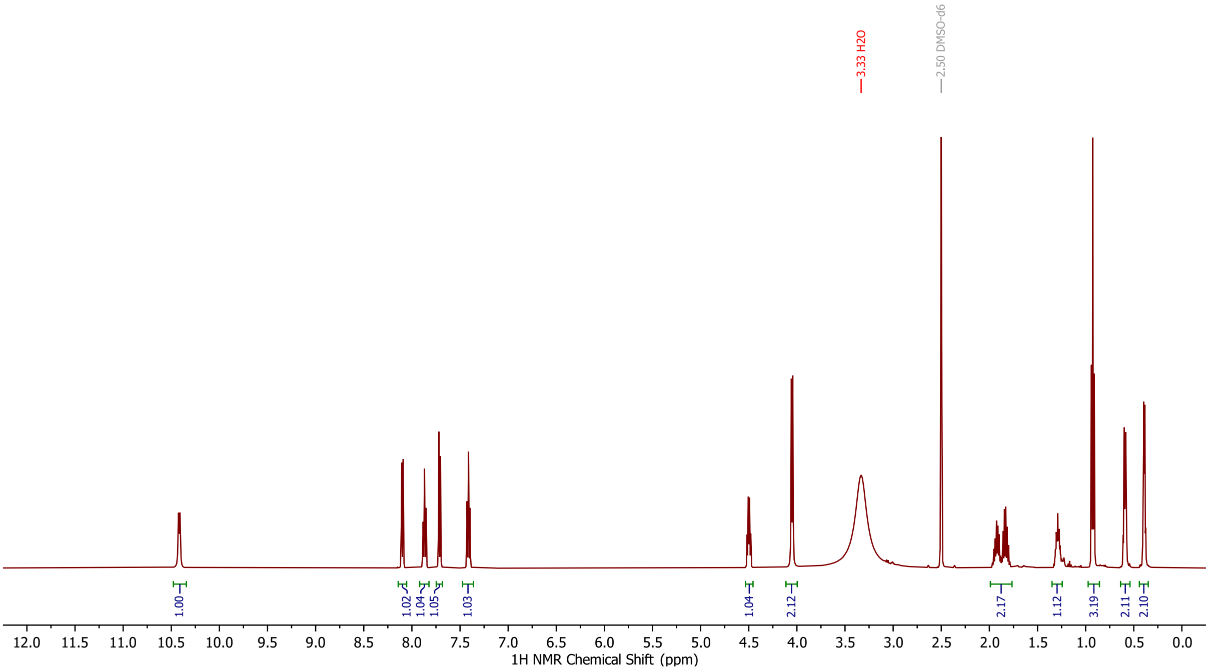

^{13}C NMR (126 MHz, 300 K, DMSO-d_6):

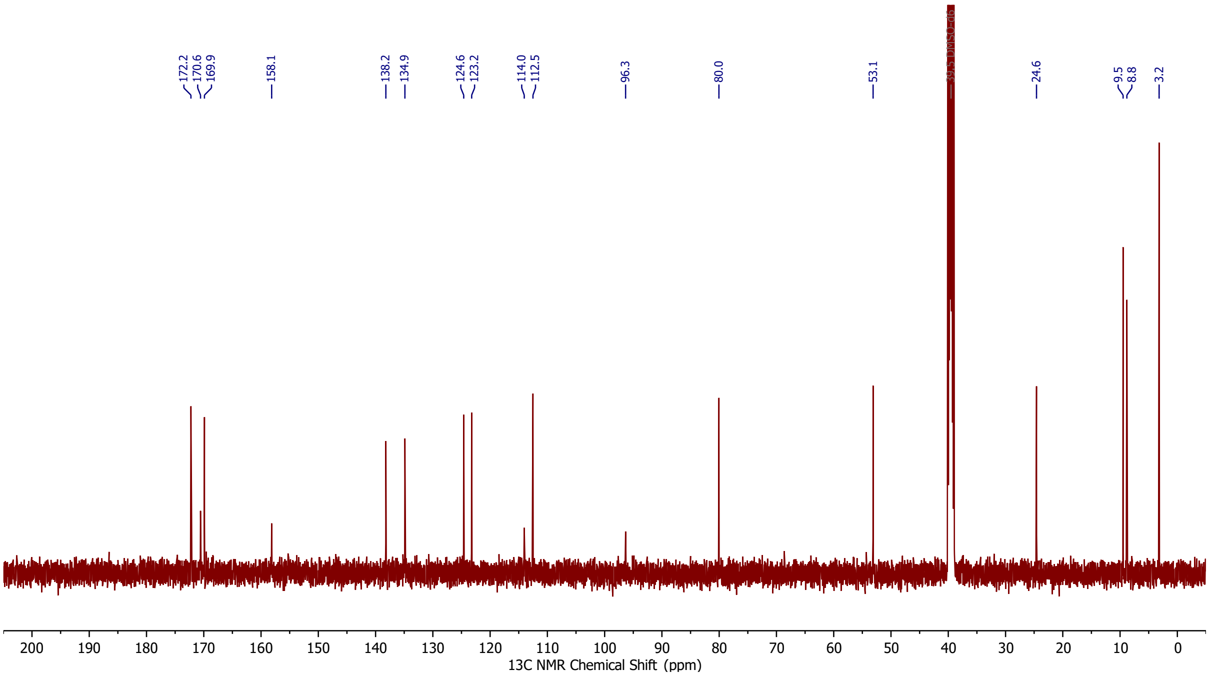

(±)-Methyl (1-(cyclopropylmethoxy)-4-hydroxy-2-oxo-1,2-dihydroquinoline-3-carbonyl)valinate (10a)

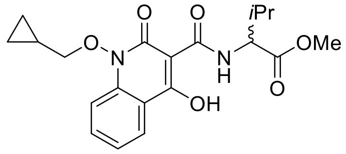

^{1}H NMR (500 MHz, 300 K, $CDCl_3$):

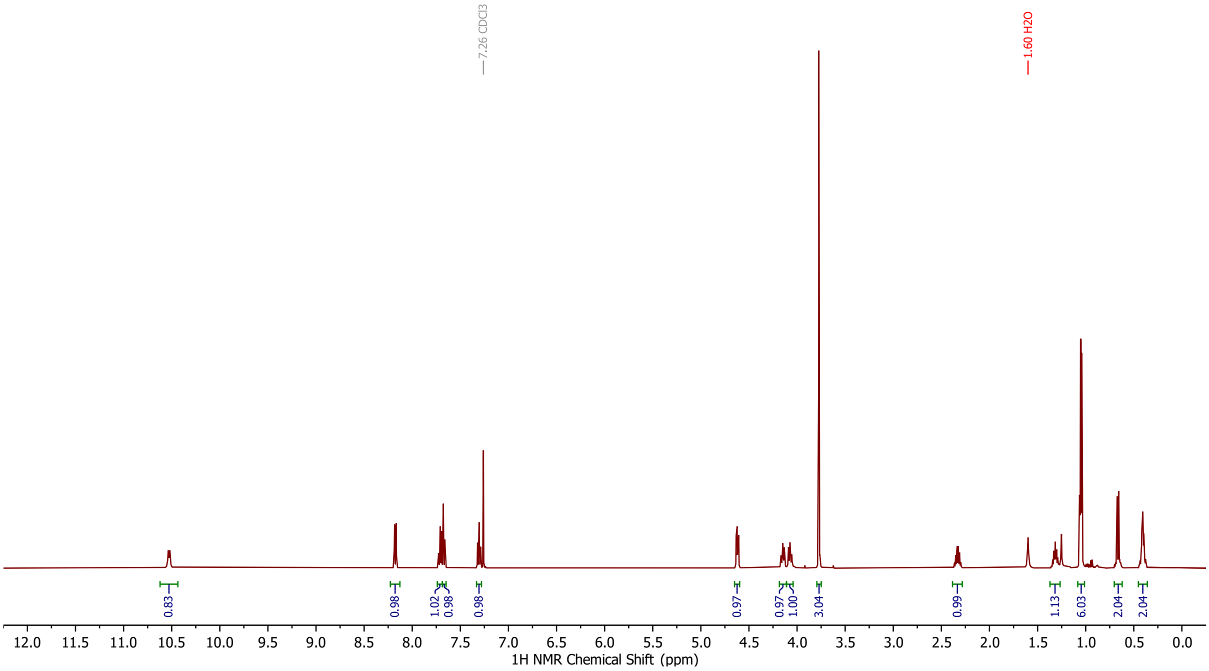

^{13}C NMR (126 MHz, 300 K, $CDCl_3$):

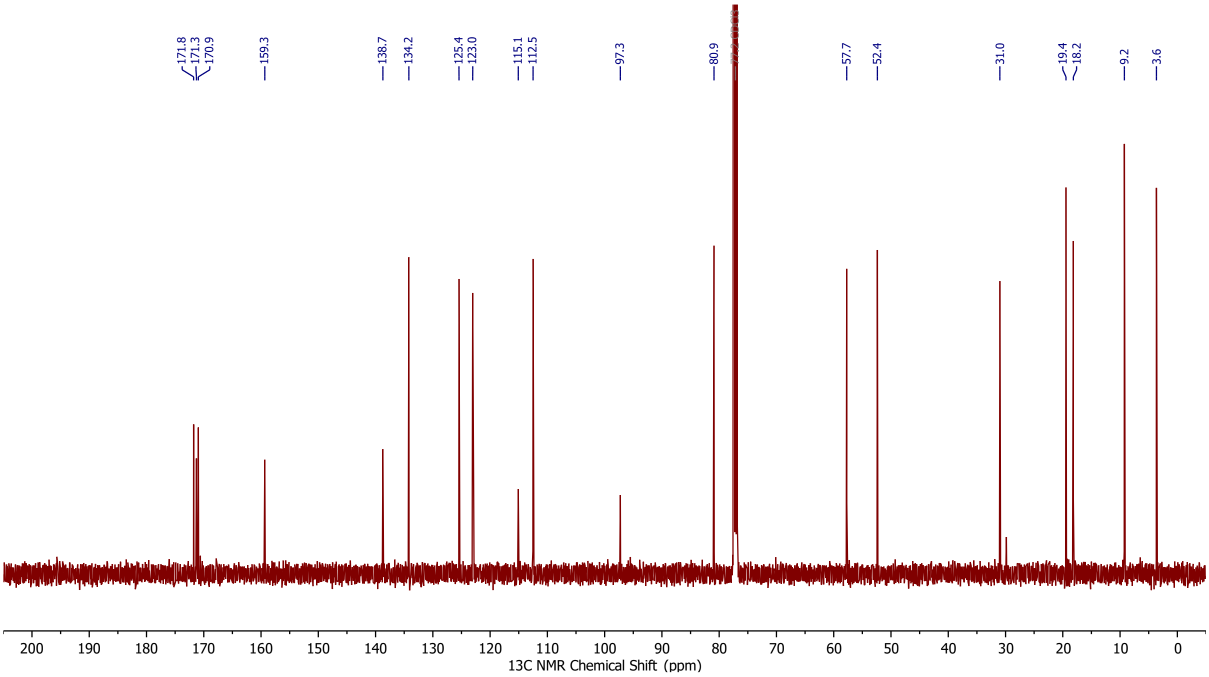

(±)-(1-(Cyclopropylmethoxy)-4-hydroxy-2-oxo-1,2-dihydroquinoline-3-carbonyl)valine (10)

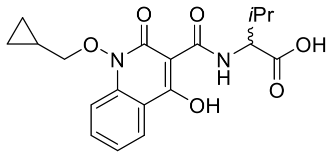

^{1}H NMR (600 MHz, 300 K, DMSO-d_6):

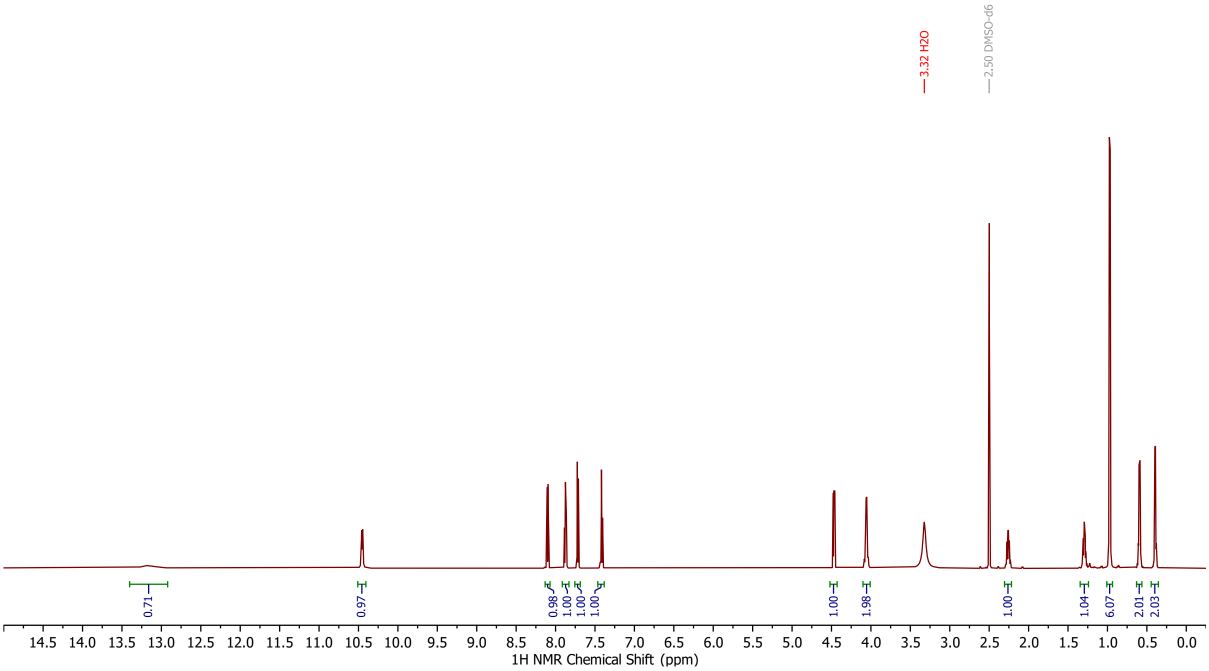

^{13}C NMR (151 MHz, 300 K, DMSO-d_6):

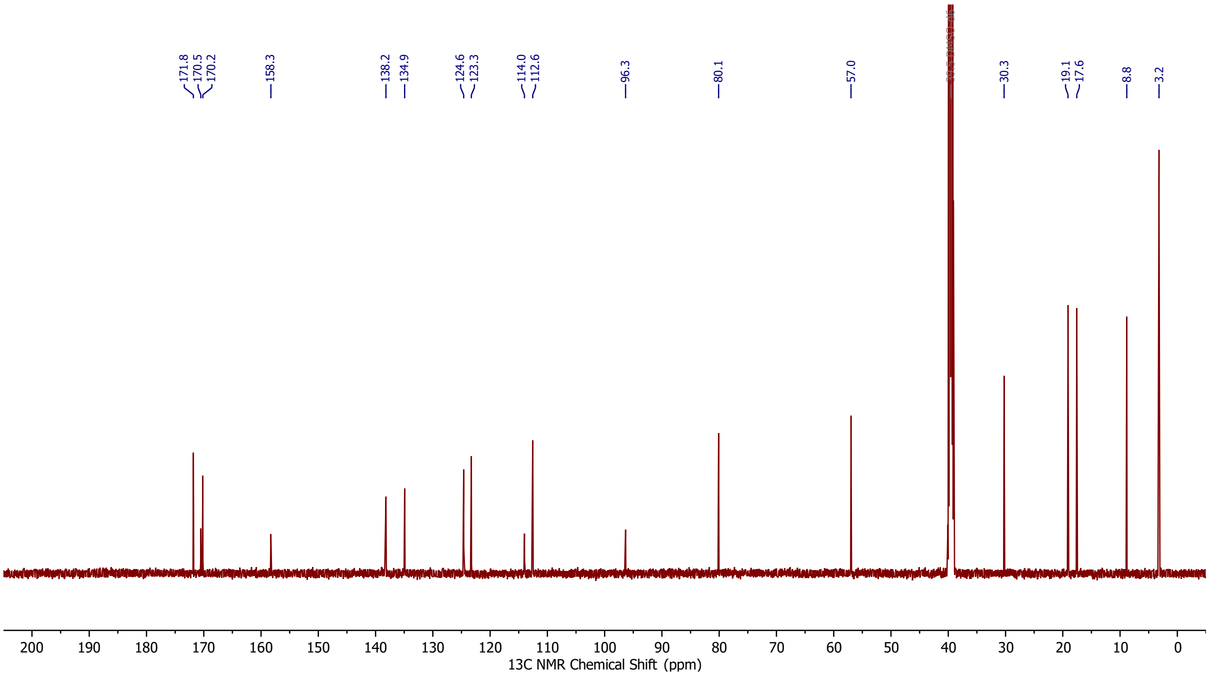

(±)-Methyl 2-(1-(cyclopropylmethoxy)-4-hydroxy-2-oxo-1,2-dihydroquinoline-3-carboxamido)-3,3,3-trifluoropropanoate (11a)

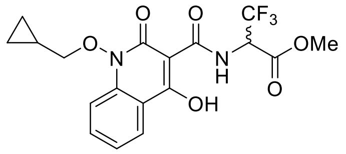

^{1}H NMR (500 MHz, 300 K, $CDCl_3$):

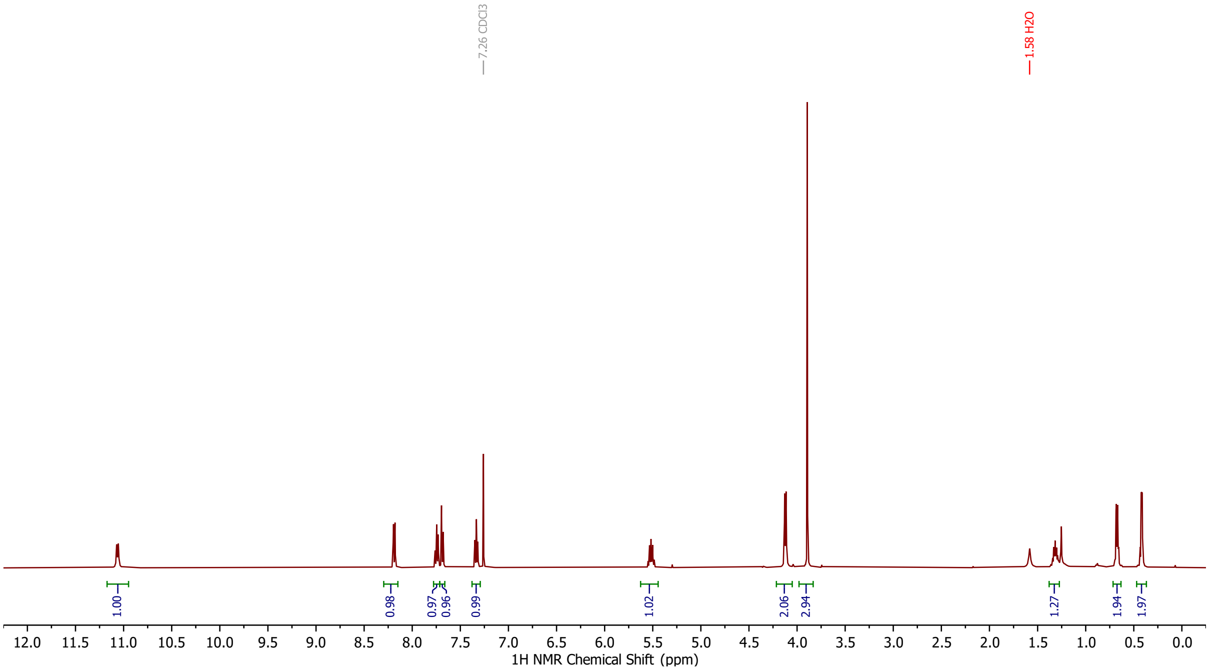

^{13}C NMR (126 MHz, 300 K, $CDCl_3$):

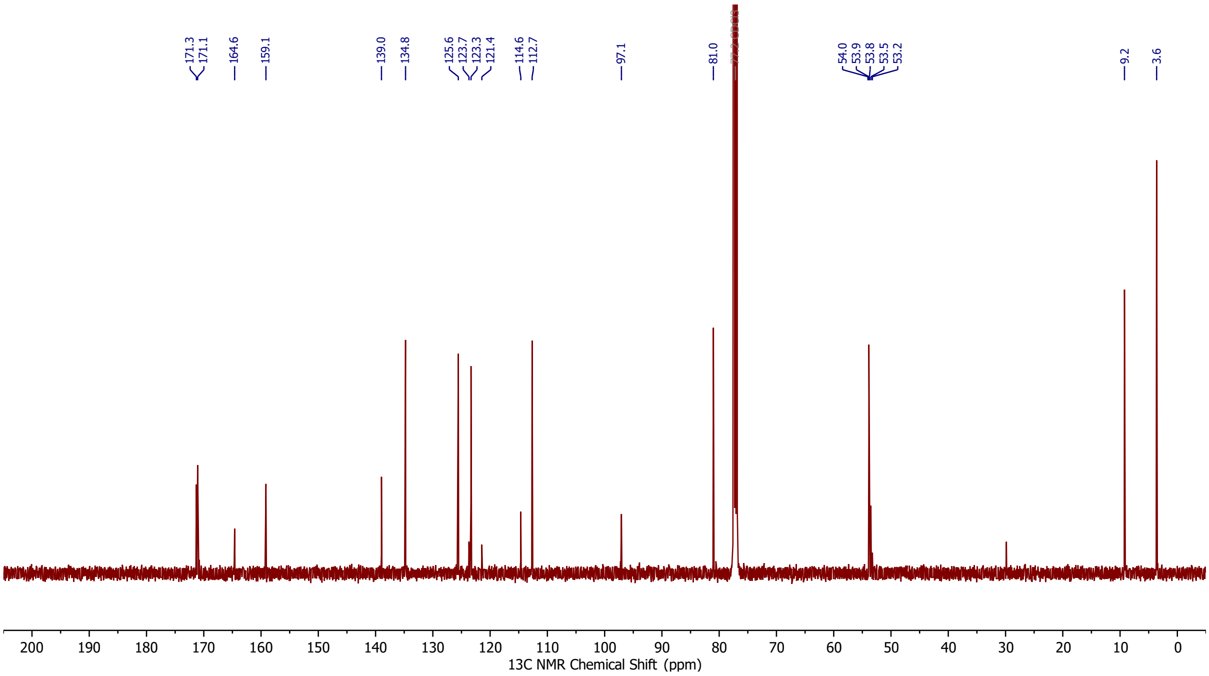

(±)-2-(1-(Cyclopropylmethoxy)-4-hydroxy-2-oxo-1,2-dihydroquinoline-3-carboxamido)-3,3,3-trifluoropropanoic acid (11)

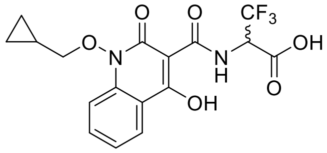

^{1}H NMR (600 MHz, 300 K, DMSO-d_6):

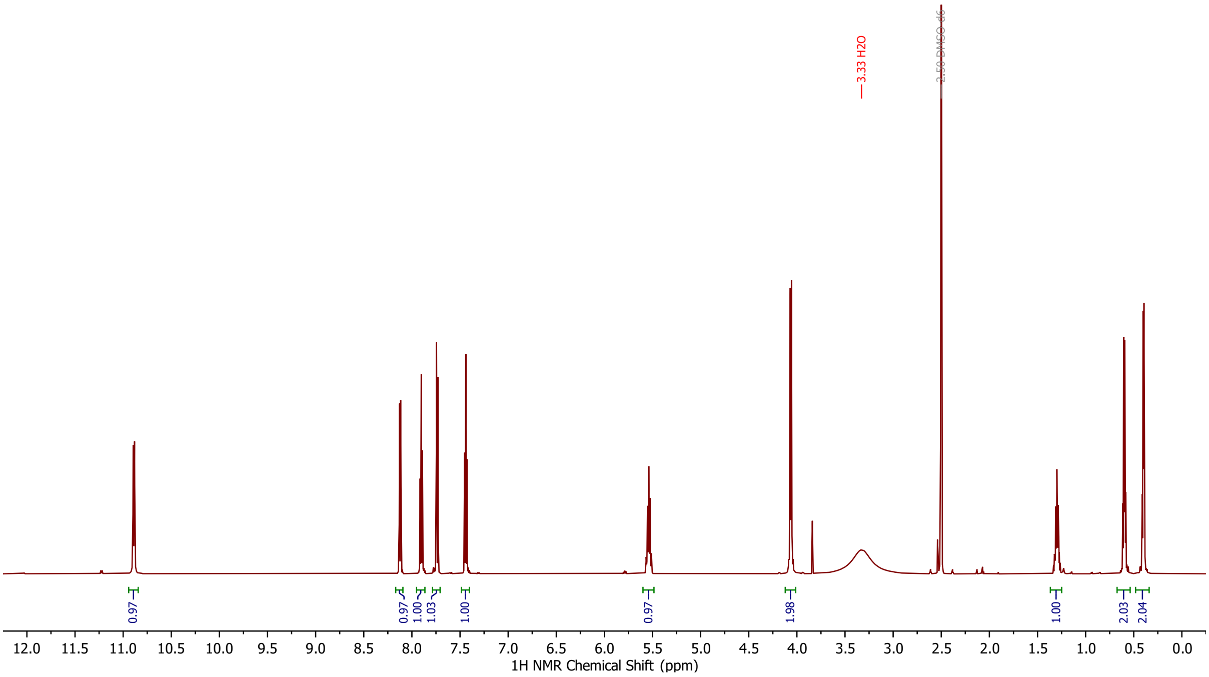

^{13}C NMR (151 MHz, 300 K, DMSO-d_6):

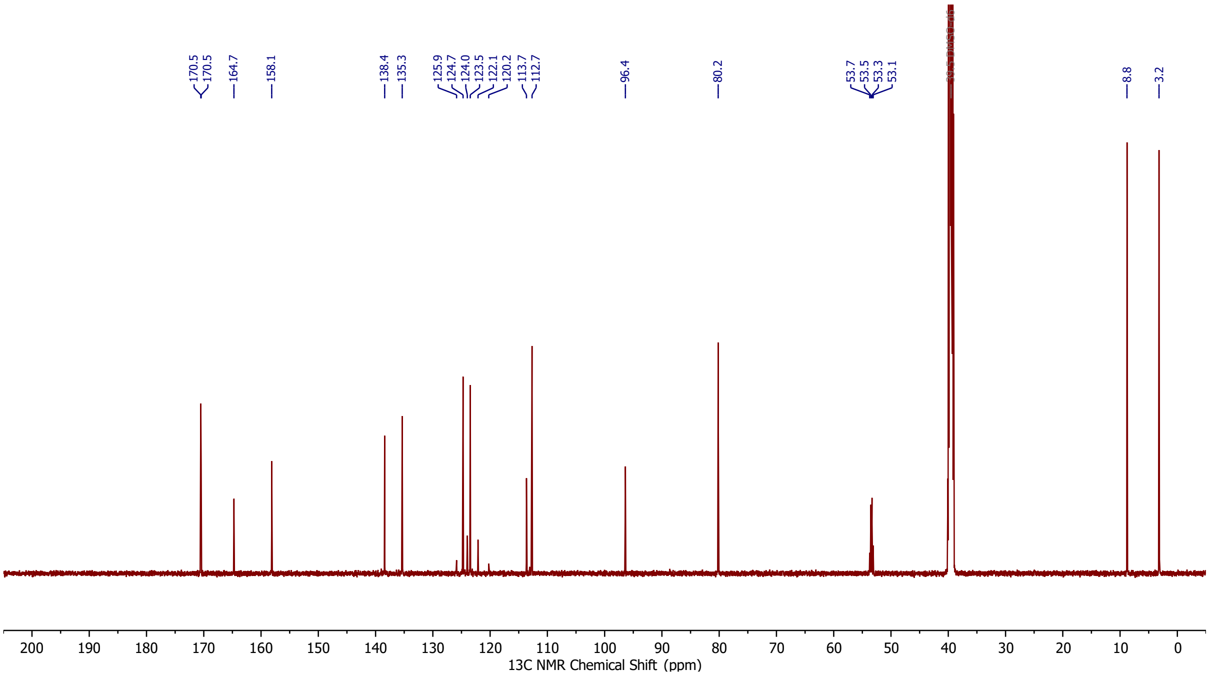

(±)-Methyl 2-cyclopropyl-2-(1-(cyclopropylmethoxy)-4-hydroxy-2-oxo-1,2-dihydroquinoline-3-carboxamido)acetate (12a)

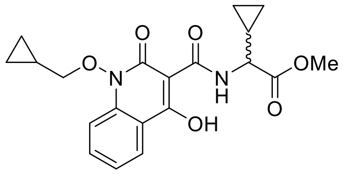

^{1}H NMR (500 MHz, 300 K, $CDCl_3$):

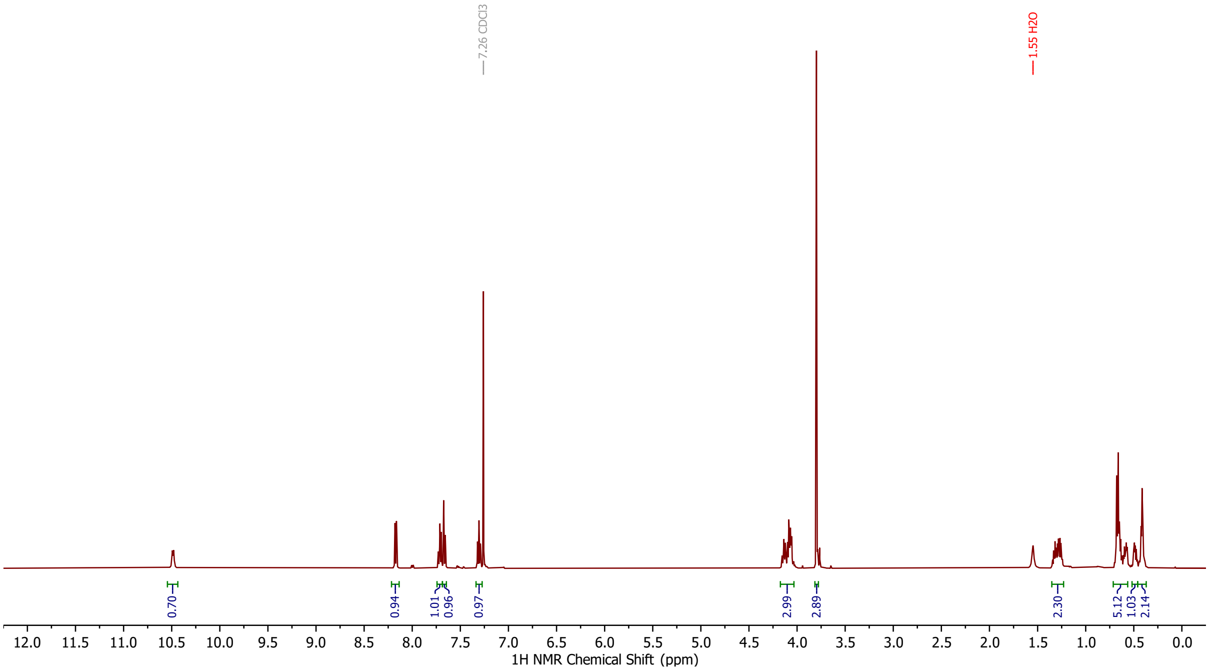

^{13}C NMR (126 MHz, 300 K, $CDCl_3$):

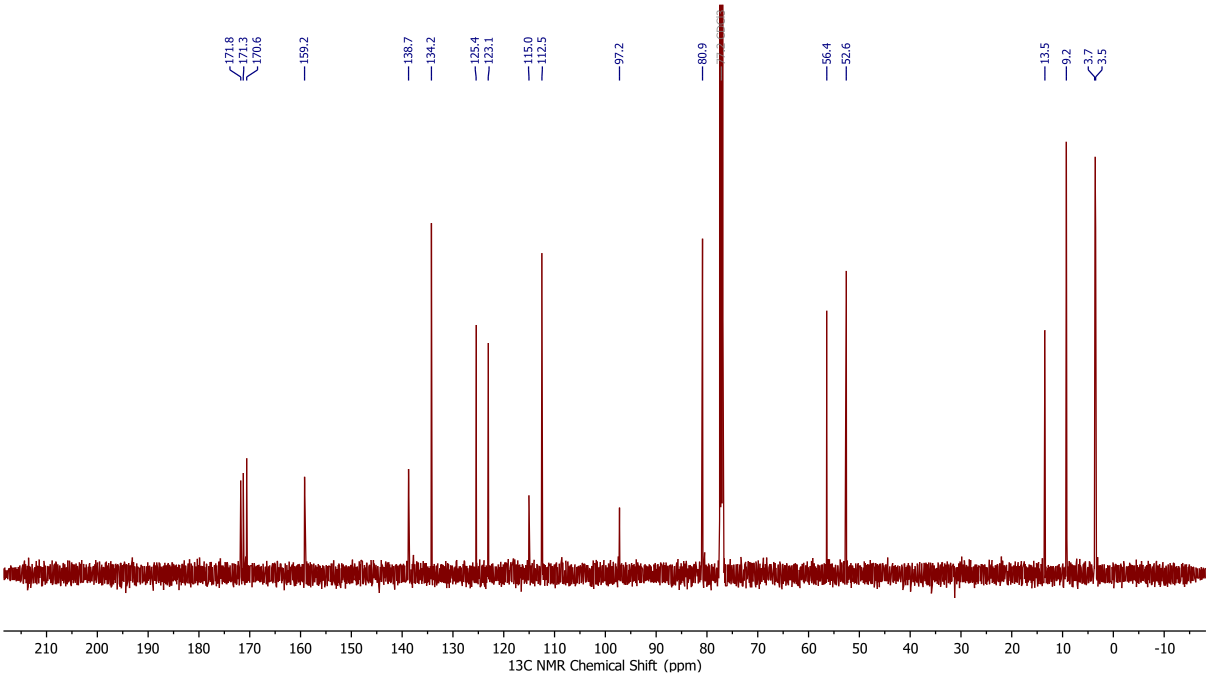

(±)-2-Cyclopropyl-2-(1-(cyclopropylmethoxy)-4-hydroxy-2-oxo-1,2-dihydroquinoline-3-carboxamido)acetic acid (12)

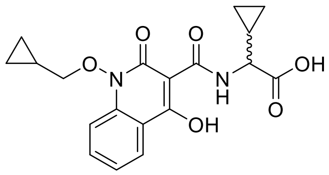

^{1}H NMR (600 MHz, 300 K, DMSO-d_6):

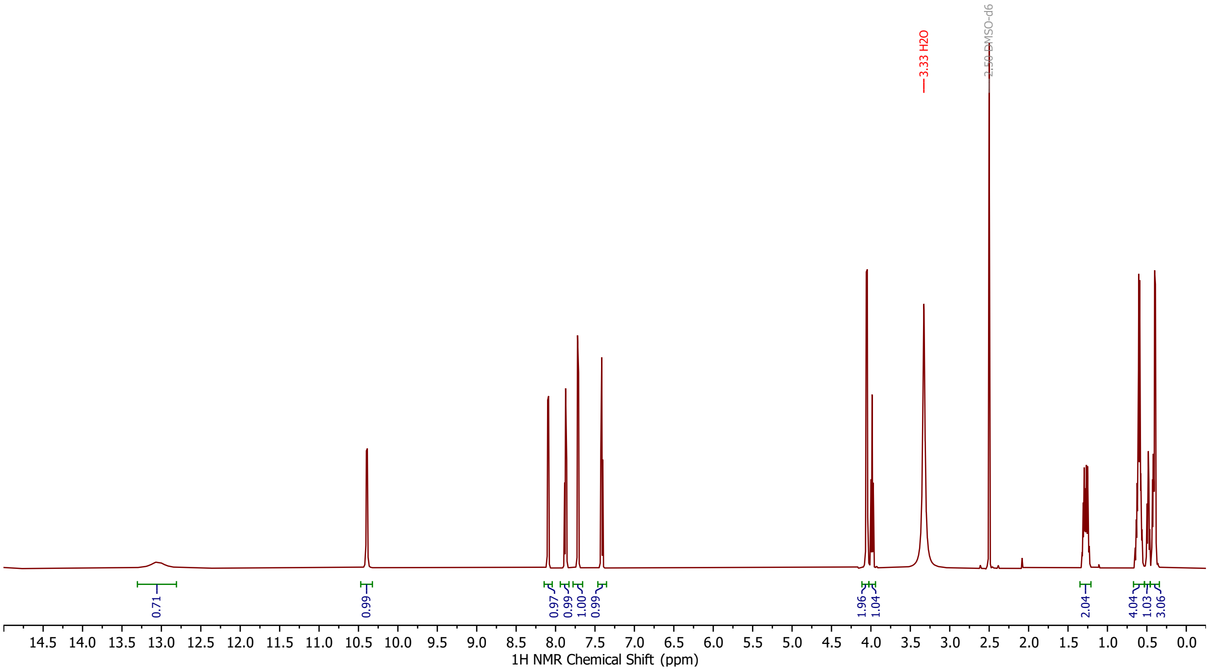

^{13}C NMR (151 MHz, 300 K, DMSO-d_6):

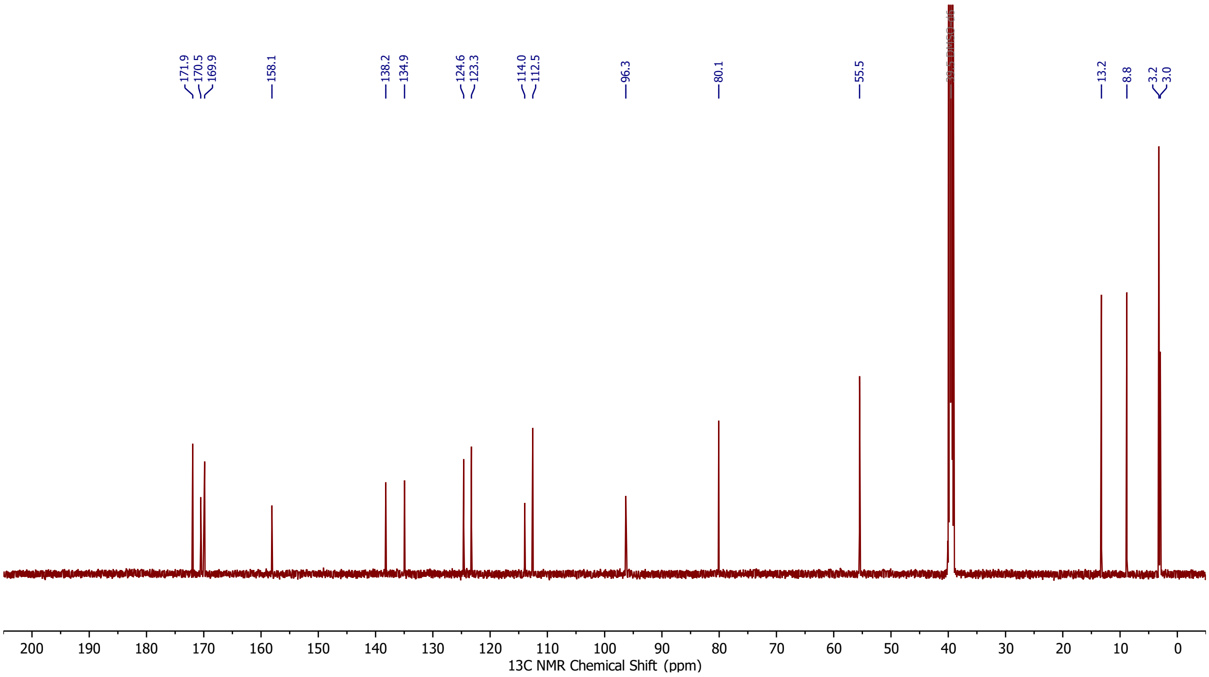

Ethyl 2-(1-(cyclopropylmethoxy)-4-hydroxy-2-oxo-1,2-dihydroquinoline-3-carboxamido)-2-methylpropanoate (13a)

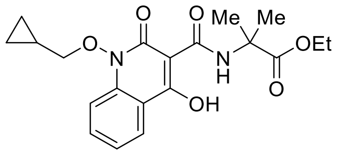

^{1}H NMR (600 MHz, 300 K, $CDCl_3$):

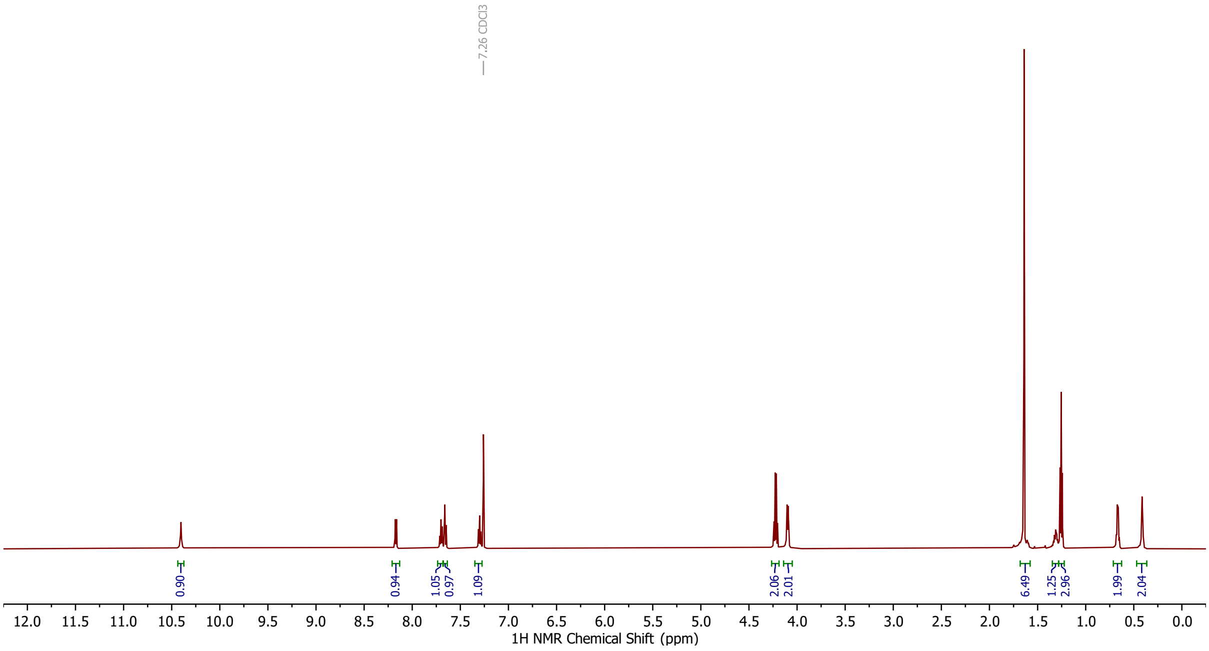

^{13}C NMR (151 MHz, 300 K, $CDCl_3$):

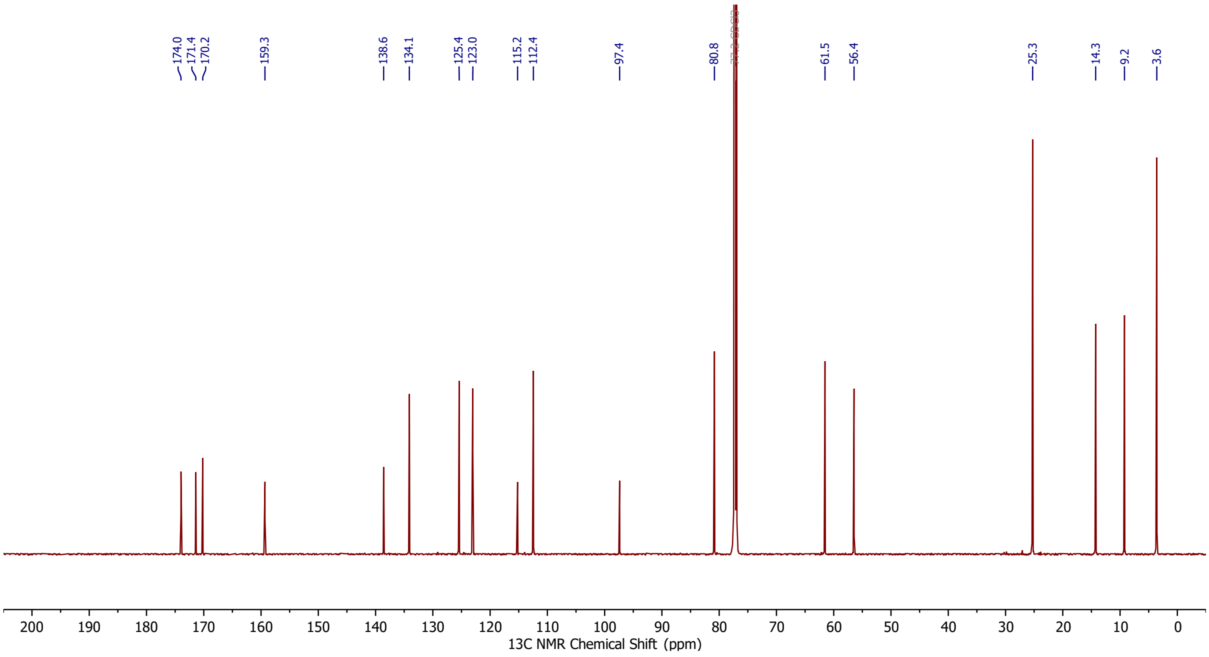

2-(1-(Cyclopropylmethoxy)-4-hydroxy-2-oxo-1,2-dihydroquinoline-3-carboxamido)-2-methylpropanoic acid (13)

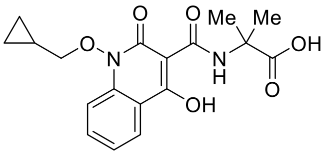

^{1}H NMR (600 MHz, 300 K, DMSO-d_6):

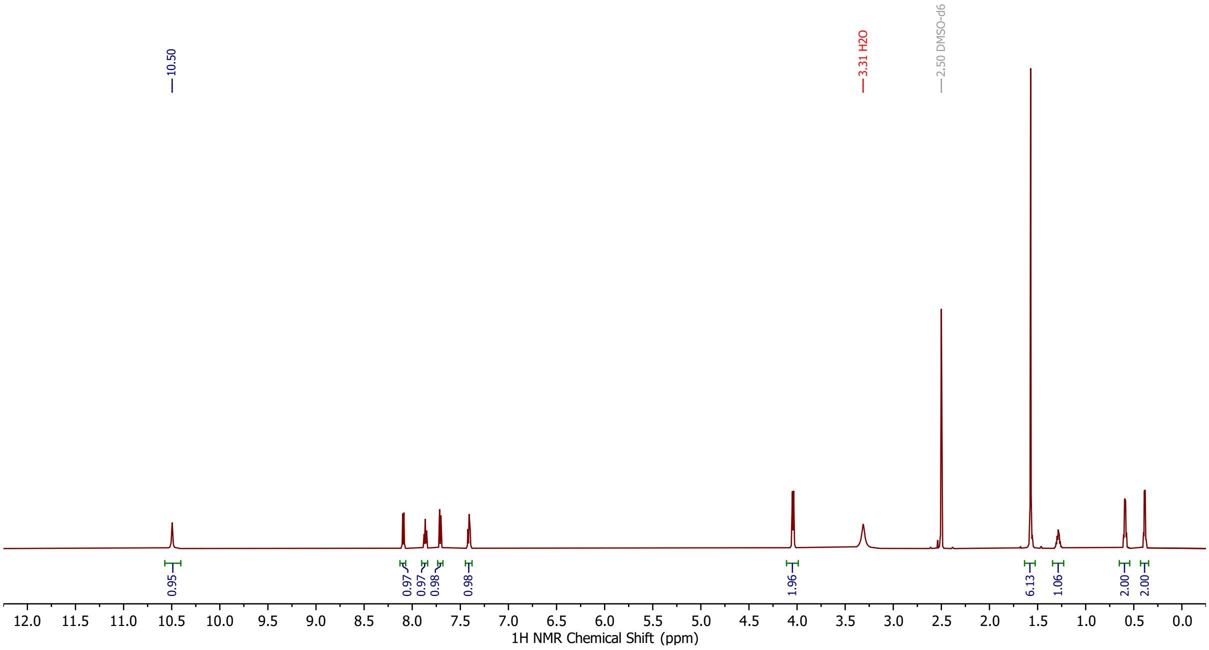

^{13}C NMR (151 MHz, 300 K, DMSO-d_6):

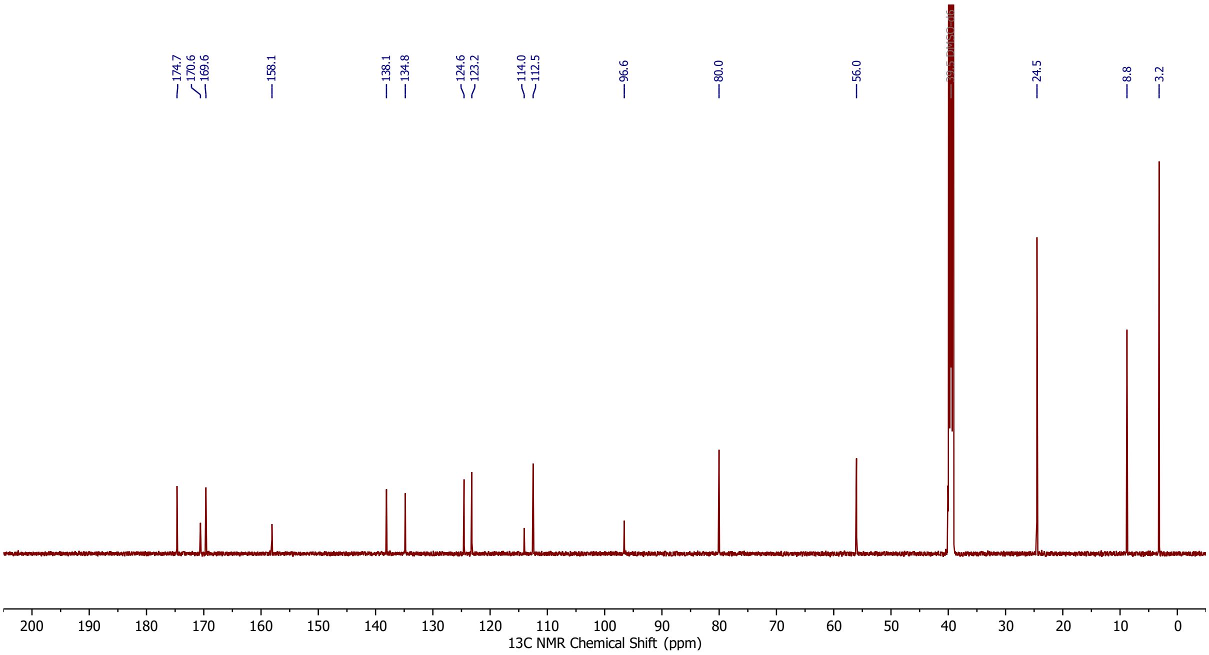

Methyl 1-(1-(cyclopropylmethoxy)-4-hydroxy-2-oxo-1,2-dihydroquinoline-3-carboxamido)-cyclopropane-1-carboxylate (14a)

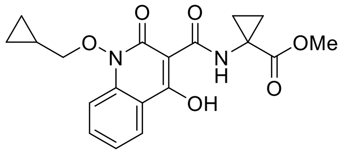

^{1}H NMR (500 MHz, 300 K, $CDCl_3$):

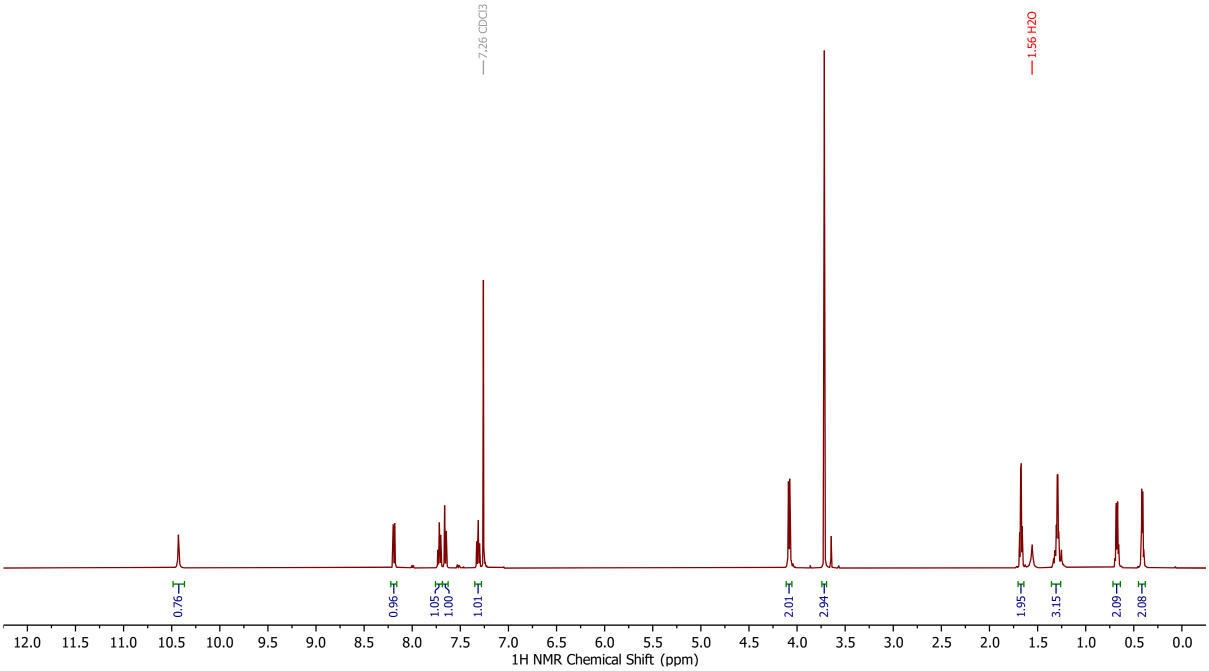

^{13}C NMR (126 MHz, 300 K, $CDCl_3$):

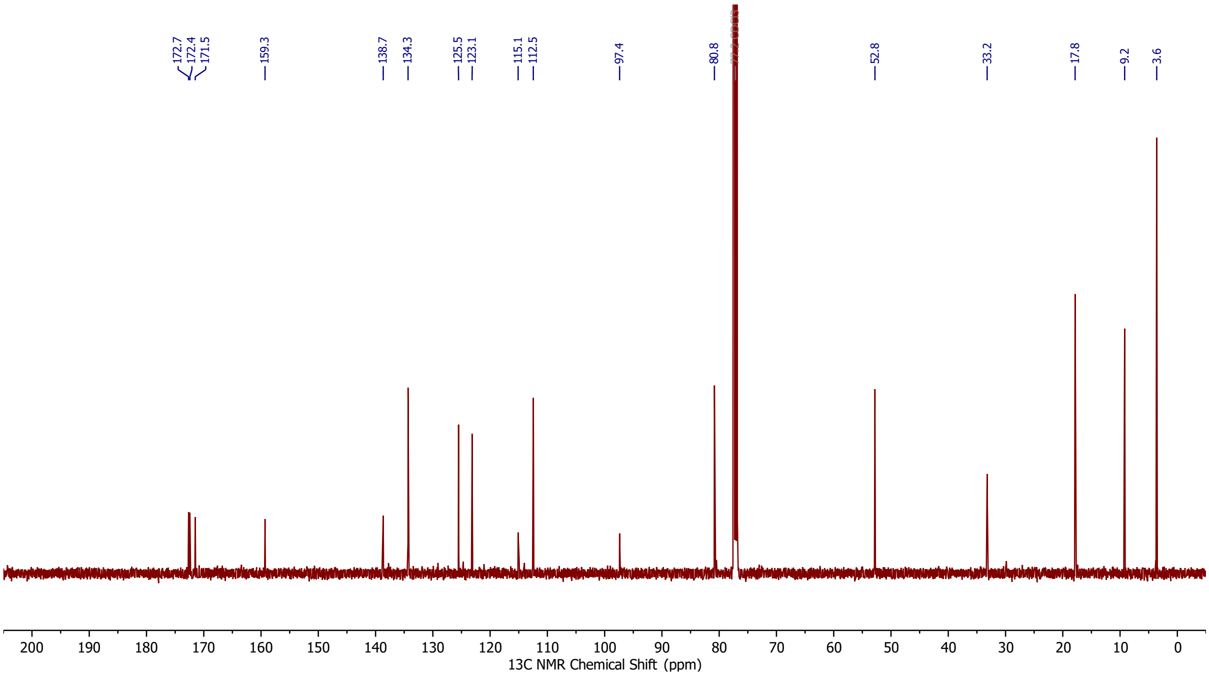

1-(1-(Cyclopropylmethoxy)-4-hydroxy-2-oxo-1,2-dihydroquinoline-3-carboxamido)cyclopropane-1-carboxylic acid (14)

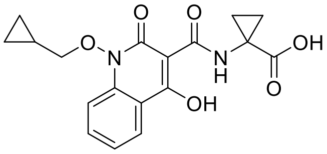

^{1}H NMR (500 MHz, 300 K, DMSO-d_6):

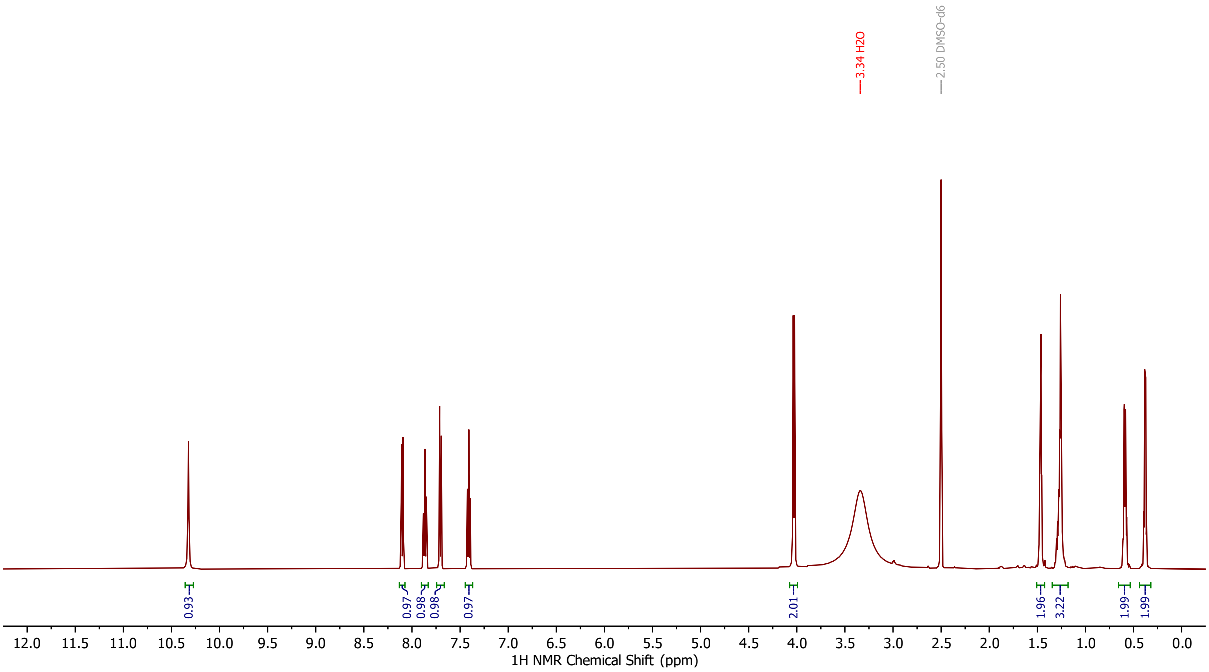

^{13}C NMR (126 MHz, 300 K, DMSO-d_6):

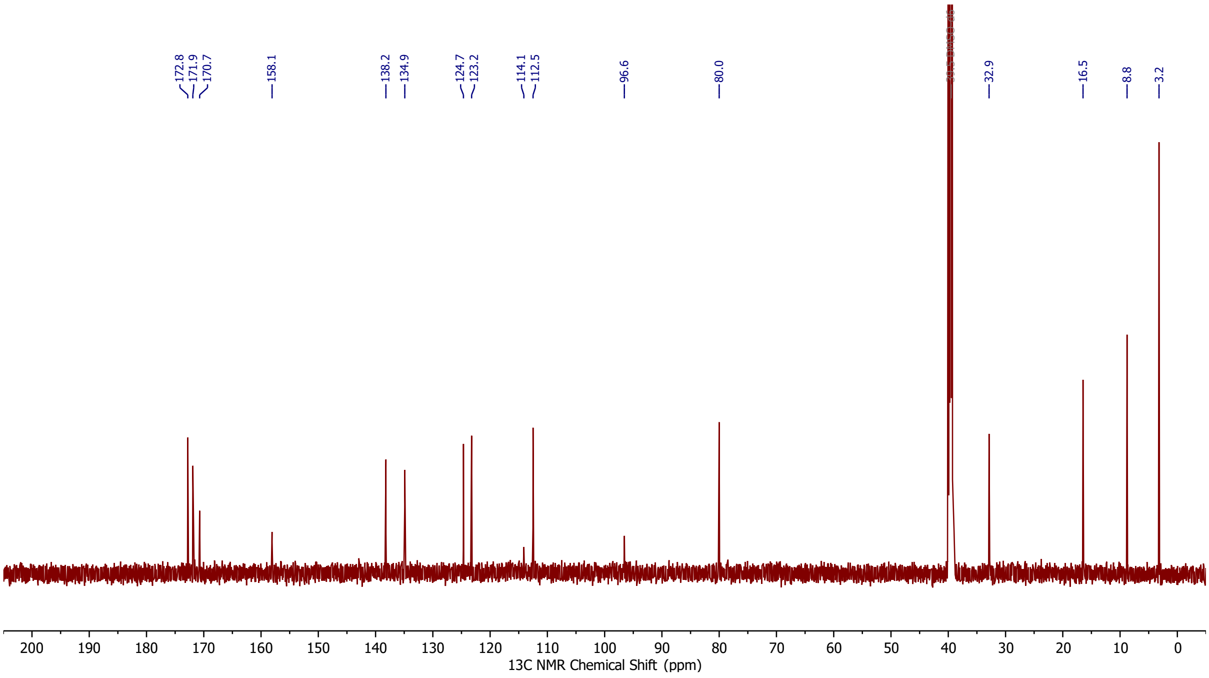

Methyl 1-(1-(cyclopropylmethoxy)-4-hydroxy-2-oxo-1,2-dihydroquinoline-3-carboxamido)-cyclobutane-1-carboxylate (15a)

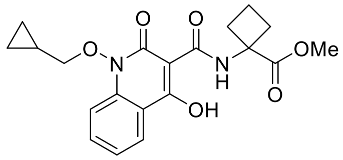

^{1}H NMR (500 MHz, 300 K, $CDCl_3$):

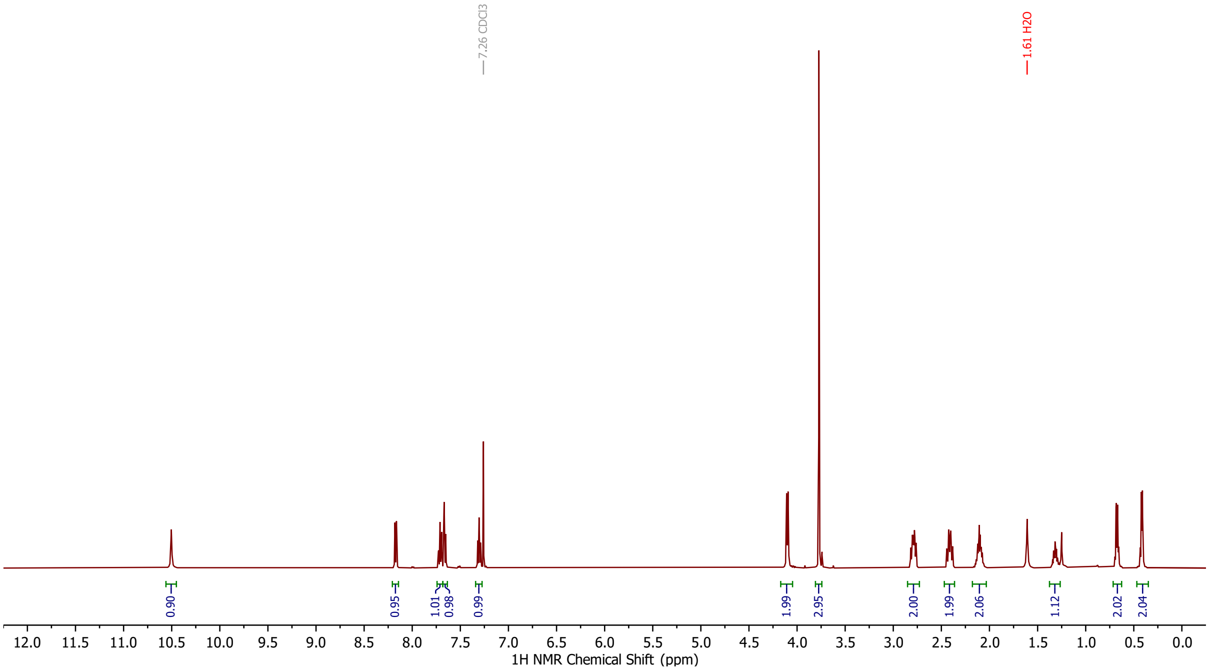

^{13}C NMR (126 MHz, 300 K, $CDCl_3$):

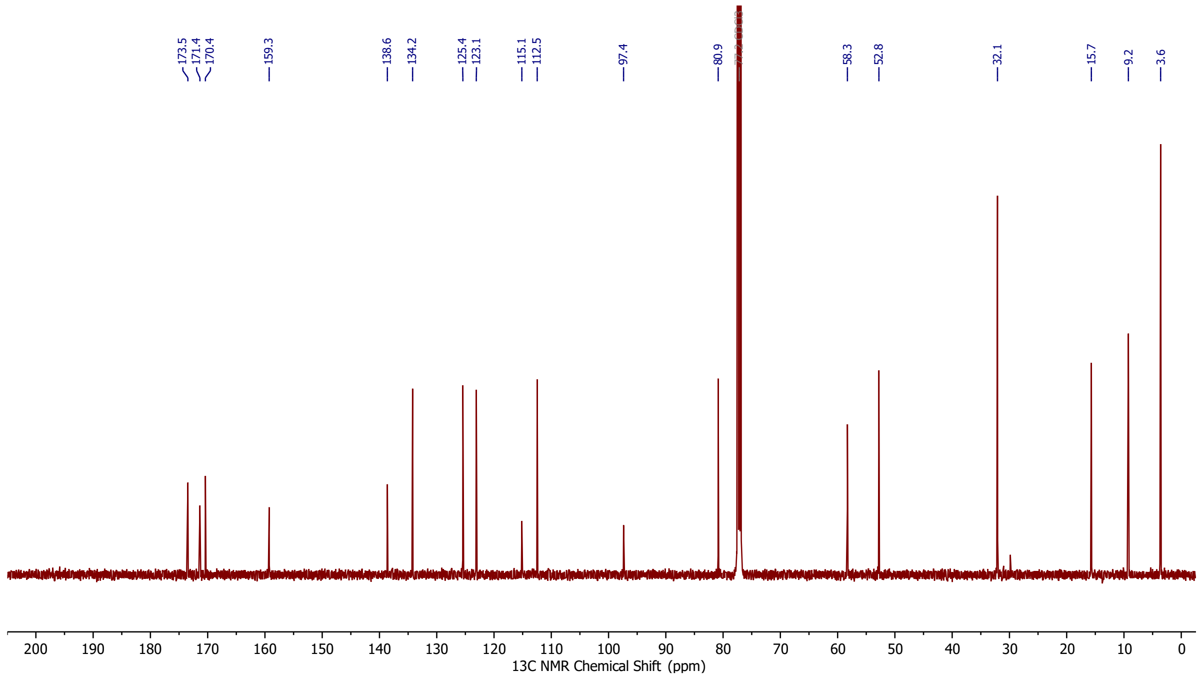

1-(1-(Cyclopropylmethoxy)-4-hydroxy-2-oxo-1,2-dihydroquinoline-3-carboxamido)cyclobutane-1-carboxylic acid (15)

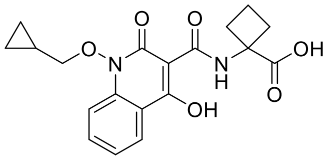

^{1}H NMR (500 MHz, 300 K, DMSO-d_6):

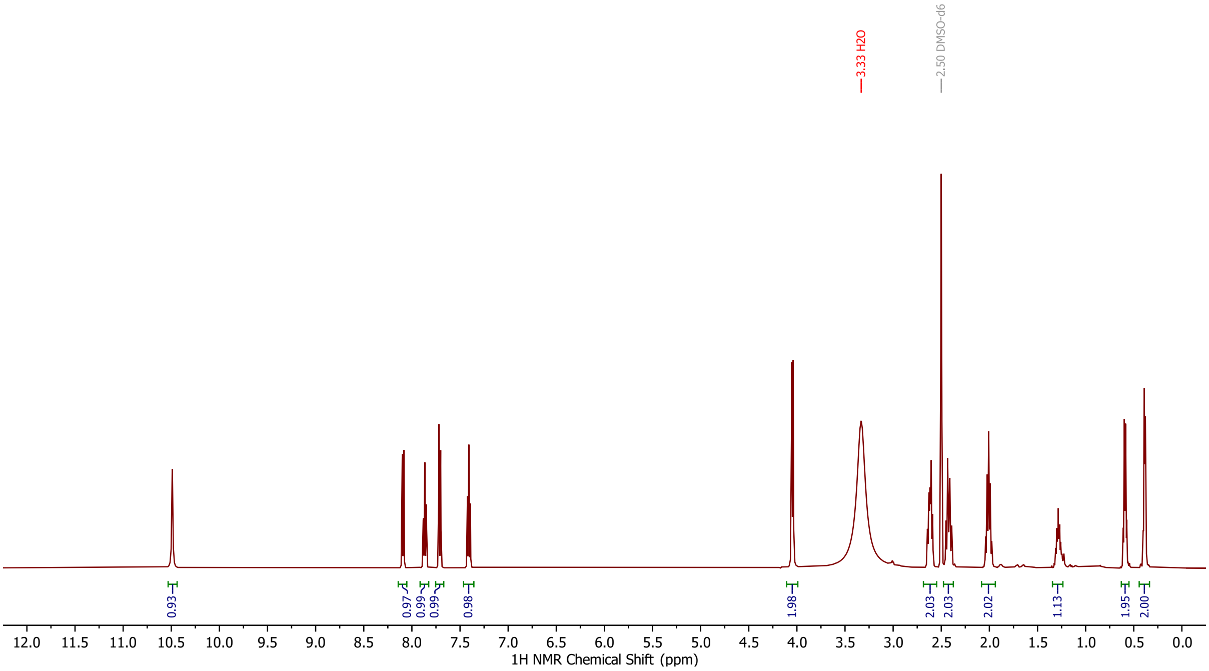

^{13}C NMR (126 MHz, 300 K, DMSO-d_6):

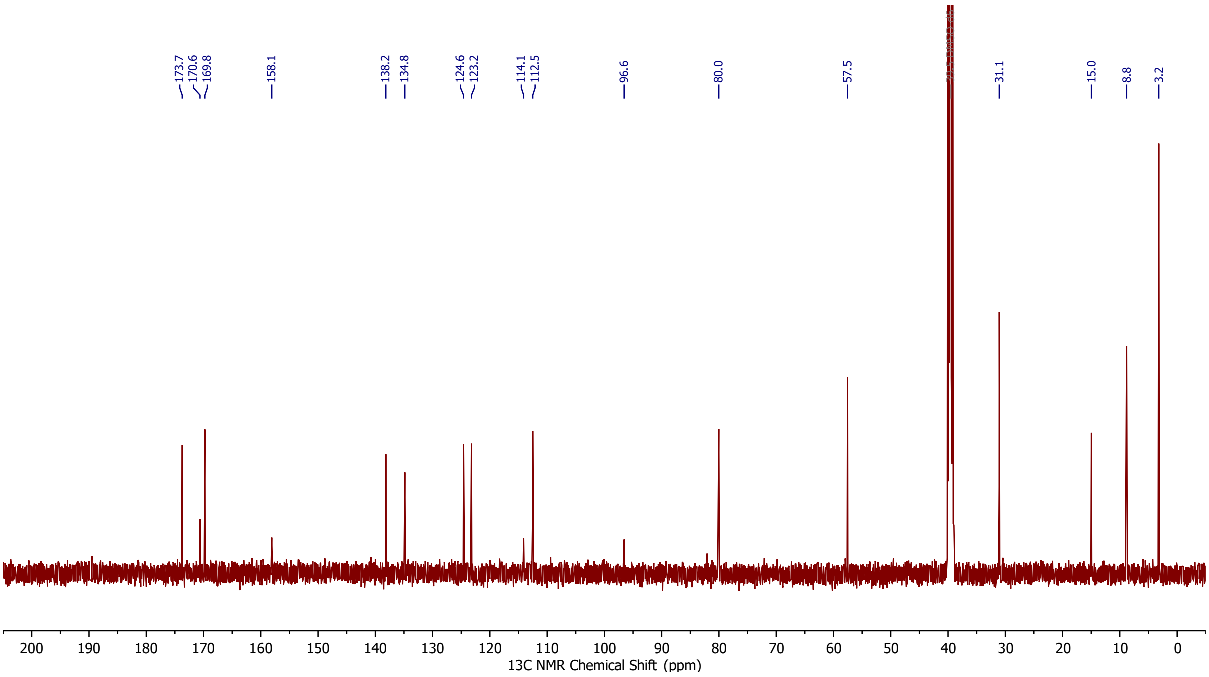

(±)-Methyl 2-(1-(cyclopropylmethoxy)-4-hydroxy-2-oxo-1,2-dihydroquinoline-3-carboxamido)-2-phenylacetate (16a)

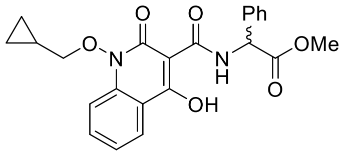

^{1}H NMR (500 MHz, 300 K, $CDCl_3$):

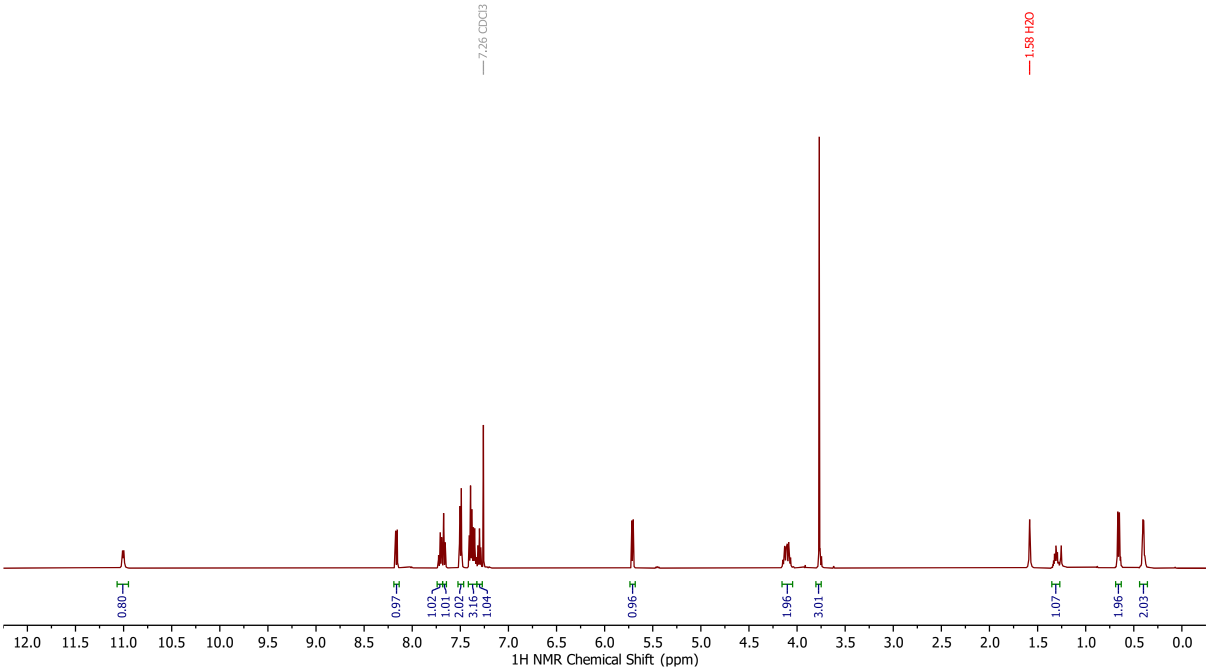

^{13}C NMR (126 MHz, 300 K, $CDCl_3$):

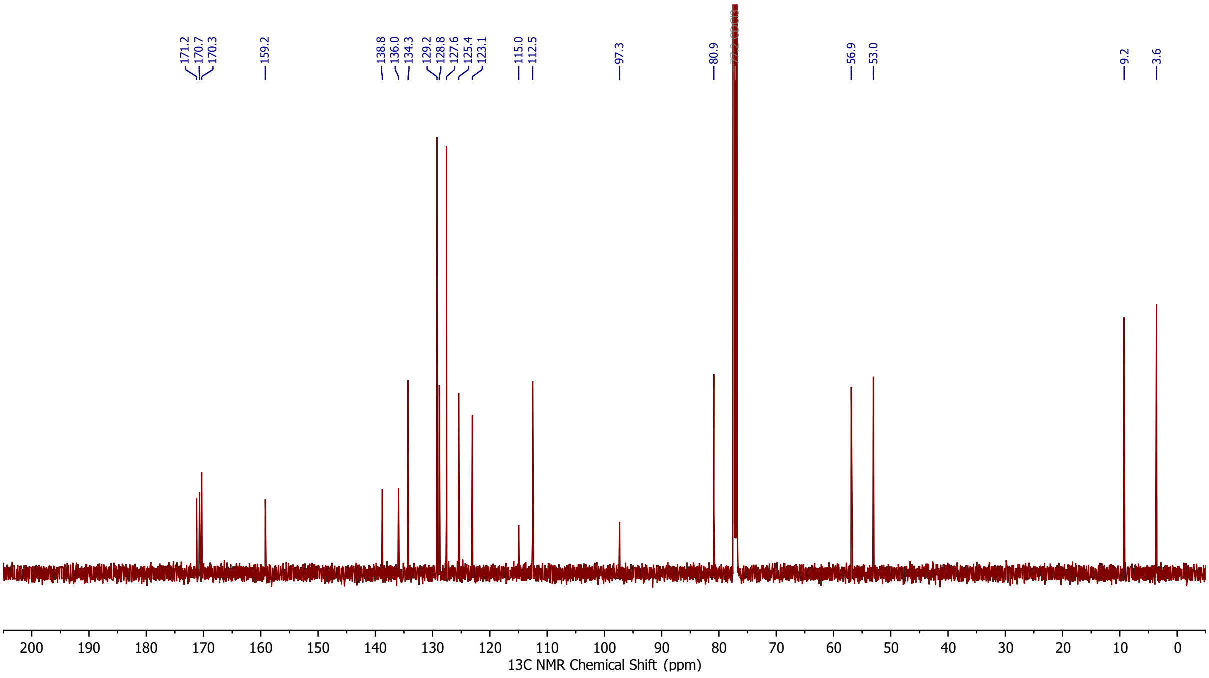

(±)-2-(1-(Cyclopropylmethoxy)-4-hydroxy-2-oxo-1,2-dihydroquinoline-3-carboxamido)-2-phenylacetic acid (16)

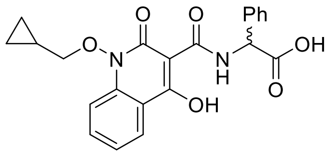

^{1}H NMR (600 MHz, 300 K, DMSO-d_6):

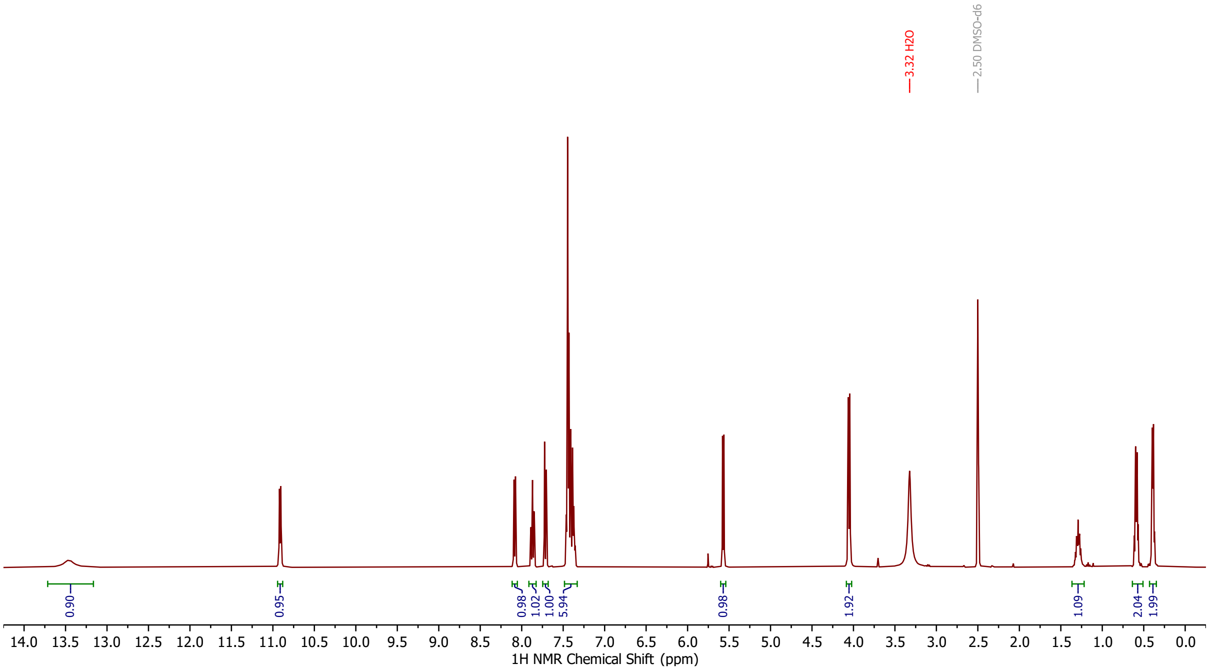

^{13}C NMR (151 MHz, 300 K, DMSO-d_6):

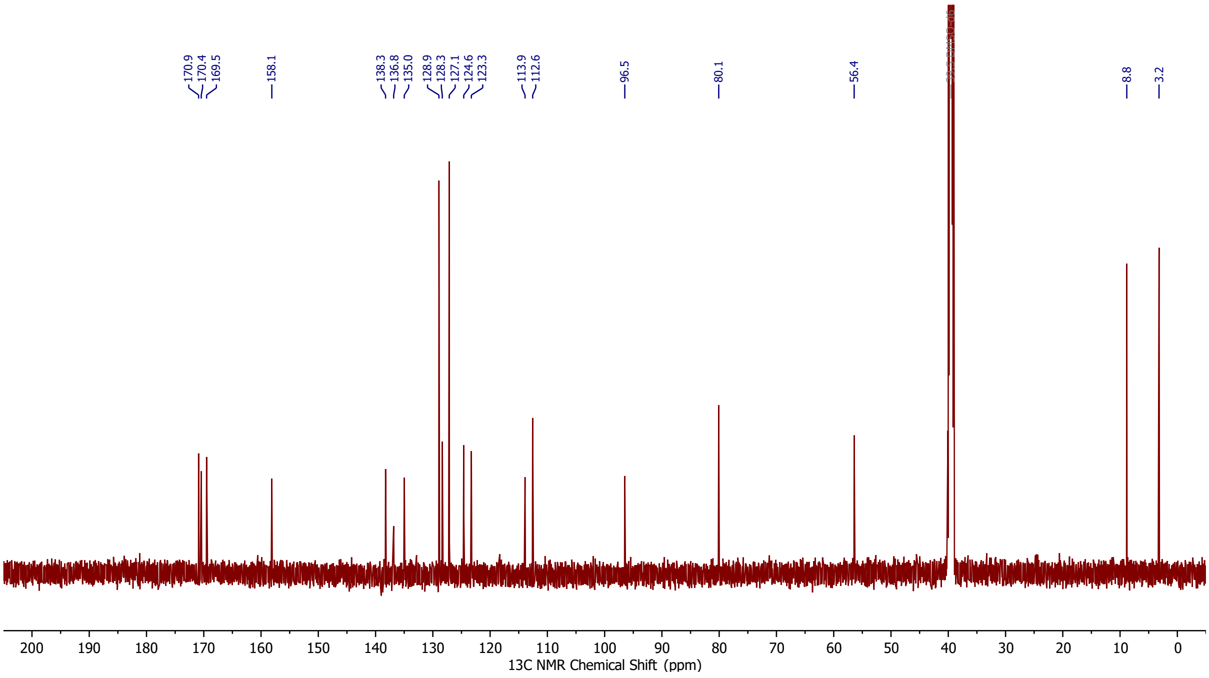

(±)-Methyl (1-(cyclopropylmethoxy)-4-hydroxy-2-oxo-1,2-dihydroquinoline-3-carbonyl)-phenylalaninate (17a)

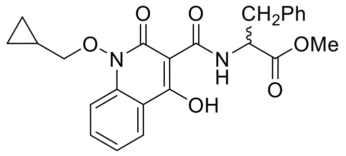

^{1}H NMR (600 MHz, 300 K, $CDCl_3$):

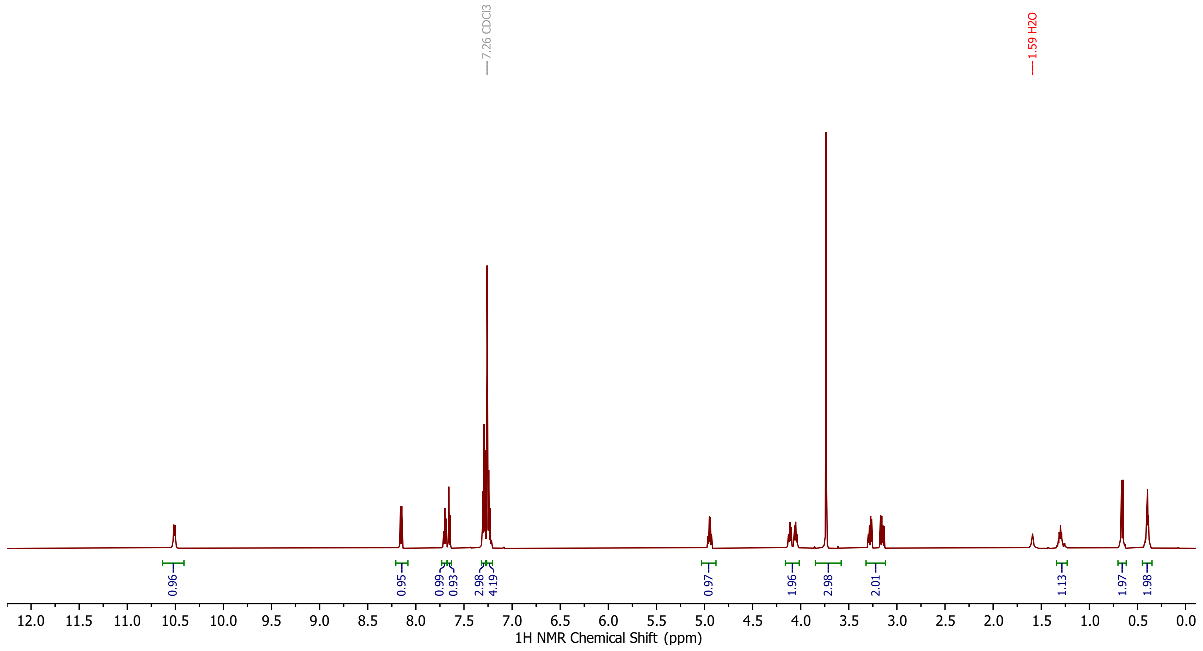

^{13}C NMR (151 MHz, 300 K, $CDCl_3$):

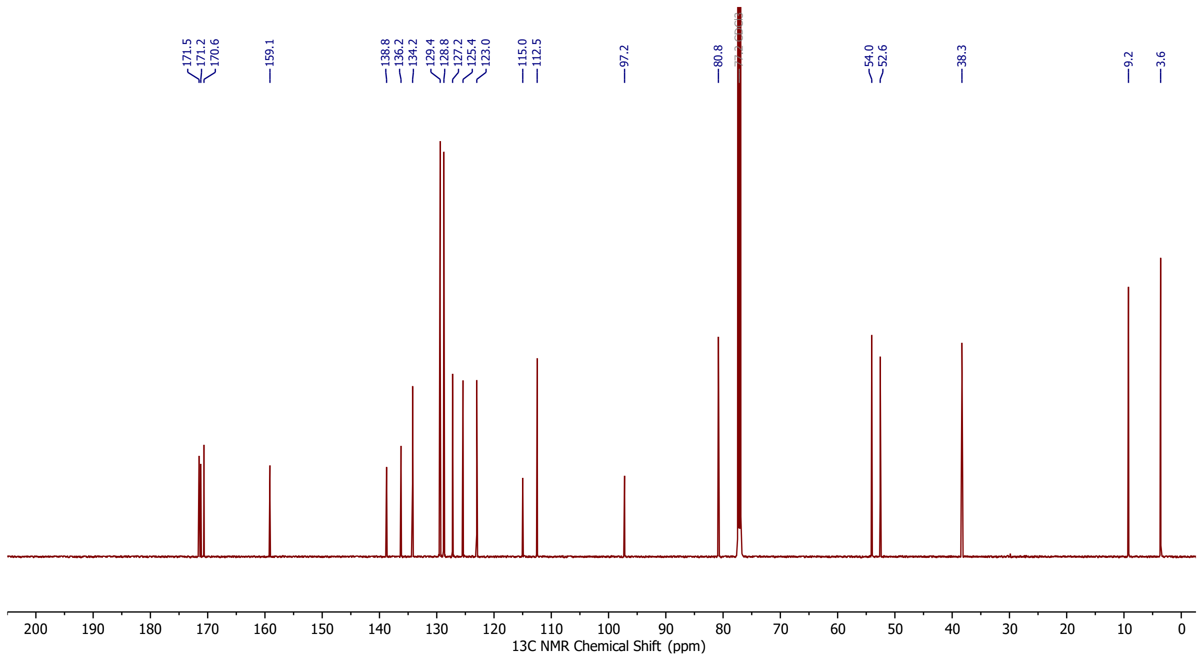

(±)-(1-(Cyclopropylmethoxy)-4-hydroxy-2-oxo-1,2-dihydroquinoline-3-carbonyl)phenylalanine (17)

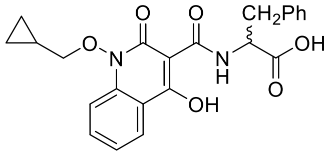

^{1}H NMR (600 MHz, 300 K, DMSO-d_6):

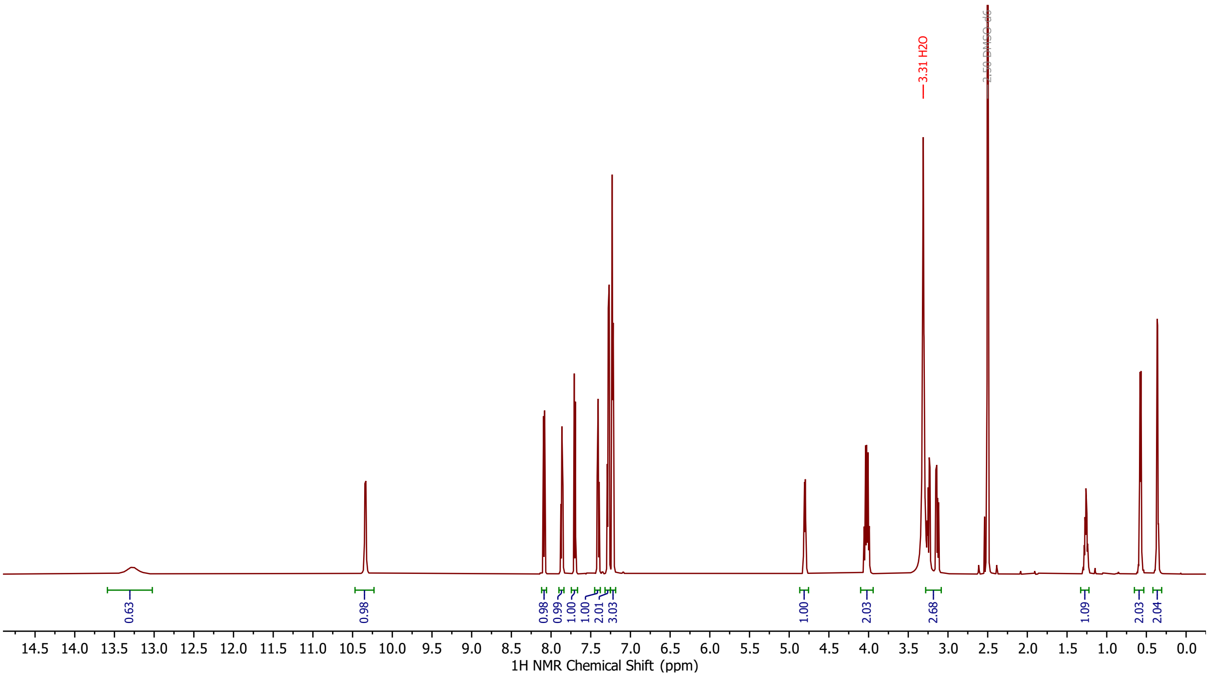

^{13}C NMR (151 MHz, 300 K, DMSO-d_6):

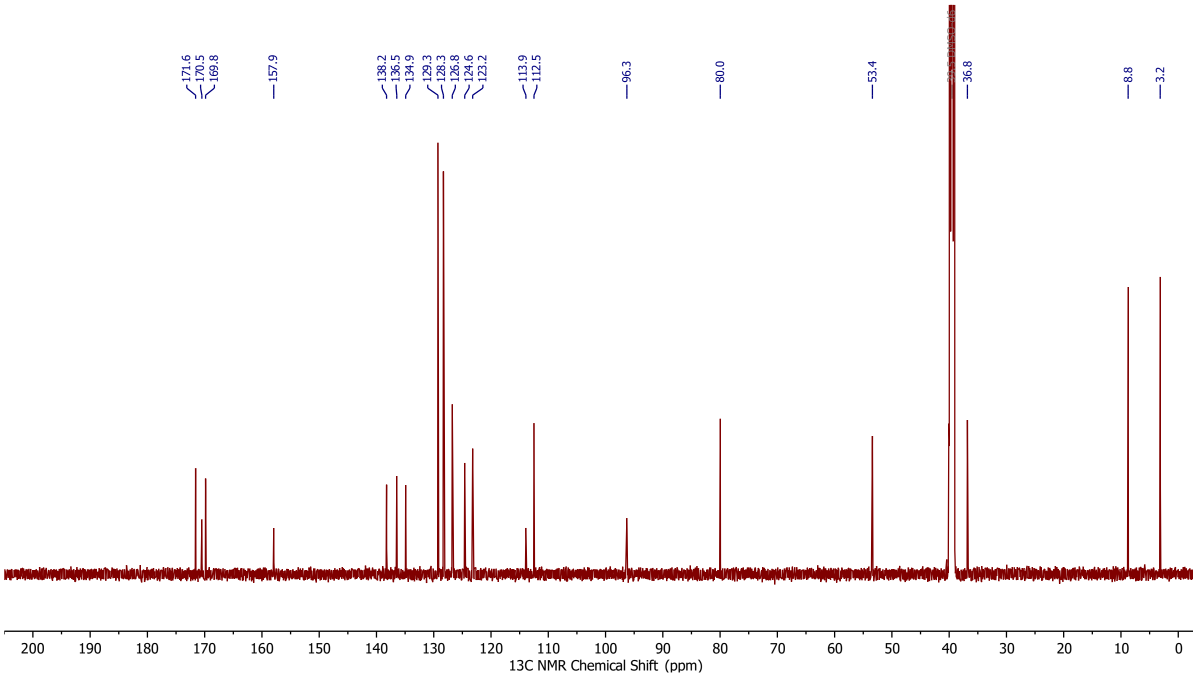

(±)-Methyl 2-(1-(cyclopropylmethoxy)-4-hydroxy-2-oxo-1,2-dihydroquinoline-3-carboxamido)-4-phenylbutanoate (18a)

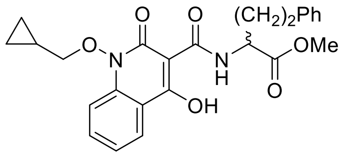

^1H NMR (600 MHz, 300 K, $CDCl_3$):

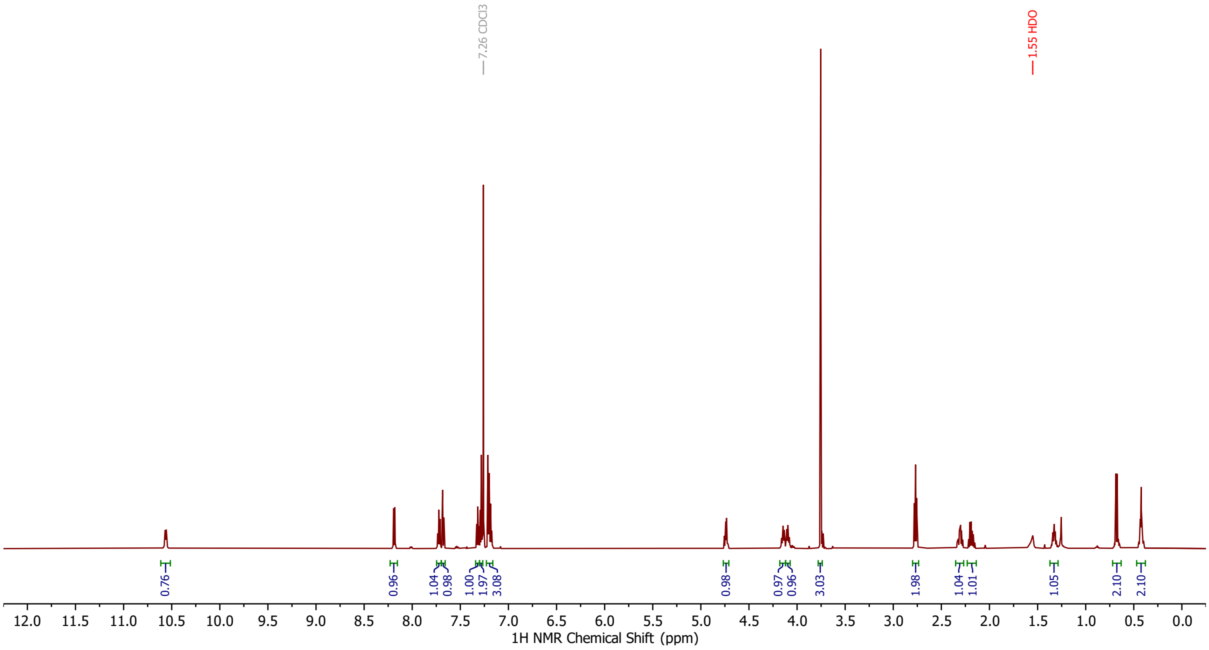

^{13}C NMR (151 MHz, 300 K, $CDCl_3$):

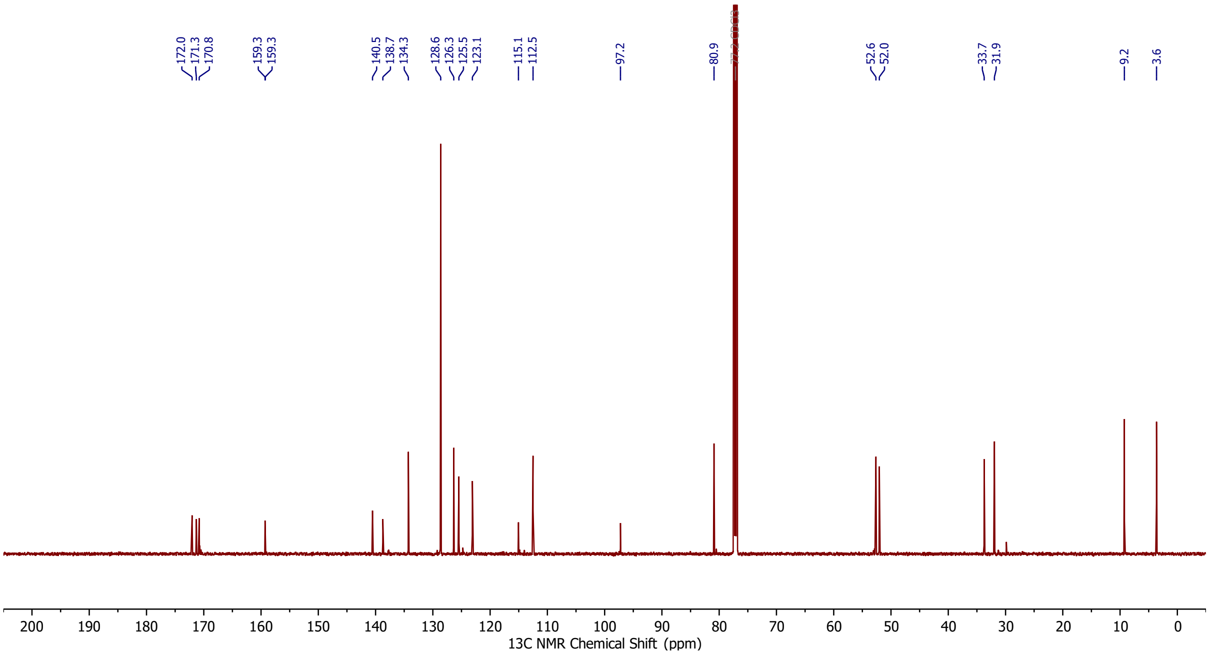

(±)-2-(1-(Cyclopropylmethoxy)-4-hydroxy-2-oxo-1,2-dihydroquinoline-3-carboxamido)-4-phenyl-butanoic acid (18)

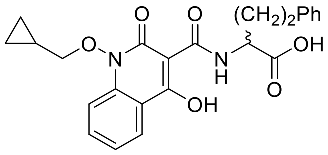

^{1}H NMR (500 MHz, 300 K, DMSO-d_6):

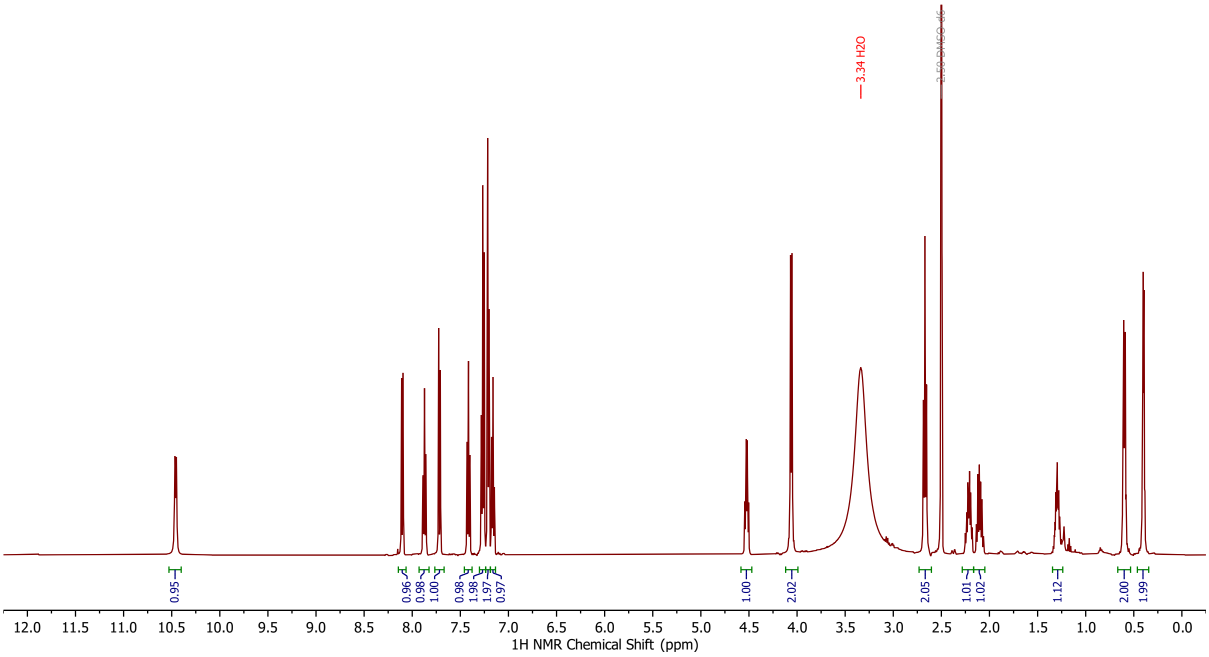

^{13}C NMR (126 MHz, 300 K, DMSO-d_6):

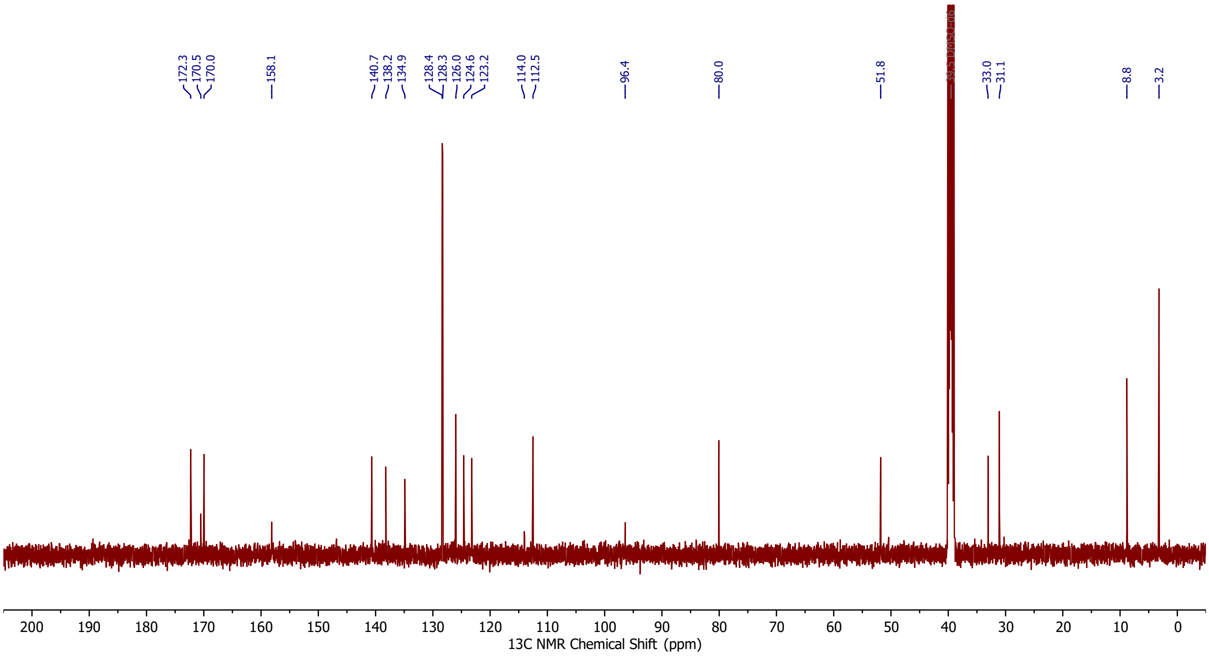

(±)-Methyl (1-(cyclopropylmethoxy)-4-hydroxy-2-oxo-1,2-dihydroquinoline-3-carbonyl)-tryptophanate (19a)

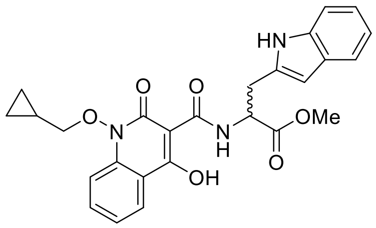

^{1}H NMR (500 MHz, 300 K, $CDCl_3$):

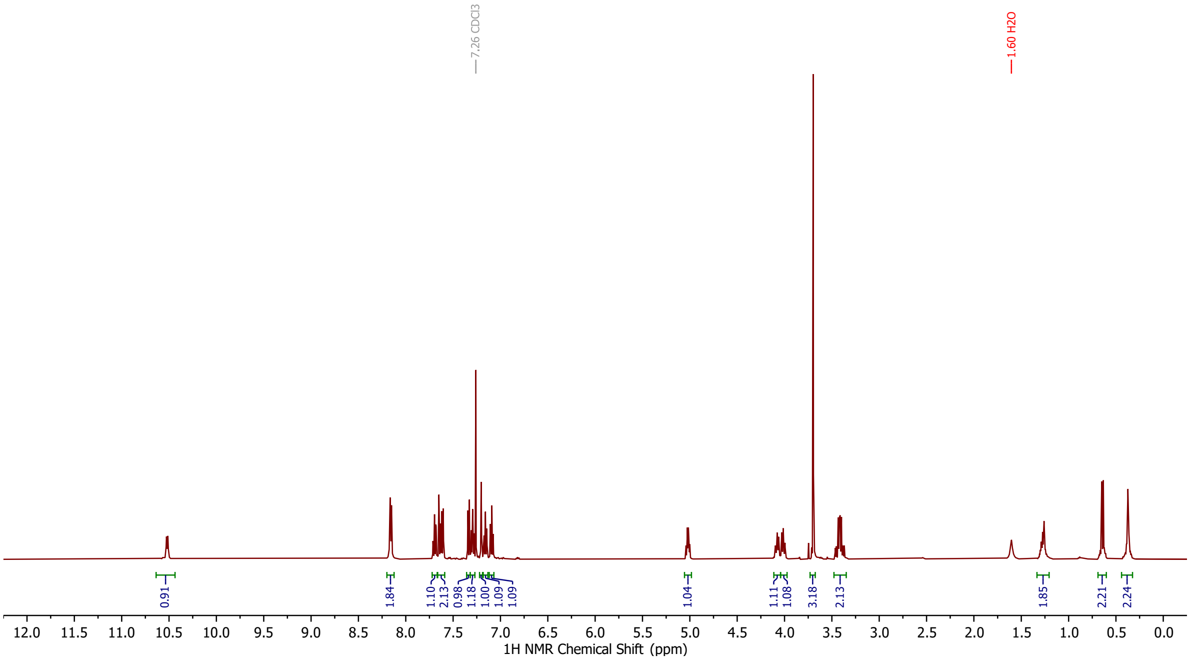

^{13}C NMR (126 MHz, 300 K, $CDCl_3$):

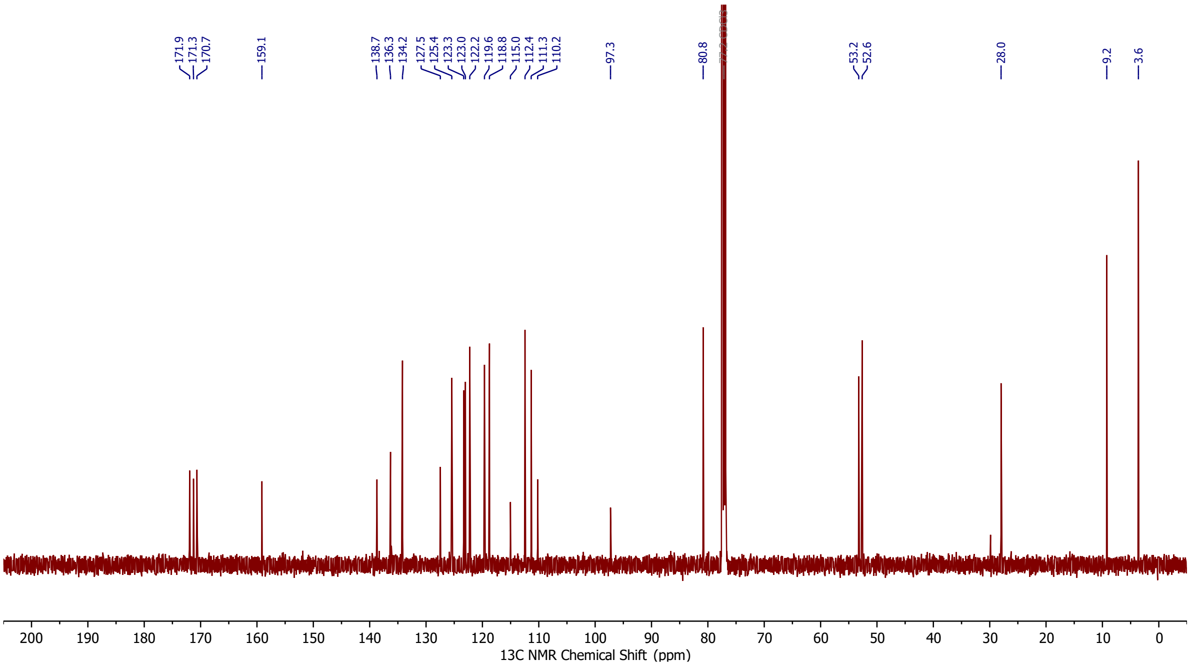

(±)-(1-(Cyclopropylmethoxy)-4-hydroxy-2-oxo-1,2-dihydroquinoline-3-carbonyl)tryptophan (19)

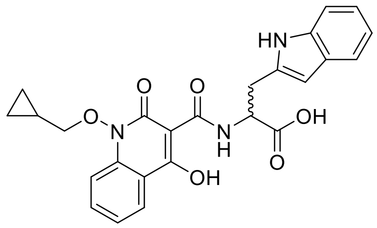

^{1}H NMR (500 MHz, 300 K, DMSO-d_6):

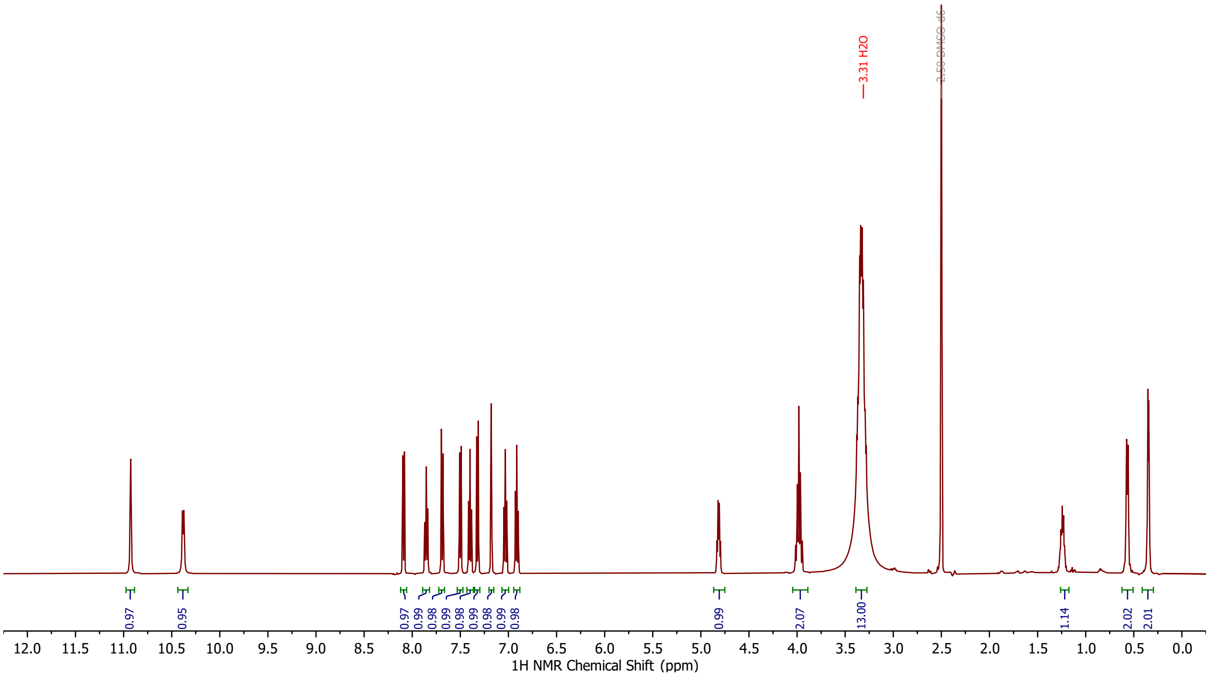

^{13}C NMR (126 MHz, 300 K, DMSO-d_6):

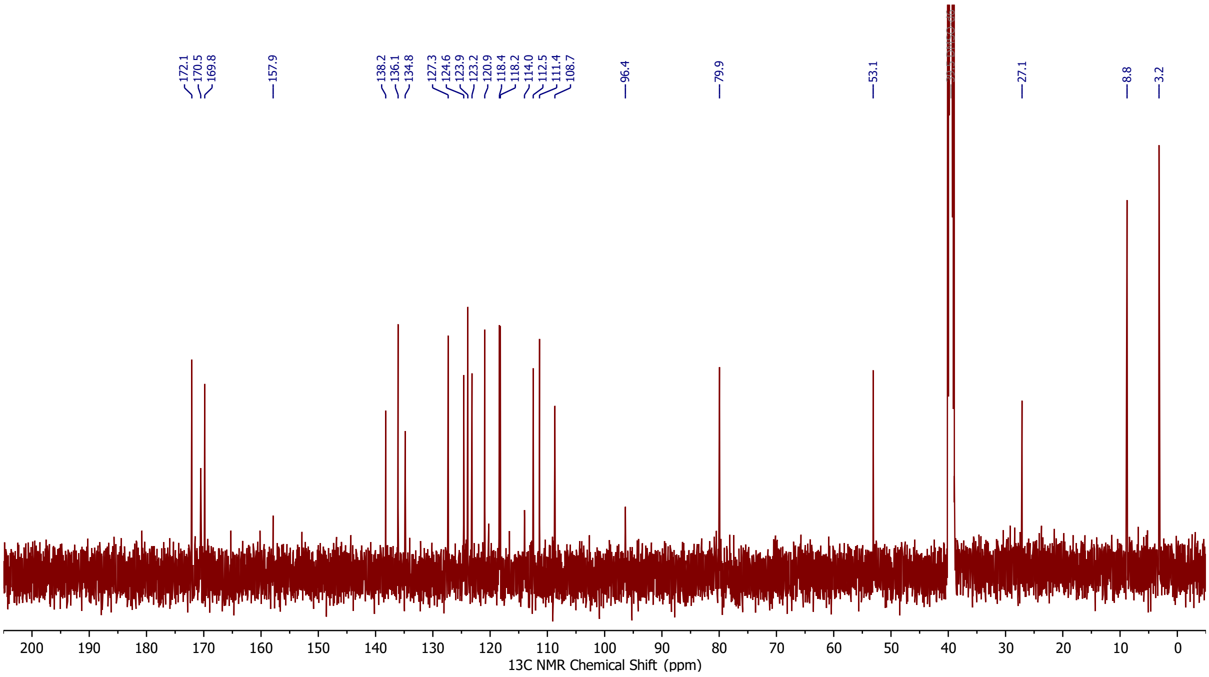

1-(Cyclopropylmethoxy)-4-hydroxy-2-oxo-1,2-dihydroquinoline-3-carboxamide (20)

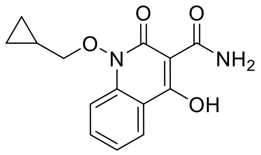

^{1}H NMR (600 MHz, 300 K, DMSO-d_6):

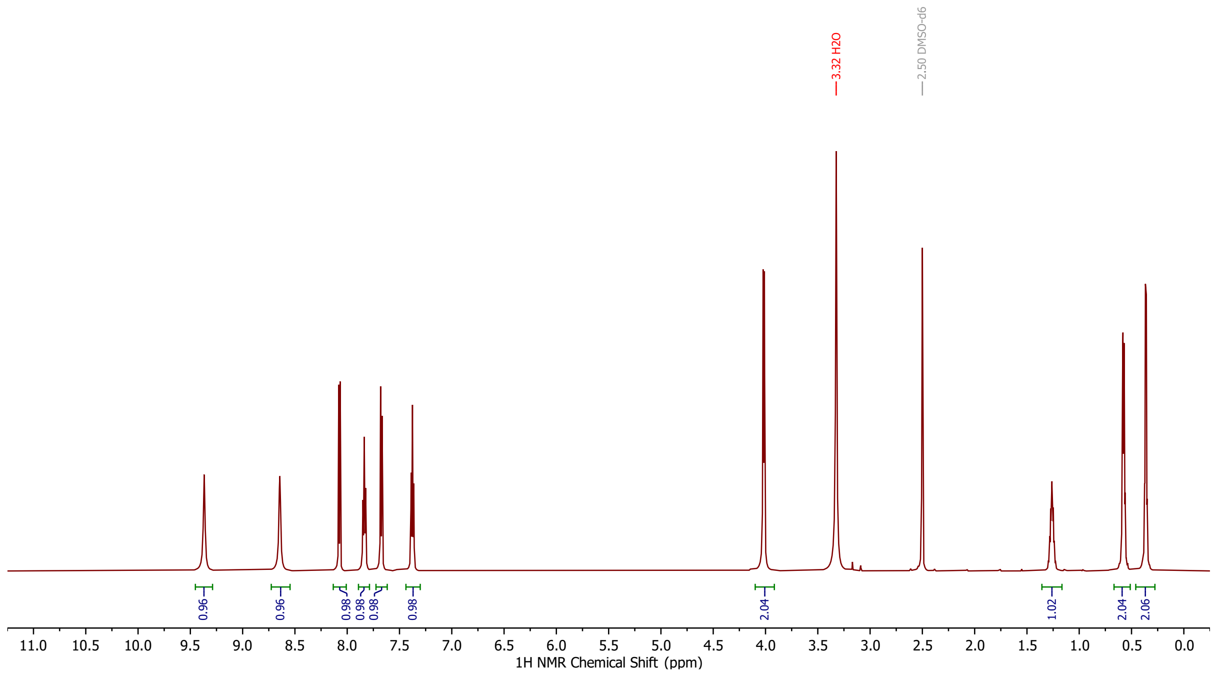

^{13}C NMR (151 MHz, 300 K, DMSO-d_6):

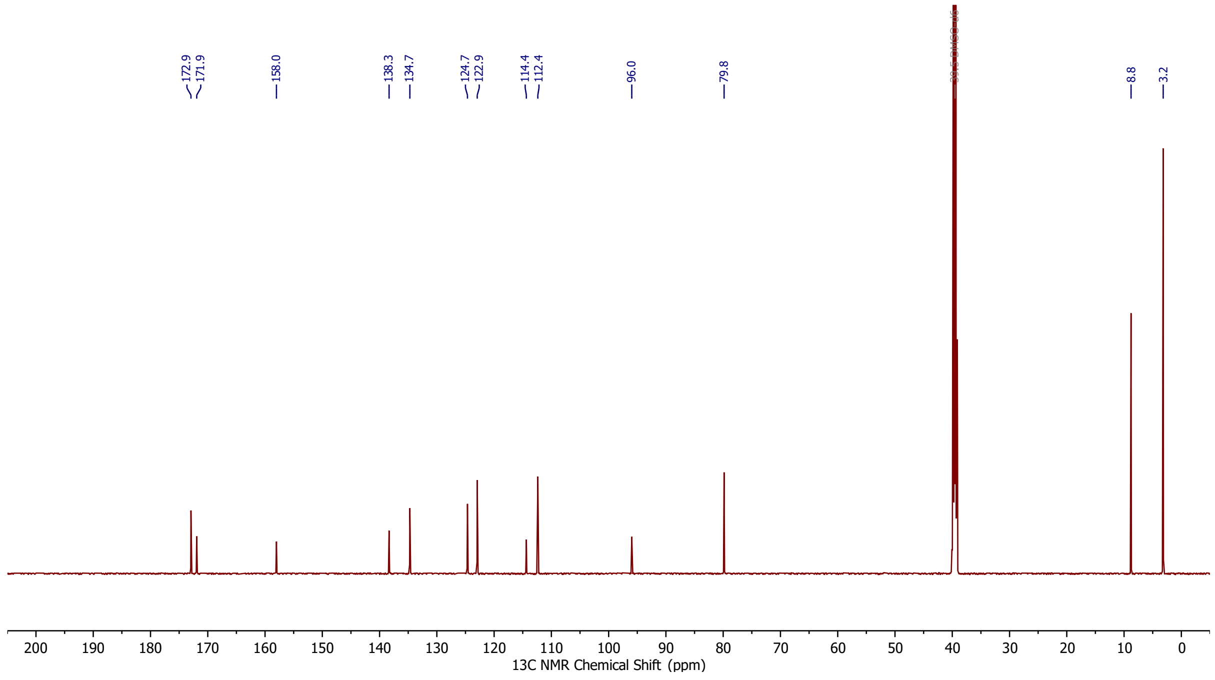

Ethyl 3-(1-(cyclopropylmethoxy)-4-hydroxy-2-oxo-1,2-dihydroquinoline-3-carboxamido)-propanoate (21a)

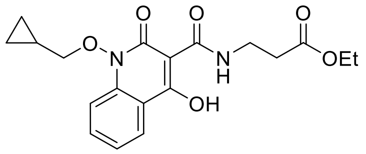

^{1}H NMR (600 MHz, 300 K, $CDCl_3$):

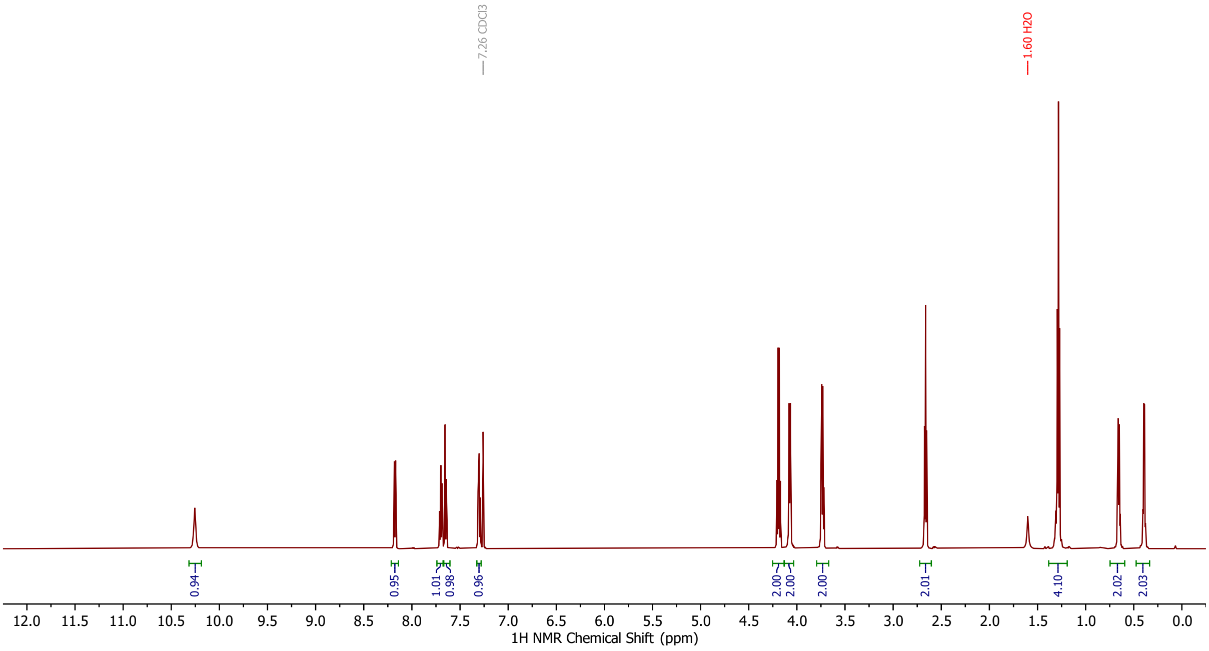

^{13}C NMR (151 MHz, 300 K, $CDCl_3$):

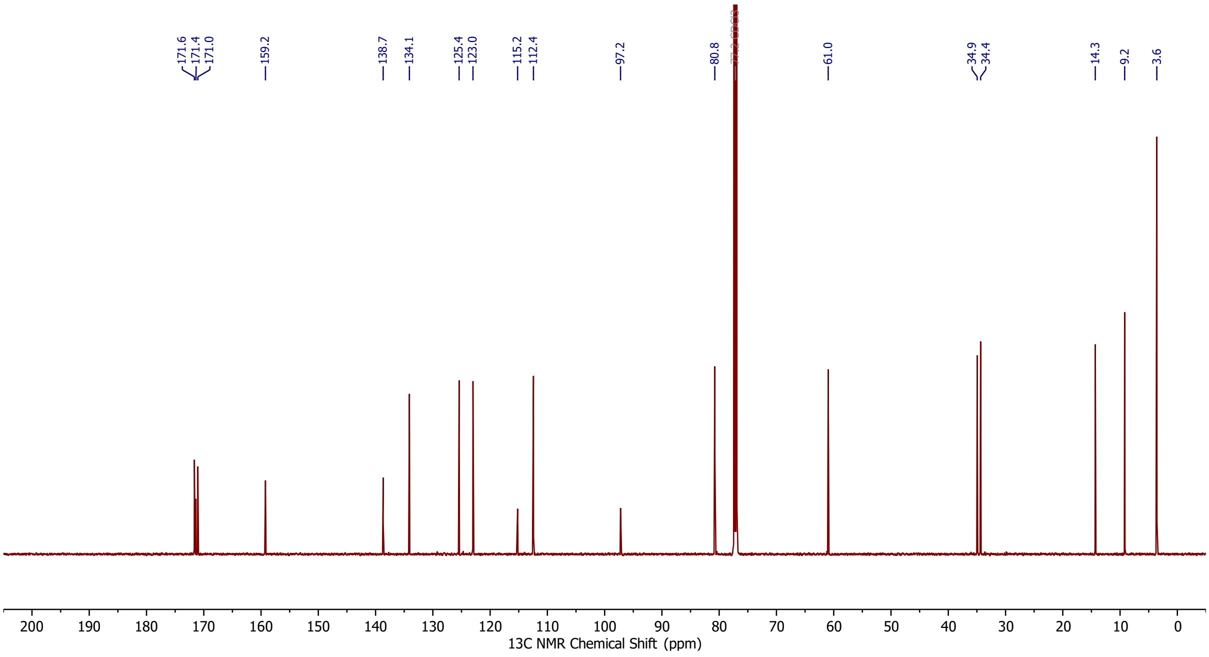

Ethyl *N*-(1-(cyclopropylmethoxy)-4-hydroxy-2-oxo-1,2-dihydroquinoline-3-carbonyl)-*N*-methylglycinate (22a)

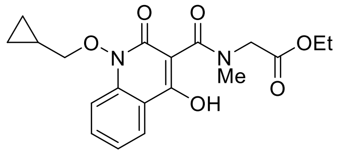

^{1}H NMR (600 MHz, 300 K, $CDCl_3$):

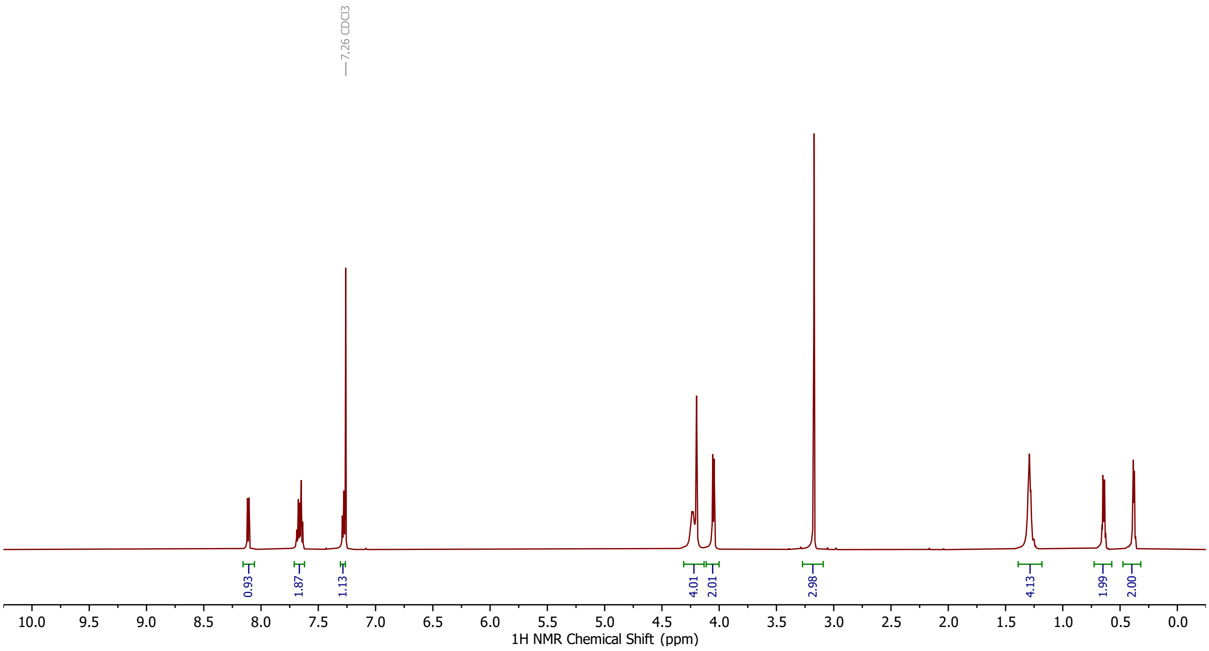

^{13}C NMR (151 MHz, 300 K, $CDCl_3$):

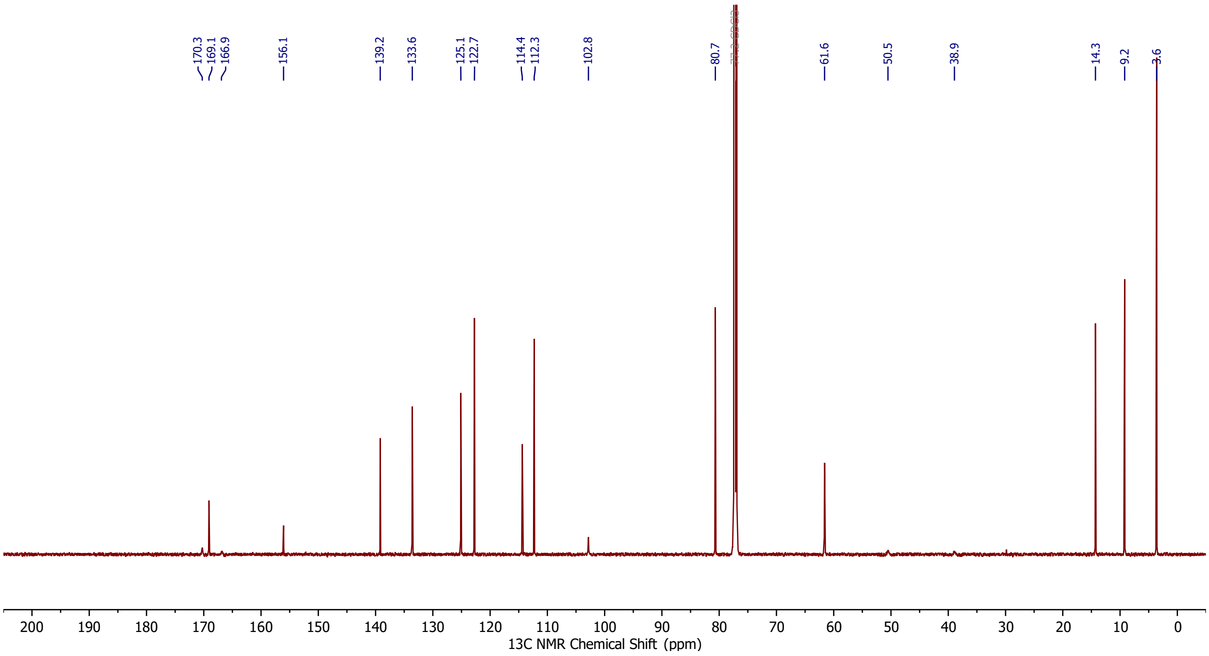

_N_-(1-(Cyclopropylmethoxy)-4-hydroxy-2-oxo-1,2-dihydroquinoline-3-carbonyl)-N-methylglycine (22)

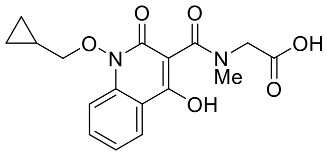

^{1}H NMR (600 MHz, 300 K, DMSO-d_6):

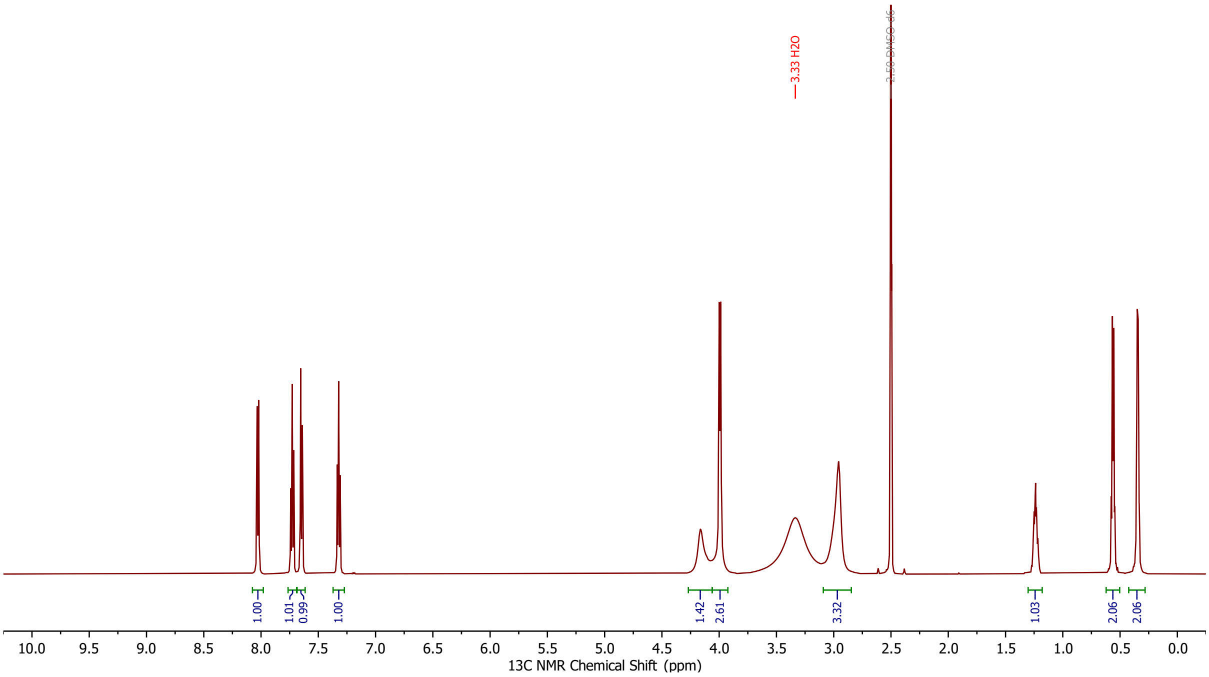

^{13}C NMR (151 MHz, 300 K, DMSO-d_6):

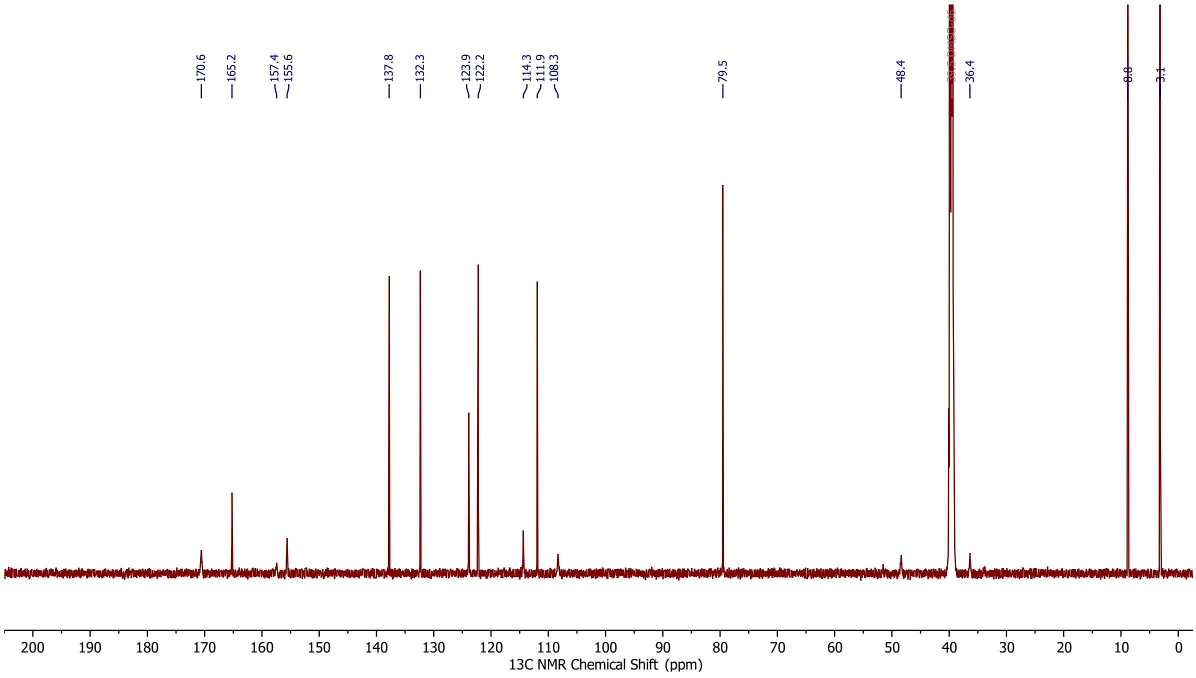

_N_-(2-Amino-2-oxoethyl)-1-(cyclopropylmethoxy)-4-hydroxy-2-oxo-1,2-dihydroquinoline-3-carboxamide (25)

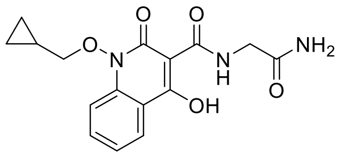

^{1}H NMR (600 MHz, 300 K, DMSO-d_6):

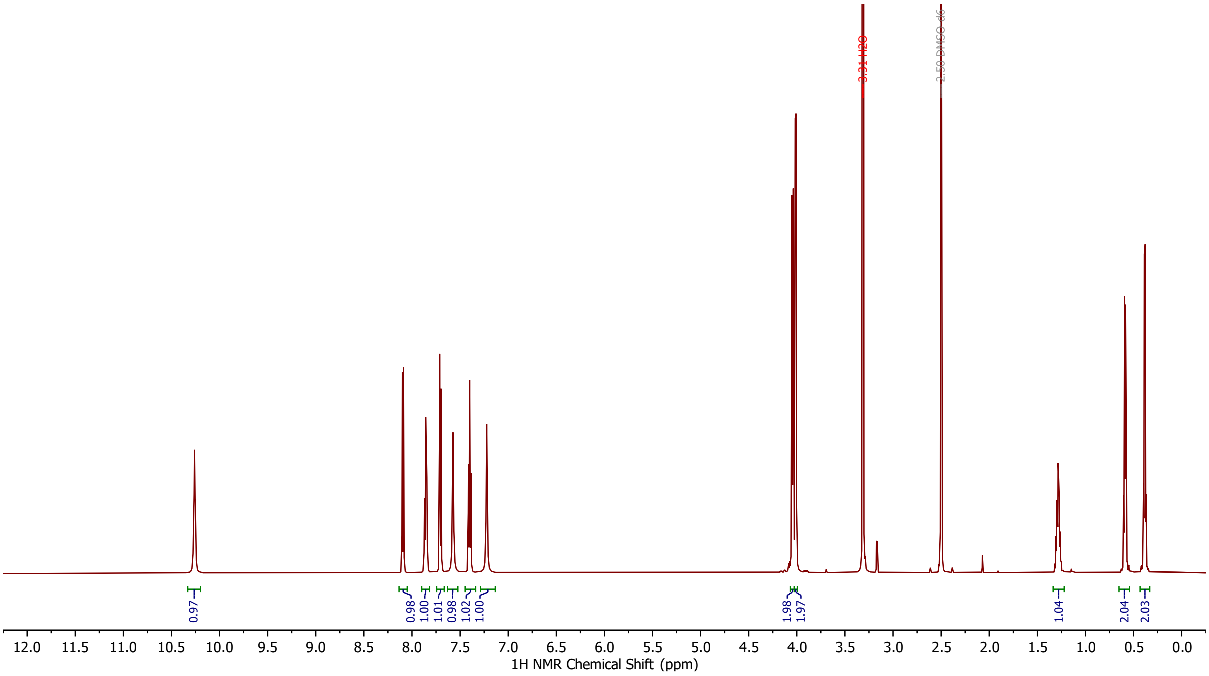

^{13}C NMR (151 MHz, 300 K, DMSO-d_6):

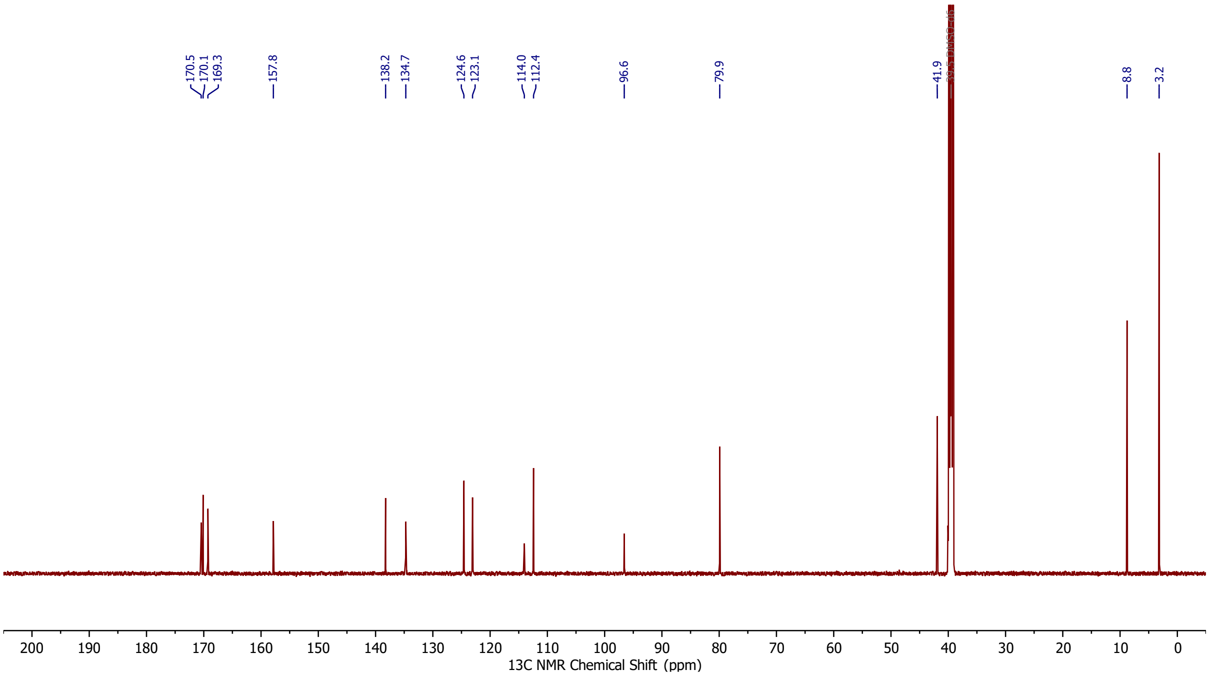

1-(Cyclopropylmethoxy)-4-hydroxy-*N*-(2-(methylamino)-2-oxoethyl)-2-oxo-1,2-dihydroquinoline-3-carboxamide (26)

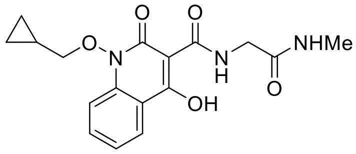

^{1}H NMR (600 MHz, 300 K, DMSO-d_6):

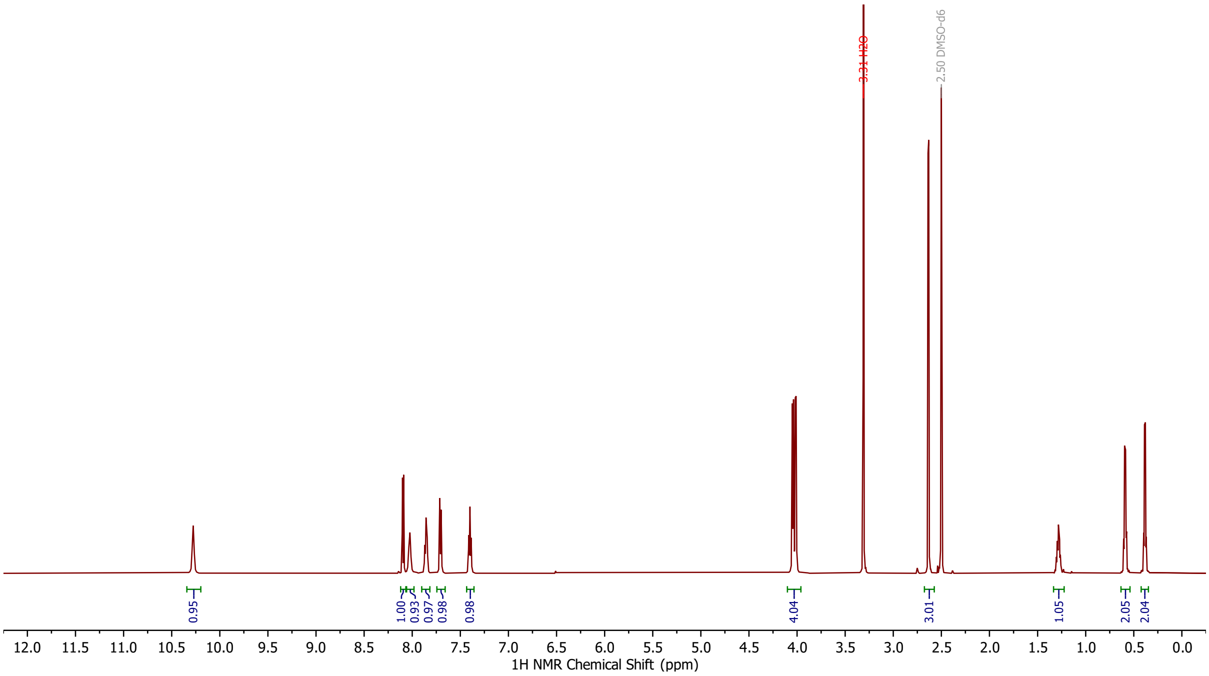

^{13}C NMR (151 MHz, 300 K, DMSO-d_6):

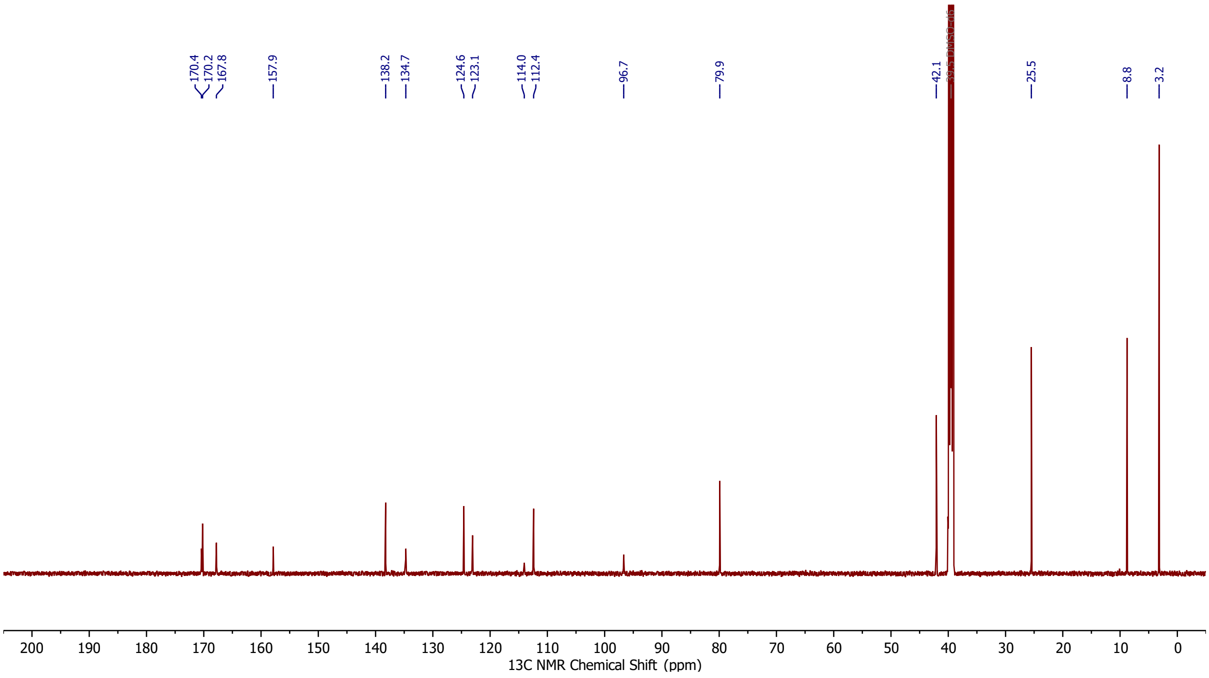

(±)-1-(Cyclopropylmethoxy)-4-hydroxy-2-oxo-*N*-(3,3,3-trifluoro-2-hydroxypropyl)-1,2-dihydroquinoline-3-carboxamide (28)

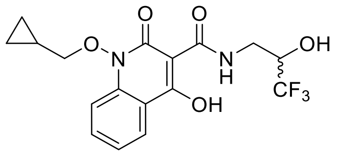

^{1}H NMR (600 MHz, 300 K, $CDCl_3$):

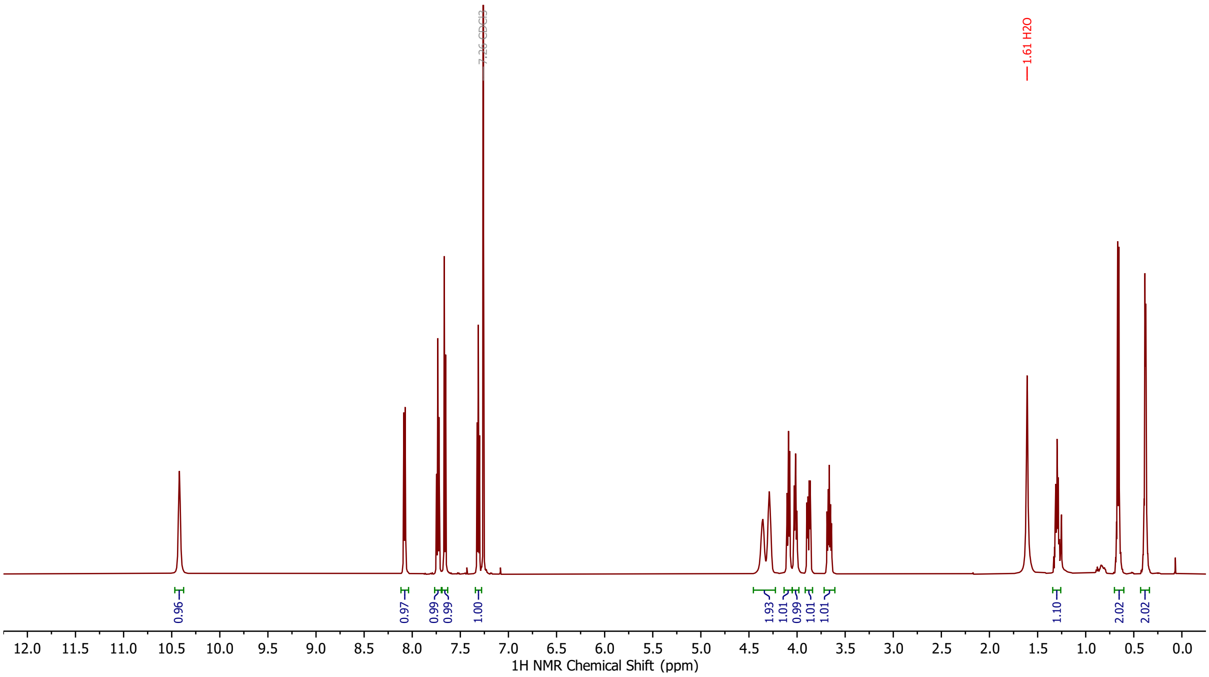

^{13}C NMR (151 MHz, 300 K, $CDCl_3$):

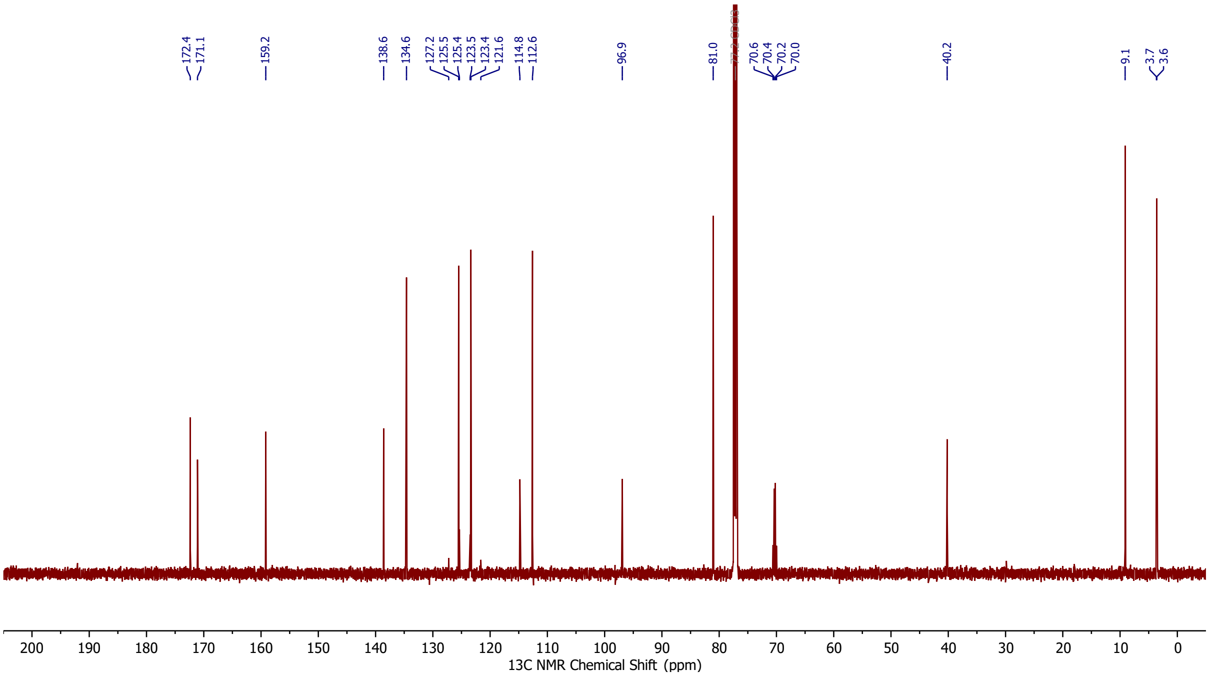

1-(Cyclopropylmethoxy)-4-hydroxy-*N*-((3-hydroxyoxetan-3-yl)methyl)-2-oxo-1,2-dihydroquinoline-3-carboxamide (29)

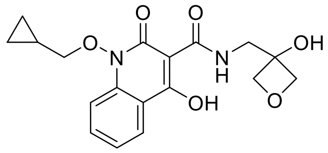

^{1}H NMR (700 MHz, 300 K, $CDCl_3$):

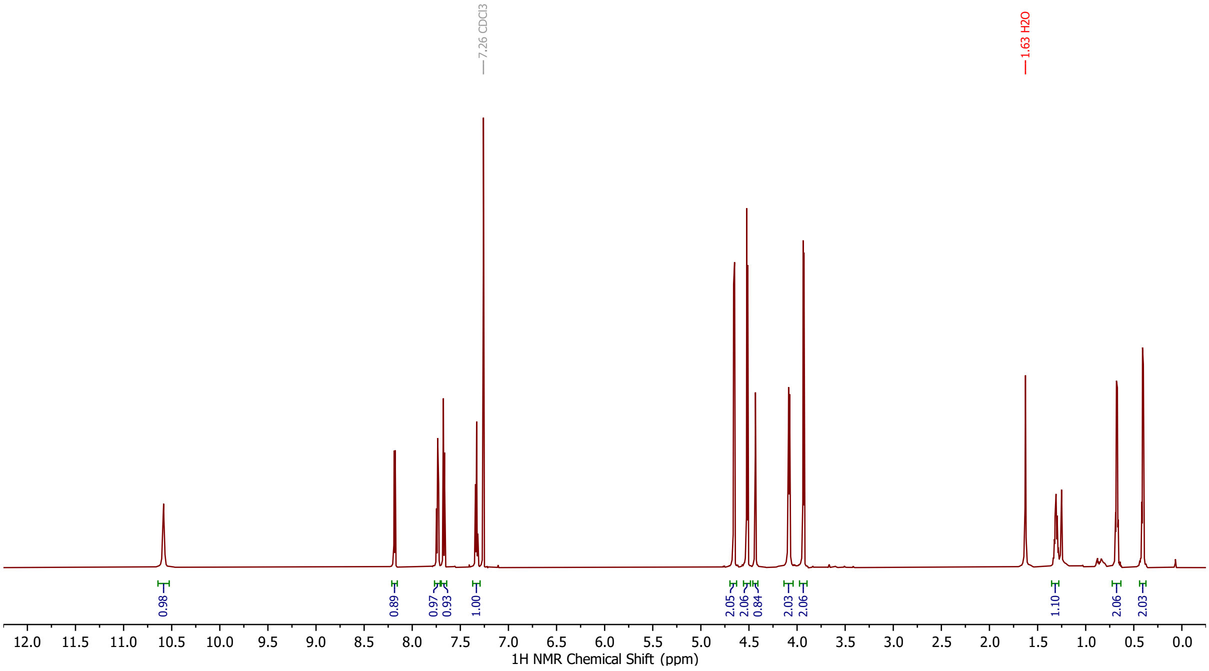

^{13}C NMR (176 MHz, 300 K, $CDCl_3$):

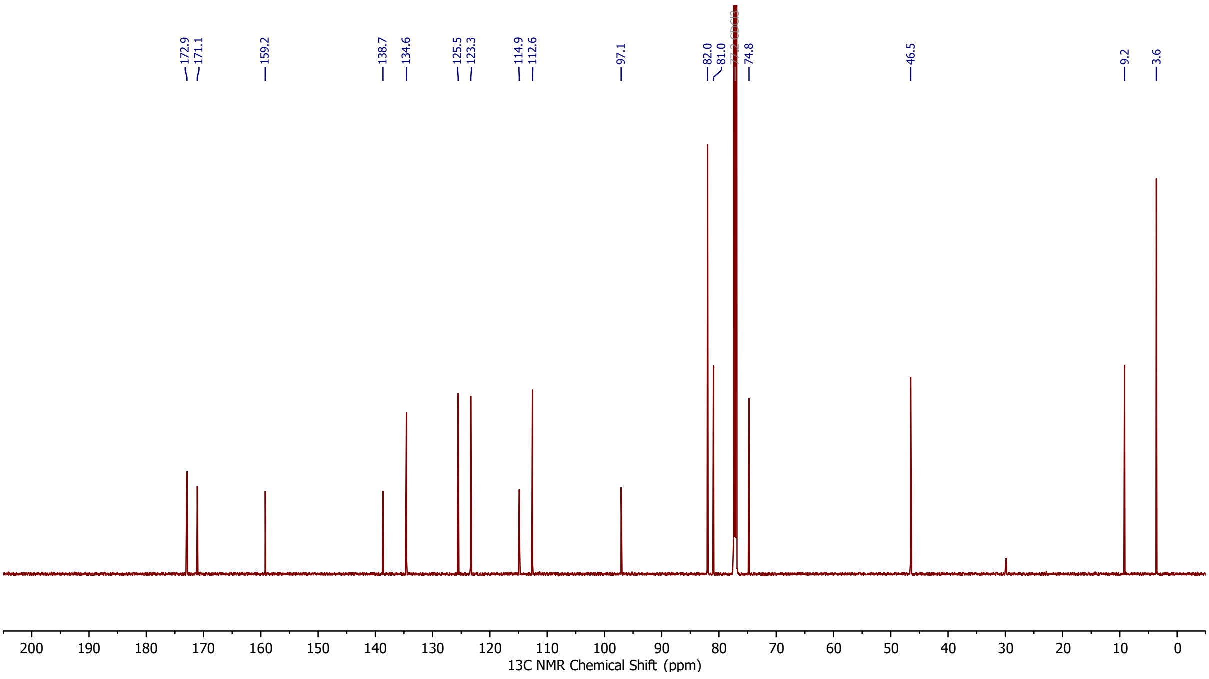

_N_-((1_H_-1,2,3-Triazol-1-yl)methyl)-1-(cyclopropylmethoxy)-4-hydroxy-2-oxo-1,2-dihydroquinoline-3-carboxamide (30)

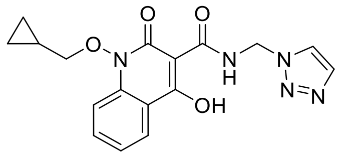

^{1}H NMR (600 MHz, 300 K, DMSO-d_6):

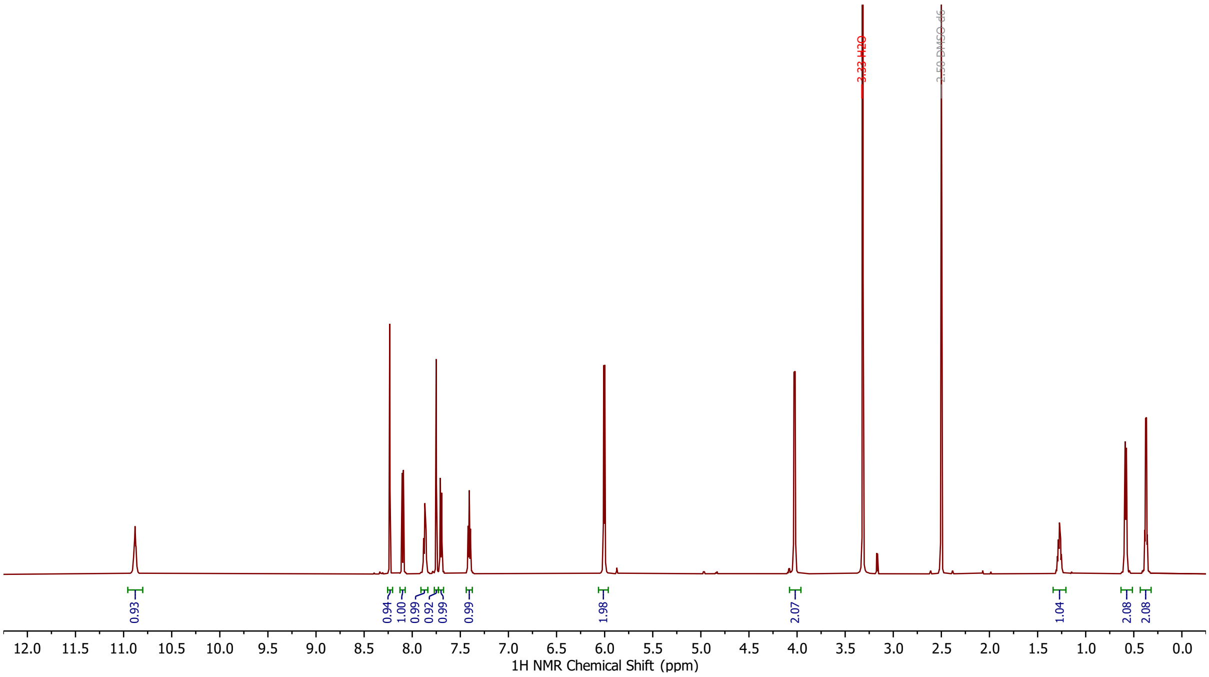

^{13}C NMR (151 MHz, 300 K, DMSO-d_6):

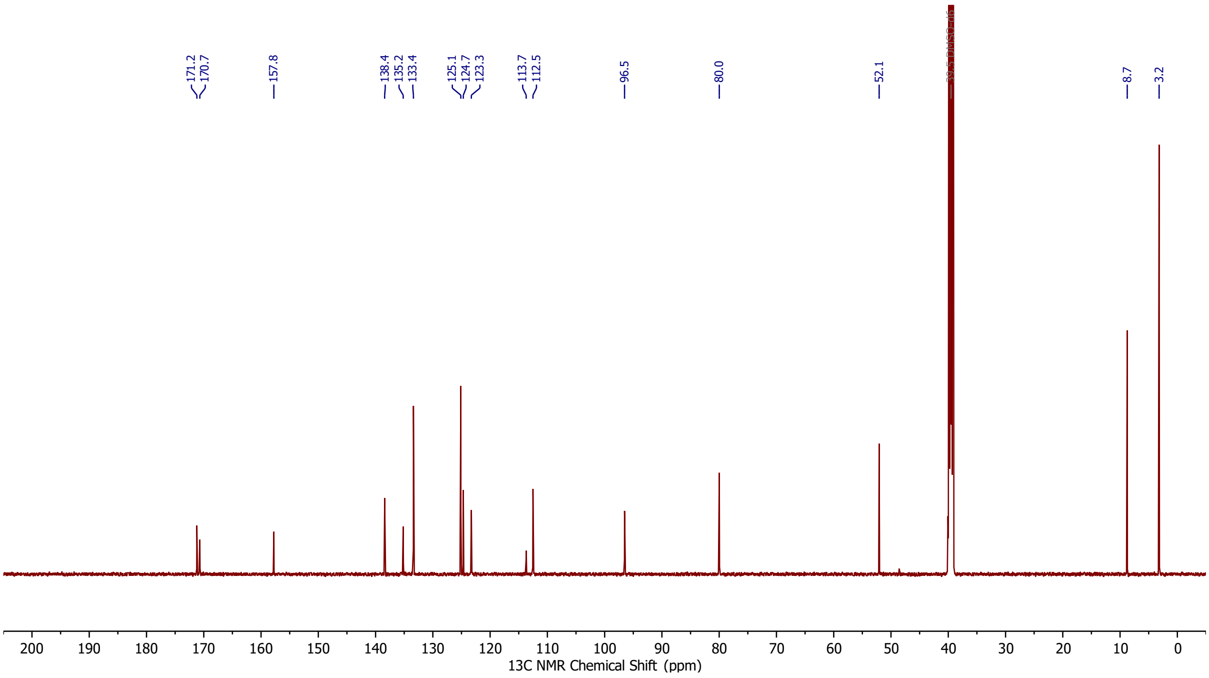

***N*-((1*H*-1,2,4-Triazol-1-yl)methyl)-1-(cyclopropylmethoxy)-4-hydroxy-2-oxo-1,2-dihydroquinoline-3-carboxamide (31)**

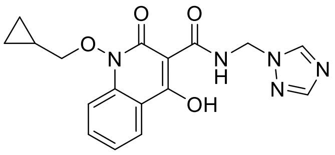

^{1}H NMR (600 MHz, 300 K, DMSO-d_6):

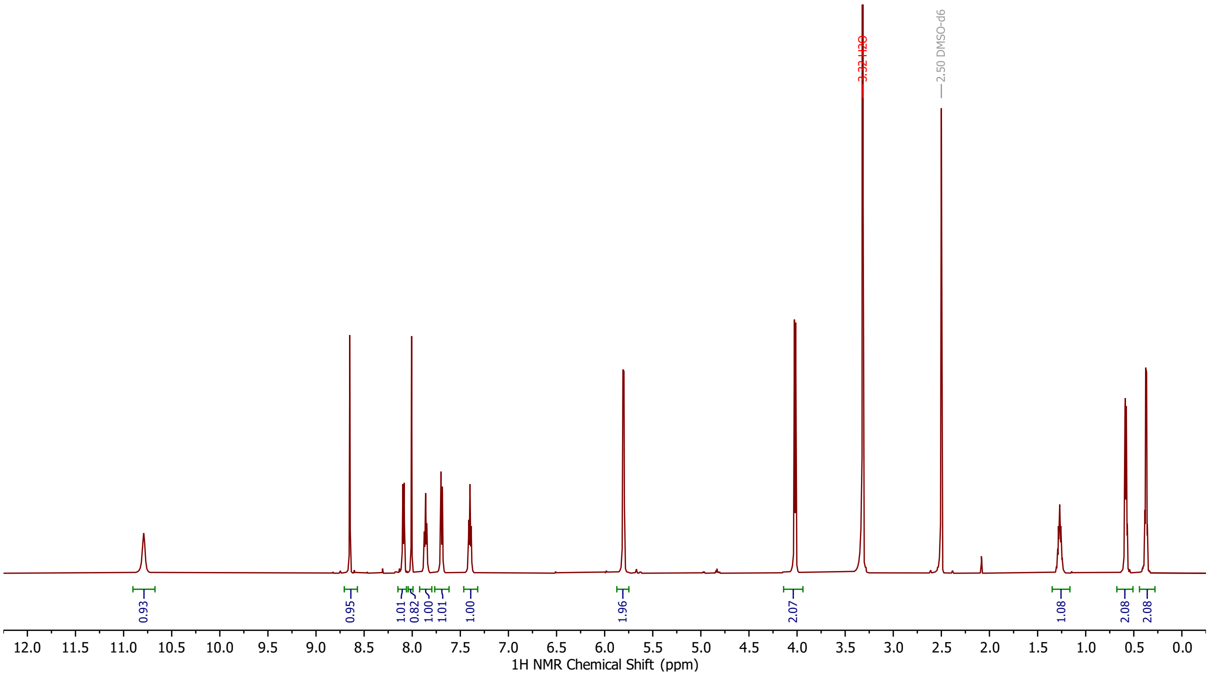

^{13}C NMR (151 MHz, 300 K, DMSO-d_6):

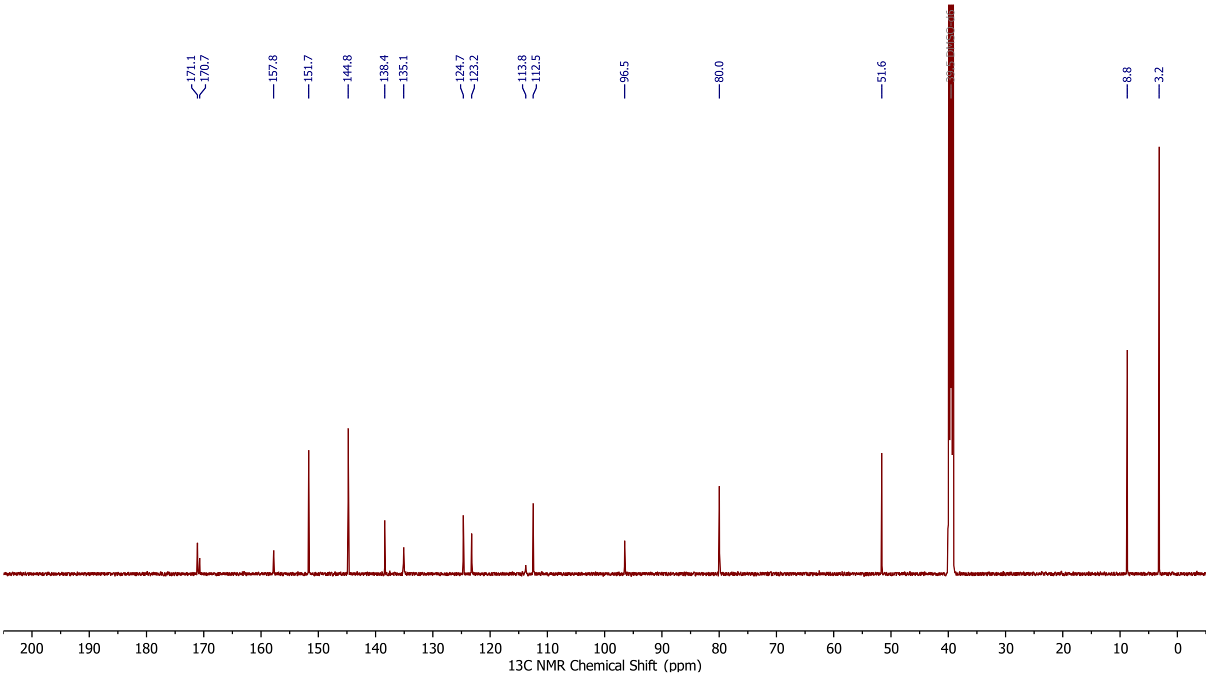

N-((2H-Tetrazol-5-yl)methyl)-1-(cyclopropylmethoxy)-4-hydroxy-2-oxo-1,2-dihydroquinoline-3-carboxamide (32)

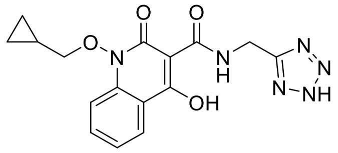

^{1}H NMR (600 MHz, 300 K, DMSO-d_6):

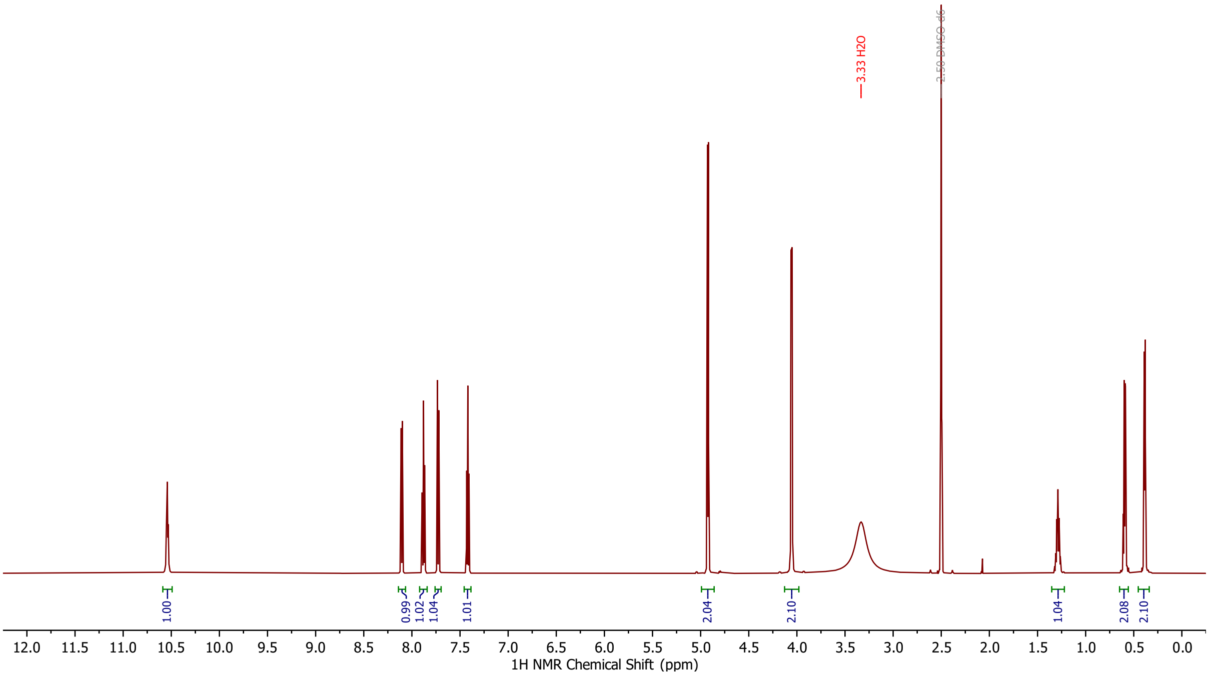

^{13}C NMR (151 MHz, 300 K, DMSO-d_6):

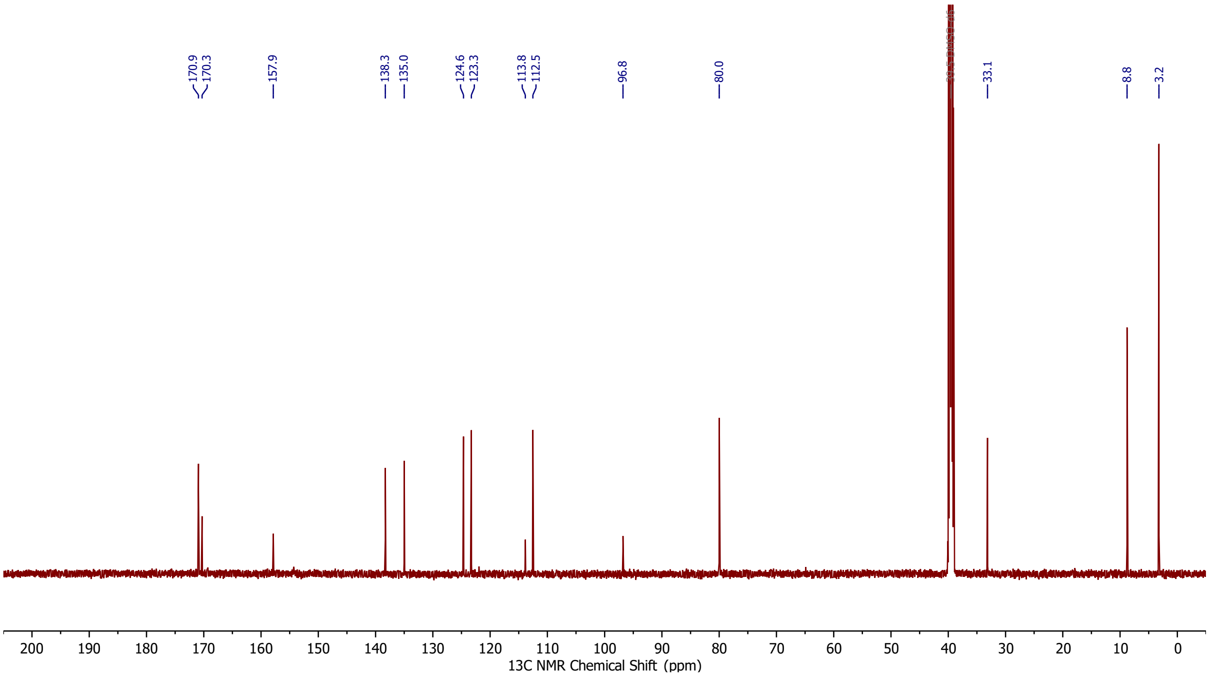

Ethyl 2-((benzyloxy)(*tert*-butoxycarbonyl)amino)benzoate (34)

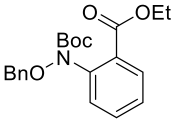

^{1}H NMR (600 MHz, 300 K, $CDCl_3$):

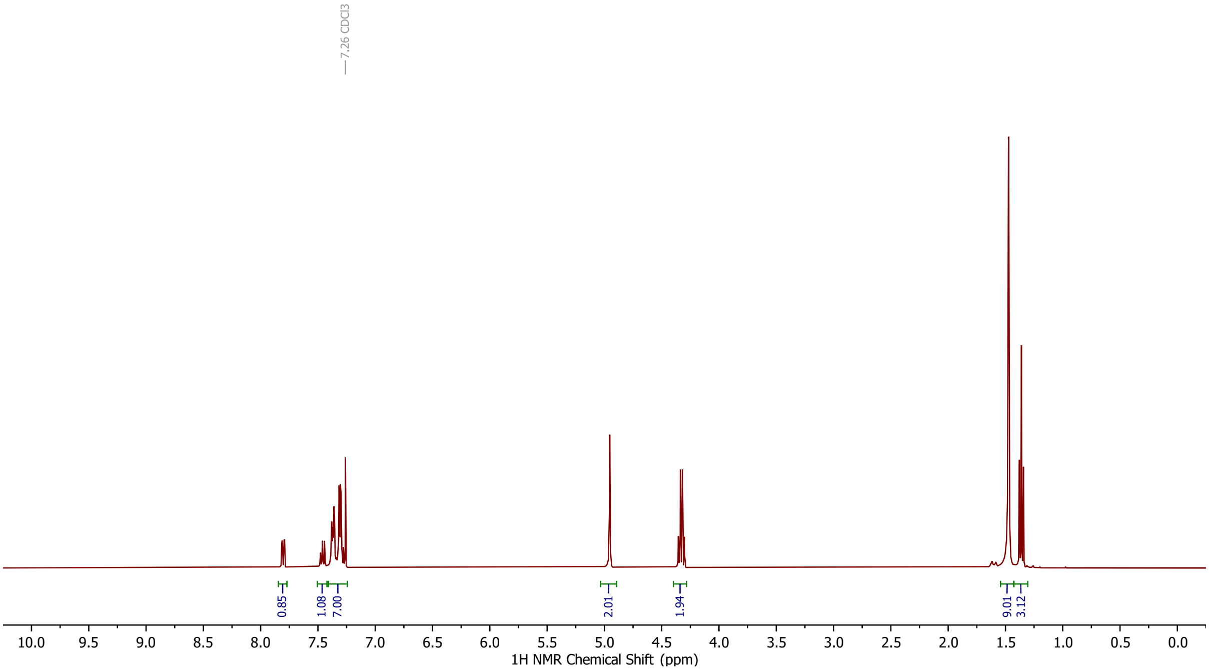

^{13}C NMR (151 MHz, 300 K, $CDCl_3$):

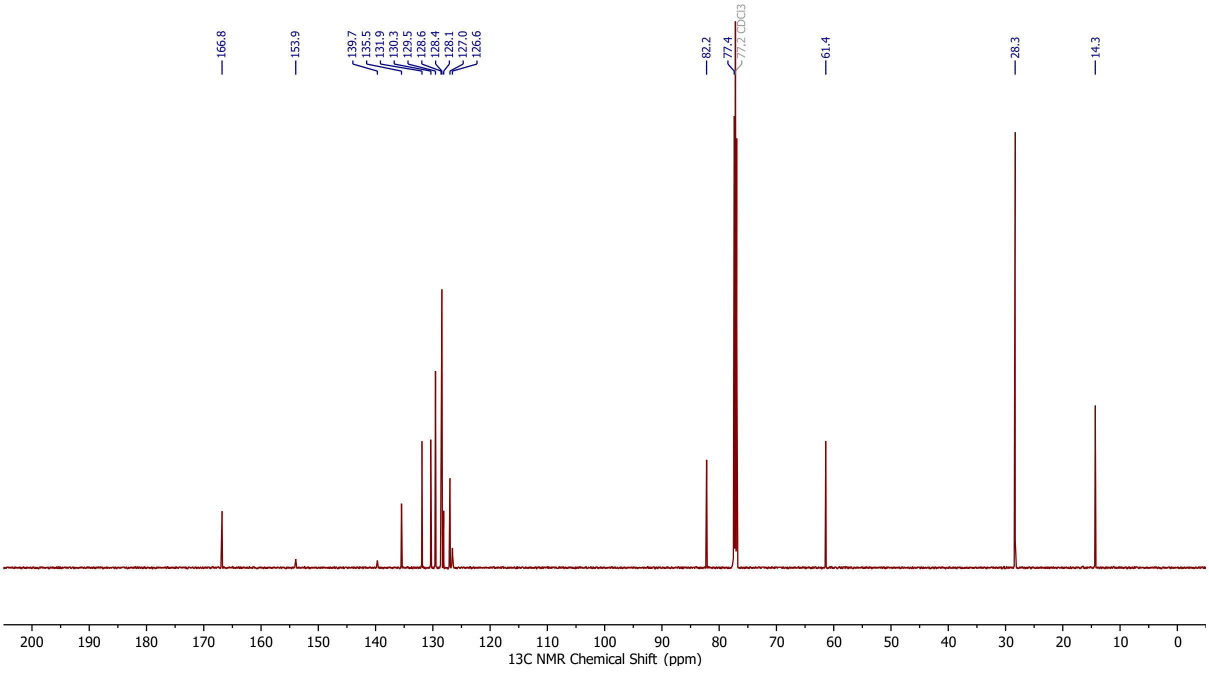

tert-Butyl (1-(benzyloxy)-4-hydroxy-2-oxo-1,2-dihydroquinoline-3-carbonyl)glycinate (38)

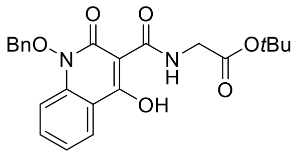

^{1}H NMR (600 MHz, 300 K, $CDCl_3$):

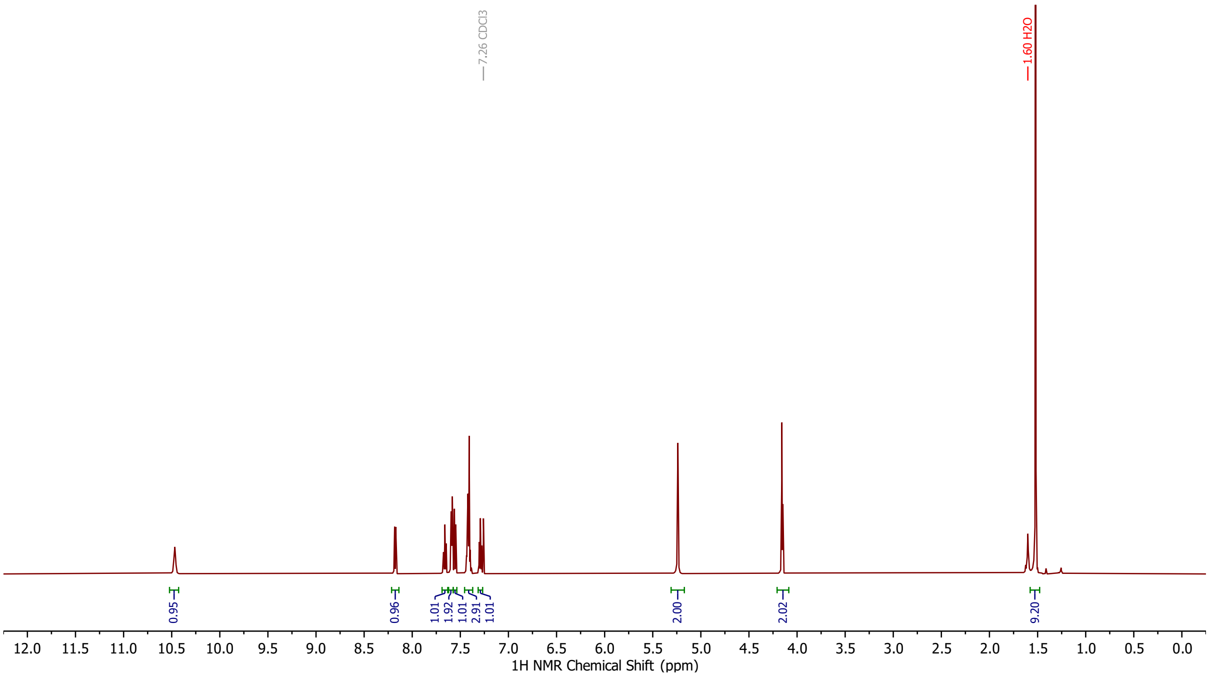

^{13}C NMR (151 MHz, 300 K, $CDCl_3$):

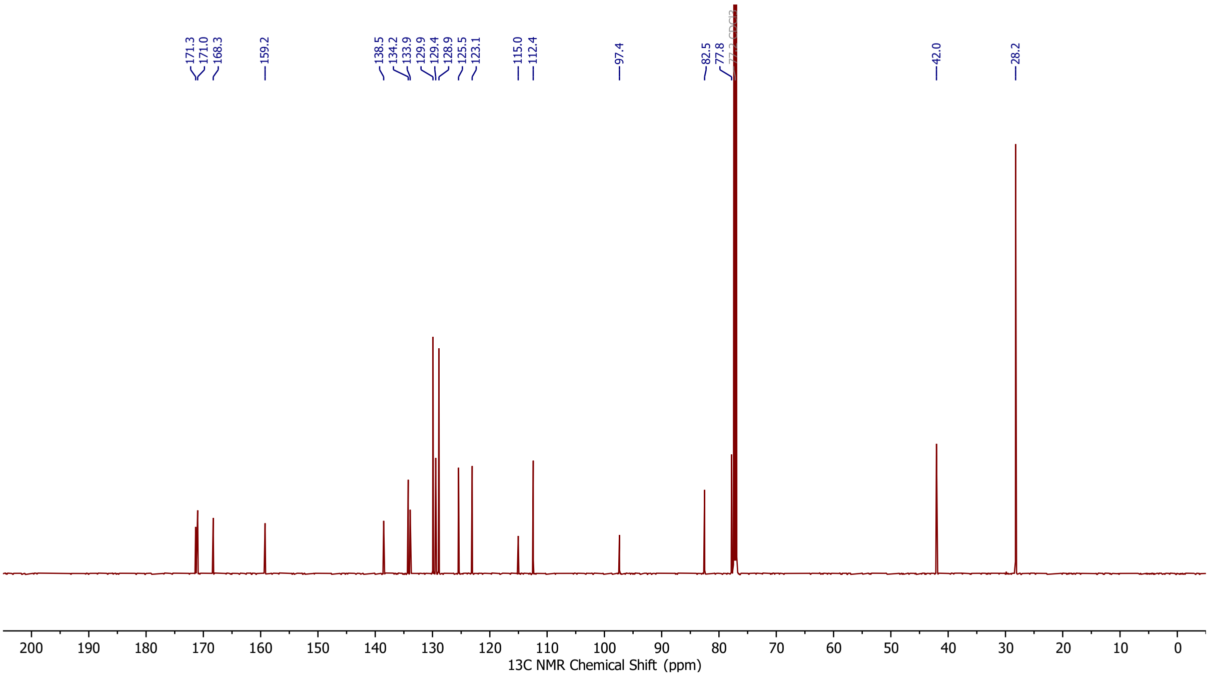

tert-Butyl (1,4-dihydroxy-2-oxo-1,2-dihydroquinoline-3-carbonyl)glycinate (39)

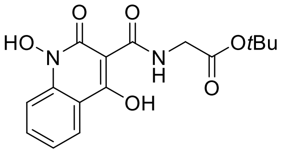

^{1}H NMR (600 MHz, 300 K, $CDCl_3$):

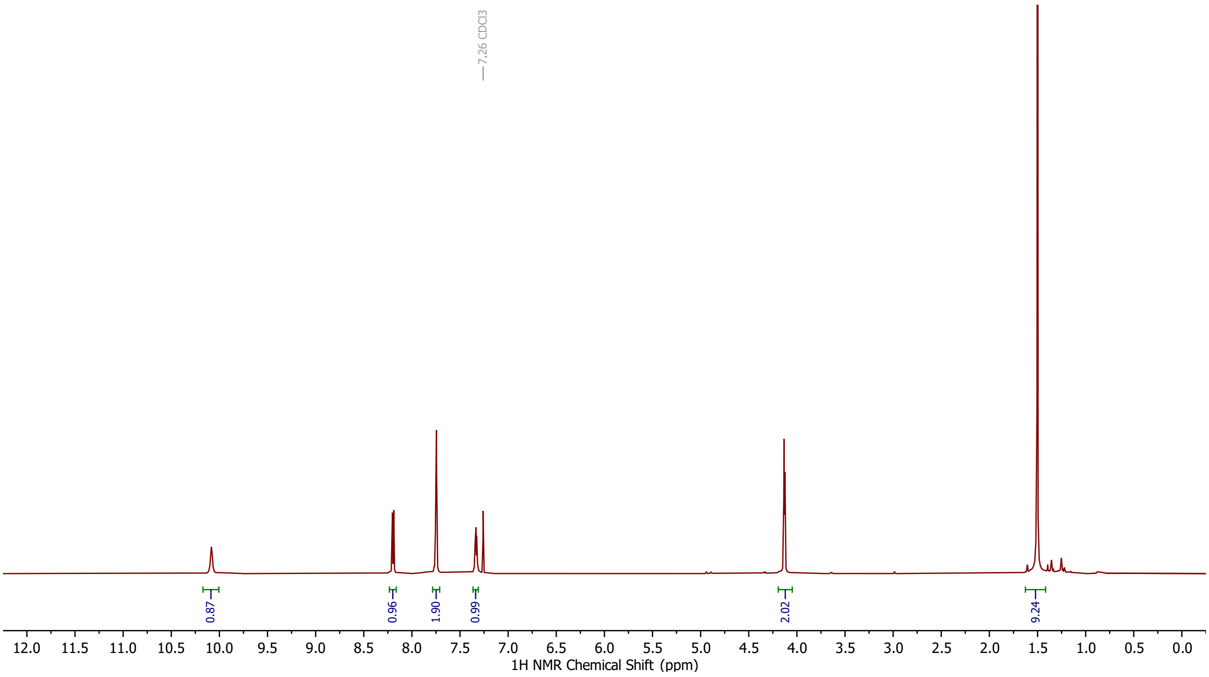

^{13}C NMR (151 MHz, 300 K, $CDCl_3$):

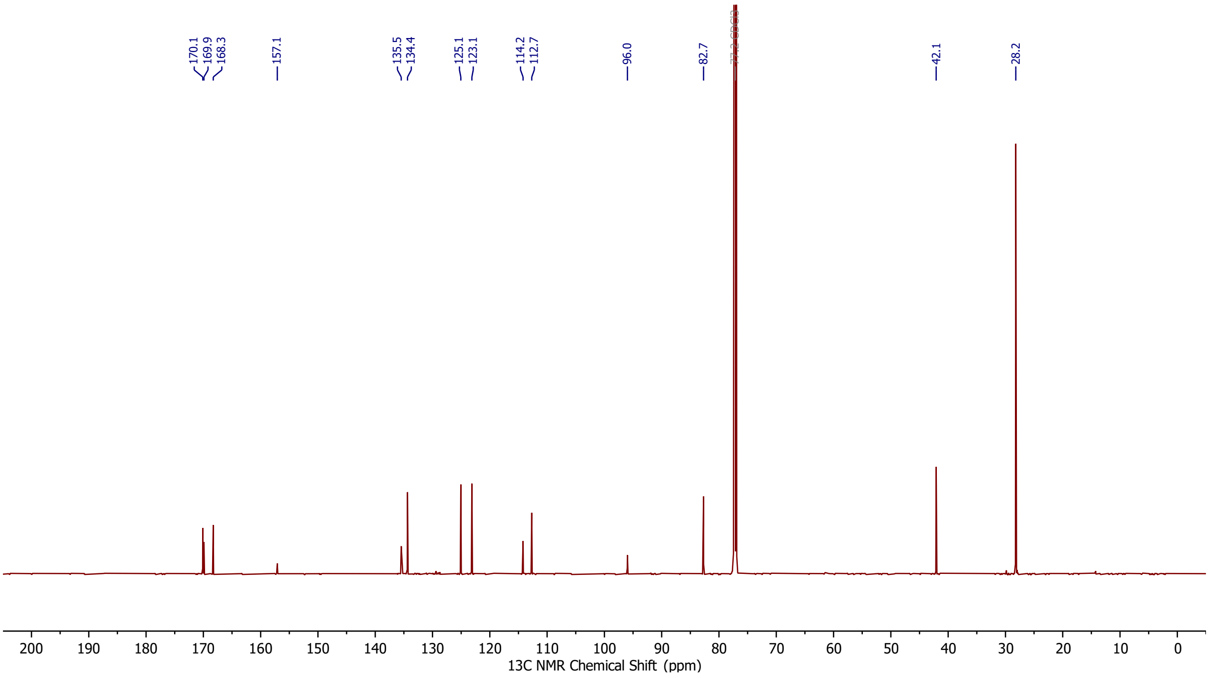

***tert*-Butyl (4-hydroxy-2-oxo-1-(pyridin-2-ylmethoxy)-1,2-dihydroquinoline-3-carbonyl)glycinate (40a)**

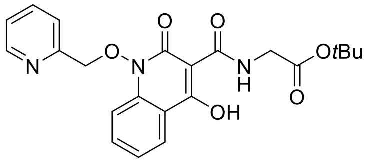

^{1}H NMR (500 MHz, 300 K, $CDCl_3$):

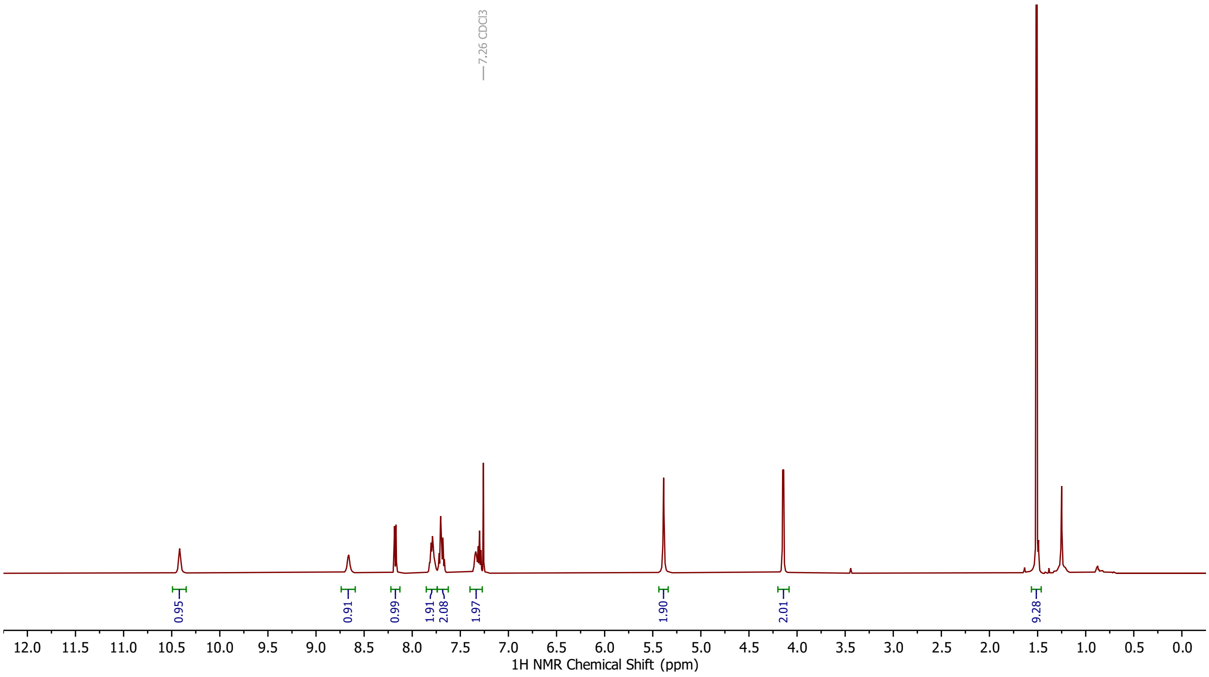

^{13}C NMR (126 MHz, 300 K, $CDCl_3$):

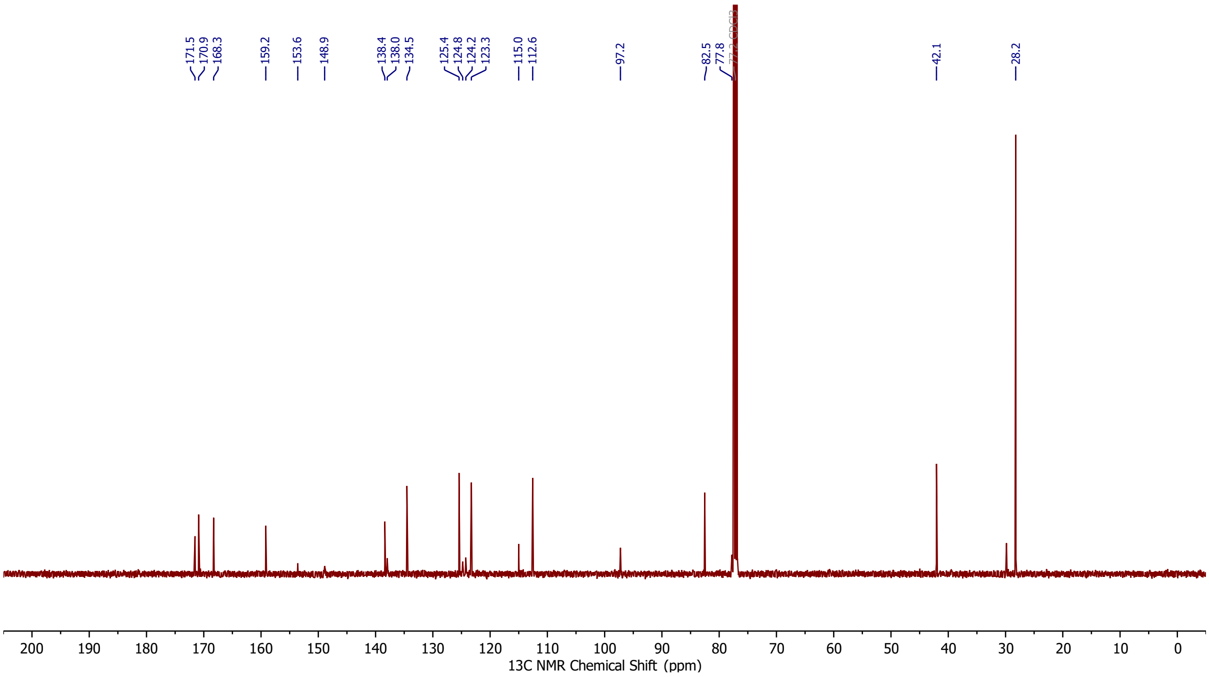

tert-Butyl (4-hydroxy-2-oxo-1-(thiazol-4-ylmethoxy)-1,2-dihydroquinoline-3-carbonyl)glycinate (41a)

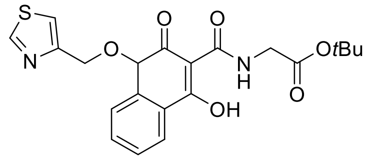

^1H NMR (600 MHz, 300 K, $CDCl_3$):

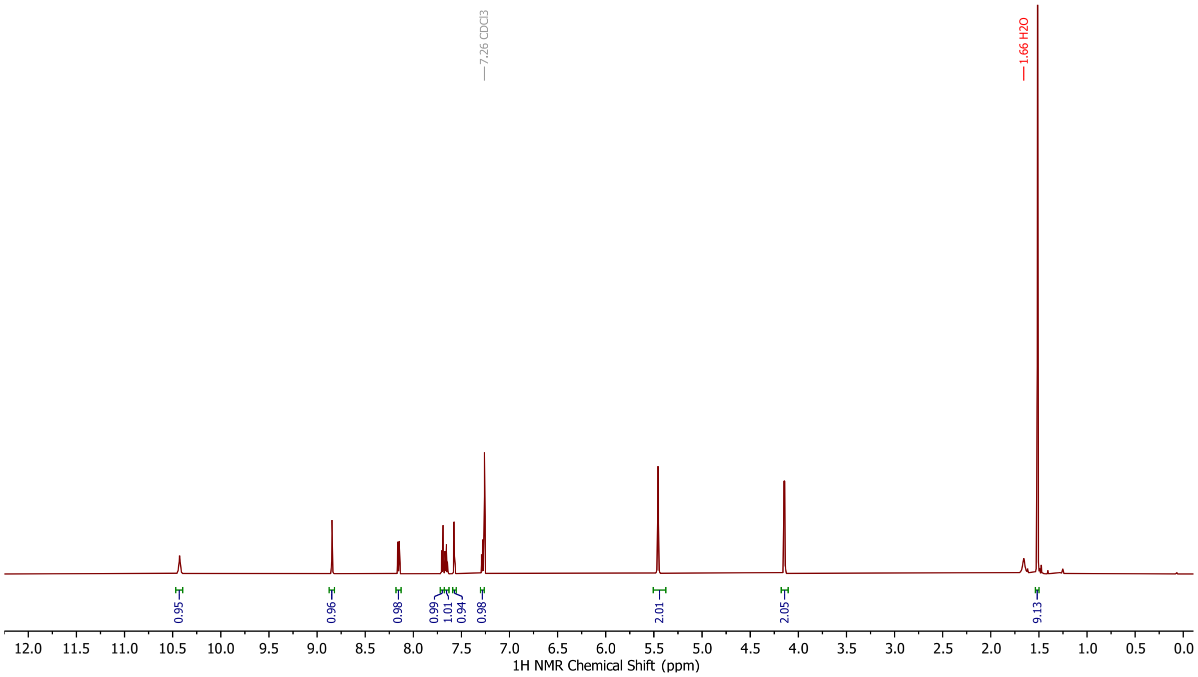

^{13}C NMR (151 MHz, 300 K, $CDCl_3$):

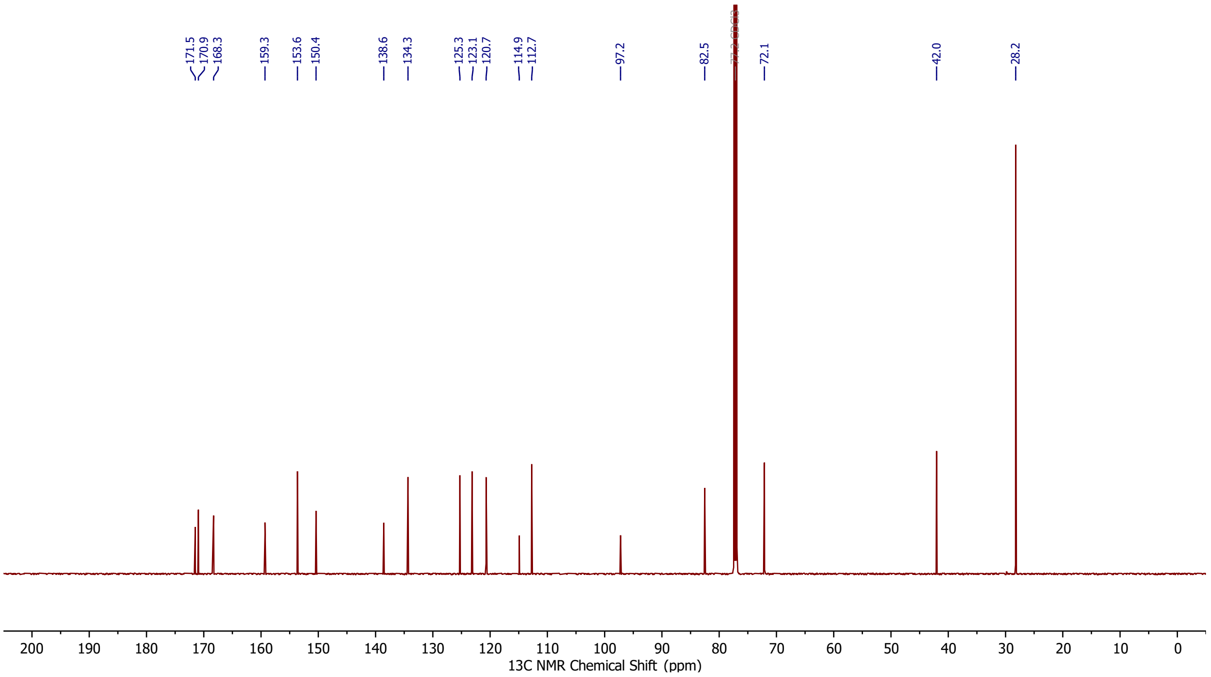

(4-Hydroxy-2-oxo-1-(thiazol-4-ylmethoxy)-1,2-dihydroquinoline-3-carbonyl)glycine (41)

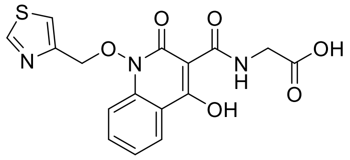

^{1}H NMR (600 MHz, 300 K, DMSO-d_6):

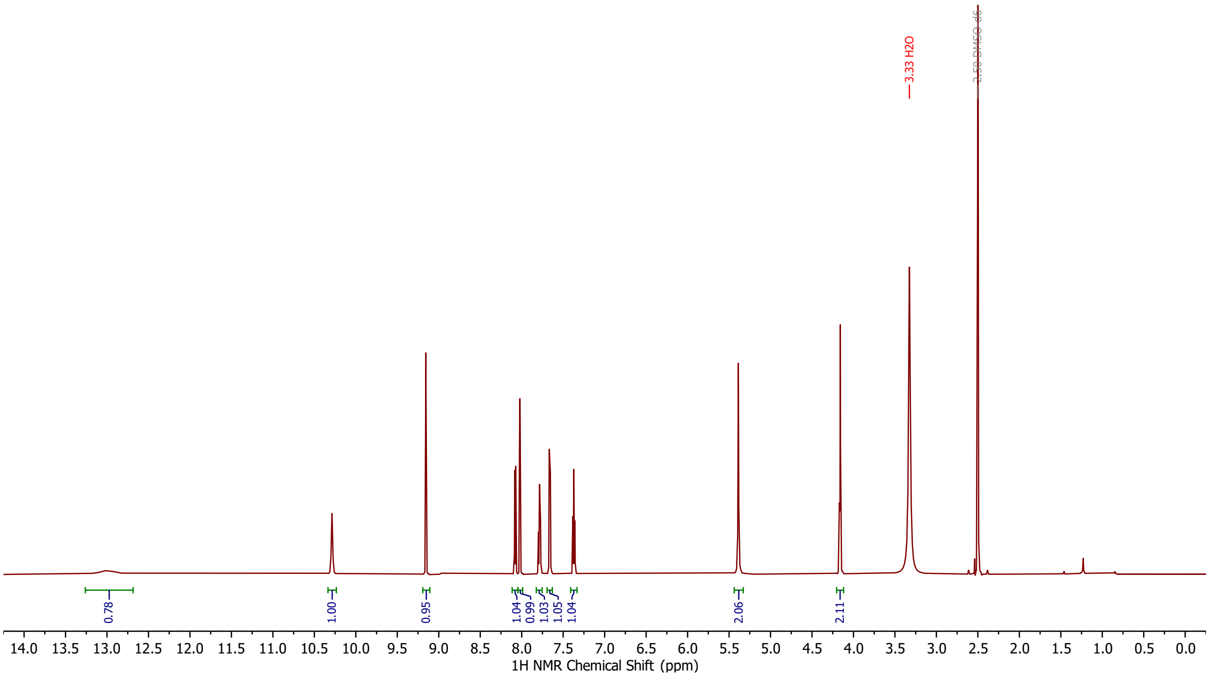

^{13}C NMR (151 MHz, 300 K, DMSO-d_6):

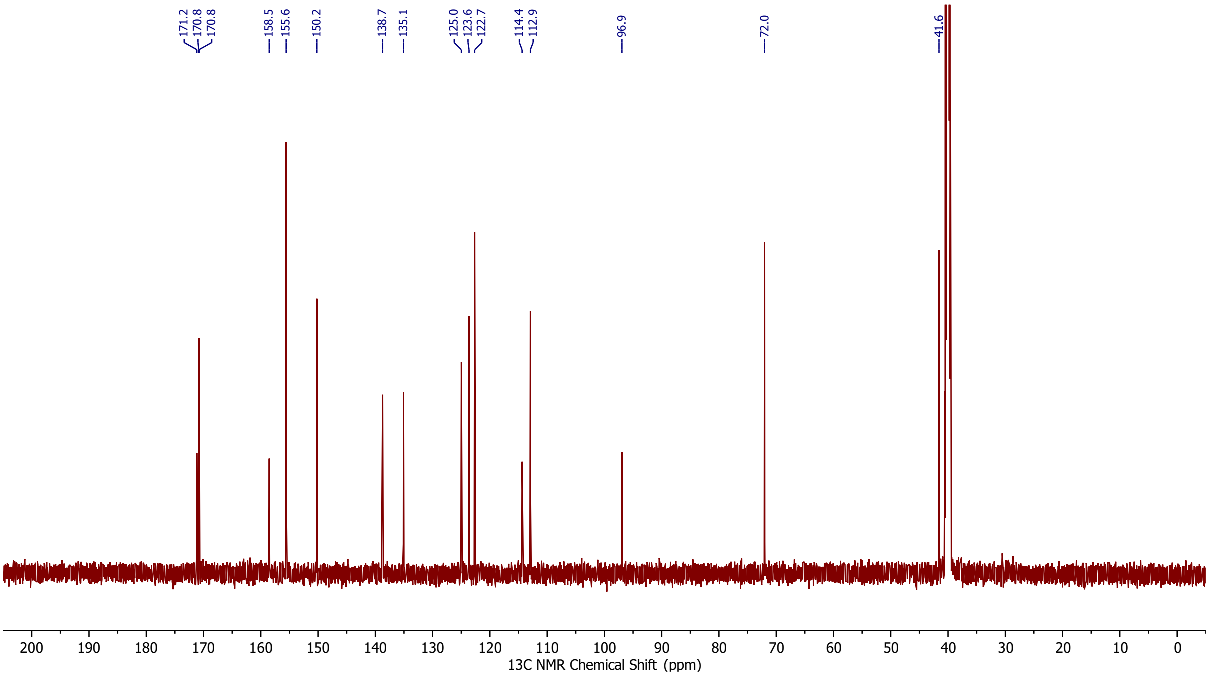

***tert*-Butyl (4-hydroxy-1-(isoxazol-3-ylmethoxy)-2-oxo-1,2-dihydroquinoline-3-carbonyl)glycinate (42a)**

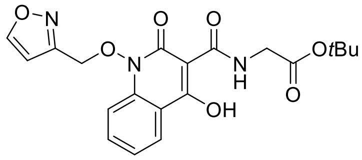

^{1}H NMR (500 MHz, 300 K, $CDCl_3$):

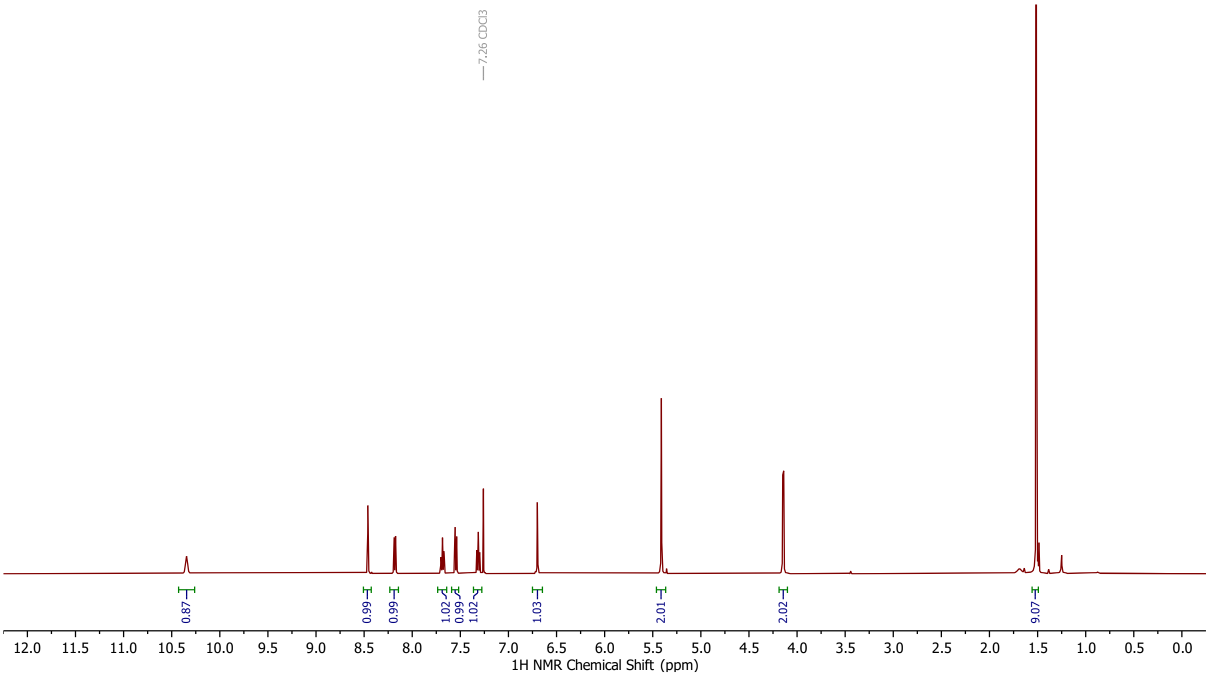

^{13}C NMR (126 MHz, 300 K, $CDCl_3$):

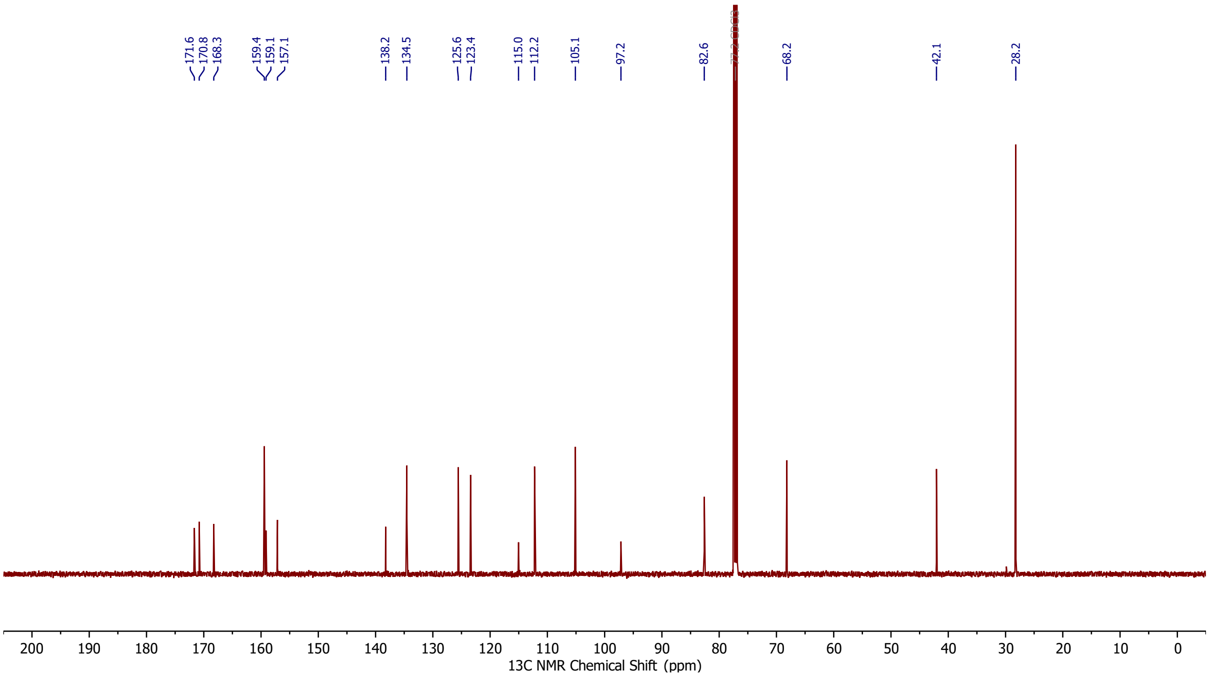

(4-Hydroxy-1-(isoxazol-3-ylmethoxy)-2-oxo-1,2-dihydroquinoline-3-carbonyl)glycine (42)

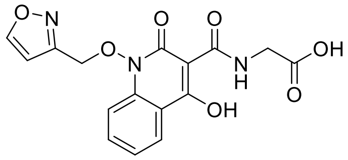

^{1}H NMR (500 MHz, 300 K, DMSO-d_6):

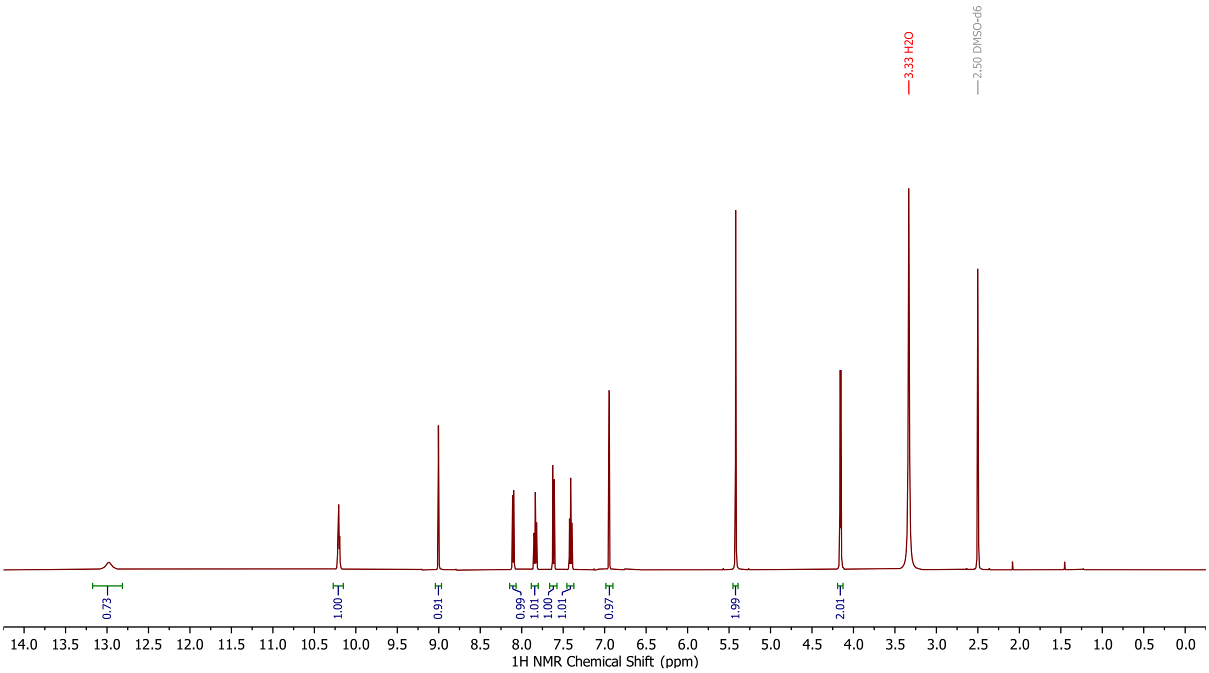

^{13}C NMR (126 MHz, 300 K, DMSO-d_6):

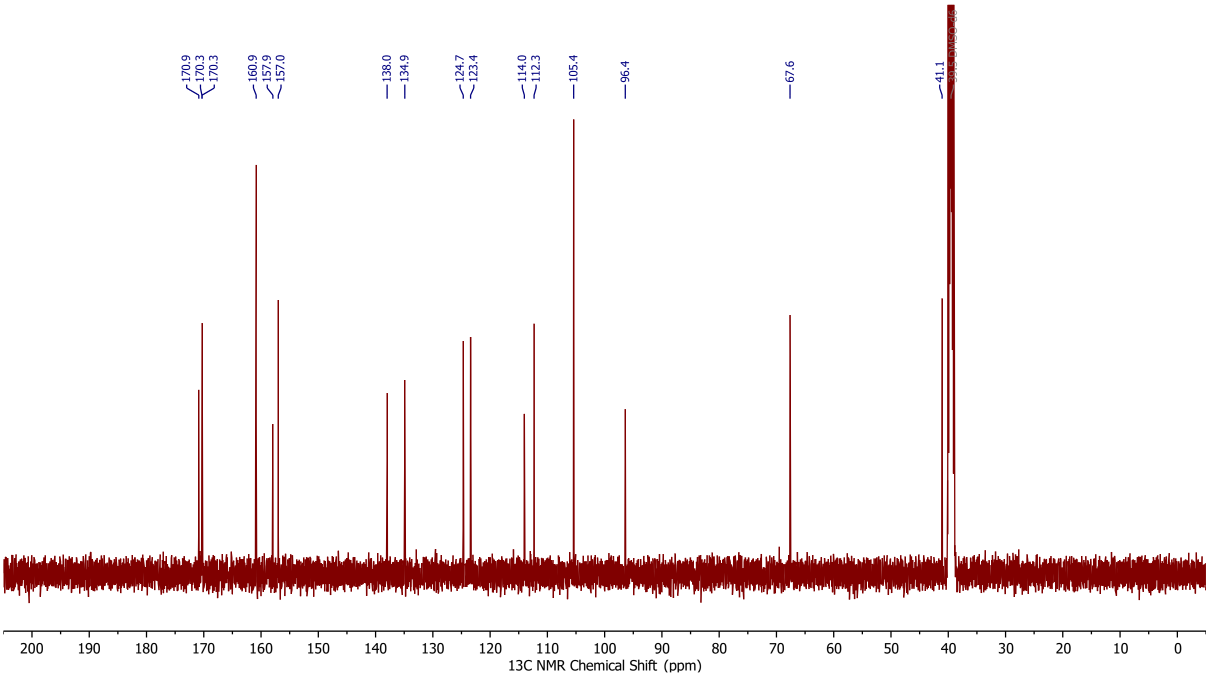

tert-Butyl (4-hydroxy-1-((1-methyl-1H-imidazol-4-yl)methoxy)-2-oxo-1,2-dihydroquinoline-3-carbonyl)glycinate (43a)

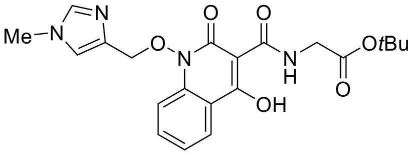

^{1}H NMR (500 MHz, 300 K, $CDCl_3$):

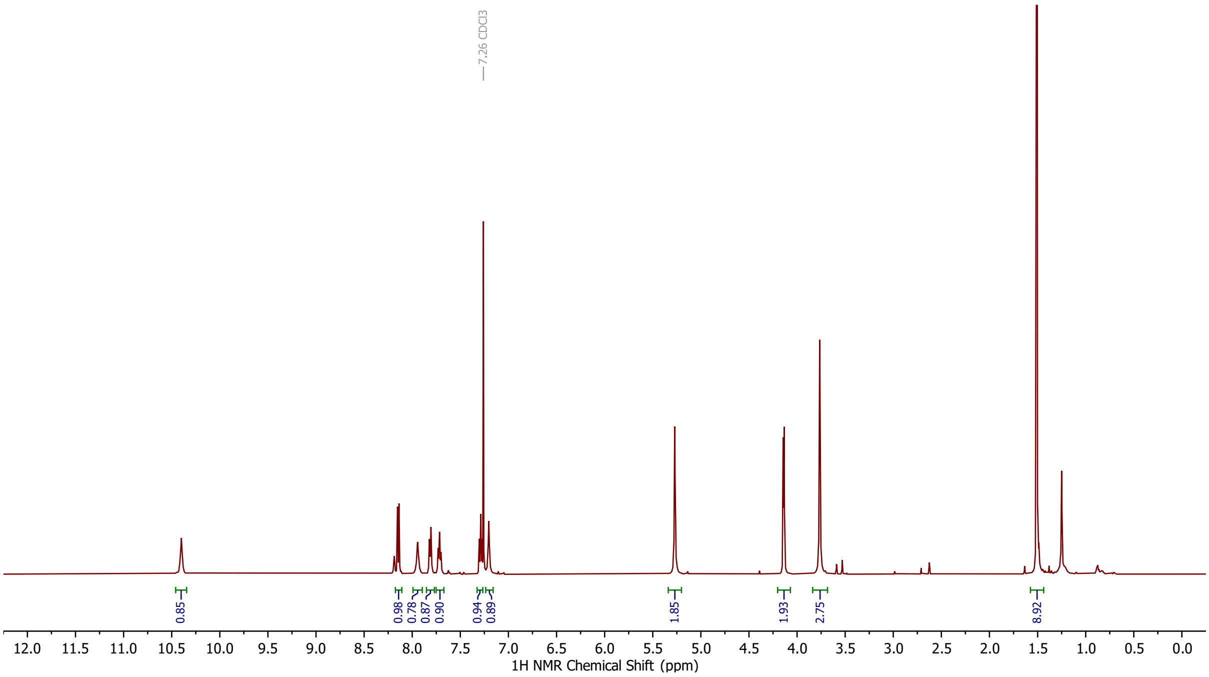

^{13}C NMR (126 MHz, 300 K, $CDCl_3$):

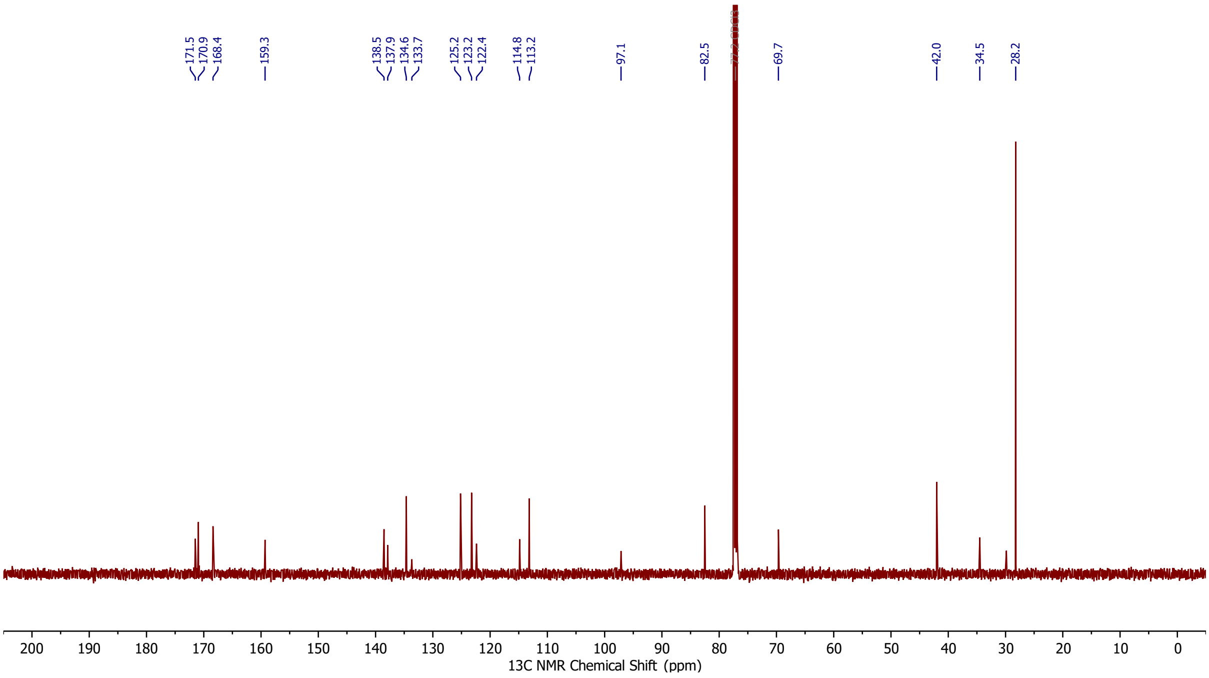

(4-Hydroxy-1-((1-methyl-1*H*-imidazol-4-yl)methoxy)-2-oxo-1,2-dihydroquinoline-3-carbonyl)glycine (43)

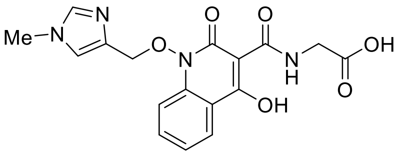

^{1}H NMR (600 MHz, 300 K, DMSO-d_6):

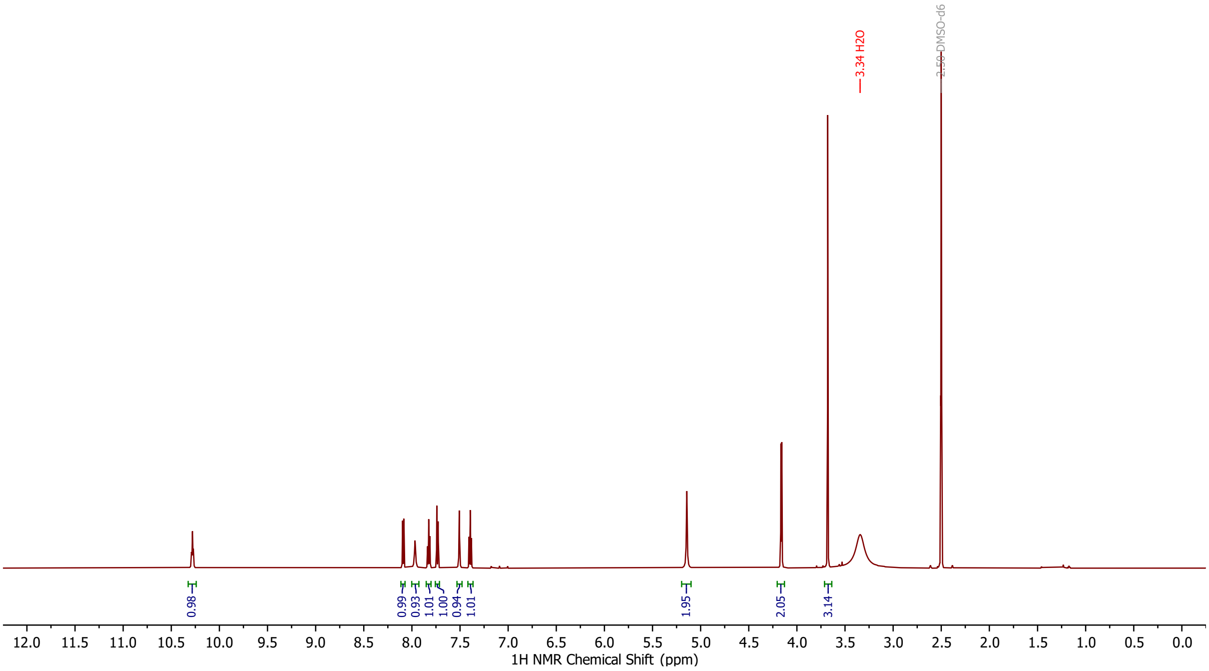

^{13}C NMR (151 MHz, 300 K, DMSO-d_6):

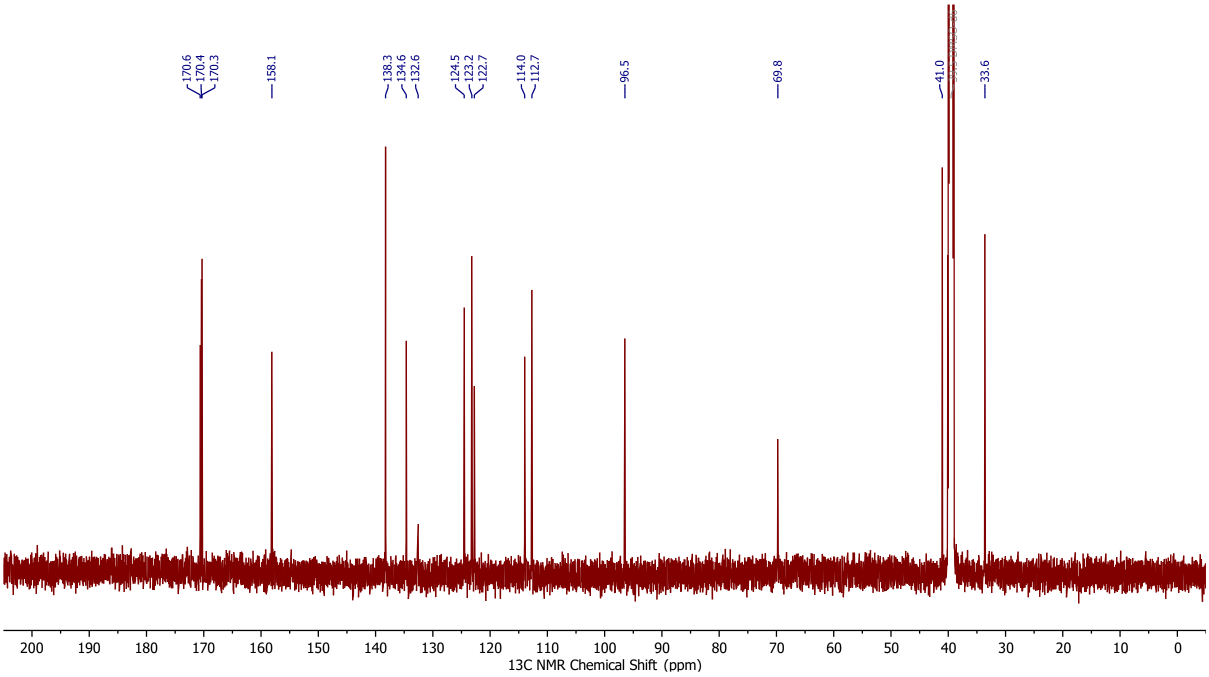

tert-Butyl (4-hydroxy-2-oxo-1-(pyridin-4-ylmethoxy)-1,2-dihydroquinoline-3-carbonyl)glycinate (44a)

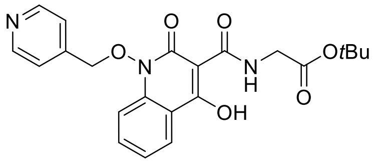

^1H NMR (500 MHz, 300 K, $CDCl_3$):

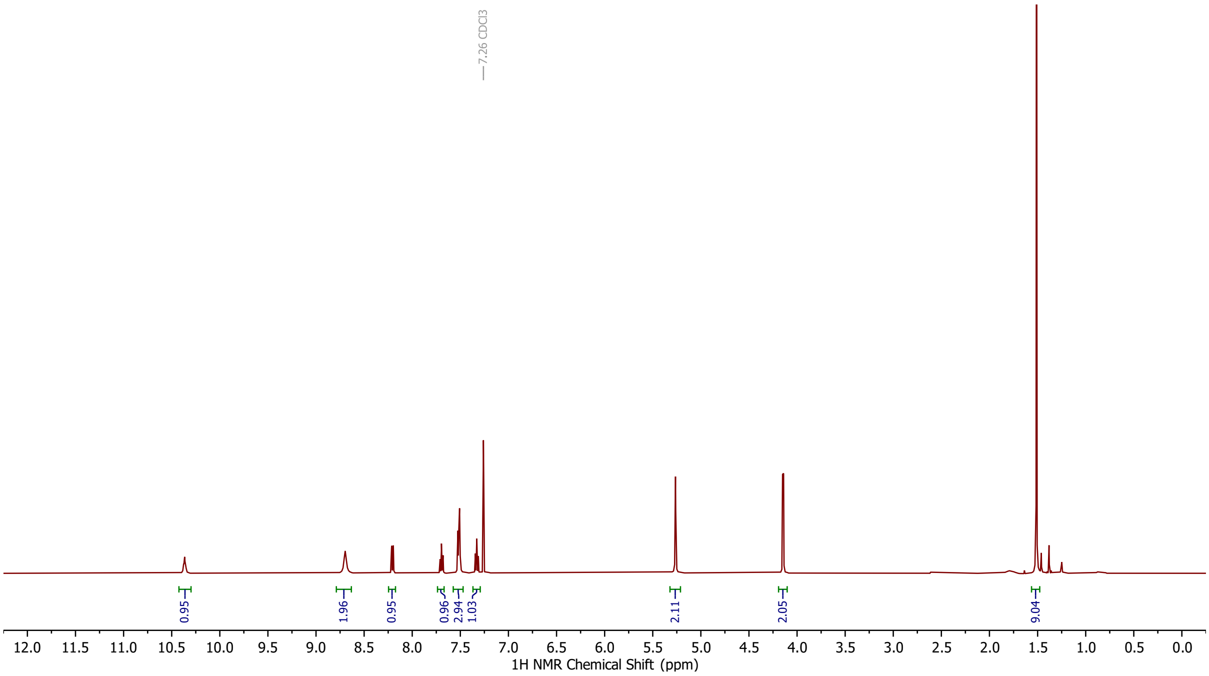

^{13}C NMR (126 MHz, 300 K, $CDCl_3$):

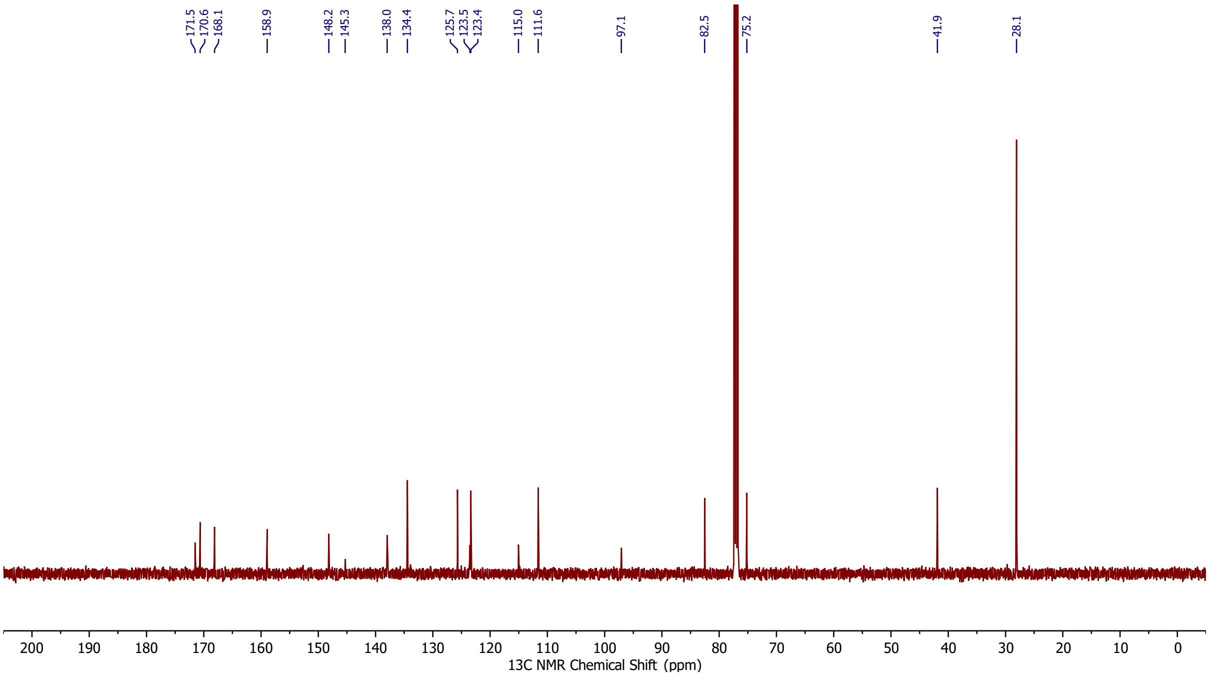

(4-Hydroxy-2-oxo-1-(pyridin-4-ylmethoxy)-1,2-dihydroquinoline-3-carbonyl)glycine (44)

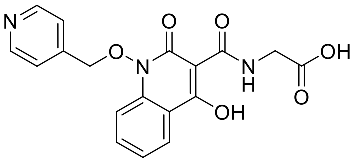

^{1}H NMR (500 MHz, 300 K, DMSO-d_6):

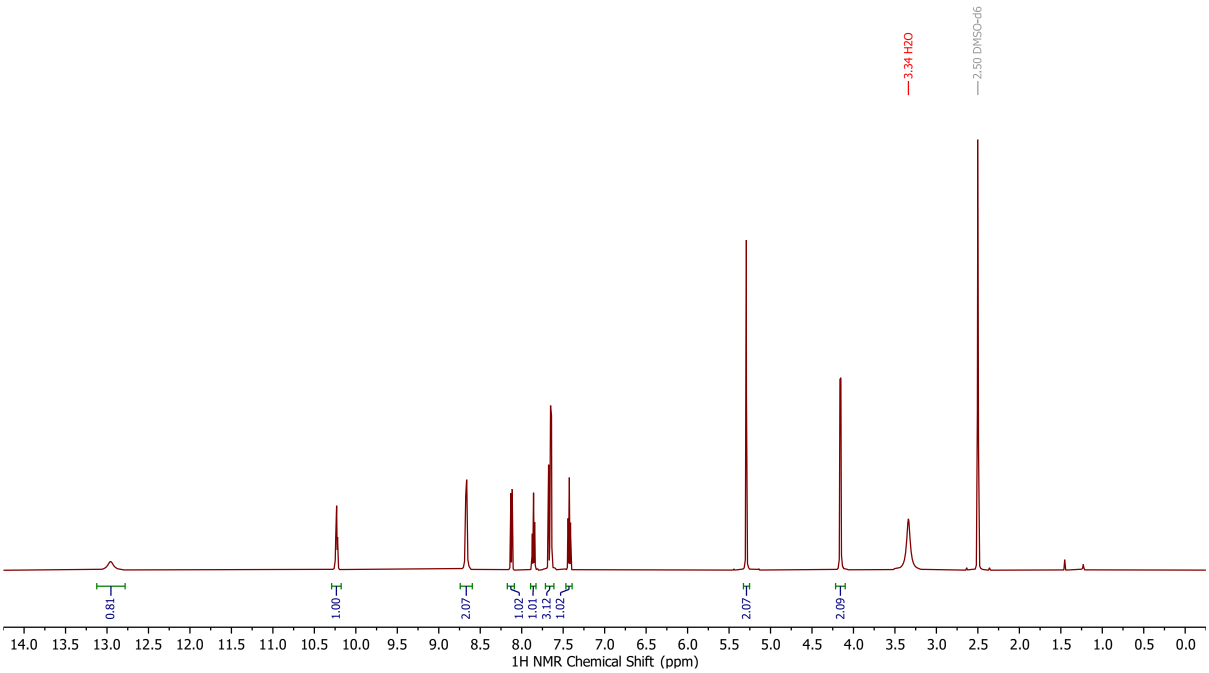

^{13}C NMR (126 MHz, 300 K, DMSO-d_6):

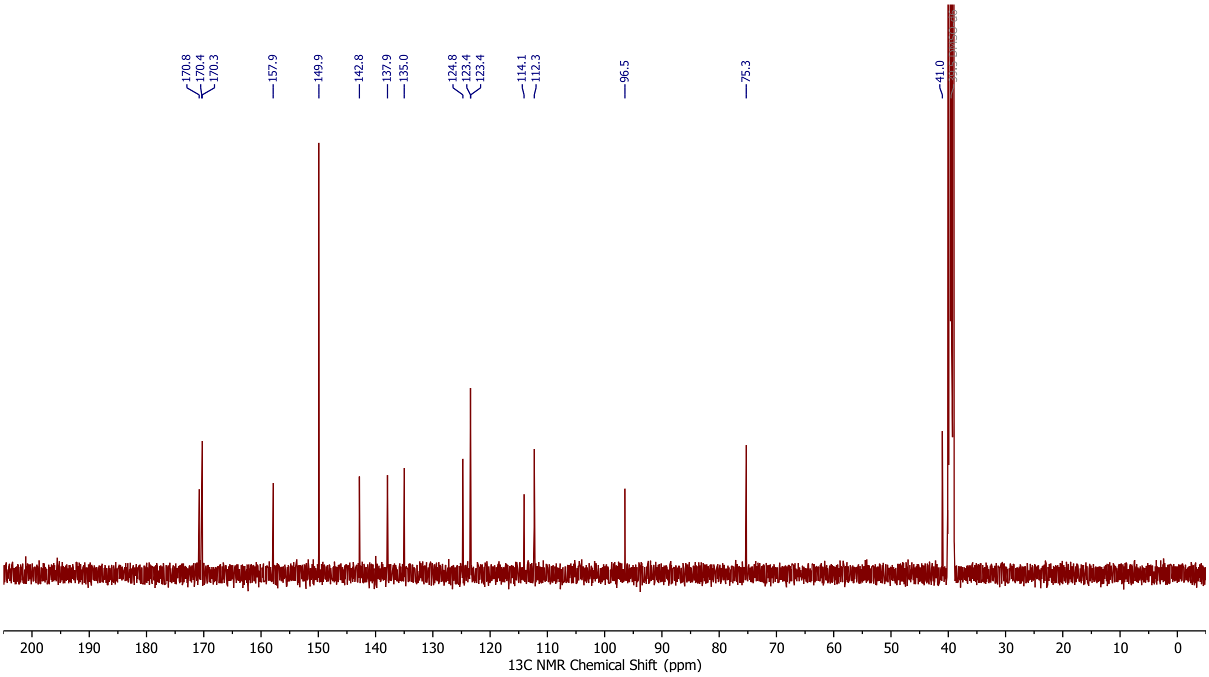

tert-Butyl (4-hydroxy-2-oxo-1-((tetrahydro-2H-pyran-4-yl)methoxy)-1,2-dihydroquinoline-3-carbonyl)glycinate (45a)

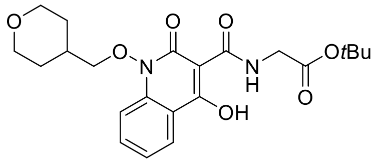

^{1}H NMR (600 MHz, 300 K, $CDCl_3$):

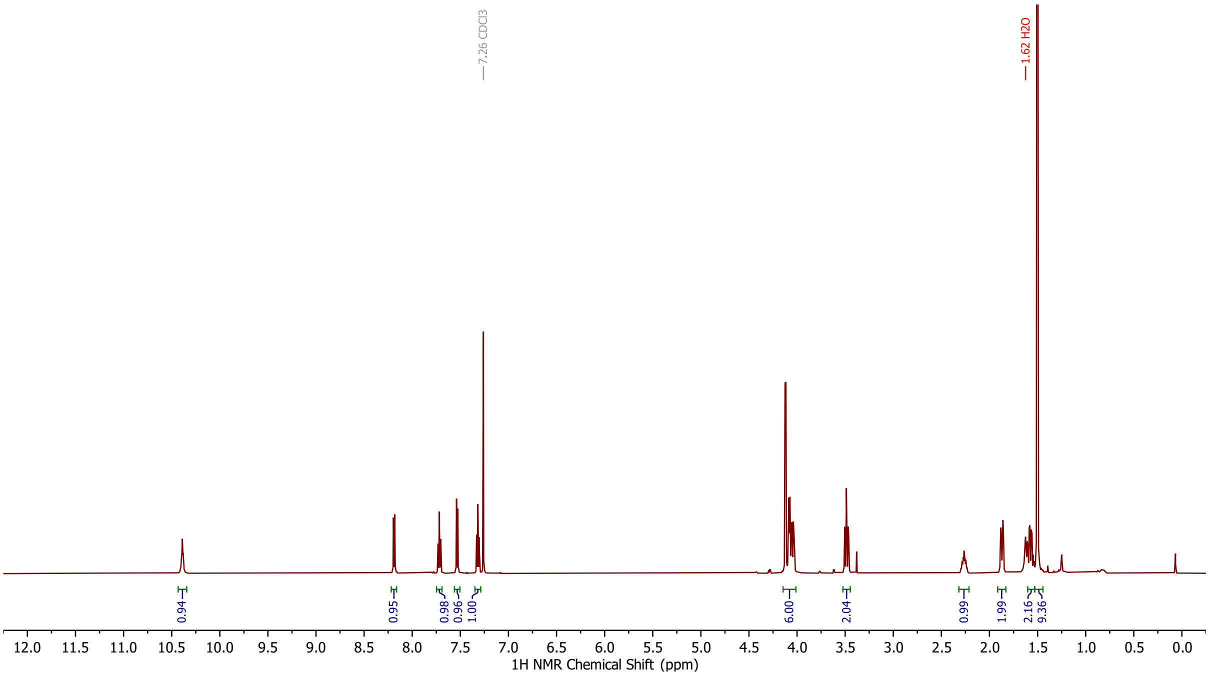

^{13}C NMR (151 MHz, 300 K, $CDCl_3$):

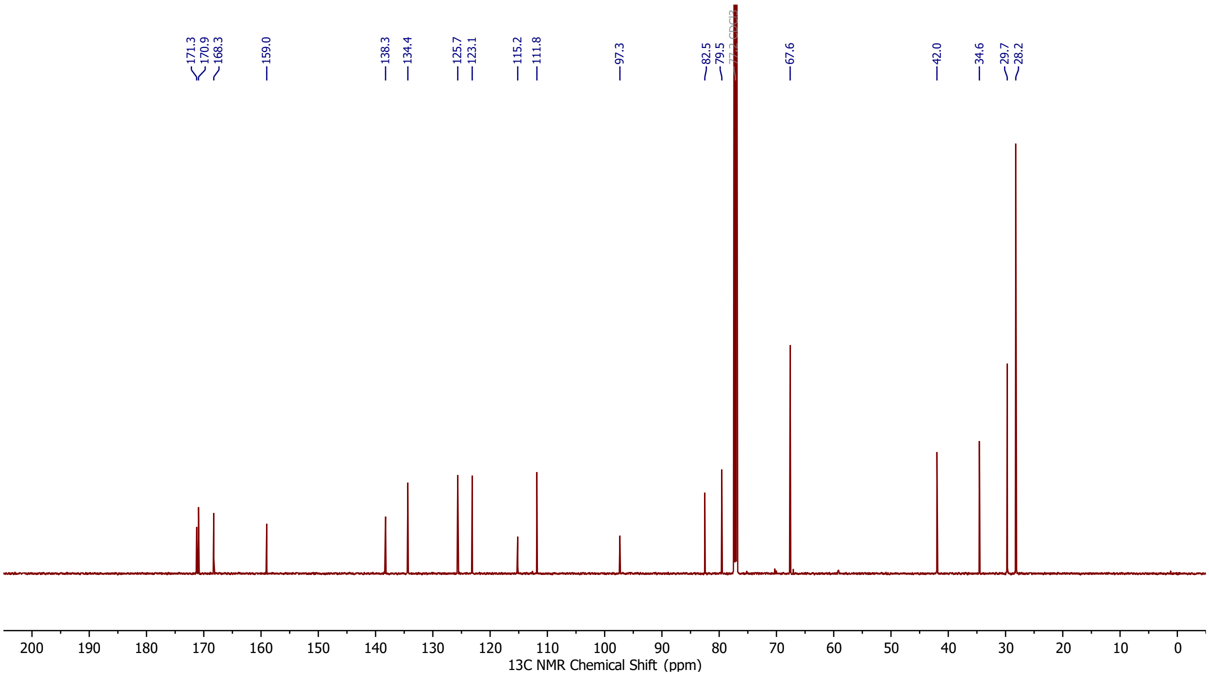

(4-Hydroxy-2-oxo-1-((tetrahydro-2*H*-pyran-4-yl)methoxy)-1,2-dihydroquinoline-3-carbonyl)glycine (45)

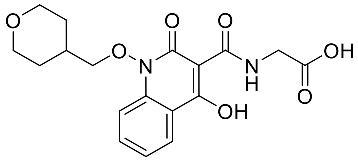

^{1}H NMR (600 MHz, 300 K, DMSO-d_6):

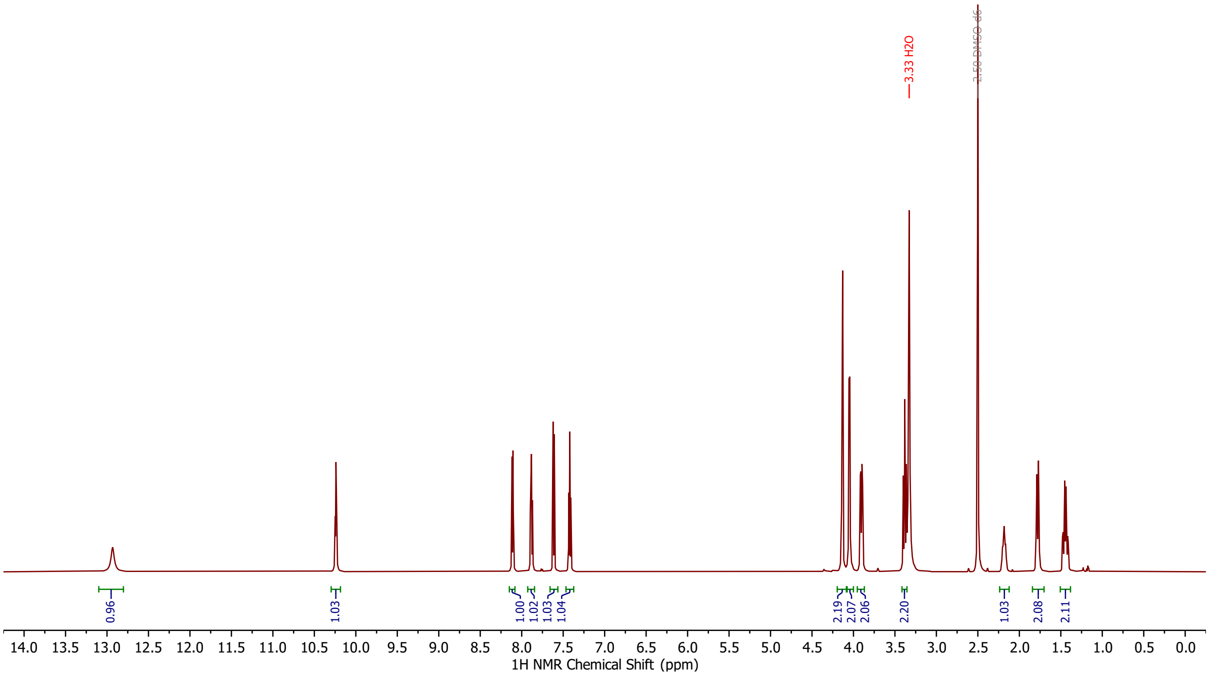

^{13}C NMR (151 MHz, 300 K, DMSO-d_6):

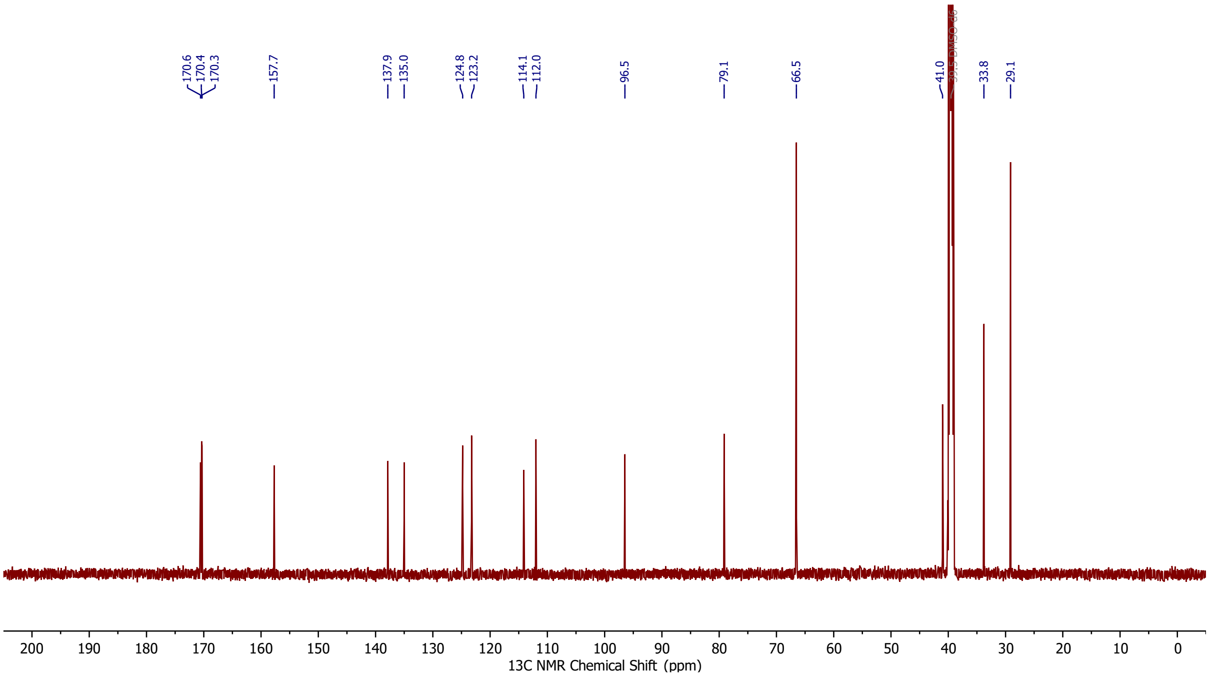

tert-Butyl (4-hydroxy-2-oxo-1-(2,2,2-trifluoroethoxy)-1,2-dihydroquinoline-3-carbonyl)glycinate (46a)

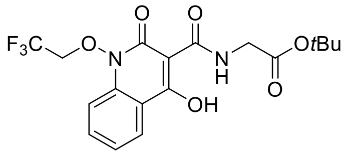

^{1}H NMR (600 MHz, 300 K, DMSO-d_6):

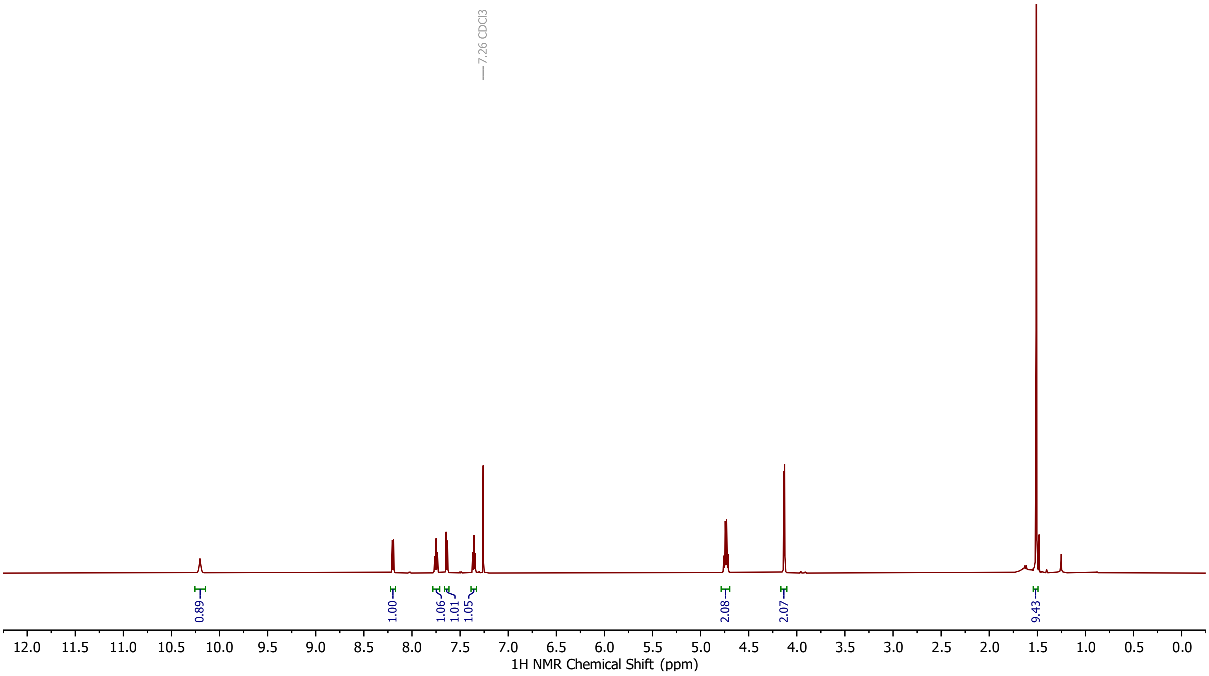

^{13}C NMR (151 MHz, 300 K, DMSO-d_6):

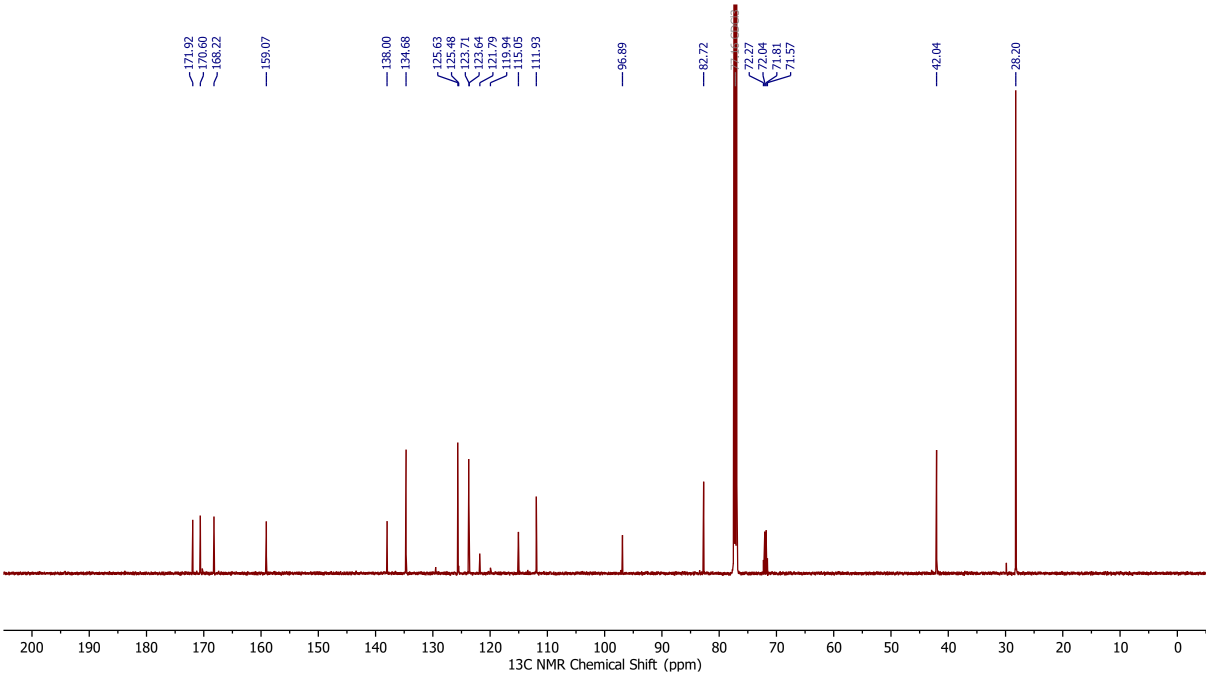

***tert*-Butyl (1-(cyclopentylmethoxy)-4-hydroxy-2-oxo-1,2-dihydroquinoline-3-carbonyl)glycinate (48a)**

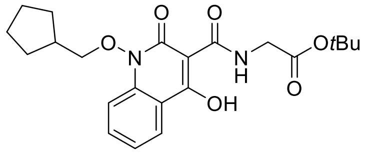

^{1}H NMR (500 MHz, 300 K, $CDCl_3$):

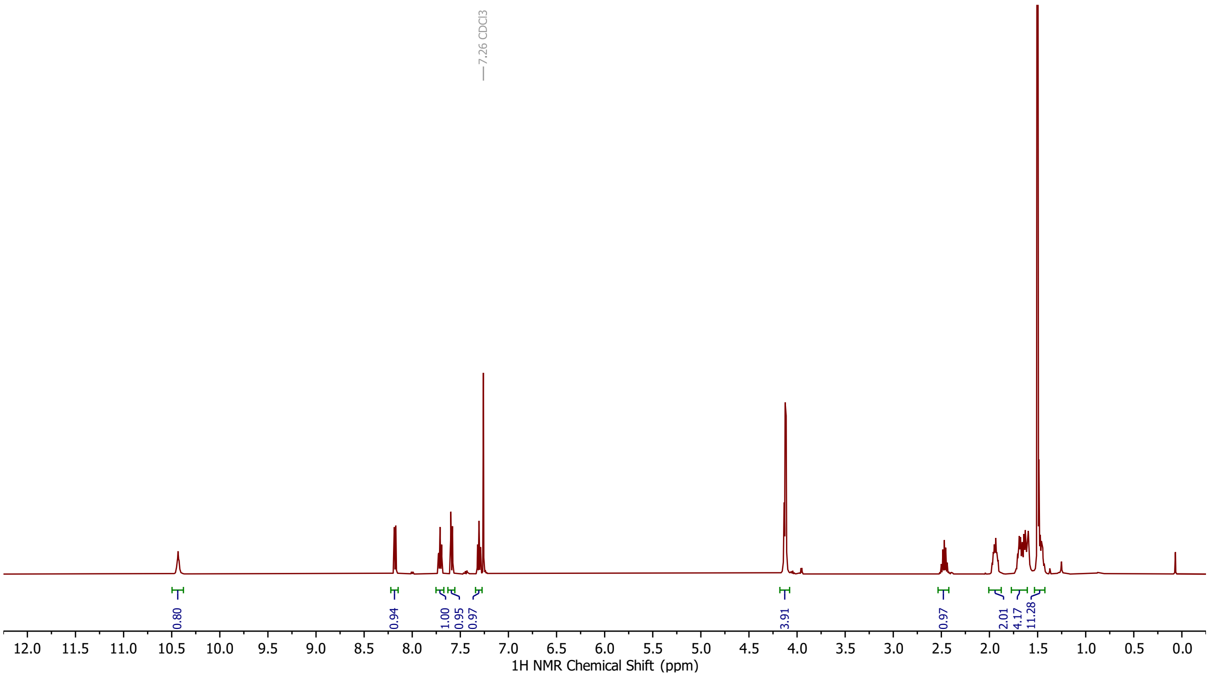

^{13}C NMR (126 MHz, 300 K, $CDCl_3$):

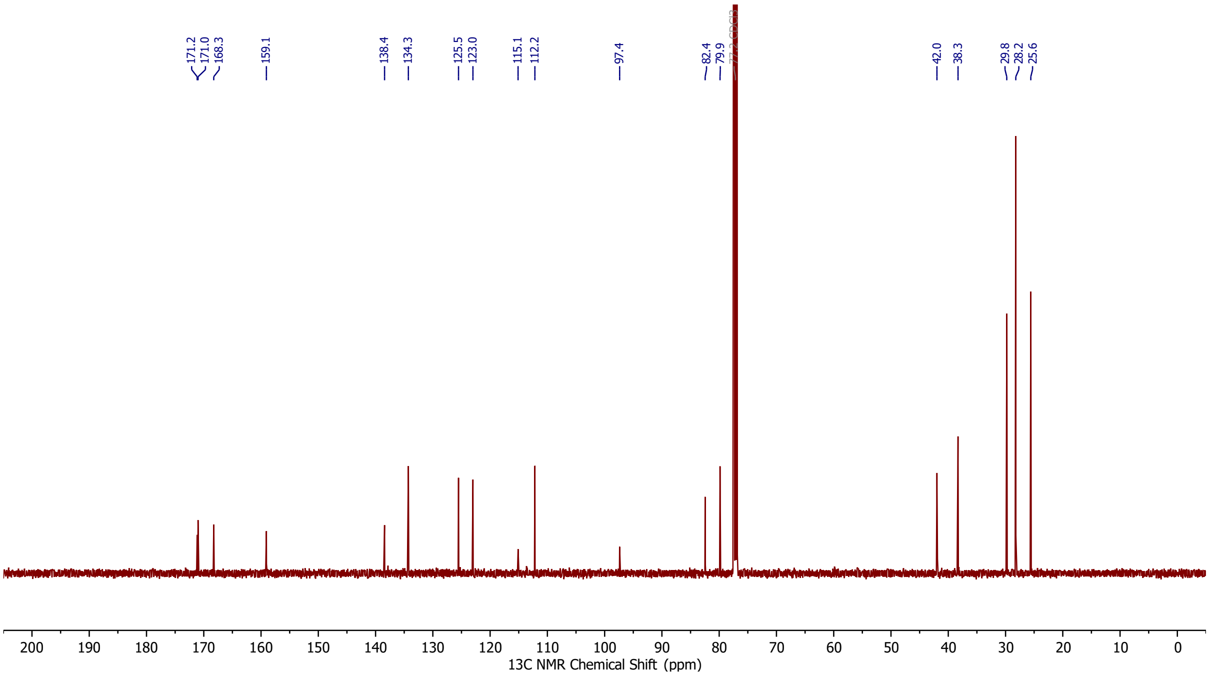

***tert*-Butyl (1-(cyclohexylmethoxy)-4-hydroxy-2-oxo-1,2-dihydroquinoline-3-carbonyl)glycinate (49a)**

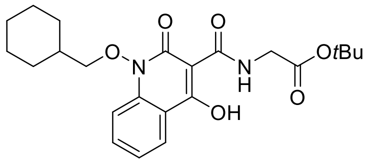

^{1}H NMR (600 MHz, 300 K, $CDCl_3$):

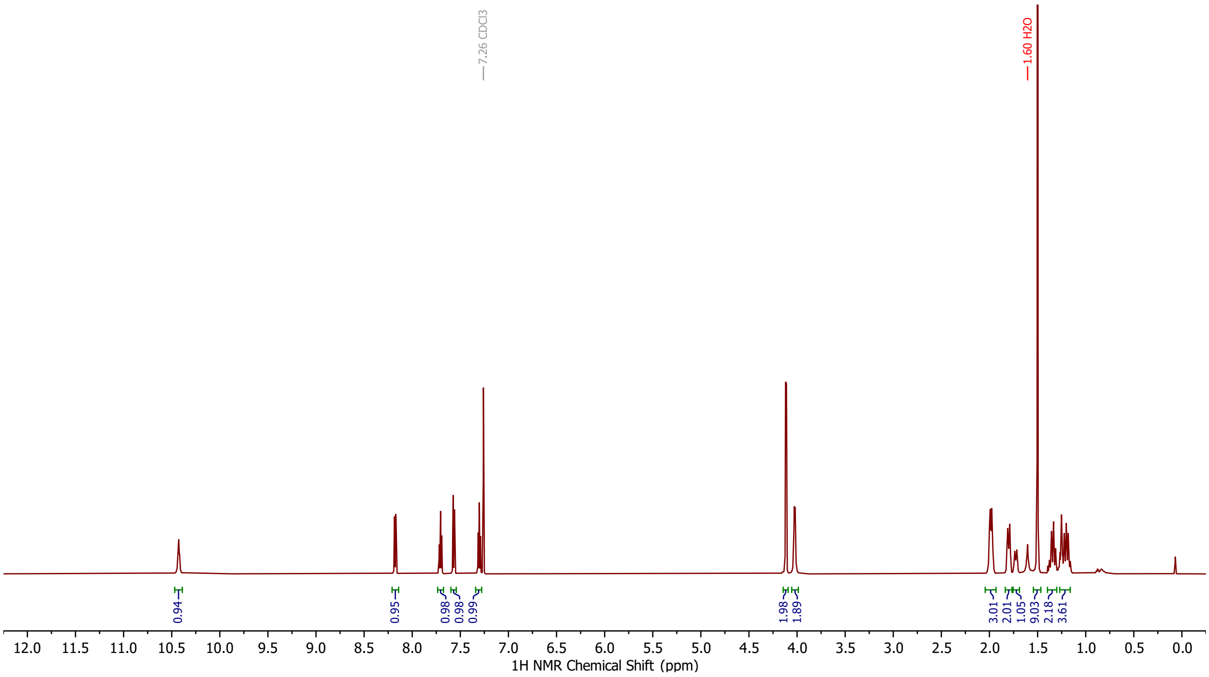

^{13}C NMR (151 MHz, 300 K, $CDCl_3$):

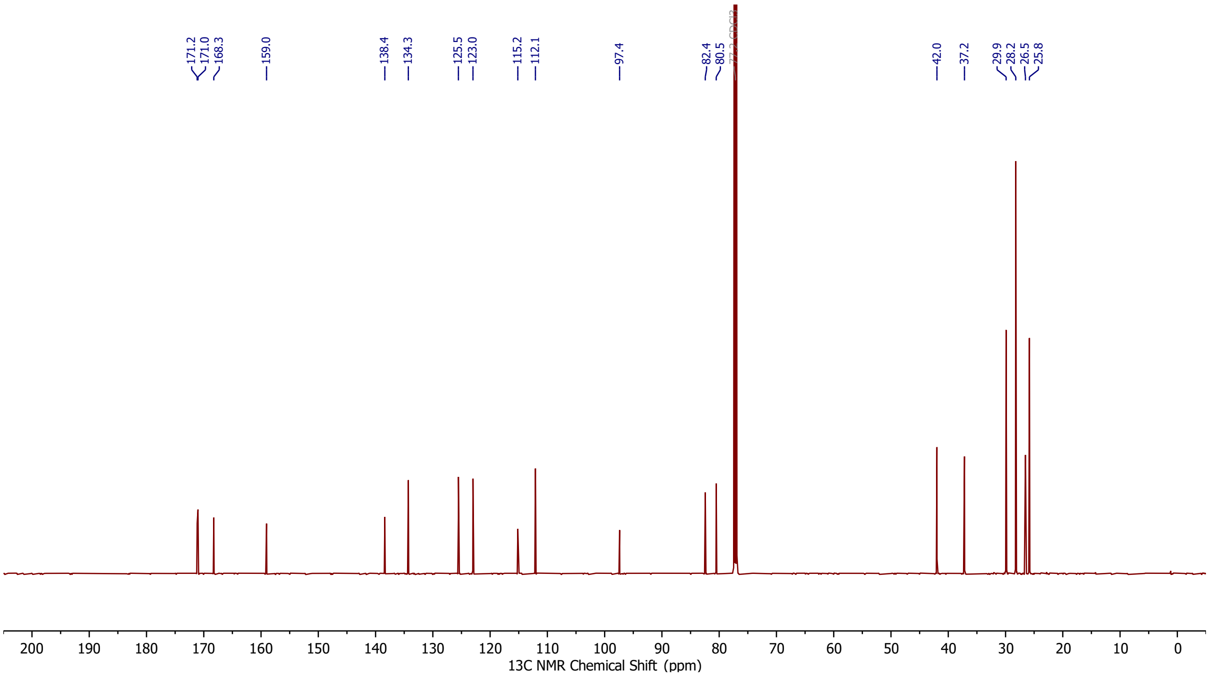

***tert*-Butyl (4-hydroxy-1-(naphthalen-1-ylmethoxy)-2-oxo-1,2-dihydroquinoline-3-carbonyl)-glycinate (50a)**

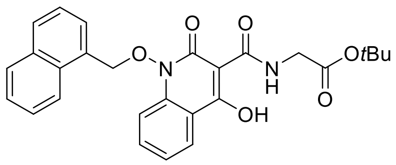

^{1}H NMR (600 MHz, 300 K, $CDCl_3$):

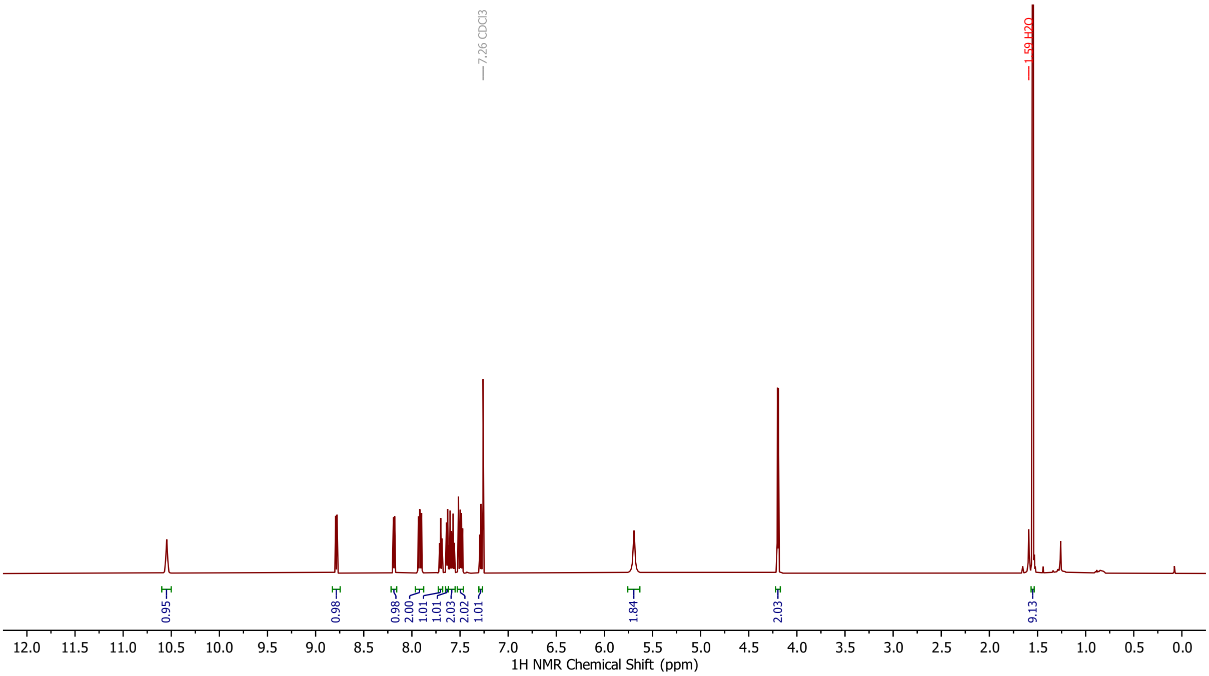

^{13}C NMR (151 MHz, 300 K, $CDCl_3$):

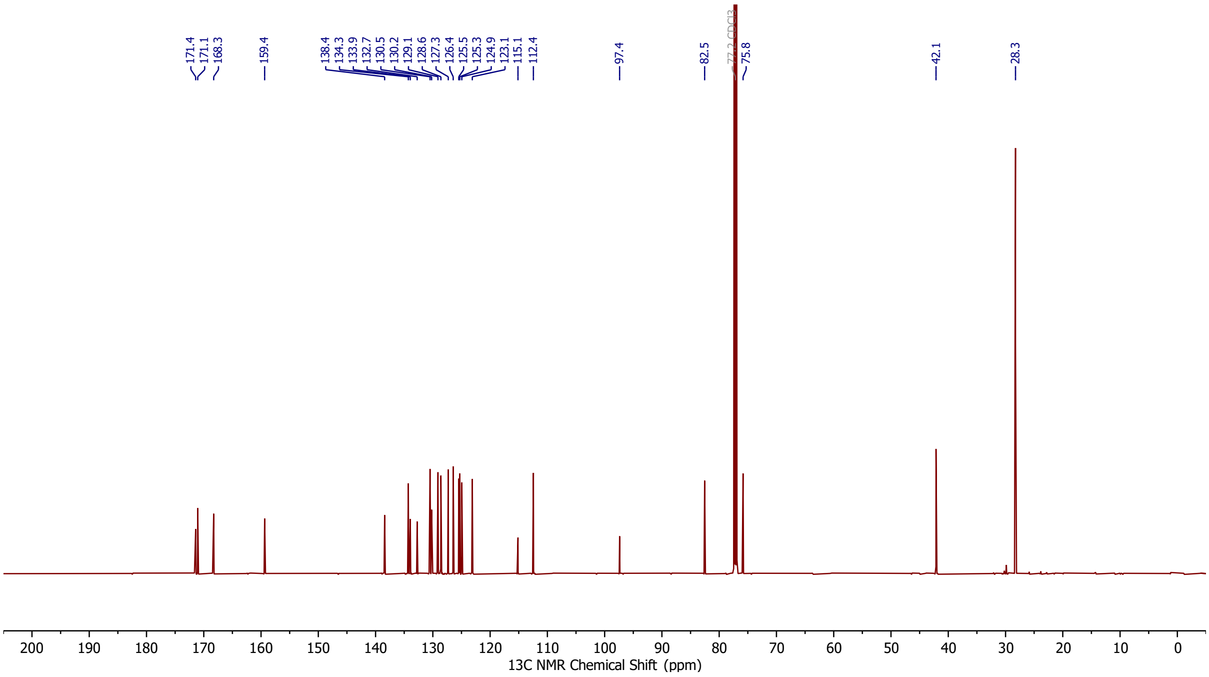

(4-Hydroxy-1-(naphthalen-1-ylmethoxy)-2-oxo-1,2-dihydroquinoline-3-carbonyl)glycine (50)

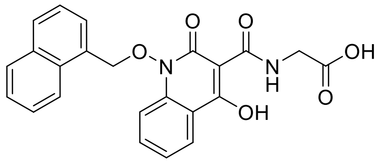

^{1}H NMR (600 MHz, 300 K, DMSO-d_6):

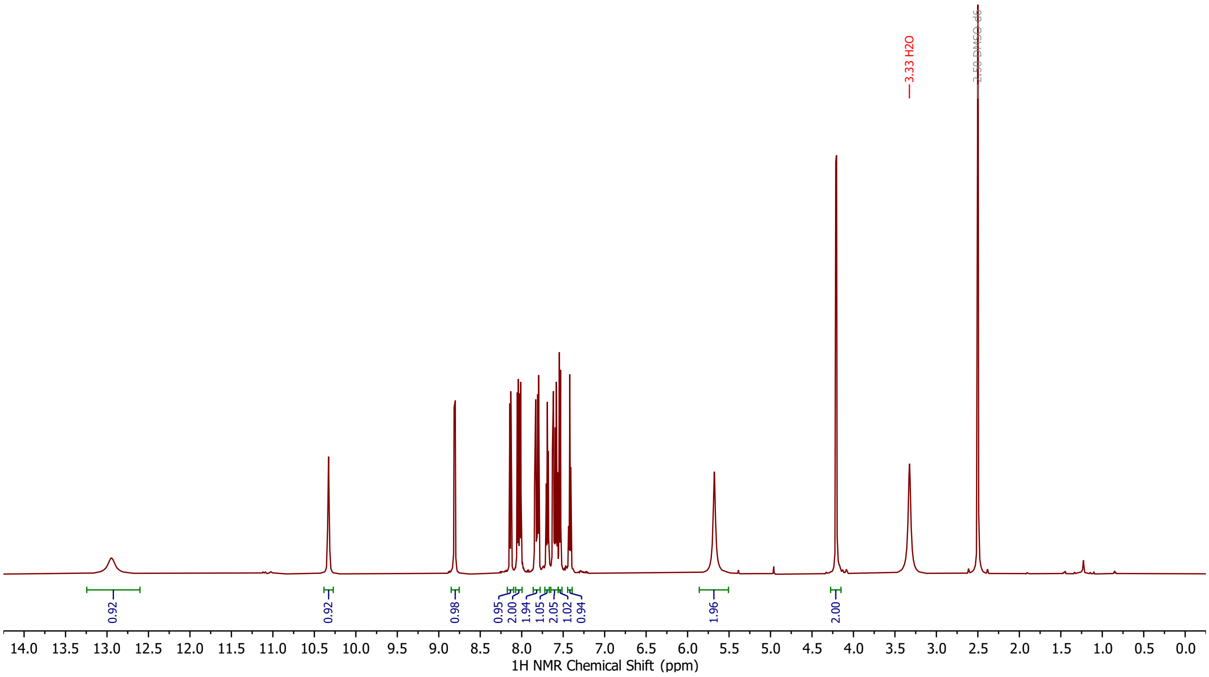

^{13}C NMR (151 MHz, 300 K, DMSO-d_6):

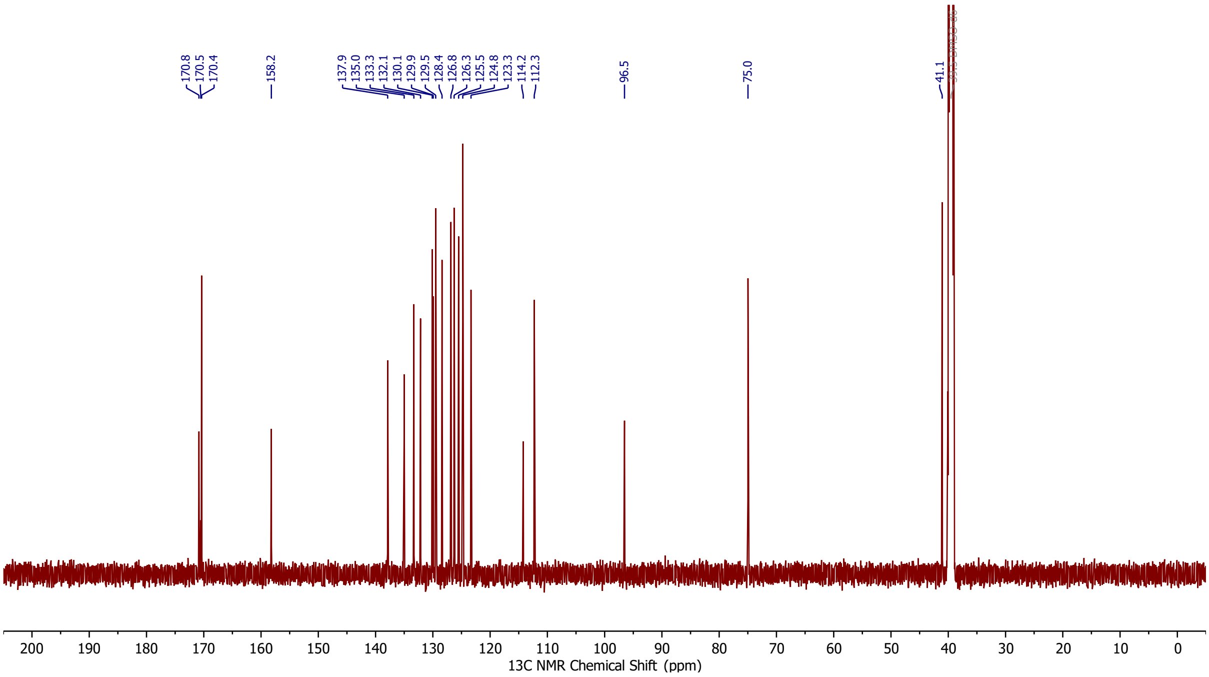

tert-Butyl (4-hydroxy-1-(neopentyloxy)-2-oxo-1,2-dihydroquinoline-3-carbonyl)glycinate (51a)

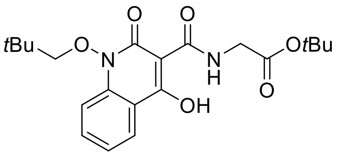

^{1}H NMR (600 MHz, 300 K, $CDCl_3$):

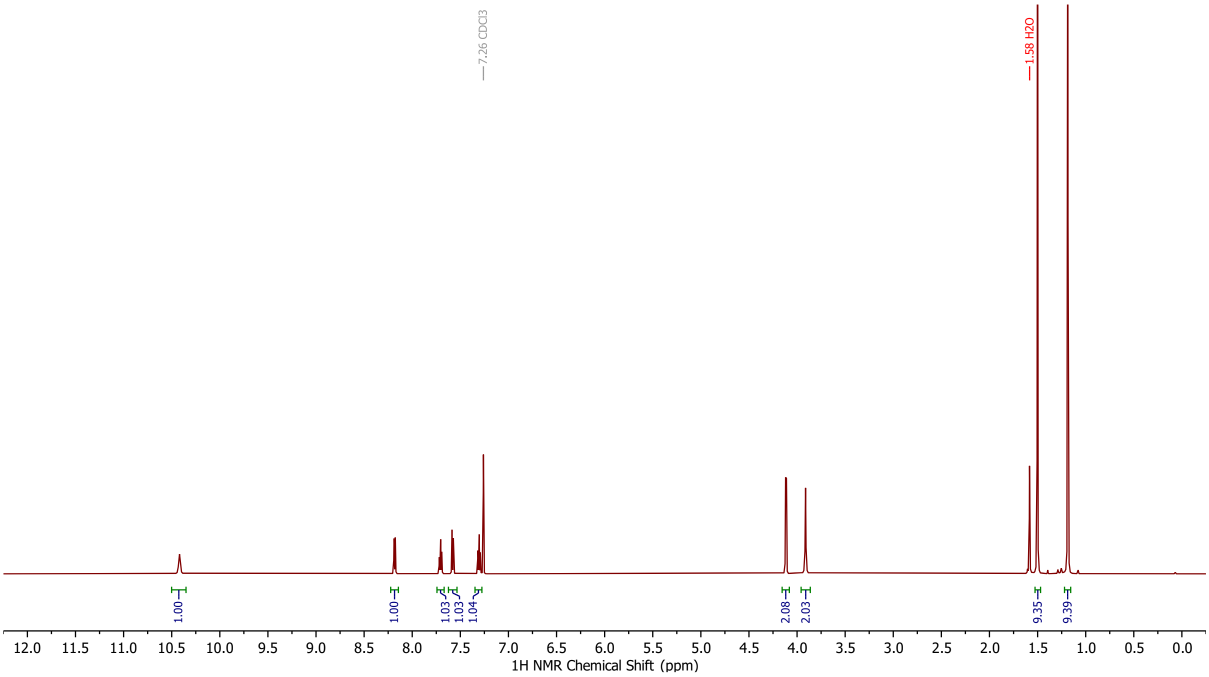

^{13}C NMR (151 MHz, 300 K, $CDCl_3$):

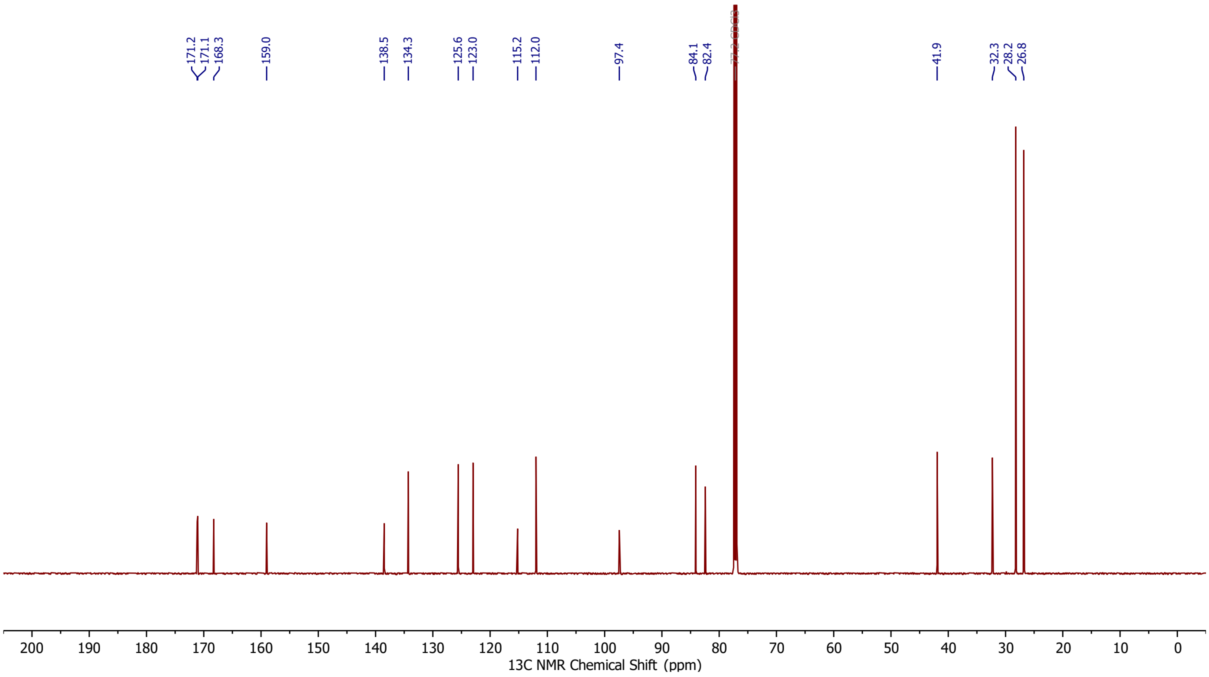

(4-Hydroxy-1-(neopentyloxy)-2-oxo-1,2-dihydroquinoline-3-carbonyl)glycine (51)

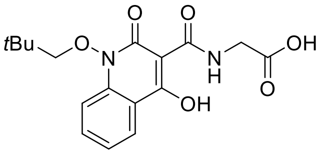

^{1}H NMR (600 MHz, 300 K, DMSO-d_6):

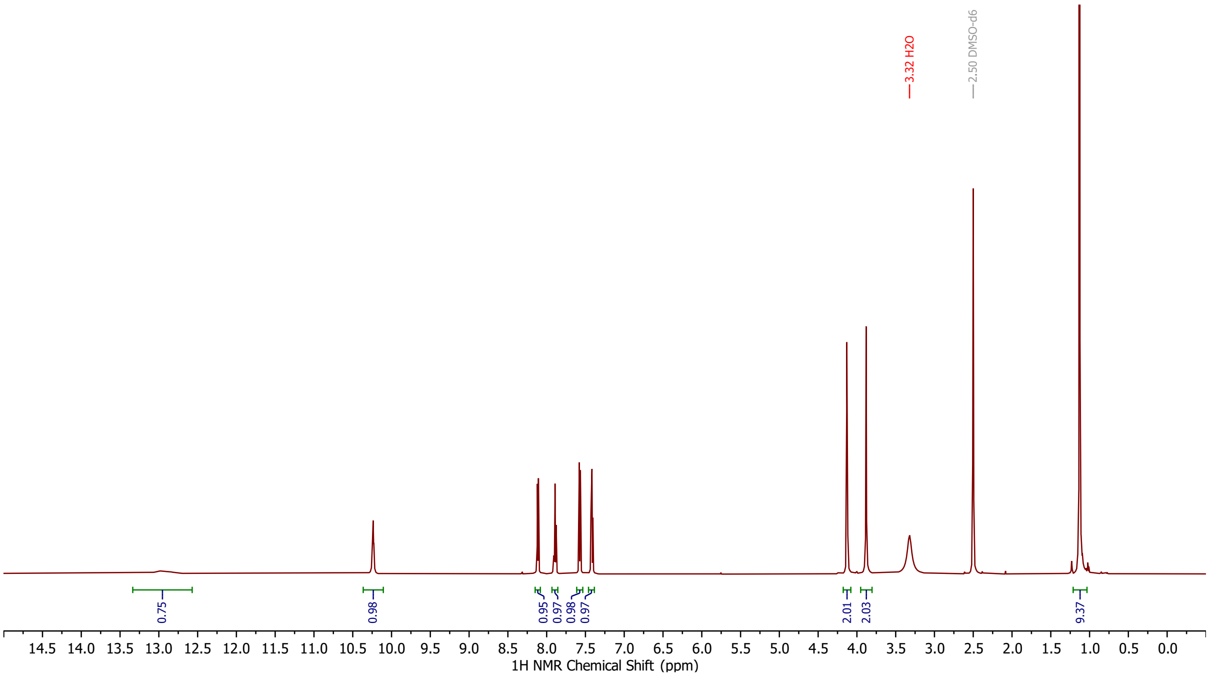

^{13}C NMR (151 MHz, 300 K, DMSO-d_6):

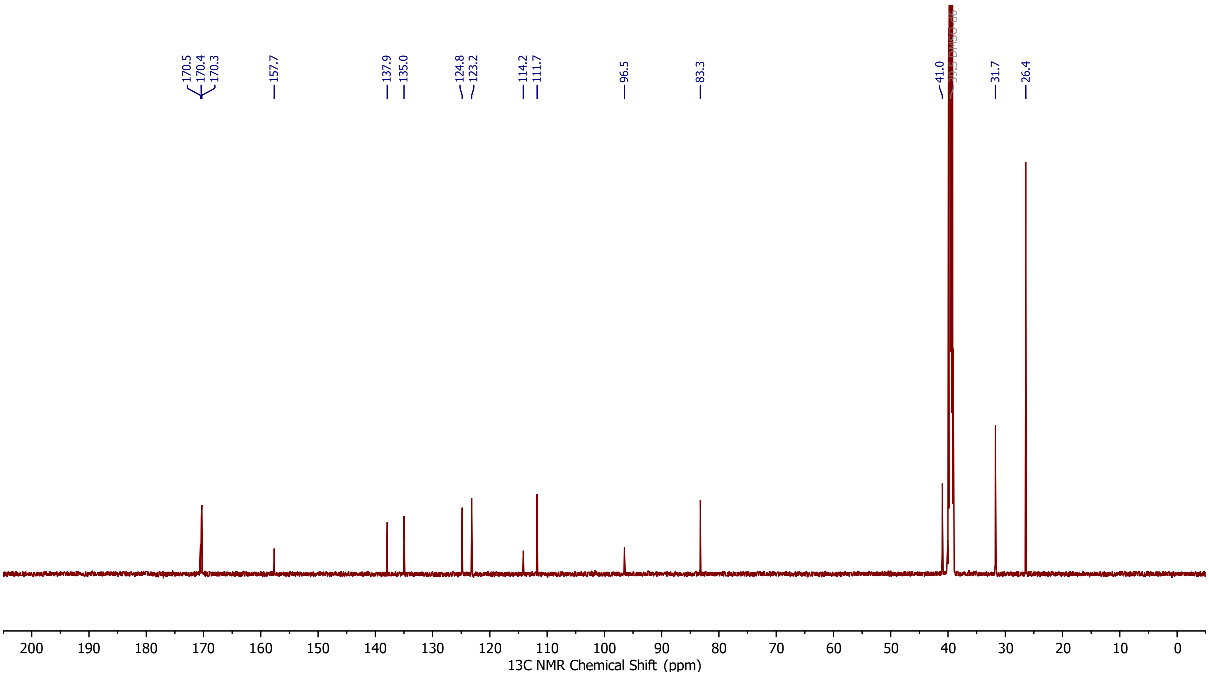

tert-Butyl (1-(cyclopropylmethyl)-4-hydroxy-2-oxo-1,2-dihydroquinoline-3-carbonyl)glycinate (52d)

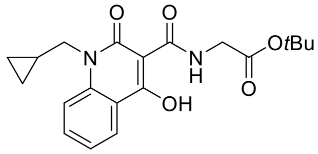

^{1}H NMR (600 MHz, 300 K, $CDCl_3$):

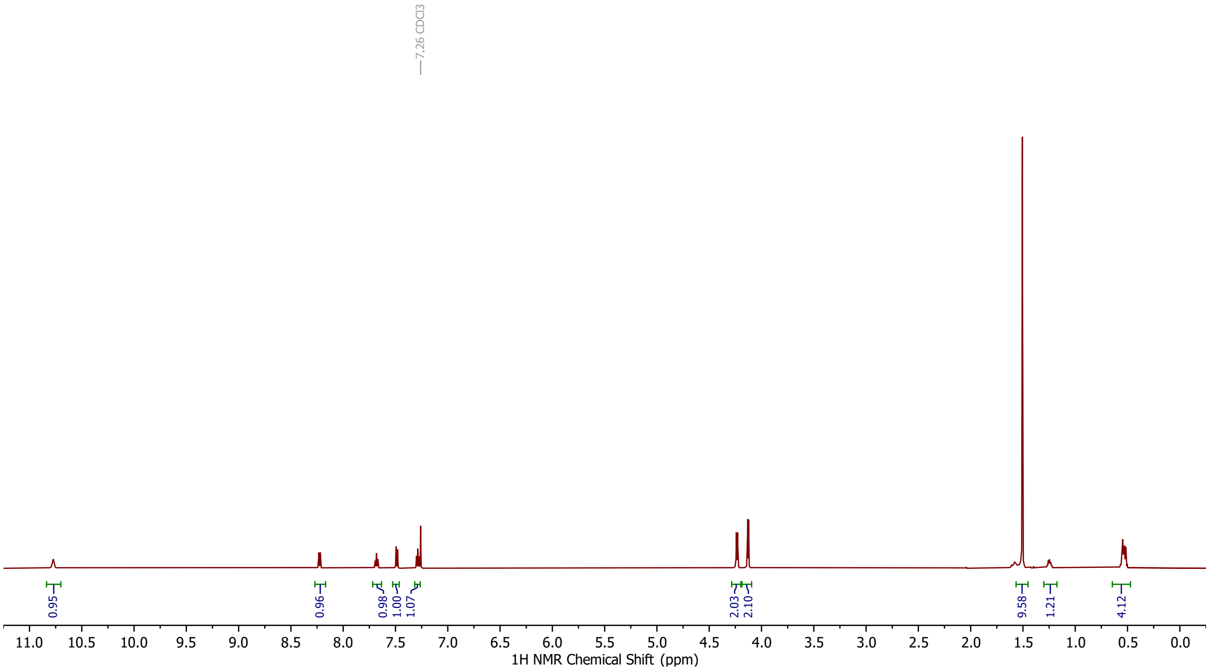

^{13}C NMR (151 MHz, 300 K, $CDCl_3$):

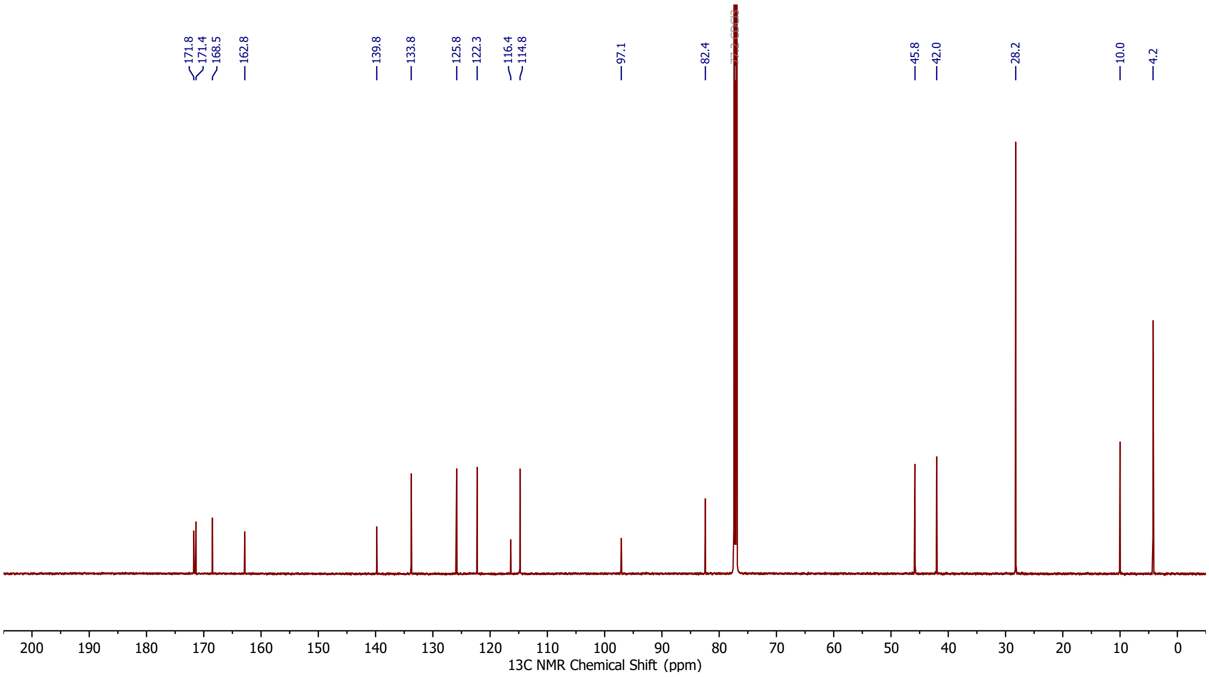

1-(2-Cyclopropylethyl)-2*H*-benzo[*d*][1,3]oxazine-2,4(1*H*)-dione (53a)

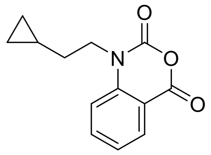

^{1}H NMR (600 MHz, 300 K, $CDCl_3$):

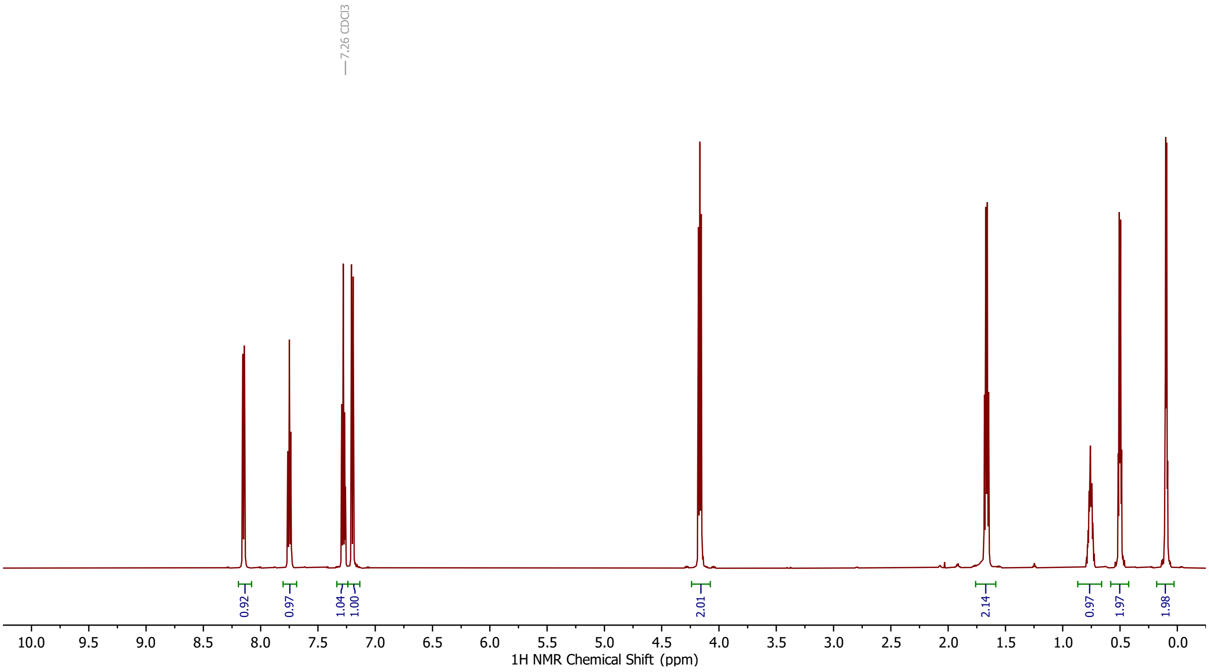

^{13}C NMR (151 MHz, 300 K, $CDCl_3$):

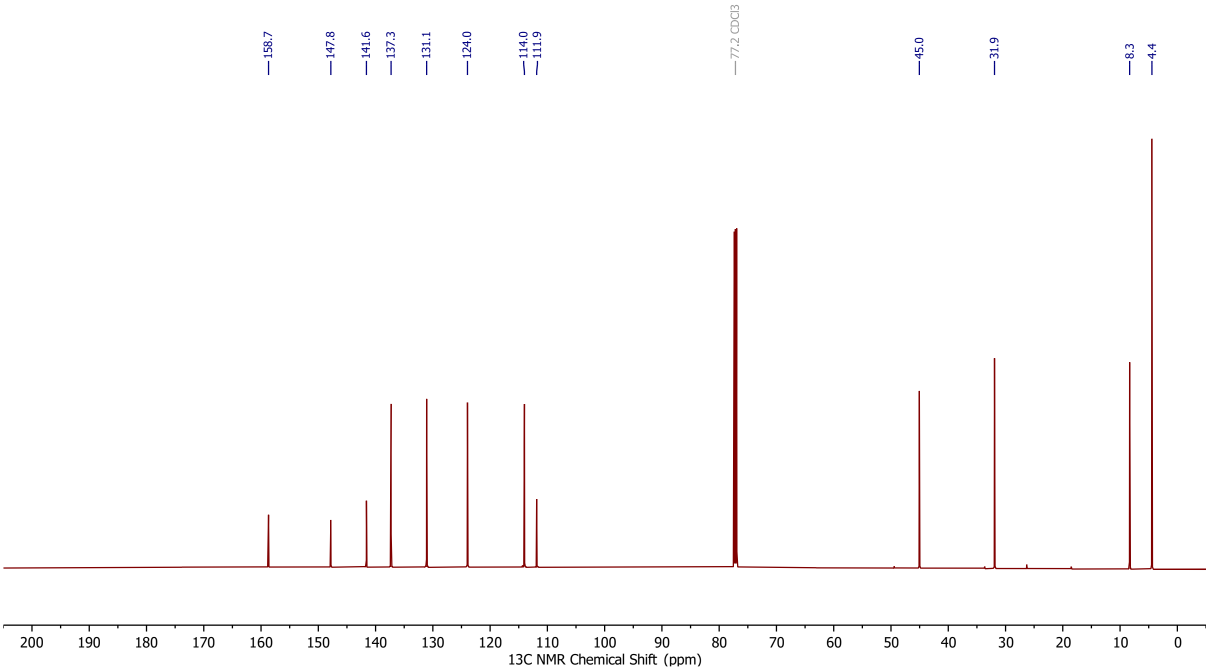

Ethyl 1-(2-cyclopropylethyl)-4-hydroxy-2-oxo-1,2-dihydroquinoline-3-carboxylate (53b)

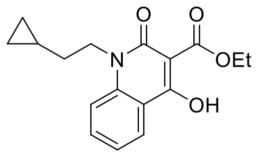

^{1}H NMR (600 MHz, 300 K, $CDCl_3$):

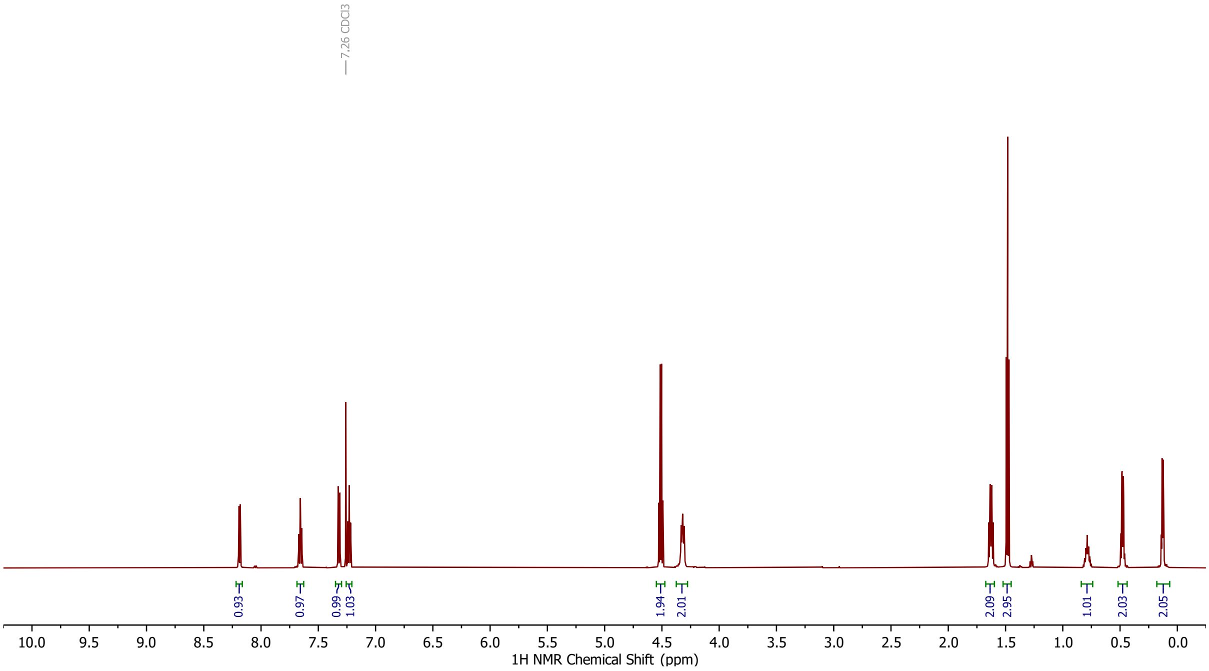

^{13}C NMR (151 MHz, 300 K, $CDCl_3$):

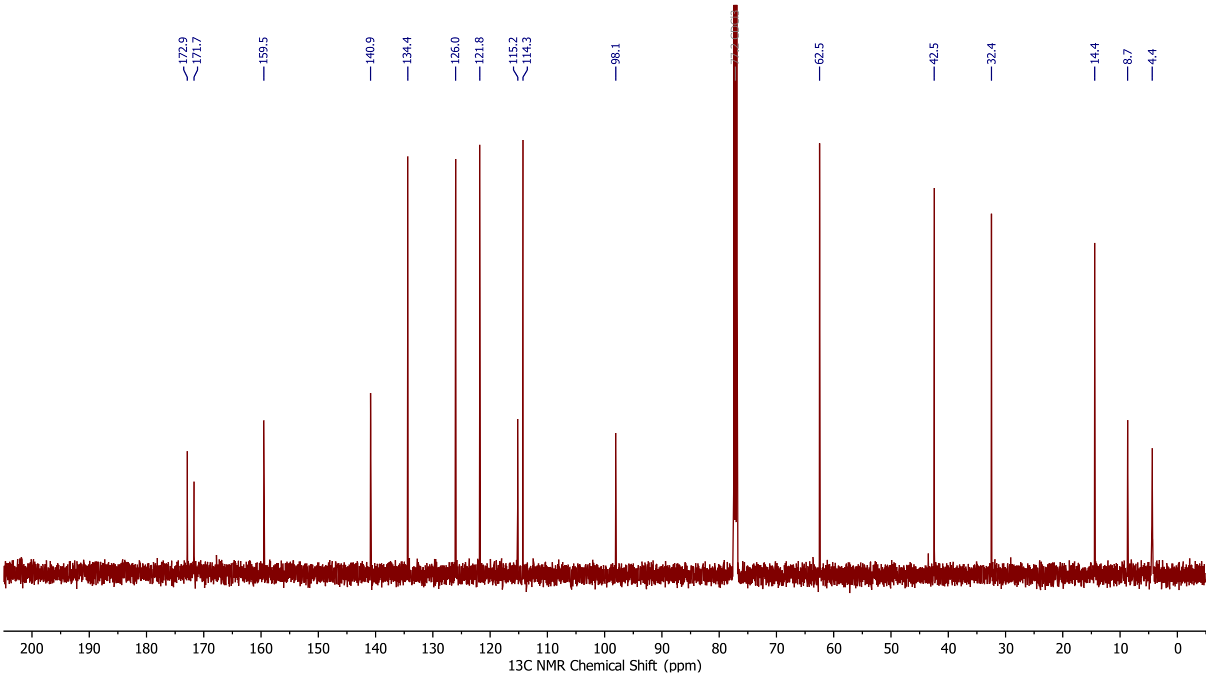

tert-Butyl (1-(2-cyclopropylethyl)-4-hydroxy-2-oxo-1,2-dihydroquinoline-3-carbonyl)glycinate (53c)

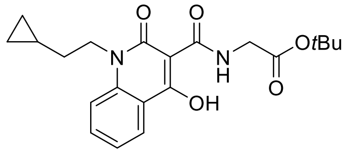

^{1}H NMR (600 MHz, 300 K, $CDCl_3$):

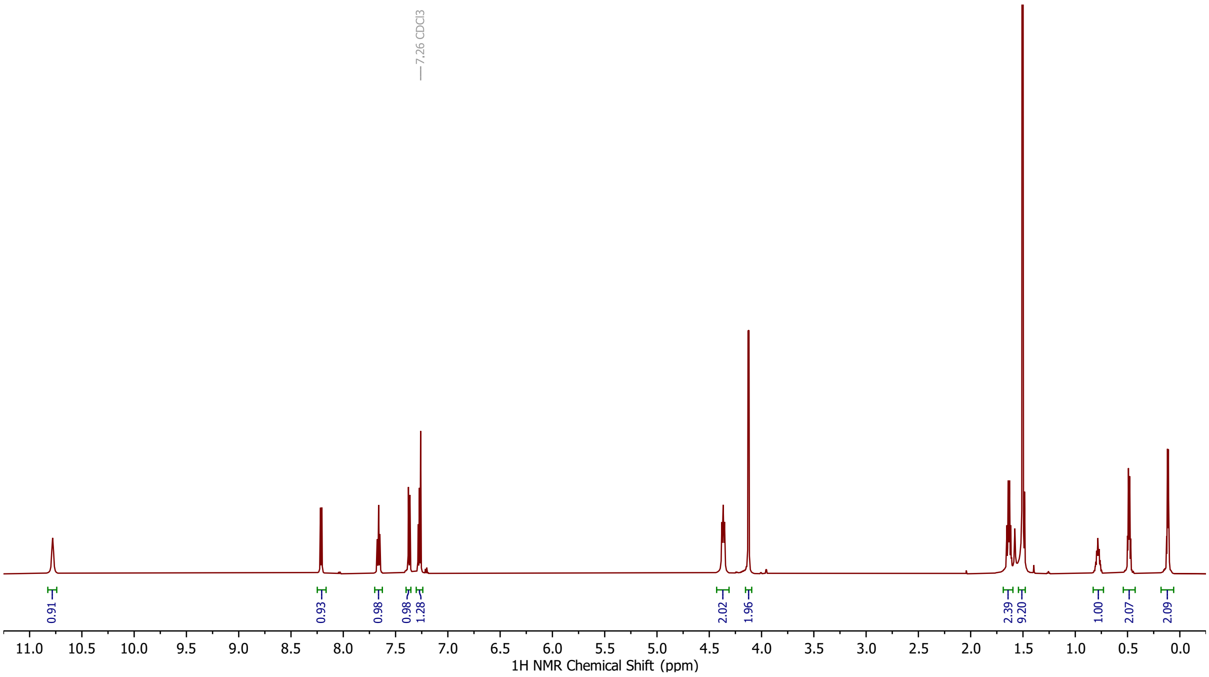

^{13}C NMR (151 MHz, 300 K, $CDCl_3$):

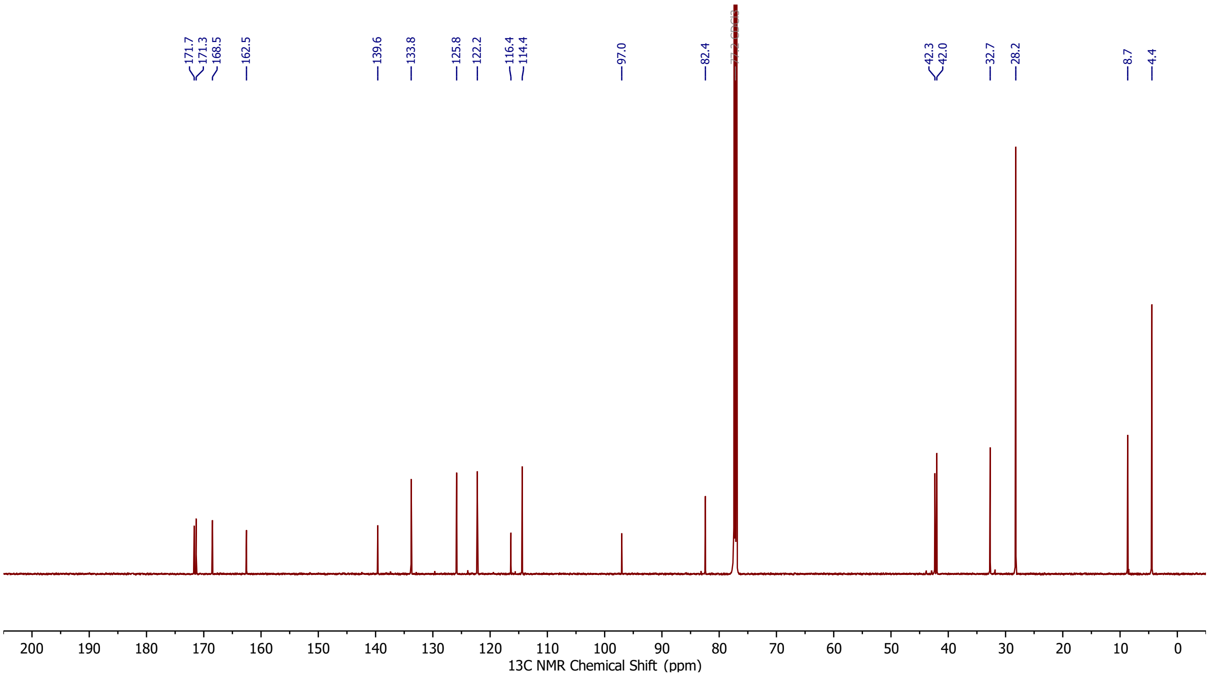

Ethyl 3-((*tert*-butoxycarbonyl)(cyclopropylmethoxy)amino)propanoate (54a)

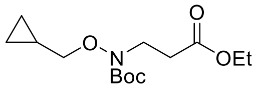

^{1}H NMR (500 MHz, 300 K, $CDCl_3$):

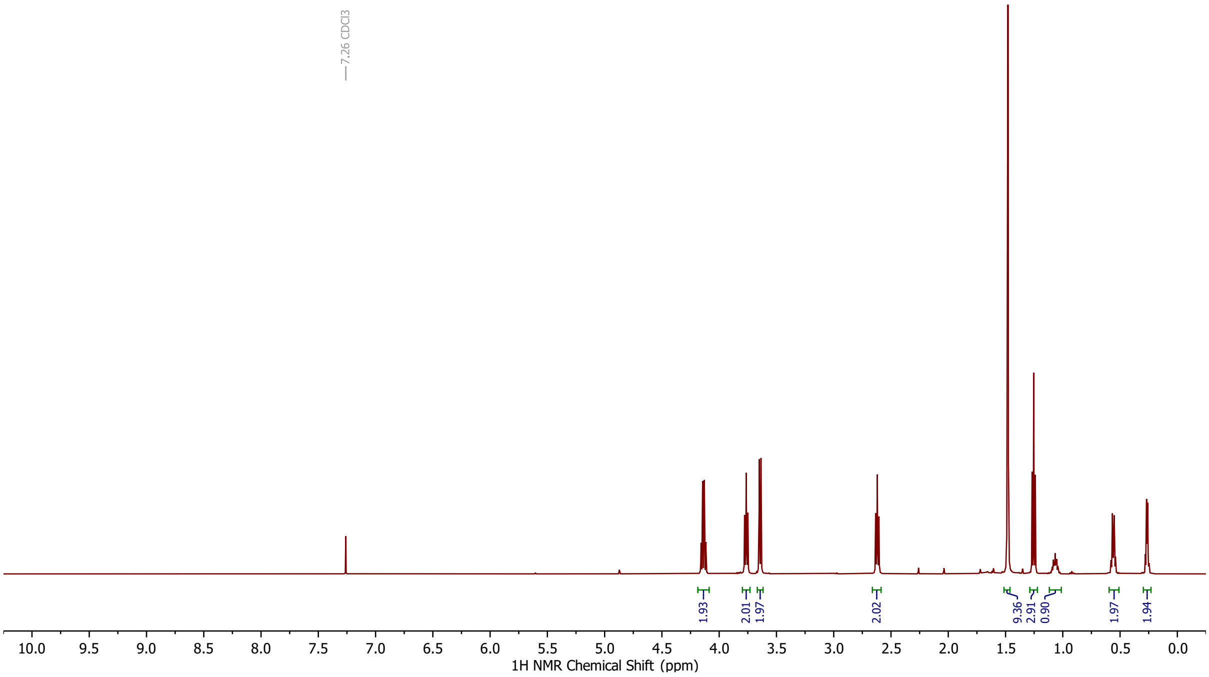

^{13}C NMR (126 MHz, 300 K, $CDCl_3$):

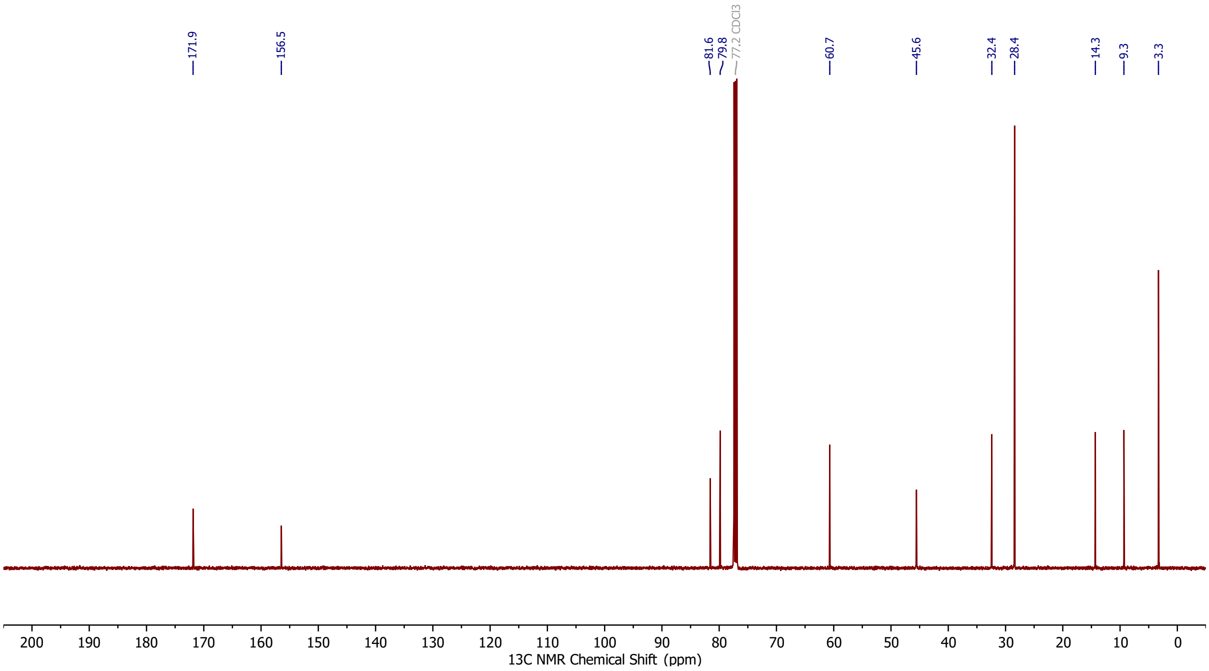

Ethyl 3-((cyclopropylmethoxy)(3-ethoxy-3-oxopropyl)amino)-3-oxopropanoate (54c)

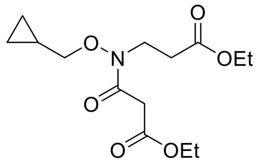

^{1}H NMR (600 MHz, 300 K, $CDCl_3$):

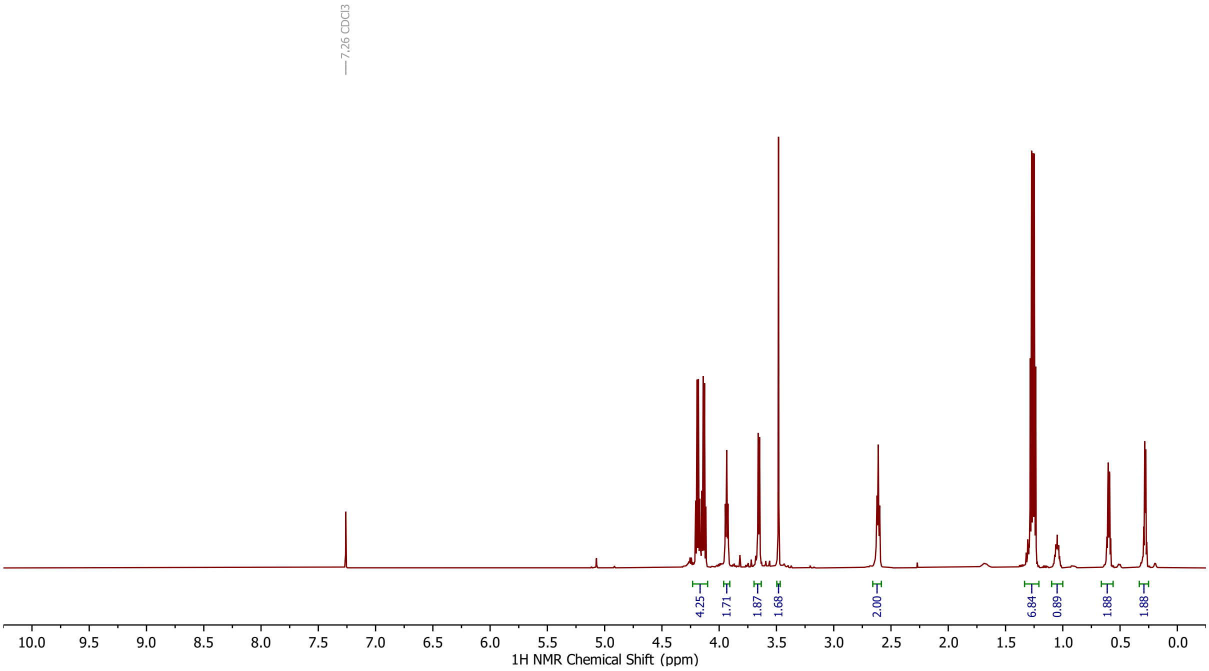

^{13}C NMR (151 MHz, 300 K, $CDCl_3$):

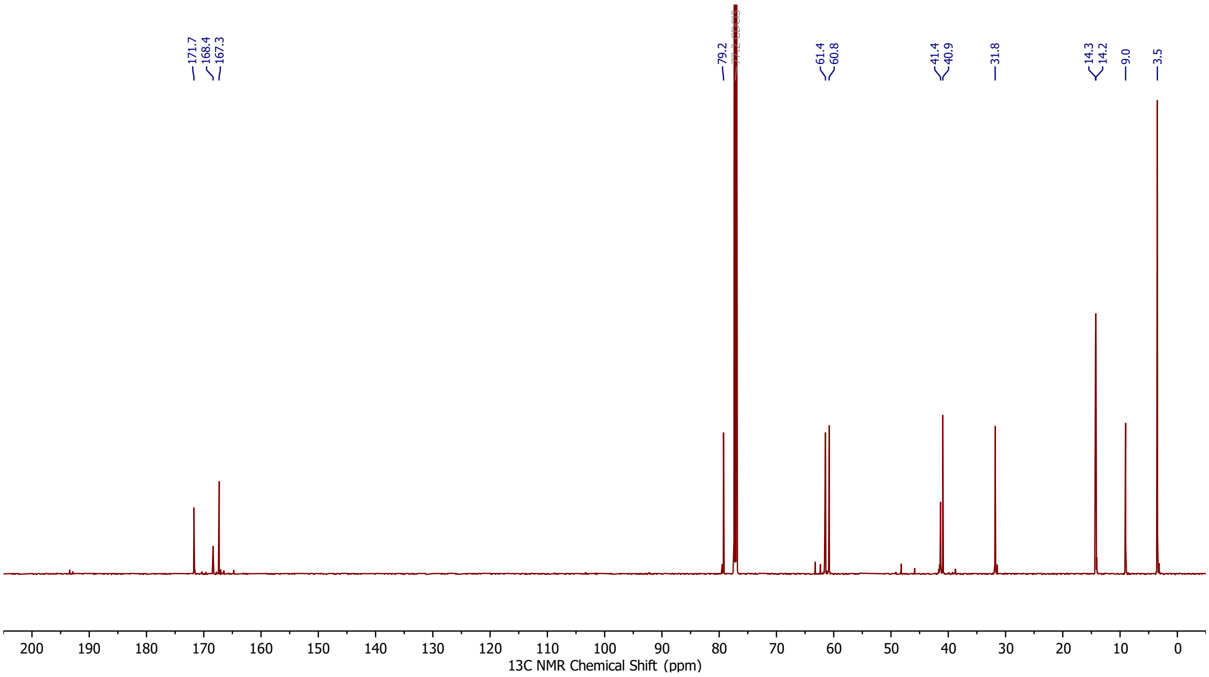

Methyl (1-(cyclopropylmethoxy)-4-hydroxy-2-oxo-1,2,5,6-tetrahydropyridine-3-carbonyl)glycinate (54e)

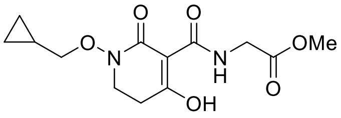

^{1}H NMR (600 MHz, 300 K, $CDCl_3$):

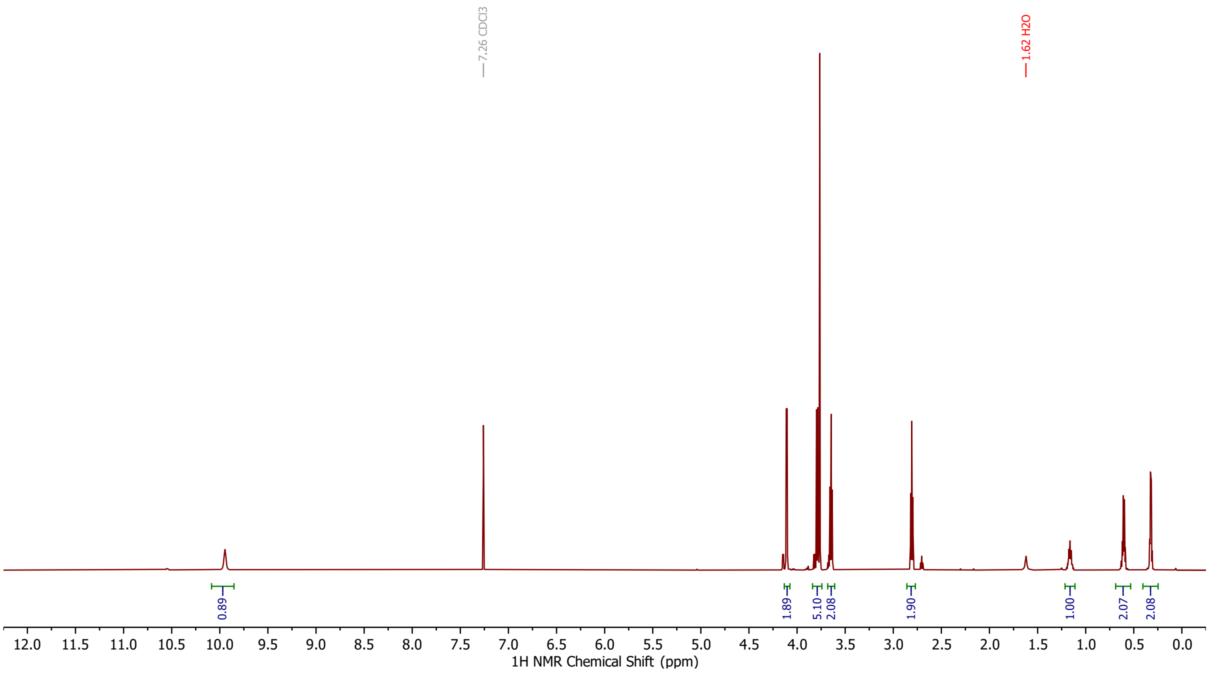

^{13}C NMR (151 MHz, 300 K, $CDCl_3$):

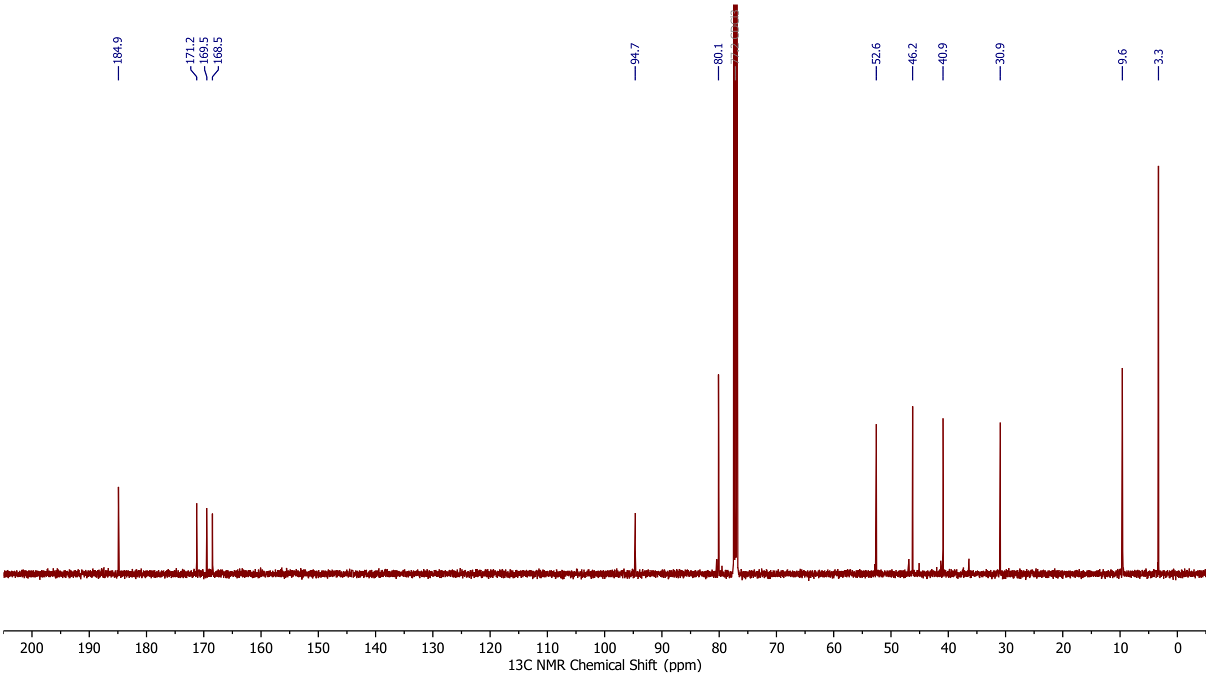

(1-(Cyclopropylmethoxy)-4-hydroxy-2-oxo-1,2,5,6-tetrahydropyridine-3-carbonyl)glycine (54)

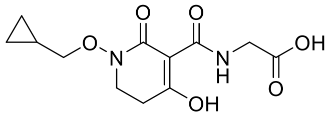

^{1}H NMR (600 MHz, 300 K, DMSO-d_6):

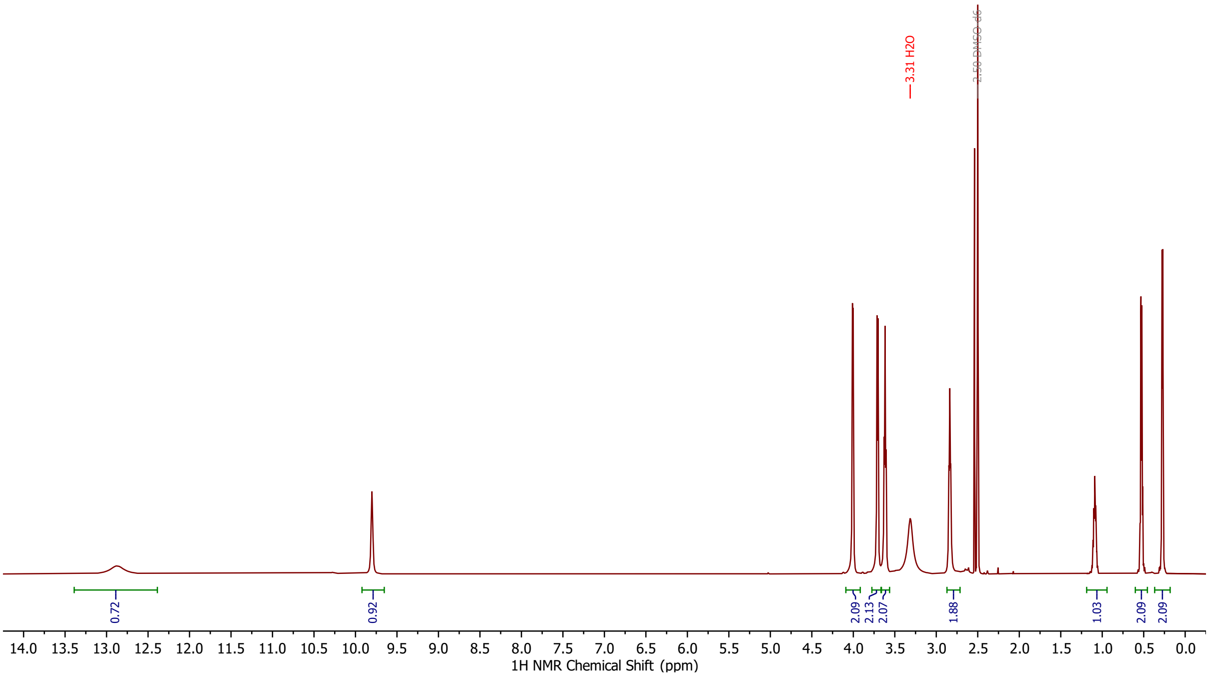

^{13}C NMR (151 MHz, 300 K, DMSO-d_6):

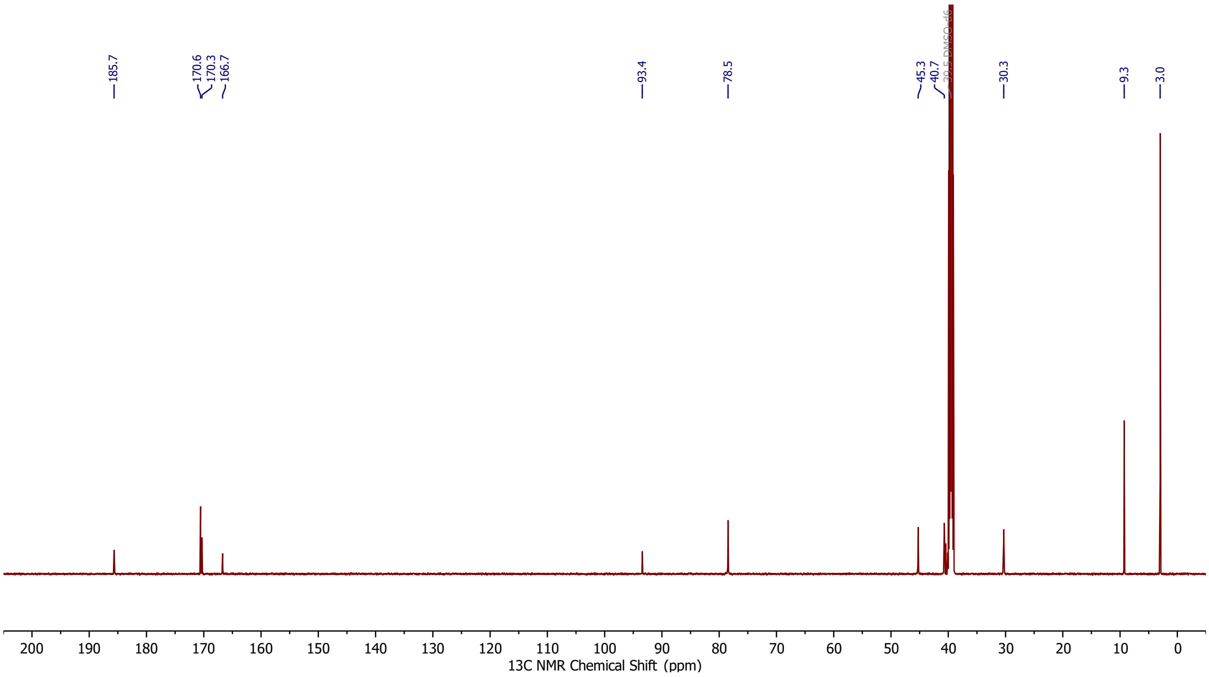

Ethyl 2-(1-(benzyloxy)-4-hydroxy-2-oxo-1,2-dihydroquinoline-3-carboxamido)-2-methylpropanoate (55)

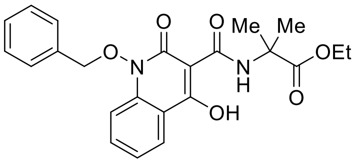

^{1}H NMR (600 MHz, 300 K, $CDCl_3$):

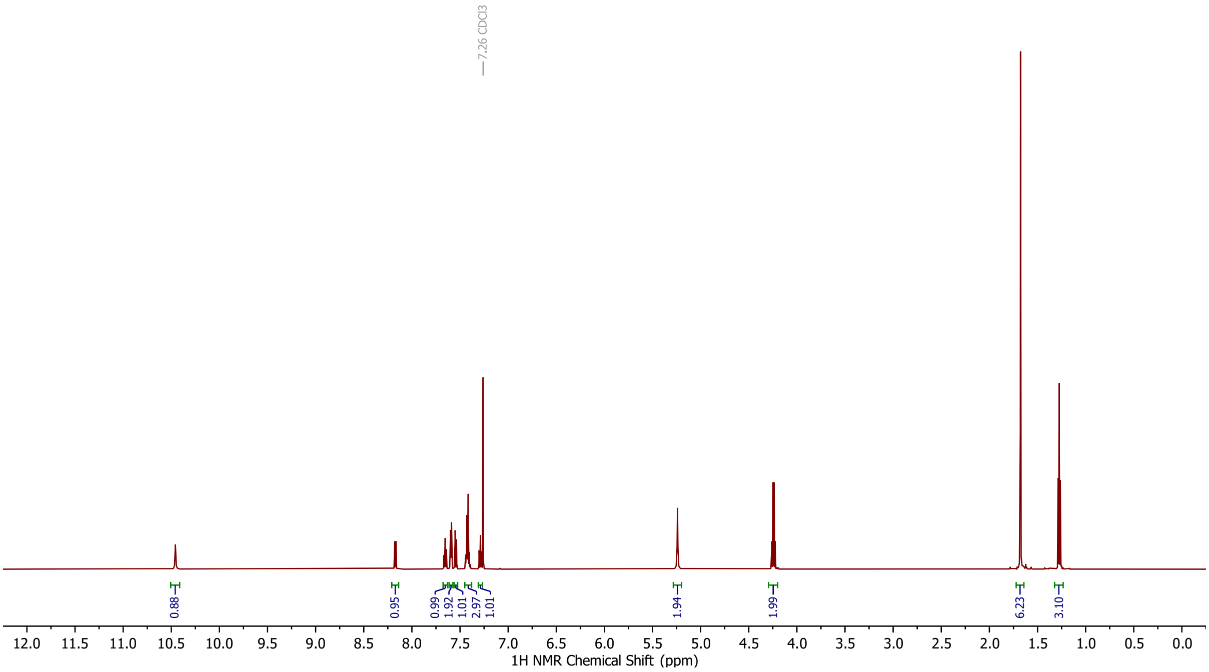

^{13}C NMR (151 MHz, 300 K, $CDCl_3$):

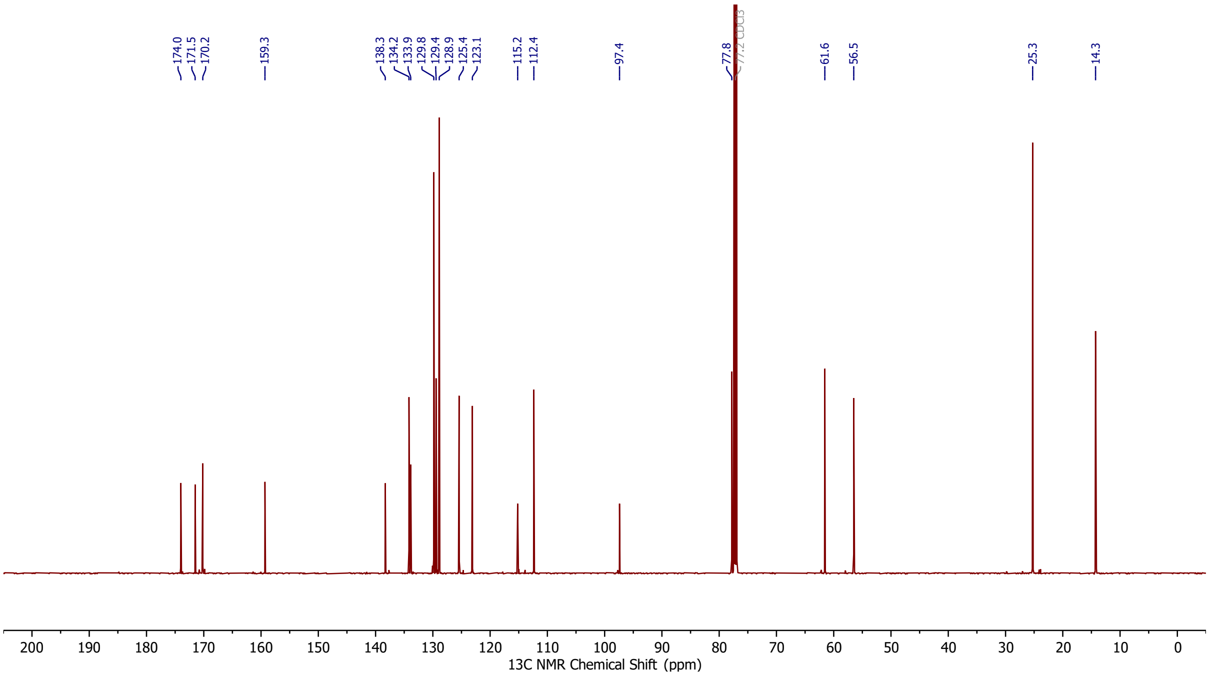

Ethyl 2-(1,4-dihydroxy-2-oxo-1,2-dihydroquinoline-3-carboxamido)-2-methylpropanoate (56)

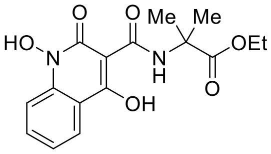

^{1}H NMR (500 MHz, 300 K, DMSO-d_6):

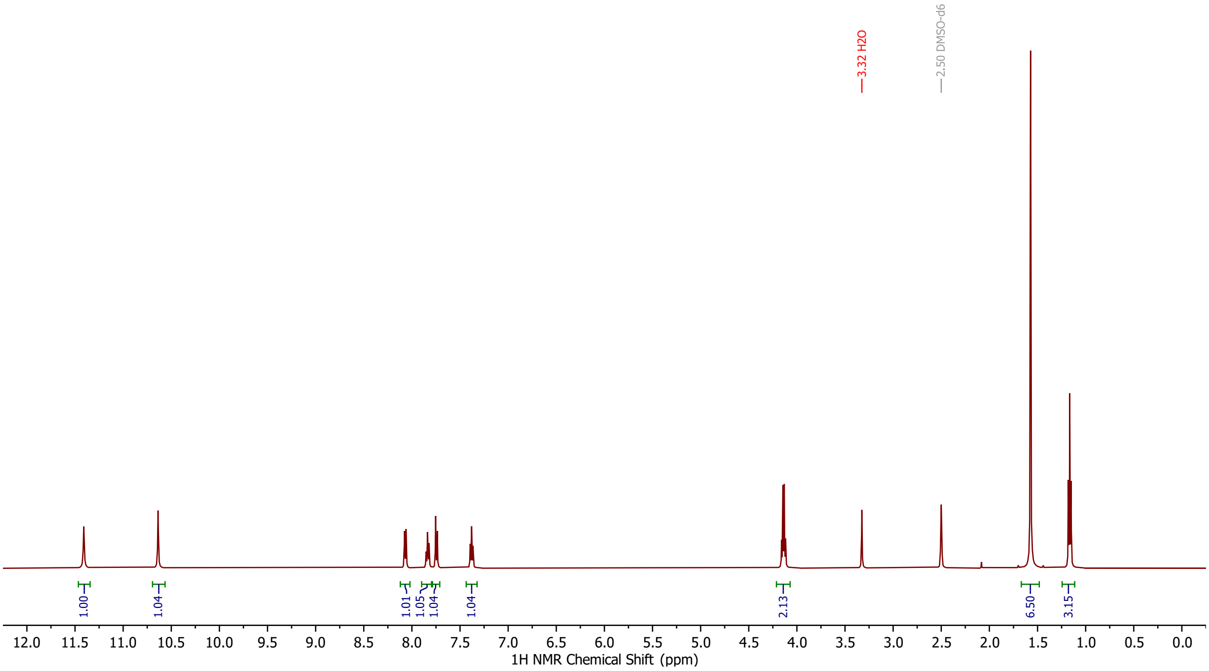

^{13}C NMR (126 MHz, 300 K, DMSO-d_6):

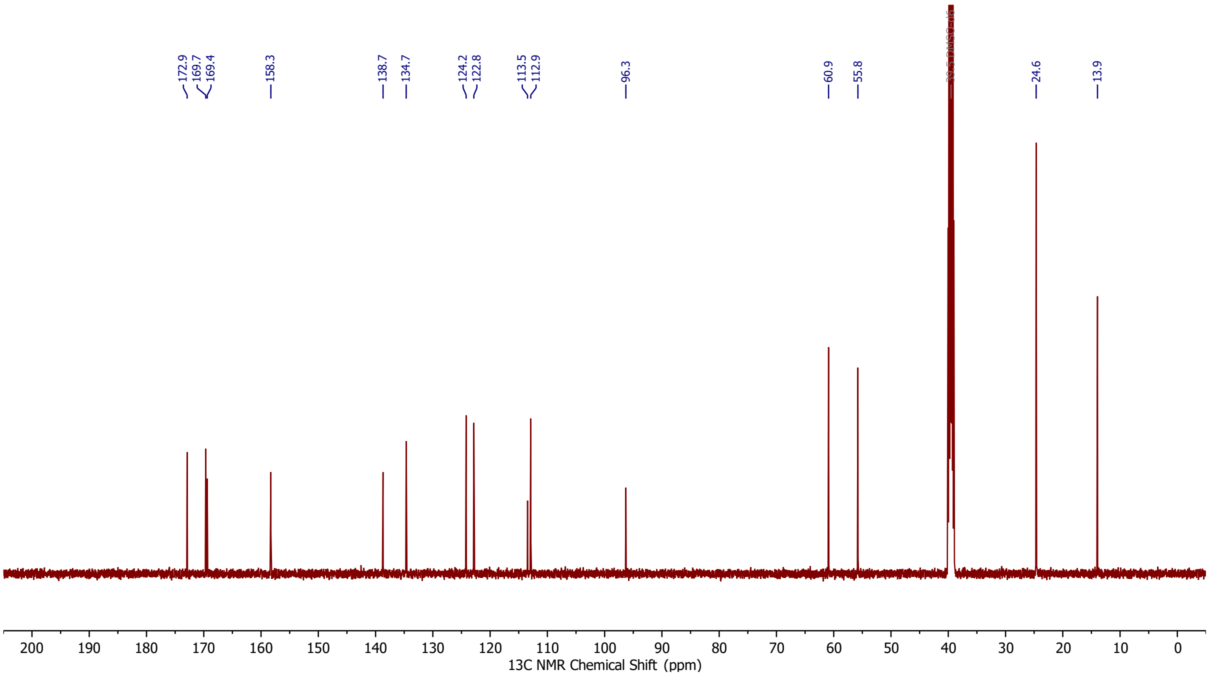

Ethyl 2-(4-hydroxy-2-oxo-1-(pyridin-2-ylmethoxy)-1,2-dihydroquinoline-3-carboxamido)-2-methylpropanoate (57a)

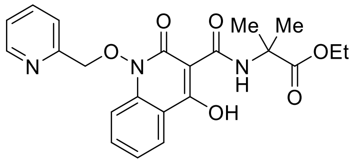

^1H NMR (600 MHz, 300 K, $CDCl_3$):

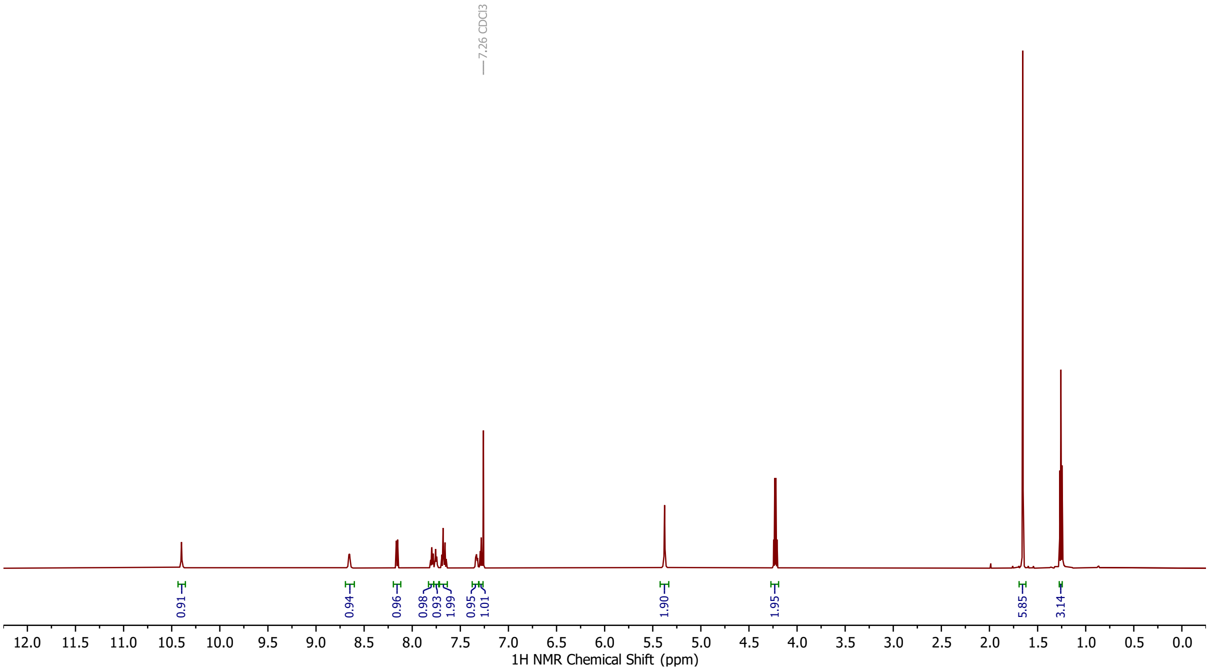

^{13}C NMR (151 MHz, 300 K, $CDCl_3$):

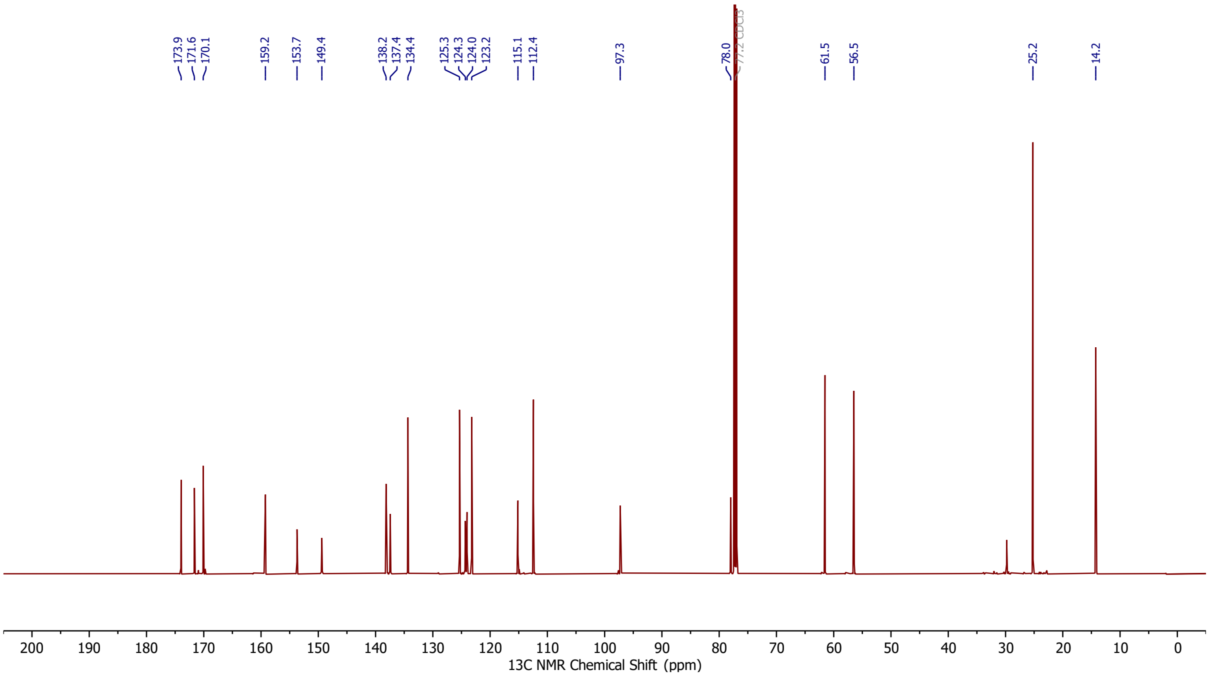

2-(4-Hydroxy-2-oxo-1-(pyridin-2-ylmethoxy)-1,2-dihydroquinoline-3-carboxamido)-2-methylpropanoic acid (57)

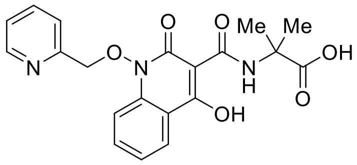

^{1}H NMR (600 MHz, 300 K, DMSO-d_6):

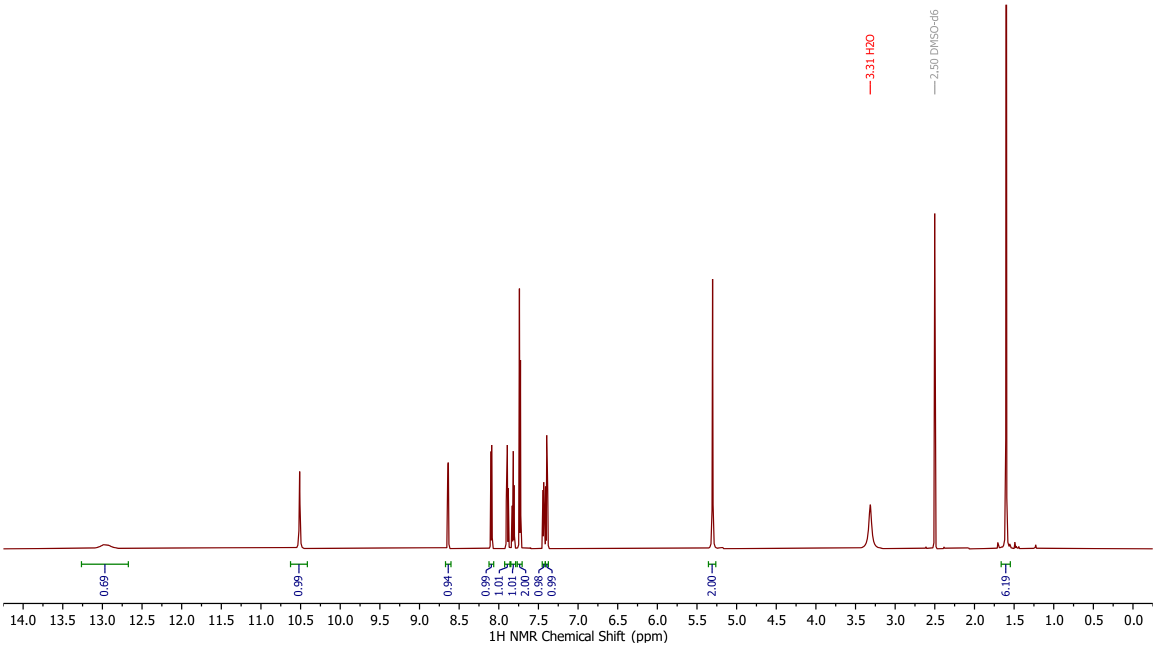

^{13}C NMR (151 MHz, 300 K, DMSO-d_6):

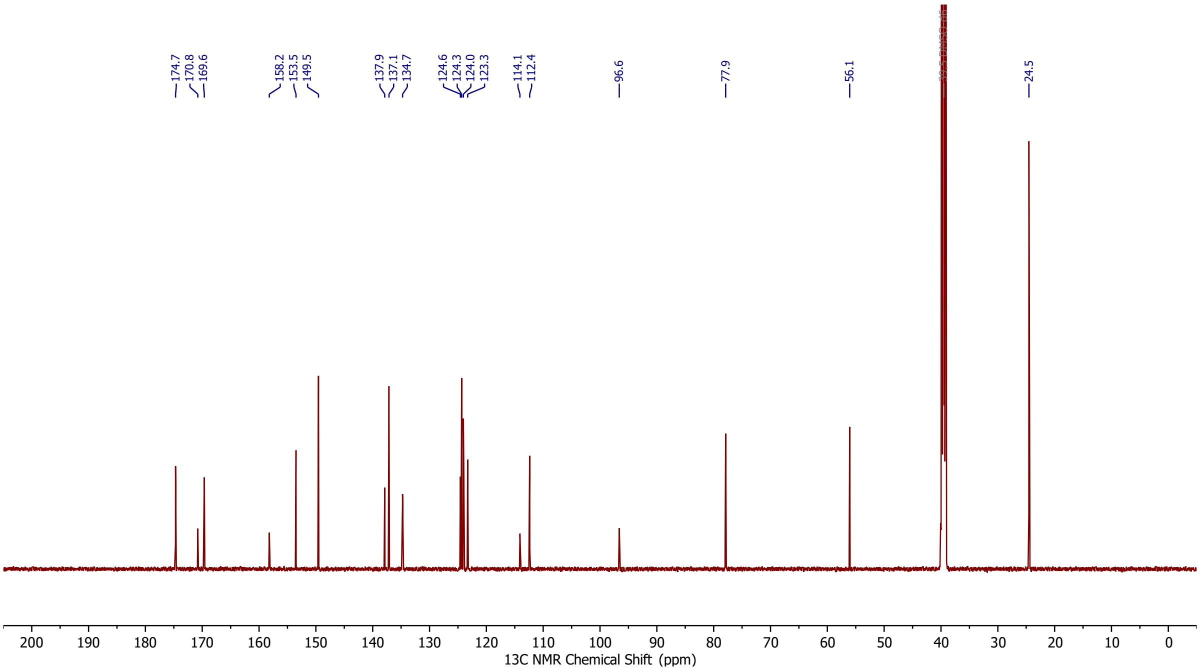

Ethyl 2-(4-hydroxy-2-oxo-1-(thiazol-4-ylmethoxy)-1,2-dihydroquinoline-3-carboxamido)-2-methylpropanoate (58a)

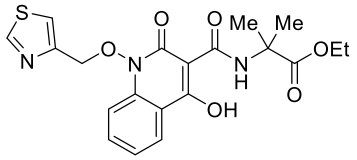

^{1}H NMR (600 MHz, 300 K, DMSO-d_6):

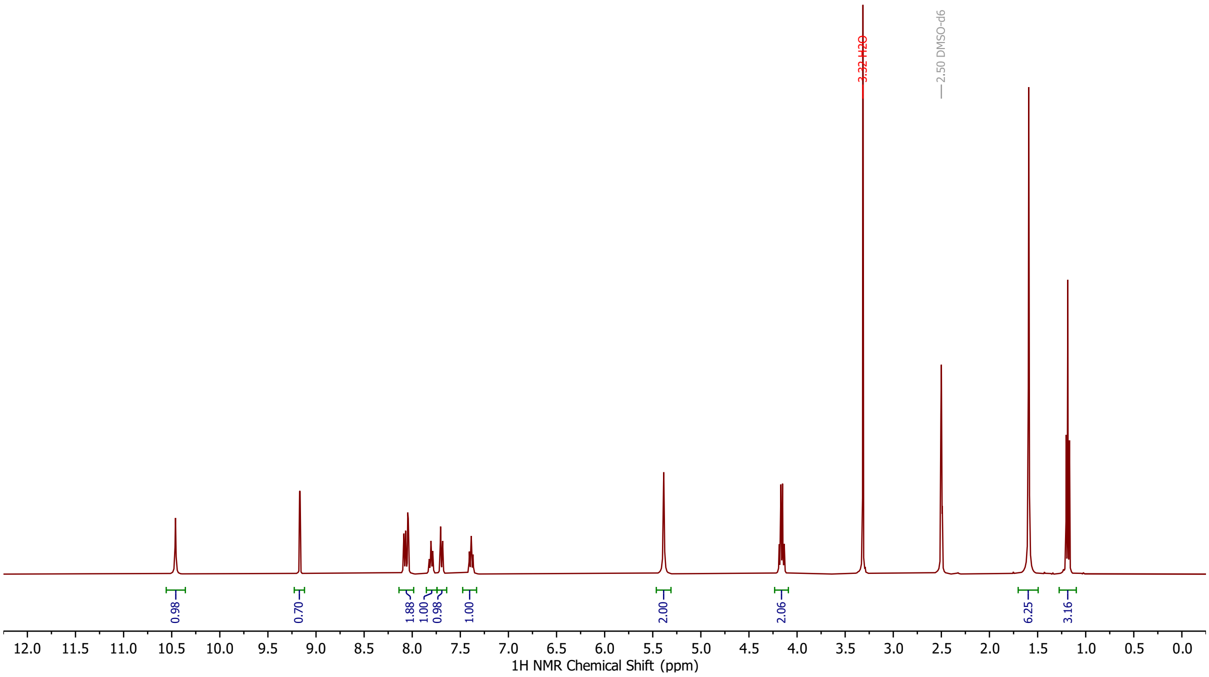

^{13}C NMR (151 MHz, 300 K, DMSO-d_6):

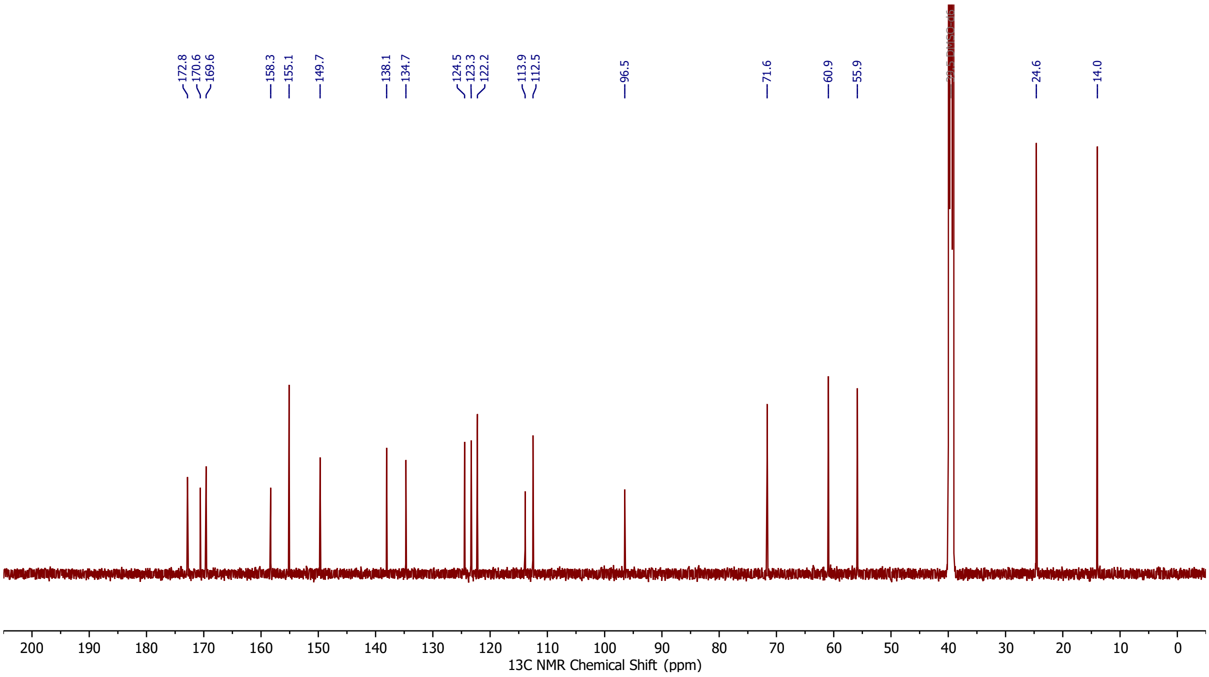

2-(4-Hydroxy-2-oxo-1-(thiazol-4-ylmethoxy)-1,2-dihydroquinoline-3-carboxamido)-2-methylpropanoic acid (58)

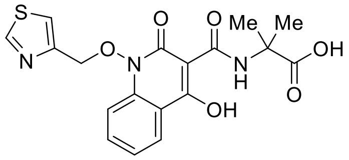

^{1}H NMR (600 MHz, 300 K, DMSO-d_6):

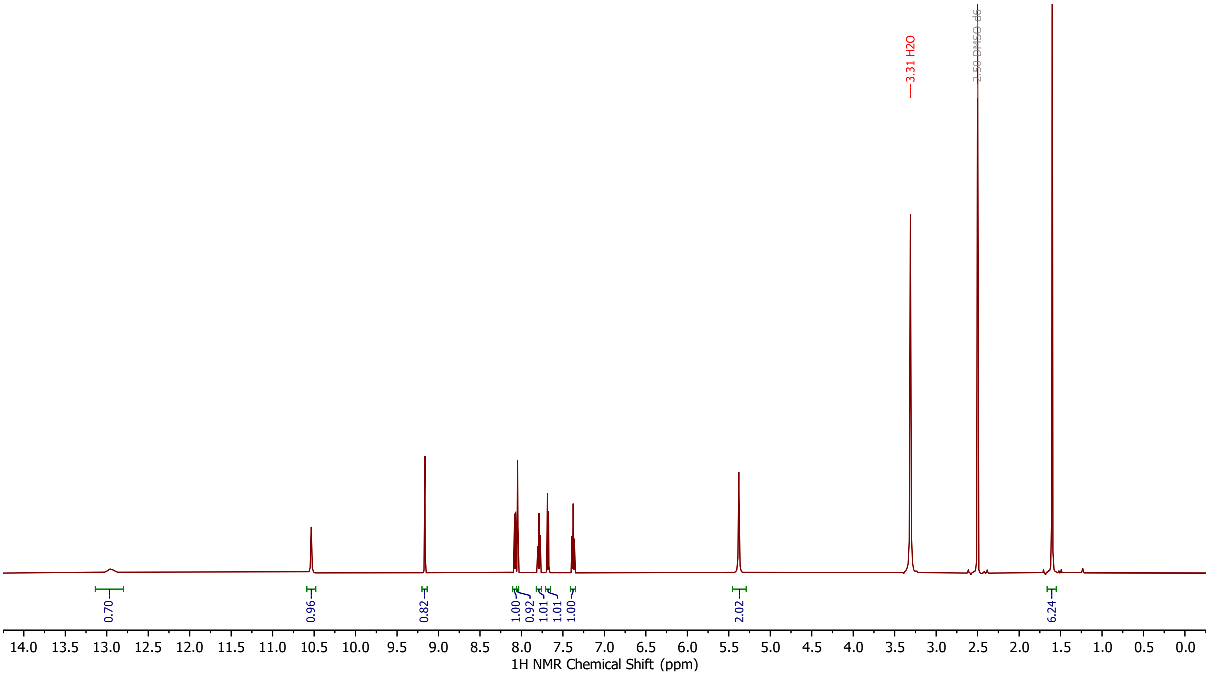

^{13}C NMR (151 MHz, 300 K, DMSO-d_6):

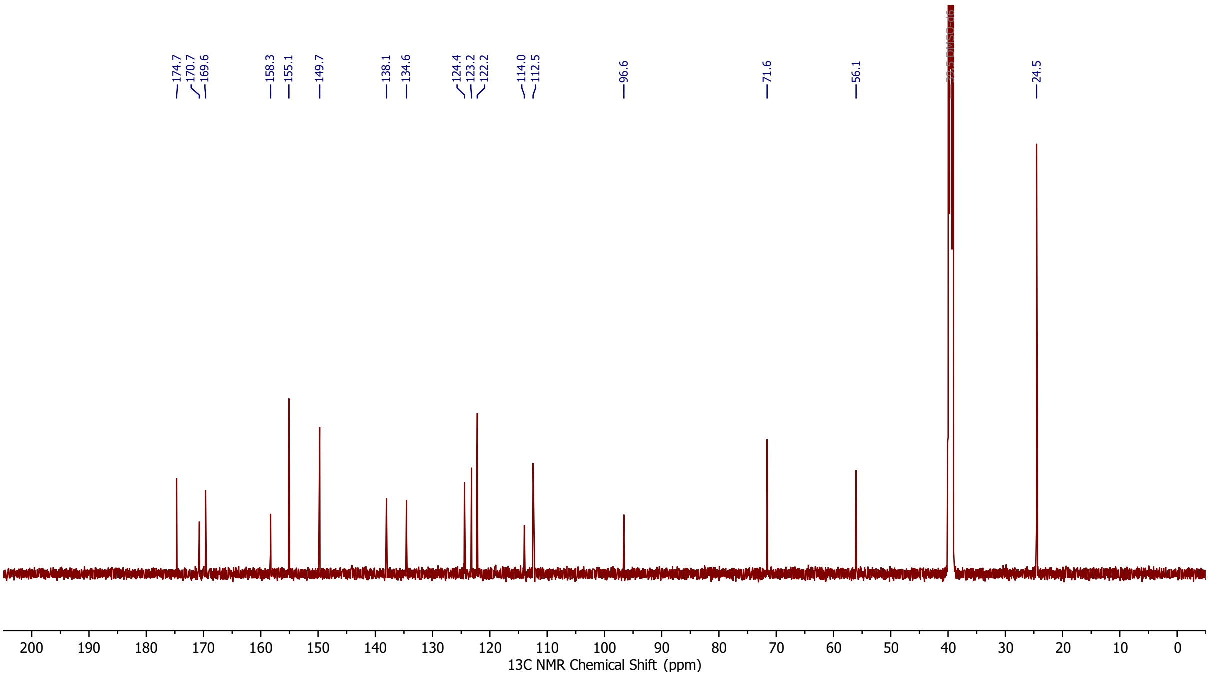

7. HPLC traces of final compounds prepared for this study

HPLC traces were recorded using an Agilent 1260 Infinity II HPLC system equipped with an Agilent InfinityLab Poroshell 120 EC-C18 column (dimensions: 150 mm length, 4.6 mm inner diameter, 4.0 µM particle size). A linear gradient (2-98%$_{v/v}$ over 10 min) of acetonitrile in water (each containing 0.1 %$_{v/v}$ formic acid), followed by a 5 min isocratic hold at 95%$_{v/v}$ acetonitrile in water, was used as eluent (flow rate: 1 mL/min; wavelength monitored: 254 nm). The %-area of the major peak is ≥95% with respect to the sum of the %-area of all peaks detected (excluding the injection peak at ~1.5-2.0 min).

(±)-(1-(Cyclopropylmethoxy)-4-hydroxy-2-oxo-1,2-dihydroquinoline-3-carbonyl)alanine (8)

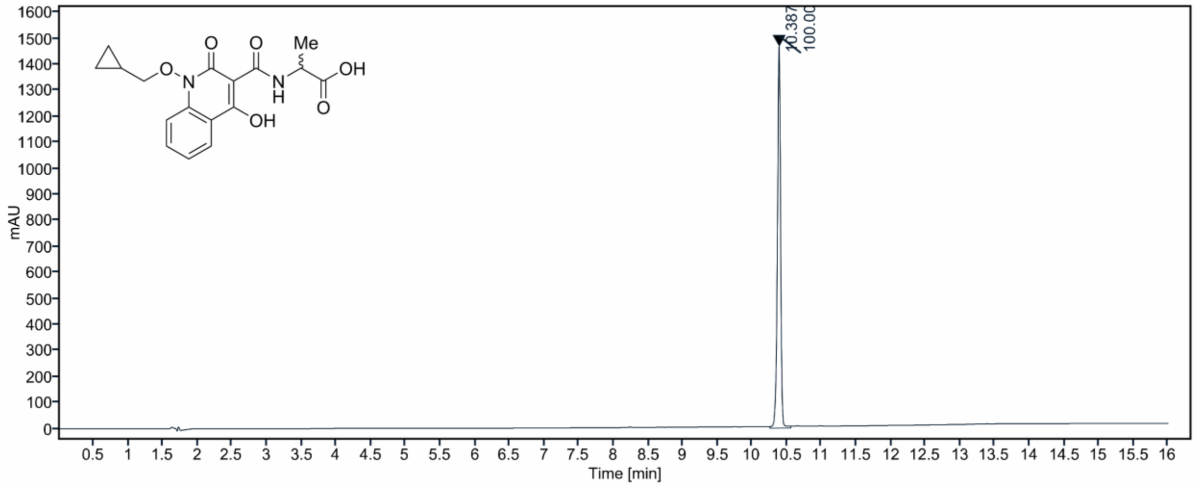

(±)-2-(1-(Cyclopropylmethoxy)-4-hydroxy-2-oxo-1,2-dihydroquinoline-3-carboxamido)butanoic acid (9)

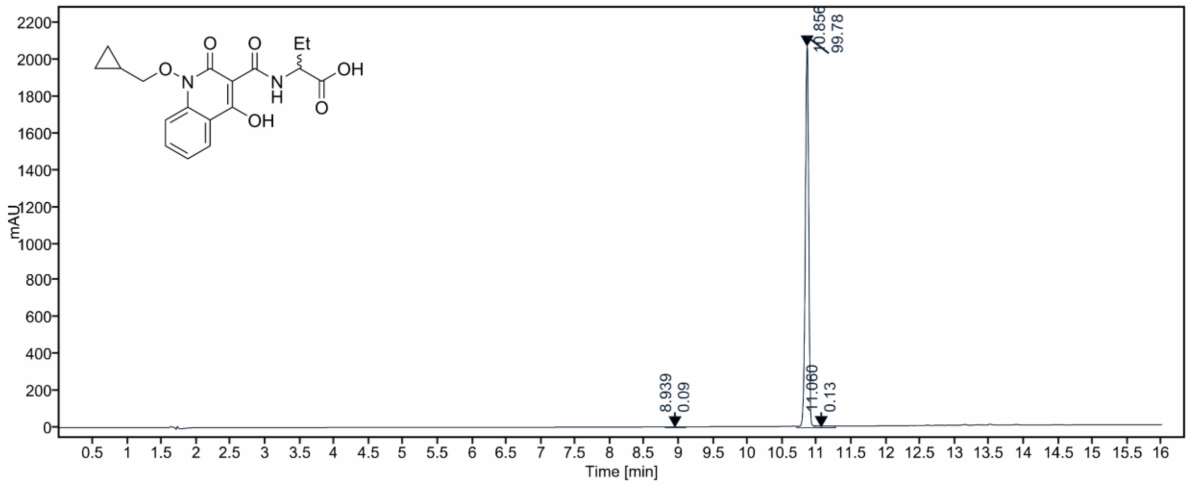

(±)-(1-(Cyclopropylmethoxy)-4-hydroxy-2-oxo-1,2-dihydroquinoline-3-carbonyl)valine (10)

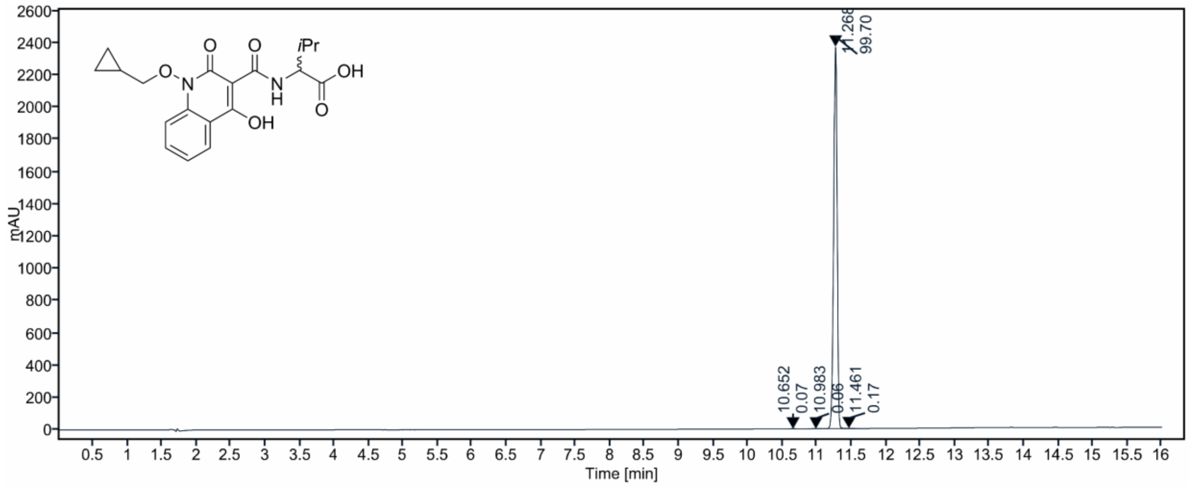

(±)-2-(1-(Cyclopropylmethoxy)-4-hydroxy-2-oxo-1,2-dihydroquinoline-3-carboxamido)-3,3,3-trifluoropropanoic acid (11)

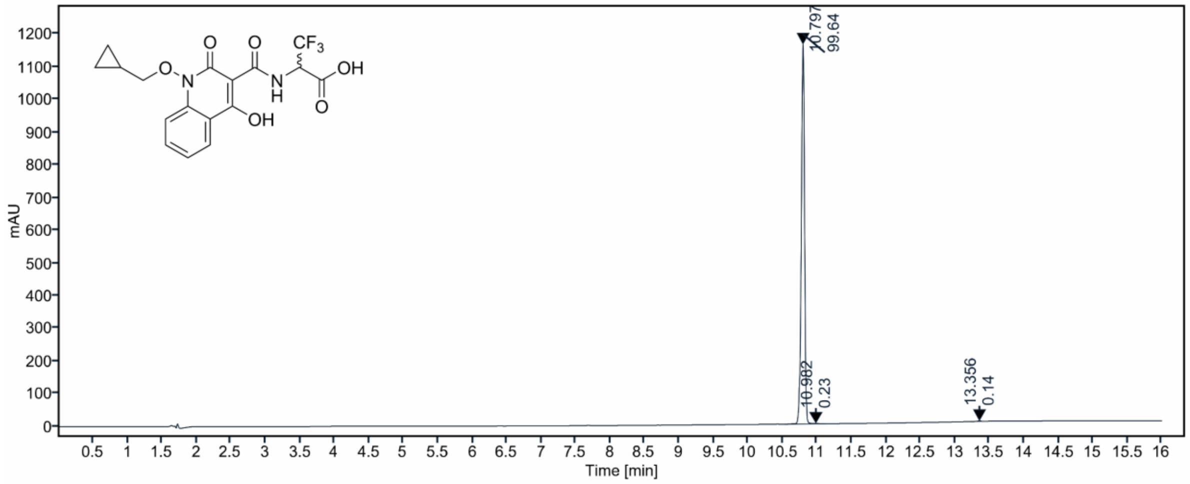

(±)-2-Cyclopropyl-2-(1-(cyclopropylmethoxy)-4-hydroxy-2-oxo-1,2-dihydroquinoline-3-carboxamido)acetic acid (12)

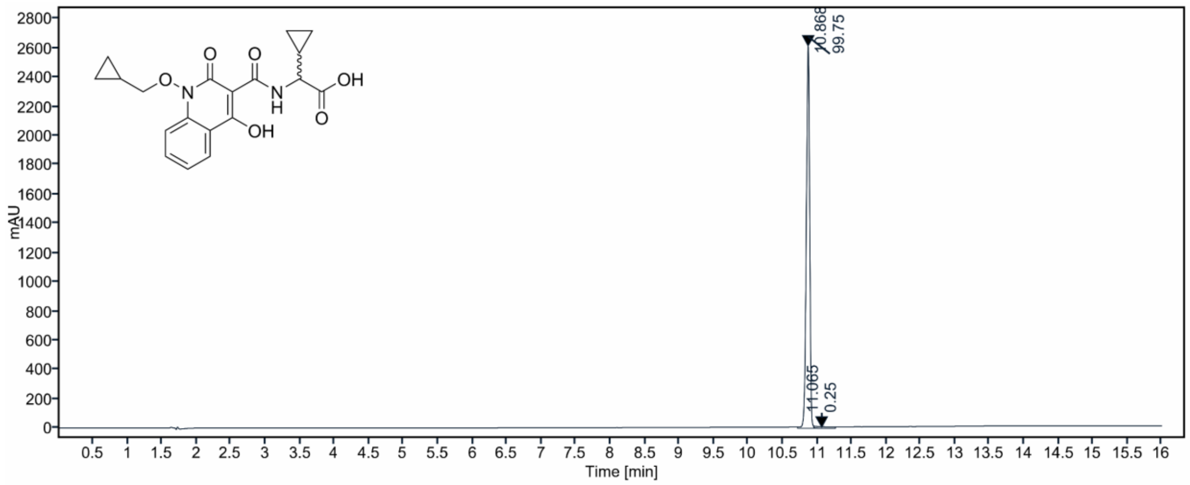

2-(1-(Cyclopropylmethoxy)-4-hydroxy-2-oxo-1,2-dihydroquinoline-3-carboxamido)-2-methylpropanoic acid (13)

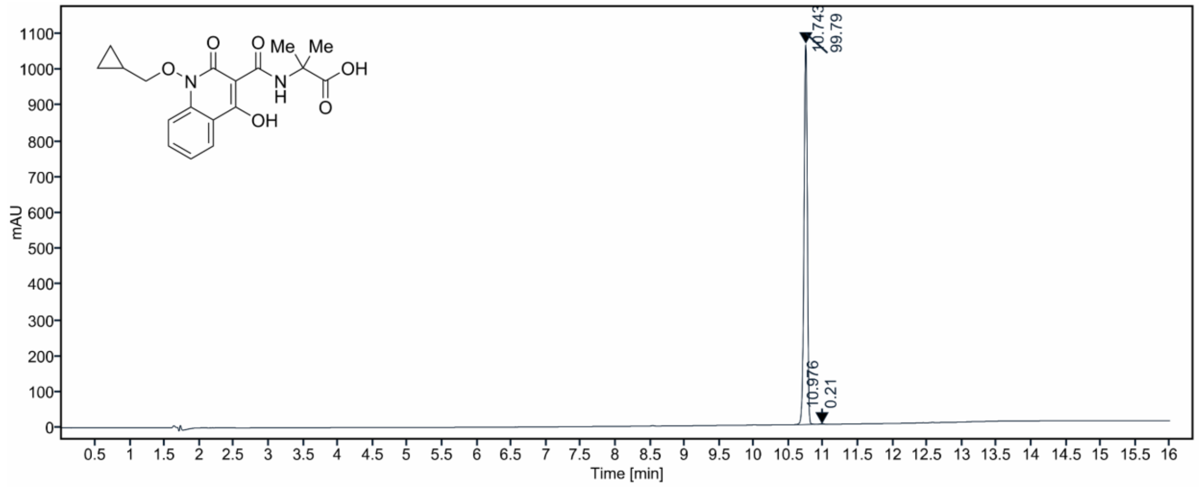

1-(1-(Cyclopropylmethoxy)-4-hydroxy-2-oxo-1,2-dihydroquinoline-3-carboxamido)cyclopropane-1-carboxylic acid (14)

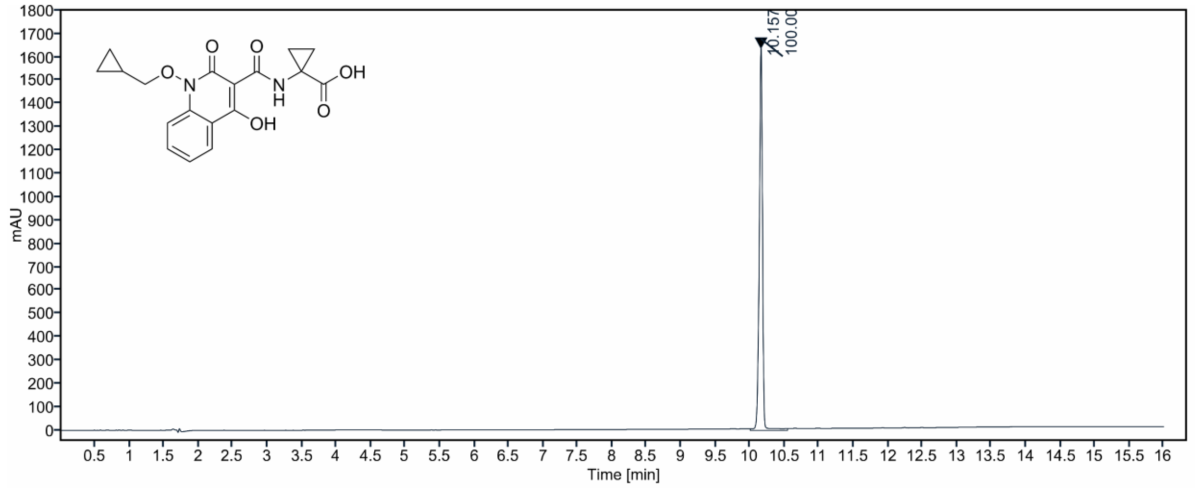

1-(1-(Cyclopropylmethoxy)-4-hydroxy-2-oxo-1,2-dihydroquinoline-3-carboxamido)cyclobutane-1-carboxylic acid (15)

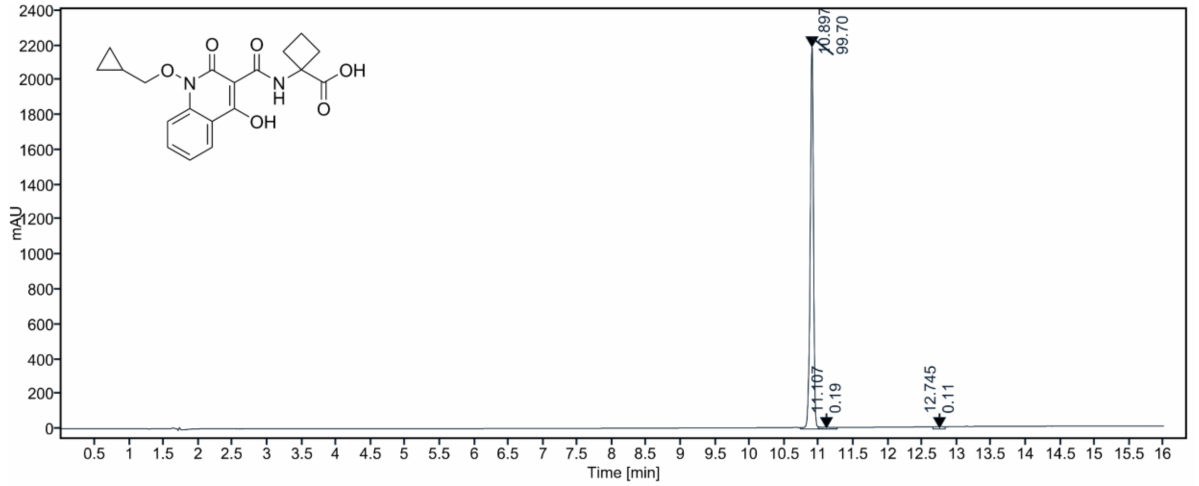

(±)-2-(1-(Cyclopropylmethoxy)-4-hydroxy-2-oxo-1,2-dihydroquinoline-3-carboxamido)-2-phenylacetic acid (16)

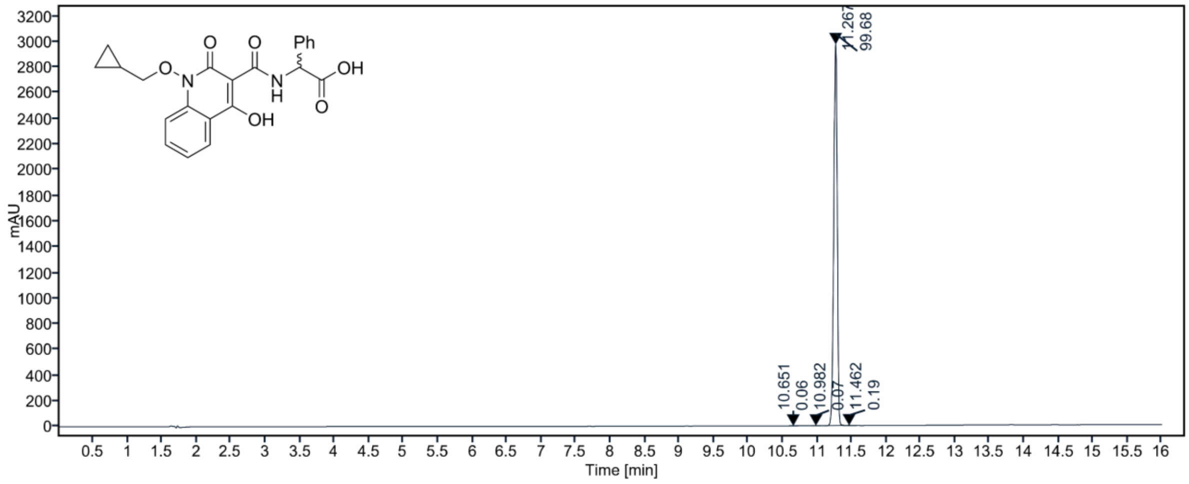

(±)-(1-(Cyclopropylmethoxy)-4-hydroxy-2-oxo-1,2-dihydroquinoline-3-carbonyl)phenylalanine (17)

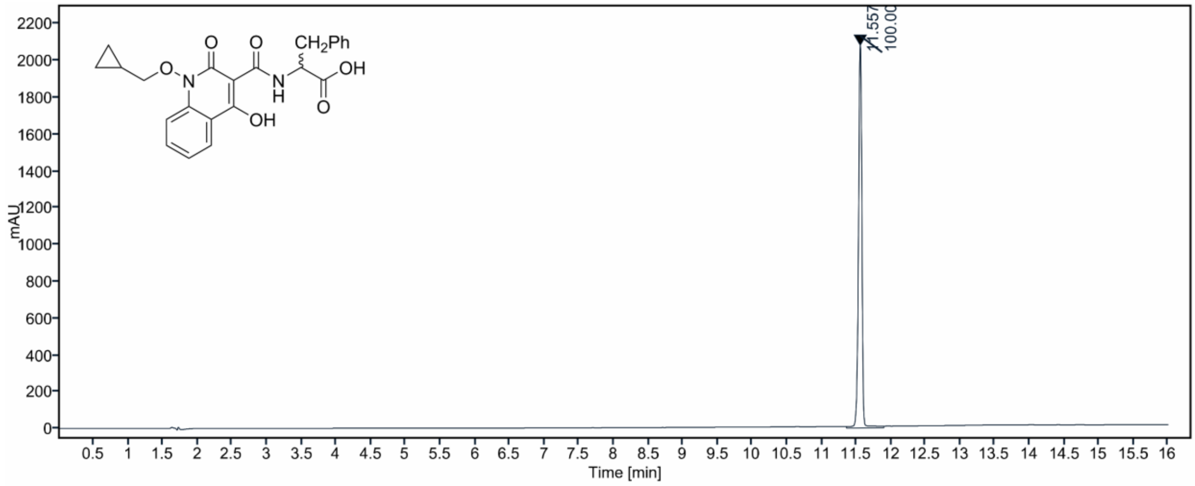

(±)-2-(1-(Cyclopropylmethoxy)-4-hydroxy-2-oxo-1,2-dihydroquinoline-3-carboxamido)-4-phenyl-butanoic acid (18)

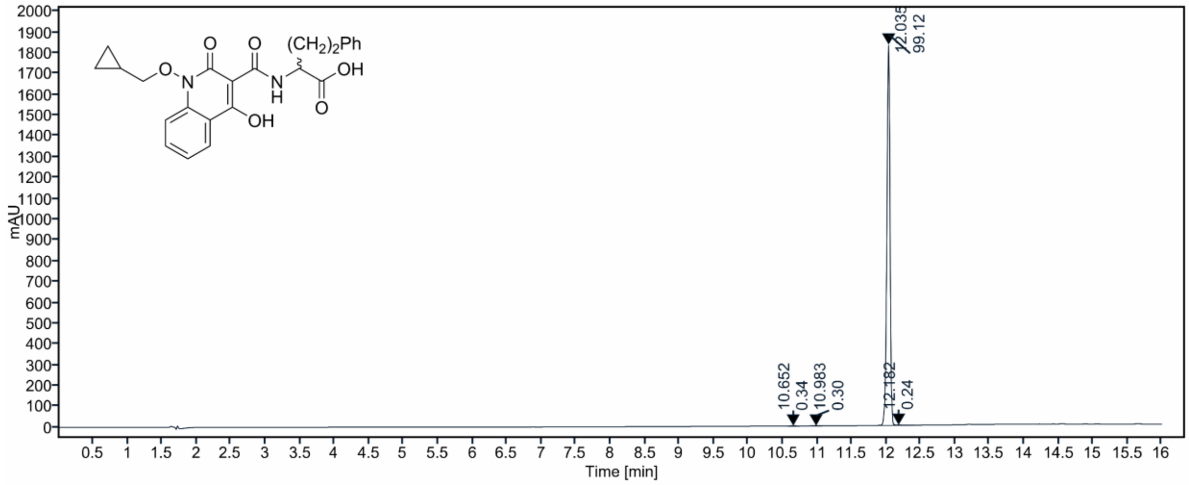

(±)-(1-(Cyclopropylmethoxy)-4-hydroxy-2-oxo-1,2-dihydroquinoline-3-carbonyl)tryptophan (19)

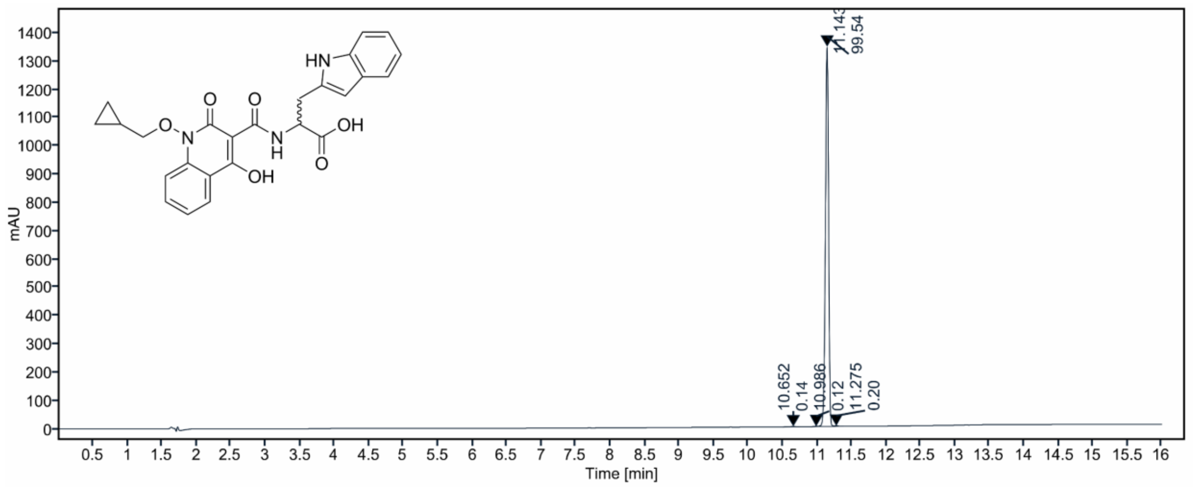

1-(Cyclopropylmethoxy)-4-hydroxy-2-oxo-1,2-dihydroquinoline-3-carboxamide (20)

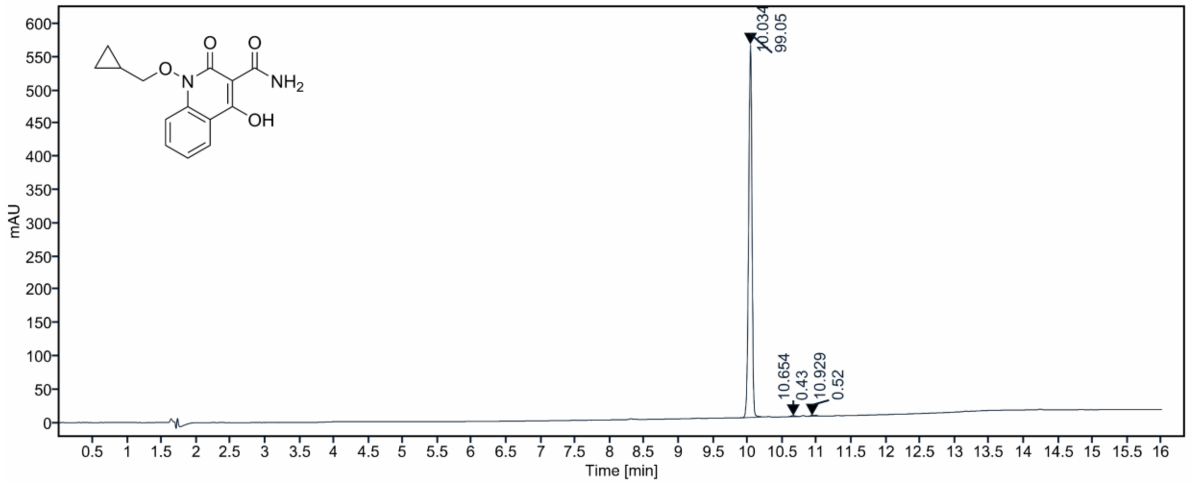

3-(1-(Cyclopropylmethoxy)-4-hydroxy-2-oxo-1,2-dihydroquinoline-3-carboxamido)propanoic acid (21)

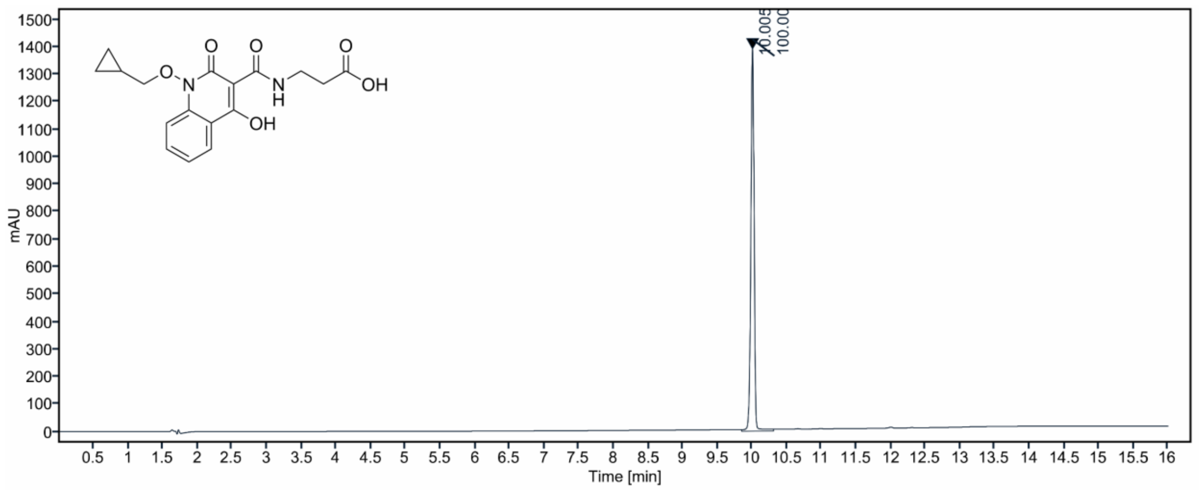

_N_-(1-(Cyclopropylmethoxy)-4-hydroxy-2-oxo-1,2-dihydroquinoline-3-carbonyl)-_N_-methylglycine (22)

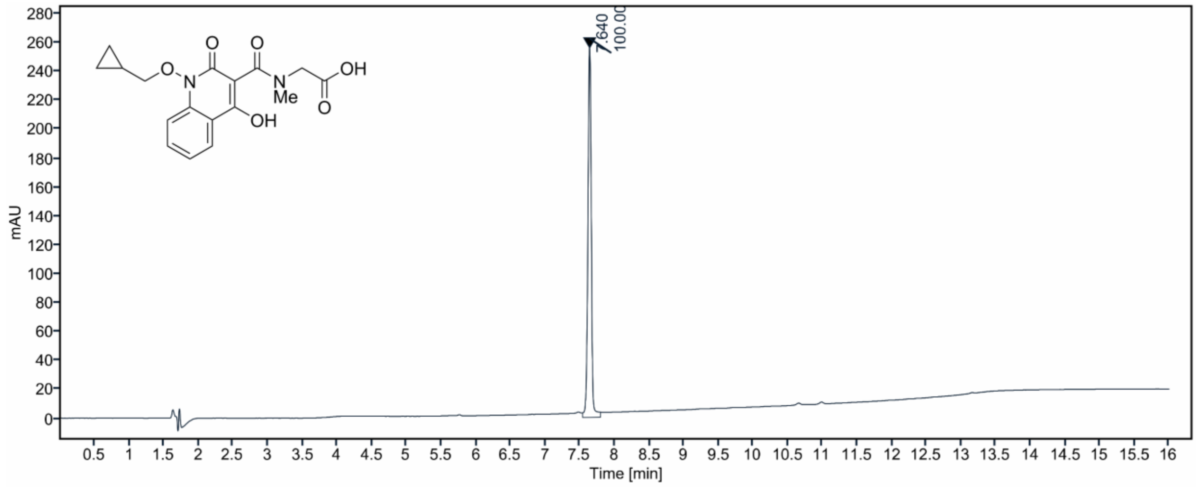

Methyl (1-(cyclopropylmethoxy)-4-hydroxy-2-oxo-1,2-dihydroquinoline-3-carbonyl)glycinate (23)

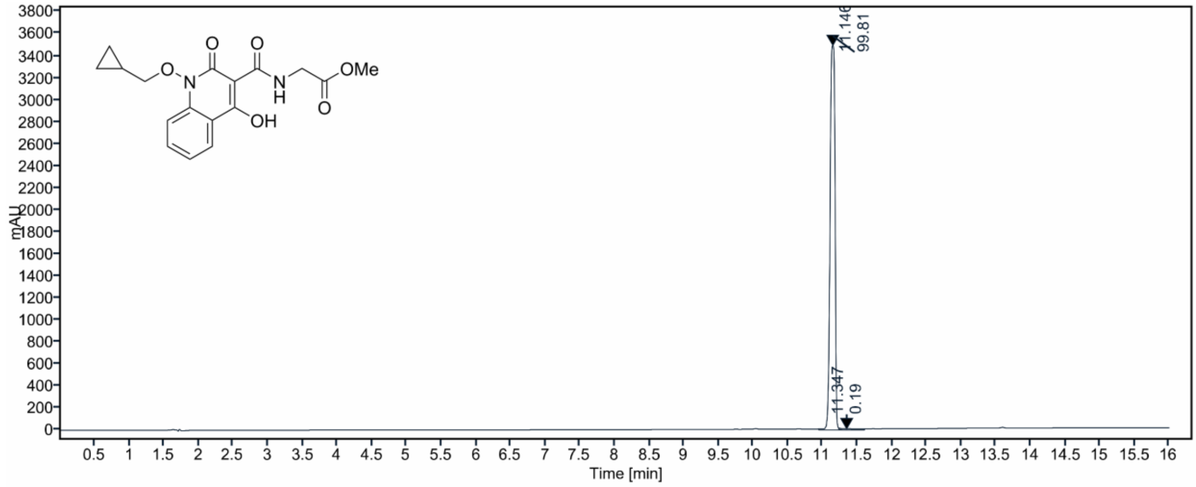

Ethyl (1-(cyclopropylmethoxy)-4-hydroxy-2-oxo-1,2-dihydroquinoline-3-carbonyl)glycinate (24)

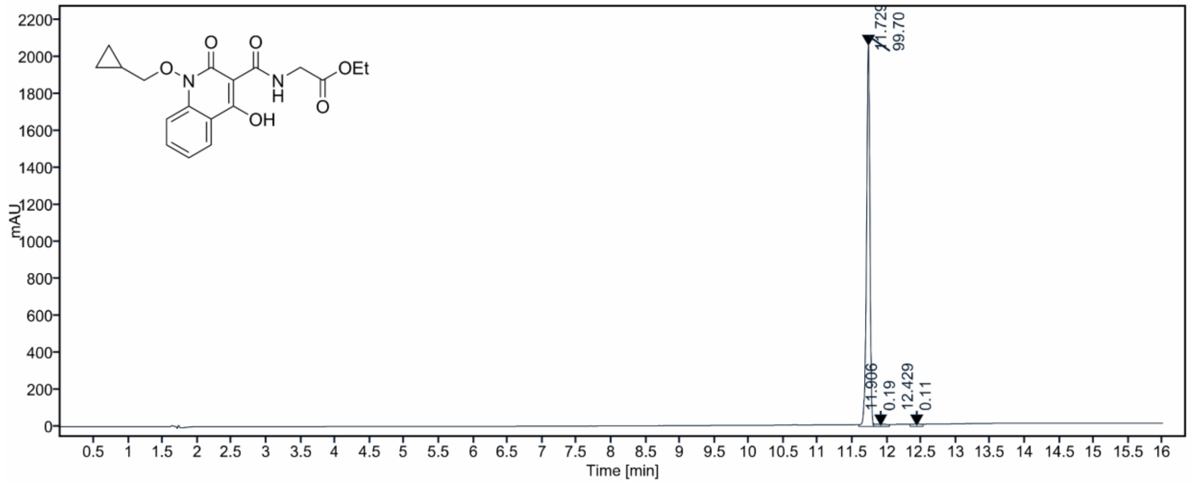

(1-(Cyclopropylmethoxy)-4-hydroxy-2-oxo-1,2-dihydroquinoline-3-carbonyl)glycine – (Desidustat)

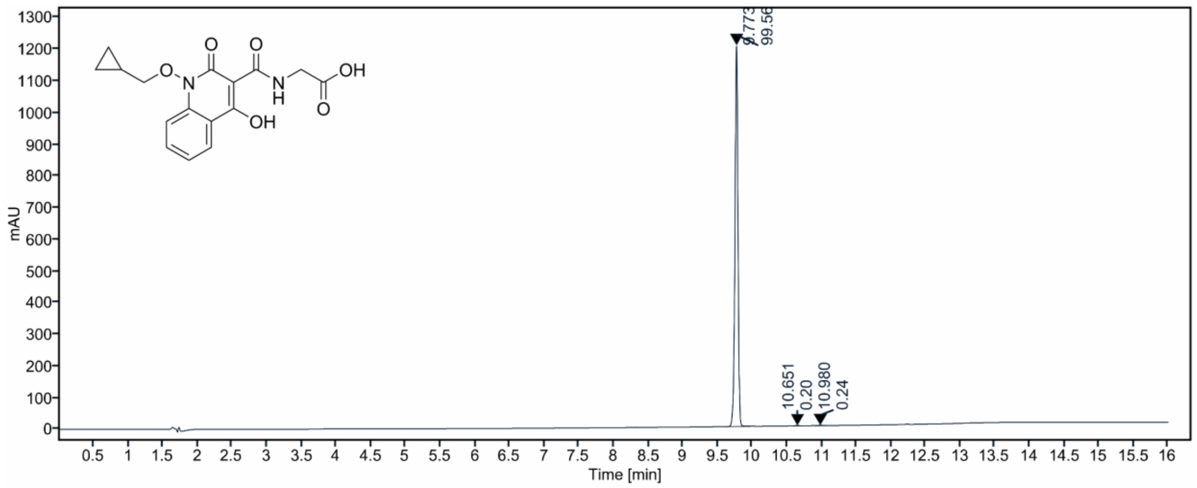

***N*-(2-Amino-2-oxoethyl)-1-(cyclopropylmethoxy)-4-hydroxy-2-oxo-1,2-dihydroquinoline-3-carboxamide (25)**

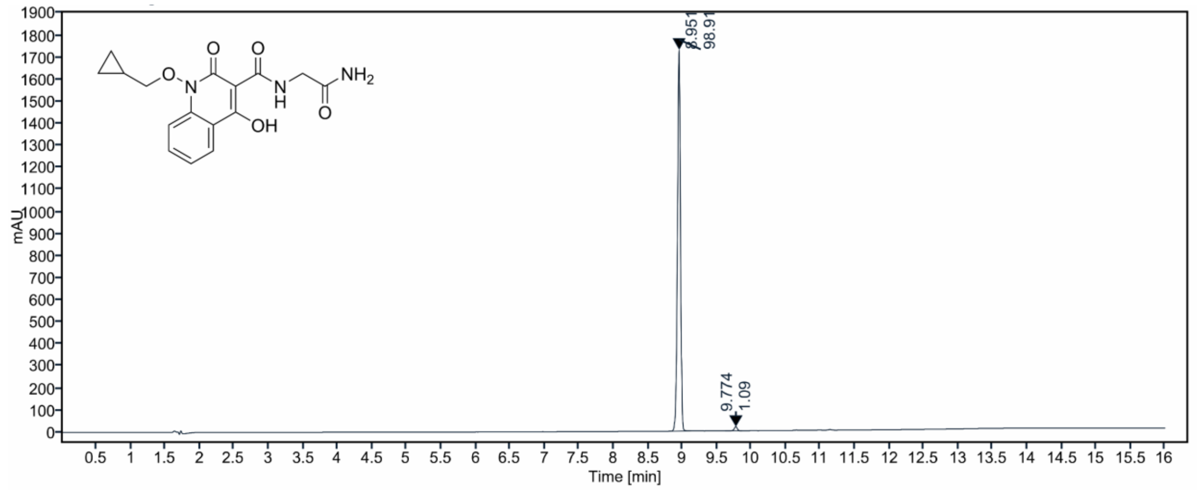

1-(Cyclopropylmethoxy)-4-hydroxy-*N*-(2-(methylamino)-2-oxoethyl)-2-oxo-1,2-dihydroquinoline-3-carboxamide (26)

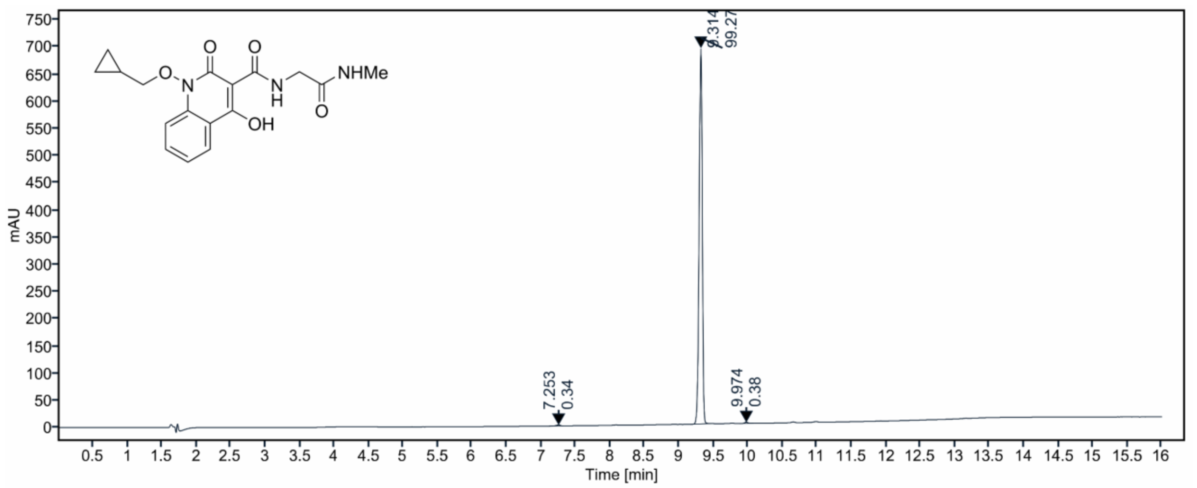

***N*-(Cyanomethyl)-1-(cyclopropylmethoxy)-4-hydroxy-2-oxo-1,2-dihydroquinoline-3-carboxamide (27)**

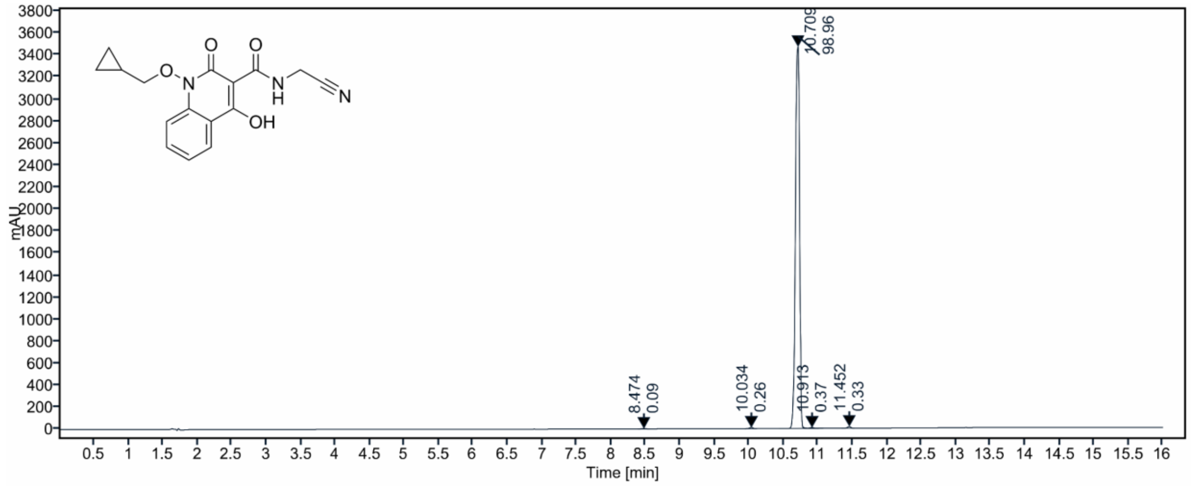

(±)-1-(Cyclopropylmethoxy)-4-hydroxy-2-oxo-*N*-(3,3,3-trifluoro-2-hydroxypropyl)-1,2-dihydroquinoline-3-carboxamide (28)

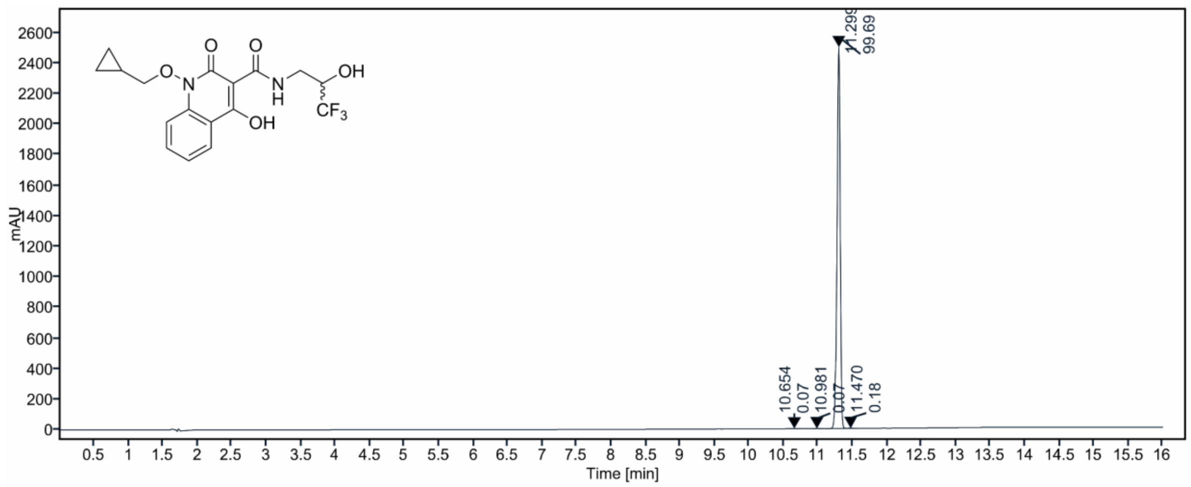

1-(Cyclopropylmethoxy)-4-hydroxy-*N*-((3-hydroxyoxetan-3-yl)methyl)-2-oxo-1,2-dihydroquinoline-3-carboxamide (29)

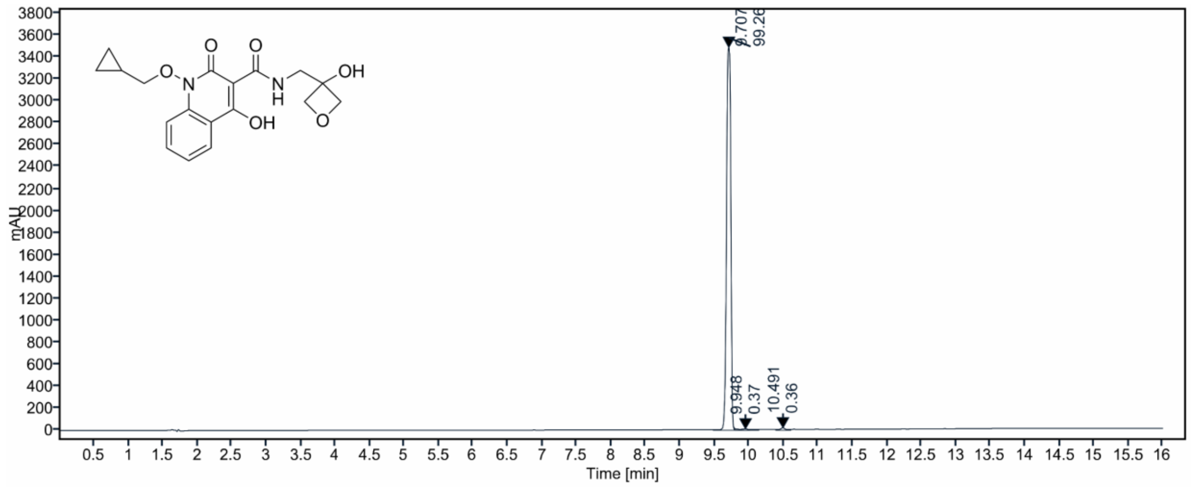

***N*-((1*H*-1,2,3-Triazol-1-yl)methyl)-1-(cyclopropylmethoxy)-4-hydroxy-2-oxo-1,2-dihydroquinoline-3-carboxamide (30)**

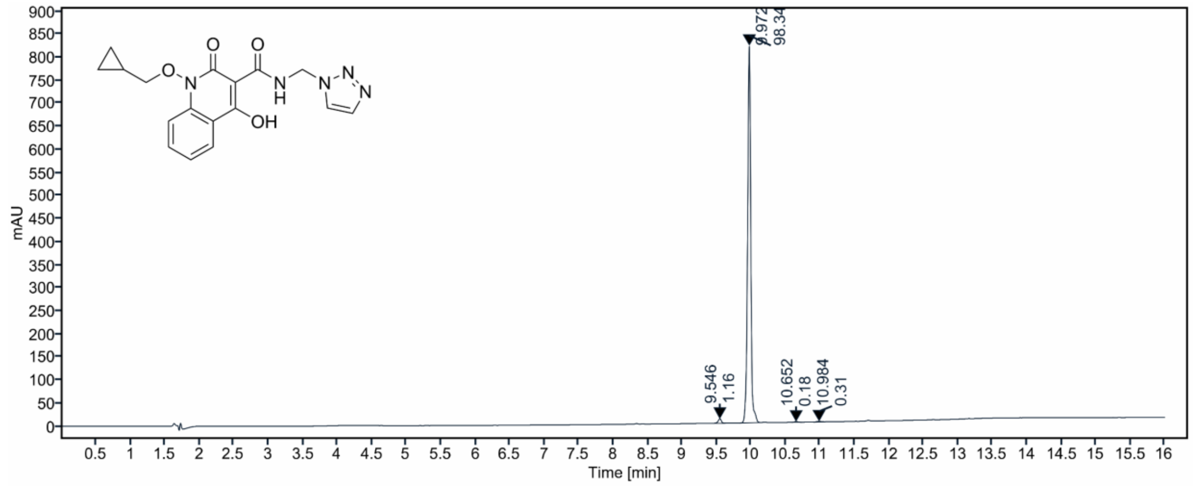

***N*-((1*H*-1,2,4-Triazol-1-yl)methyl)-1-(cyclopropylmethoxy)-4-hydroxy-2-oxo-1,2-dihydroquinoline-3-carboxamide (31)**

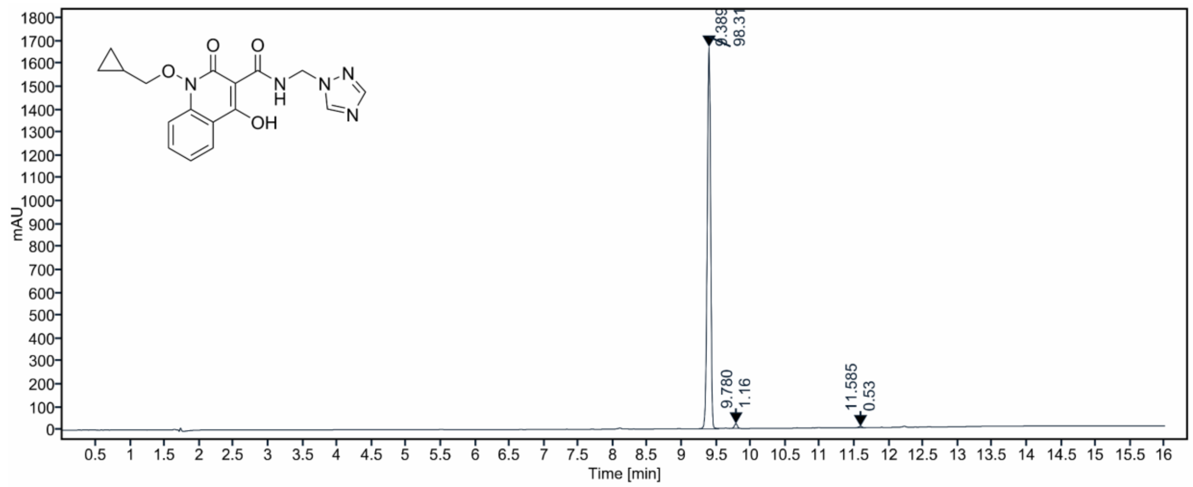

***N*-((2*H*-Tetrazol-5-yl)methyl)-1-(cyclopropylmethoxy)-4-hydroxy-2-oxo-1,2-dihydroquinoline-3-carboxamide (32)**

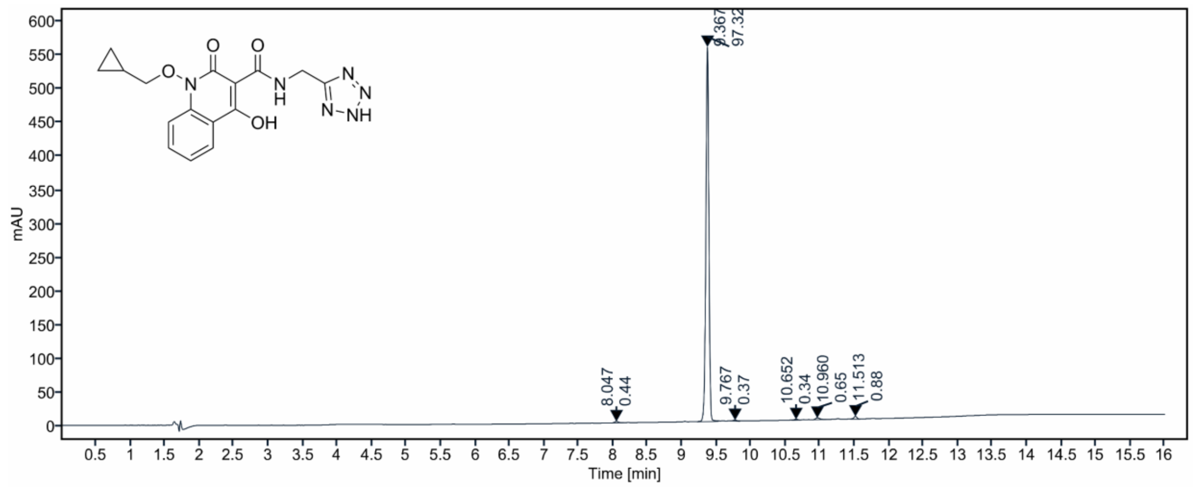

(4-Hydroxy-2-oxo-1-(thiazol-4-ylmethoxy)-1,2-dihydroquinoline-3-carbonyl)glycine (41)

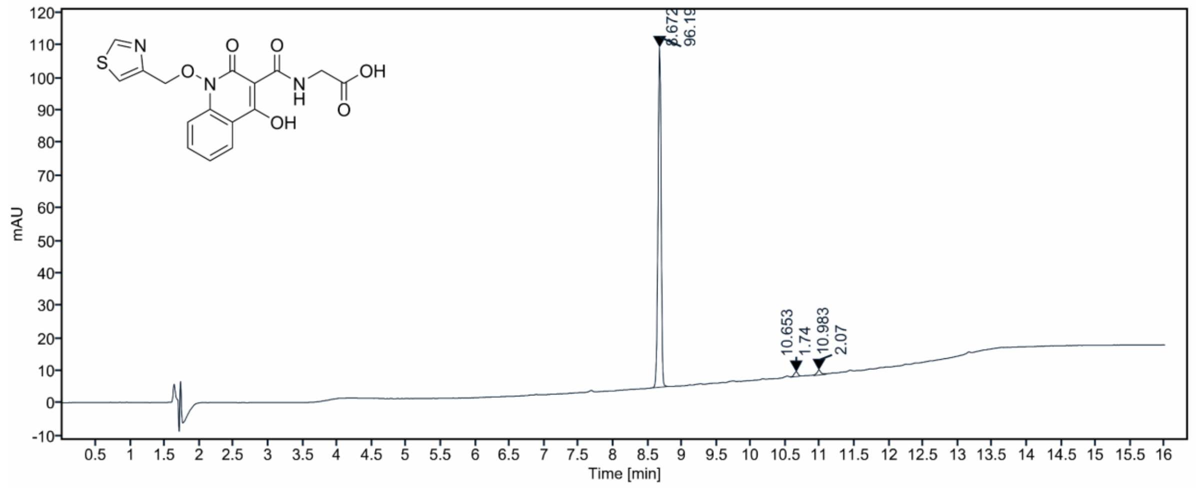

(4-Hydroxy-1-(isoxazol-3-ylmethoxy)-2-oxo-1,2-dihydroquinoline-3-carbonyl)glycine (42)

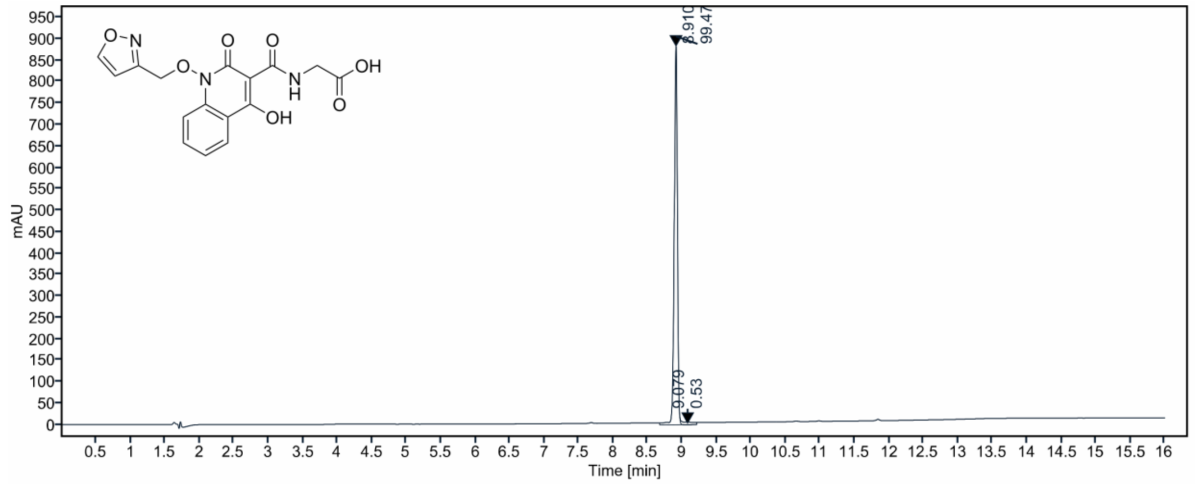

(4-Hydroxy-1-((1-methyl-1*H*-imidazol-4-yl)methoxy)-2-oxo-1,2-dihydroquinoline-3-carbonyl)glycine (43)

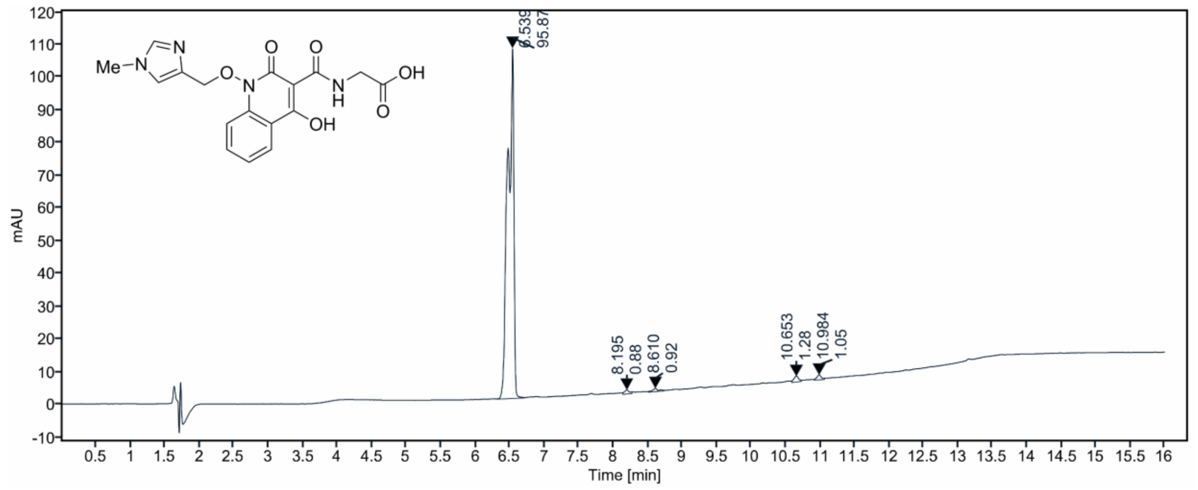

(4-Hydroxy-2-oxo-1-(pyridin-4-ylmethoxy)-1,2-dihydroquinoline-3-carbonyl)glycine (44)

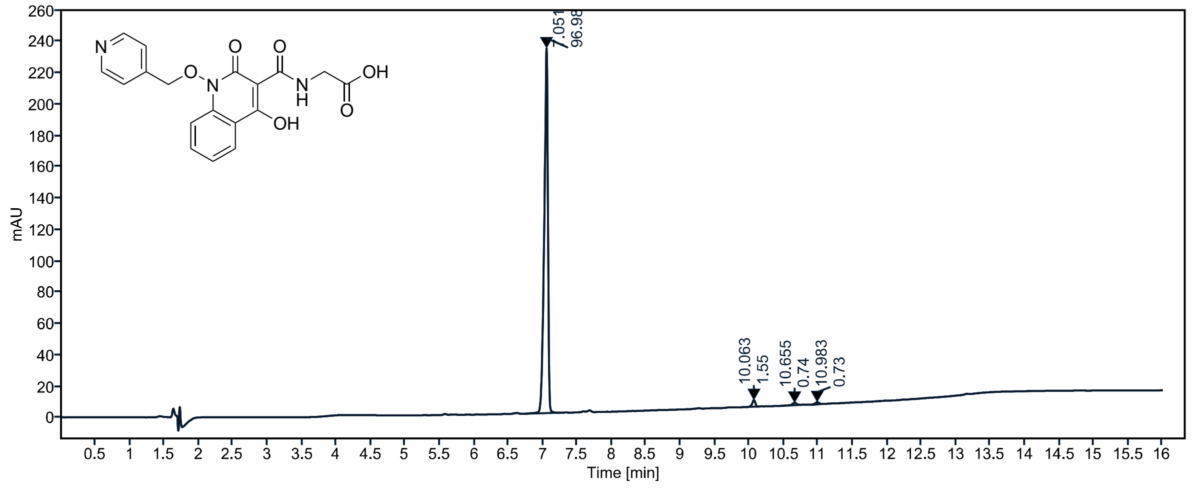

(4-Hydroxy-2-oxo-1-((tetrahydro-2*H*-pyran-4-yl)methoxy)-1,2-dihydroquinoline-3-carbonyl)glycine (45)

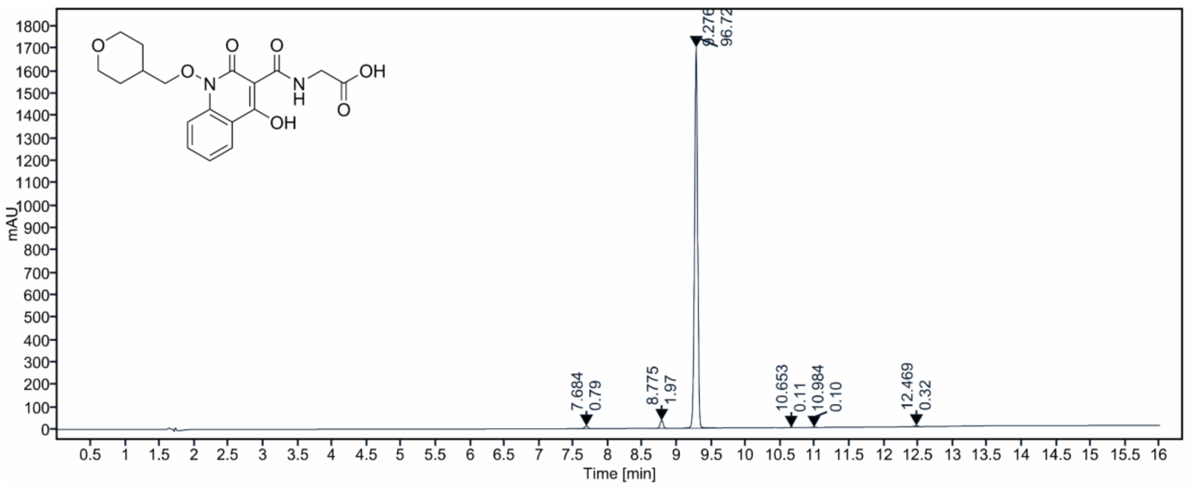

(4-Hydroxy-2-oxo-1-(2,2,2-trifluoroethoxy)-1,2-dihydroquinoline-3-carbonyl)glycine (46)

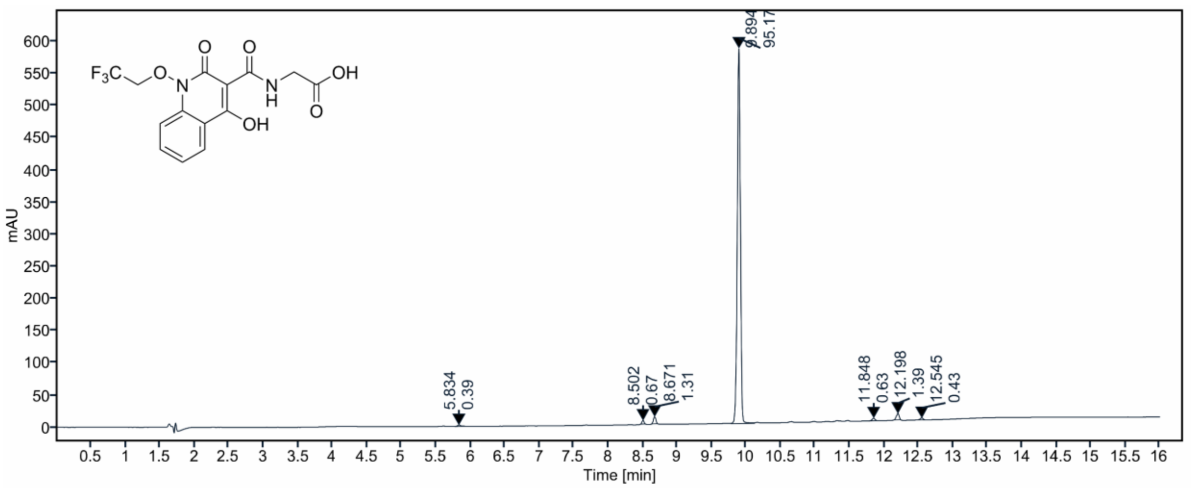

(1-(Benzyloxy)-4-hydroxy-2-oxo-1,2-dihydroquinoline-3-carbonyl)glycine (47)

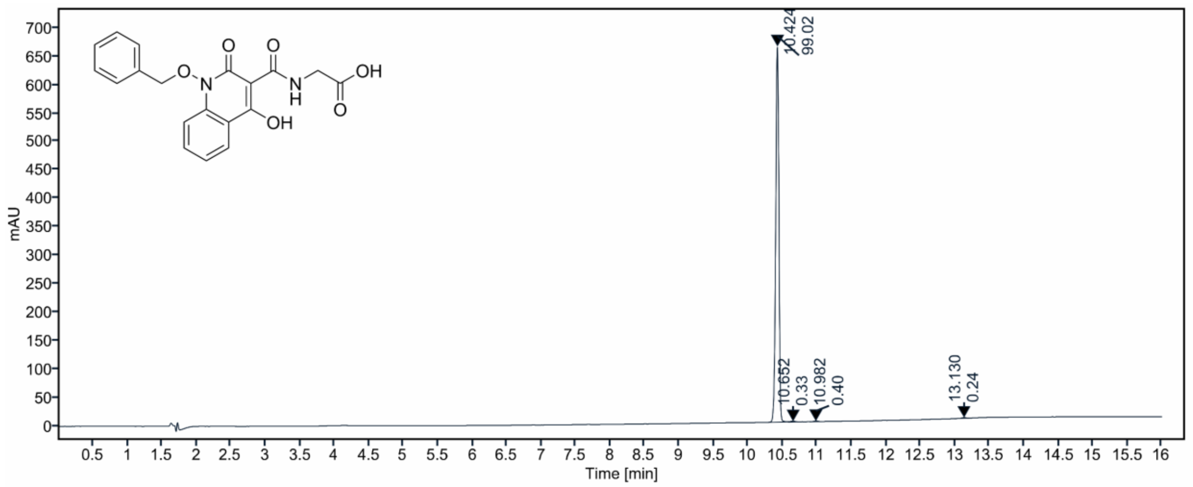

(1-(Cyclopentylmethoxy)-4-hydroxy-2-oxo-1,2-dihydroquinoline-3-carbonyl)glycine (48)

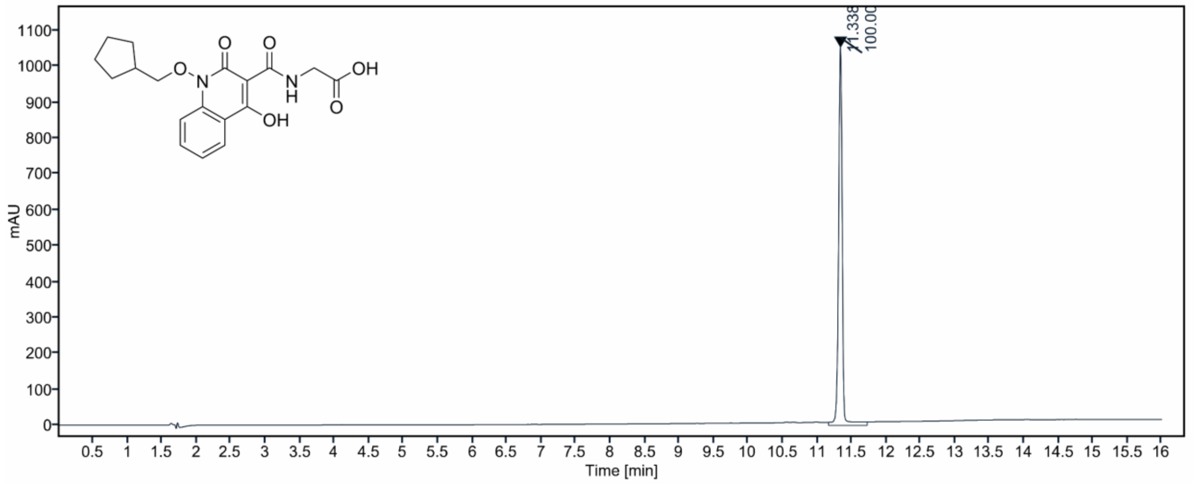

(1-(Cyclohexylmethoxy)-4-hydroxy-2-oxo-1,2-dihydroquinoline-3-carbonyl)glycine (49)

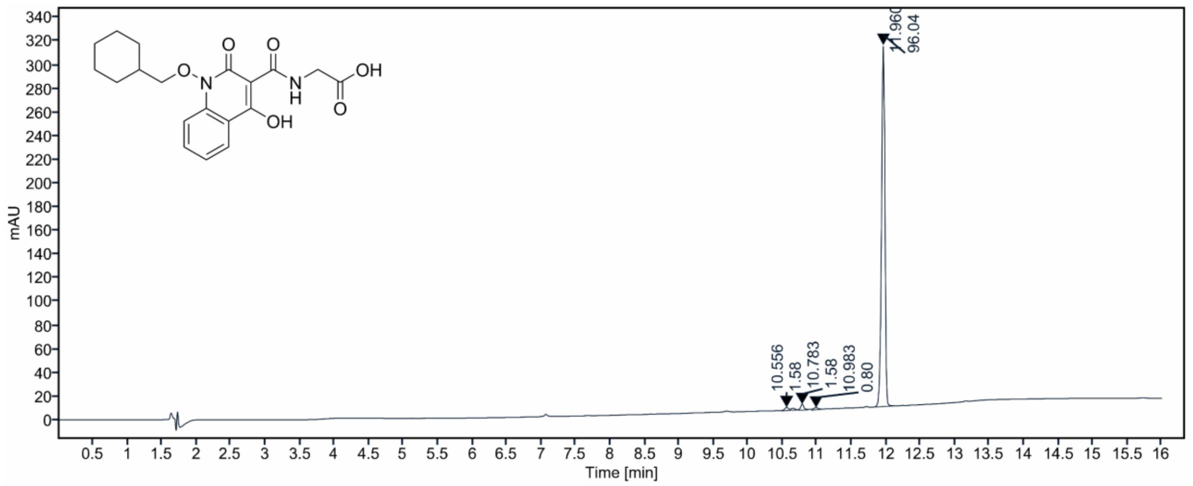

(4-Hydroxy-1-(naphthalen-1-ylmethoxy)-2-oxo-1,2-dihydroquinoline-3-carbonyl)glycine (50)

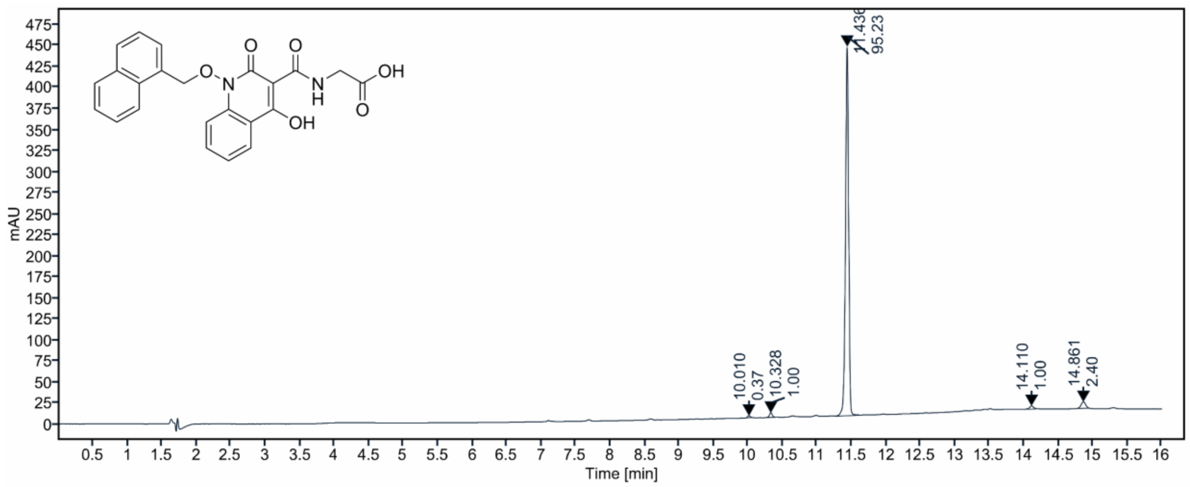

(4-Hydroxy-1-(neopentyloxy)-2-oxo-1,2-dihydroquinoline-3-carbonyl)glycine (51)

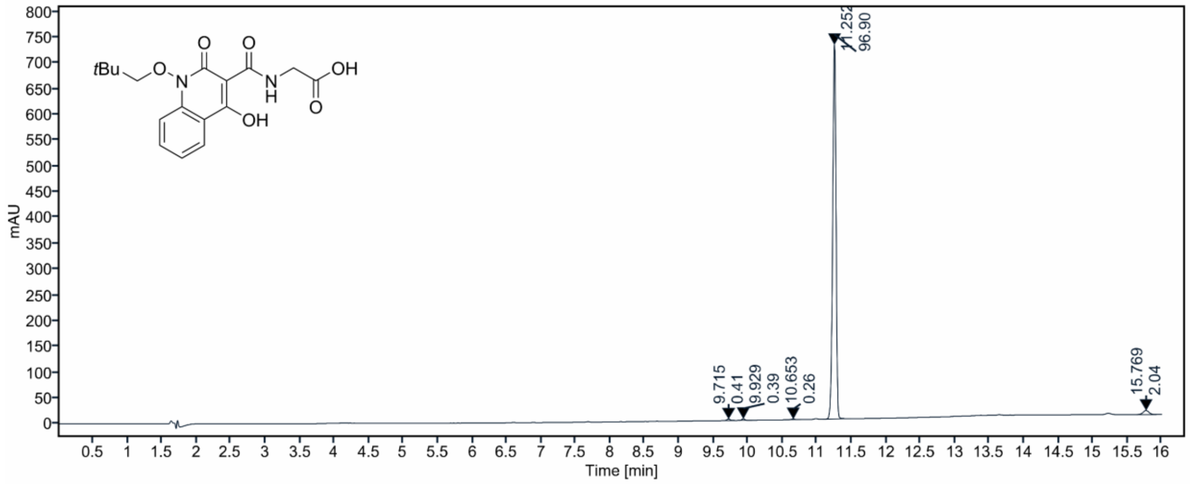

(1-(Cyclopropylmethyl)-4-hydroxy-2-oxo-1,2-dihydroquinoline-3-carbonyl)glycine (52)

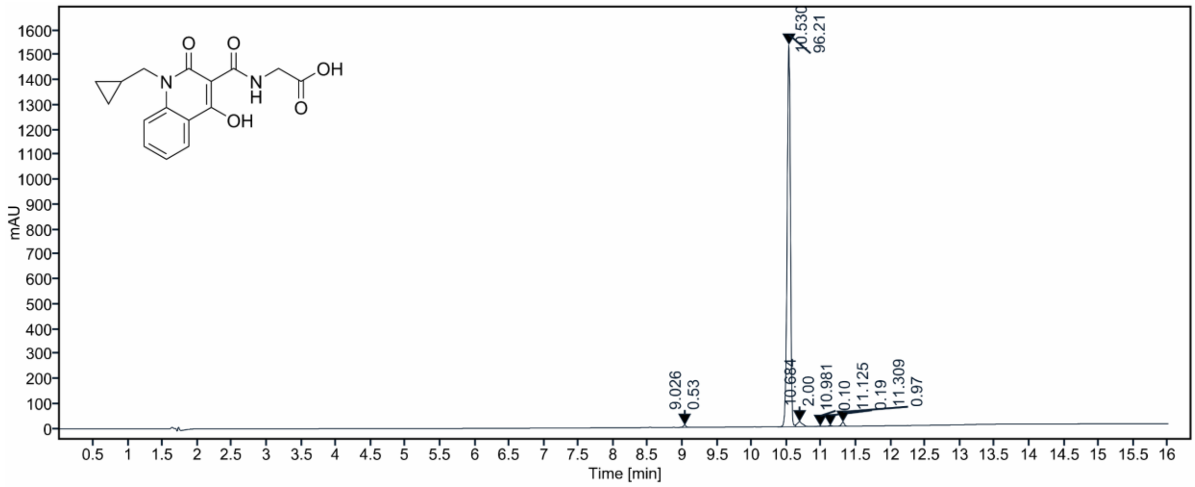

(1-(2-Cyclopropylethyl)-4-hydroxy-2-oxo-1,2-dihydroquinoline-3-carbonyl)glycine (53)

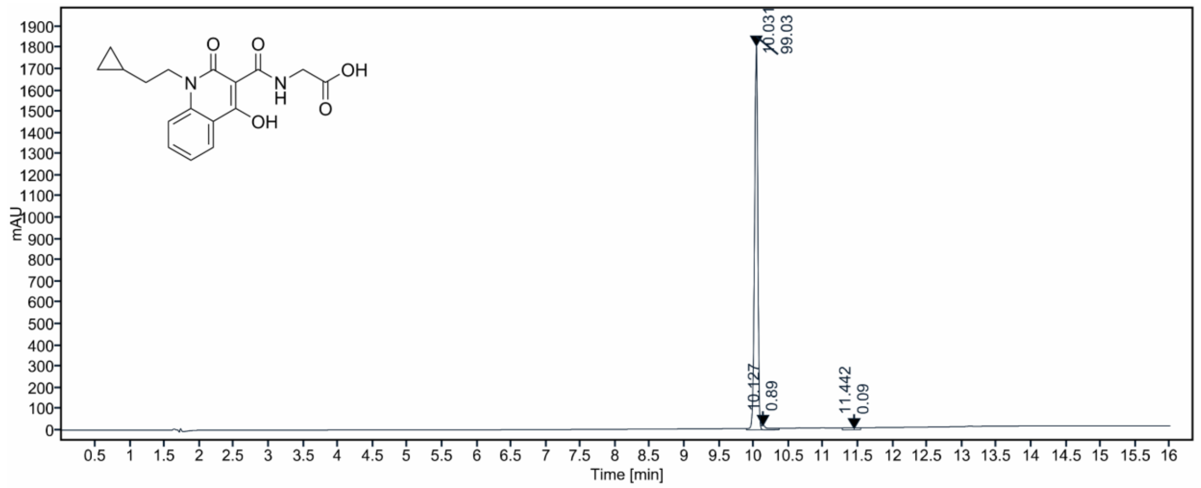

(1-(Cyclopropylmethoxy)-4-hydroxy-2-oxo-1,2,5,6-tetrahydropyridine-3-carbonyl)glycine (54)

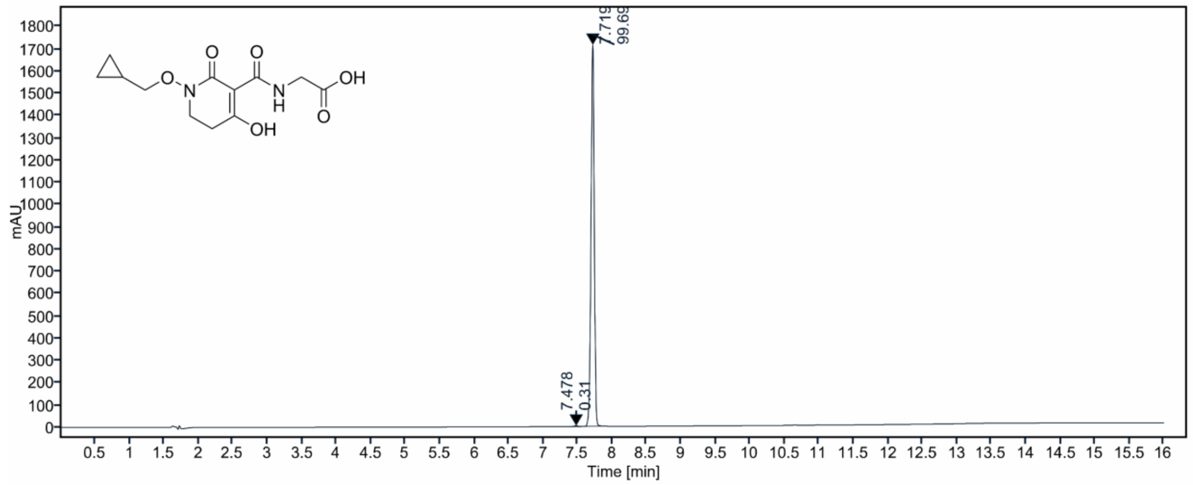

Ethyl 2-(4-hydroxy-2-oxo-1-(pyridin-2-ylmethoxy)-1,2-dihydroquinoline-3-carboxamido)-2-methylpropanoate (57a)

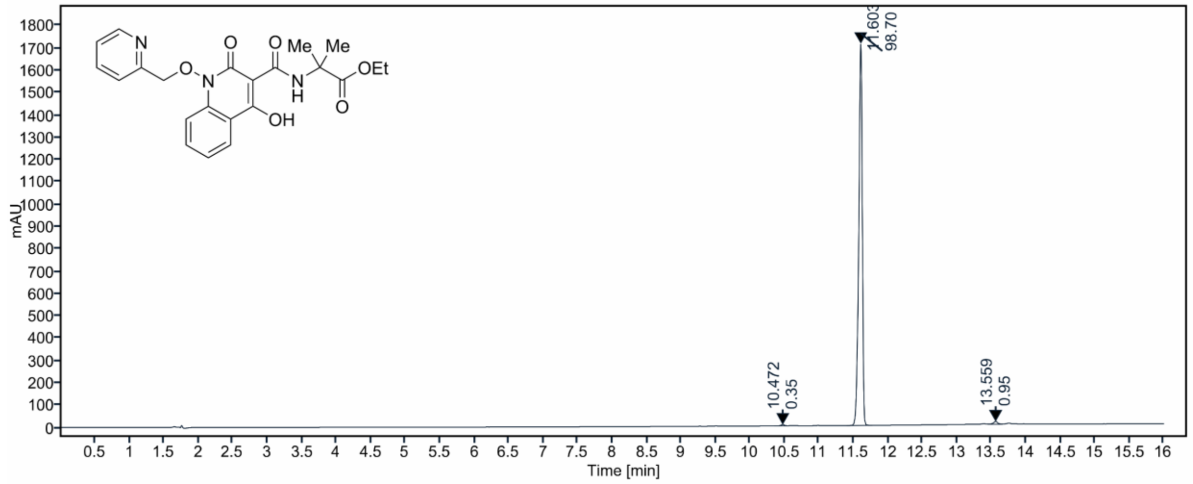

2-(4-Hydroxy-2-oxo-1-(pyridin-2-ylmethoxy)-1,2-dihydroquinoline-3-carboxamido)-2-methylpropanoic acid (57)

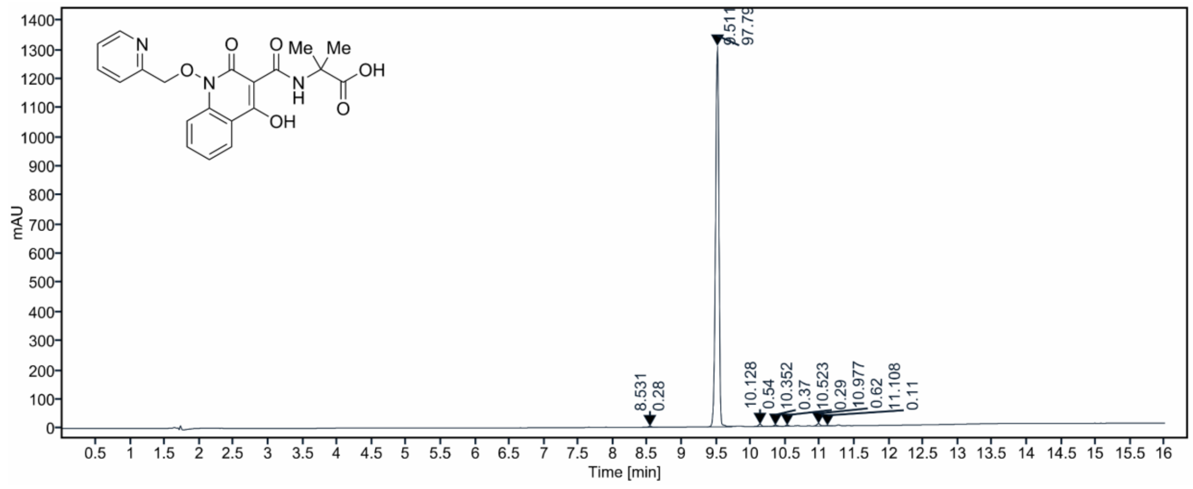

Ethyl 2-(4-hydroxy-2-oxo-1-(thiazol-4-ylmethoxy)-1,2-dihydroquinoline-3-carboxamido)-2-methylpropanoate (58a)

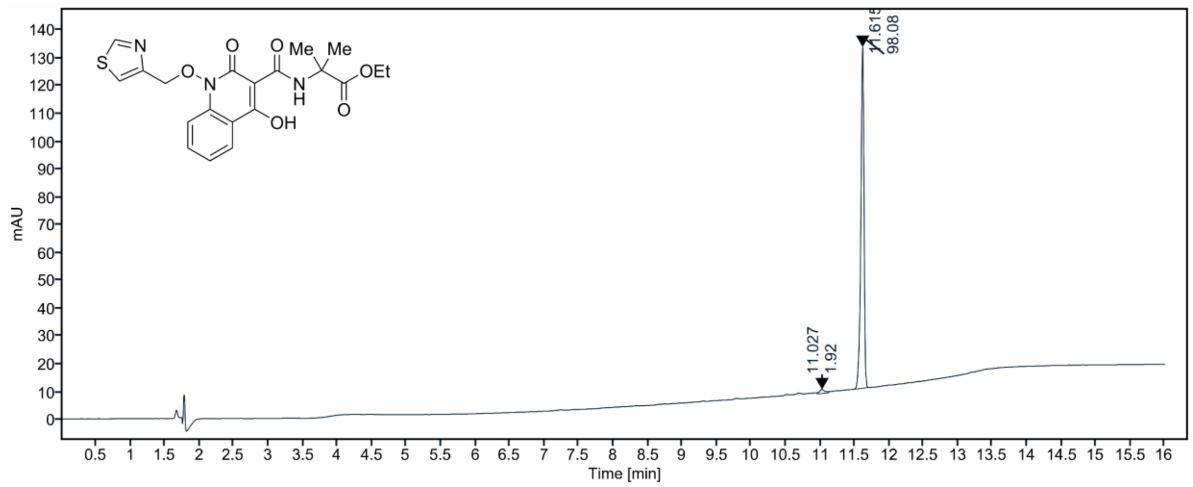

2-(4-Hydroxy-2-oxo-1-(thiazol-4-ylmethoxy)-1,2-dihydroquinoline-3-carboxamido)-2-methylpropanoic acid (58)

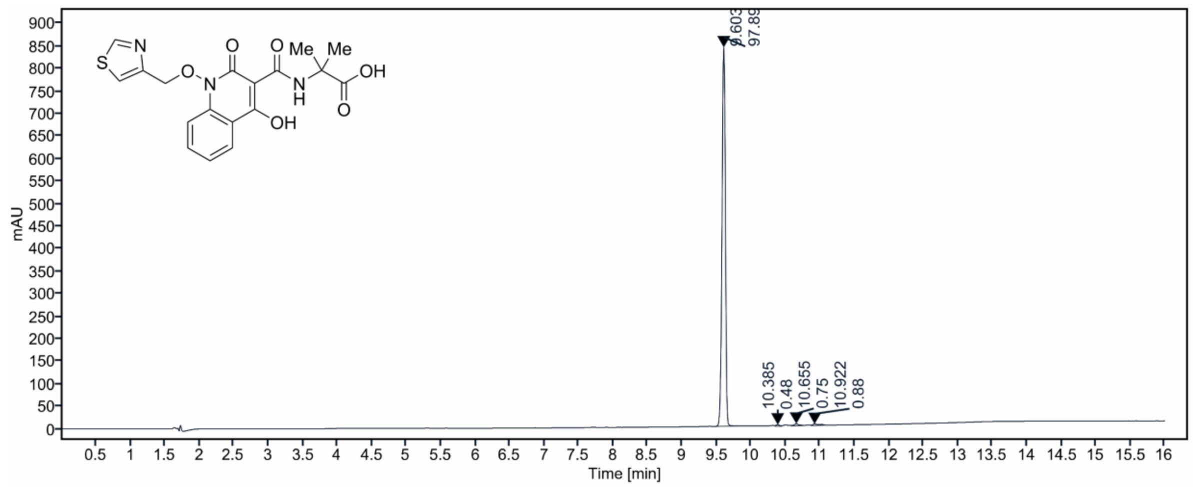

8. References

(1) Buszewski, B.; Noga, S. Hydrophilic interaction liquid chromatography (HILIC)—a powerful separation technique. *Anal. Bioanal. Chem.* **2012**, *402* (1), 231-247. DOI: 10.1007/s00216-011-5308-5.

(2) Dambrova, M.; Liepinsh, E.; Kalvinsh, I. Mildronate: cardioprotective action through carnitine-lowering effect. *Trends Cardiovasc. Med.* **2002**, *12* (6), 275-279. DOI: 10.1016/S1050-1738(02)00175-5.

(3) Rydzik, A. M.; Chowdhury, R.; Kochan, G. T.; Williams, S. T.; McDonough, M. A.; Kawamura, A.; Schofield, C. J. Modulating carnitine levels by targeting its biosynthesis – selective inhibition of γ-butyrobetaine hydroxylase. *Chem. Sci.* **2014**, *5* (5), 1765-1771. DOI: 10.1039/C4SC00020J.

(4) Parmar, D. V.; Kansagra, K. A.; Patel, J. C.; Joshi, S. N.; Sharma, N. S.; Shelat, A. D.; Patel, N. B.; Nakrani, V. B.; Shaikh, F. A.; Patel, H. V.; ZYAN1 Trial Investigators. Outcomes of Desidustat treatment in people with anemia and chronic kidney disease: a phase 2 study. *Am. J. Nephrol.* **2019**, *49* (6), 470-478. DOI: 10.1159/000500232.

(5) Ogoshi, Y.; Matsui, T.; Mitani, I.; Yokota, M.; Terashita, M.; Motoda, D.; Ueyama, K.; Hotta, T.; Ito, T.; Hase, Y.; Fukui, K.; Deai, K.; Yoshiuchi, H.; Ito, S.; Abe, H. Discovery of JTZ-951: a HIF prolyl hydroxylase inhibitor for the treatment of renal anemia. *ACS Med. Chem. Lett.* **2017**, *8* (12), 1320-1325. DOI: 10.1021/acsmedchemlett.7b00404.

(6) Pergola, P. E.; Spinowitz, B. S.; Hartman, C. S.; Maroni, B. J.; Haase, V. H. Vadadustat, a novel oral HIF stabilizer, provides effective anemia treatment in nondialysis-dependent chronic kidney disease. *Kidney Int.* **2016**, *90* (5), 1115-1122. DOI: 10.1016/j.kint.2016.07.019.

(7) Ariazi, J. L.; Duffy, K. J.; Adams, D. F.; Fitch, D. M.; Luo, L.; Pappalardi, M.; Biju, M.; DiFilippo, E. H.; Shaw, T.; Wiggall, K.; Erickson-Miller, C. Discovery and preclinical characterization of GSK1278863 (Daprodustat), a small molecule Hypoxia Inducible Factor–Prolyl Hydroxylase inhibitor for anemia. *J. Pharmacol. Exp. Ther.* **2017**, *363* (3), 336-347. DOI: 10.1124/jpet.117.242503.

(8) Wu, K.; Zhou, K.; Wang, Y.; Zhou, Y.; Tian, N.; Wu, Y.; Chen, D.; Zhang, D.; Wang, X.; Xu, H.; Zhang, X. Stabilization of HIF-1α by FG-4592 promotes functional recovery and neural protection in experimental spinal cord injury. *Brain Res.* **2016**, *1632*, 19-26. DOI: 10.1016/j.brainres.2015.12.017.

(9) Zhang, J.-H.; Chung, T. D. Y.; Oldenburg, K. R. A simple statistical parameter for use in evaluation and validation of high throughput screening assays. *J. Biomol. Screen.* **1999**, *4* (2), 67-73.

(10) Leung, I. K. H.; Krojer, T. J.; Kochan, G. T.; Henry, L.; von Delft, F.; Claridge, T. D. W.; Oppermann, U.; McDonough, M. A.; Schofield, C. J. Structural and mechanistic studies on γ-butyrobetaine hydroxylase. *Chem. Biol.* **2010**, *17* (12), 1316-1324. DOI: 10.1016/j.chembiol.2010.09.016.

(11) Yeh, T.-L.; Leissing, T. M.; Abboud, M. I.; Thinnes, C. C.; Atasoylu, O.; Holt-Martyn, J. P.; Zhang, D.; Tumber, A.; Lippl, K.; Lohans, C. T.; Leung, I. K. H.; Morcrette, H.; Clifton, I. J.; Claridge, T. D. W.; Kawamura, A.; Flashman, E.; Lu, X.; Ratcliffe, P. J.; Chowdhury, R.; Pugh, C. W.; Schofield, C. J. Molecular and cellular mechanisms of HIF prolyl hydroxylase inhibitors in clinical trials. *Chem. Sci.* **2017**, *8* (11), 7651-7668. DOI: 10.1039/C7SC02103H.

(12) Meiboom, S.; Gill, D. Modified spin-echo method for measuring nuclear relaxation times. *Rev. Sci. Instrum.* **1958**, *29* (8), 688-691. DOI: 10.1063/1.1716296.

(13) Khan, A.; Leśniak, R. K.; Brem, J.; Rydzik, A. M.; Choi, H.; Leung, I. K. H.; McDonough, M. A.; Schofield, C. J.; Claridge, T. D. W. Development and application of ligand-based NMR screening assays for γ-butyrobetaine hydroxylase. *MedChemComm* **2016**, *7* (5), 873-880. DOI: 10.1039/C6MD00004E.

(14) Leśniak, R. K.; Rydzik, A. M.; Kamps, J. J. A. G.; Kahn, A.; Claridge, T. D. W.; Schofield, C. J. ^{19}F NMR studies on γ-butyrobetaine hydroxylase provide mechanistic insights and suggest a dual inhibition mode. *Chem. Commun.* **2019**, *55* (98), 14717-14720. DOI: 10.1039/C9CC06466D.

(15) Carpino, L. A. 1-Hydroxy-7-azabenzotriazole. An efficient peptide coupling additive. *J. Am. Chem. Soc.* **1993**, *115* (10), 4397-4398. DOI: 10.1021/ja00063a082.

(16) Chowdhury, R.; Candela-Lena, J. I.; Chan, M. C.; Greenald, D. J.; Yeoh, K. K.; Tian, Y.-M.; McDonough, M. A.; Tumber, A.; Rose, N. R.; Conejo-Garcia, A.; Demetriades, M.; Mathavan, S.; Kawamura, A.; Lee, M. K.; van Eeden, F.; Pugh, C. W.; Ratcliffe, P. J.; Schofield, C. J. Selective small molecule probes for the hypoxia inducible factor (HIF) prolyl hydroxylases. *ACS Chem. Biol.* **2013**, *8* (7), 1488-1496. DOI: 10.1021/cb400088q.

(17) Sugimura, T.; Hagiya, K. Di-2-methoxyethyl azodicarboxylate (DMEAD): an inexpensive and separation-friendly alternative reagent for the Mitsunobu Reaction. *Chem. Lett.* **2007**, *36* (4), 566-567. DOI: 10.1246/cl.2007.566.

(18) Holt-Martyn, J. P.; Chowdhury, R.; Tumber, A.; Yeh, T.-L.; Abboud, M. I.; Lippl, K.; Lohans, C. T.; Langley, G. W.; Figg Jr., W.; McDonough, M. A.; Pugh, C. W.; Ratcliffe, P. J.; Schofield, C. J. Structure-activity relationship and crystallographic studies on 4-hydroxypyrimidine HIF Prolyl Hydroxylase Domain inhibitors. *ChemMedChem* **2020**, *15* (3), 270-273. DOI: 10.1002/cmdc.201900557.

(19) Brewitz, L.; Tumber, A.; Pfeffer, I.; McDonough, M. A.; Schofield, C. J. Aspartate/asparagine-β-hydroxylase: a high-throughput mass spectrometric assay for discovery of small molecule inhibitors. *Sci. Rep.* **2020**, *10* (1), 8650. DOI: 10.1038/s41598-020-65123-9.

(20) Hutchinson, S. E.; Leveridge, M. V.; Heathcote, M. L.; Francis, P.; Williams, L.; Gee, M.; Munoz-Muriedas, J.; Leavens, B.; Shillings, A.; Jones, E.; Homes, P.; Baddeley, S.; Chung, C.-w.; Bridges, A.; Argyrou, A. Enabling lead discovery for histone lysine demethylases by high-throughput RapidFire mass spectrometry. *J. Biomol. Screen.* **2012**, *17* (1), 39-48. DOI: 10.1177/1087057111416660.

(21) Tumber, A.; Salah, E.; Brewitz, L.; Corner, T. P.; Schofield, C. J. Kinetic and inhibition studies on human Jumonji-C (JmjC) domain-containing protein 5. *RSC Chem. Biol.* **2023**, *4* (6), 399-413. DOI: 10.1039/d2cb00249c.

(22) Nakashima, Y.; Brewitz, L.; Tumber, A.; Salah, E.; Schofield, C. J. 2-Oxoglutarate derivatives can selectively enhance or inhibit the activity of human oxygenases. *Nat. Commun.* **2021**, *12* (1), 6478. DOI: 10.1038/s41467-021-26673-2.

(23) Desai, R. C.; Sharma, R.; Pandya, V.; Shah, K.; Patel, S.; Chauhan, R.; Nair, R.; Joshi, V.; Patel, M.; Shukla, M. Process for the preparation of quinolone based compounds. US 2019/0359574 A1, **2019**.

(24) Corner, T. P.; Salah, E.; Tumber, A.; Kaur, S.; Nakashima, Y.; Allen, M. D.; Schnaubelt, L. I.; Fiorini, G.; Brewitz, L.; Schofield, C. J. Crystallographic and selectivity studies on the approved HIF prolyl hydroxylase inhibitors Desidustat and Enarodustat. *ChemMedChem* **2024**, *19* (24), e202400504. DOI: 10.1002/cmdc.202400504.

(25) Desai, R., C.; Pandya, V.; Patel, P., R. Novel quinolone derivatives. WO 2014/102818 A1, **2014**.

(26) Duffy, K. J.; Fitch, D. M.; Jin, J.; Liu, R.; Shaw, A. N.; Wiggall, K. Preparation of *N*-substituted pyrimidine-trione amino acid derivatives as prolyl hydroxylase inhibitors. WO 2007/150011 A2, **2007**.

(27) Kawamoto, R. M. Prolyl hydroxylase inhibitors and methods of use. US 2007/0299086 A1, **2007**.

(28) Arend, M. P.; Flippin, L. A.; Guenzler-Pukall, V.; Ho, W.-B.; Turtle, E. D.; Du, X. Nitrogen-containing heteroaryl compounds and their use in increasing endogenous erythropoietin. WO 2004/108681 A1, **2004**.

(29) Mitani, I.; Ogoshi, Y.; Matsui, T.; Yokota, M.; Terashita, M.; Motoda, D.; Ueyama, K.; Abe, H.; Hotta, T.; Ito, T. Triazolopyridine compound, and action thereof as prolyl hydroxylase inhibitor or erythropoietin production-inducing agent. US 2020/0017492 A1, **2012**.

(30) Clark, R. L.; Clements, C. J.; Barrett, M. P.; Mackay, S. P.; Rathnam, R. P.; Owusu-Dapaah, G.; Spencer, J.; Huggan, J. K. Identification and development of the 1,4-benzodiazepin-2-one and quinazoline-2,4-dione scaffolds as submicromolar inhibitors of HAT. *Biorg. Med. Chem.* **2012**, *20* (20), 6019-6033. DOI: 10.1016/j.bmc.2012.08.049.

(31) Martínez, A.; Gil, C.; Palomo Ruiz, V.; Pérez, C.; Pérez Fernández, D. I.; Reyes Rodríguez, J. A. Heterocyclic GSK-3 allosteric modulators. EP 2769720 B1, **2013**.

(32) Chai, D.; Colon, M.; Duffy, K.; Fitch, D.; Tedesco, R.; Zimmerman, M. Prolyl hydroxylase antagonists. WO 2007/038571 A2, **2007**.
